# Supplementary material for: Complex interplay between RAS GTPases and RASSF effectors regulates subcellular localization of YAP
Source: EMBO Rep. 2024 Jul 15;25(8):22. doi: 10.1038/s44319-024-00203-9 (PMC11316025; doi:10.1038/s44319-024-00203-9)
Supplement: Supplementary file 1 — Appendix [file 44319_2024_203_MOESM1_ESM.pdf]

Appendix

Appendix Figures 2
Appendix Figure S1 2
Appendix Figure S2 4
Appendix Figure S3 6
Appendix Figure S4 8
Appendix Figure S5 10
Appendix Figure S6 12

Appendix Figures

Appendix Figure S1

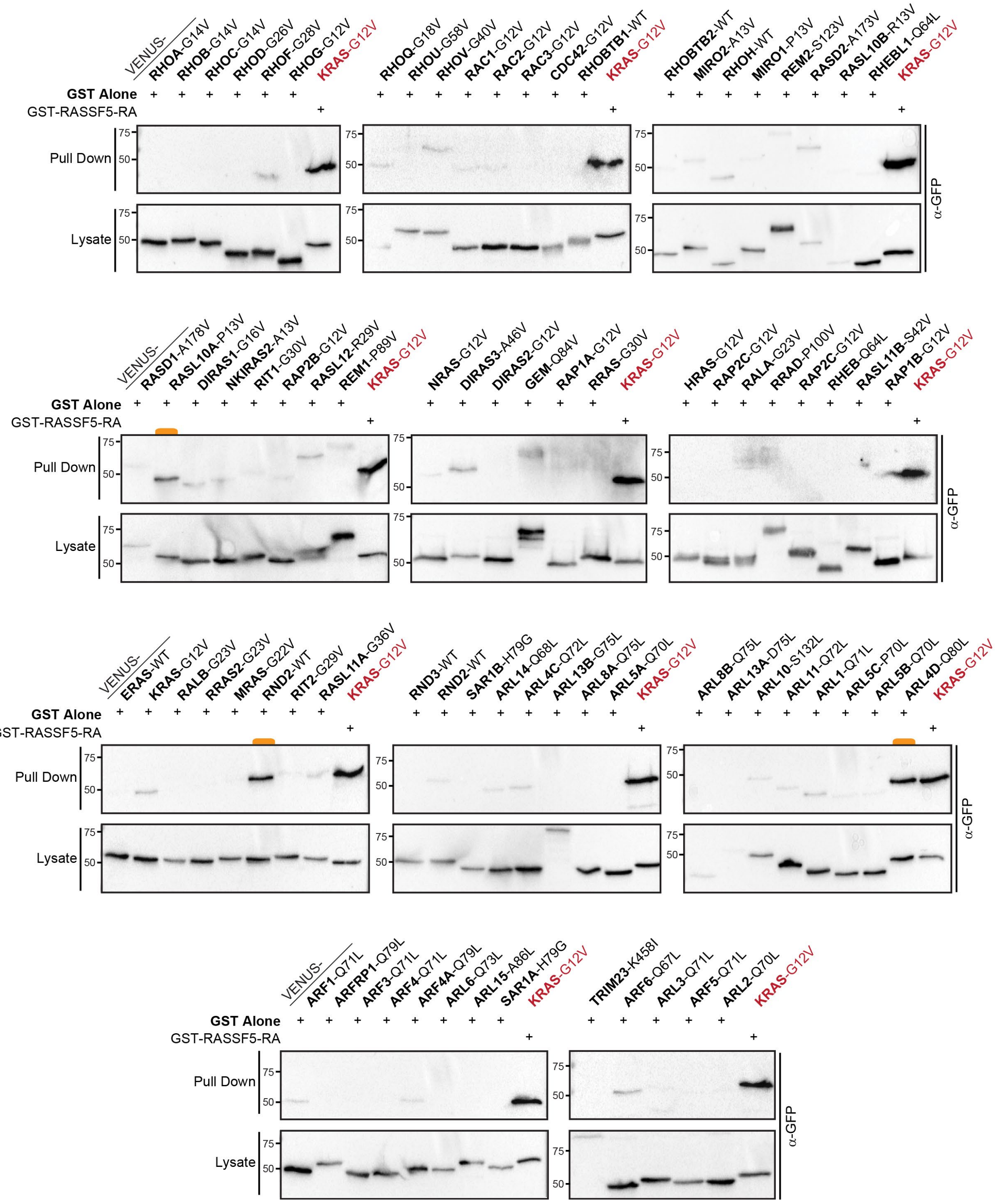

Appendix Figure S1

Precipitation of VENUS-tagged, mutationally activated RAS, RHO and ARF GTPase variants by recombinantly purified GST alone, used as a control for non-specific binding. Blots include RASSF5-RA:KRAS-G12V as a positive control to normalize between experiments. Interacting bands marked with orange on these representative blots (above) were consistently precipitated by GST alone across multiple experiments (RASL10A, RND2, and ARL4D).

Appendix Figure S2

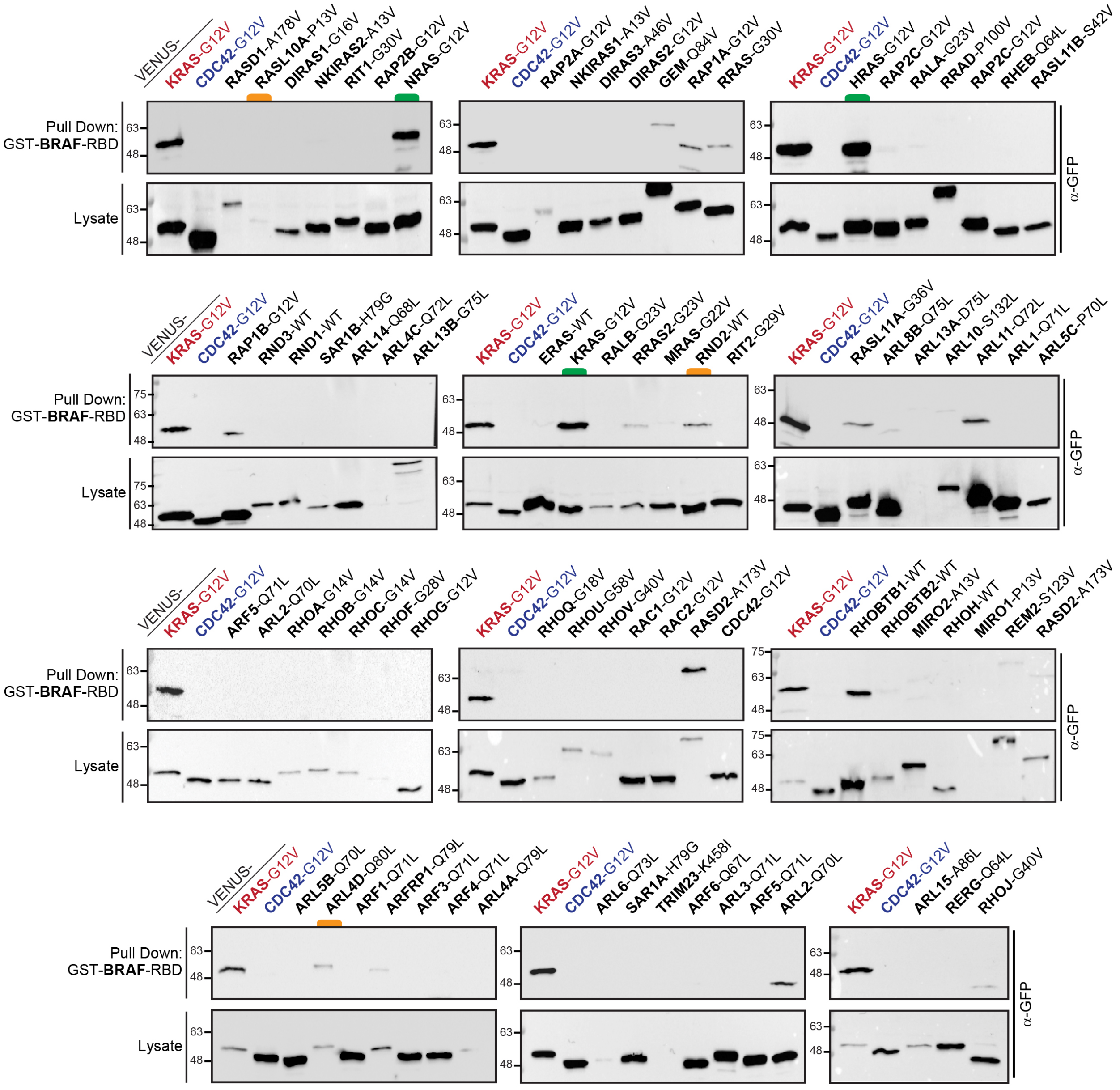

Appendix Figure S2

Precipitation of VENUS-tagged, mutationally activated RAS, RHO and ARF GTPase variants by recombinantly purified GST-BRAF RBD. Each blot includes BRAF:KRAS-G12V as a positive control to normalize between experiments, and BRAF:CDC42-G12V as a negative control. Interacting bands marked with orange on these representative blots (above) were consistently precipitated by GST alone and should be considered non-specific. Bands marked with green were precipitated robustly across multiple experiments and represent candidate GTPase interactors of the BRAF RBD domain.

Appendix Figure S3

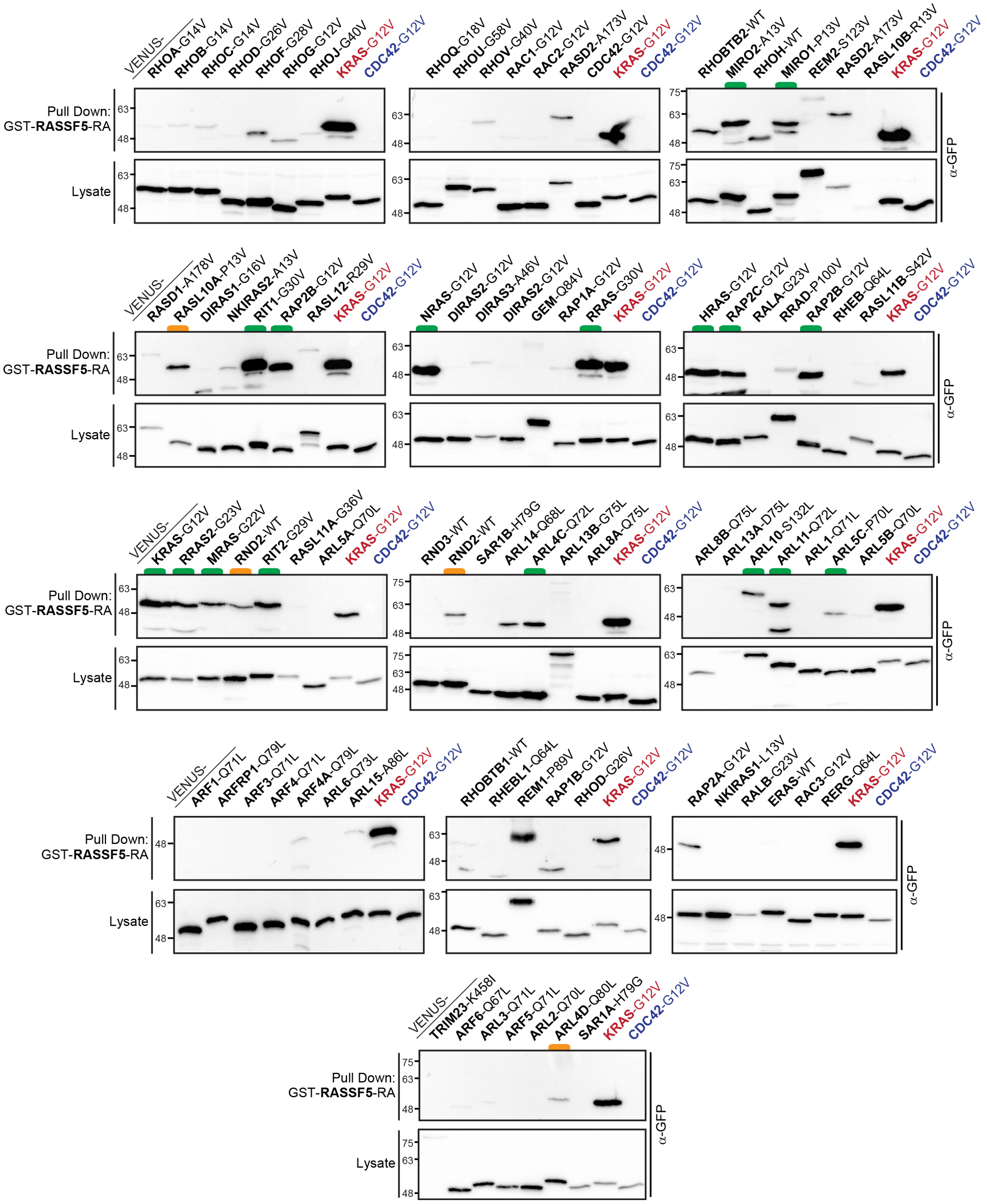

Appendix Figure S3

Precipitation of VENUS-tagged, mutationally activated RAS, RHO and ARF GTPase variants by recombinantly purified GST-RASSF5 RA. Each blot includes RASSF5:KRAS-G12V as a positive control to normalize between experiments, and RASSF5:CDC42-G12V as a negative control. Interacting bands marked with orange on these representative blots (above) were consistently precipitated by GST alone and should be considered non-specific. Bands marked with green were precipitated robustly across multiple experiments and represent candidate GTPase interactors of the RASSF5 RA domain.

Appendix Figure S4

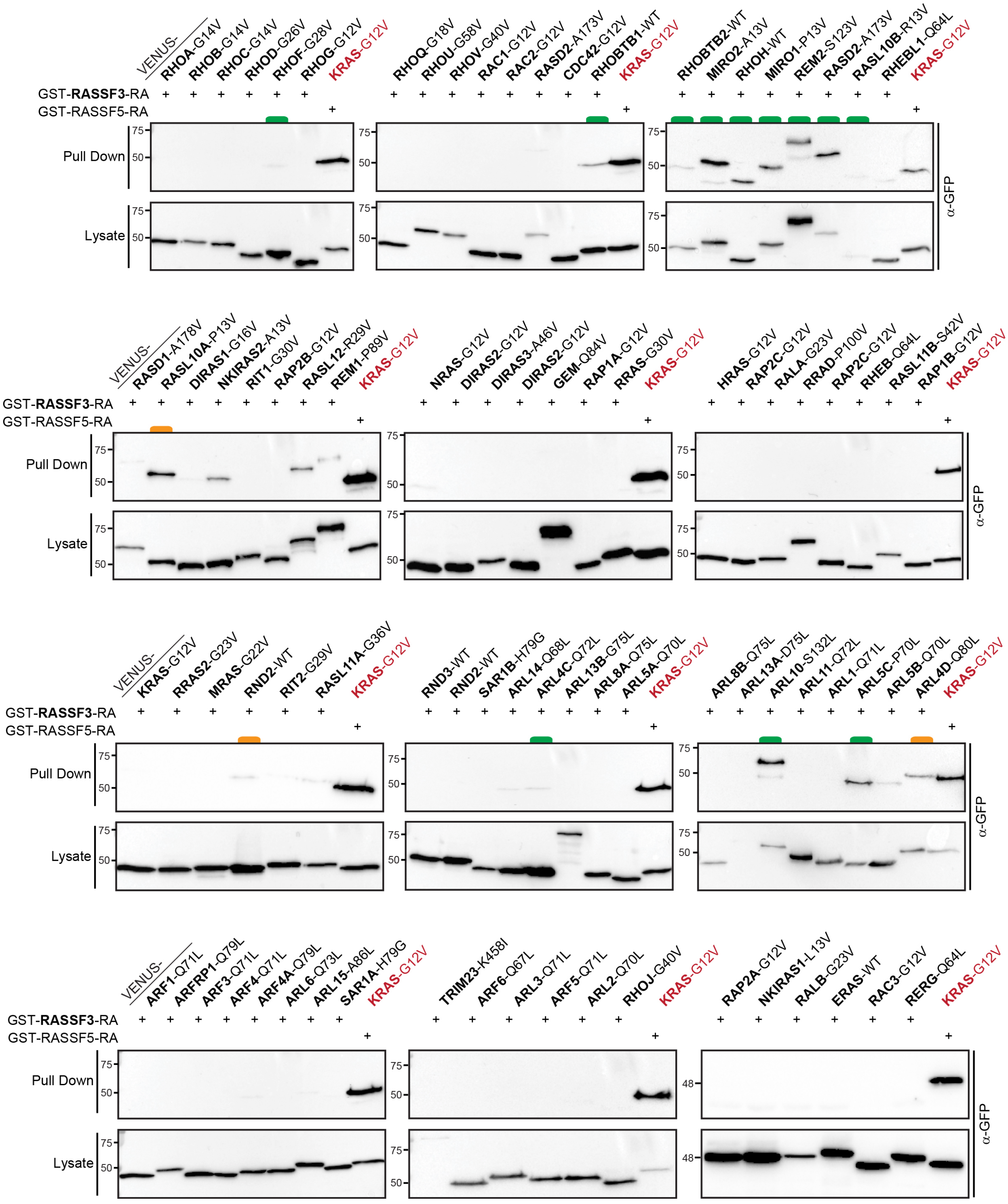

Appendix Figure S4

Precipitation of VENUS-tagged, mutationally activated RAS, RHO and ARF GTPase variants by recombinantly purified GST-RASSF3 RA. Each blot includes RASSF5:KRAS-G12V as a positive control to normalize between experiments, as there were no previously identified small GTPases known to complex with RASSF3. Interacting bands marked with orange on these representative blots (above) were consistently precipitated by GST alone and should be considered non-specific. Bands marked with green were precipitated robustly across multiple experiments and represent candidate GTPase interactors of the RASSF3 RA domain.

Appendix Figure S5

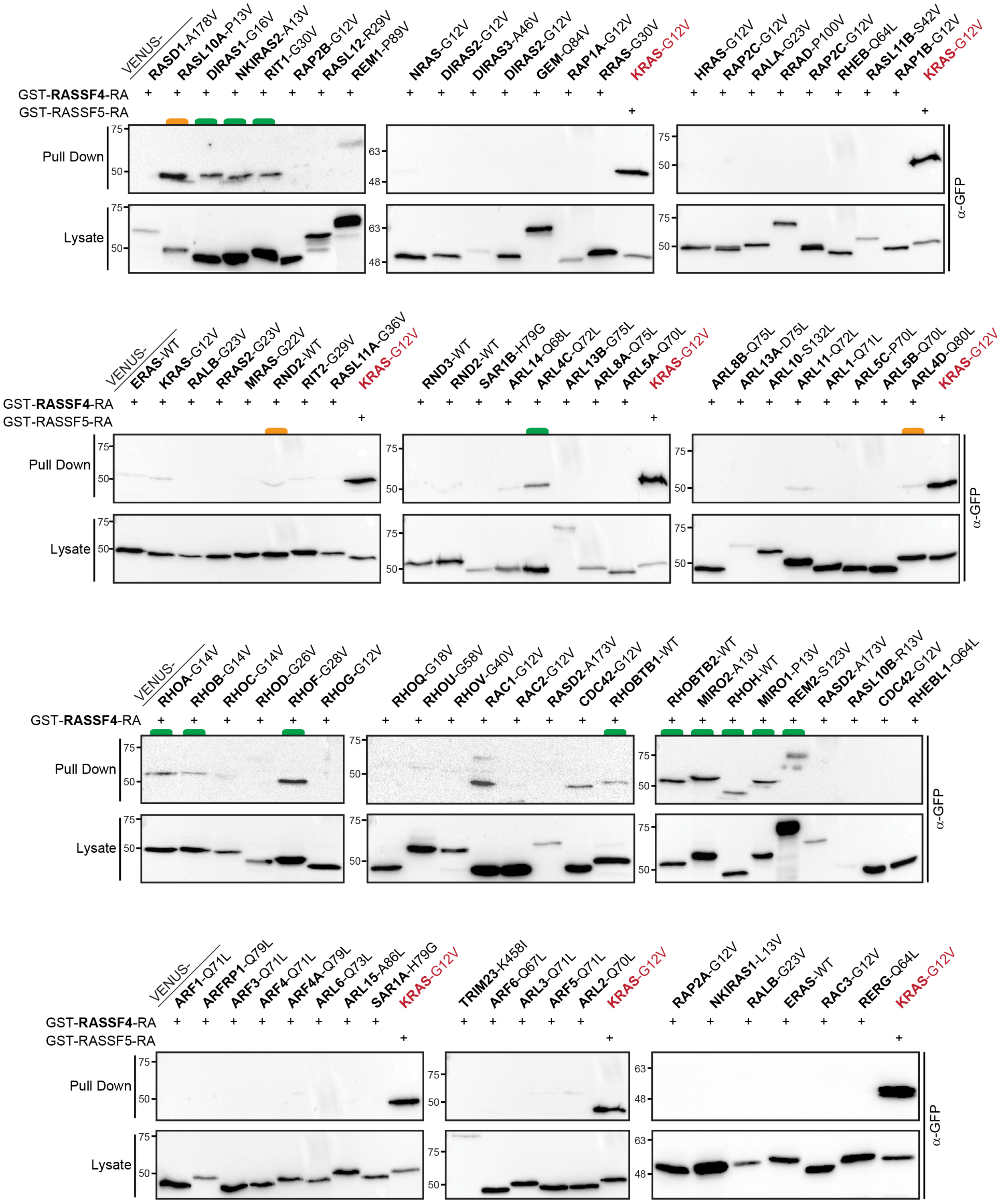

Appendix Figure S5

Precipitation of VENUS-tagged, mutationally activated RAS, RHO and ARF GTPase variants by recombinantly purified GST-RASSF4 RA. Each blot includes RASSF5:KRAS-G12V as a positive control to normalize between experiments, as there were no previously identified small GTPases known to complex with RASSF4. Interacting bands marked with orange on these representative blots (above) were consistently precipitated by GST alone and should be considered non-specific. Bands marked with green were precipitated robustly across multiple experiments and represent candidate GTPase interactors of the RASSF4 RA domain.

Appendix Figure S6

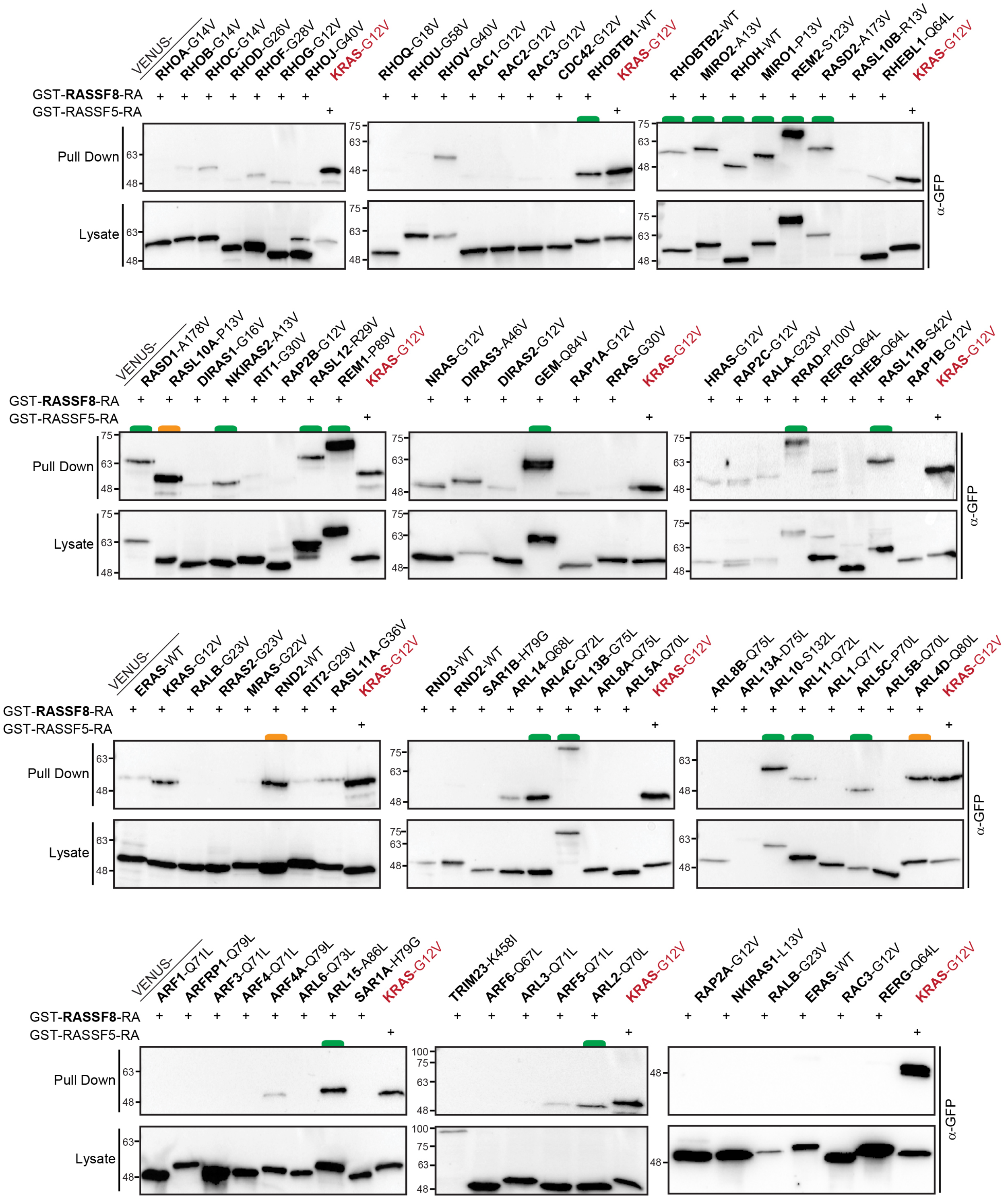

Appendix Figure S6

Precipitation of VENUS-tagged, mutationally activated RAS, RHO and ARF GTPase variants by recombinantly purified GST-RASSF8 RA. Each blot includes RASSF5:KRAS-G12V as a positive control to normalize between experiments, as there were no previously identified small GTPases known to complex with RASSF8. Interacting bands marked with orange on these representative blots (above) were consistently precipitated by GST alone and should be considered non-specific. Bands marked with green were precipitated robustly across multiple experiments and represent candidate GTPase interactors of the RASSF8 RA domain.
