# Supplementary figures and images for: Complex interplay between RAS GTPases and RASSF effectors regulates subcellular localization of YAP (part 1 of 4)
Source: EMBO Rep. 2024 Jul 15;25(8):22. doi: 10.1038/s44319-024-00203-9 (PMC11316025; doi:10.1038/s44319-024-00203-9)

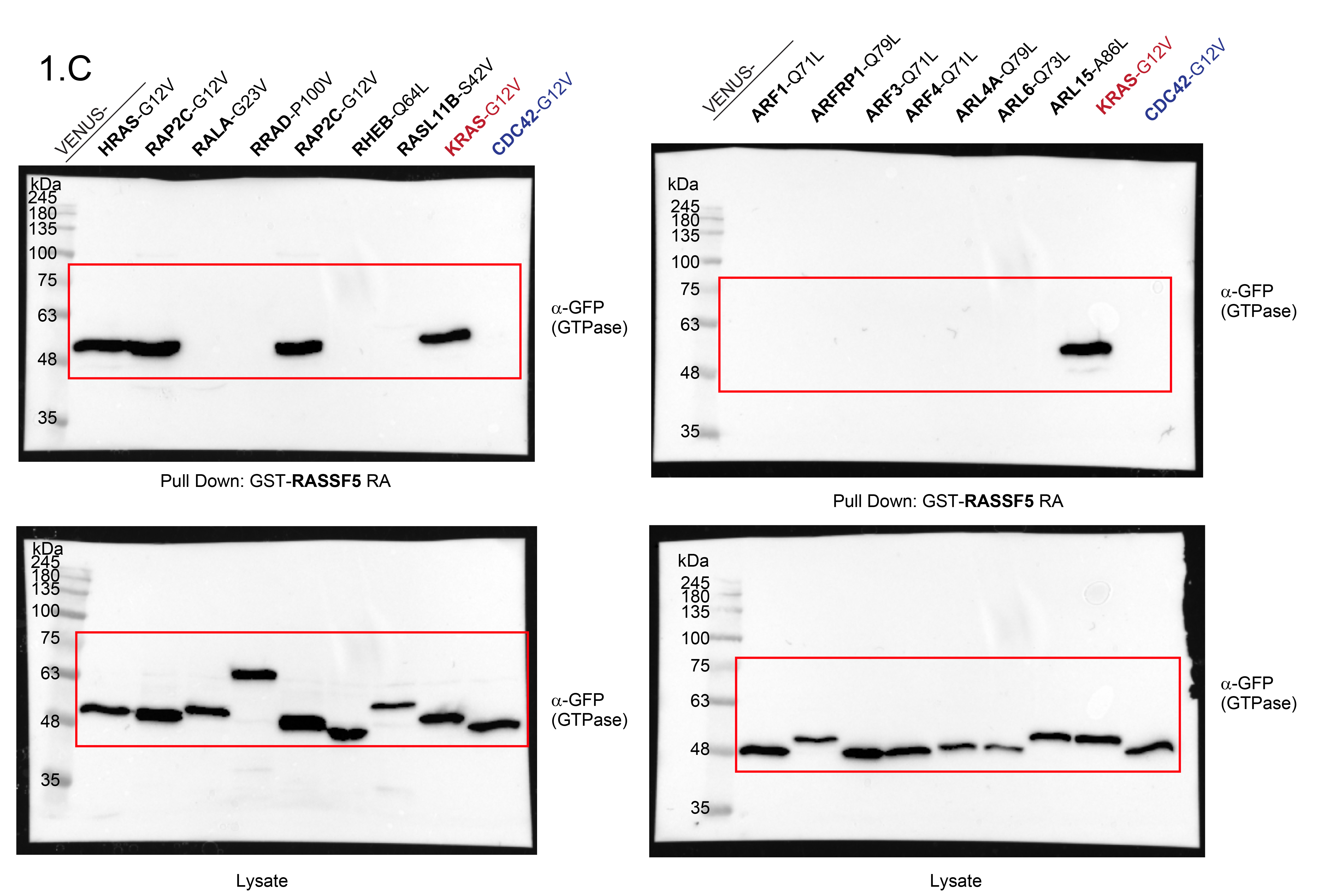

Supplement: Supplementary file 3 — Source data Fig. 1 [file 44319_2024_203_MOESM3_ESM.zip › 1C/1C_Blots.jpg]

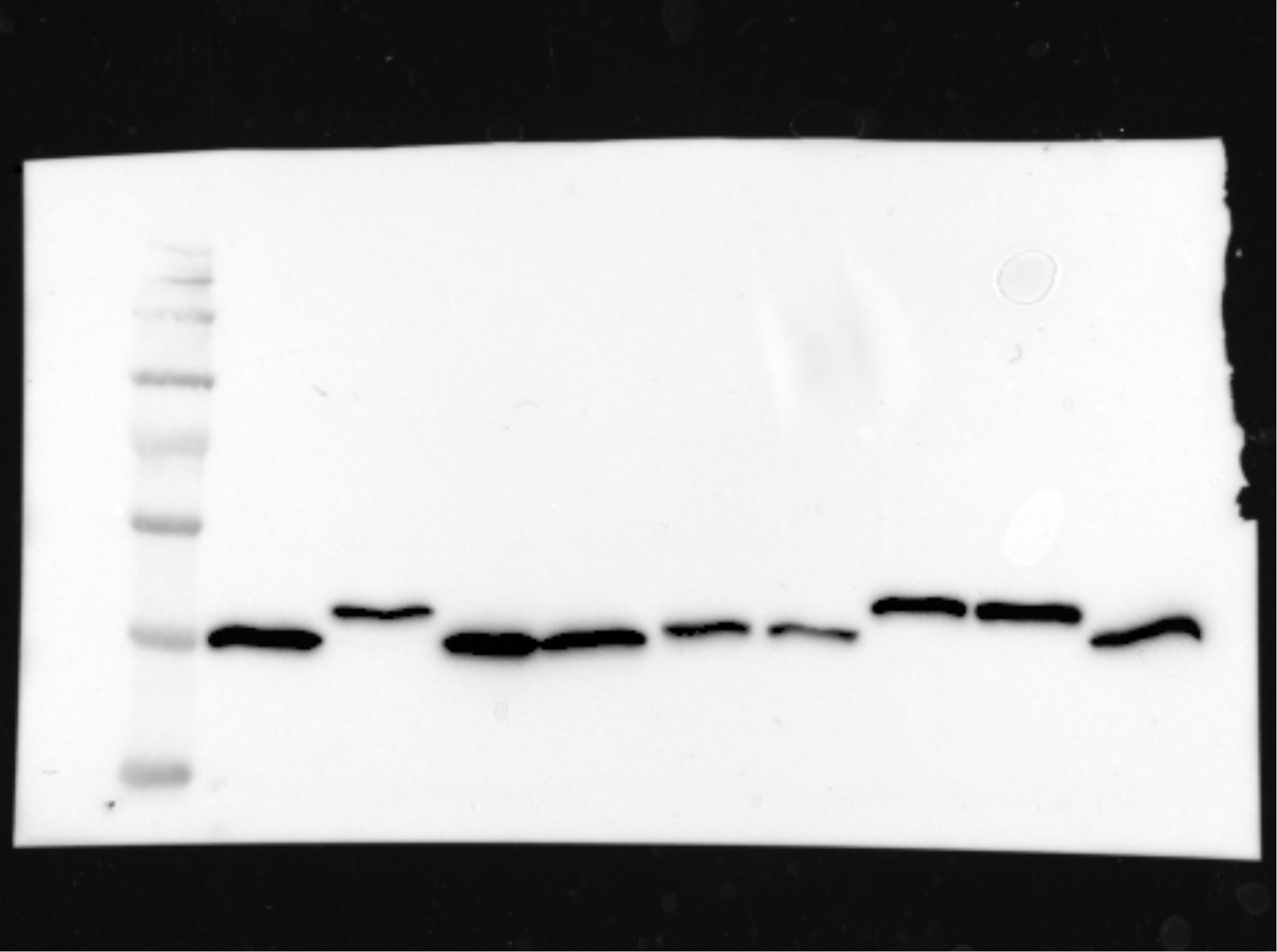

Supplement: Supplementary file 3 — Source data Fig. 1 [file 44319_2024_203_MOESM3_ESM.zip › 1C/ARF1toCDC42 Right/Lysate.jpg]

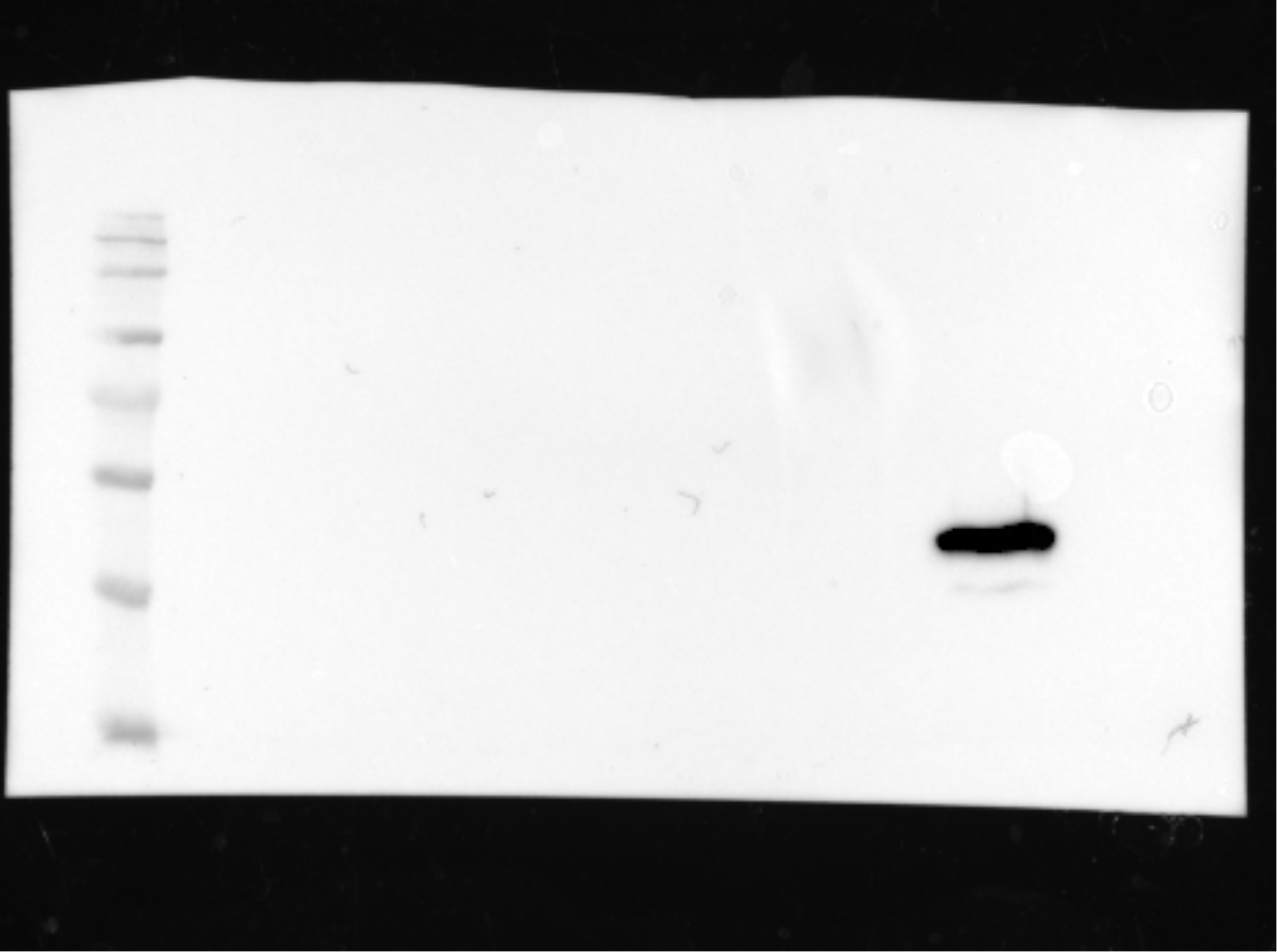

Supplement: Supplementary file 3 — Source data Fig. 1 [file 44319_2024_203_MOESM3_ESM.zip › 1C/ARF1toCDC42 Right/Pull Down GST-RASSF5-RA.jpg]

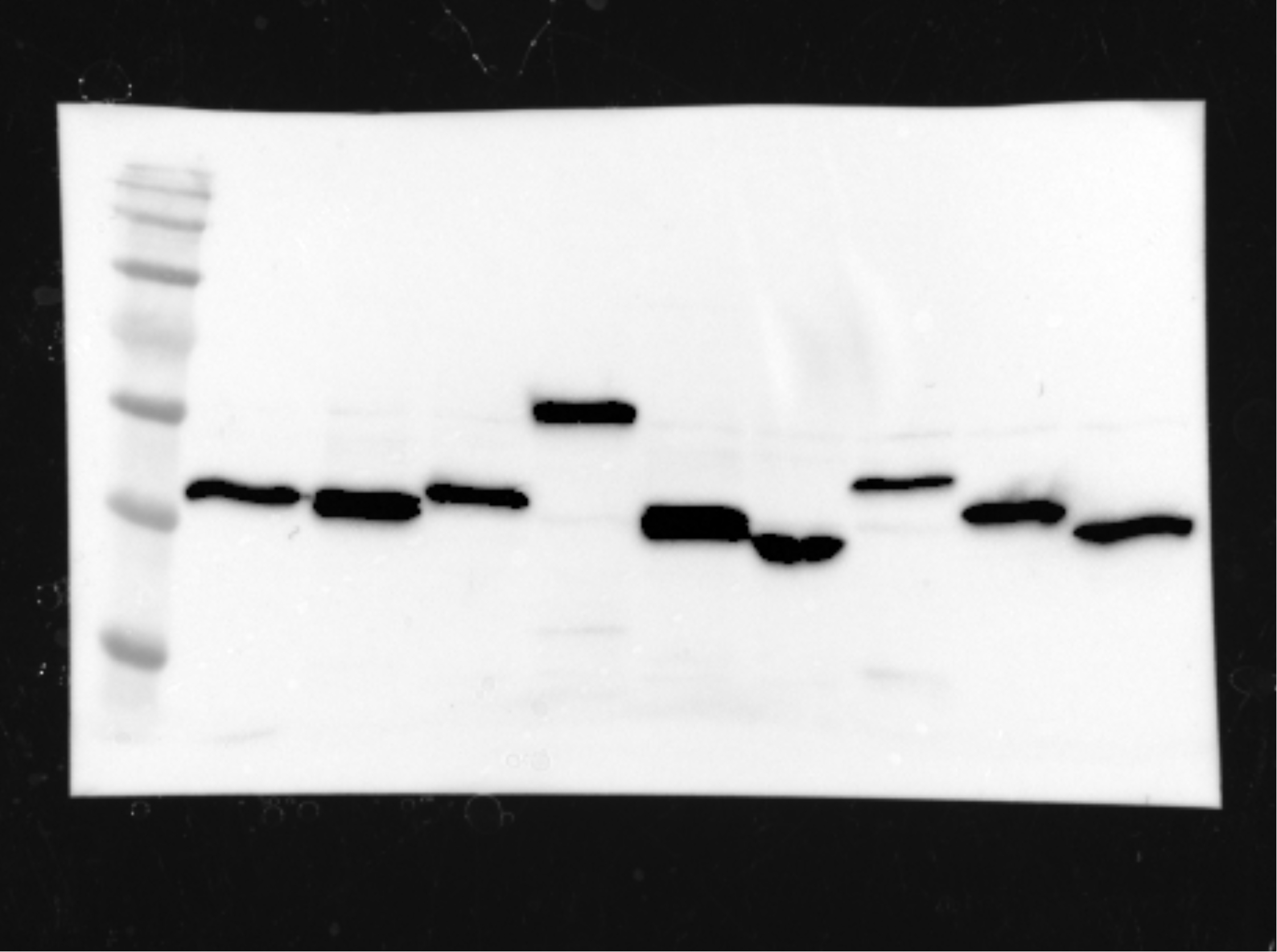

Supplement: Supplementary file 3 — Source data Fig. 1 [file 44319_2024_203_MOESM3_ESM.zip › 1C/HRAStoCDC42 Left/Lysate.jpg]

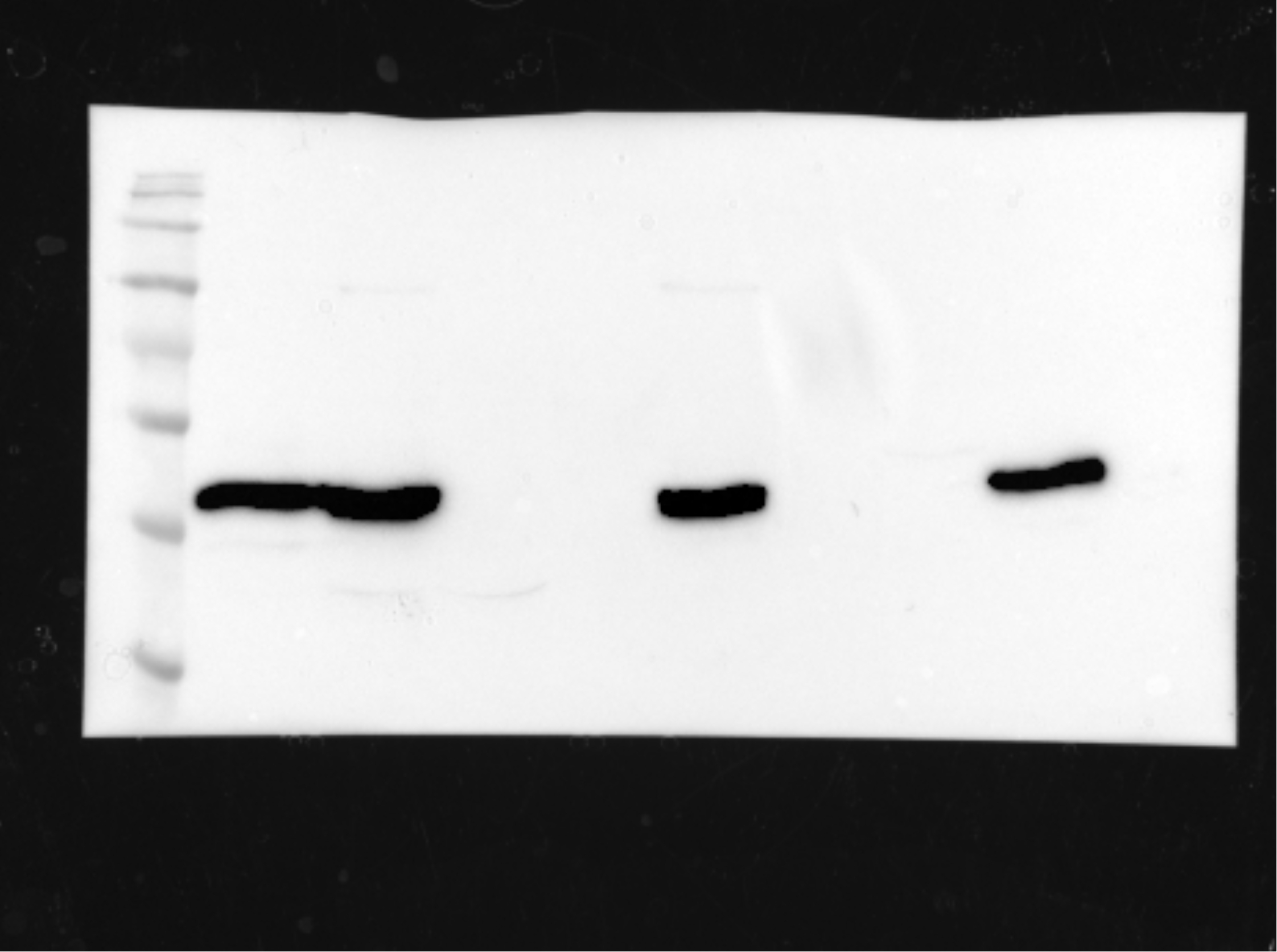

Supplement: Supplementary file 3 — Source data Fig. 1 [file 44319_2024_203_MOESM3_ESM.zip › 1C/HRAStoCDC42 Left/PullDown GST-RASSF5-RA.jpg]

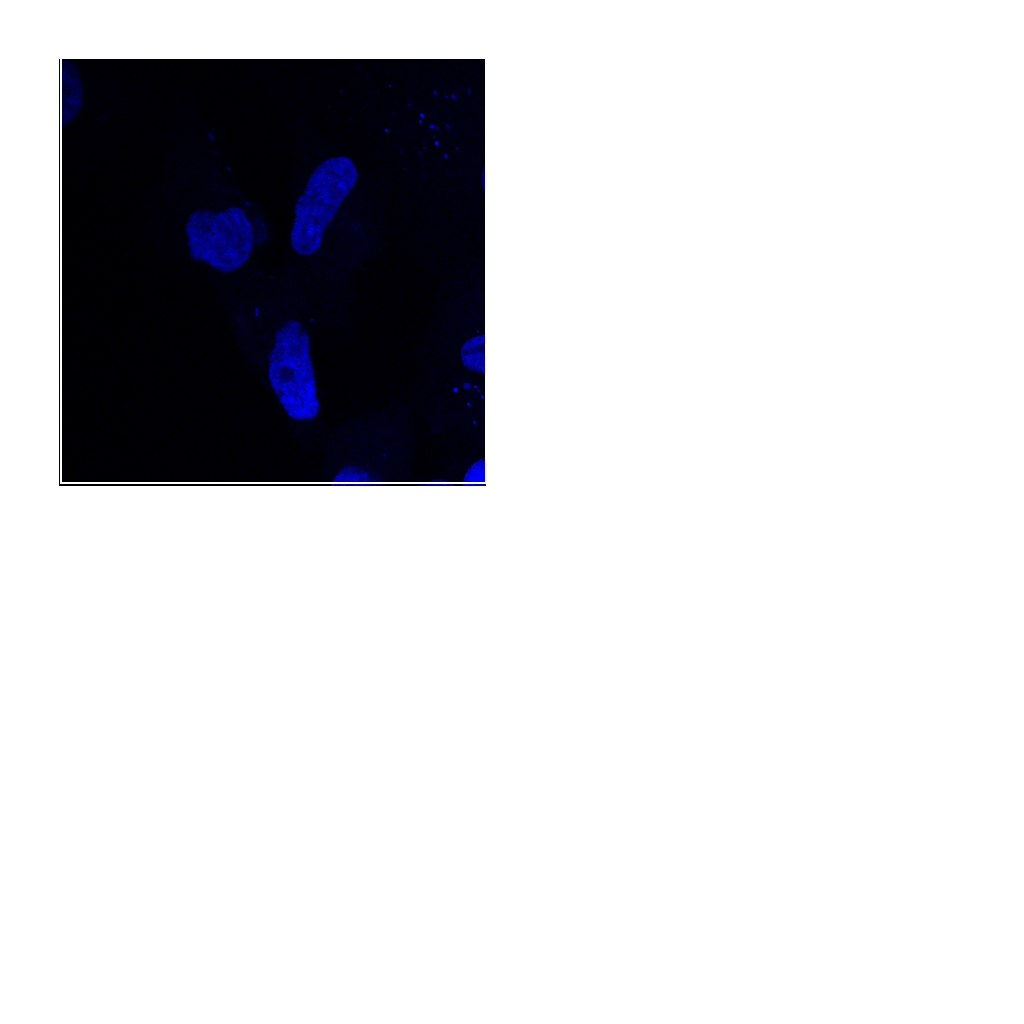

Supplement: Supplementary file 4 — Source data Fig. 2 [file 44319_2024_203_MOESM4_ESM.zip › 2B/3. VENUS-HRAS+mCherry-RASSF5/Hoechst.tif]

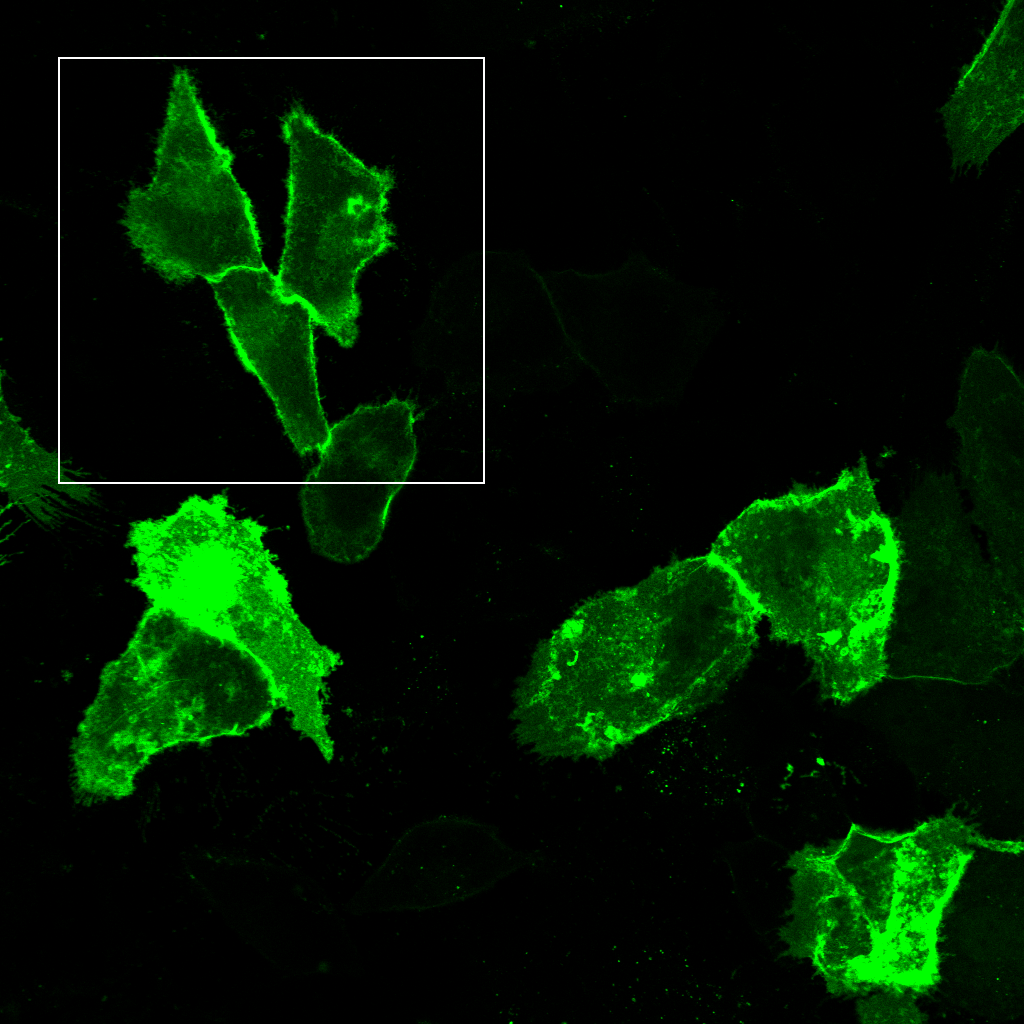

Supplement: Supplementary file 4 — Source data Fig. 2 [file 44319_2024_203_MOESM4_ESM.zip › 2B/3. VENUS-HRAS+mCherry-RASSF5/HRAS.tif]

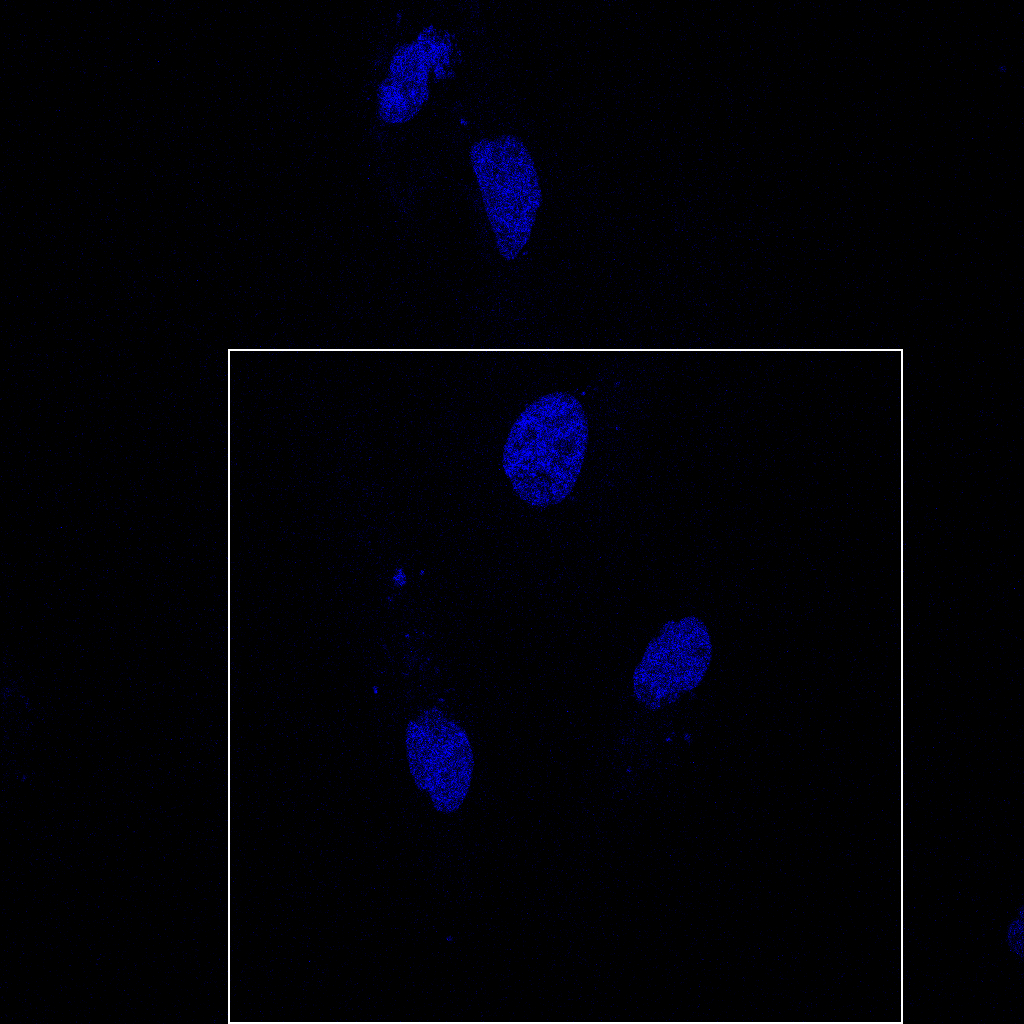

Supplement: Supplementary file 4 — Source data Fig. 2 [file 44319_2024_203_MOESM4_ESM.zip › 2B/4. VENUS-NRAS+mCherry-RASSF5/Hoechst.tif]

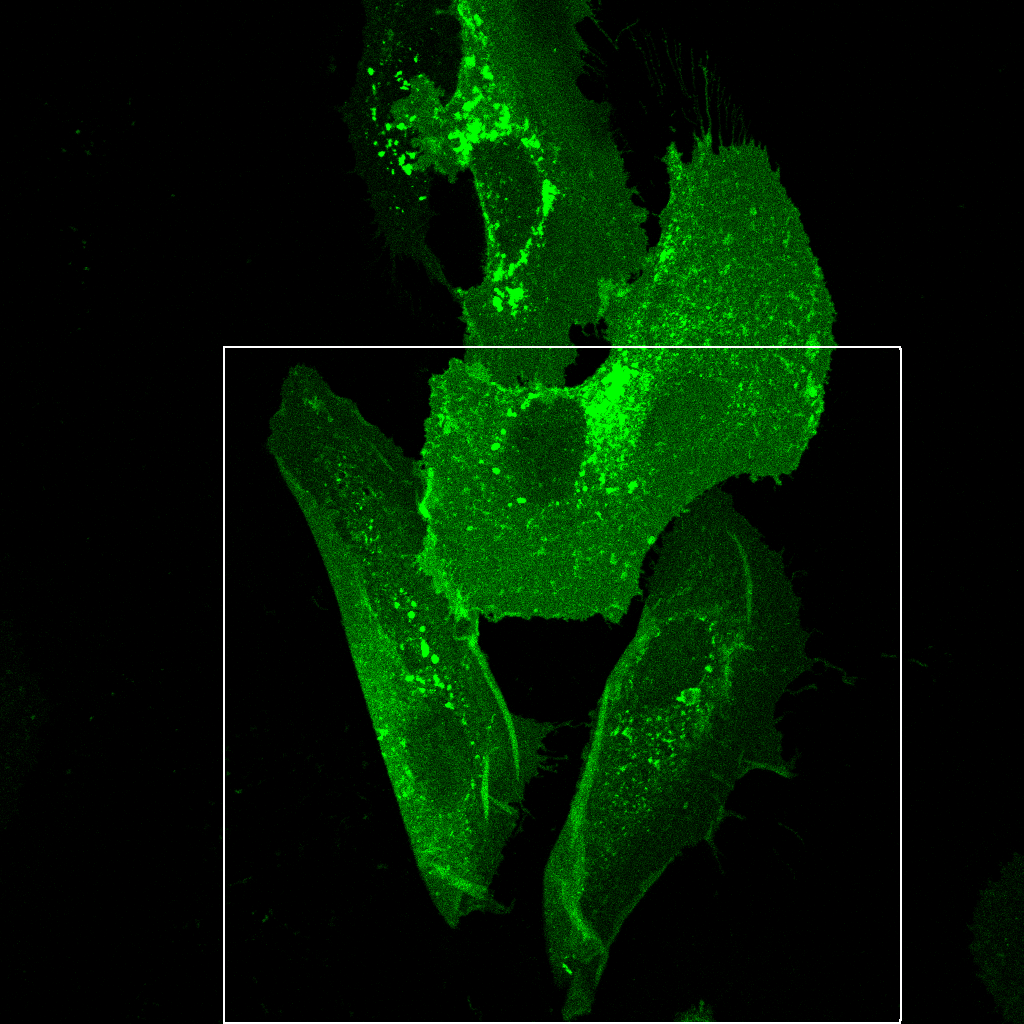

Supplement: Supplementary file 4 — Source data Fig. 2 [file 44319_2024_203_MOESM4_ESM.zip › 2B/4. VENUS-NRAS+mCherry-RASSF5/NRAS.tif]

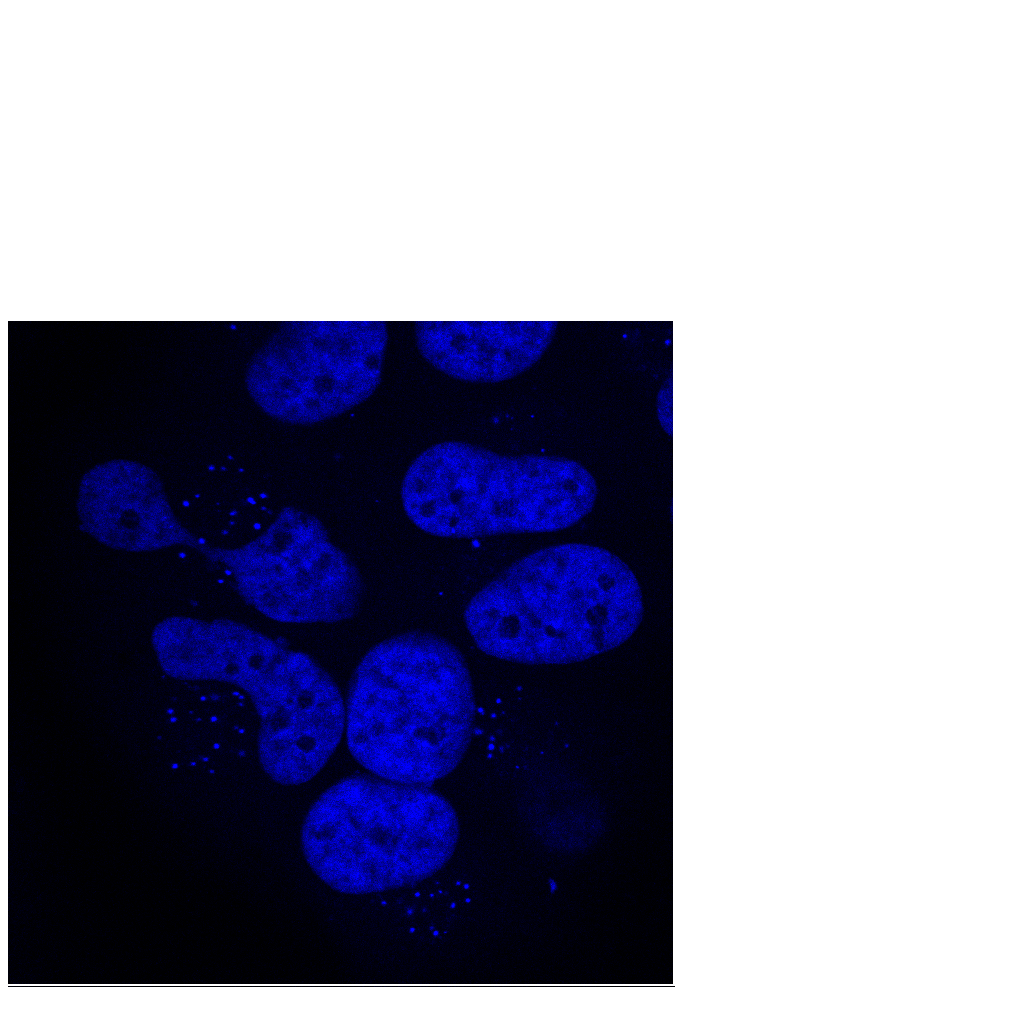

Supplement: Supplementary file 4 — Source data Fig. 2 [file 44319_2024_203_MOESM4_ESM.zip › 2C/1.VENUS-RRAS+mCherry-RASSF5/Hoechst.tif]

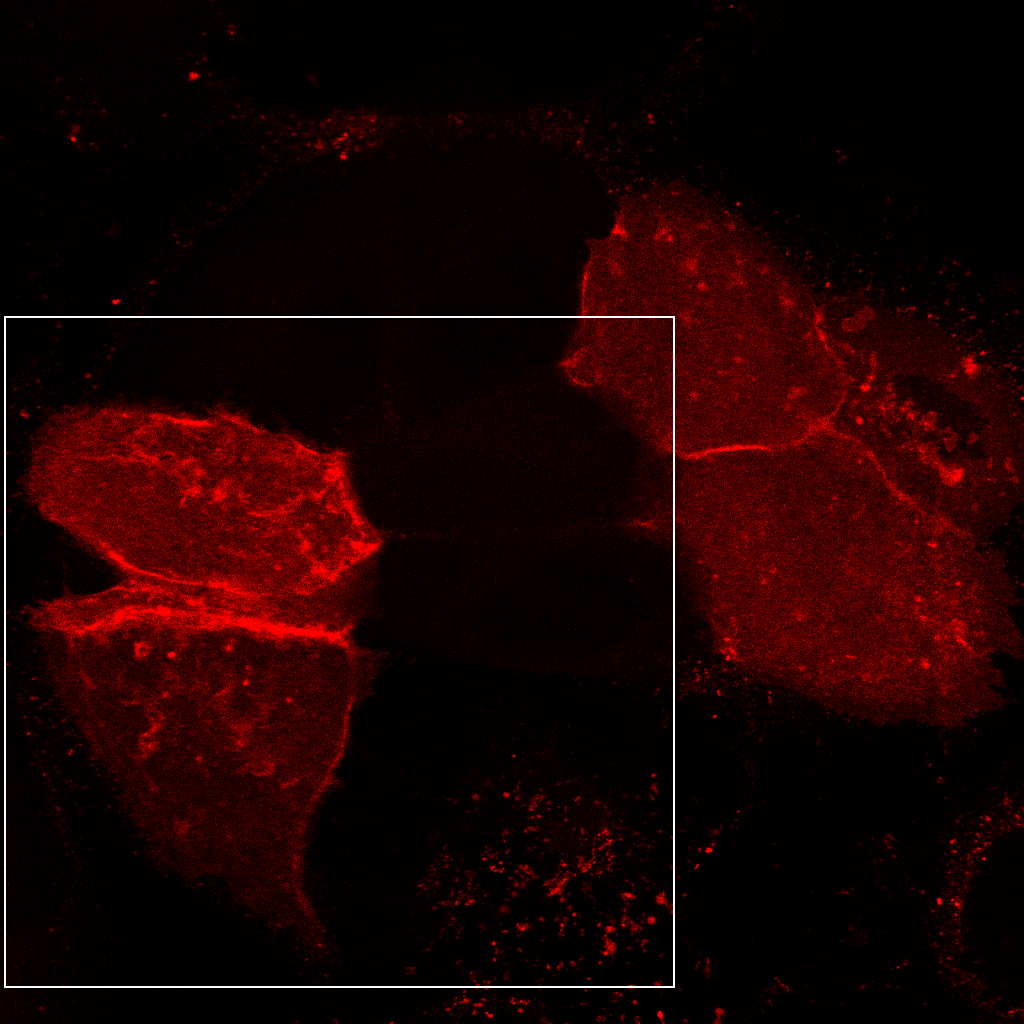

Supplement: Supplementary file 4 — Source data Fig. 2 [file 44319_2024_203_MOESM4_ESM.zip › 2C/1.VENUS-RRAS+mCherry-RASSF5/RASSF5.tif]

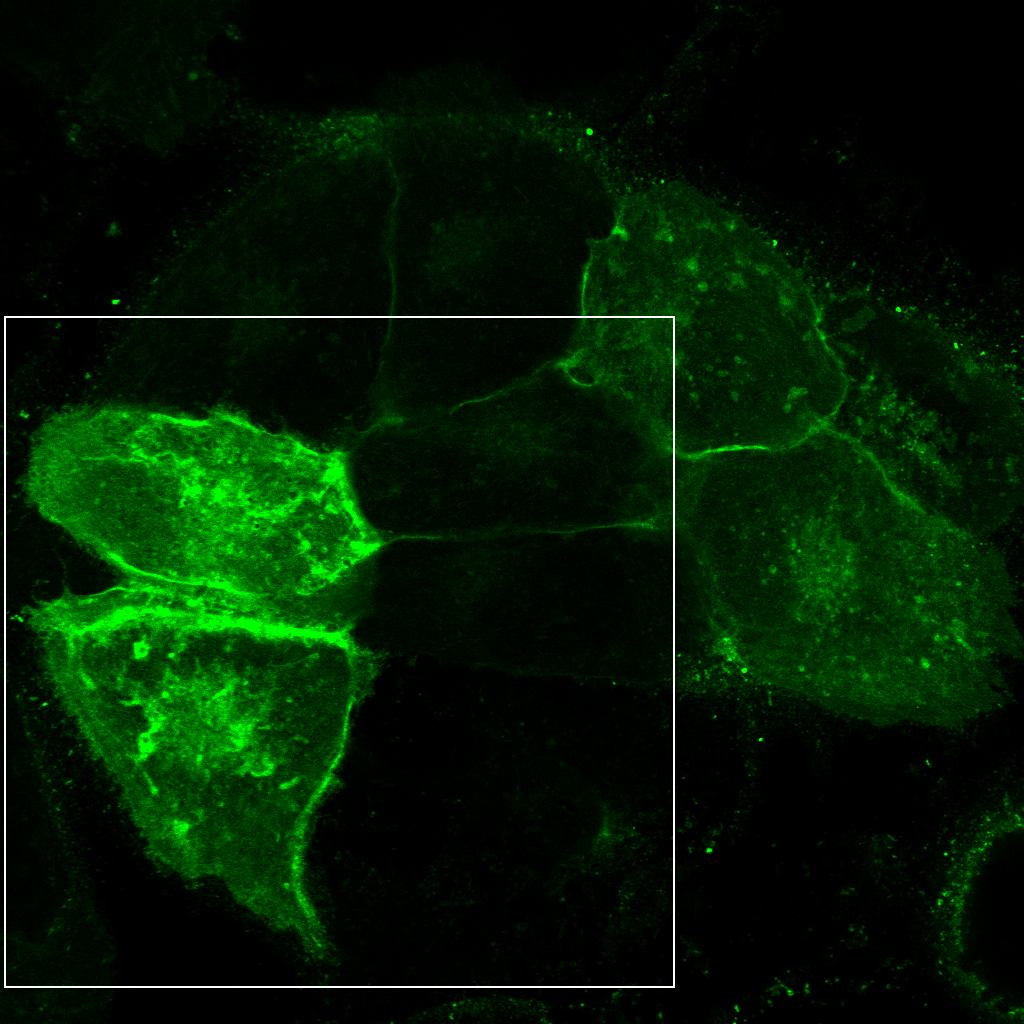

Supplement: Supplementary file 4 — Source data Fig. 2 [file 44319_2024_203_MOESM4_ESM.zip › 2C/1.VENUS-RRAS+mCherry-RASSF5/RRAS1.tif]

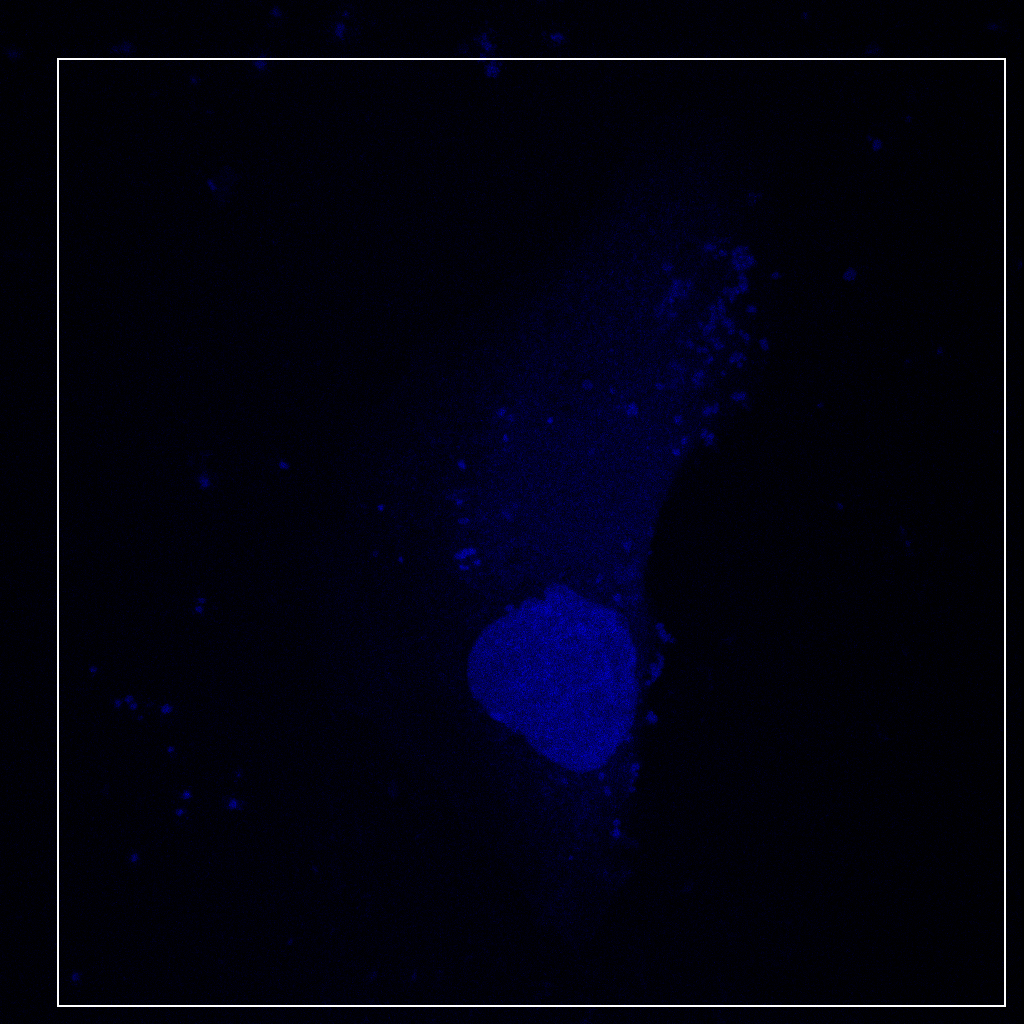

Supplement: Supplementary file 4 — Source data Fig. 2 [file 44319_2024_203_MOESM4_ESM.zip › 2C/2.VENUS-RRAS2+mCherry-RASSF5/Hoechst.tif]

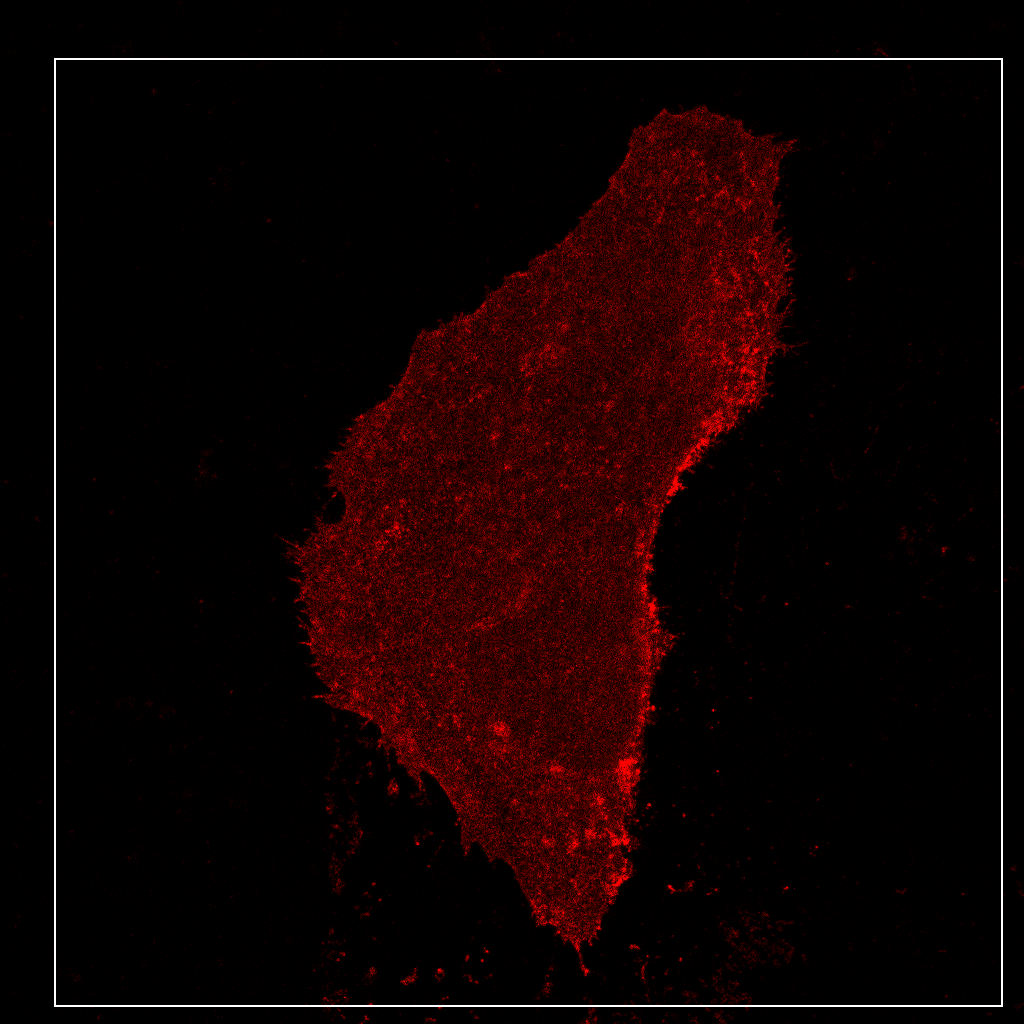

Supplement: Supplementary file 4 — Source data Fig. 2 [file 44319_2024_203_MOESM4_ESM.zip › 2C/2.VENUS-RRAS2+mCherry-RASSF5/RASSF5.tif]

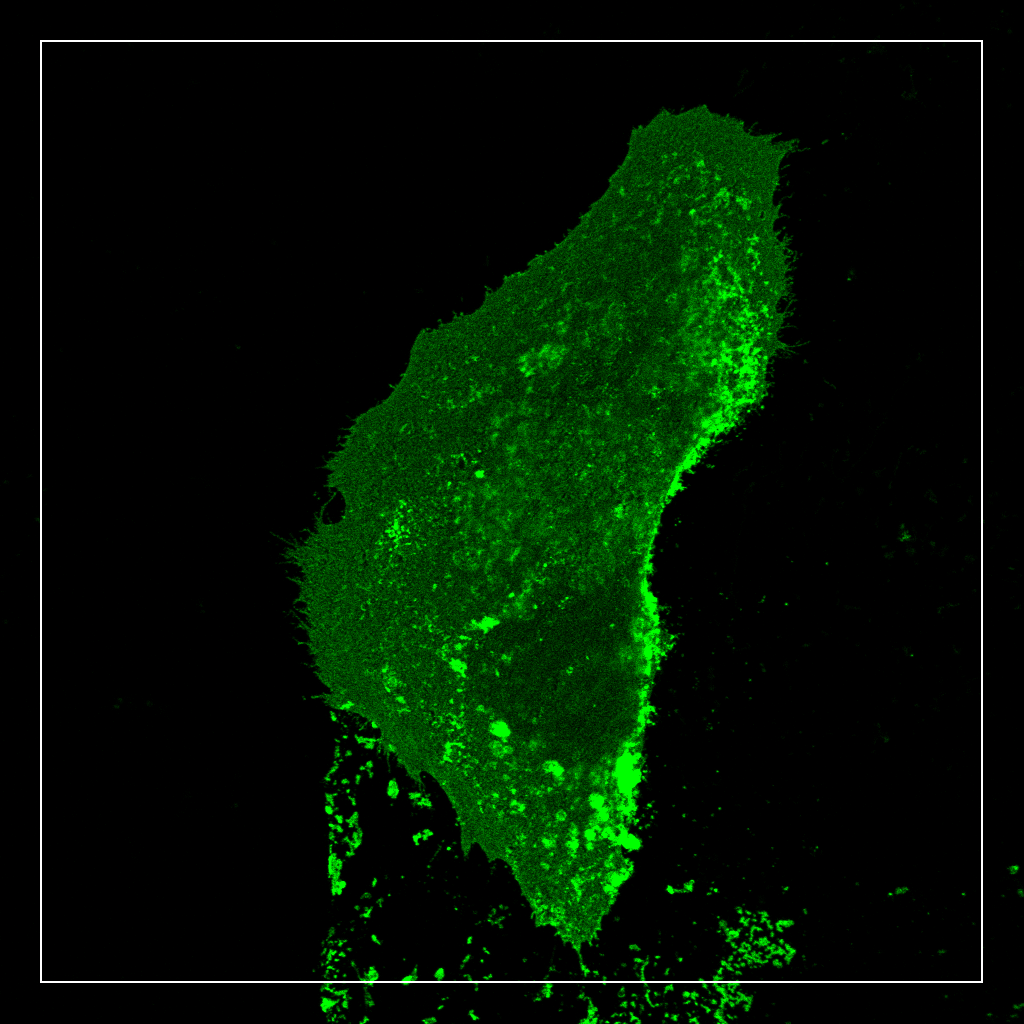

Supplement: Supplementary file 4 — Source data Fig. 2 [file 44319_2024_203_MOESM4_ESM.zip › 2C/2.VENUS-RRAS2+mCherry-RASSF5/RRAS2.tif]

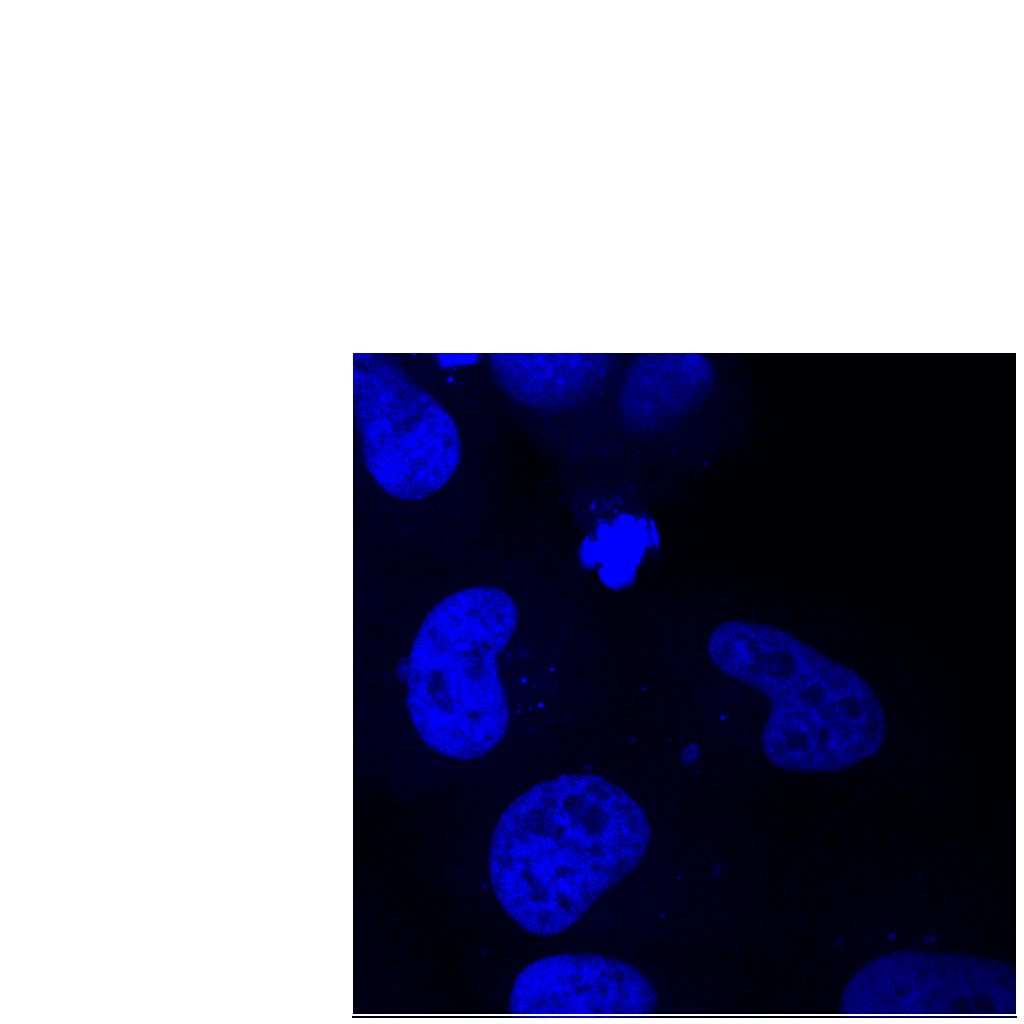

Supplement: Supplementary file 4 — Source data Fig. 2 [file 44319_2024_203_MOESM4_ESM.zip › 2C/3.VENUS-MRAS+mCherry-RASSF5/Hoechst.tif]

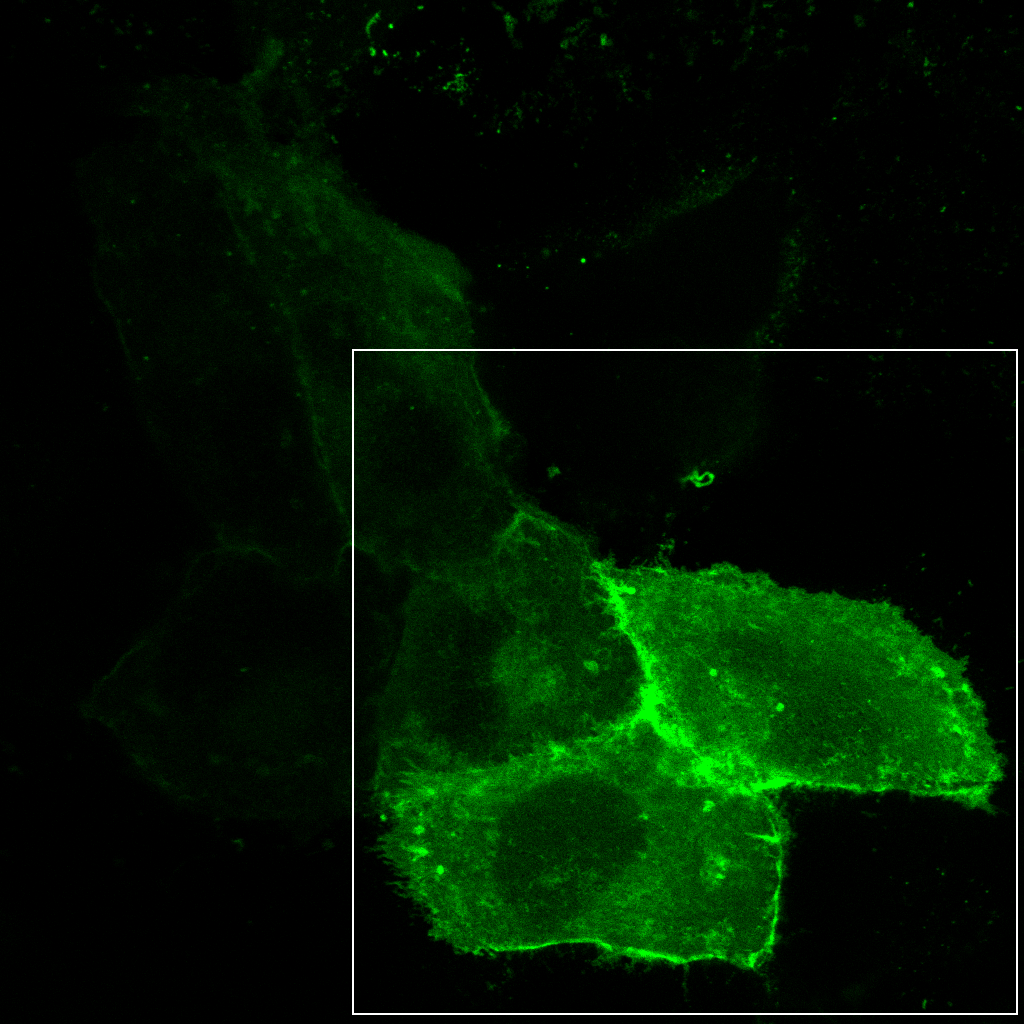

Supplement: Supplementary file 4 — Source data Fig. 2 [file 44319_2024_203_MOESM4_ESM.zip › 2C/3.VENUS-MRAS+mCherry-RASSF5/MRAS.tif]

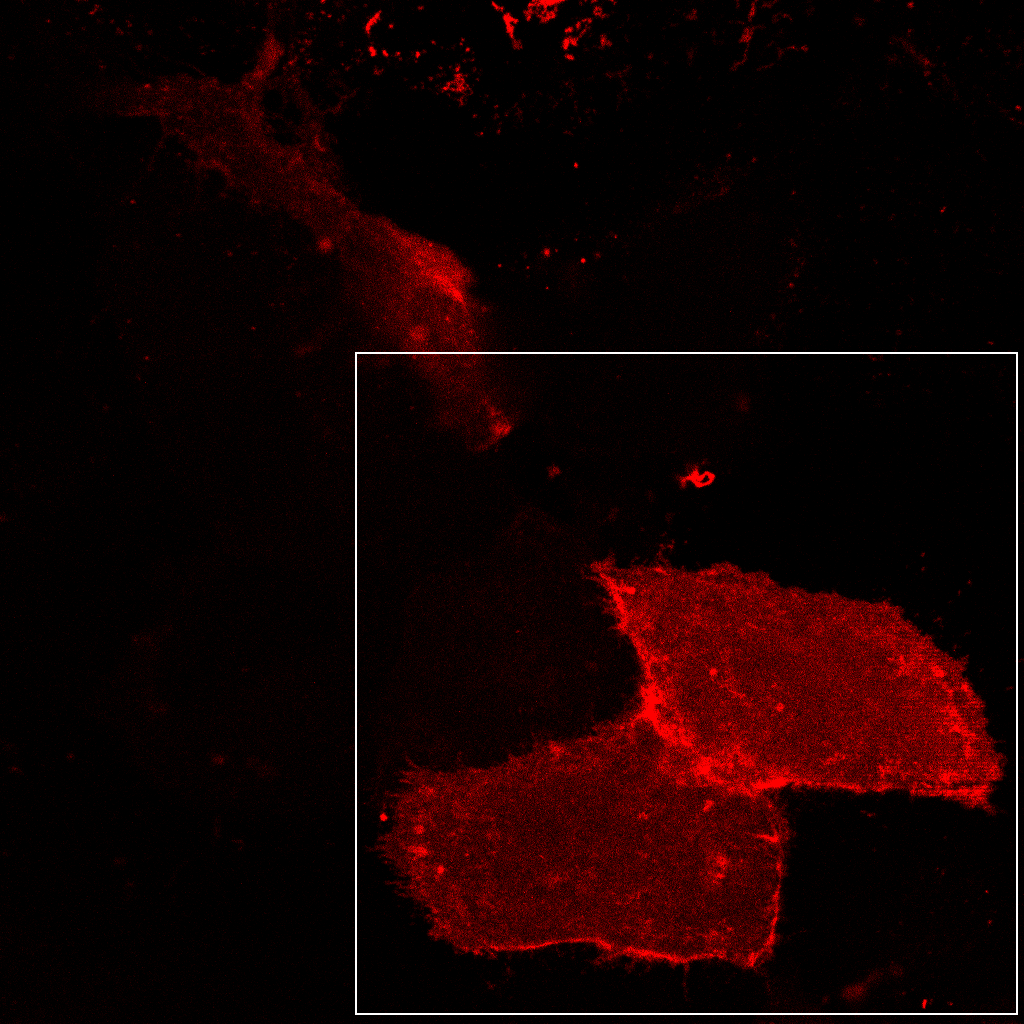

Supplement: Supplementary file 4 — Source data Fig. 2 [file 44319_2024_203_MOESM4_ESM.zip › 2C/3.VENUS-MRAS+mCherry-RASSF5/RASSF5.tif]

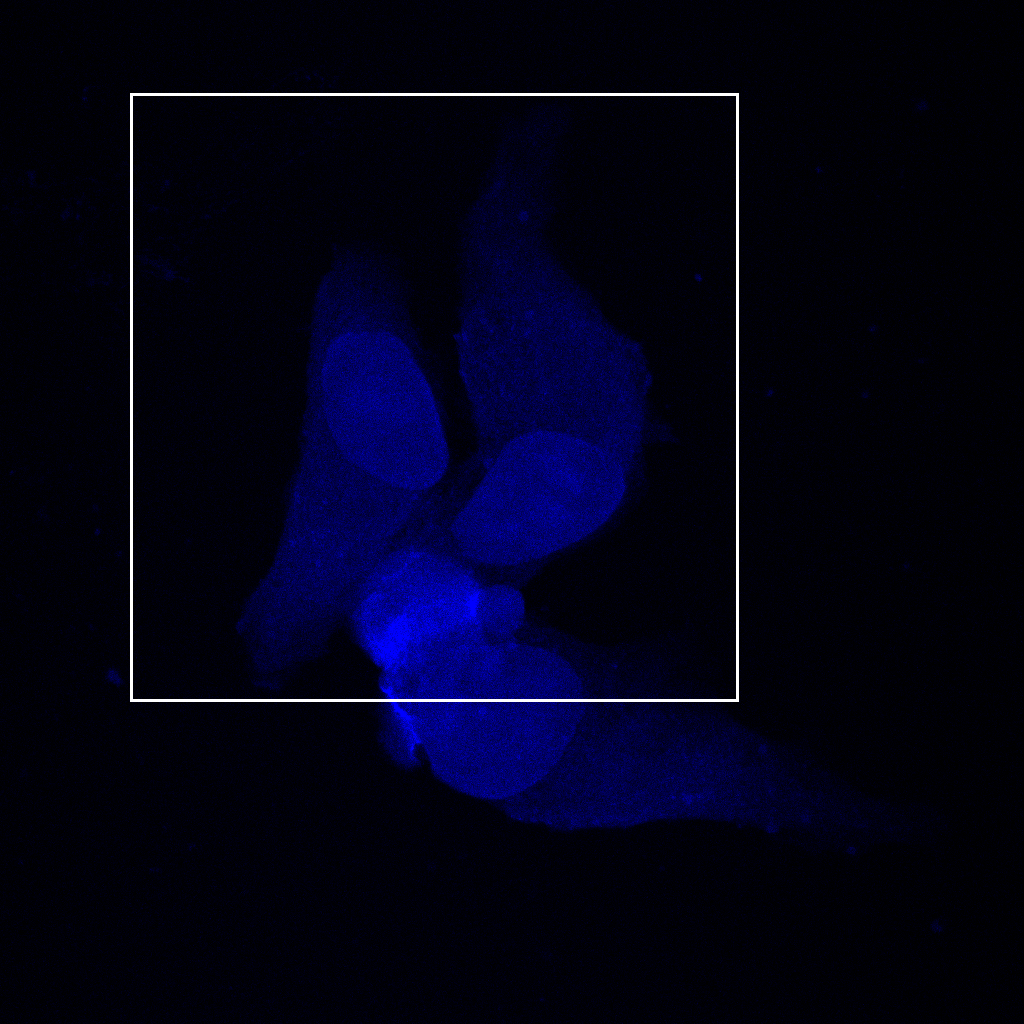

Supplement: Supplementary file 4 — Source data Fig. 2 [file 44319_2024_203_MOESM4_ESM.zip › 2C/4.VENUS-RAP2B+mCherry-RASSF5/Hoechst.tif]

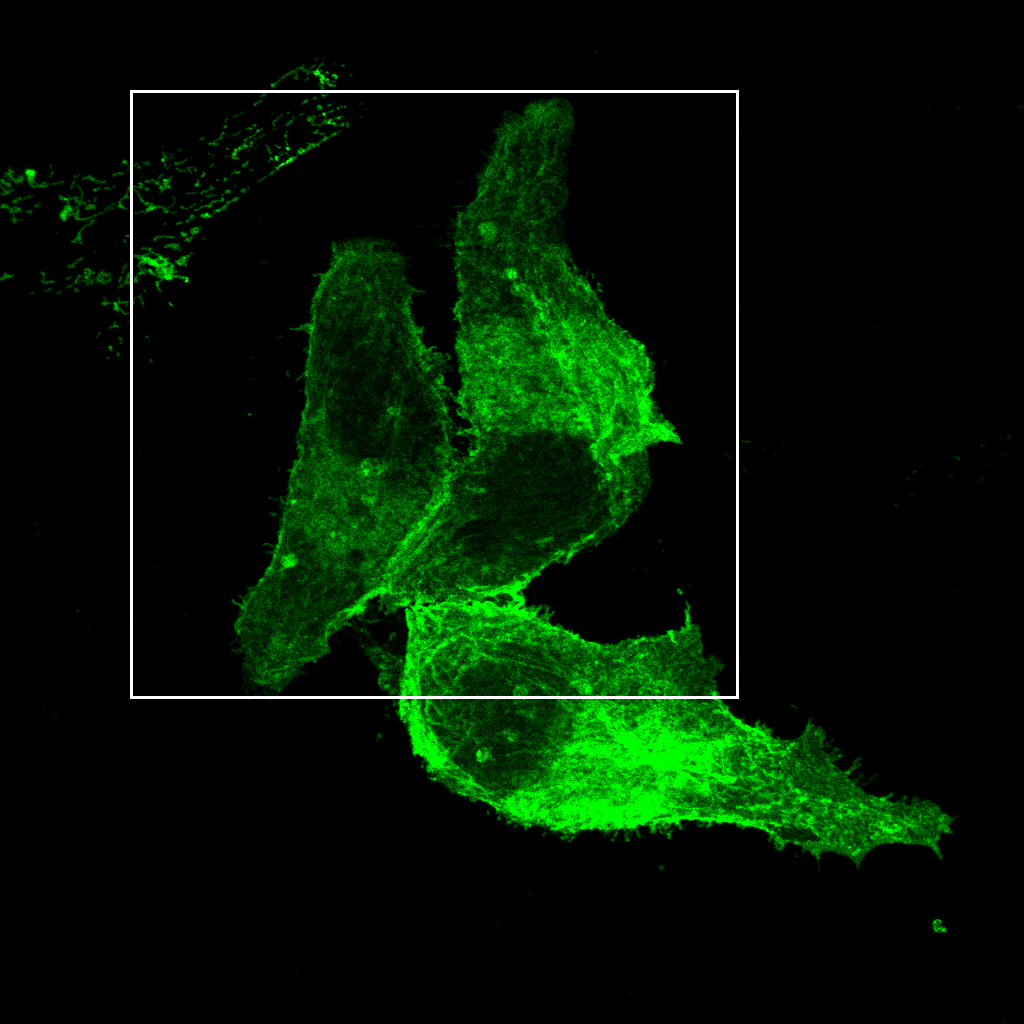

Supplement: Supplementary file 4 — Source data Fig. 2 [file 44319_2024_203_MOESM4_ESM.zip › 2C/4.VENUS-RAP2B+mCherry-RASSF5/RAP2B.tif]

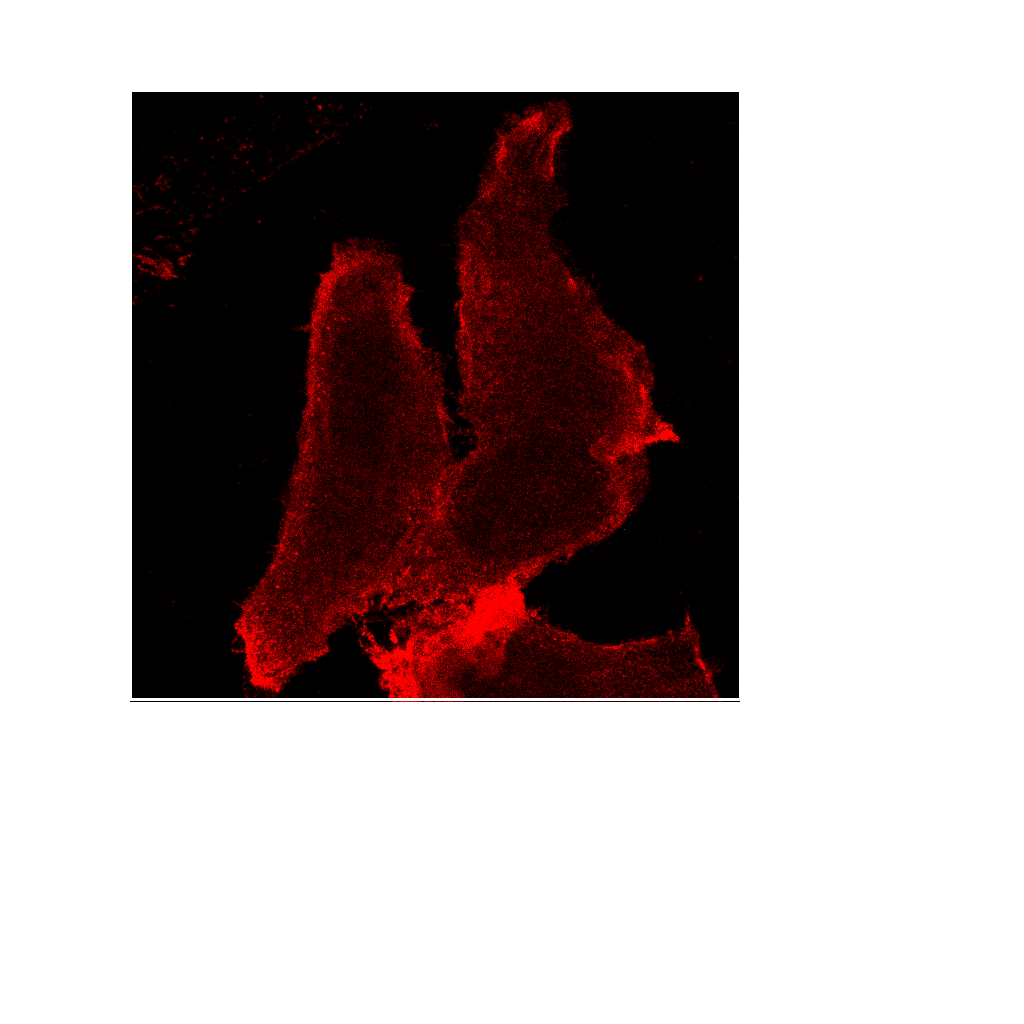

Supplement: Supplementary file 4 — Source data Fig. 2 [file 44319_2024_203_MOESM4_ESM.zip › 2C/4.VENUS-RAP2B+mCherry-RASSF5/RASSF5.tif]

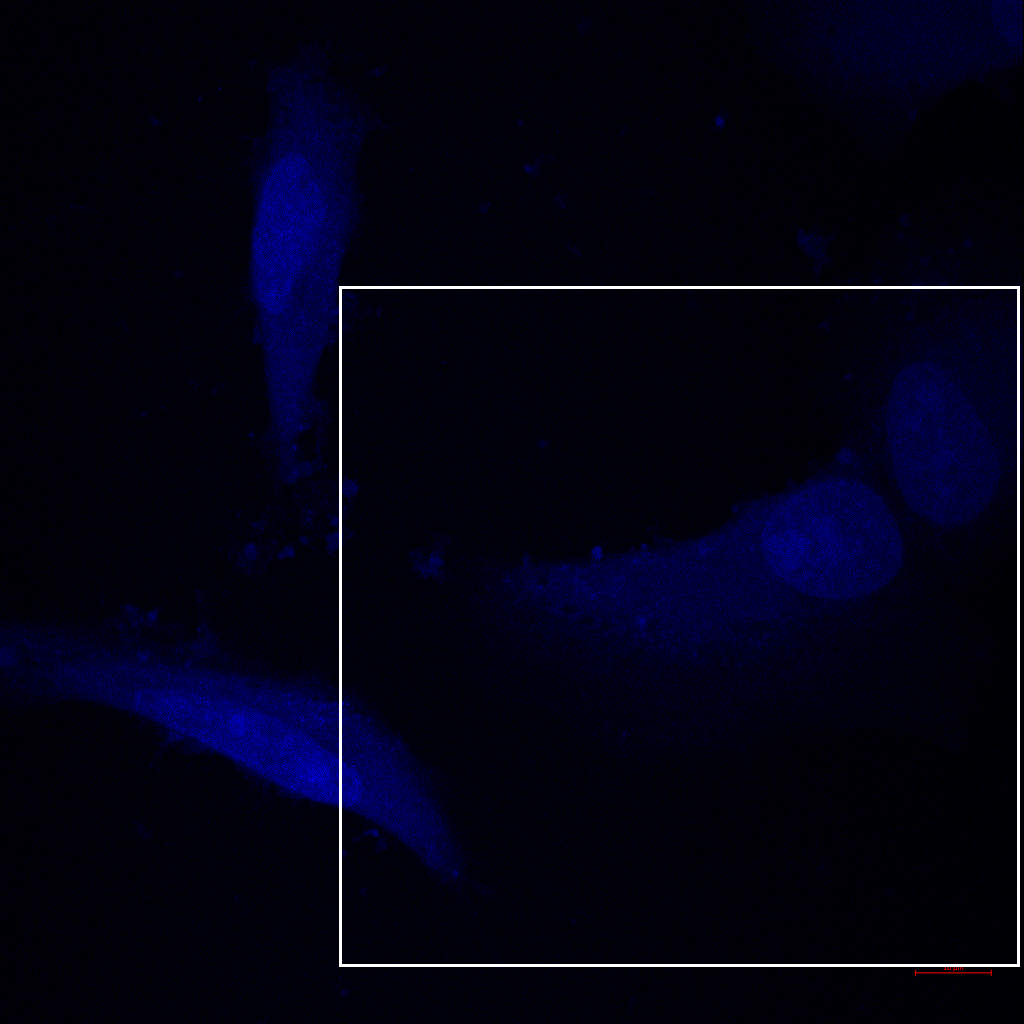

Supplement: Supplementary file 4 — Source data Fig. 2 [file 44319_2024_203_MOESM4_ESM.zip › 2C/5.VENUS-RAP2C+mCherry-RASSF5/Hoechst.tif]

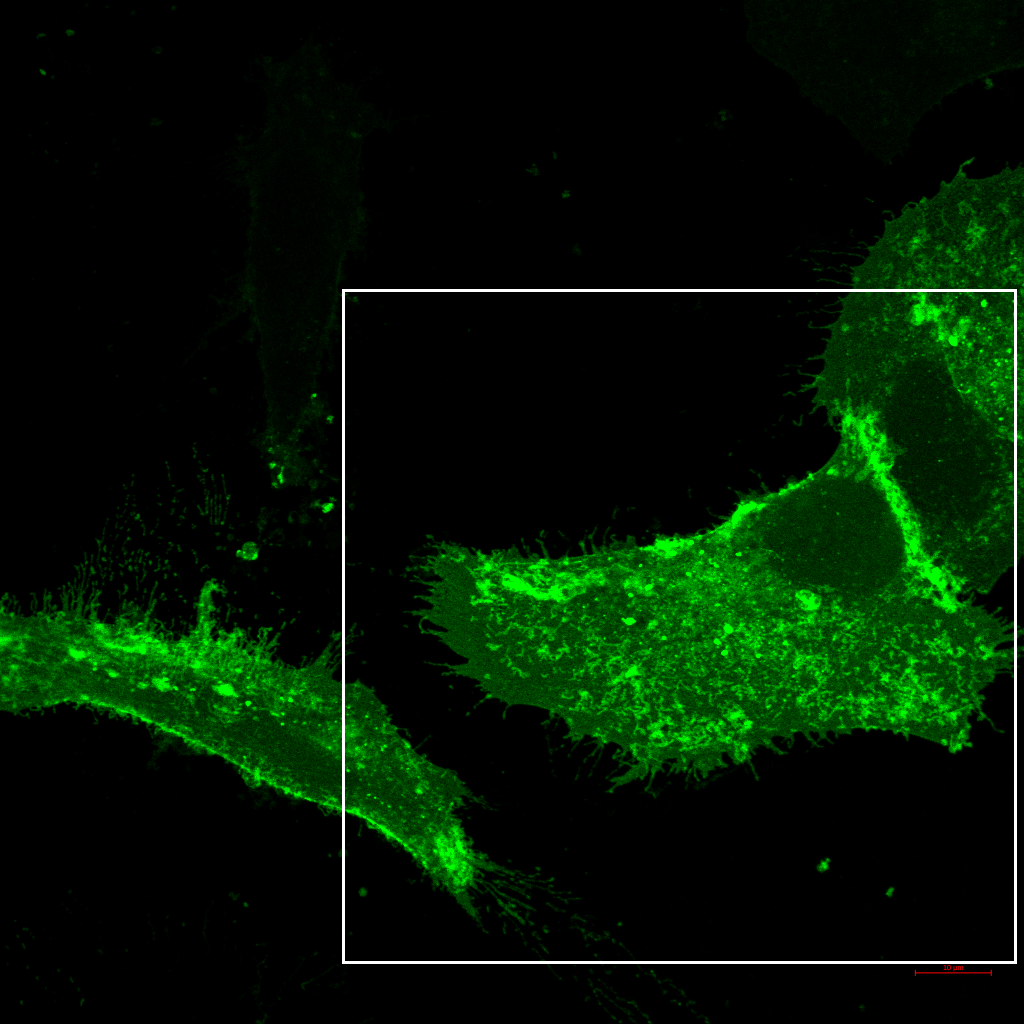

Supplement: Supplementary file 4 — Source data Fig. 2 [file 44319_2024_203_MOESM4_ESM.zip › 2C/5.VENUS-RAP2C+mCherry-RASSF5/RAP2C.tif]

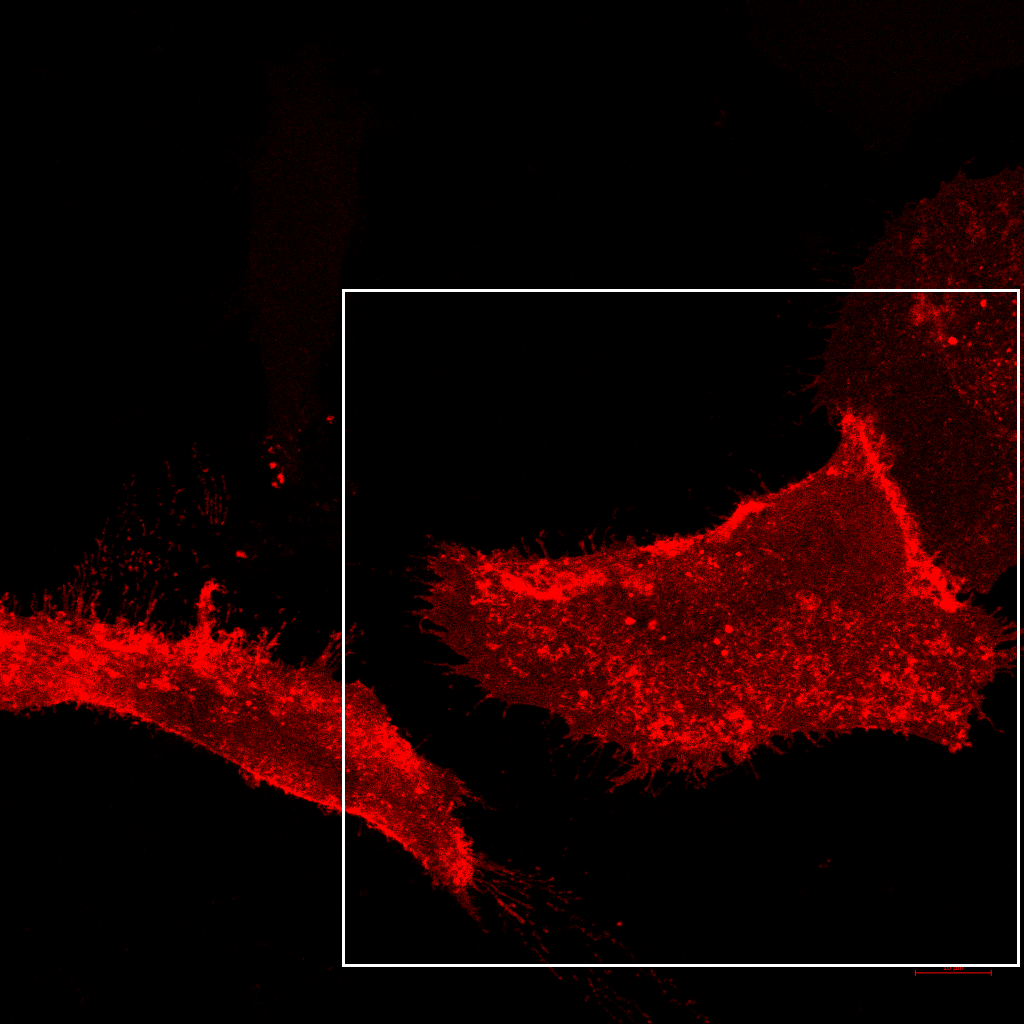

Supplement: Supplementary file 4 — Source data Fig. 2 [file 44319_2024_203_MOESM4_ESM.zip › 2C/5.VENUS-RAP2C+mCherry-RASSF5/RASSF5.tif]

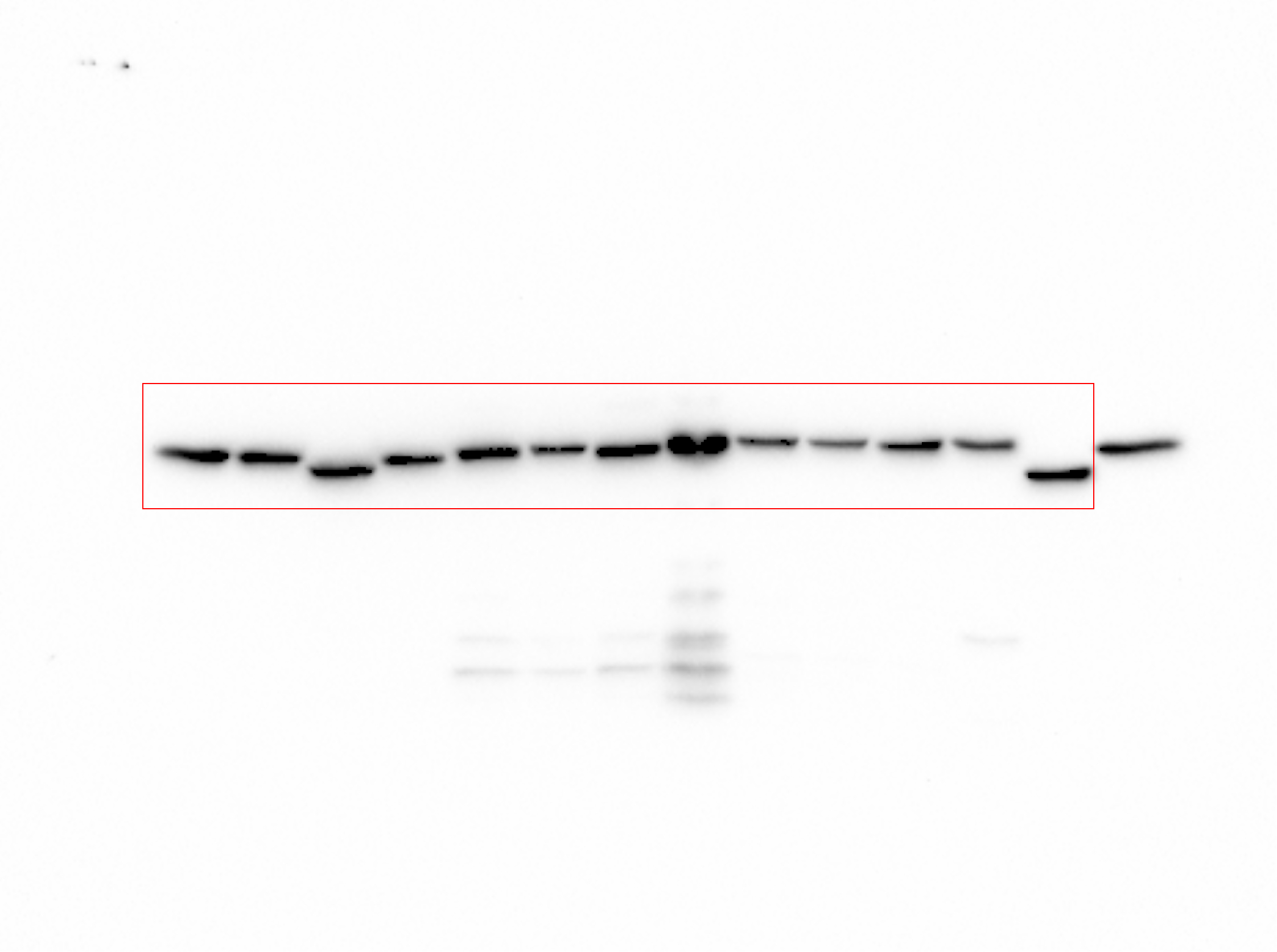

Supplement: Supplementary file 4 — Source data Fig. 2 [file 44319_2024_203_MOESM4_ESM.zip › 2A/Lysate_anti-GFP-blot.tif]

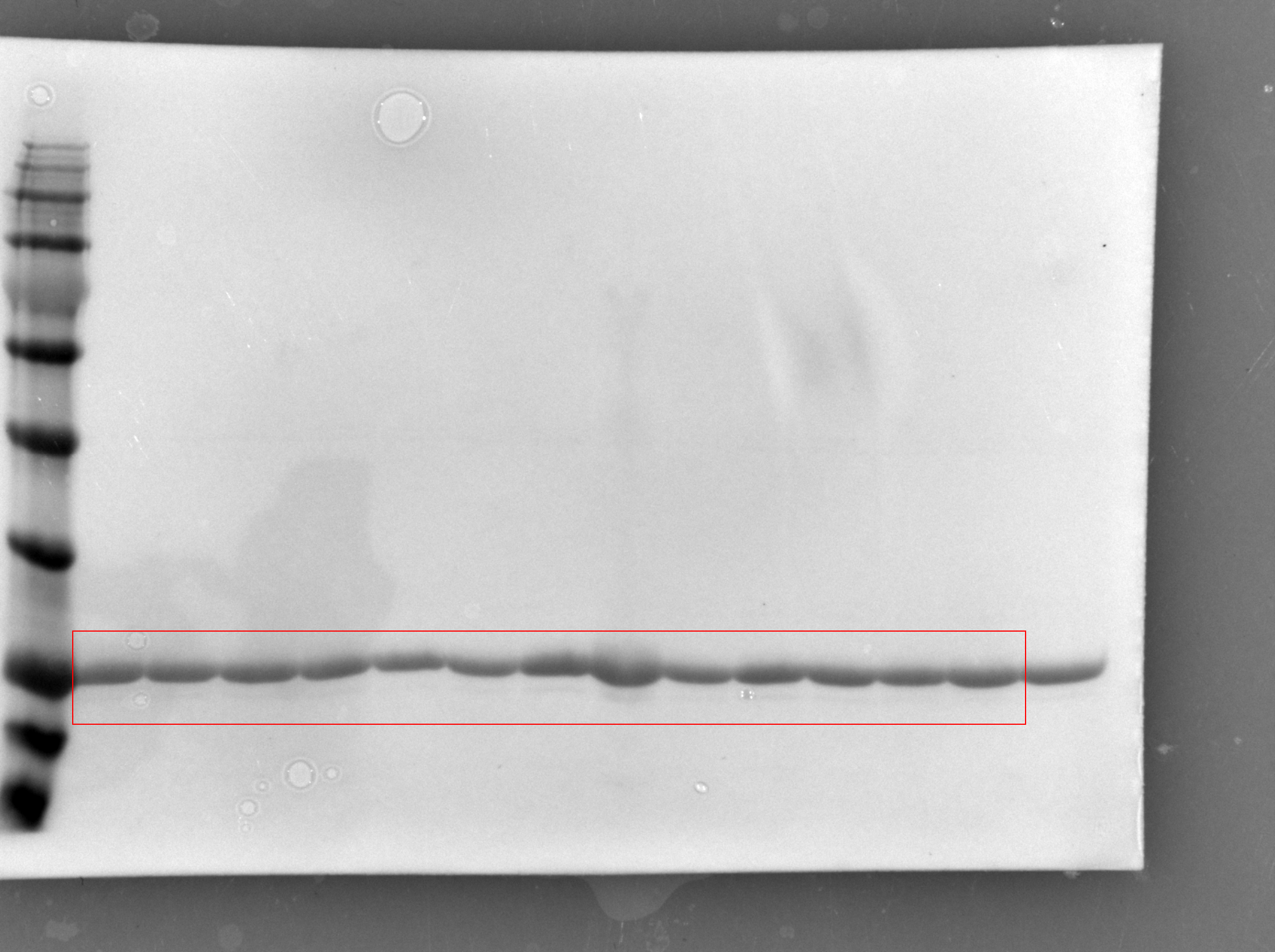

Supplement: Supplementary file 4 — Source data Fig. 2 [file 44319_2024_203_MOESM4_ESM.zip › 2A/Ponceau_GST-alone.tif]

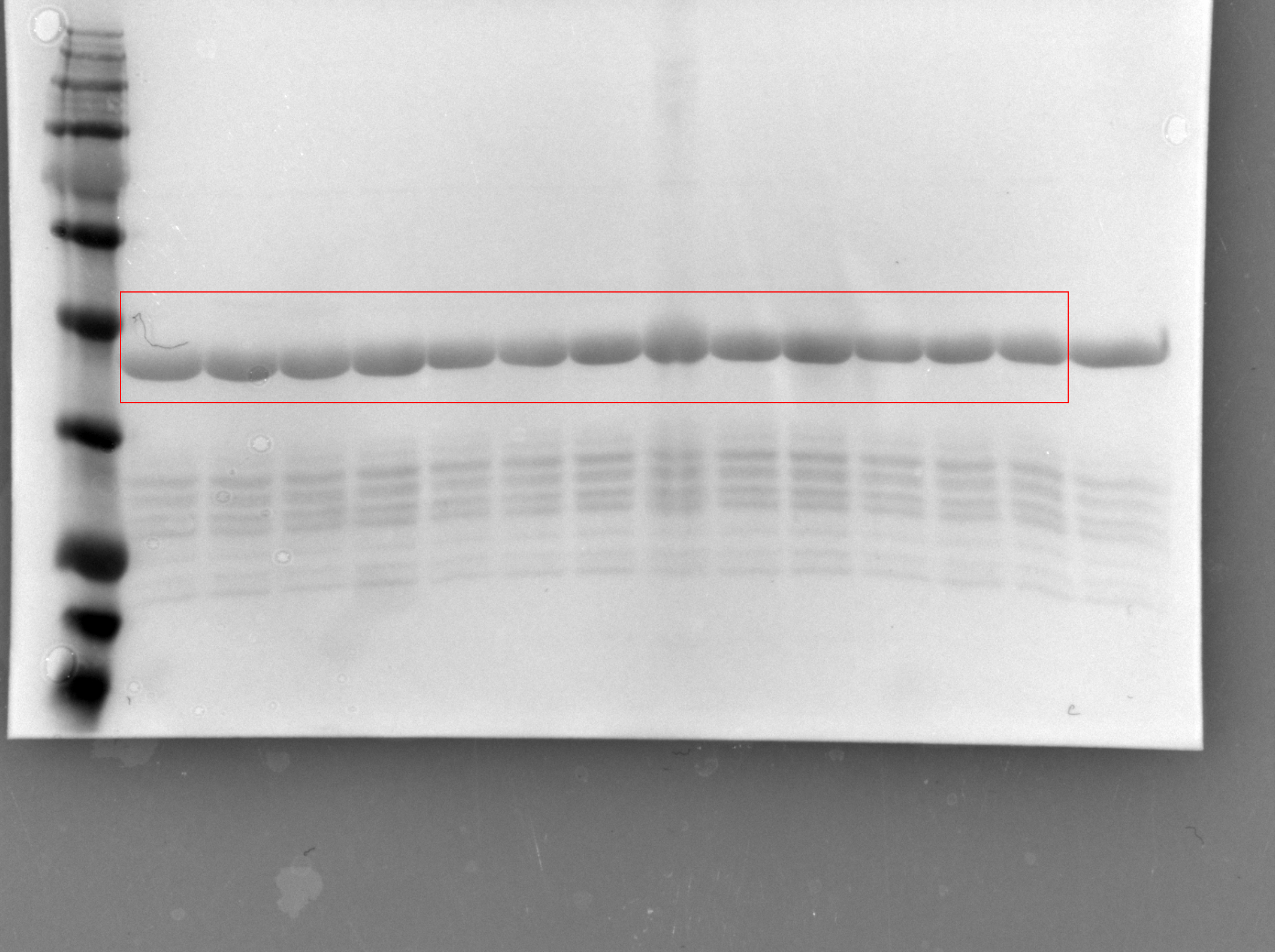

Supplement: Supplementary file 4 — Source data Fig. 2 [file 44319_2024_203_MOESM4_ESM.zip › 2A/Ponceau_GST-RASSF5-RBD.tif]

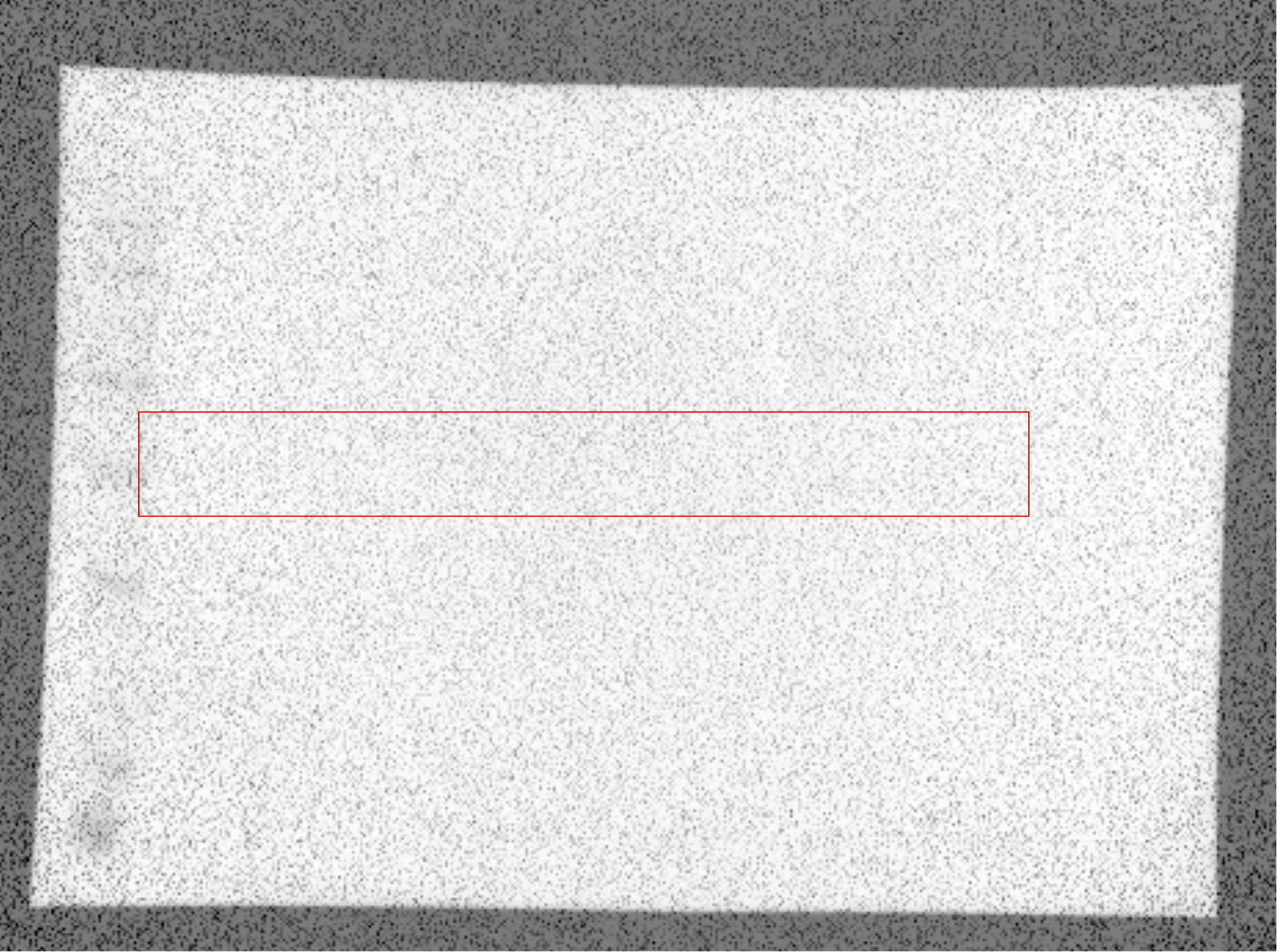

Supplement: Supplementary file 4 — Source data Fig. 2 [file 44319_2024_203_MOESM4_ESM.zip › 2A/Pull Down-GST-alone-Anti-GFP.tif]

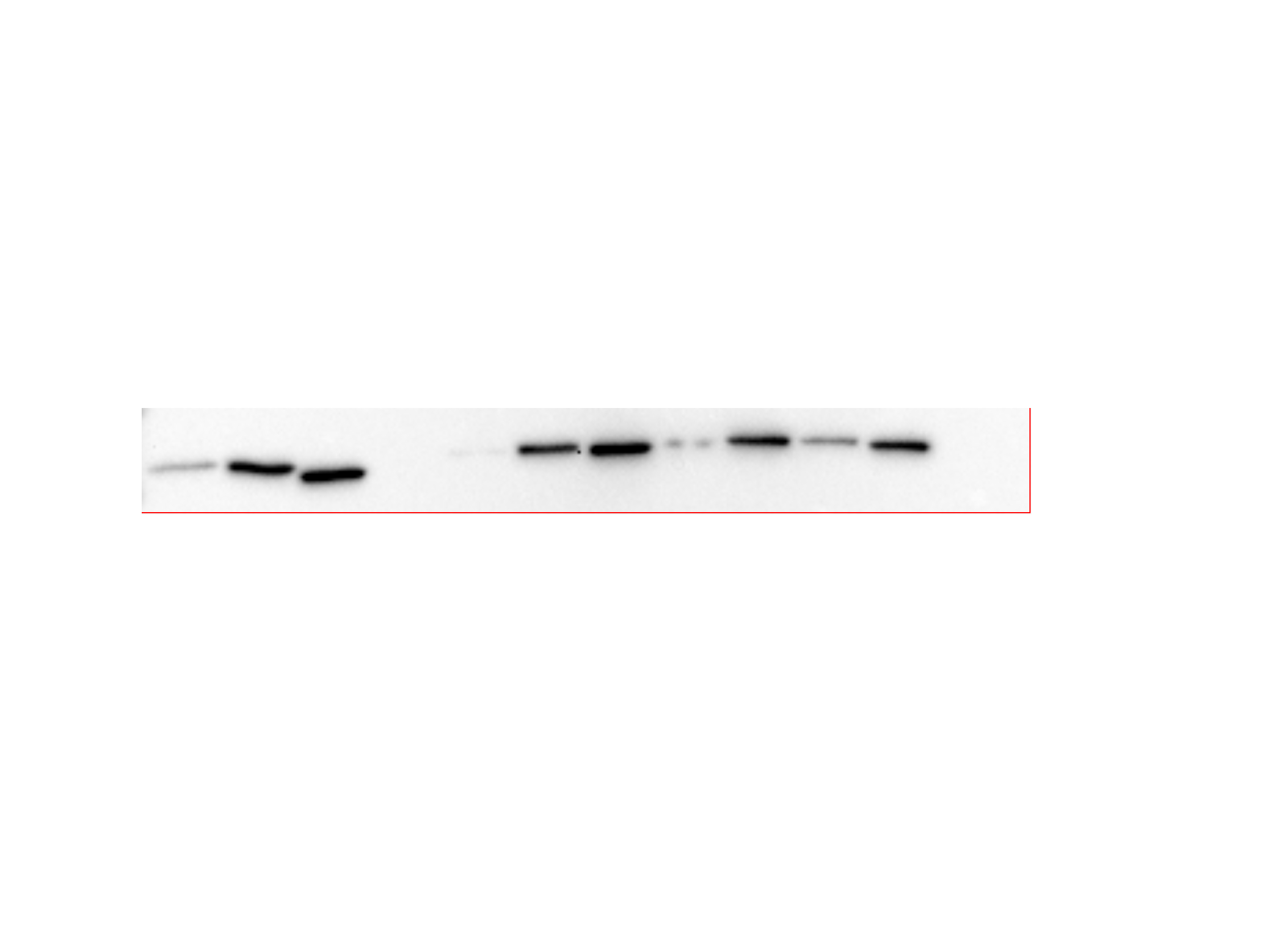

Supplement: Supplementary file 4 — Source data Fig. 2 [file 44319_2024_203_MOESM4_ESM.zip › 2A/Pull Down-GST-RASSF5-RBD-Anti-GFP.tif]

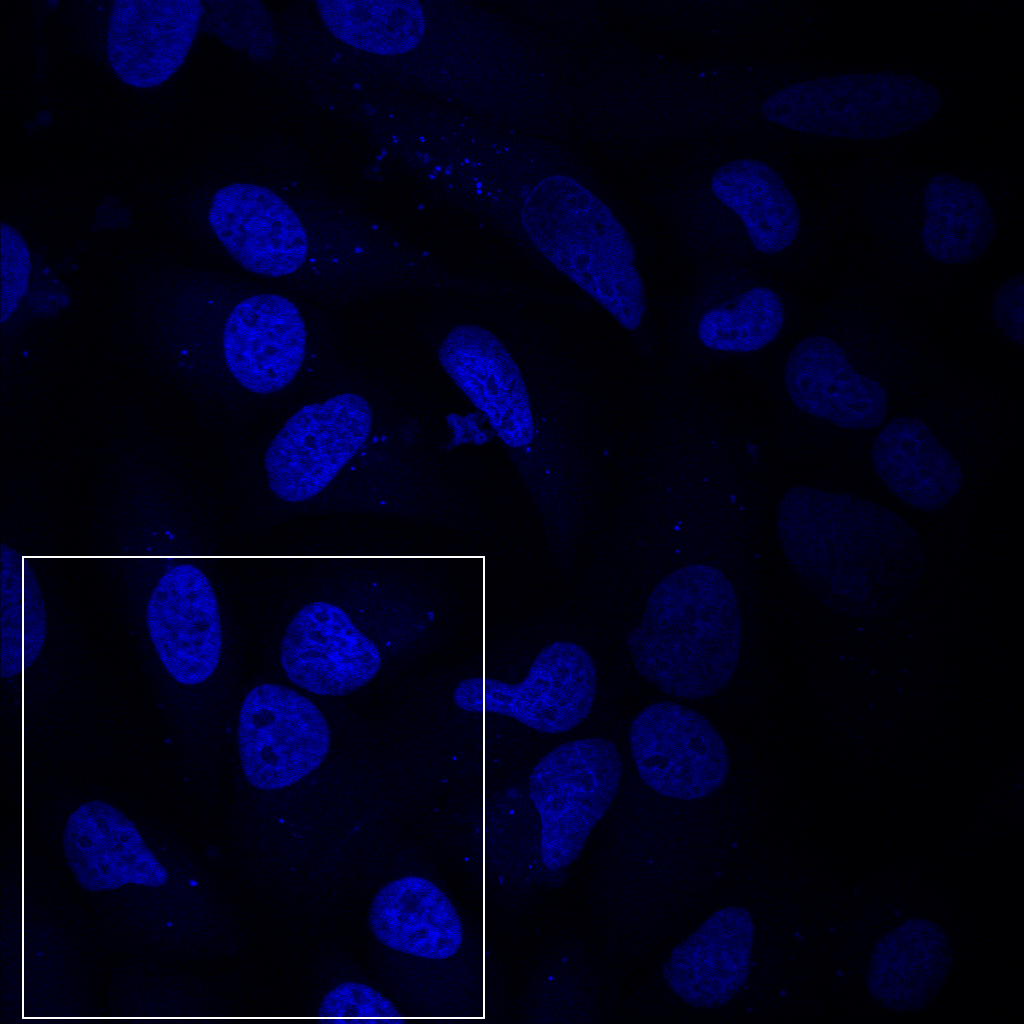

Supplement: Supplementary file 4 — Source data Fig. 2 [file 44319_2024_203_MOESM4_ESM.zip › 2D/1.VENUS-RIT1+mCherry-RASSF5/Hoechst.tif]

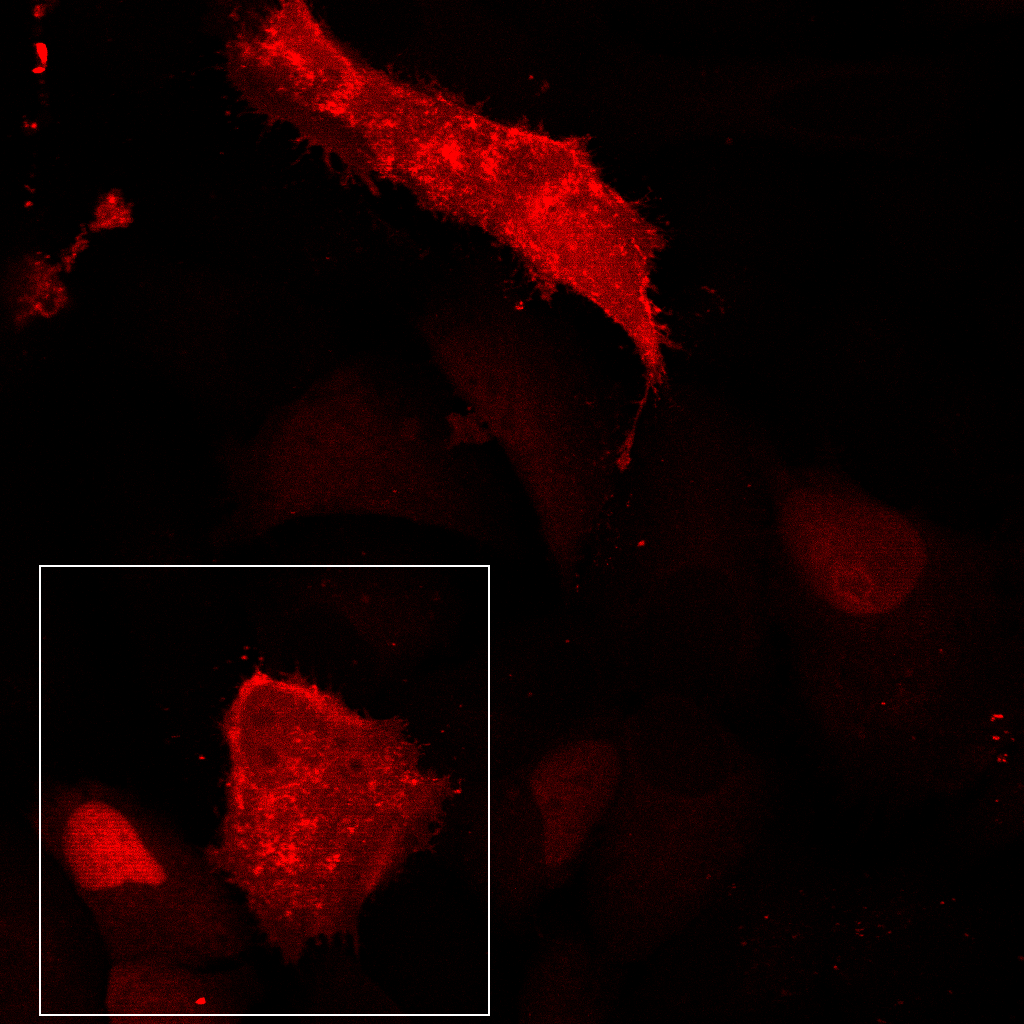

Supplement: Supplementary file 4 — Source data Fig. 2 [file 44319_2024_203_MOESM4_ESM.zip › 2D/1.VENUS-RIT1+mCherry-RASSF5/RASSF5.tif]

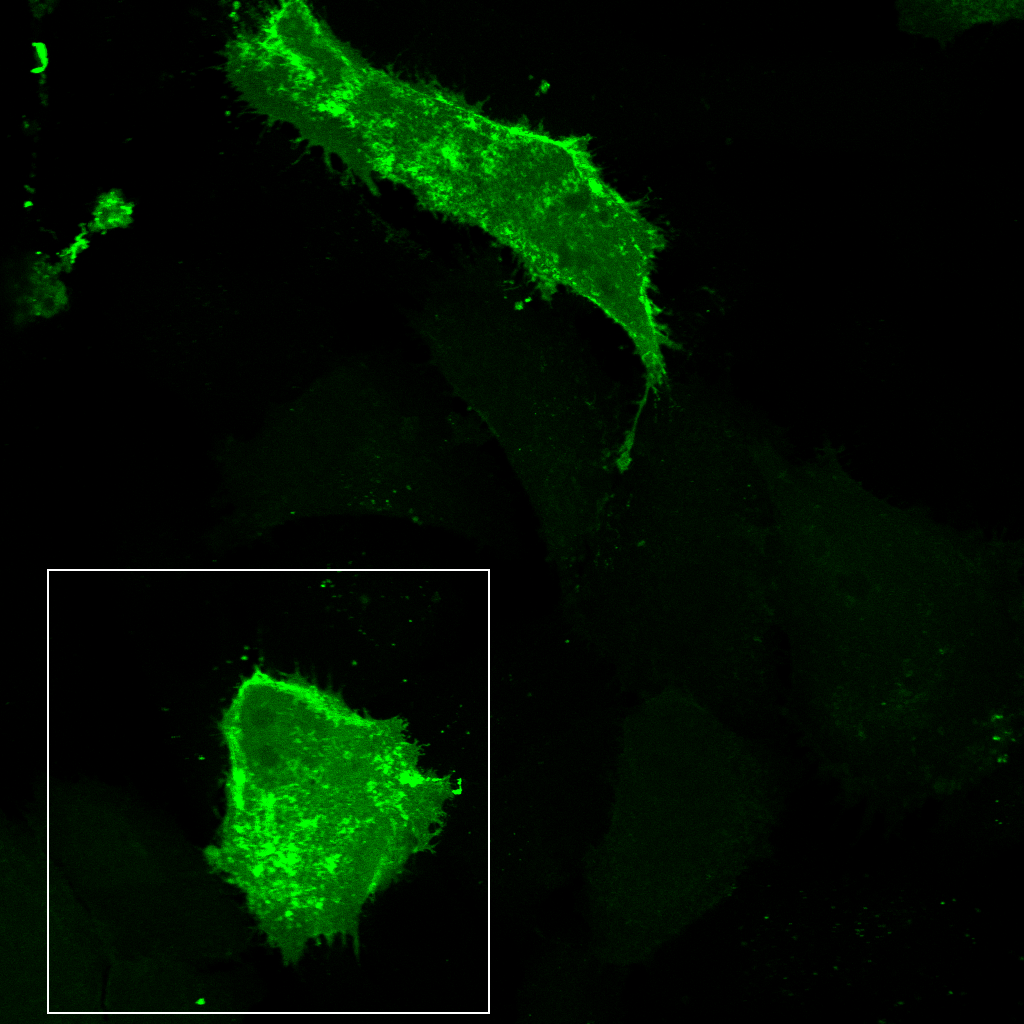

Supplement: Supplementary file 4 — Source data Fig. 2 [file 44319_2024_203_MOESM4_ESM.zip › 2D/1.VENUS-RIT1+mCherry-RASSF5/RIT1.tif]

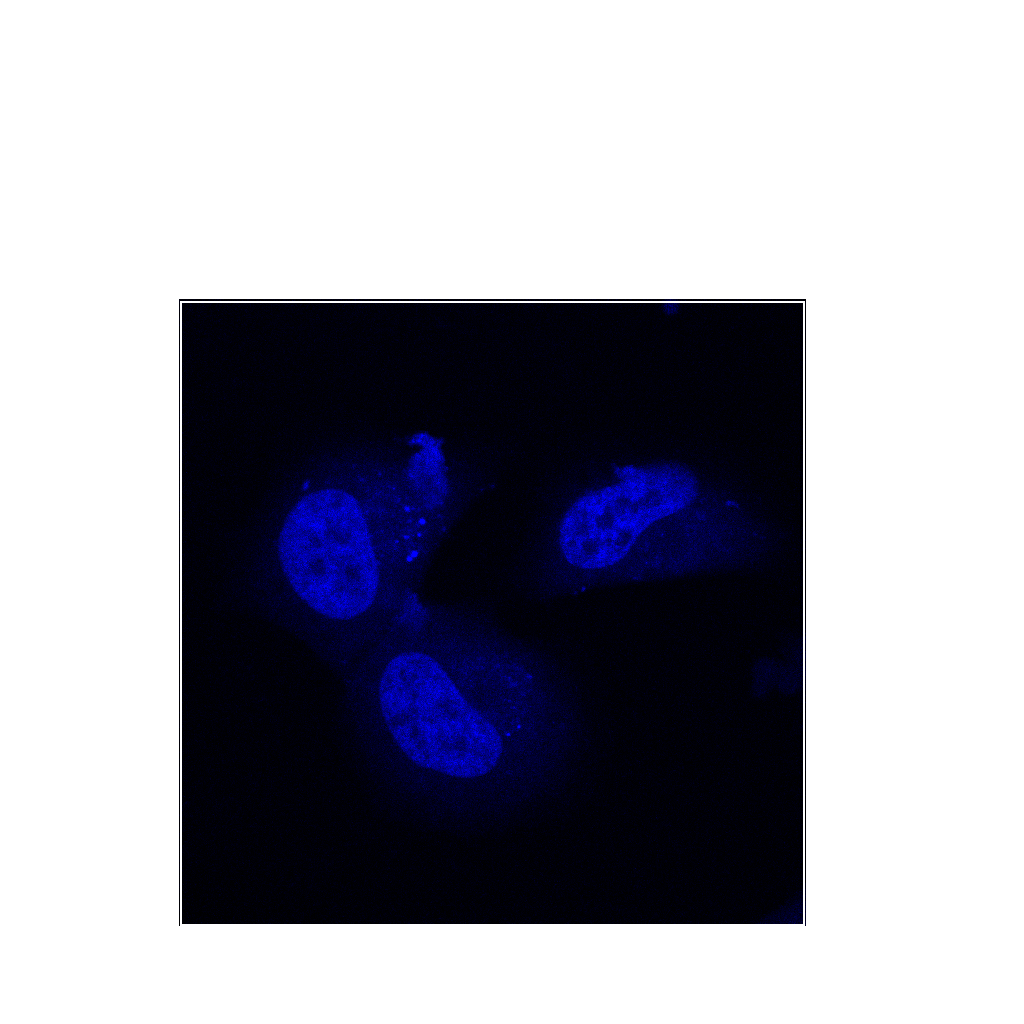

Supplement: Supplementary file 4 — Source data Fig. 2 [file 44319_2024_203_MOESM4_ESM.zip › 2D/2.VENUS-RIT2+mCherry-RASSF5/Hoechst.tif]

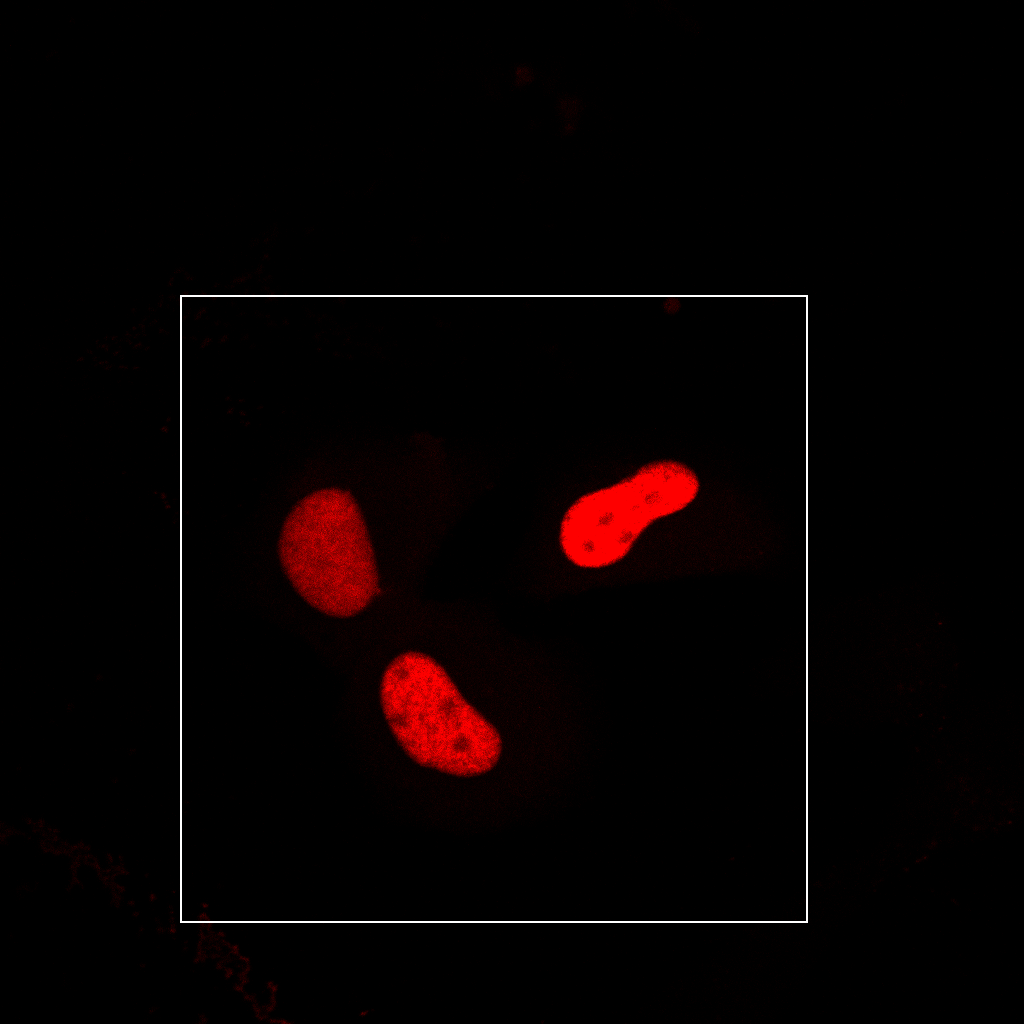

Supplement: Supplementary file 4 — Source data Fig. 2 [file 44319_2024_203_MOESM4_ESM.zip › 2D/2.VENUS-RIT2+mCherry-RASSF5/RASSF5.tif]

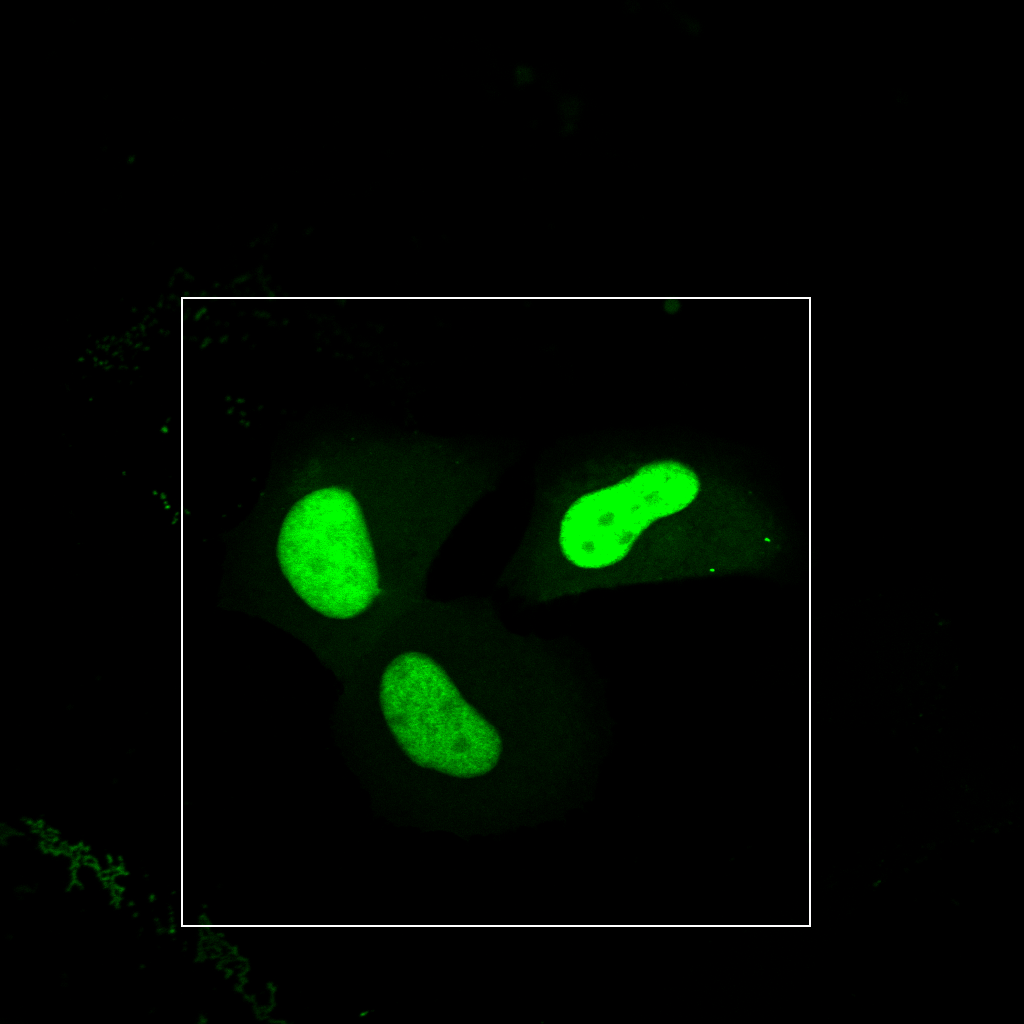

Supplement: Supplementary file 4 — Source data Fig. 2 [file 44319_2024_203_MOESM4_ESM.zip › 2D/2.VENUS-RIT2+mCherry-RASSF5/RIT2.tif]

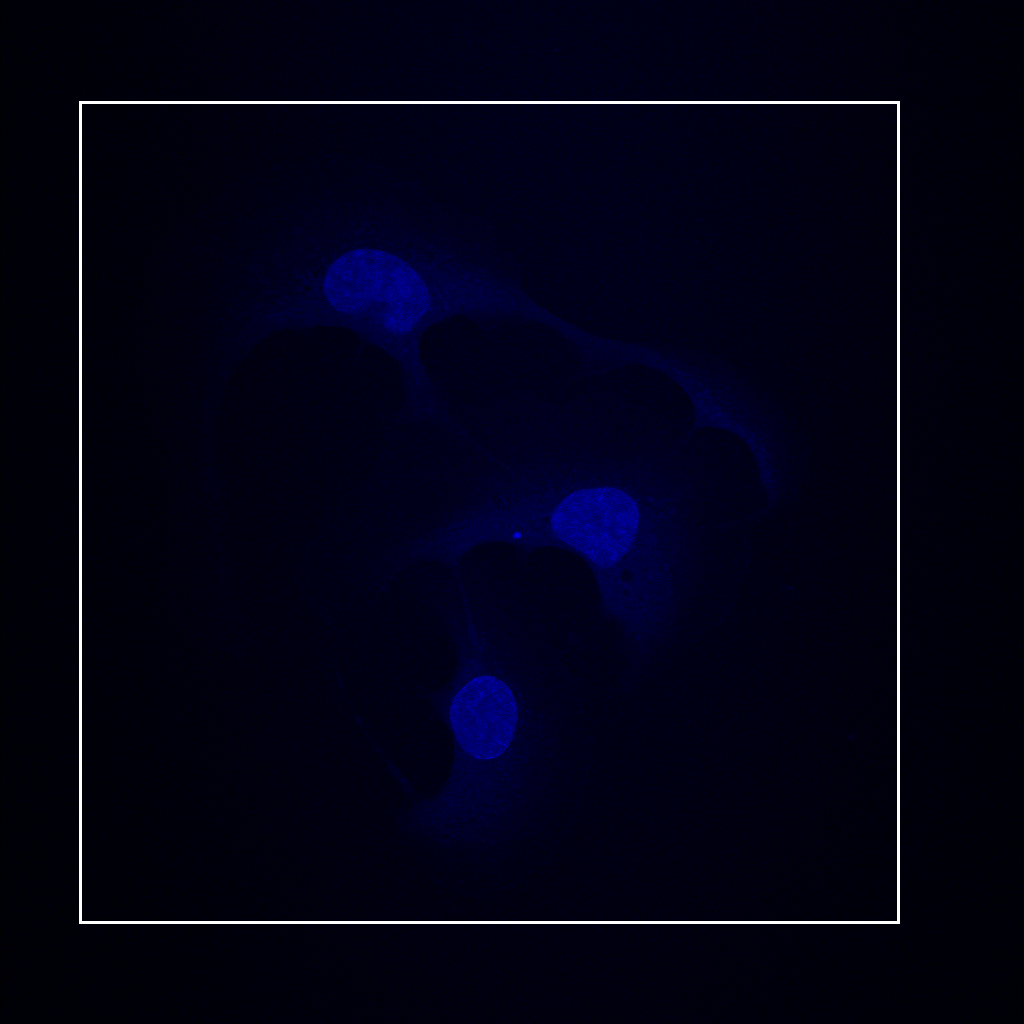

Supplement: Supplementary file 5 — Source data Fig. 3 [file 44319_2024_203_MOESM5_ESM.zip › 3B/KRAS/Hoechst.tif]

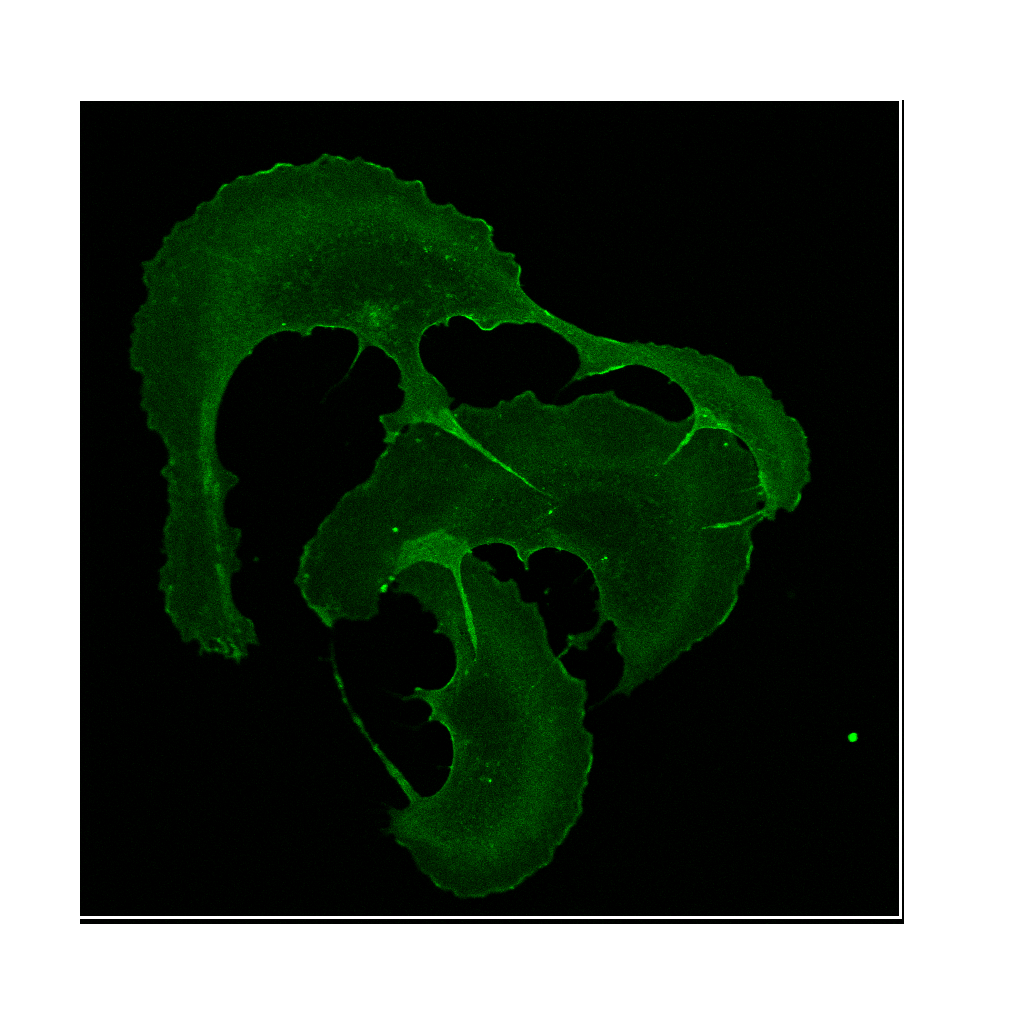

Supplement: Supplementary file 5 — Source data Fig. 3 [file 44319_2024_203_MOESM5_ESM.zip › 3B/KRAS/KRAS.tif]

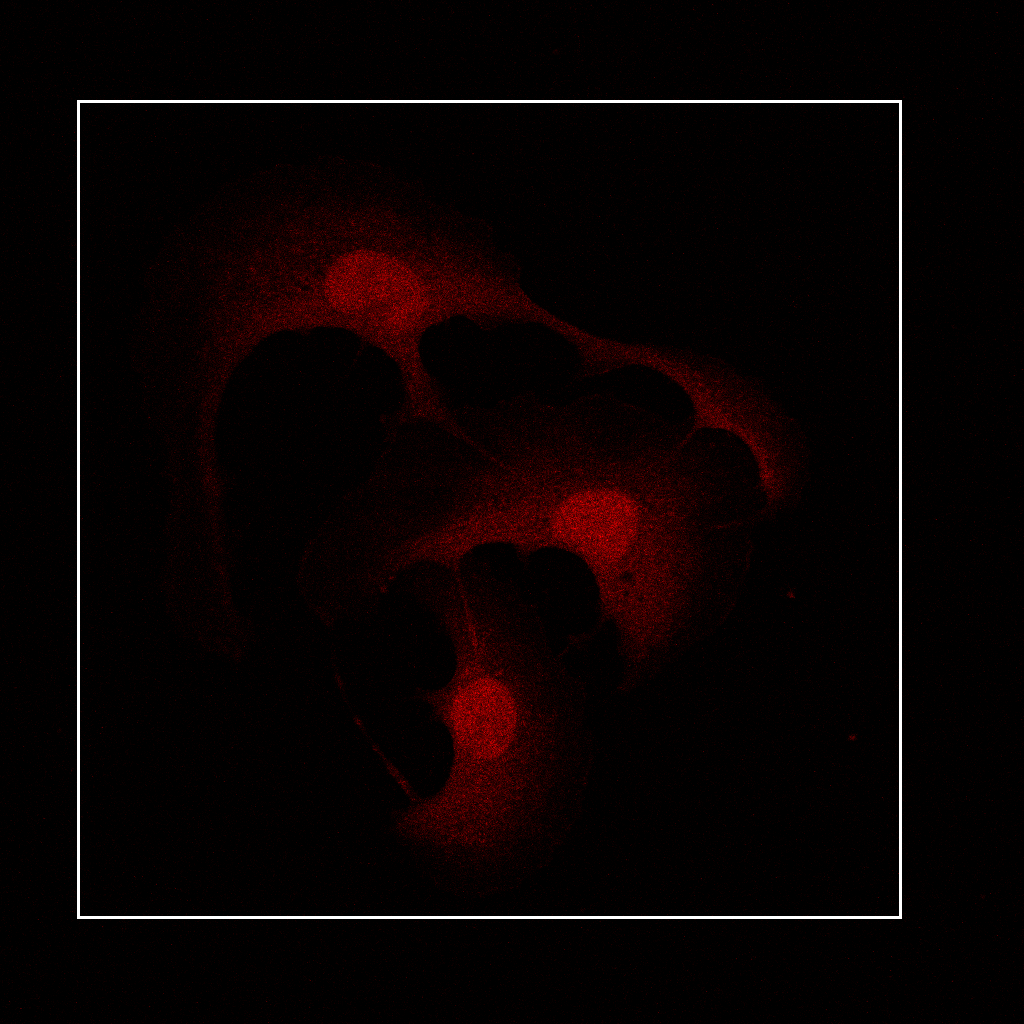

Supplement: Supplementary file 5 — Source data Fig. 3 [file 44319_2024_203_MOESM5_ESM.zip › 3B/KRAS/YAP.tif]

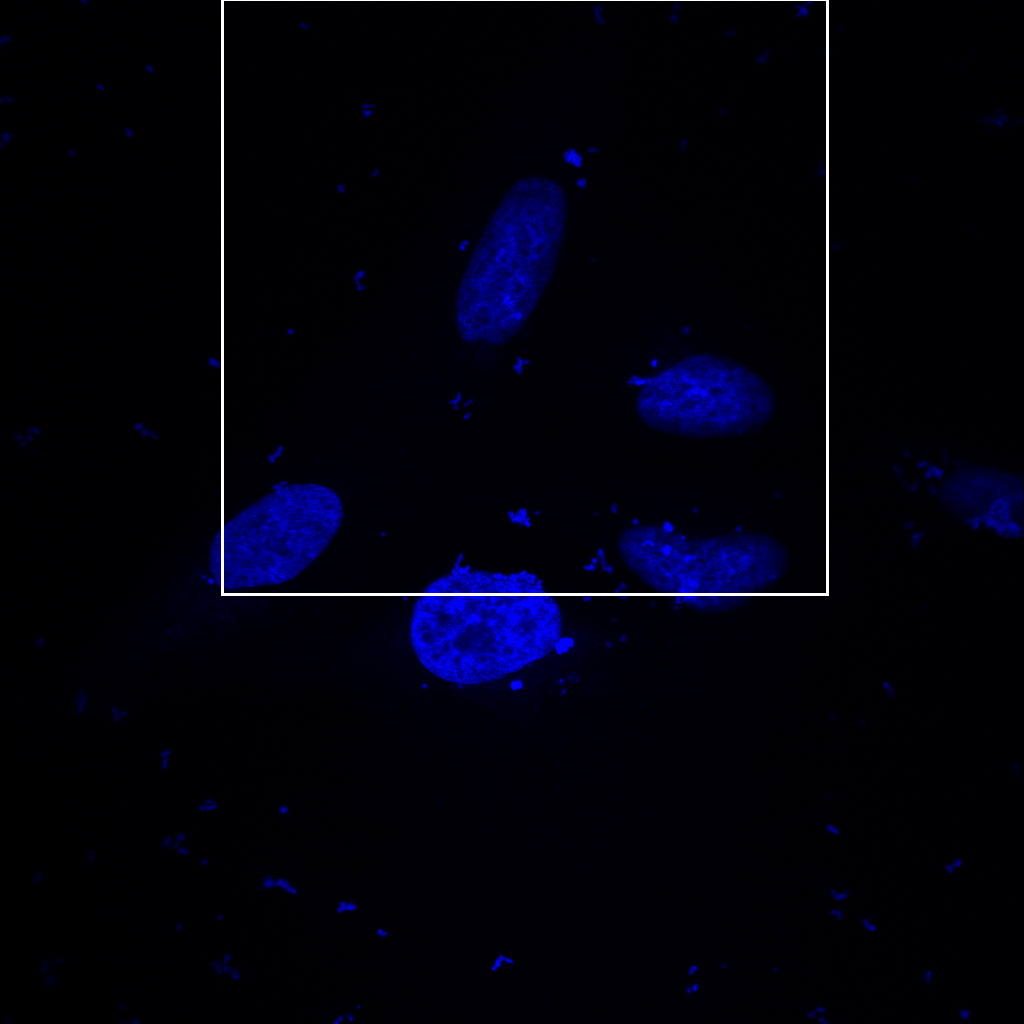

Supplement: Supplementary file 5 — Source data Fig. 3 [file 44319_2024_203_MOESM5_ESM.zip › 3B/RASSF5/Hoechst.tif]

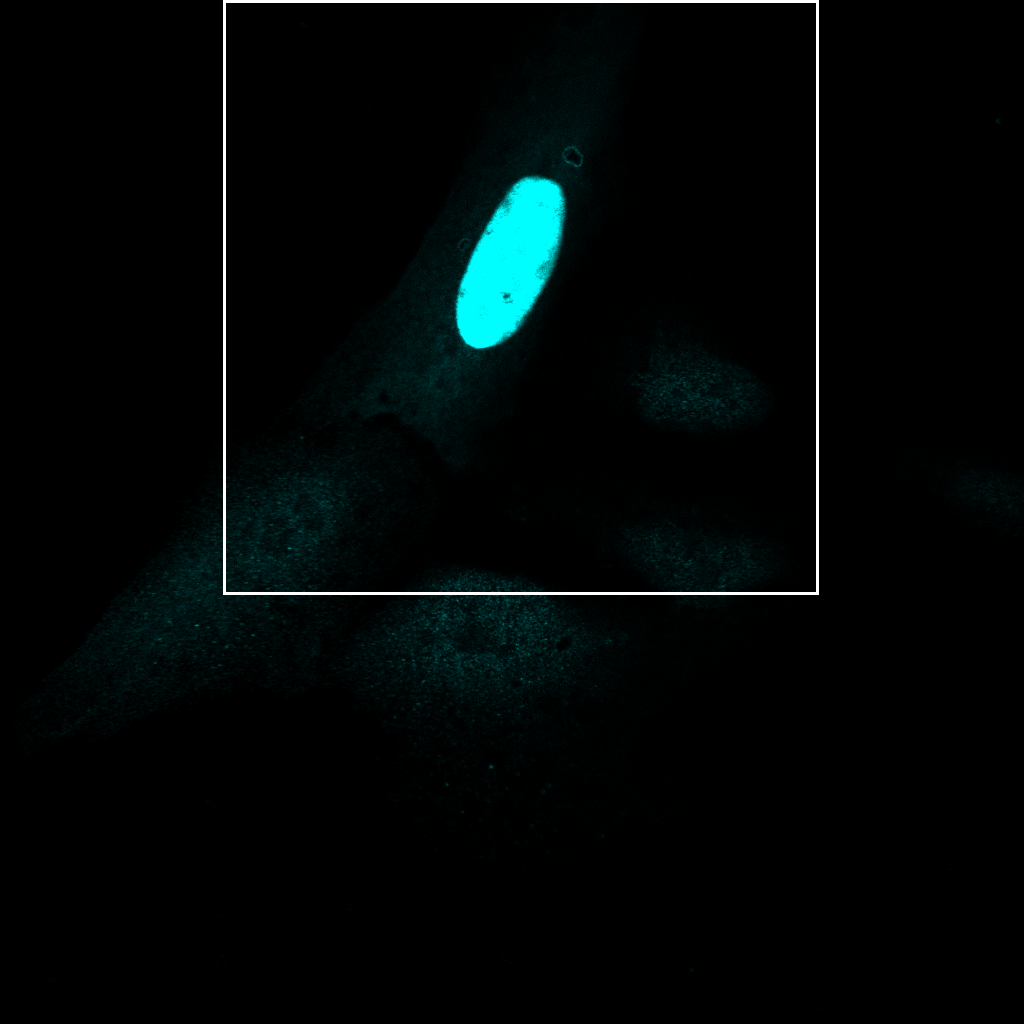

Supplement: Supplementary file 5 — Source data Fig. 3 [file 44319_2024_203_MOESM5_ESM.zip › 3B/RASSF5/RASSF5.tif]

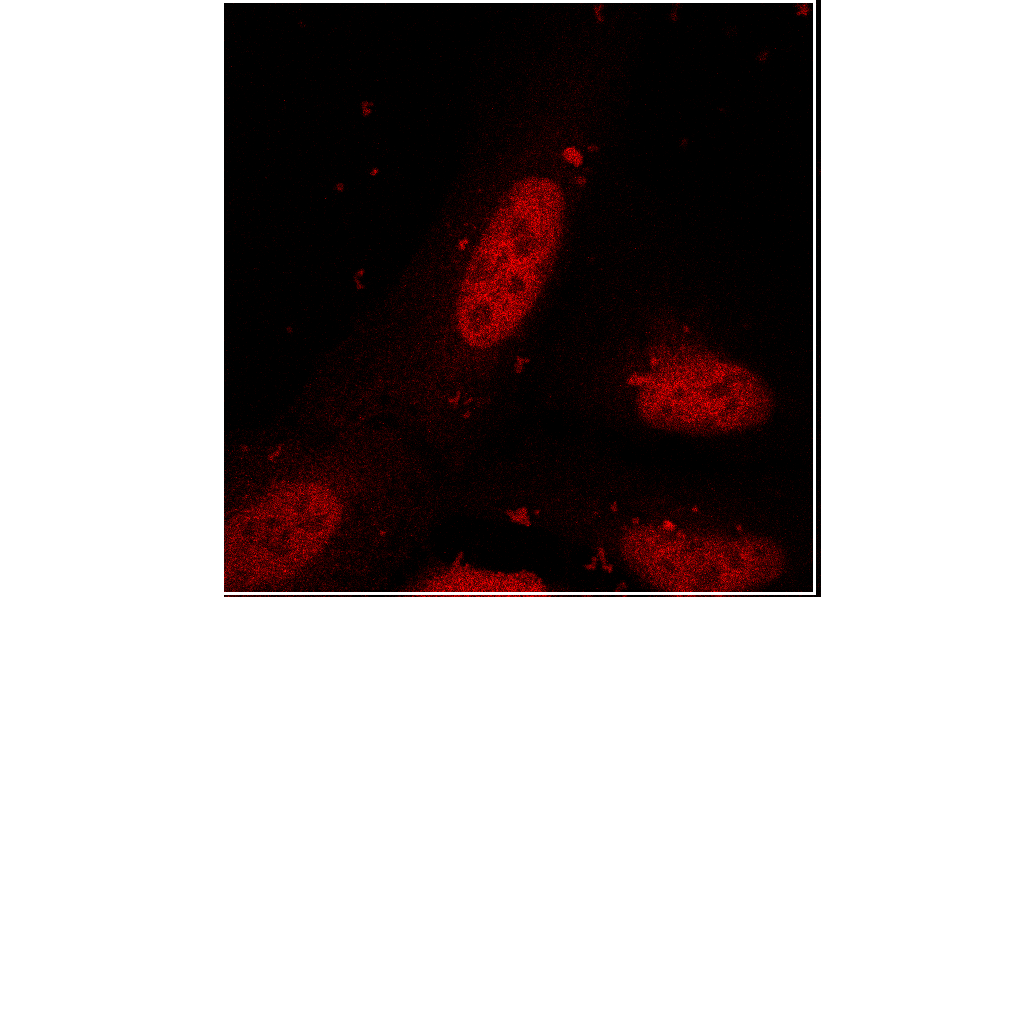

Supplement: Supplementary file 5 — Source data Fig. 3 [file 44319_2024_203_MOESM5_ESM.zip › 3B/RASSF5/YAP.tif]

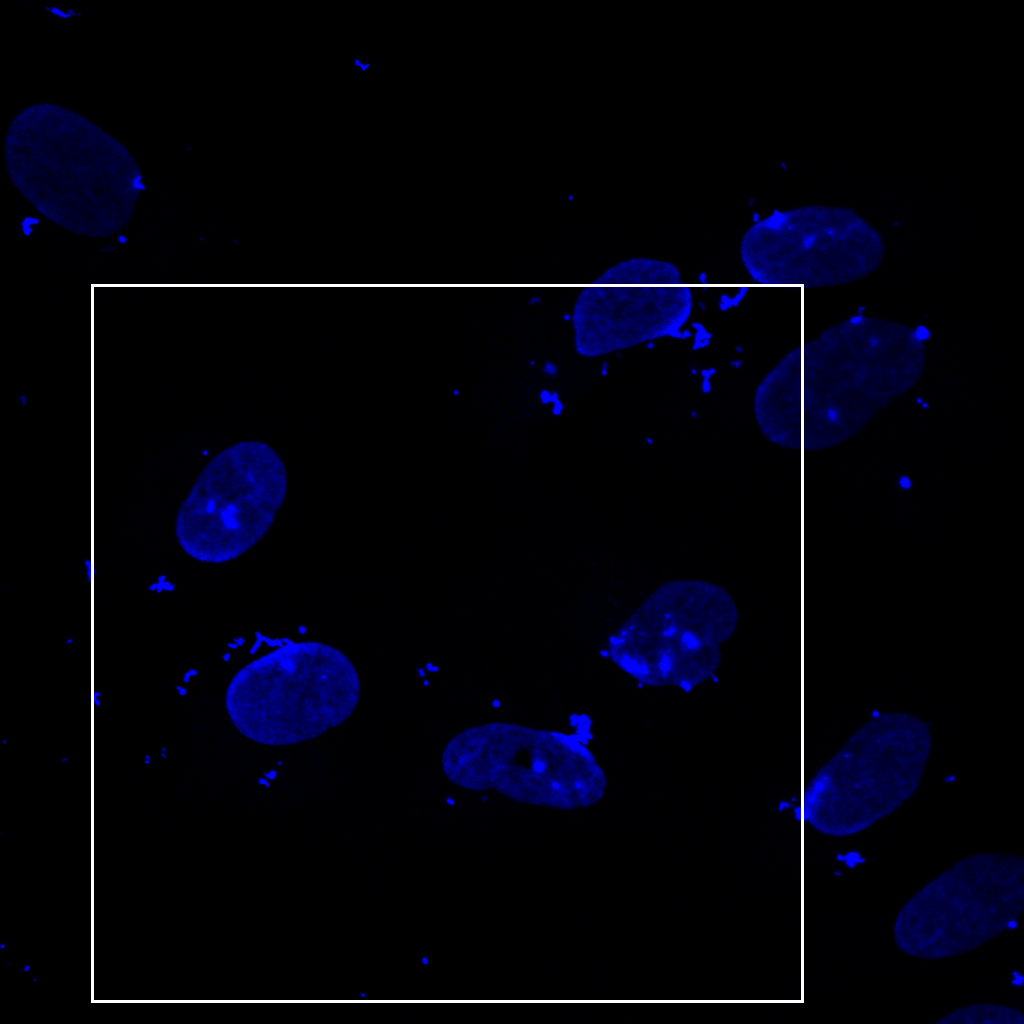

Supplement: Supplementary file 5 — Source data Fig. 3 [file 44319_2024_203_MOESM5_ESM.zip › 3B/RIT2/Hoechst.tif]

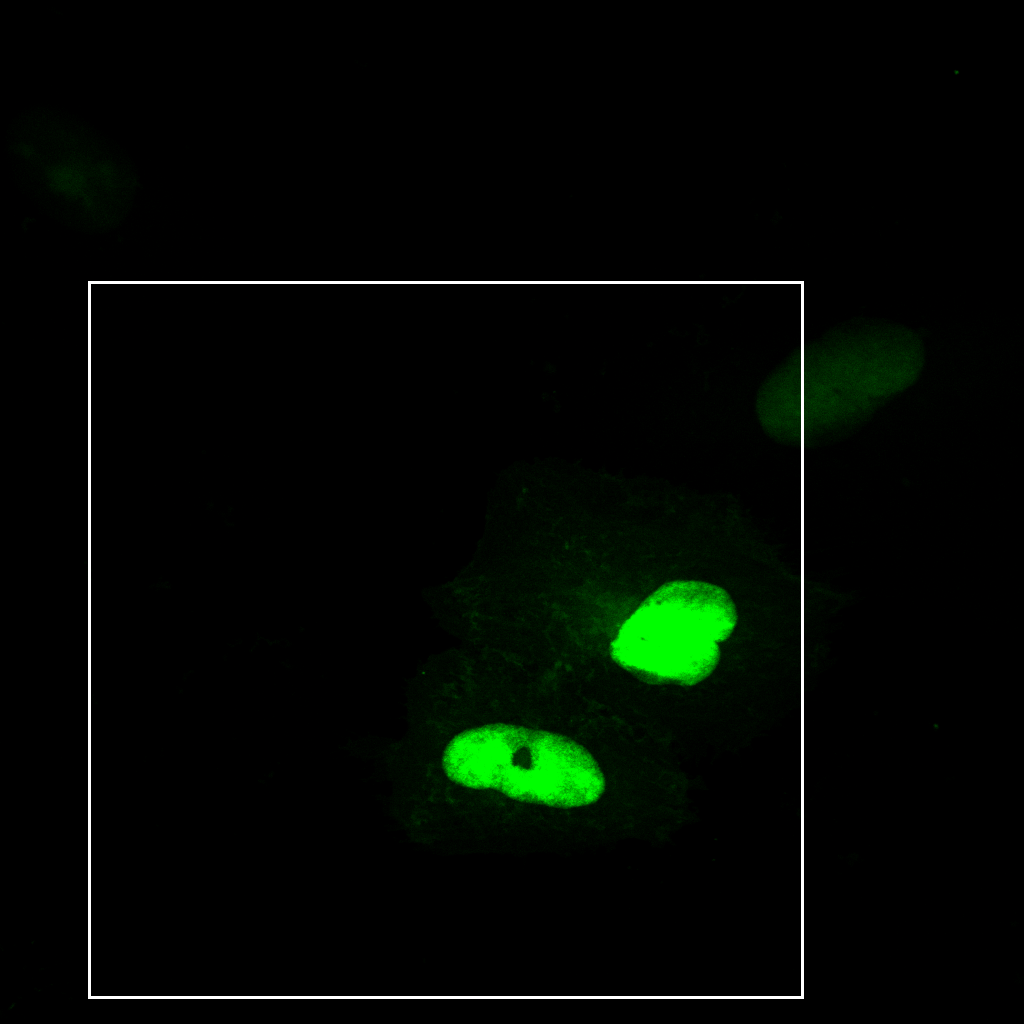

Supplement: Supplementary file 5 — Source data Fig. 3 [file 44319_2024_203_MOESM5_ESM.zip › 3B/RIT2/RIT2.tif]

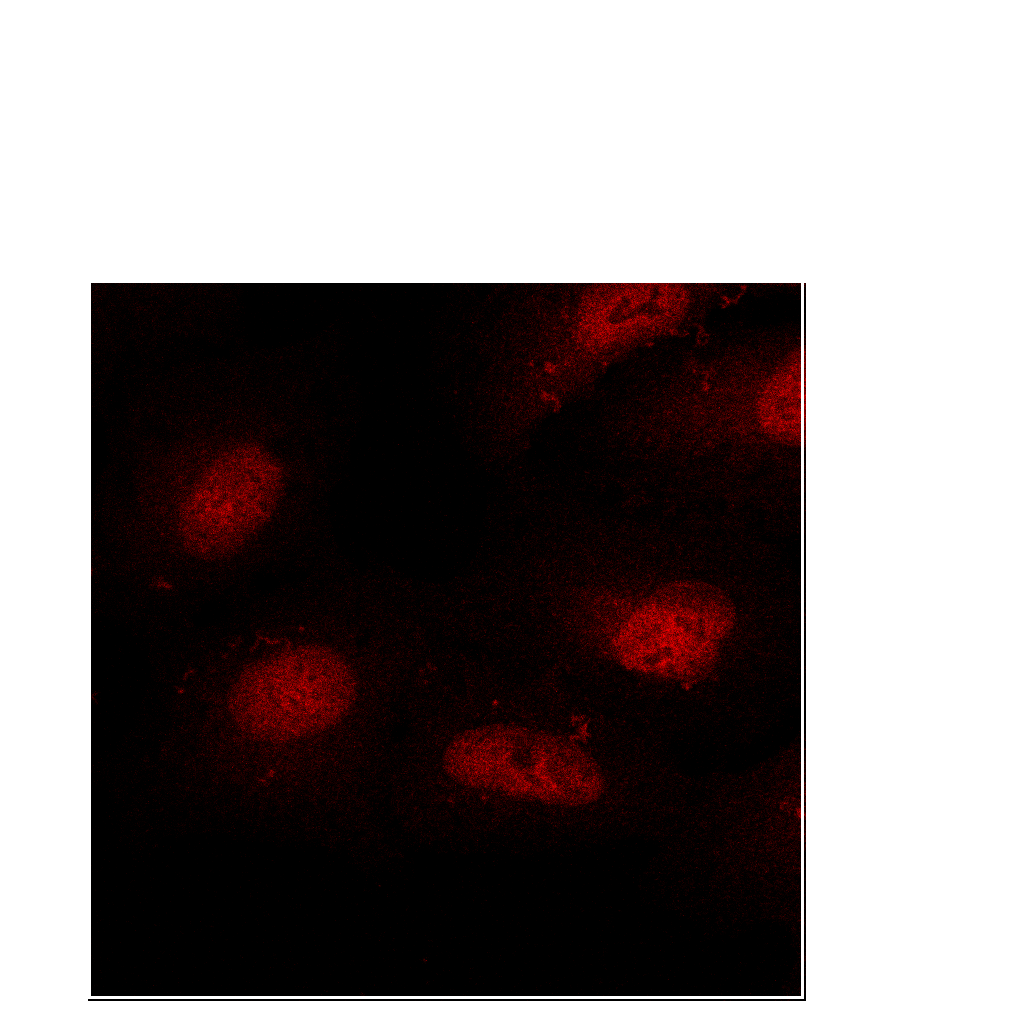

Supplement: Supplementary file 5 — Source data Fig. 3 [file 44319_2024_203_MOESM5_ESM.zip › 3B/RIT2/YAP.tif]

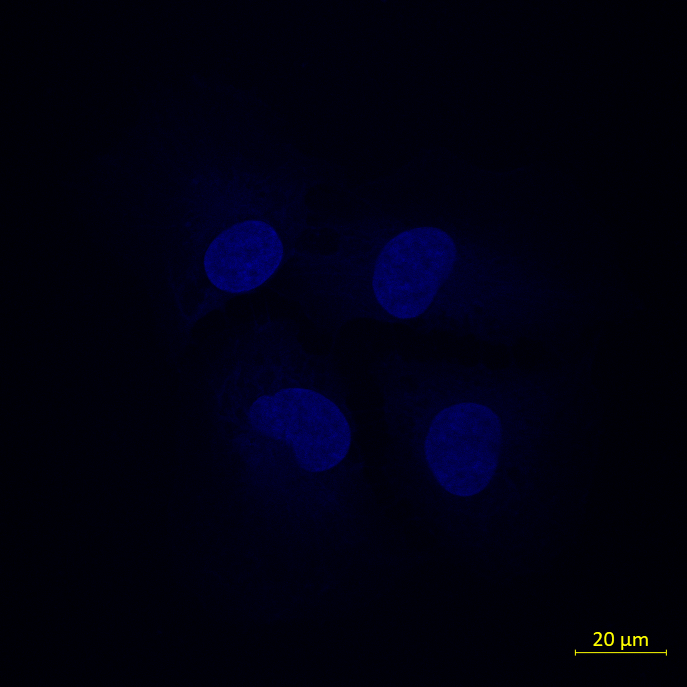

Supplement: Supplementary file 5 — Source data Fig. 3 [file 44319_2024_203_MOESM5_ESM.zip › 3B/RRAS/Hoechst.tif]

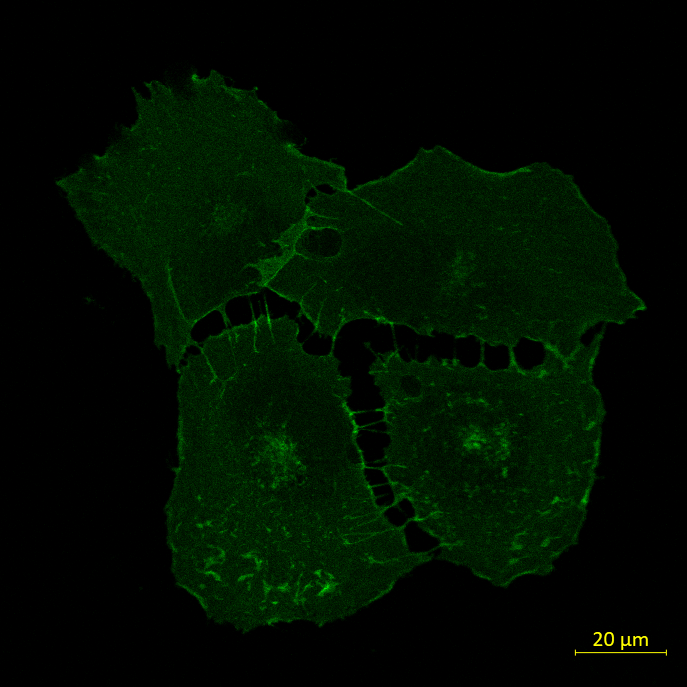

Supplement: Supplementary file 5 — Source data Fig. 3 [file 44319_2024_203_MOESM5_ESM.zip › 3B/RRAS/RRAS.tif]

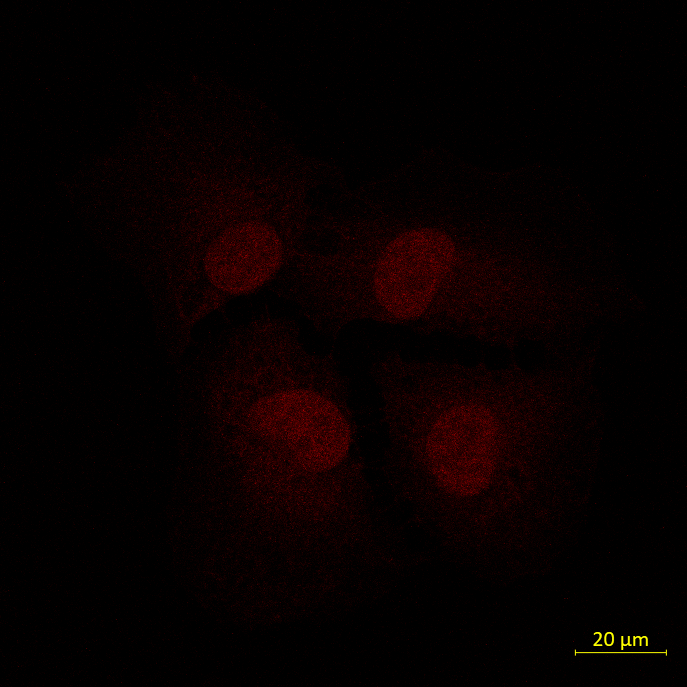

Supplement: Supplementary file 5 — Source data Fig. 3 [file 44319_2024_203_MOESM5_ESM.zip › 3B/RRAS/YAP.tif]

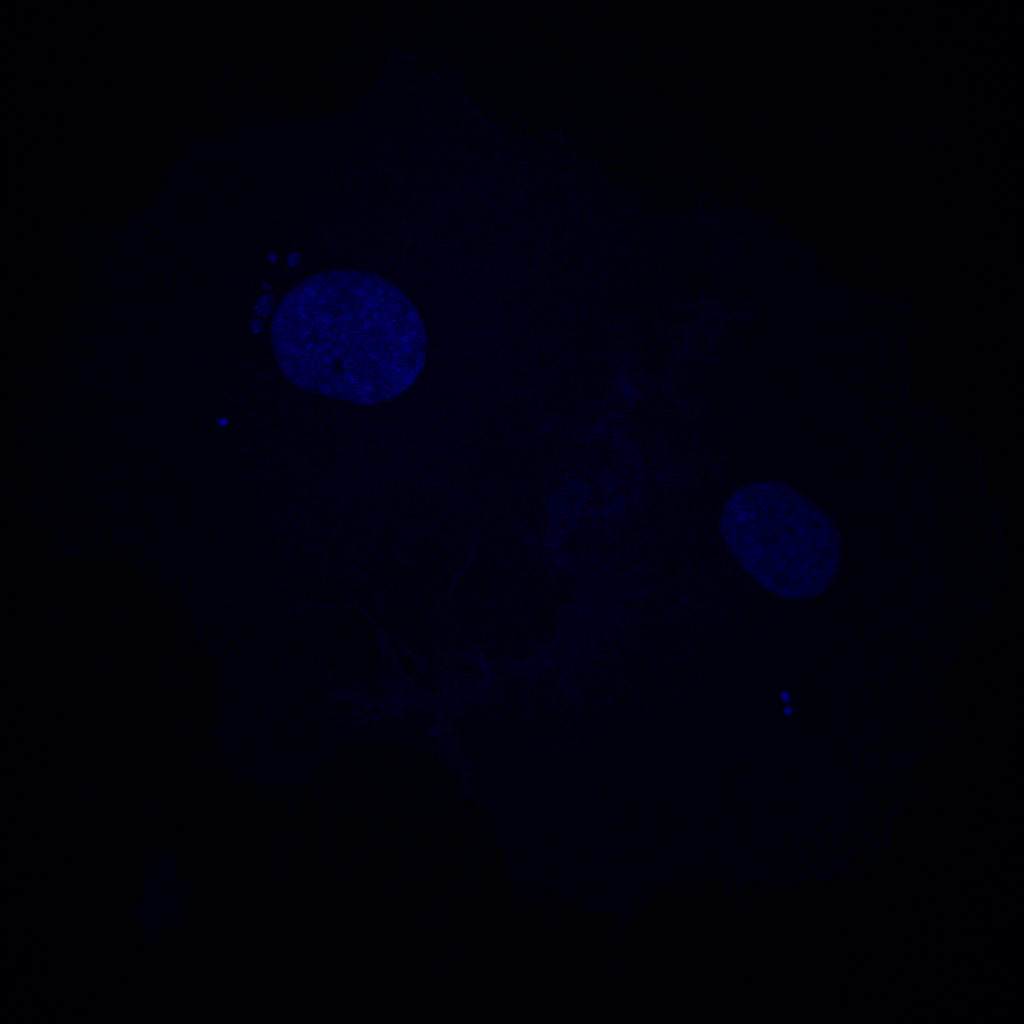

Supplement: Supplementary file 5 — Source data Fig. 3 [file 44319_2024_203_MOESM5_ESM.zip › 3C/KRAS+RASSF5/Hoechst.tif]

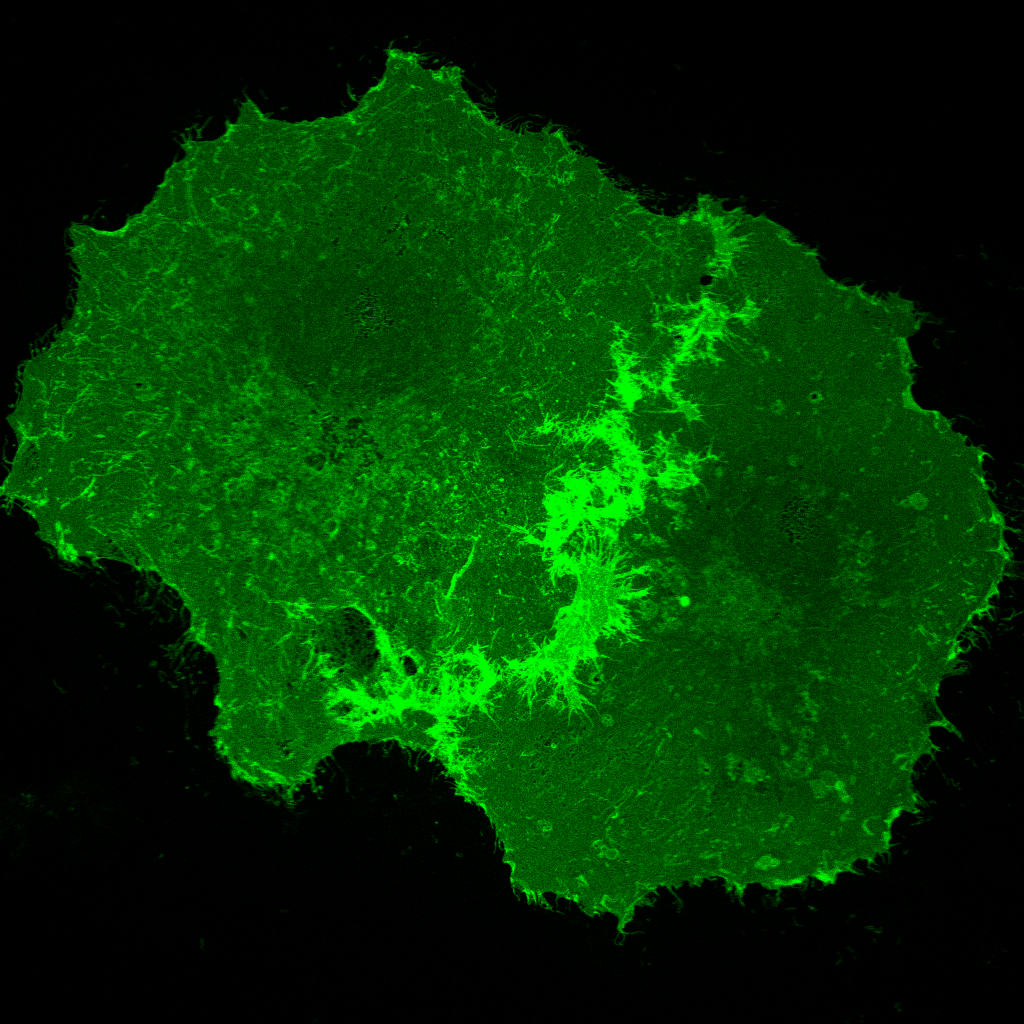

Supplement: Supplementary file 5 — Source data Fig. 3 [file 44319_2024_203_MOESM5_ESM.zip › 3C/KRAS+RASSF5/KRAS.tif]

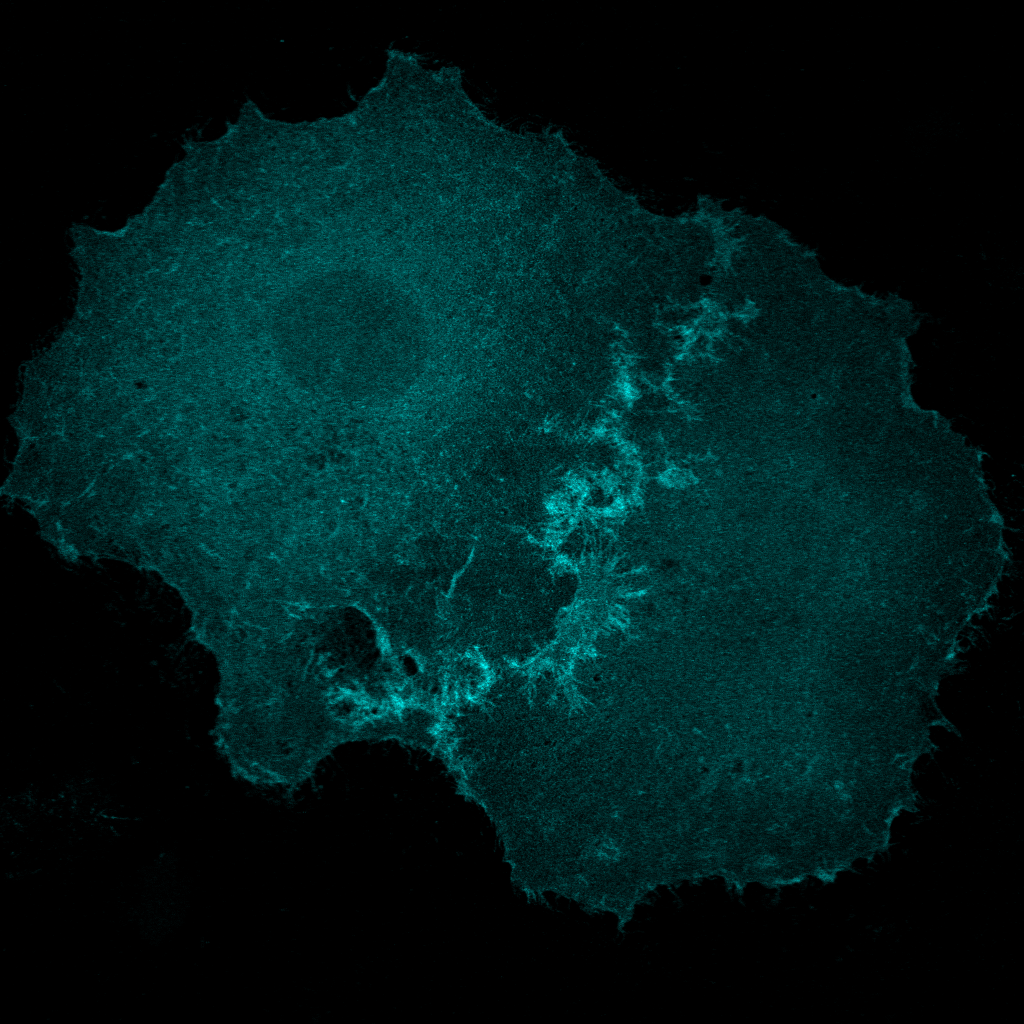

Supplement: Supplementary file 5 — Source data Fig. 3 [file 44319_2024_203_MOESM5_ESM.zip › 3C/KRAS+RASSF5/RASSF5.tif]

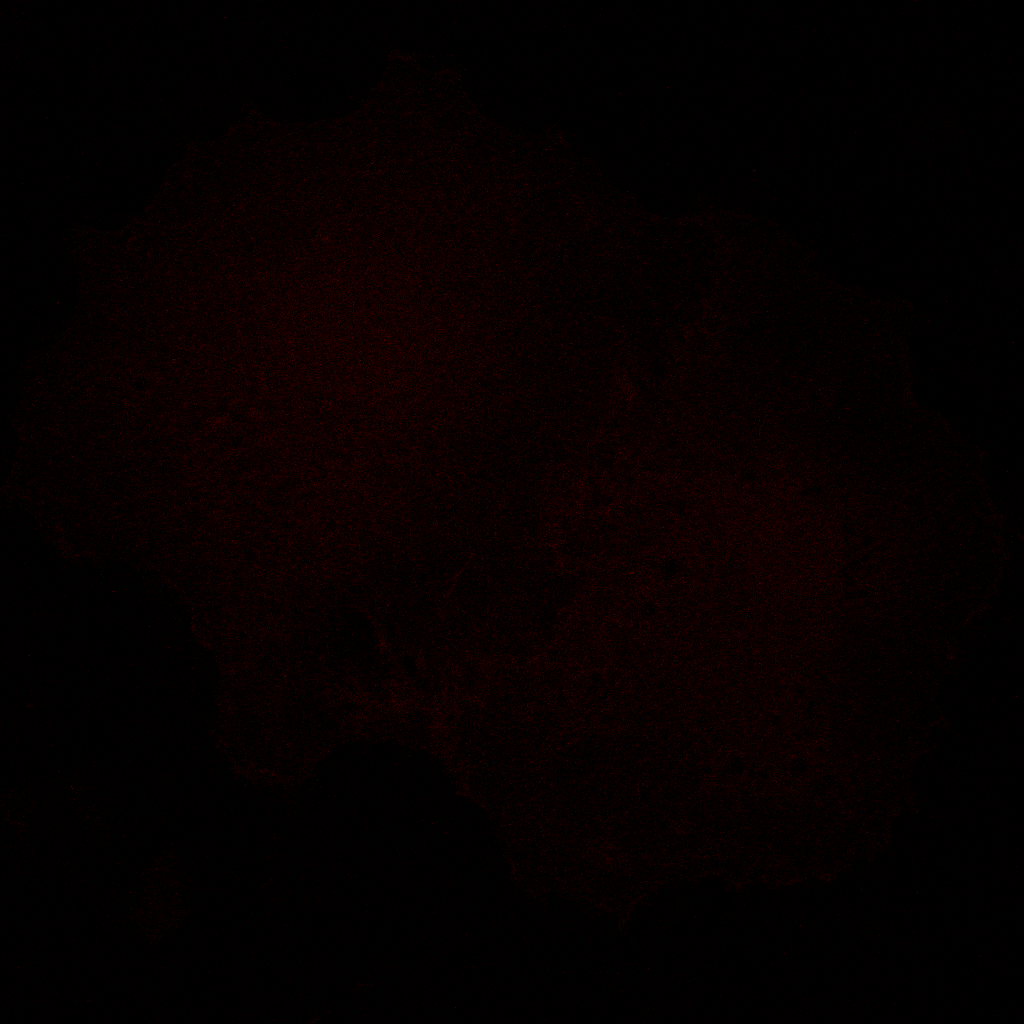

Supplement: Supplementary file 5 — Source data Fig. 3 [file 44319_2024_203_MOESM5_ESM.zip › 3C/KRAS+RASSF5/YAP.tif]

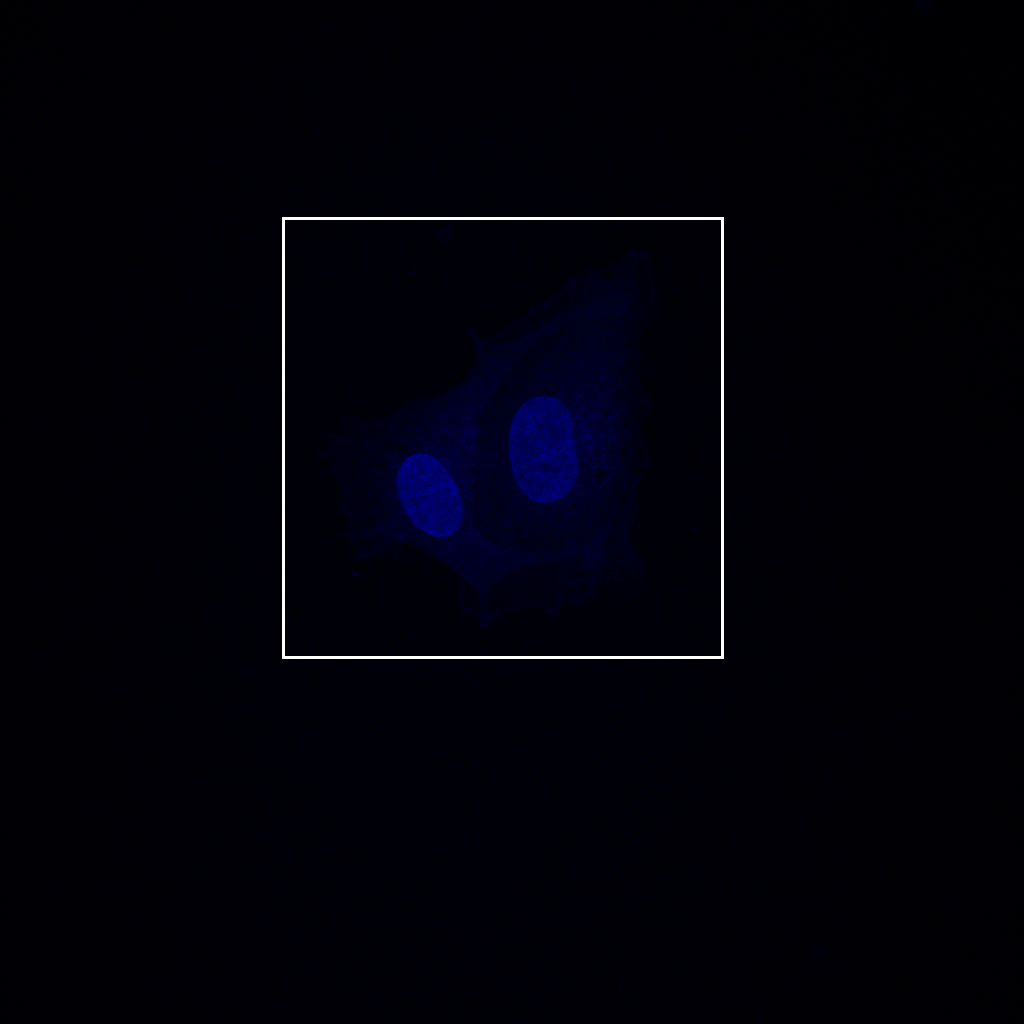

Supplement: Supplementary file 5 — Source data Fig. 3 [file 44319_2024_203_MOESM5_ESM.zip › 3C/NRAS+RASSF5/Hoechst.tif]

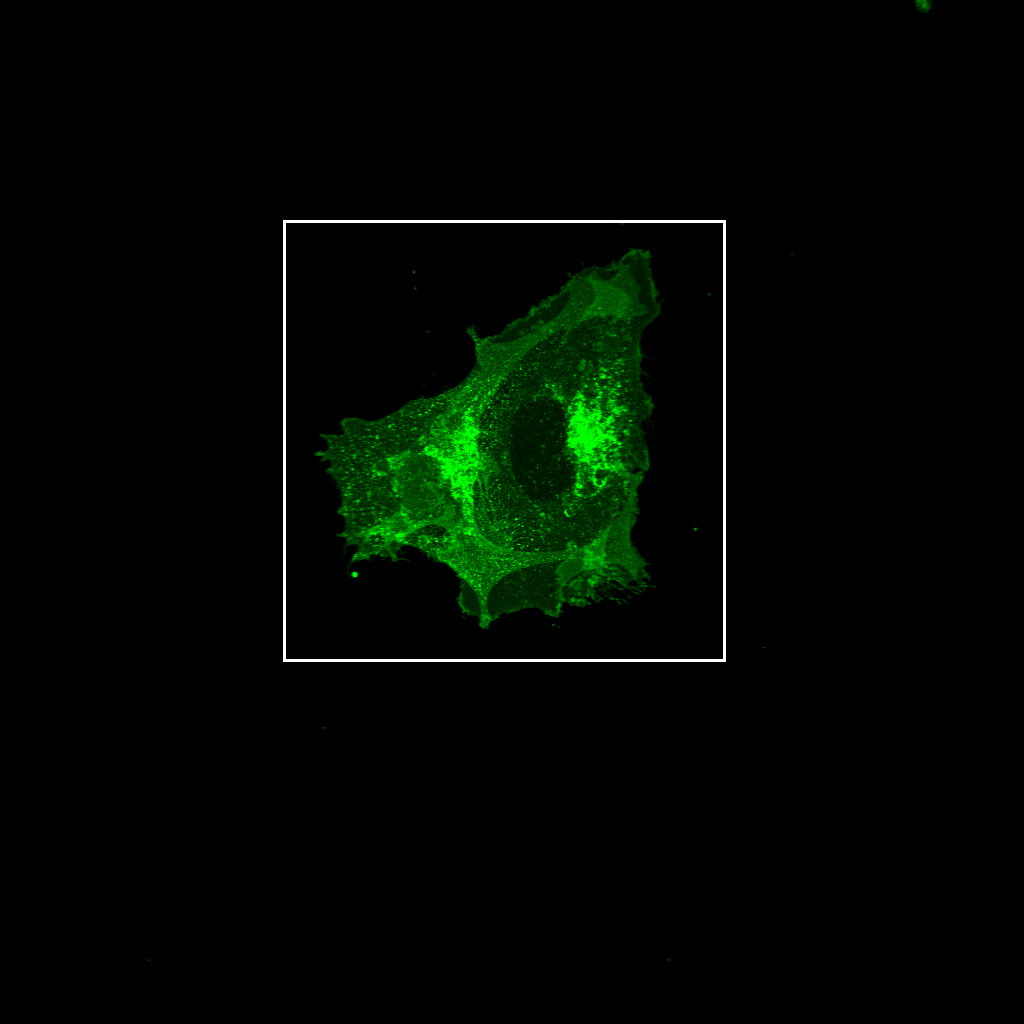

Supplement: Supplementary file 5 — Source data Fig. 3 [file 44319_2024_203_MOESM5_ESM.zip › 3C/NRAS+RASSF5/NRAS.tif]

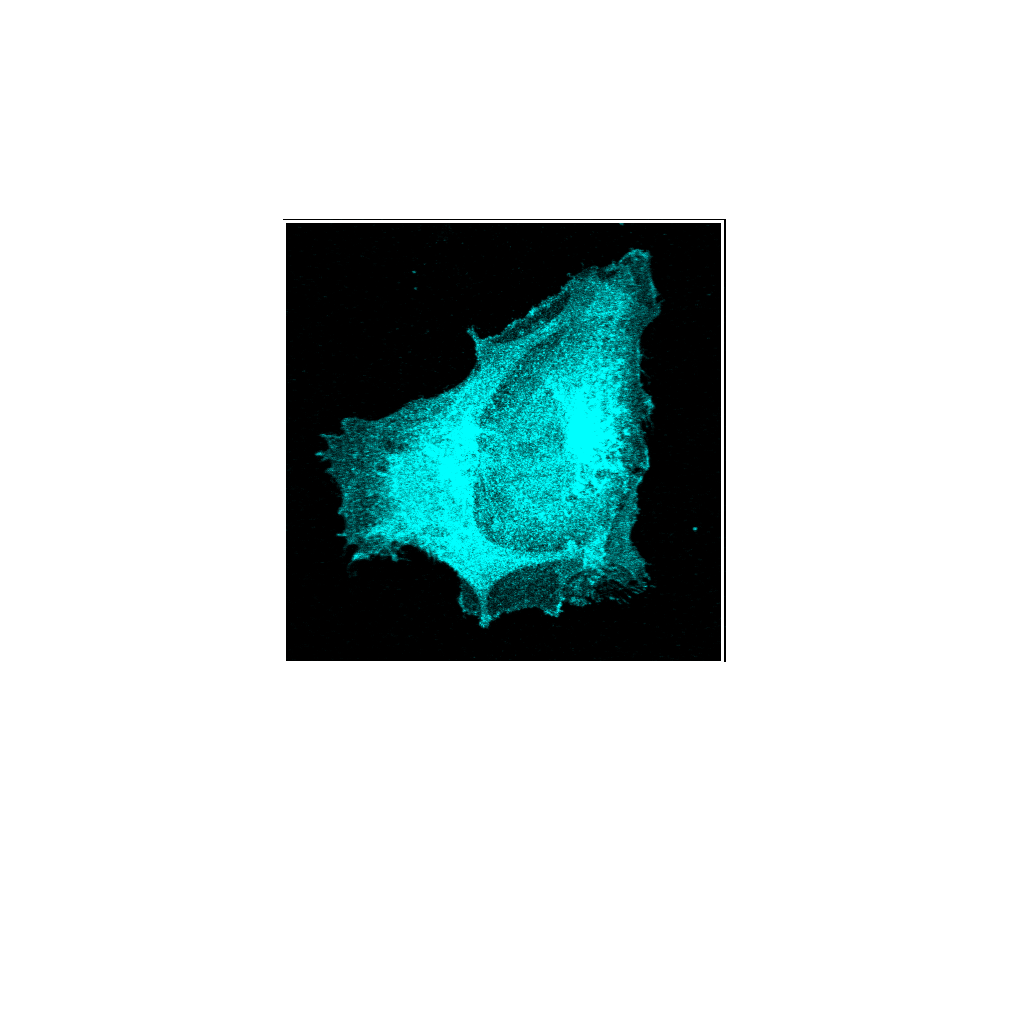

Supplement: Supplementary file 5 — Source data Fig. 3 [file 44319_2024_203_MOESM5_ESM.zip › 3C/NRAS+RASSF5/RASSF5.tif]

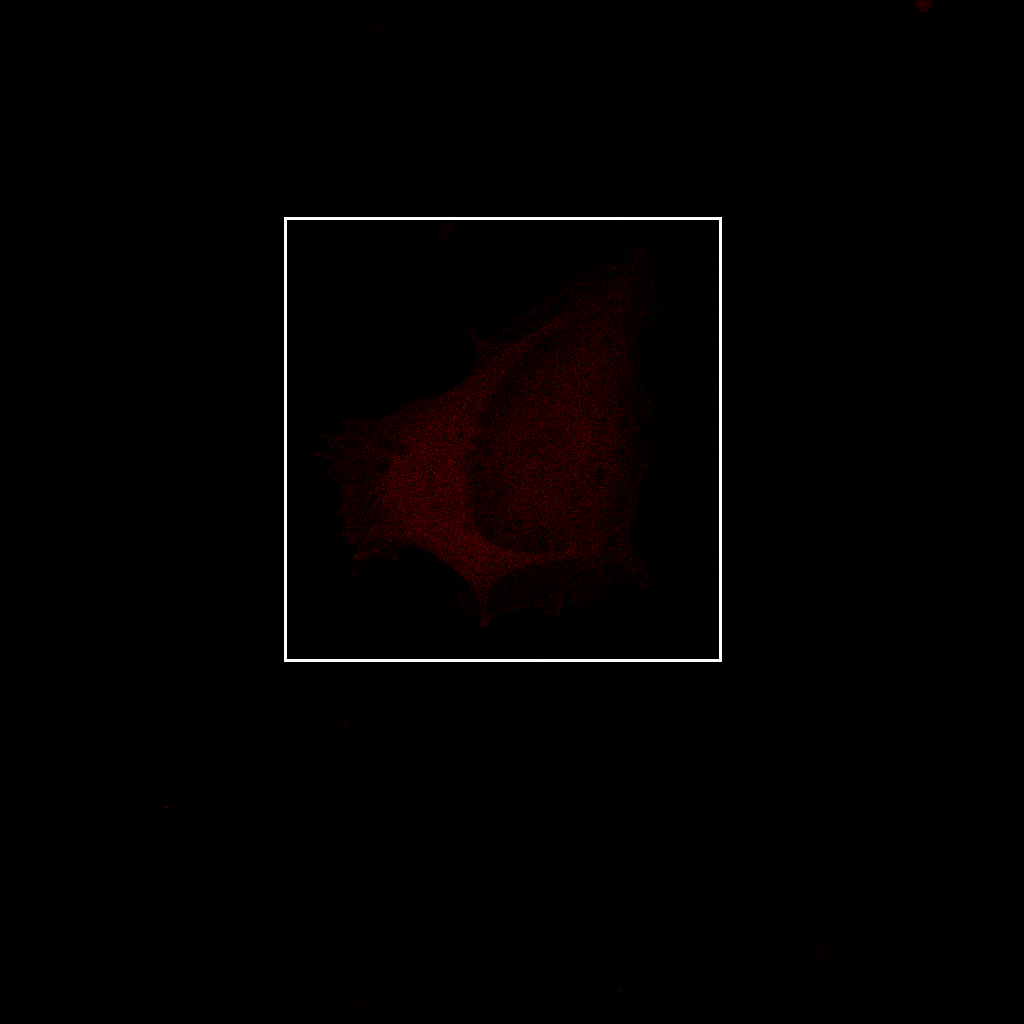

Supplement: Supplementary file 5 — Source data Fig. 3 [file 44319_2024_203_MOESM5_ESM.zip › 3C/NRAS+RASSF5/YAP.tif]

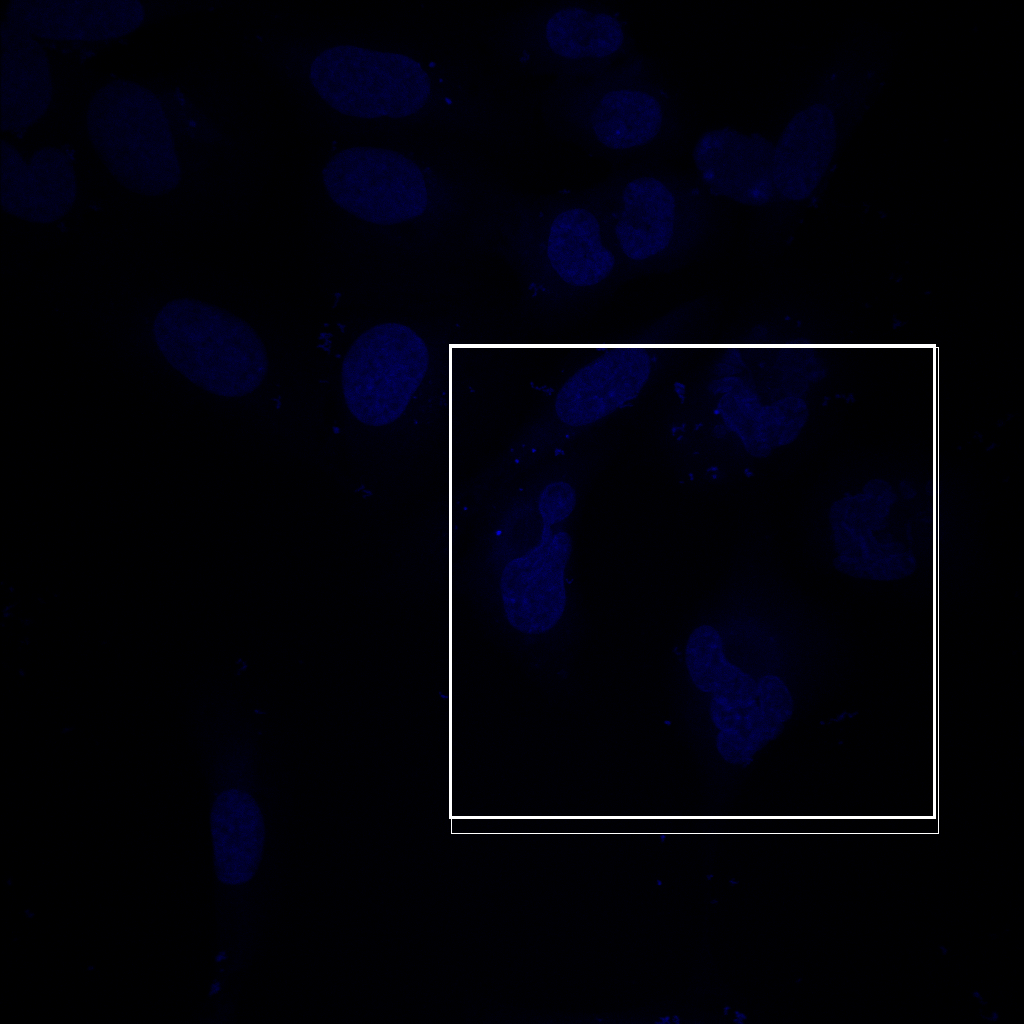

Supplement: Supplementary file 5 — Source data Fig. 3 [file 44319_2024_203_MOESM5_ESM.zip › 3C/RAP2B+RASSF5/Hoechst.tif]

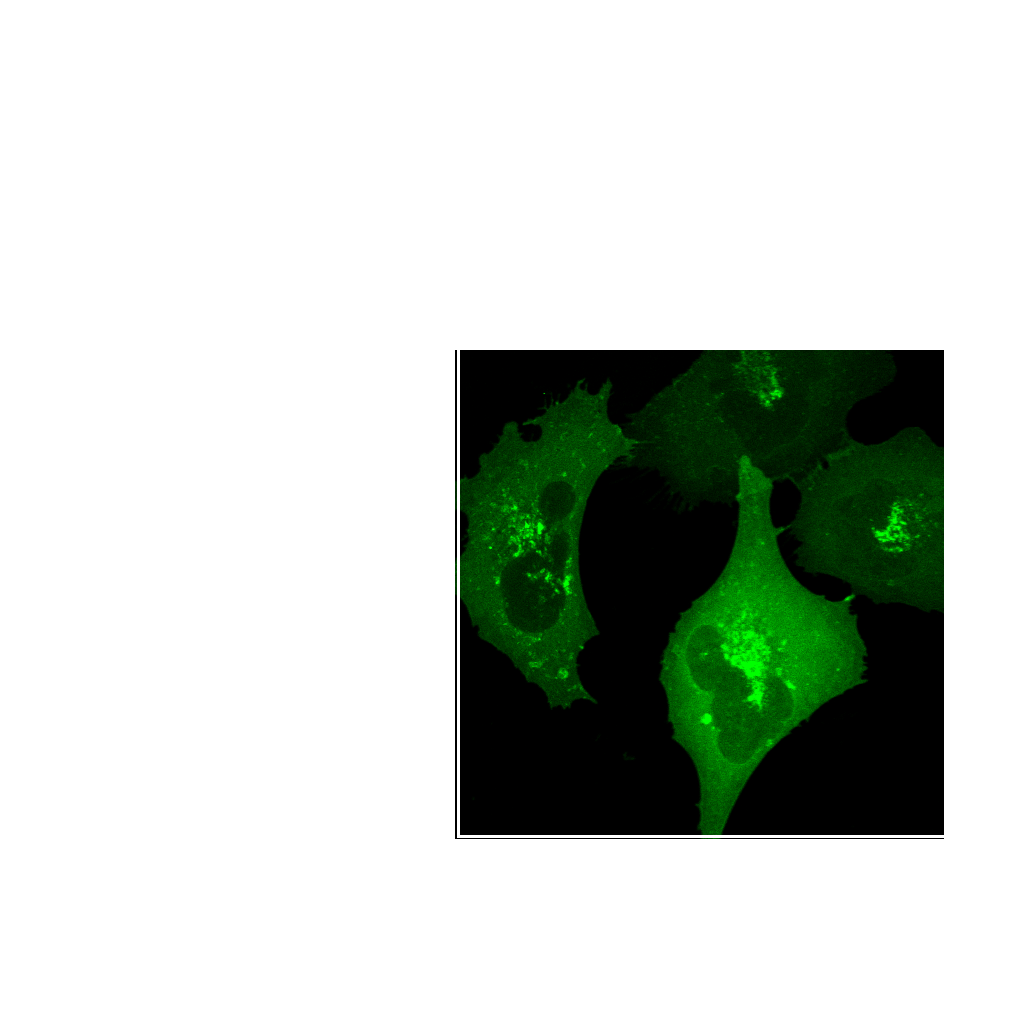

Supplement: Supplementary file 5 — Source data Fig. 3 [file 44319_2024_203_MOESM5_ESM.zip › 3C/RAP2B+RASSF5/RAP2B.tif]

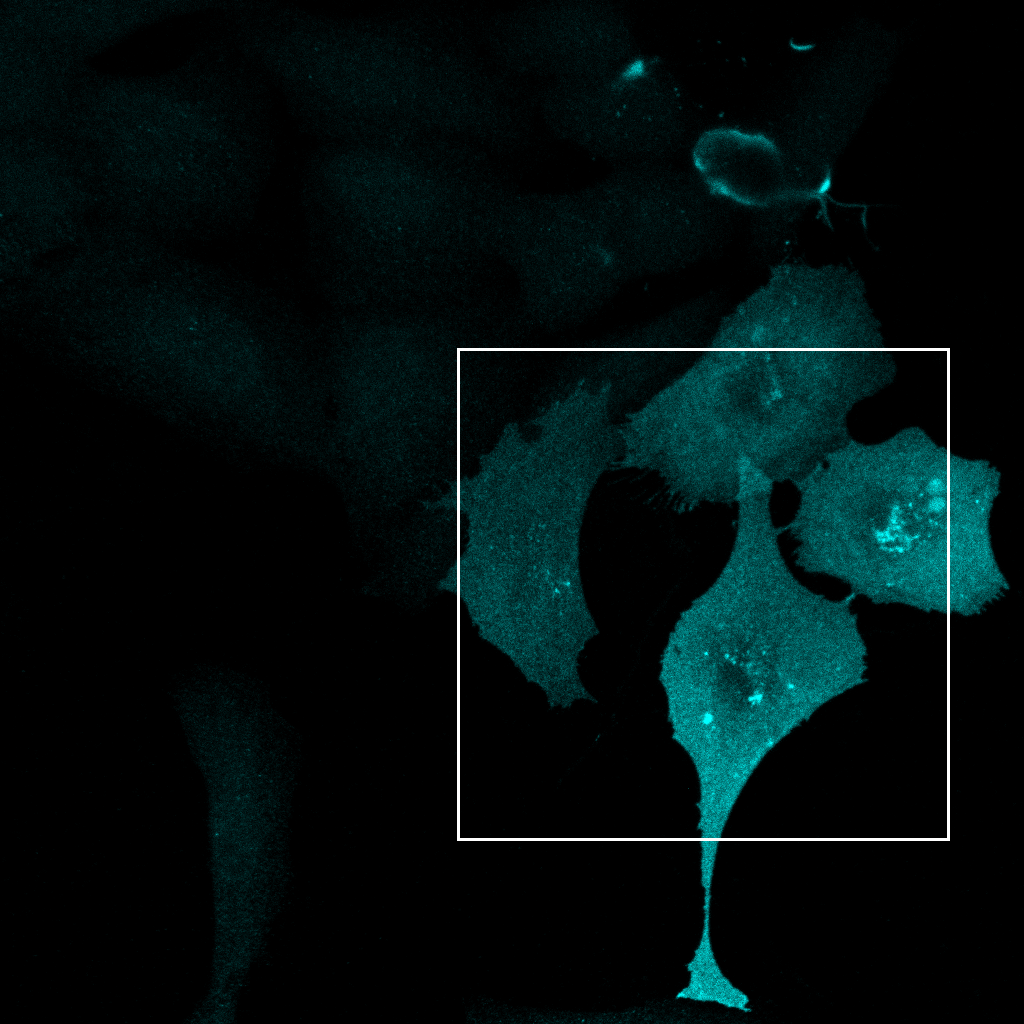

Supplement: Supplementary file 5 — Source data Fig. 3 [file 44319_2024_203_MOESM5_ESM.zip › 3C/RAP2B+RASSF5/RASSF5.tif]

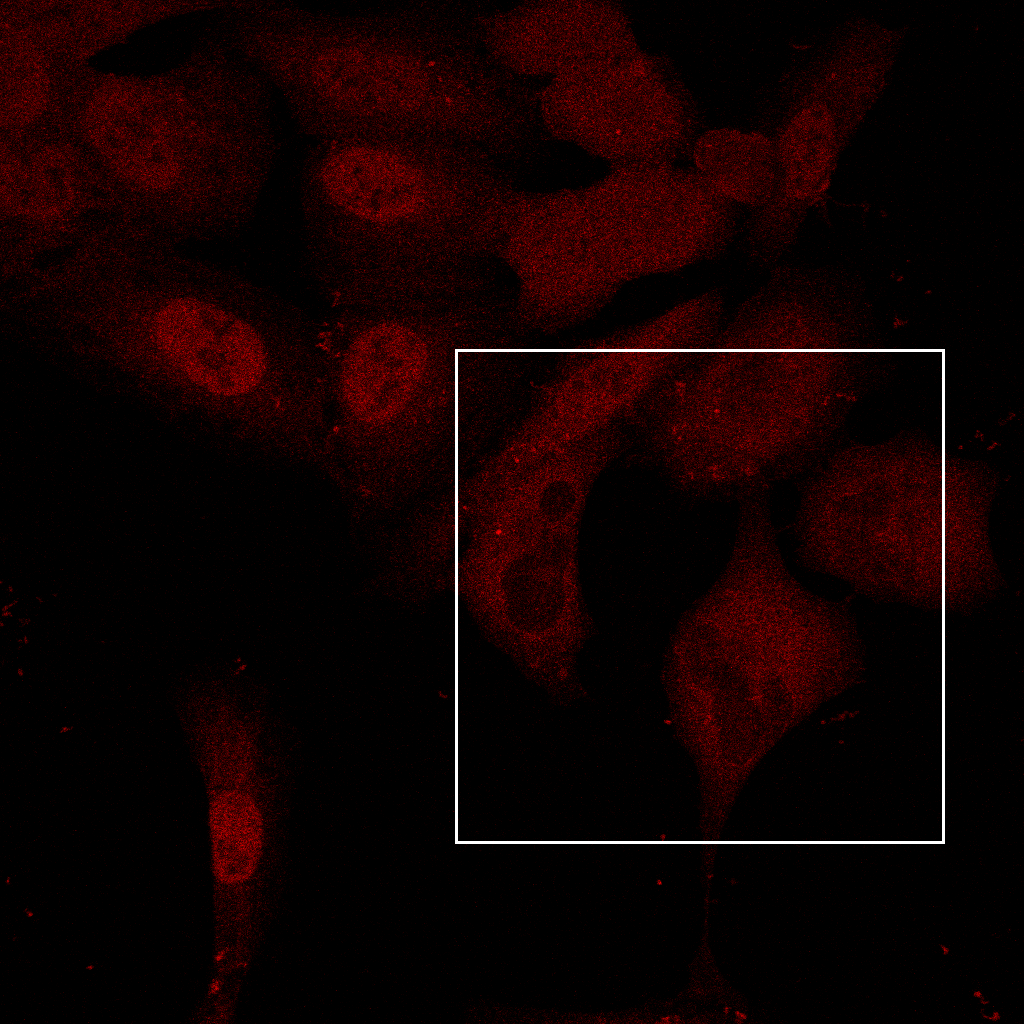

Supplement: Supplementary file 5 — Source data Fig. 3 [file 44319_2024_203_MOESM5_ESM.zip › 3C/RAP2B+RASSF5/YAP.tif]

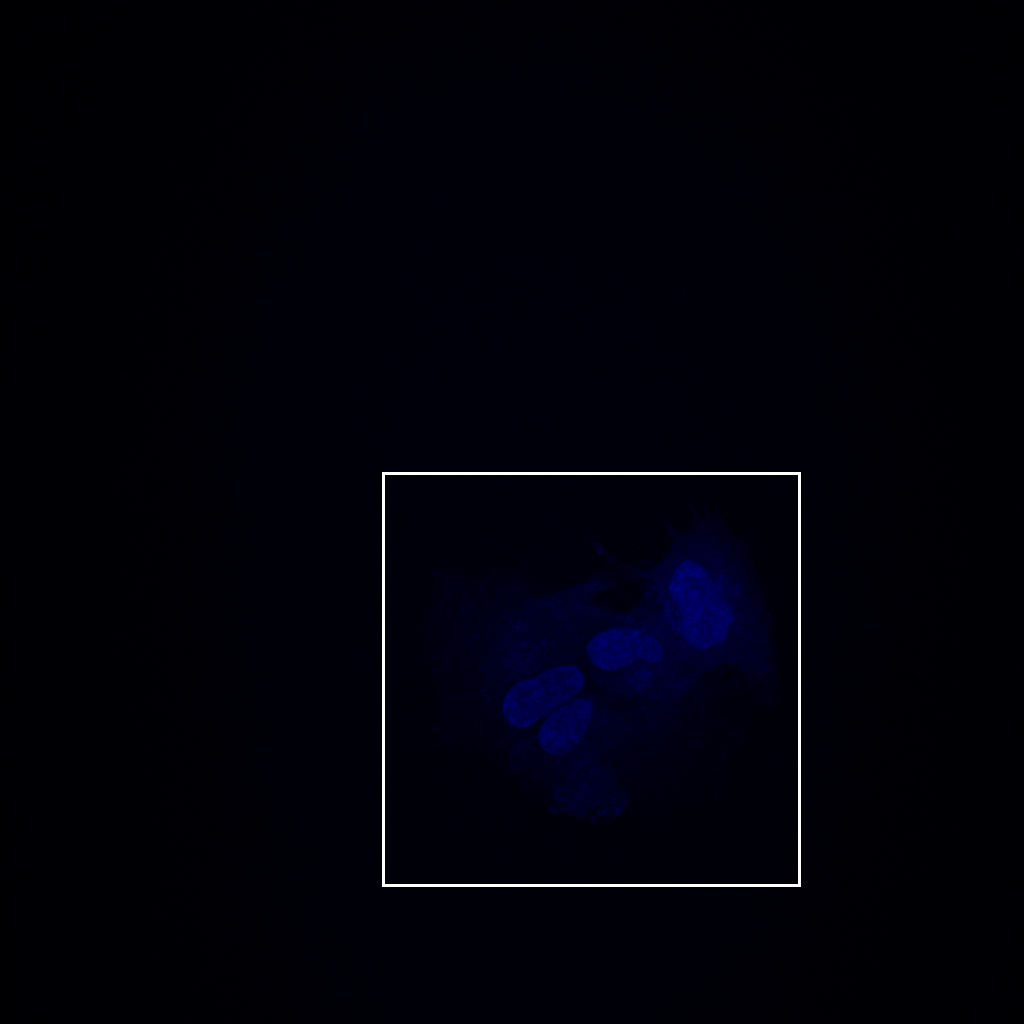

Supplement: Supplementary file 5 — Source data Fig. 3 [file 44319_2024_203_MOESM5_ESM.zip › 3C/RRAS+RASSF5/Hoechst.tif]

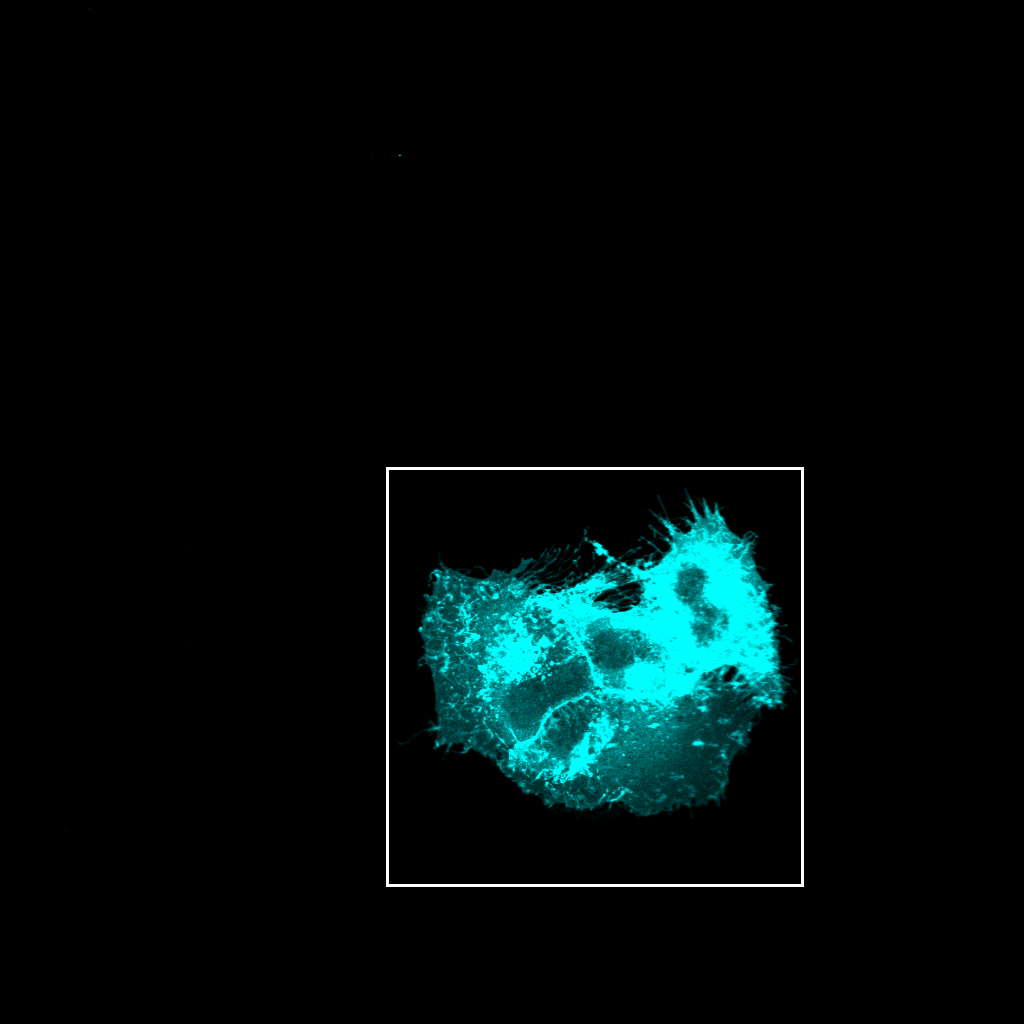

Supplement: Supplementary file 5 — Source data Fig. 3 [file 44319_2024_203_MOESM5_ESM.zip › 3C/RRAS+RASSF5/RASSF5.tif]

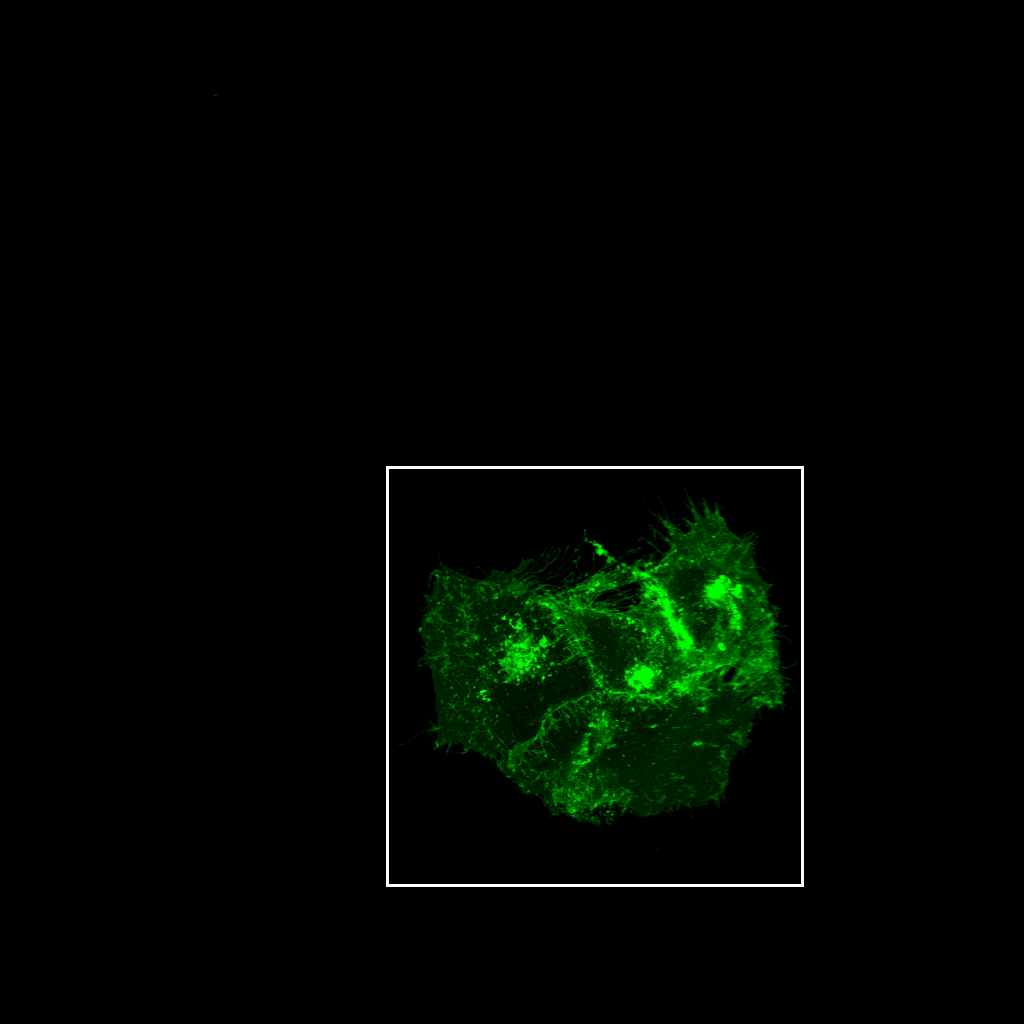

Supplement: Supplementary file 5 — Source data Fig. 3 [file 44319_2024_203_MOESM5_ESM.zip › 3C/RRAS+RASSF5/RRAS.tif]

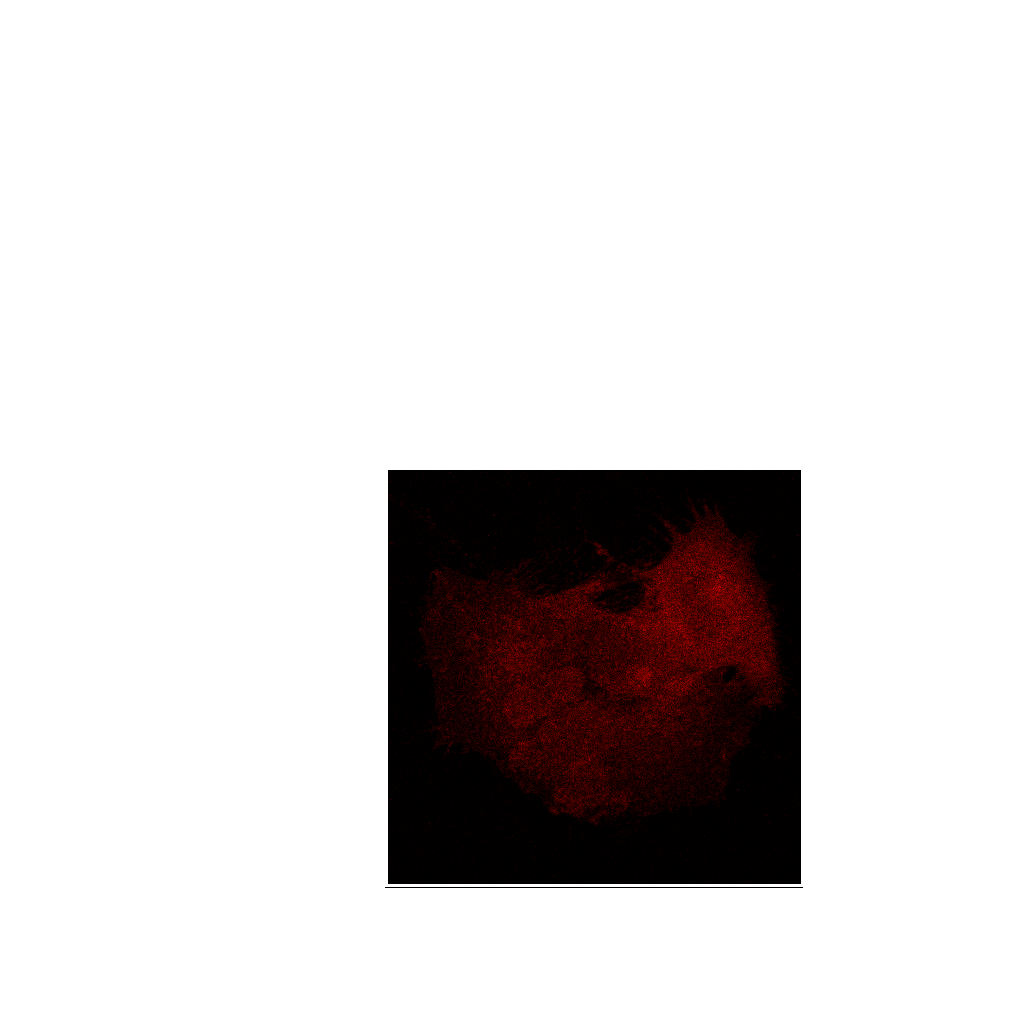

Supplement: Supplementary file 5 — Source data Fig. 3 [file 44319_2024_203_MOESM5_ESM.zip › 3C/RRAS+RASSF5/YAP.tif]

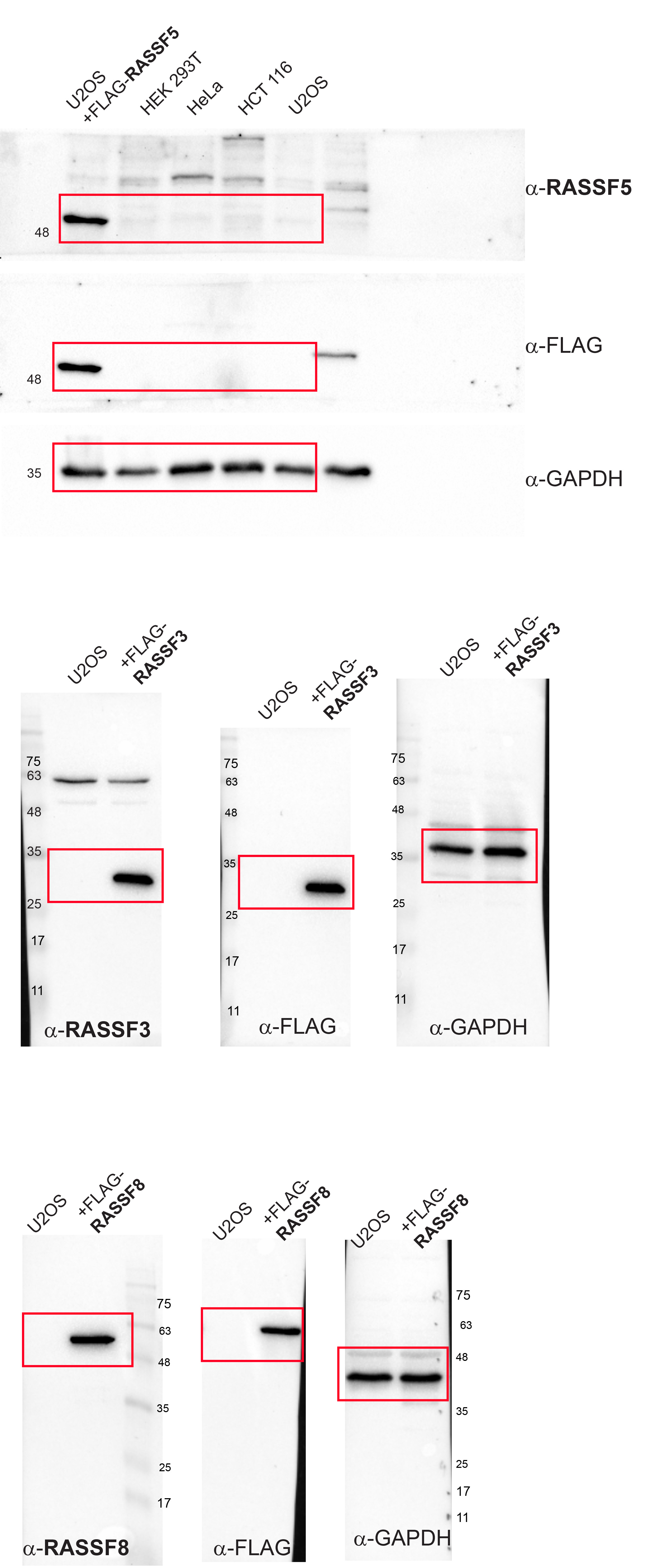

Supplement: Supplementary file 5 — Source data Fig. 3 [file 44319_2024_203_MOESM5_ESM.zip › 3A/3A_Blots.jpg]

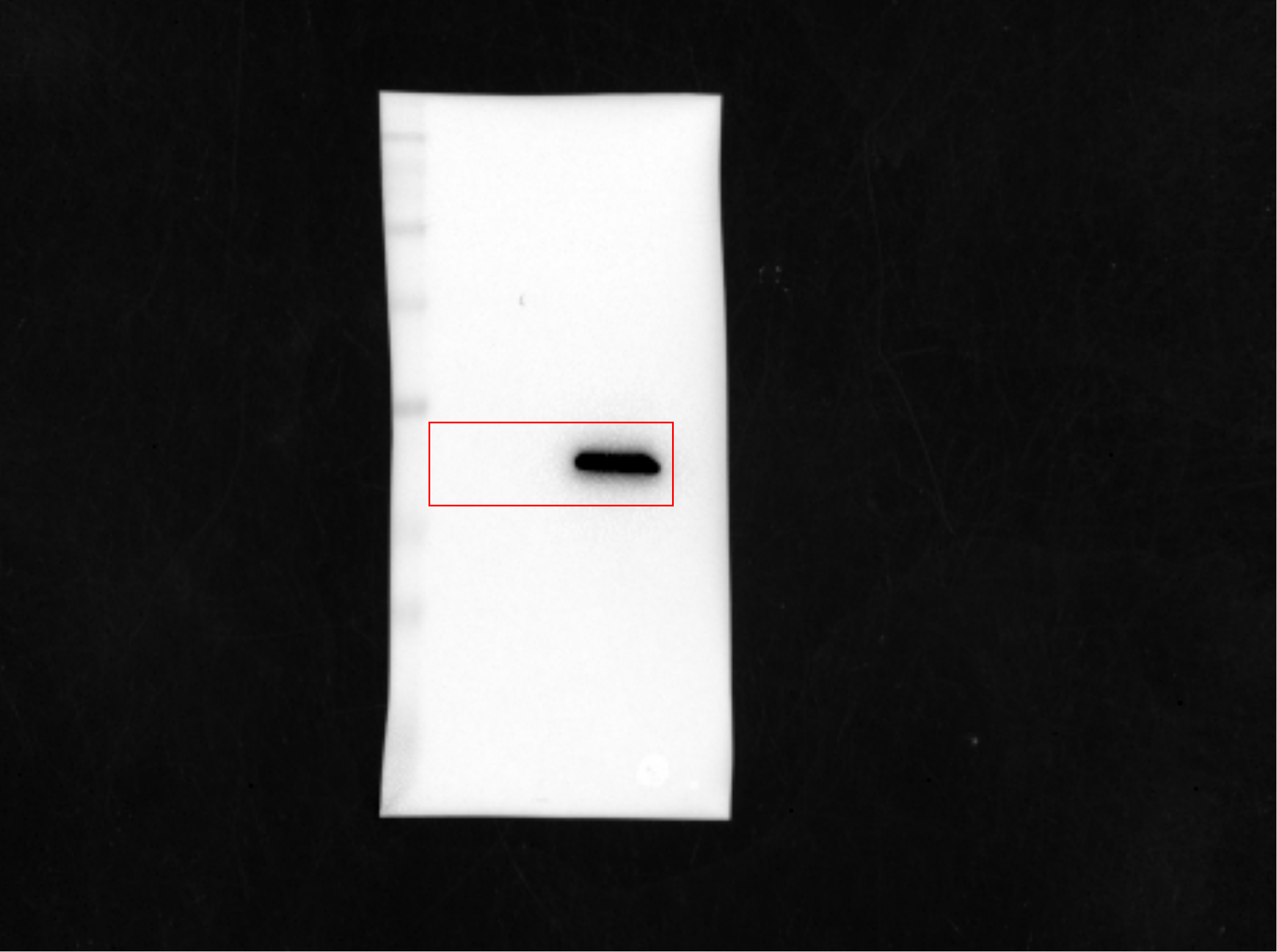

Supplement: Supplementary file 5 — Source data Fig. 3 [file 44319_2024_203_MOESM5_ESM.zip › 3A/RASSF3/Anti-FLAG.jpg]

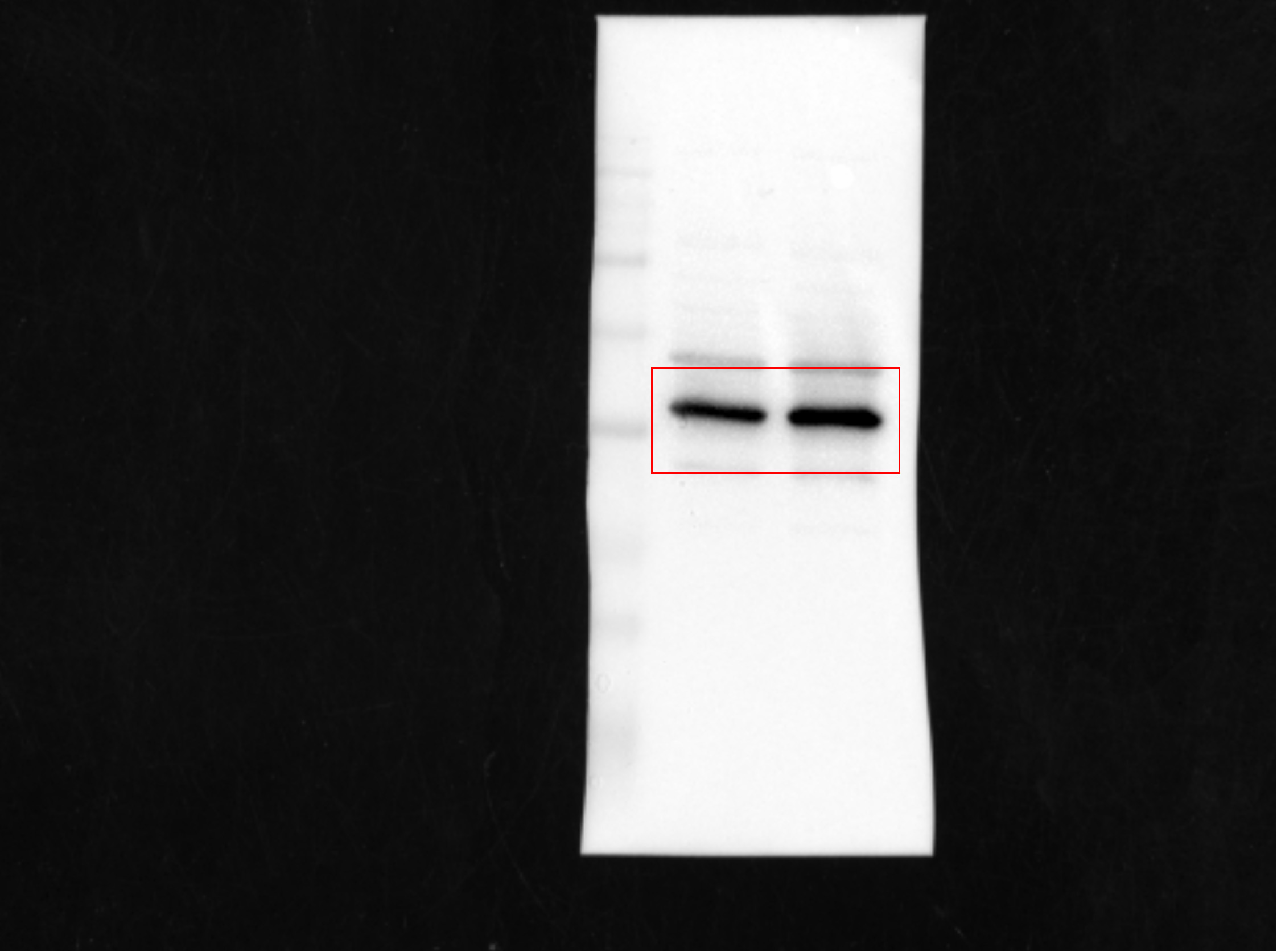

Supplement: Supplementary file 5 — Source data Fig. 3 [file 44319_2024_203_MOESM5_ESM.zip › 3A/RASSF3/Anti-GAPDH.jpg]

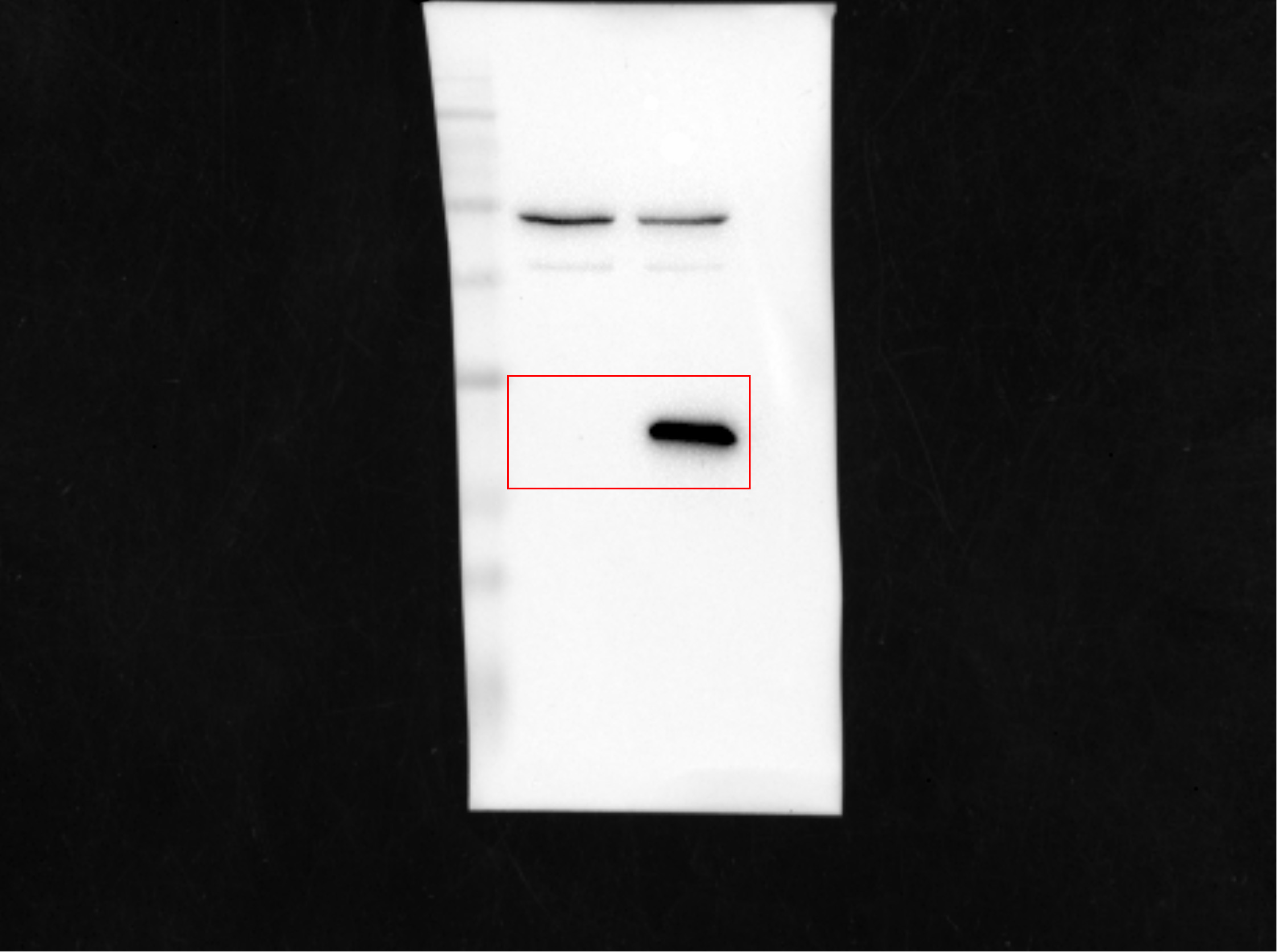

Supplement: Supplementary file 5 — Source data Fig. 3 [file 44319_2024_203_MOESM5_ESM.zip › 3A/RASSF3/Anti-RASSF3.jpg]

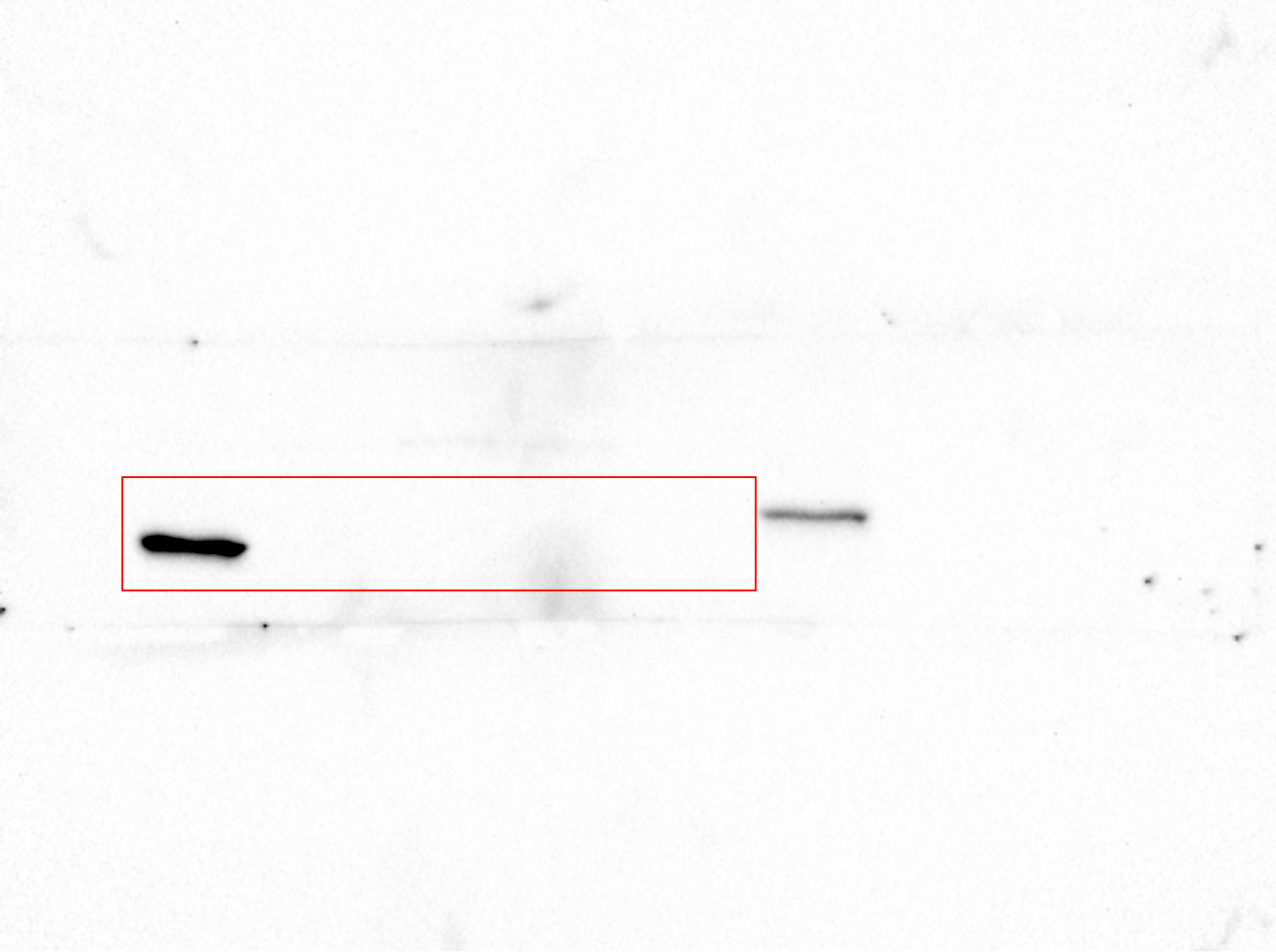

Supplement: Supplementary file 5 — Source data Fig. 3 [file 44319_2024_203_MOESM5_ESM.zip › 3A/RASSF5/Anti-FLAG blot.jpg]

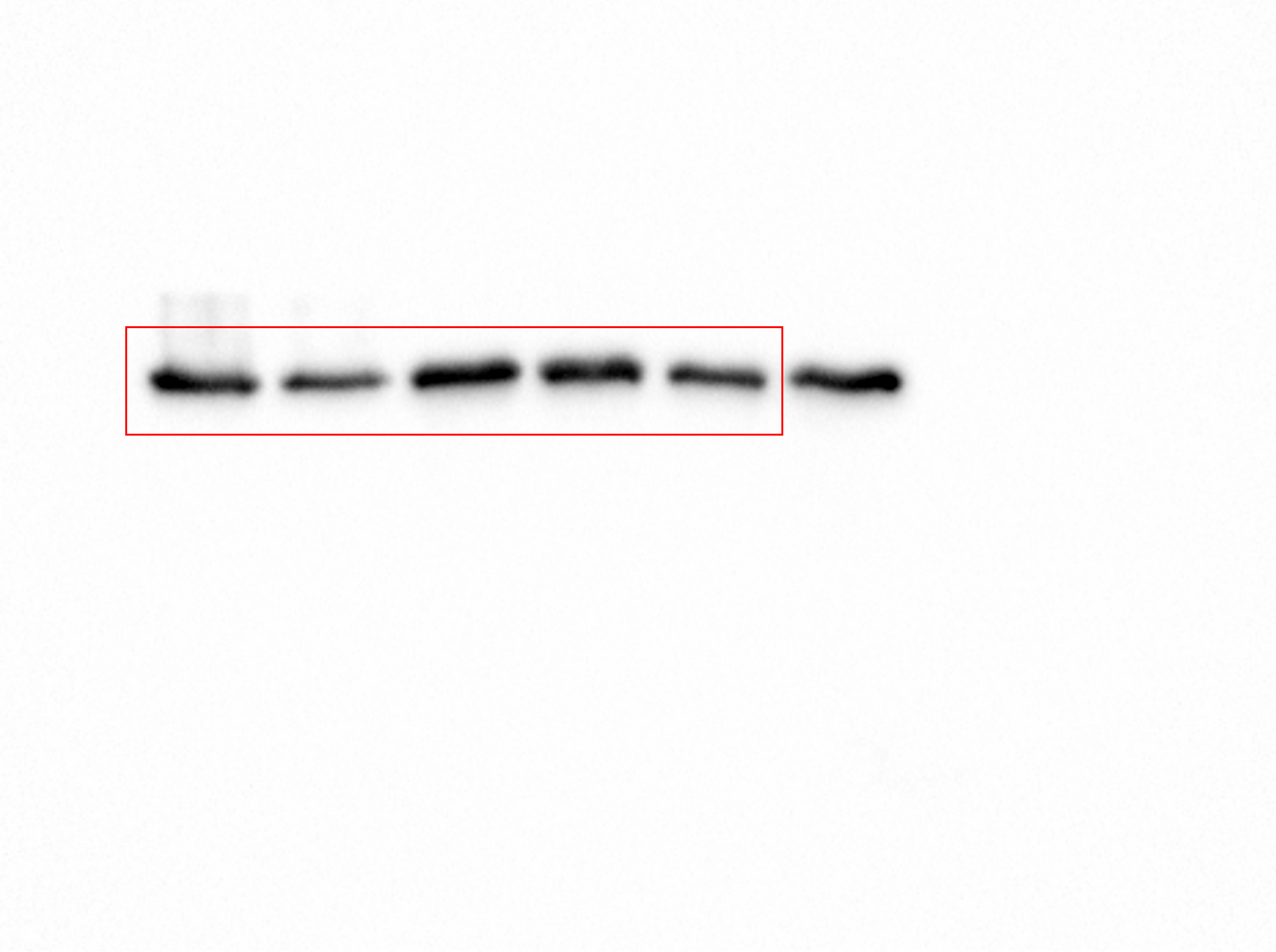

Supplement: Supplementary file 5 — Source data Fig. 3 [file 44319_2024_203_MOESM5_ESM.zip › 3A/RASSF5/Anti-GAPDH.jpg]

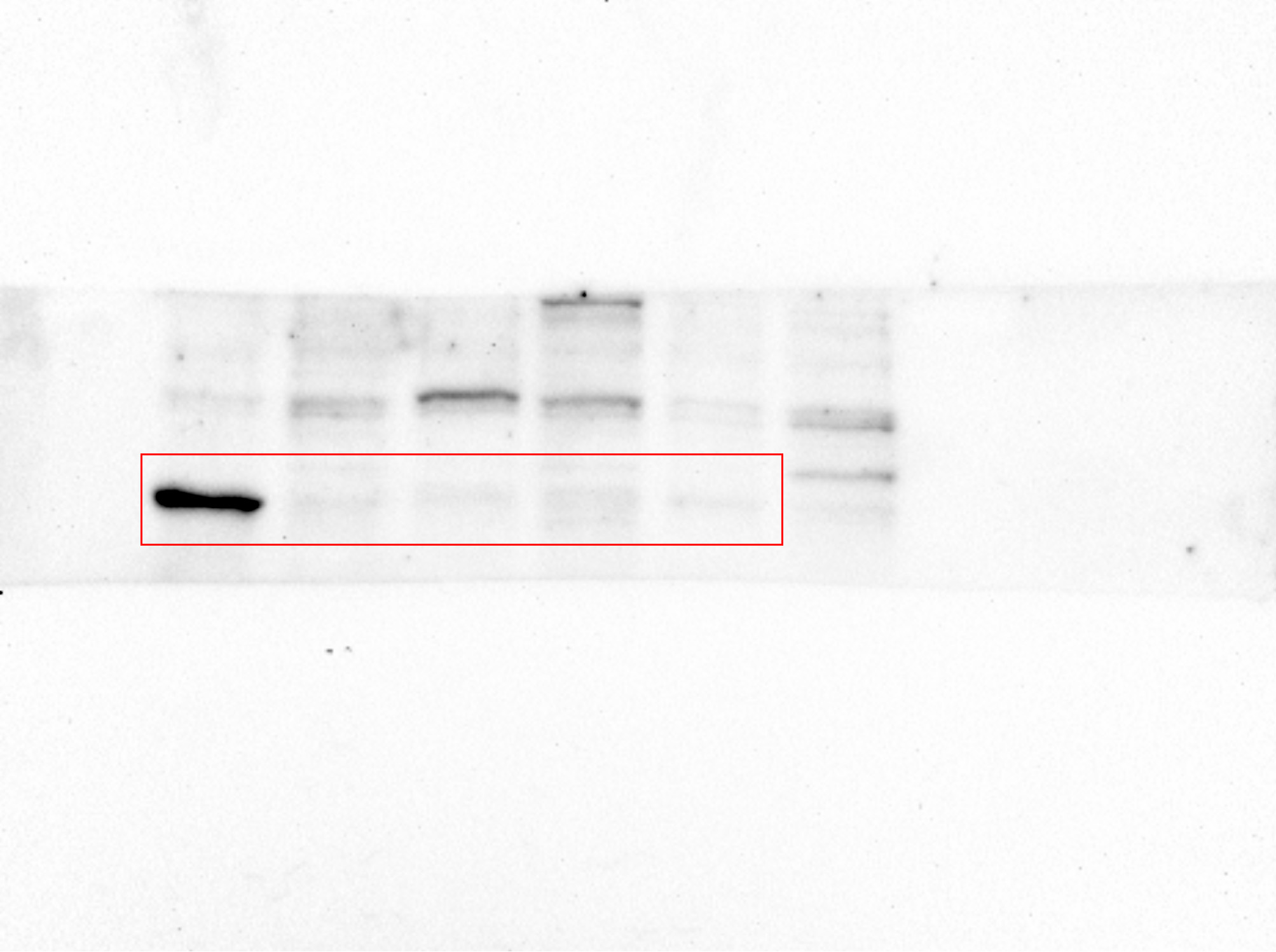

Supplement: Supplementary file 5 — Source data Fig. 3 [file 44319_2024_203_MOESM5_ESM.zip › 3A/RASSF5/Anti-RASSF5.jpg]

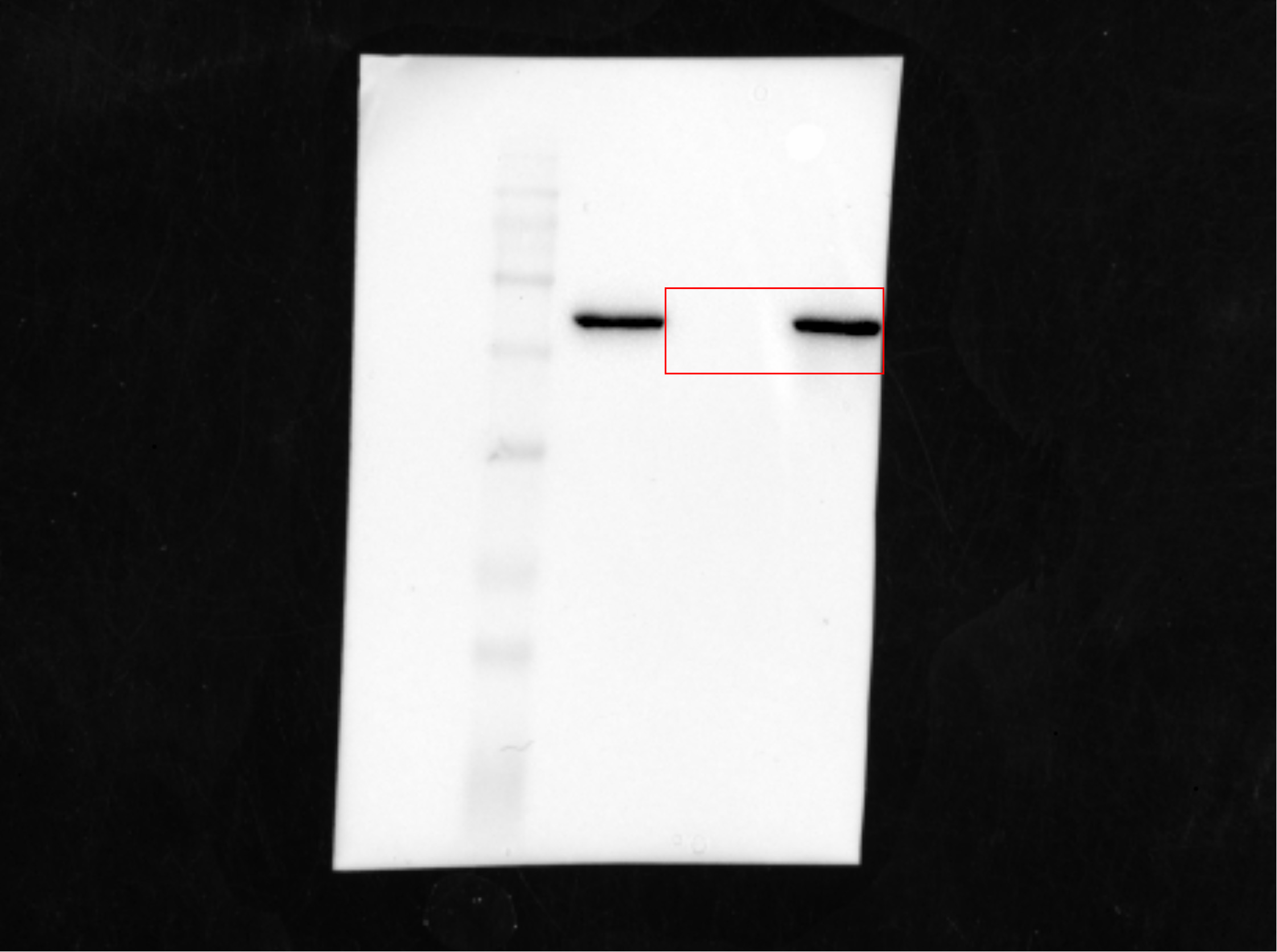

Supplement: Supplementary file 5 — Source data Fig. 3 [file 44319_2024_203_MOESM5_ESM.zip › 3A/RASSF8/Anti-FLAG.jpg]

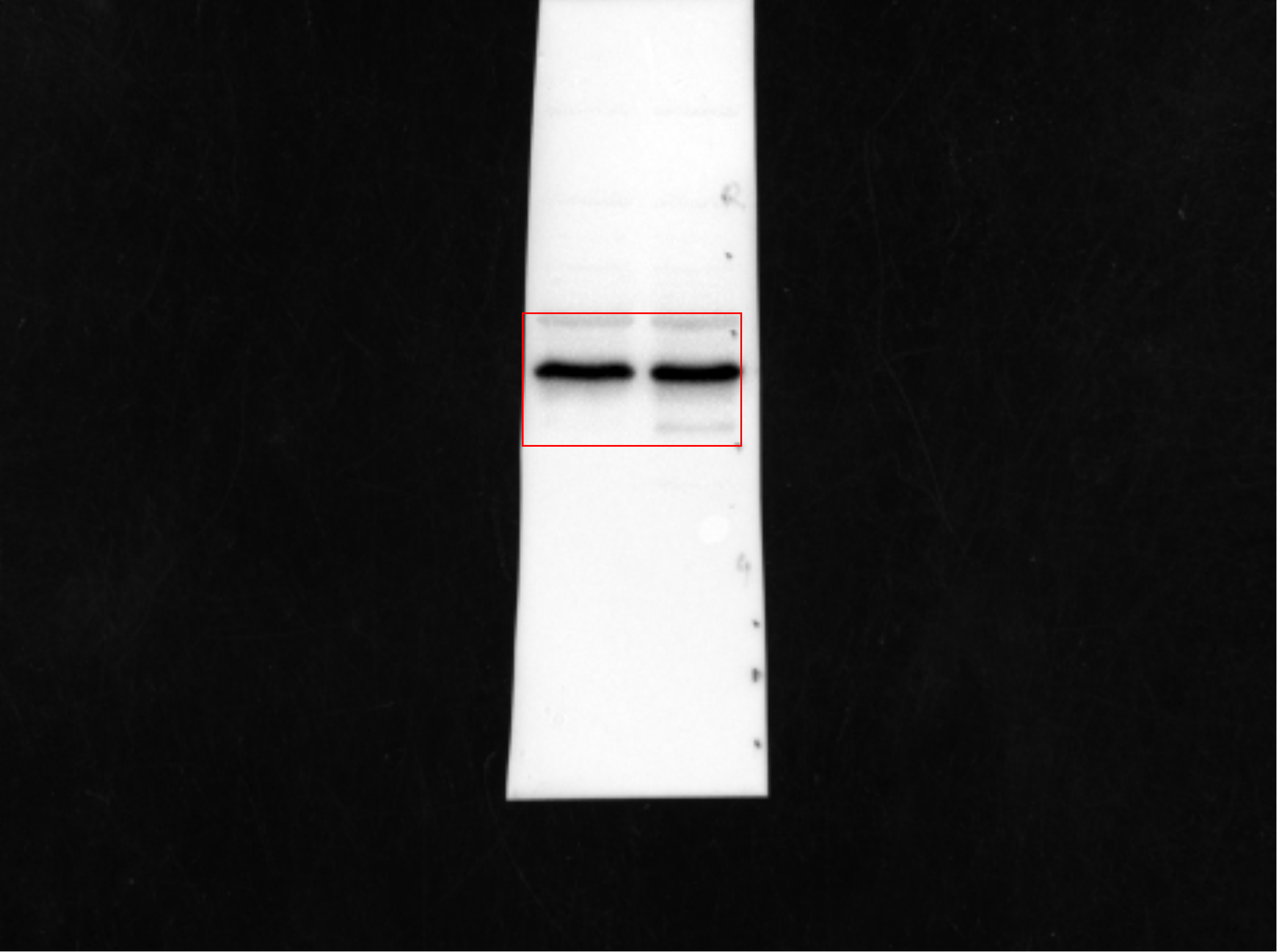

Supplement: Supplementary file 5 — Source data Fig. 3 [file 44319_2024_203_MOESM5_ESM.zip › 3A/RASSF8/Anti-GAPDH.jpg]

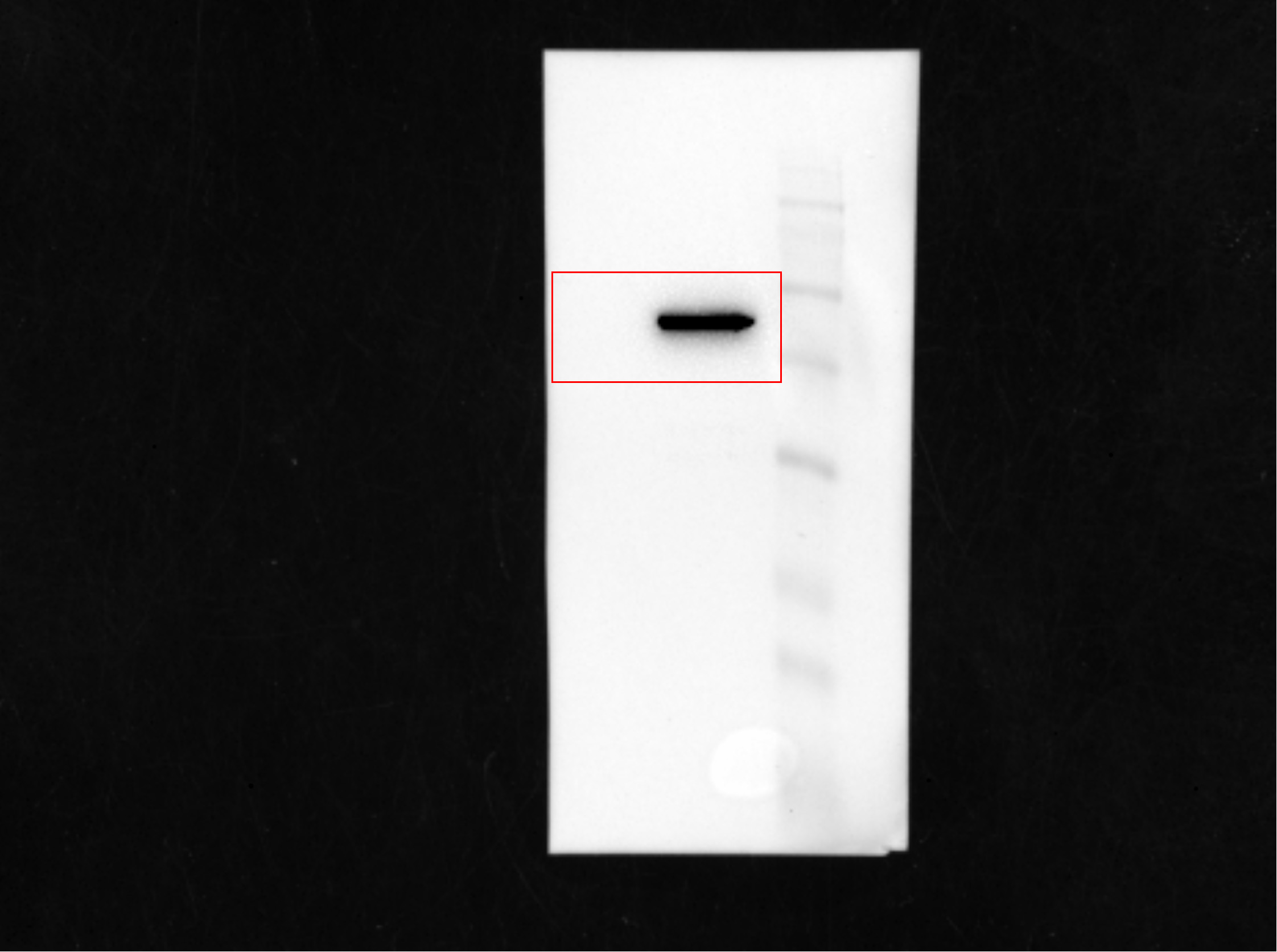

Supplement: Supplementary file 5 — Source data Fig. 3 [file 44319_2024_203_MOESM5_ESM.zip › 3A/RASSF8/Anti-RASSF8.jpg]

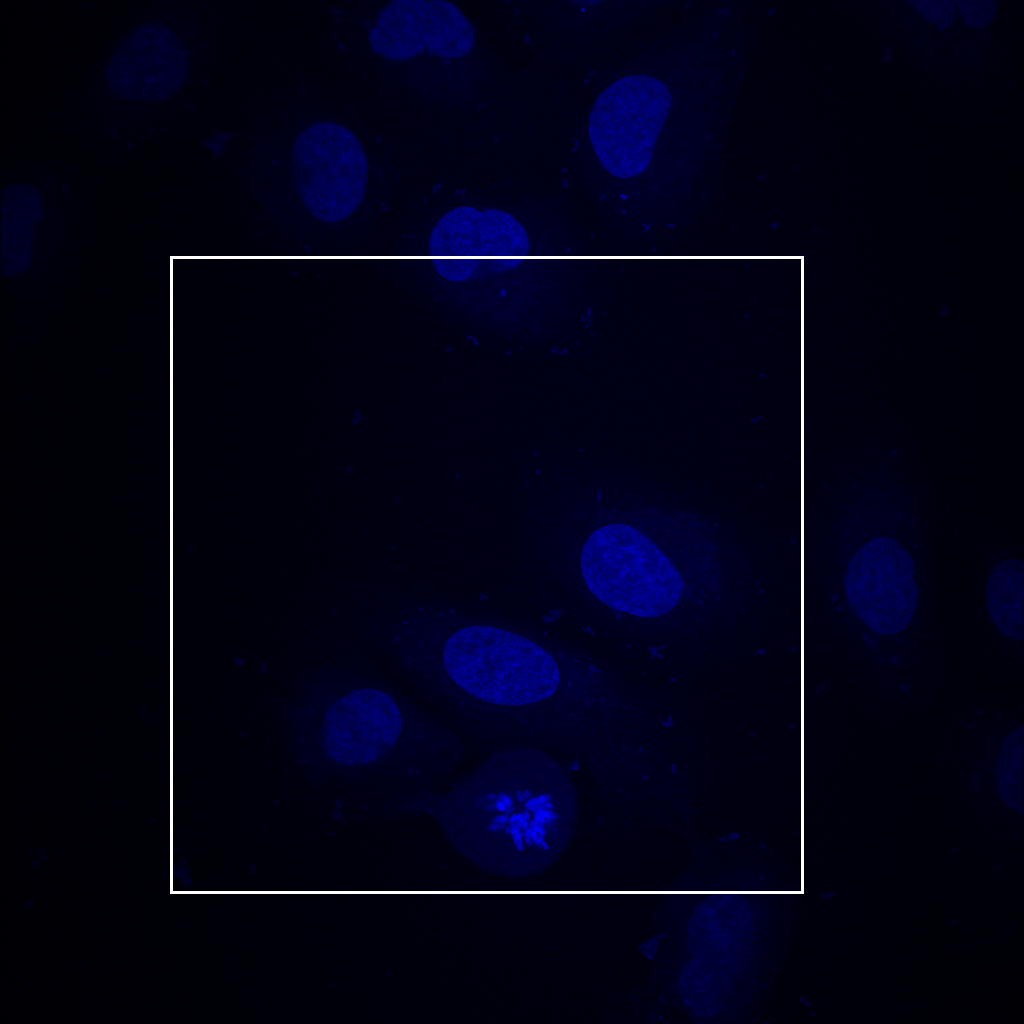

Supplement: Supplementary file 5 — Source data Fig. 3 [file 44319_2024_203_MOESM5_ESM.zip › 3D/Hoechst.tif]

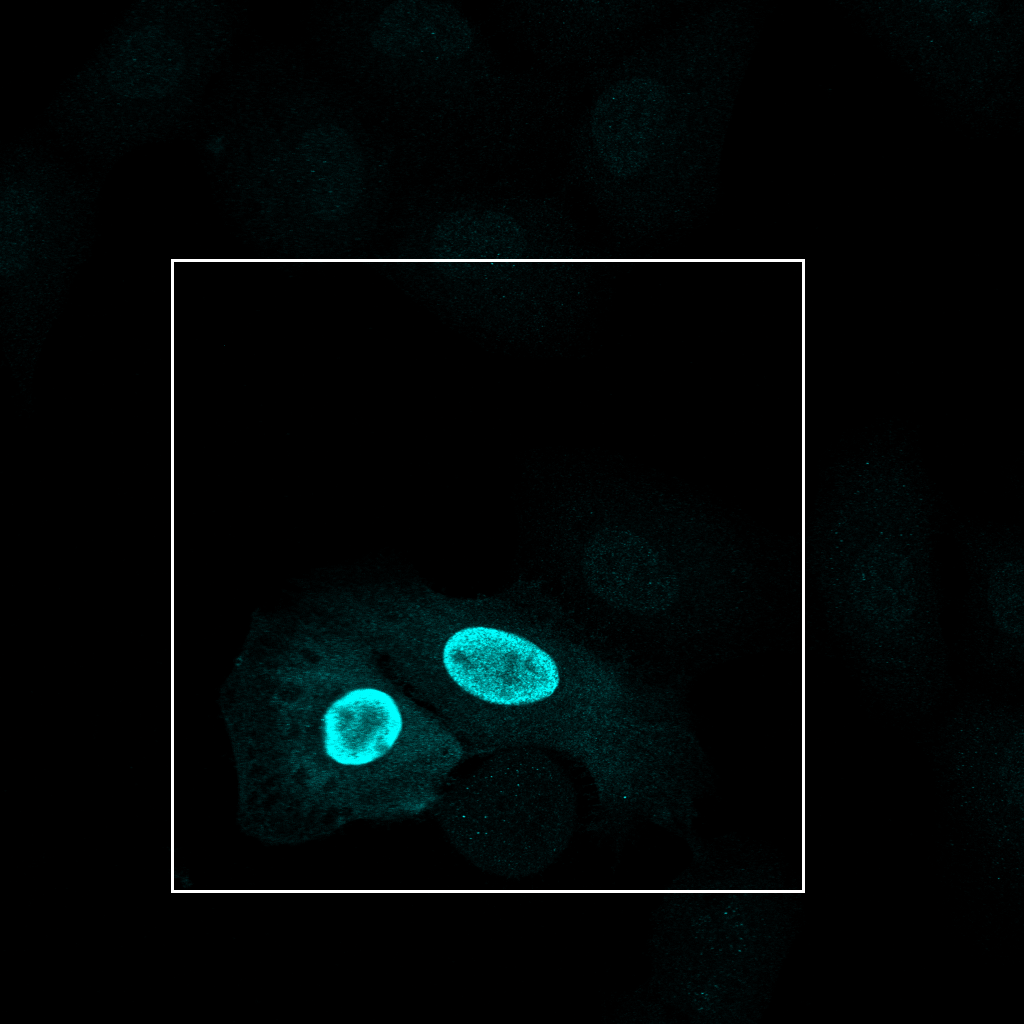

Supplement: Supplementary file 5 — Source data Fig. 3 [file 44319_2024_203_MOESM5_ESM.zip › 3D/RASSF5.tif]

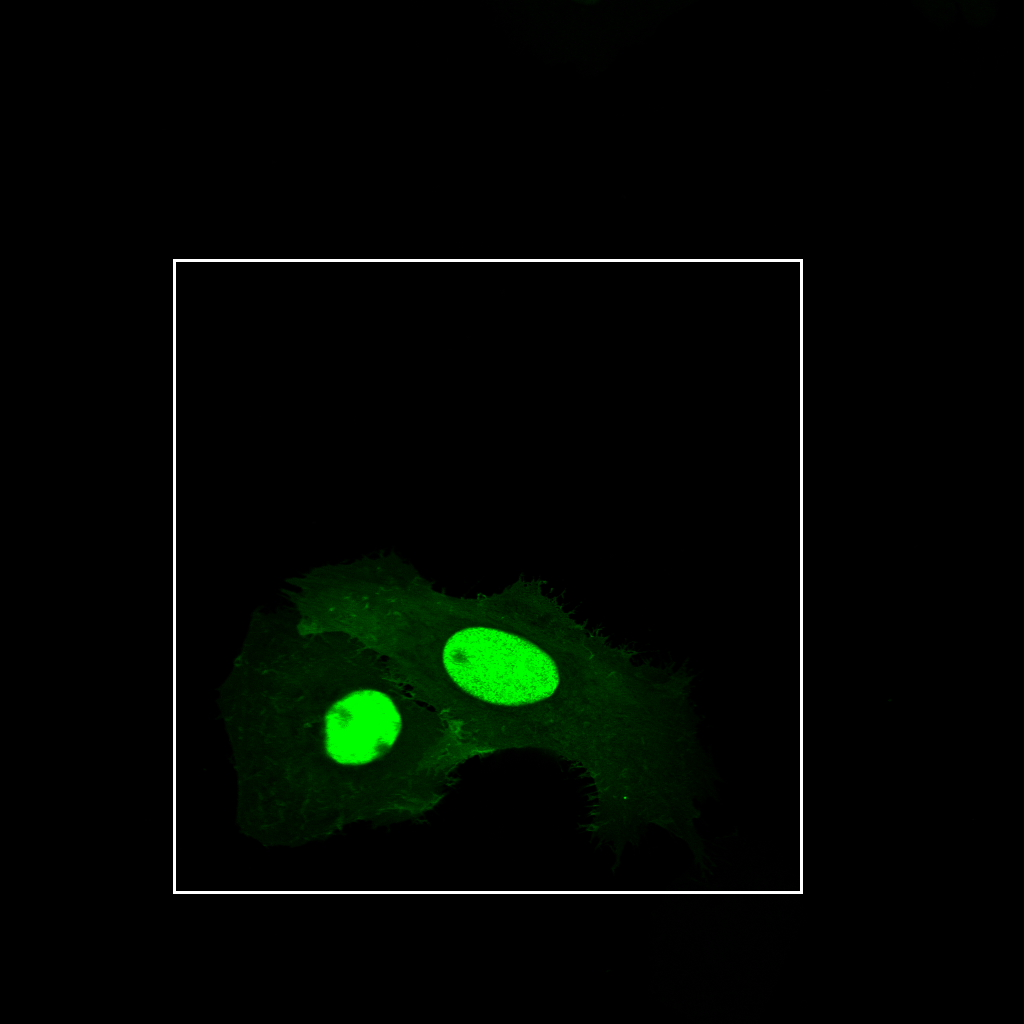

Supplement: Supplementary file 5 — Source data Fig. 3 [file 44319_2024_203_MOESM5_ESM.zip › 3D/RIT2.tif]

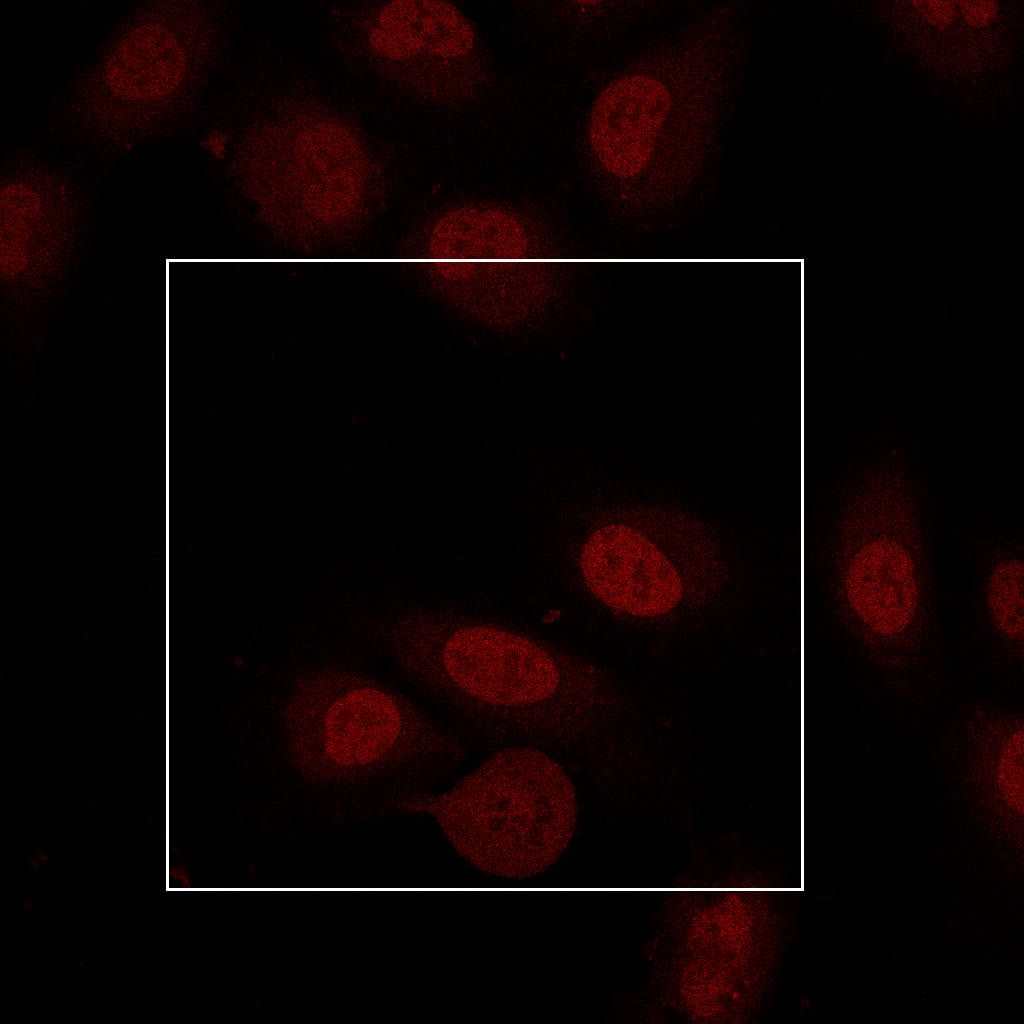

Supplement: Supplementary file 5 — Source data Fig. 3 [file 44319_2024_203_MOESM5_ESM.zip › 3D/YAP.tif]

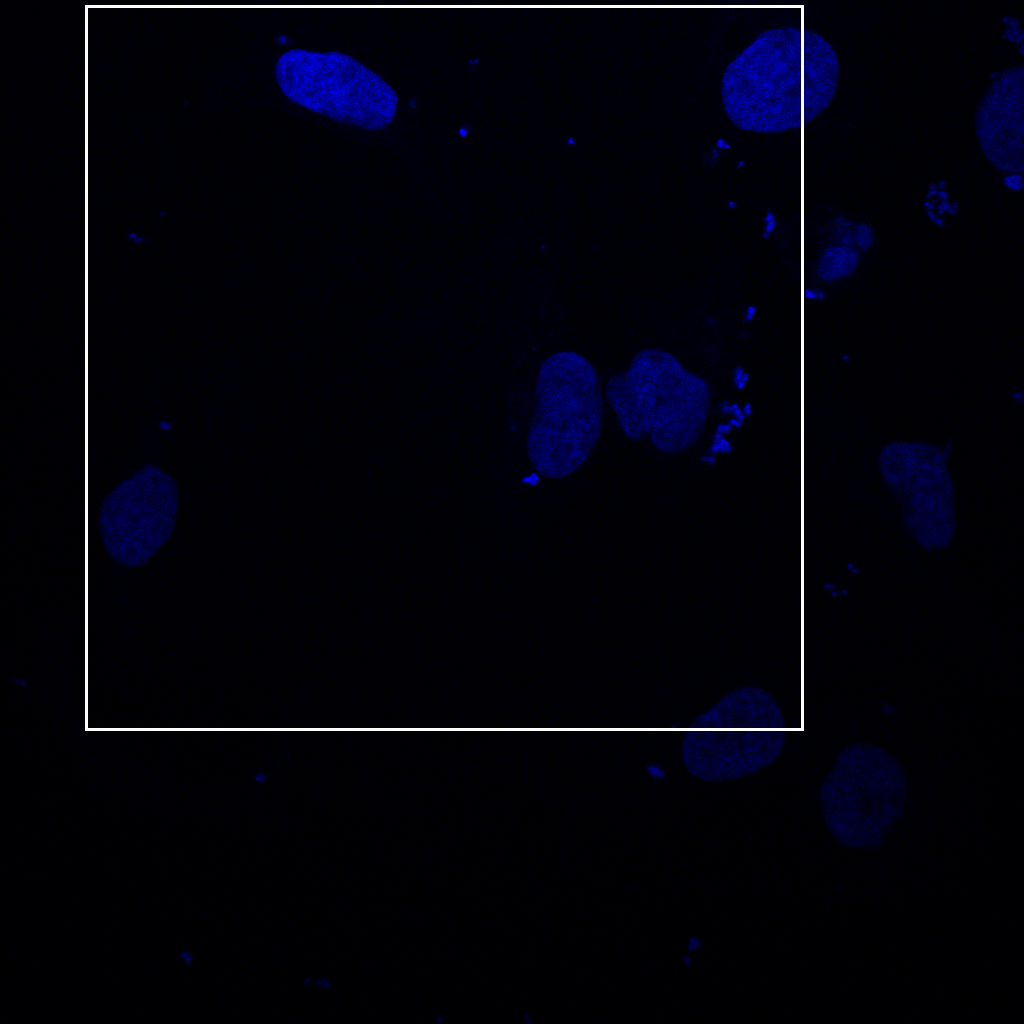

Supplement: Supplementary file 5 — Source data Fig. 3 [file 44319_2024_203_MOESM5_ESM.zip › 3E/Hoechst.tif]

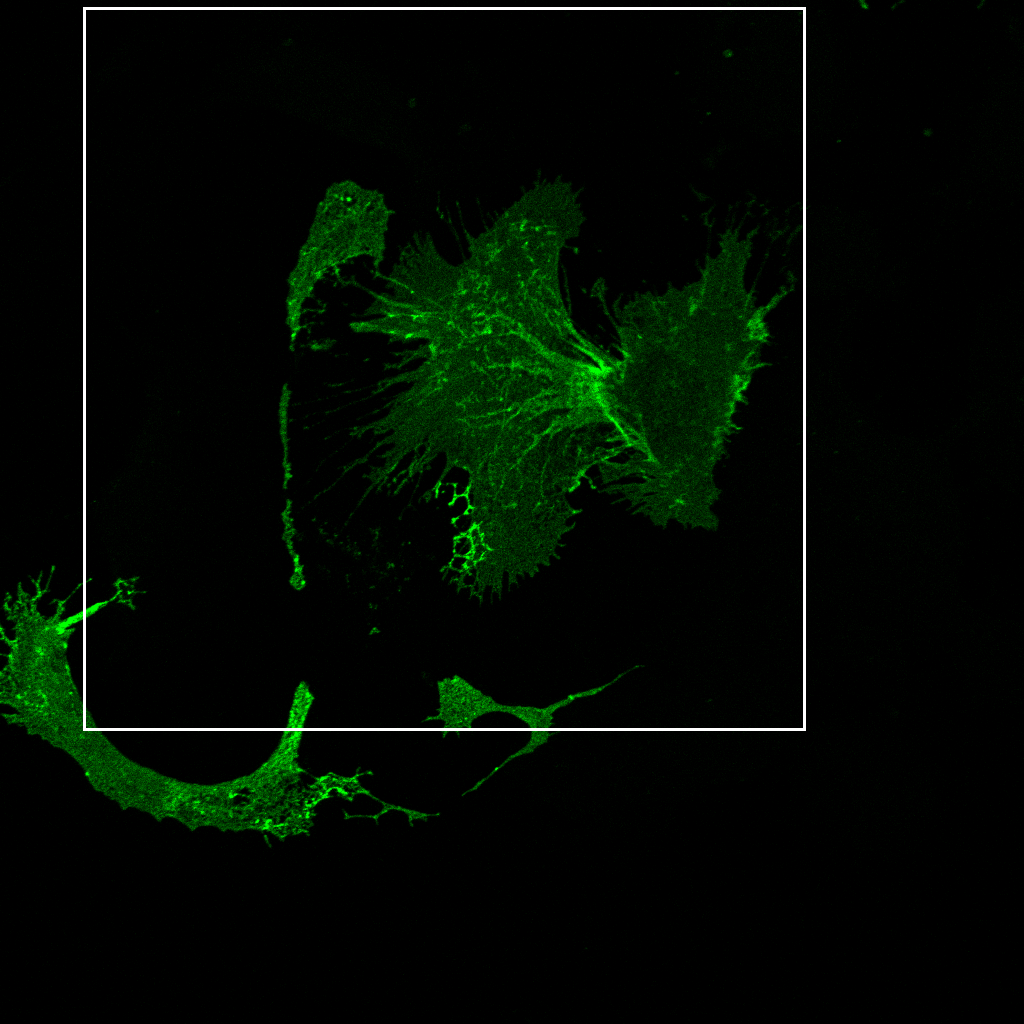

Supplement: Supplementary file 5 — Source data Fig. 3 [file 44319_2024_203_MOESM5_ESM.zip › 3E/KRAS.tif]

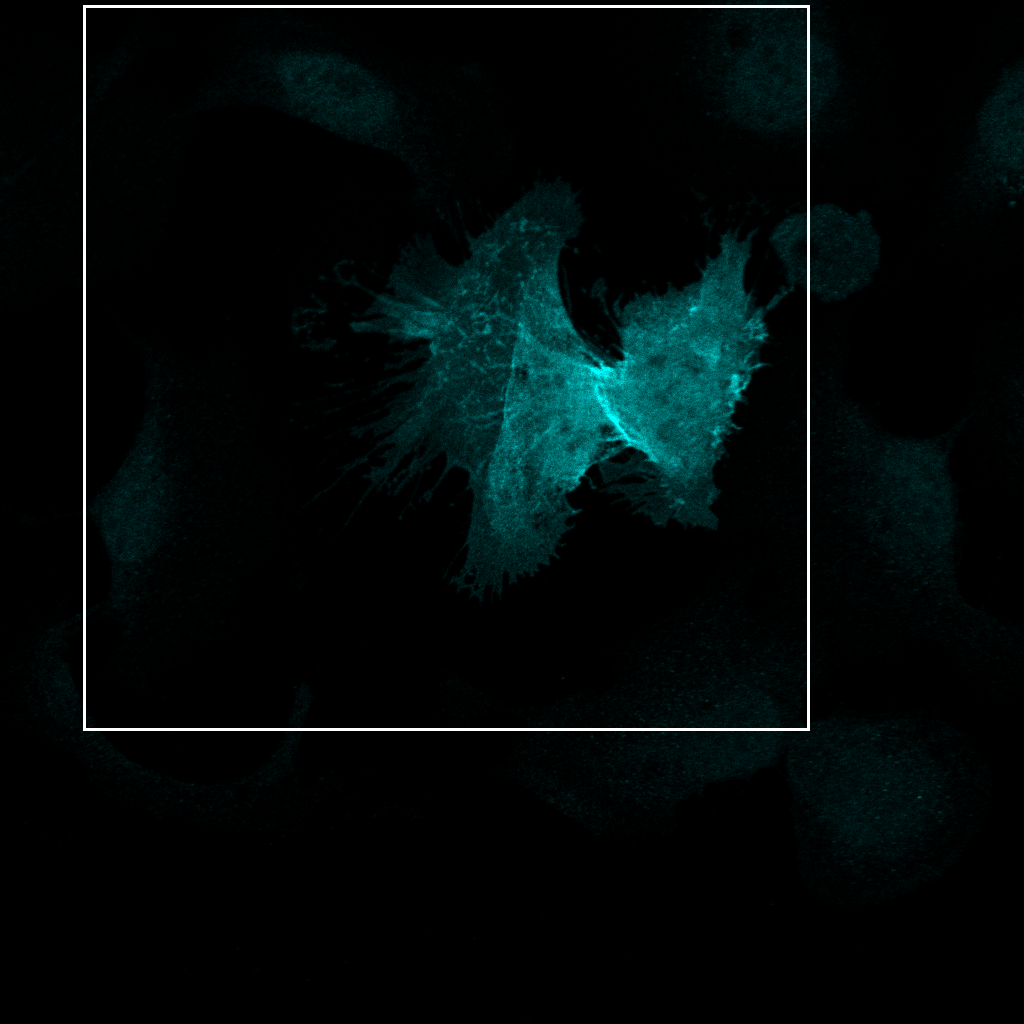

Supplement: Supplementary file 5 — Source data Fig. 3 [file 44319_2024_203_MOESM5_ESM.zip › 3E/RASSF5-delSARAH.tif]

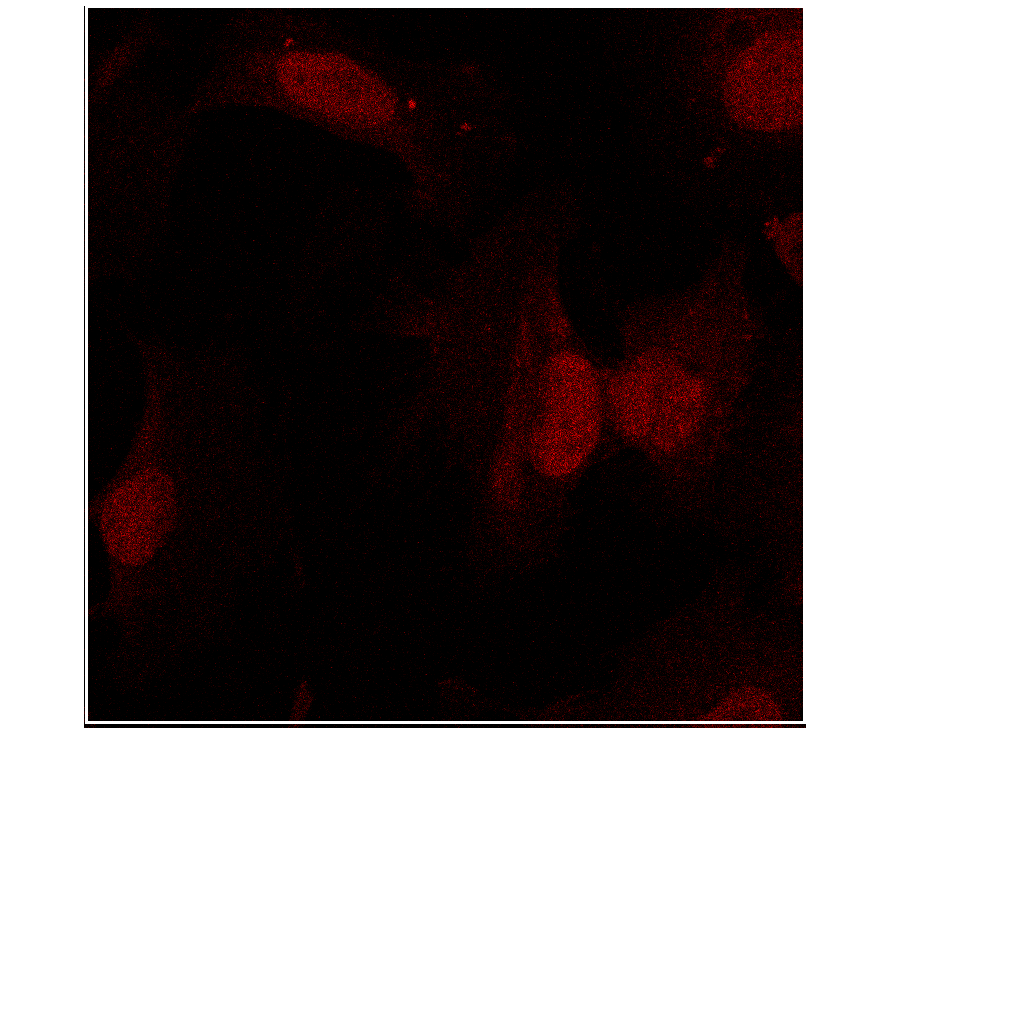

Supplement: Supplementary file 5 — Source data Fig. 3 [file 44319_2024_203_MOESM5_ESM.zip › 3E/YAP.tif]

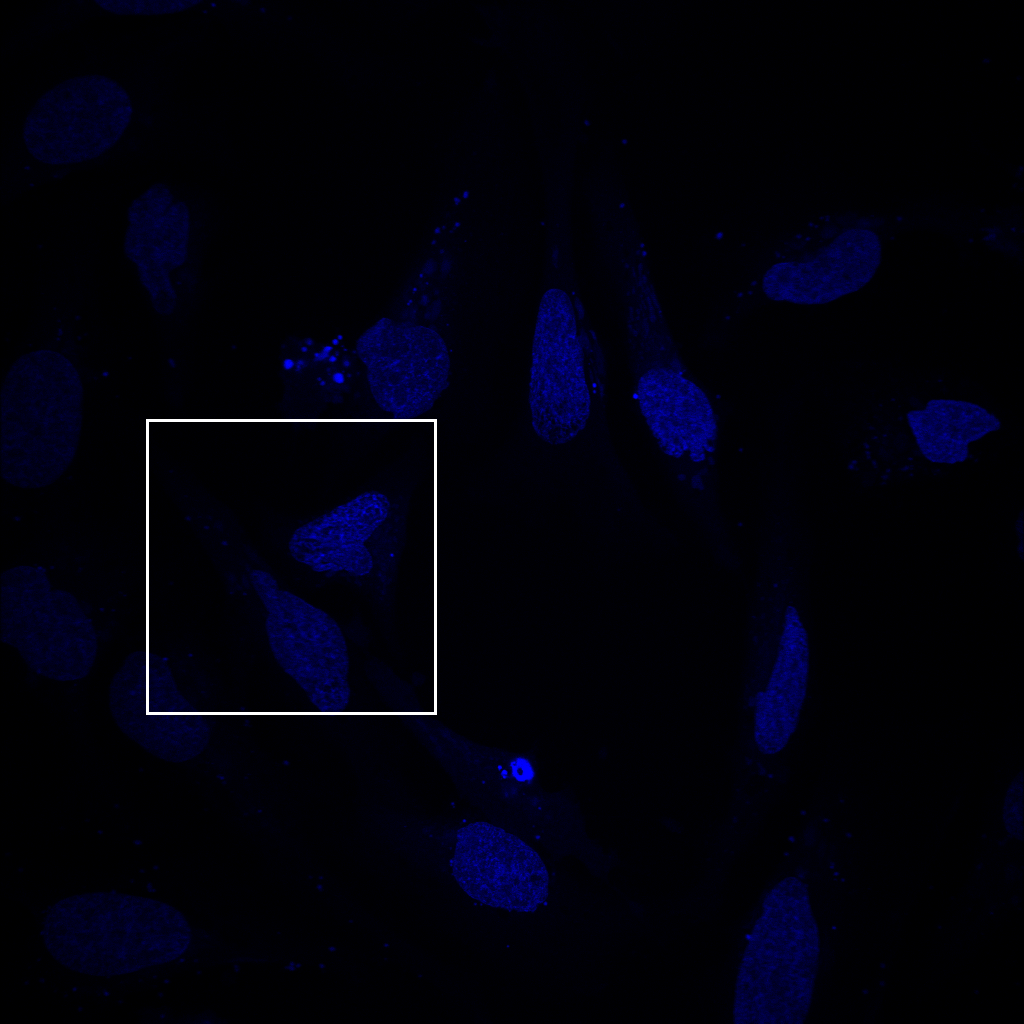

Supplement: Supplementary file 6 — Source data Fig. 4 [file 44319_2024_203_MOESM6_ESM.zip › 4A/Autophagosomes/Hoechst.tif]

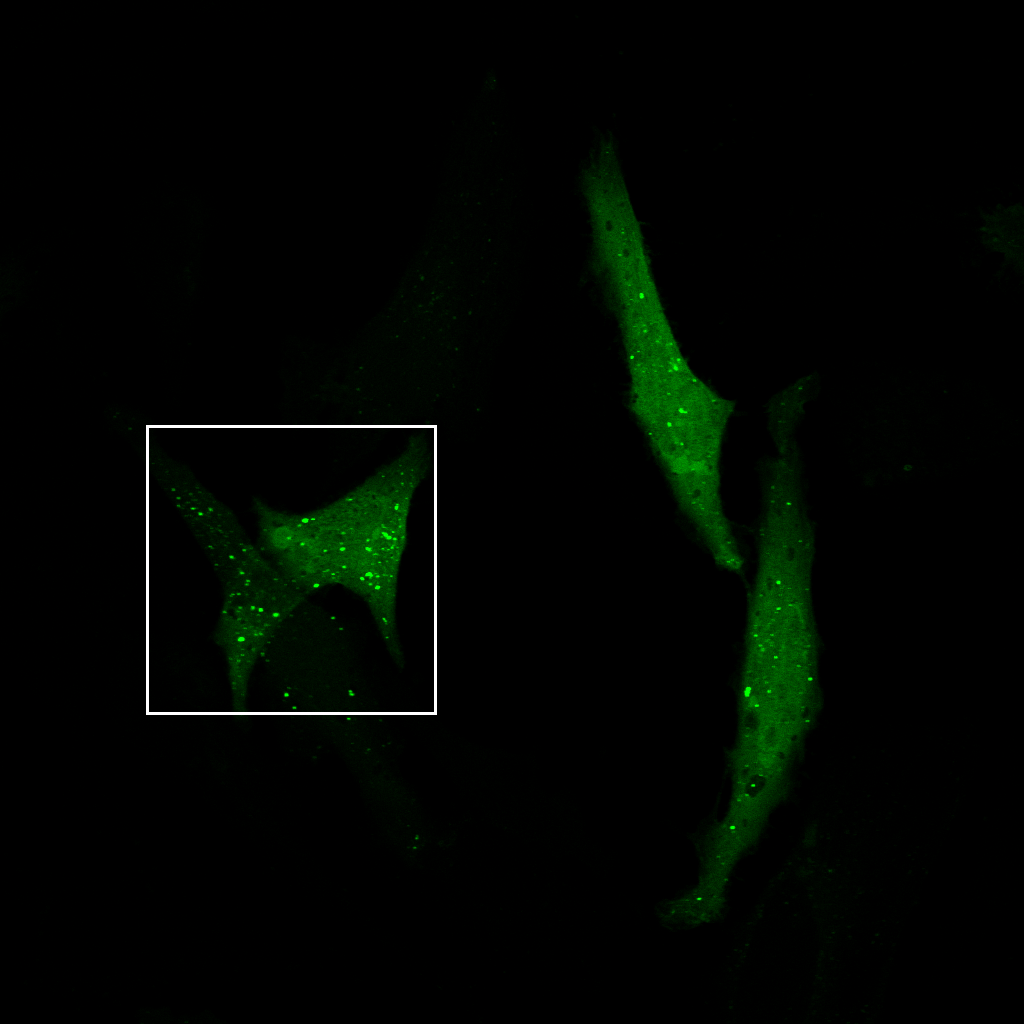

Supplement: Supplementary file 6 — Source data Fig. 4 [file 44319_2024_203_MOESM6_ESM.zip › 4A/Autophagosomes/LC3.tif]

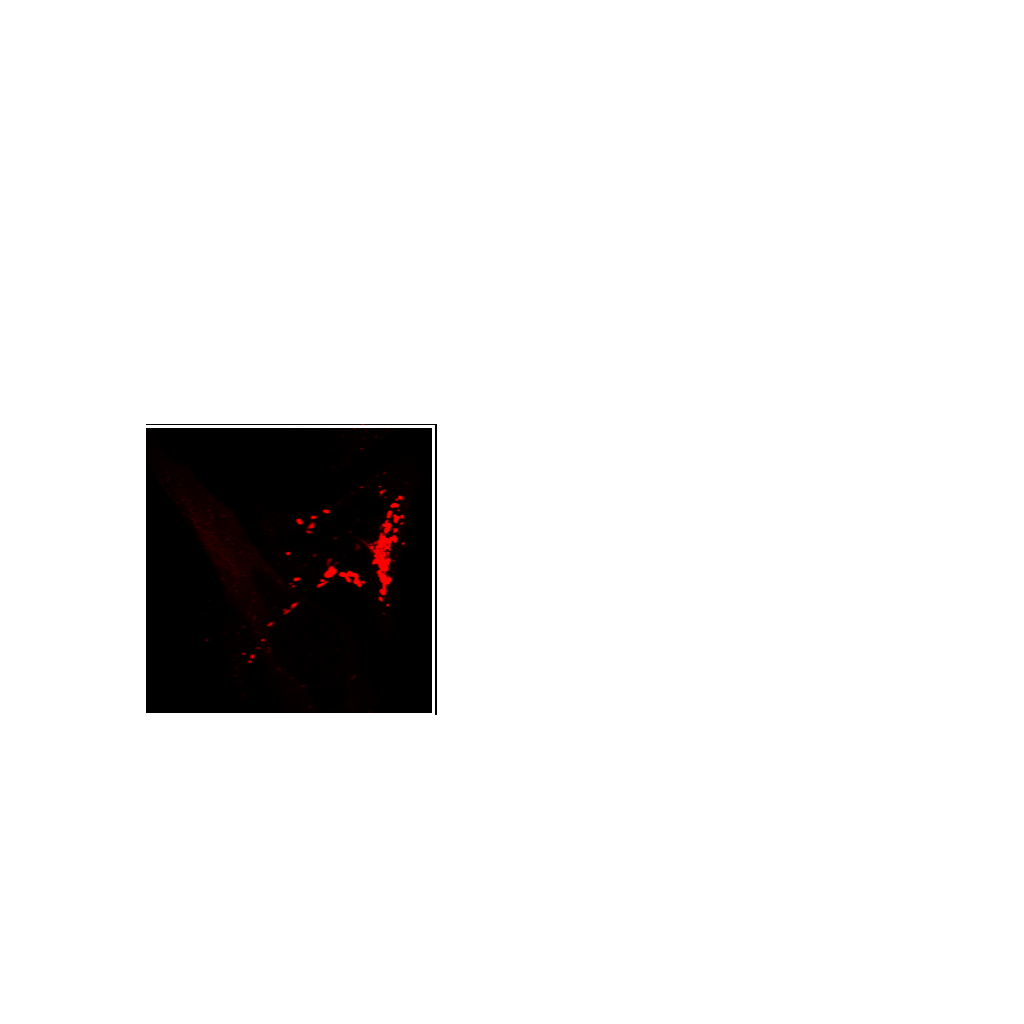

Supplement: Supplementary file 6 — Source data Fig. 4 [file 44319_2024_203_MOESM6_ESM.zip › 4A/Autophagosomes/RASSF8.tif]

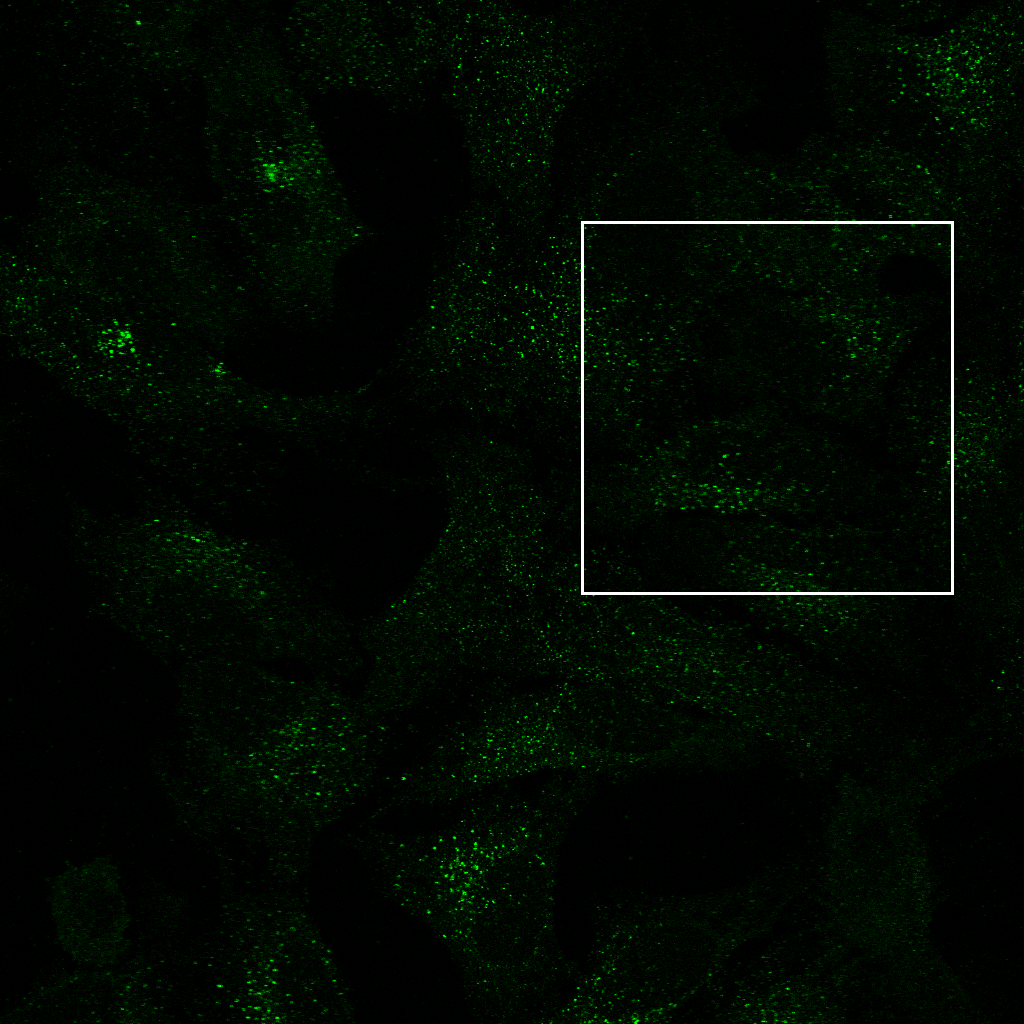

Supplement: Supplementary file 6 — Source data Fig. 4 [file 44319_2024_203_MOESM6_ESM.zip › 4A/Endosomes/EEA1.tif]

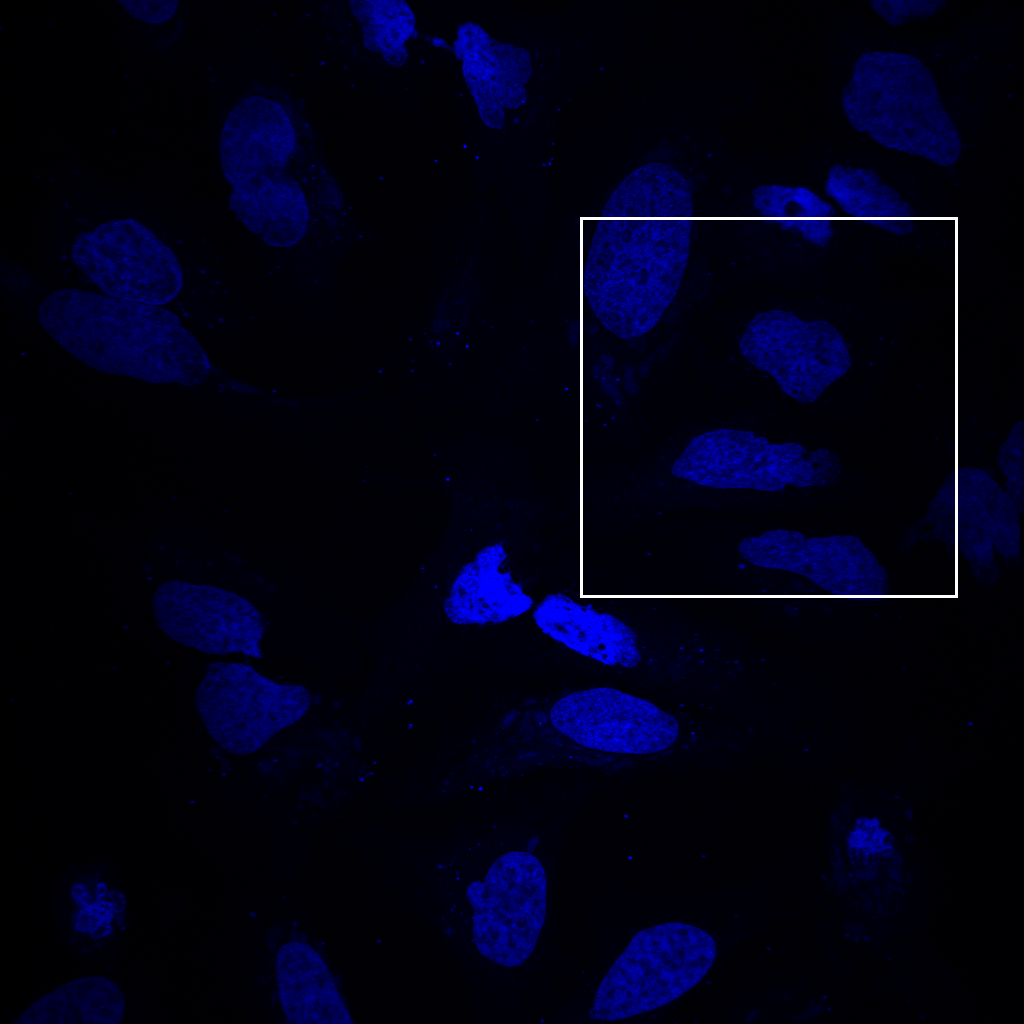

Supplement: Supplementary file 6 — Source data Fig. 4 [file 44319_2024_203_MOESM6_ESM.zip › 4A/Endosomes/Hoechst.tif]

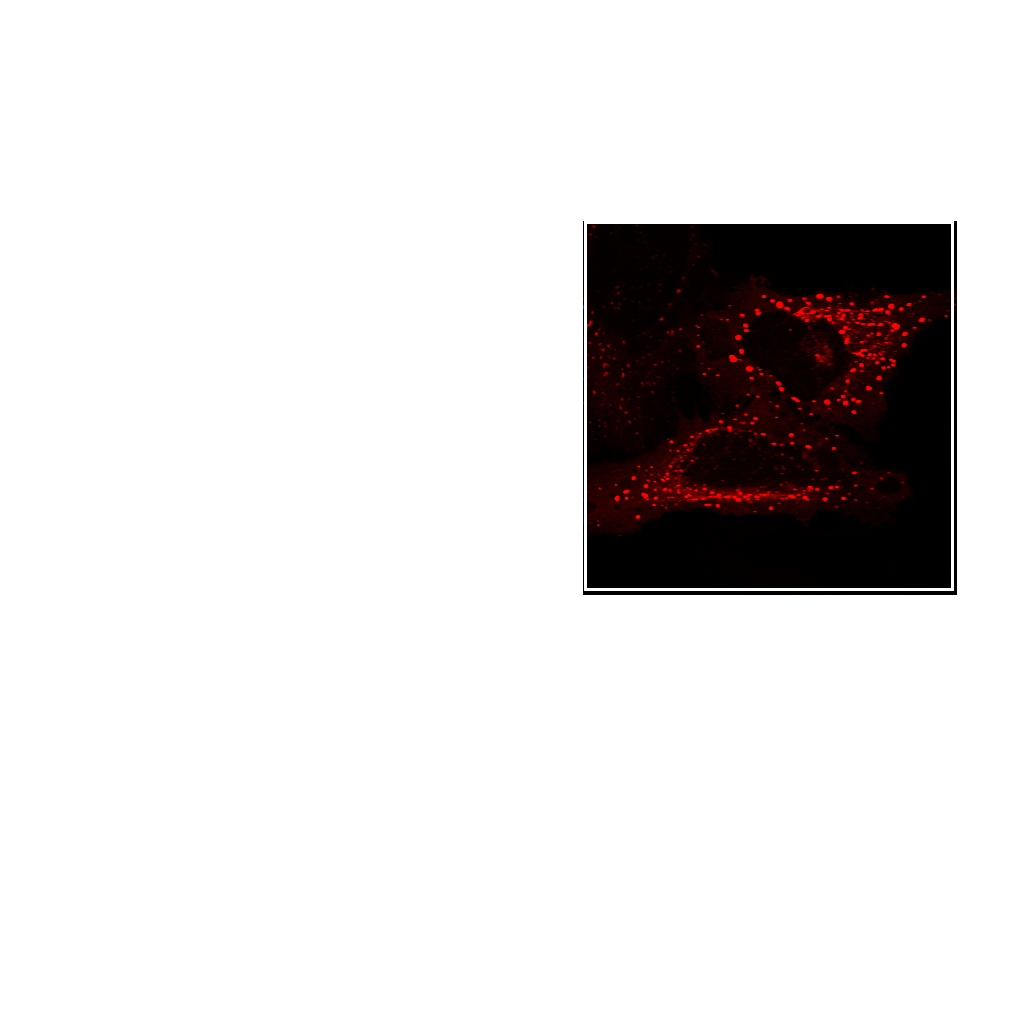

Supplement: Supplementary file 6 — Source data Fig. 4 [file 44319_2024_203_MOESM6_ESM.zip › 4A/Endosomes/RASSF8.tif]

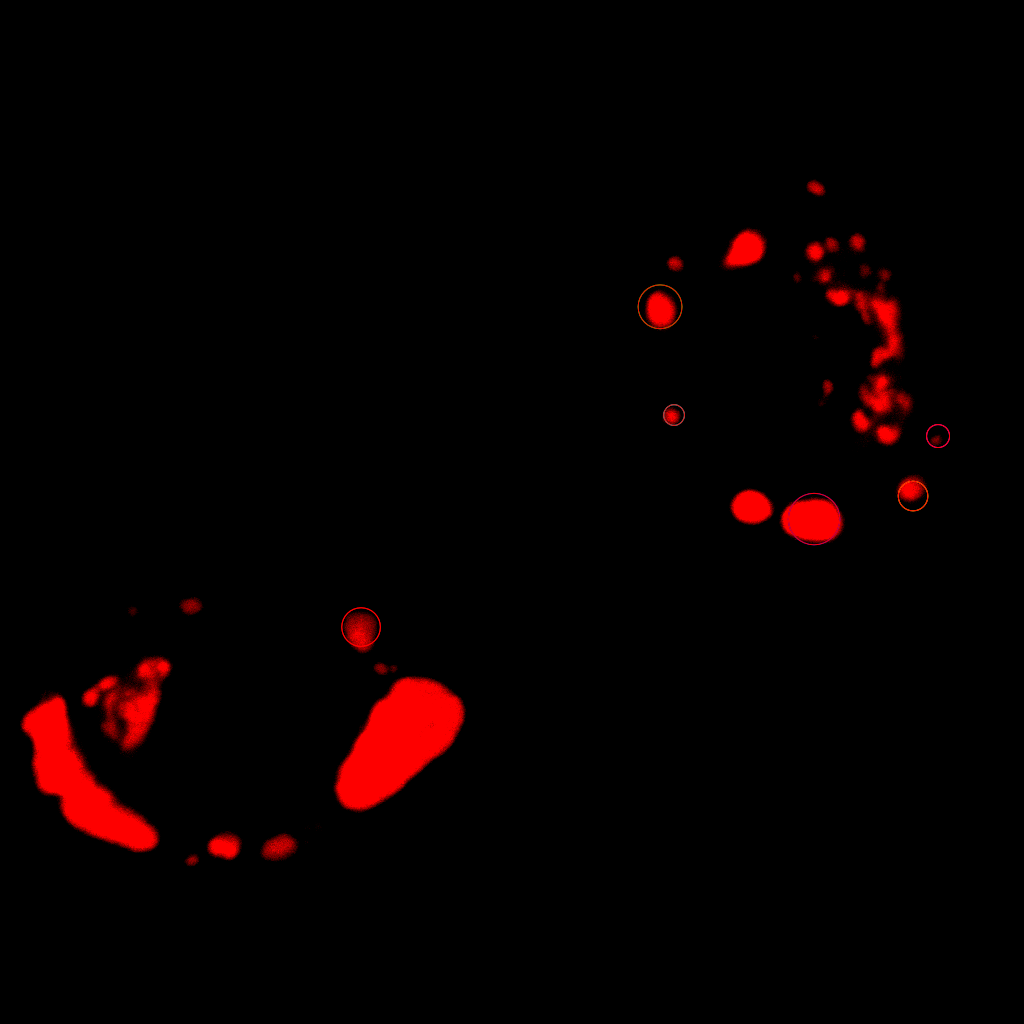

Supplement: Supplementary file 6 — Source data Fig. 4 [file 44319_2024_203_MOESM6_ESM.zip › 4B/-1min_FRAP4_MCH-RASSF8 1MIN TIME[OINT_t01c1.tif]

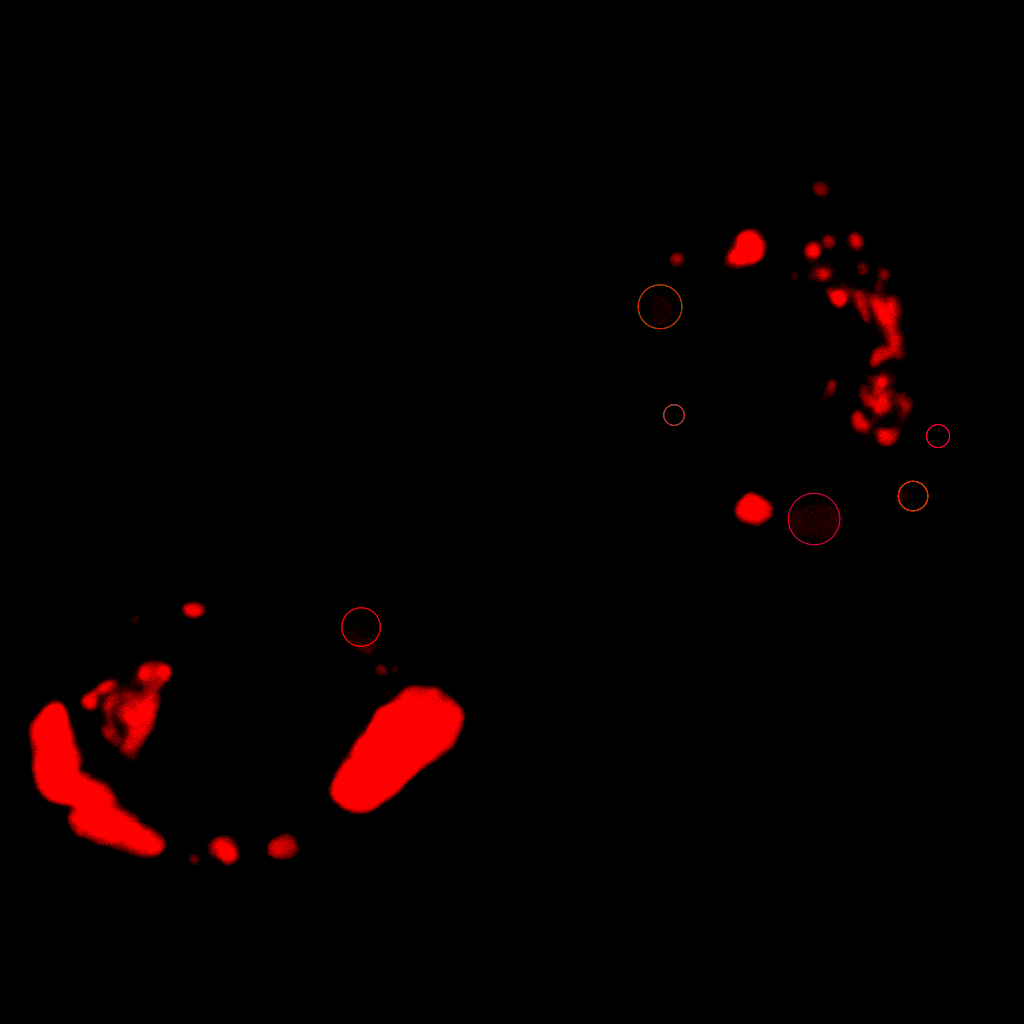

Supplement: Supplementary file 6 — Source data Fig. 4 [file 44319_2024_203_MOESM6_ESM.zip › 4B/0min_FRAP4_MCH-RASSF8 1MIN TIME[OINT_t02c1.tif]

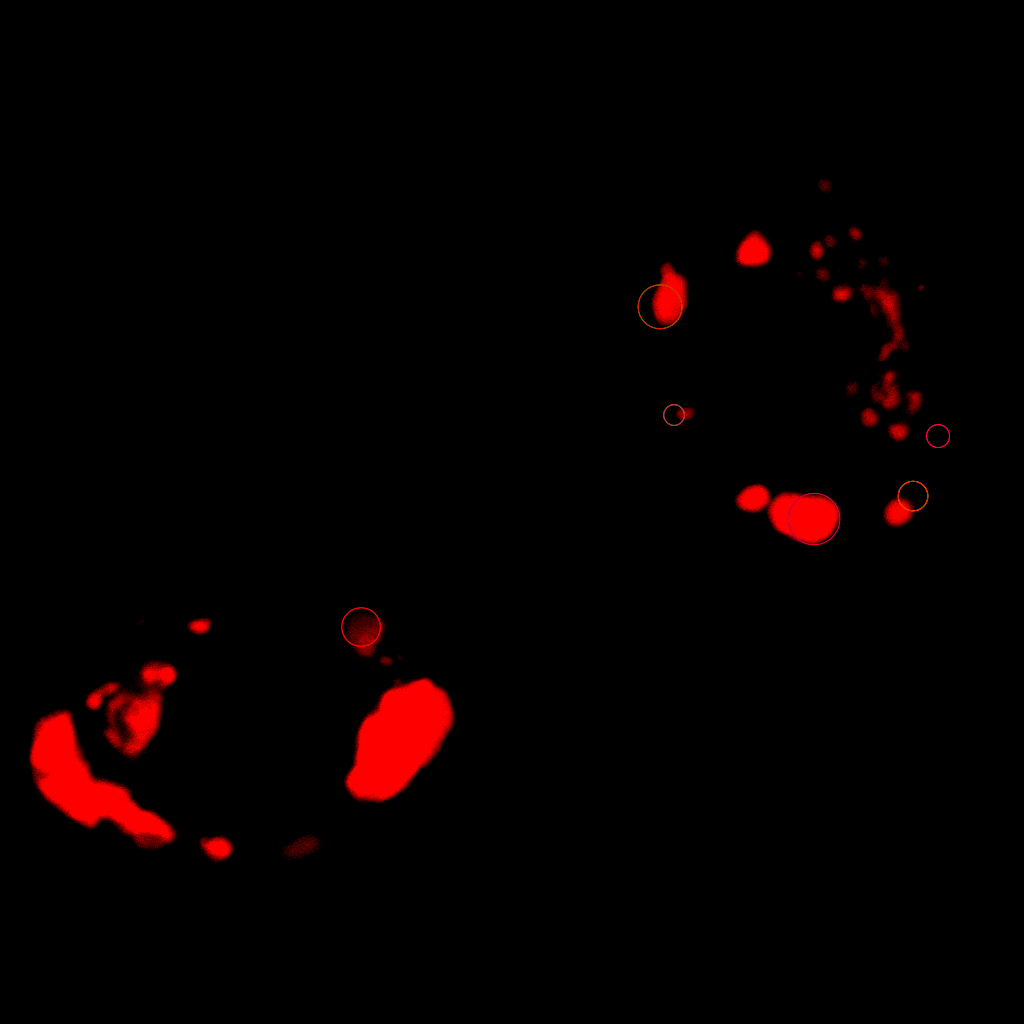

Supplement: Supplementary file 6 — Source data Fig. 4 [file 44319_2024_203_MOESM6_ESM.zip › 4B/16min_FRAP4_MCH-RASSF8 1MIN TIME[OINT_t18c1.tif]

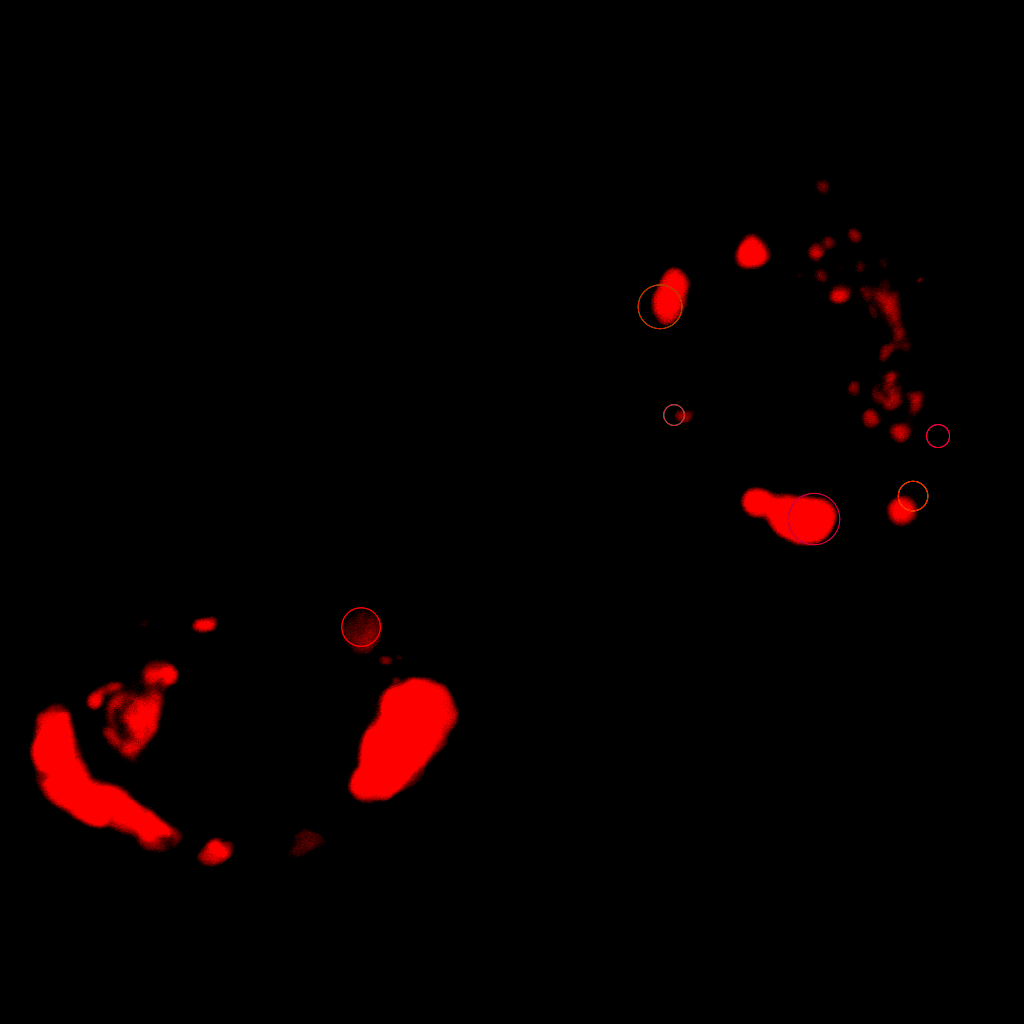

Supplement: Supplementary file 6 — Source data Fig. 4 [file 44319_2024_203_MOESM6_ESM.zip › 4B/17min_FRAP4_MCH-RASSF8 1MIN TIME[OINT_t19c1.tif]

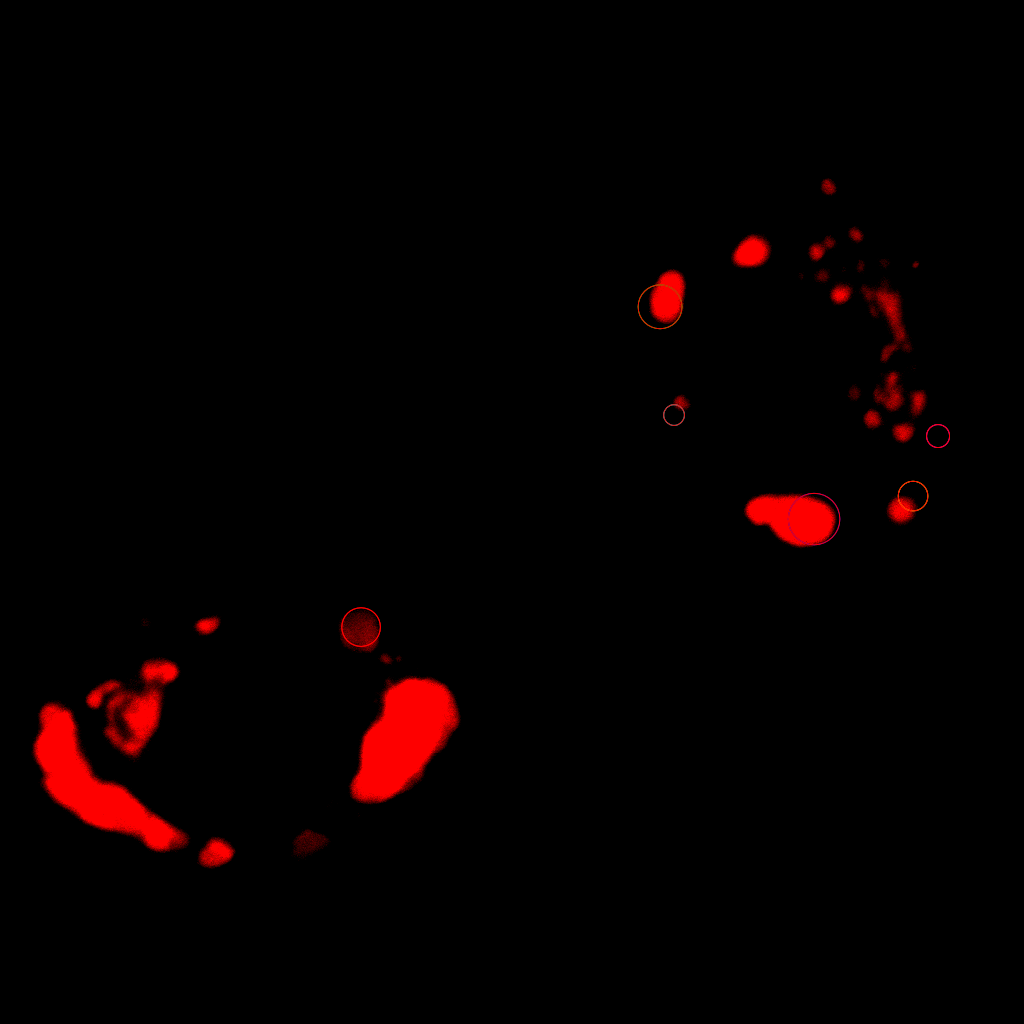

Supplement: Supplementary file 6 — Source data Fig. 4 [file 44319_2024_203_MOESM6_ESM.zip › 4B/18min_FRAP4_MCH-RASSF8 1MIN TIME[OINT_t20c1.tif]

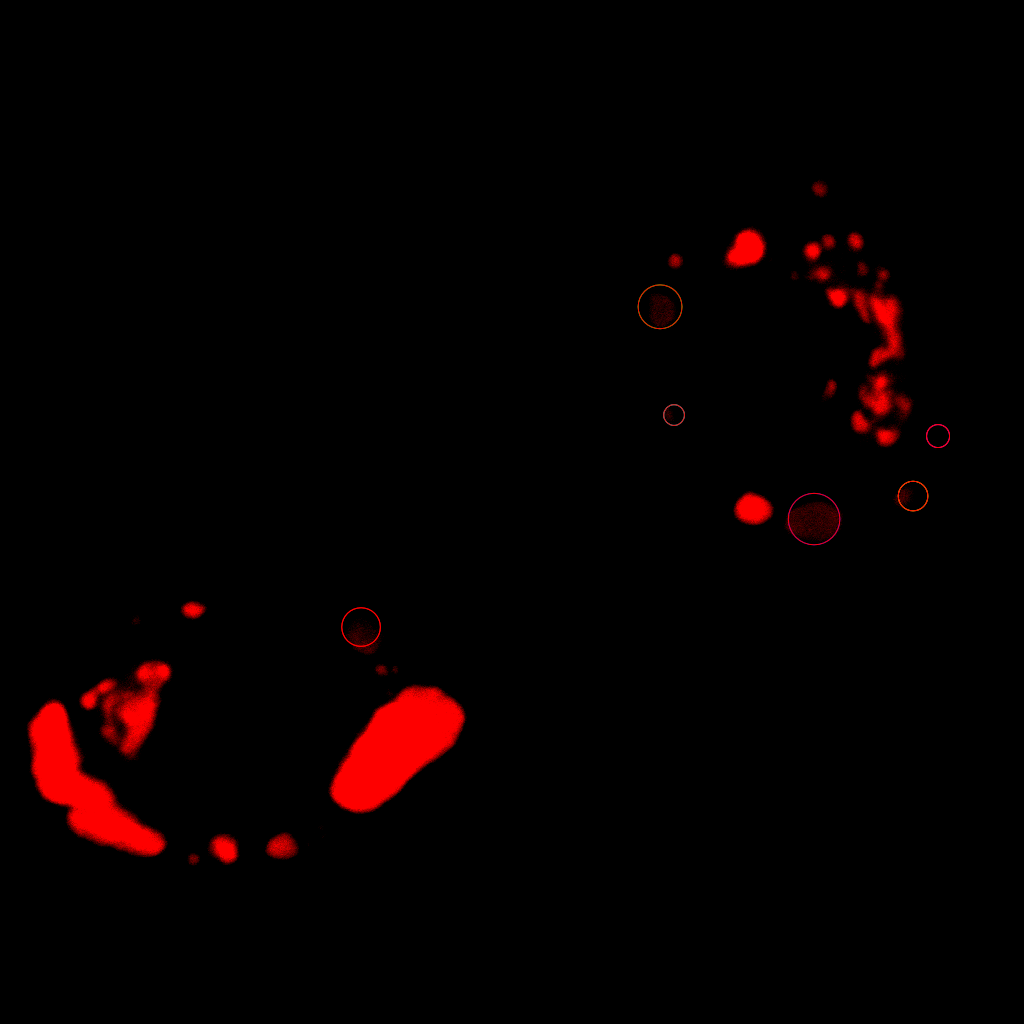

Supplement: Supplementary file 6 — Source data Fig. 4 [file 44319_2024_203_MOESM6_ESM.zip › 4B/1min_FRAP4_MCH-RASSF8 1MIN TIME[OINT_t03c1.tif]

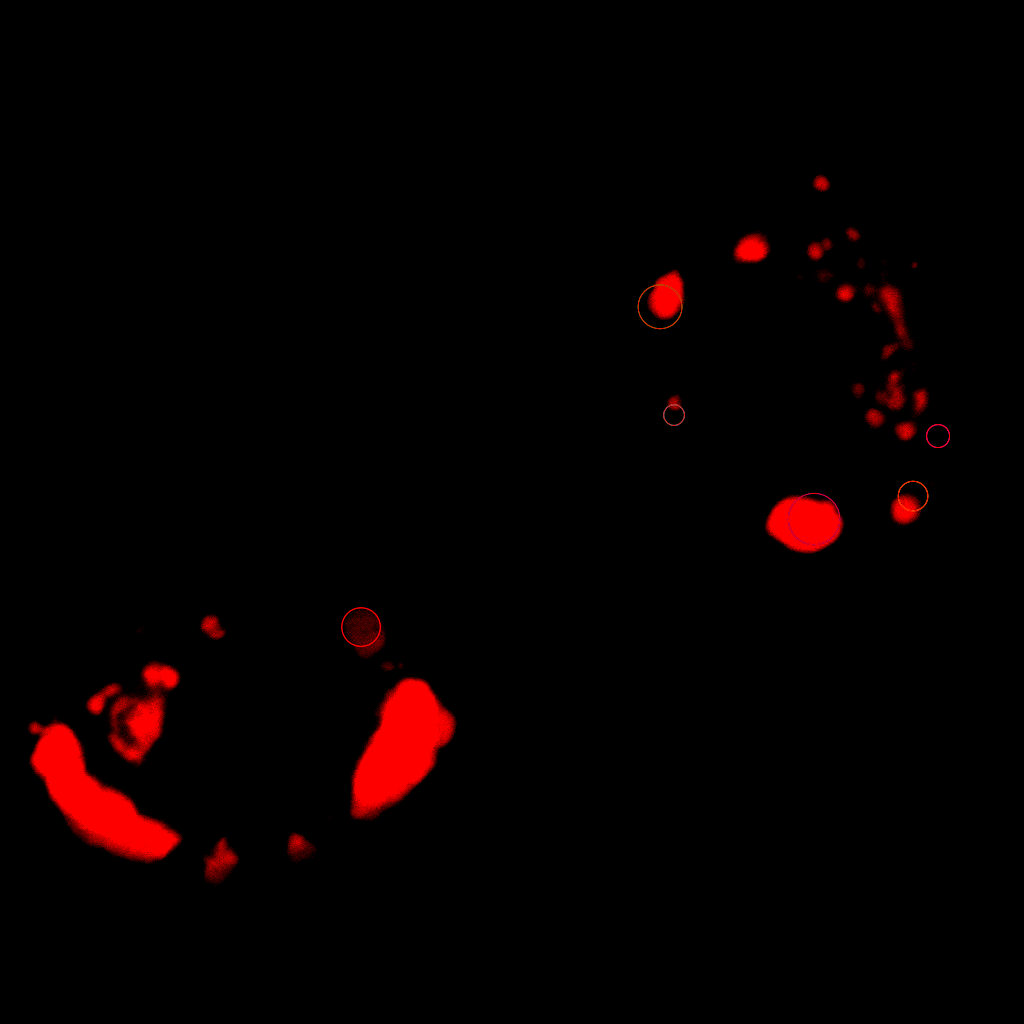

Supplement: Supplementary file 6 — Source data Fig. 4 [file 44319_2024_203_MOESM6_ESM.zip › 4B/24min_FRAP4_MCH-RASSF8 1MIN TIME[OINT_t26c1.tif]

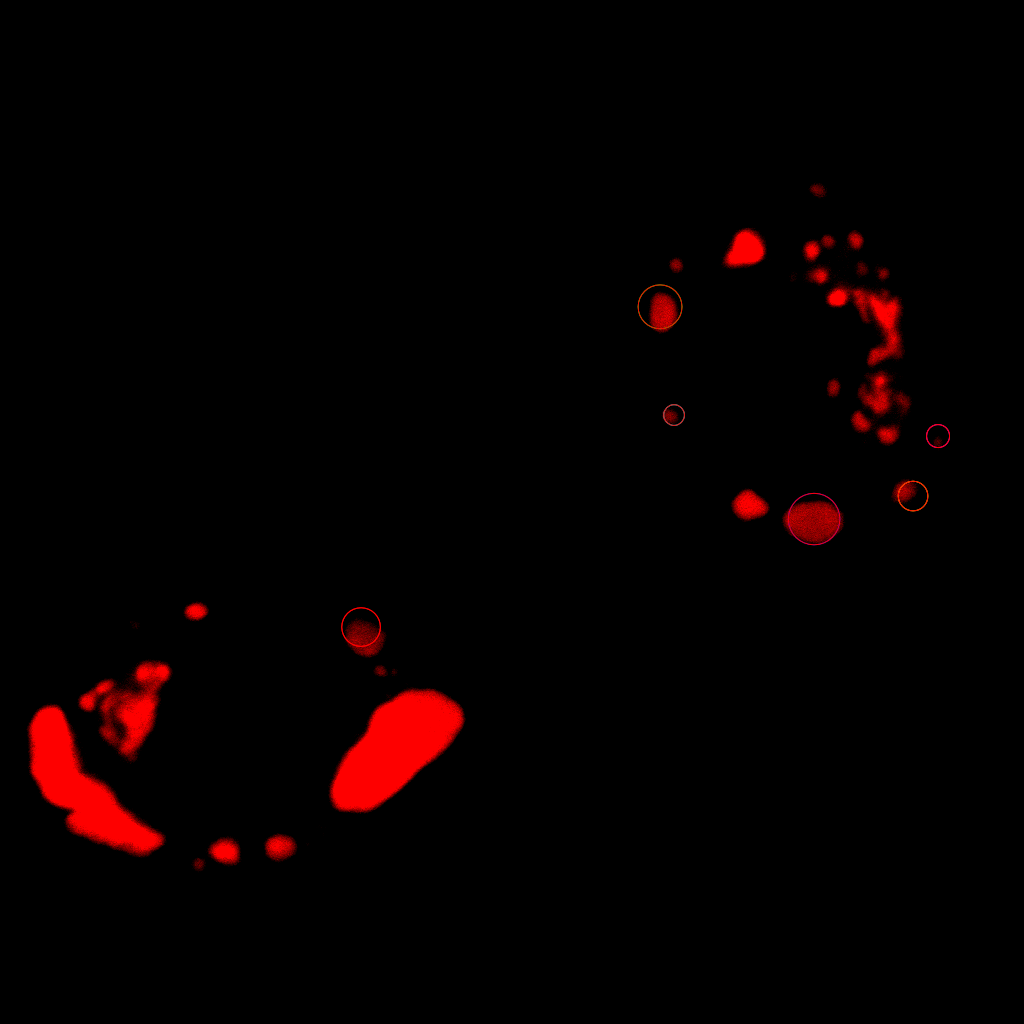

Supplement: Supplementary file 6 — Source data Fig. 4 [file 44319_2024_203_MOESM6_ESM.zip › 4B/2min_FRAP4_MCH-RASSF8 1MIN TIME[OINT_t04c1.tif]

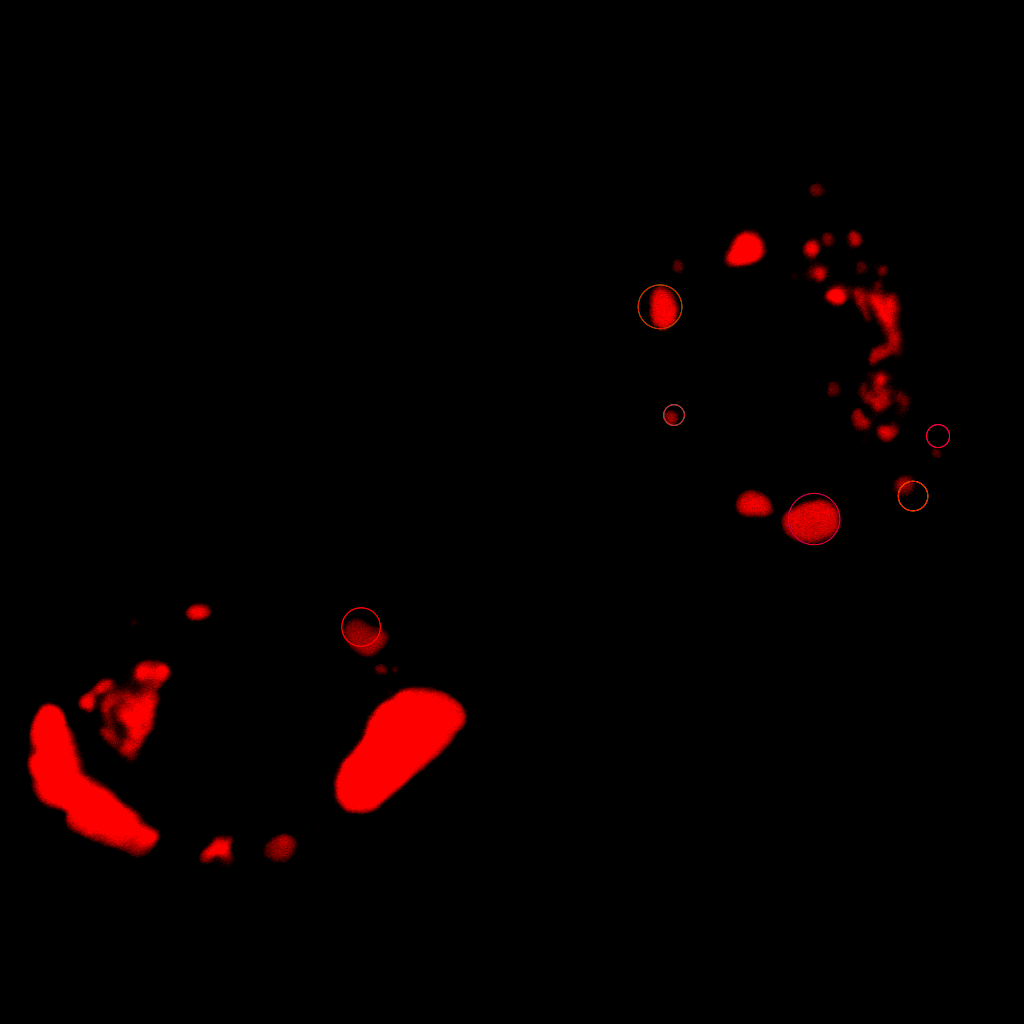

Supplement: Supplementary file 6 — Source data Fig. 4 [file 44319_2024_203_MOESM6_ESM.zip › 4B/3min_FRAP4_MCH-RASSF8 1MIN TIME[OINT_t05c1.tif]

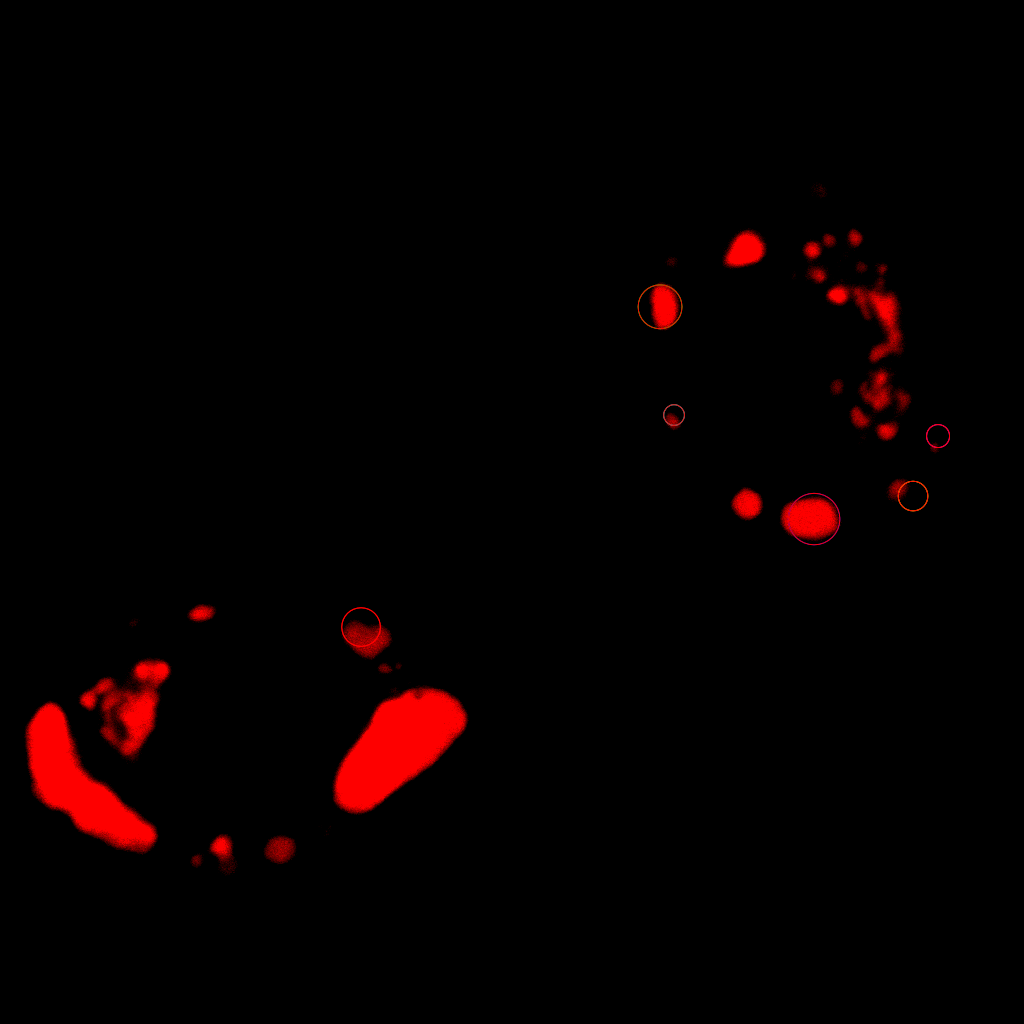

Supplement: Supplementary file 6 — Source data Fig. 4 [file 44319_2024_203_MOESM6_ESM.zip › 4B/4min_FRAP4_MCH-RASSF8 1MIN TIME[OINT_t06c1.tif]

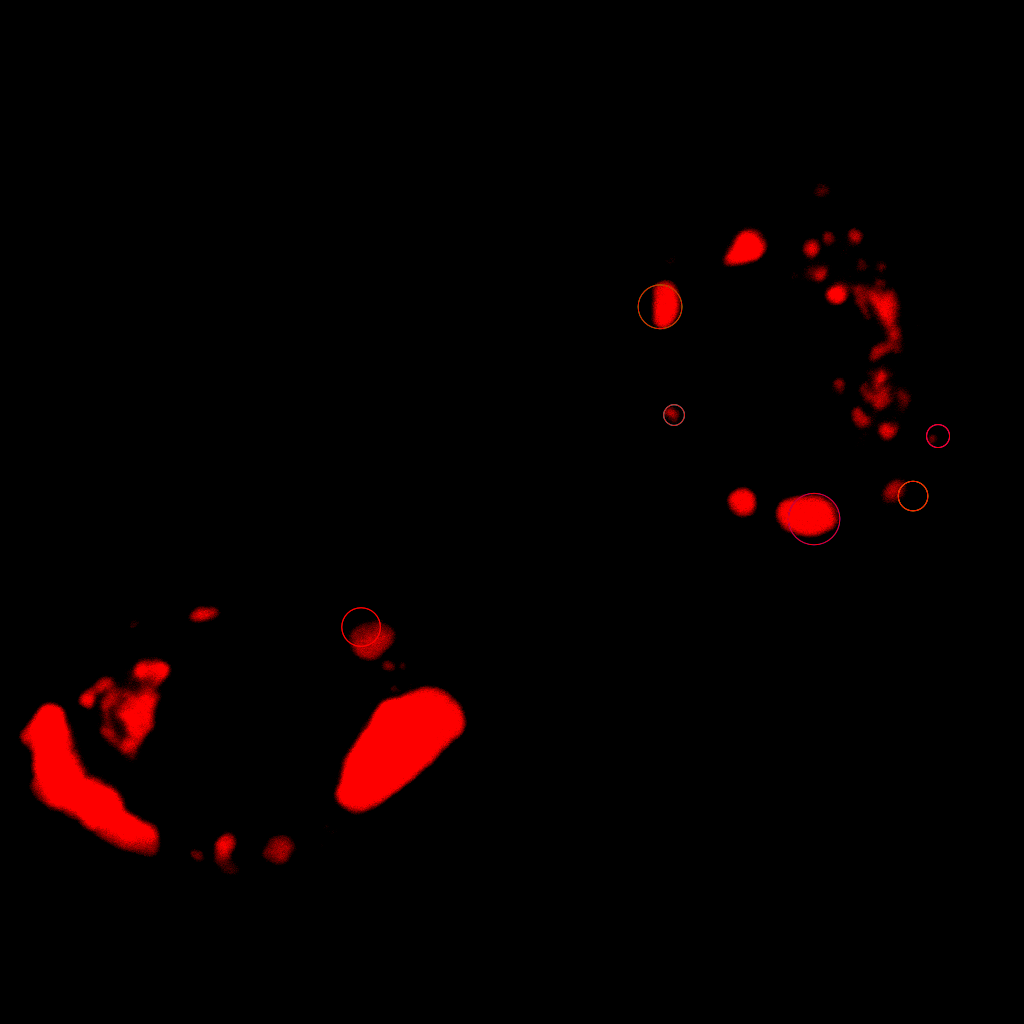

Supplement: Supplementary file 6 — Source data Fig. 4 [file 44319_2024_203_MOESM6_ESM.zip › 4B/5min_FRAP4_MCH-RASSF8 1MIN TIME[OINT_t07c1.tif]

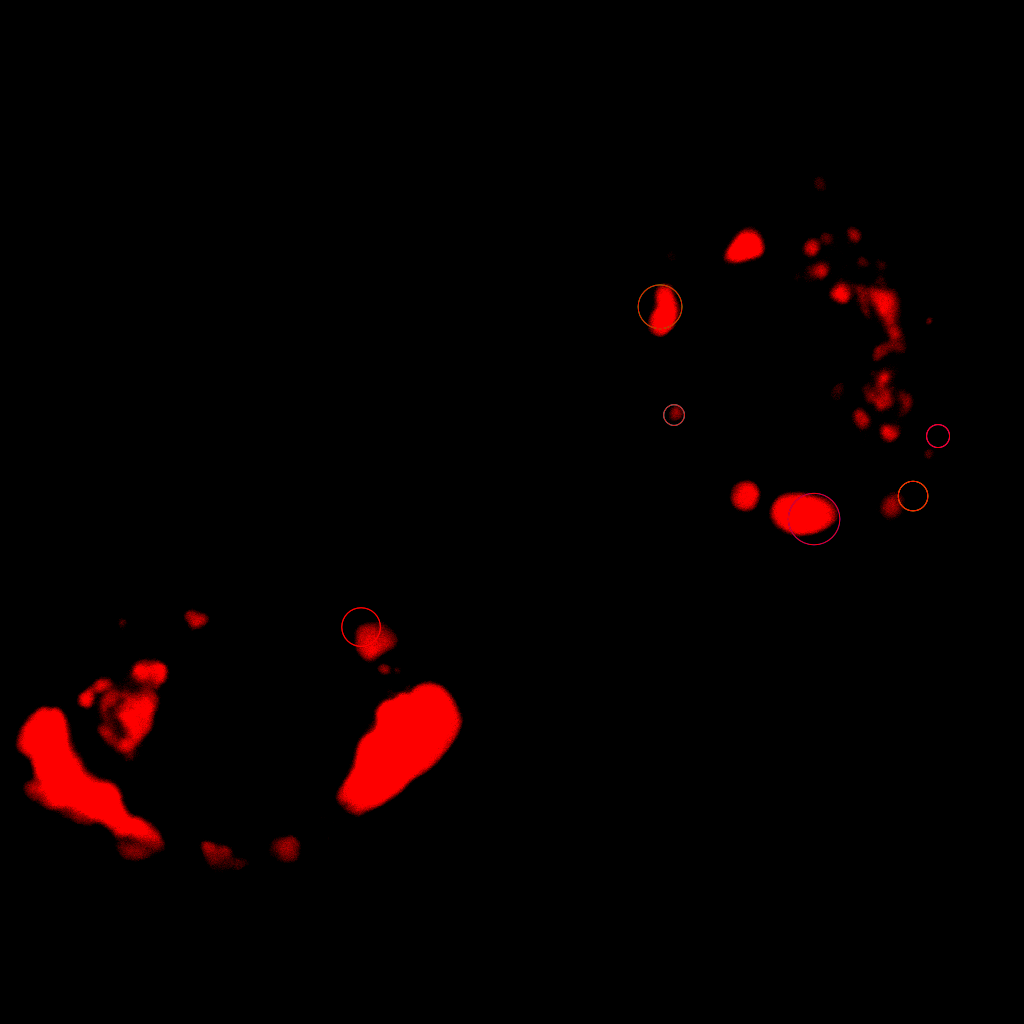

Supplement: Supplementary file 6 — Source data Fig. 4 [file 44319_2024_203_MOESM6_ESM.zip › 4B/8min_FRAP4_MCH-RASSF8 1MIN TIME[OINT_t10c1.tif]

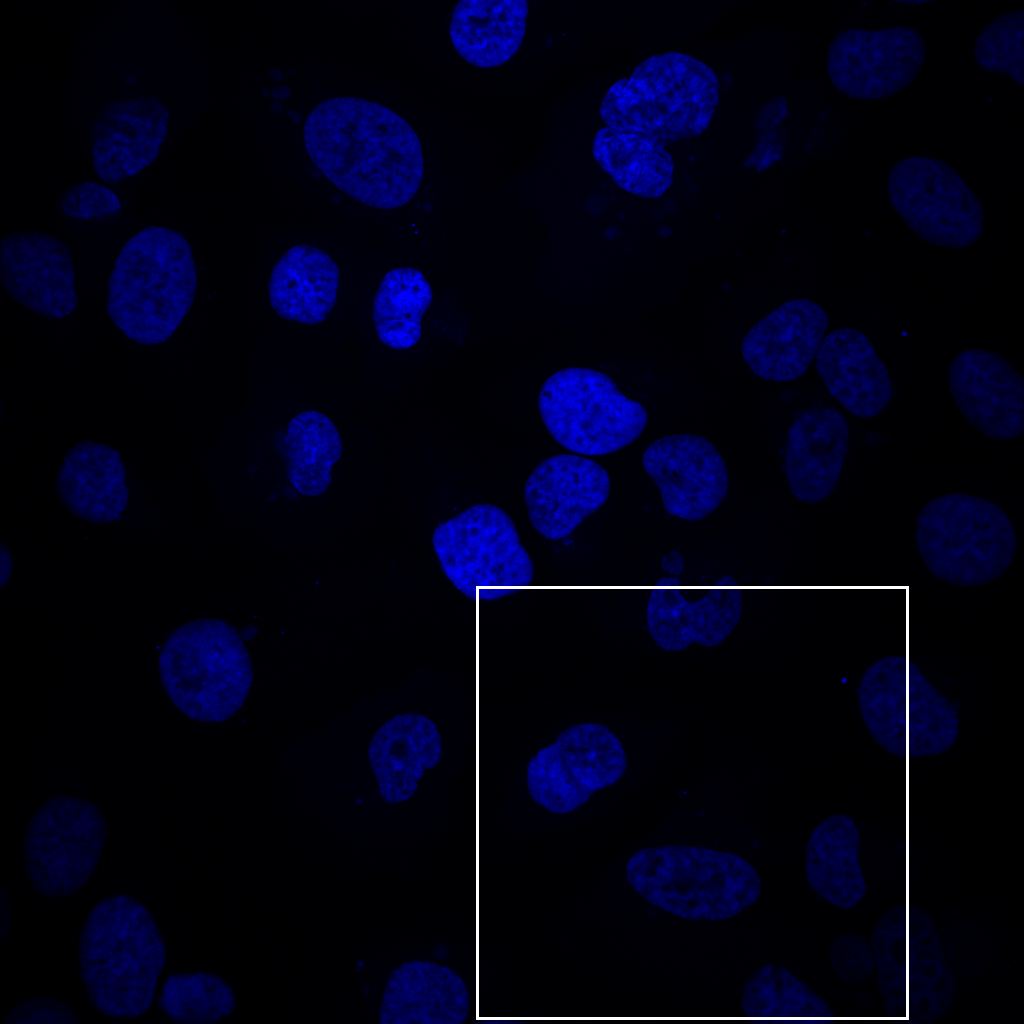

Supplement: Supplementary file 6 — Source data Fig. 4 [file 44319_2024_203_MOESM6_ESM.zip › 4D/Hoechst.tif]
