# Supplementary figures and images for: Complex interplay between RAS GTPases and RASSF effectors regulates subcellular localization of YAP (part 2 of 4)
Source: EMBO Rep. 2024 Jul 15;25(8):22. doi: 10.1038/s44319-024-00203-9 (PMC11316025; doi:10.1038/s44319-024-00203-9)

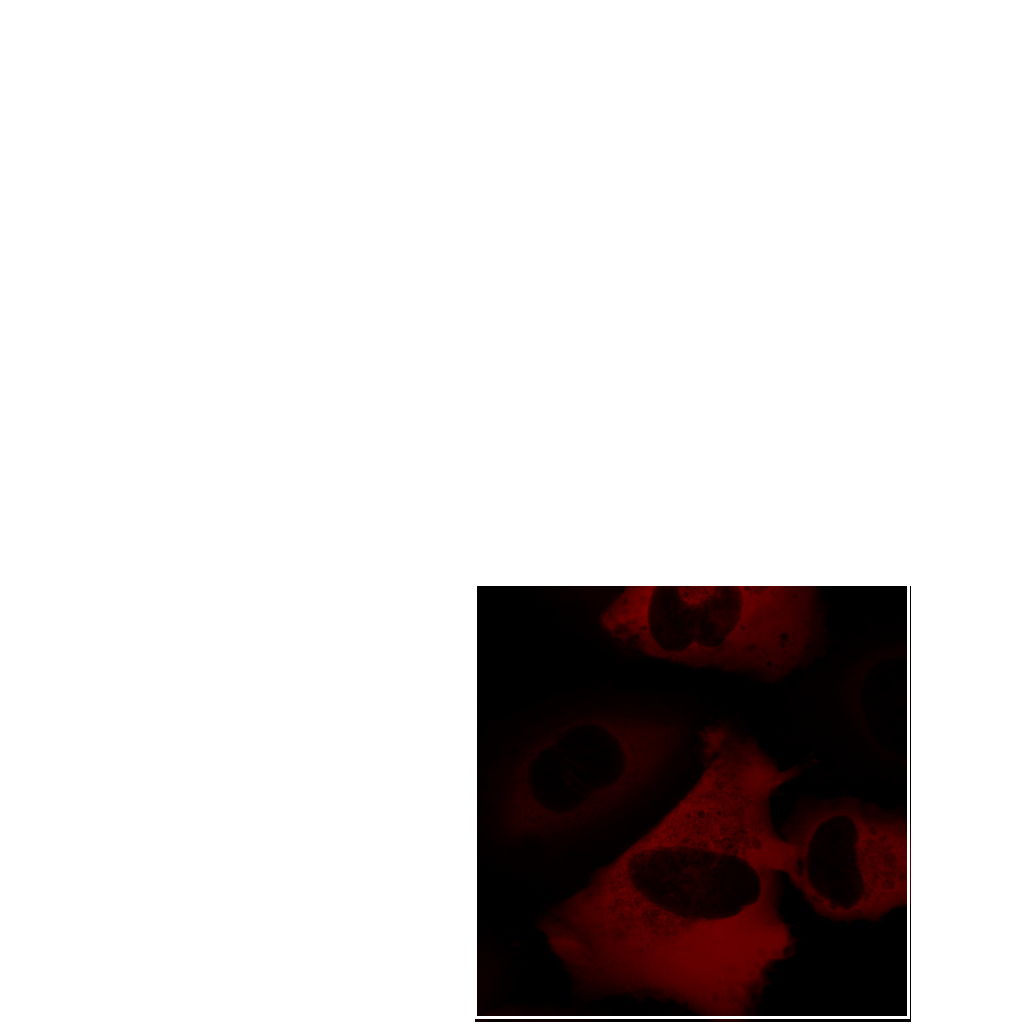

Supplement: Supplementary file 6 — Source data Fig. 4 [file 44319_2024_203_MOESM6_ESM.zip › 4D/RASSF8-delcc.tif]

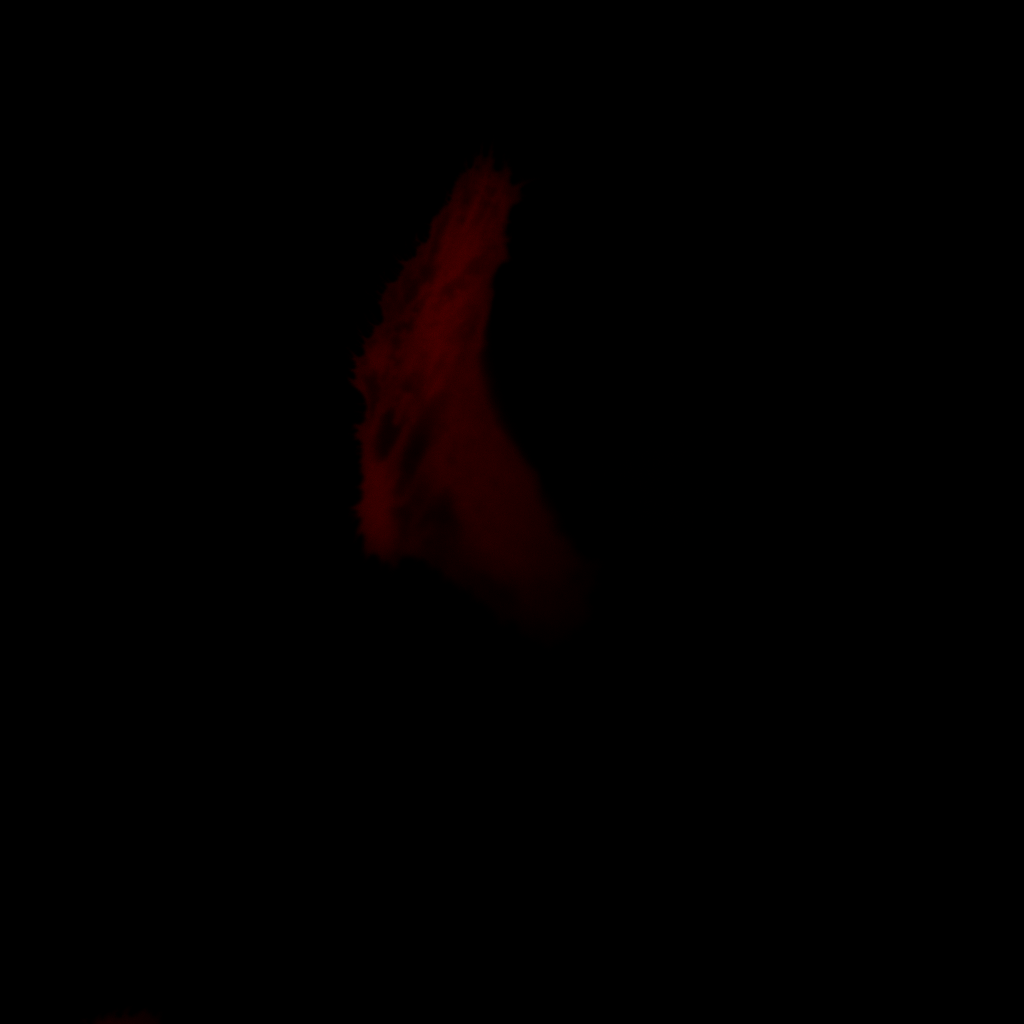

Supplement: Supplementary file 6 — Source data Fig. 4 [file 44319_2024_203_MOESM6_ESM.zip › 4F/RASSF8-delCC_Middle/After wash 20 mins/U2OS_mchRASSF8-RBD-delC-sorbitol_30mins_2023_07_26__18_20_04_s04z11.tif]

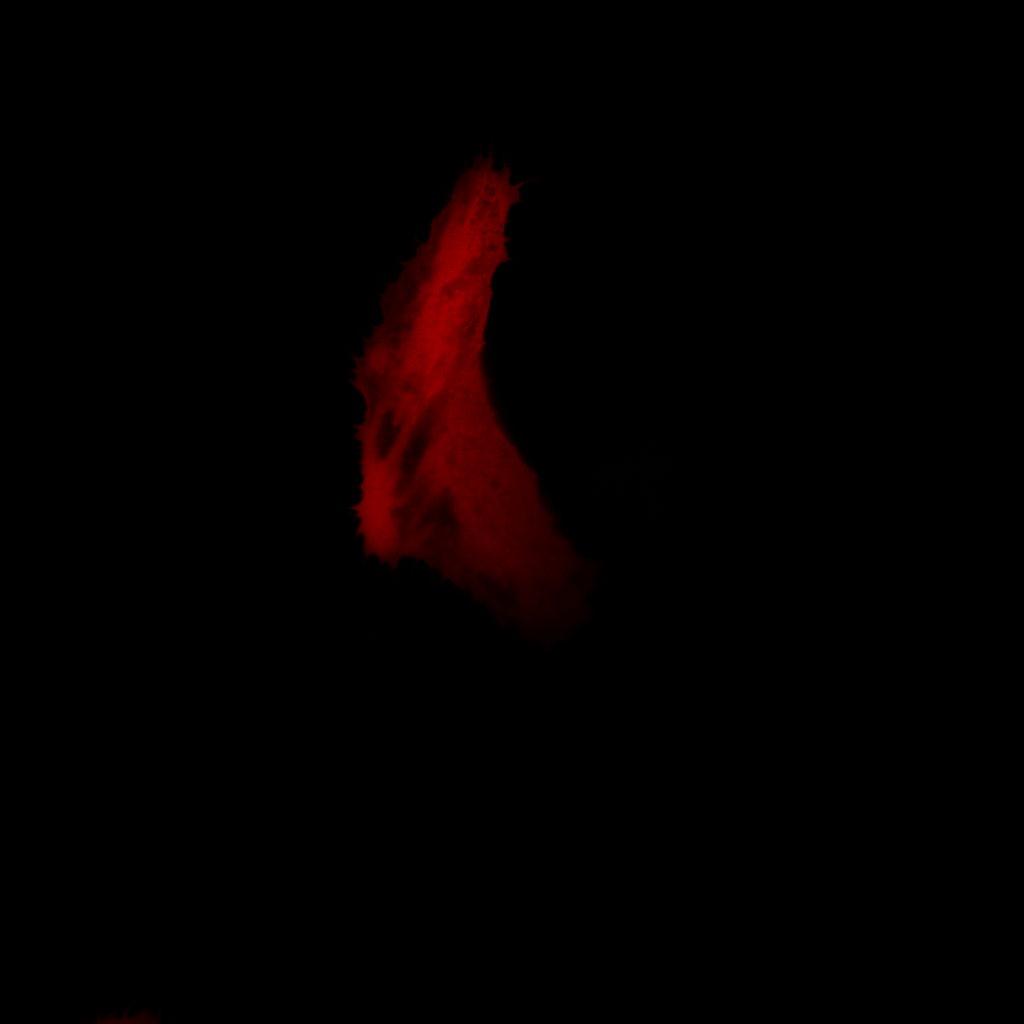

Supplement: Supplementary file 6 — Source data Fig. 4 [file 44319_2024_203_MOESM6_ESM.zip › 4F/RASSF8-delCC_Middle/After wash 20 mins/U2OS_mchRASSF8-RBD-delC-sorbitol_30mins_2023_07_26__18_20_04_s04z12.tif]

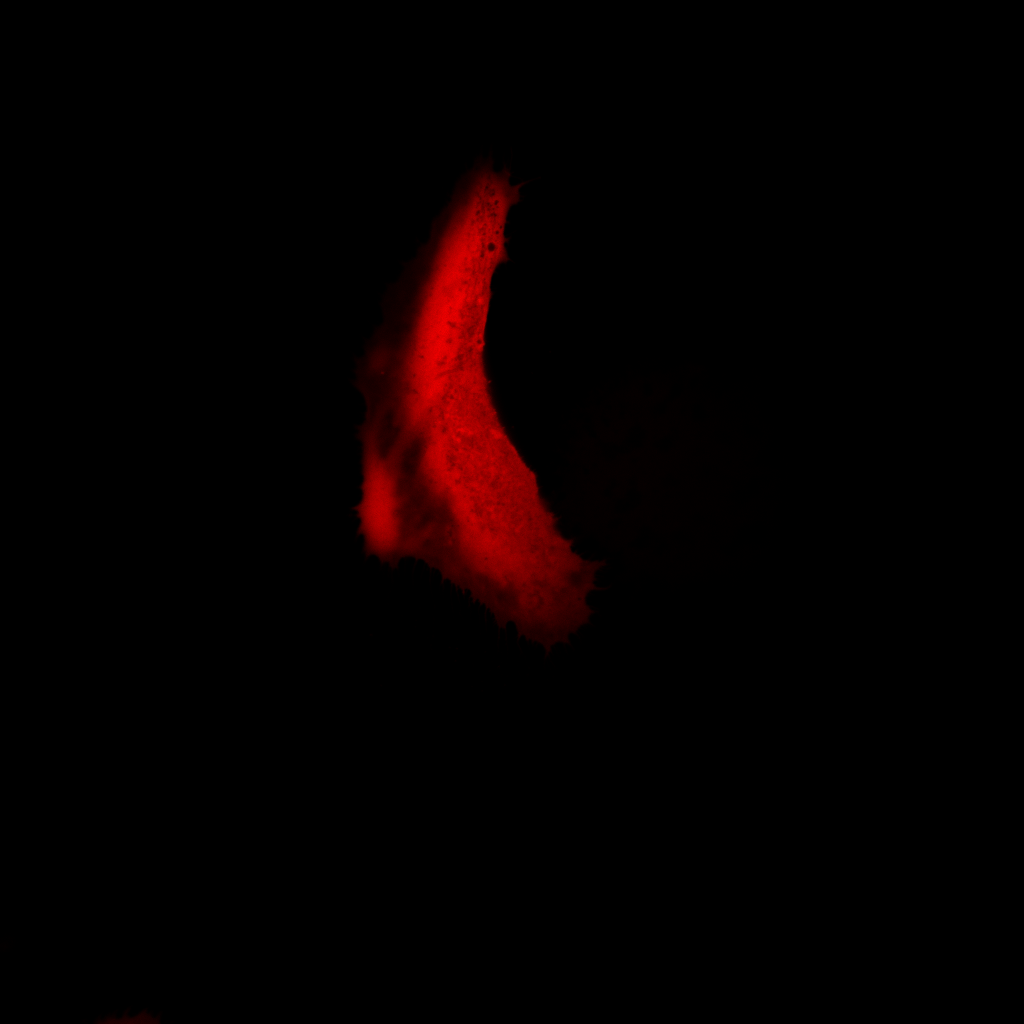

Supplement: Supplementary file 6 — Source data Fig. 4 [file 44319_2024_203_MOESM6_ESM.zip › 4F/RASSF8-delCC_Middle/After wash 20 mins/U2OS_mchRASSF8-RBD-delC-sorbitol_30mins_2023_07_26__18_20_04_s04z13.tif]

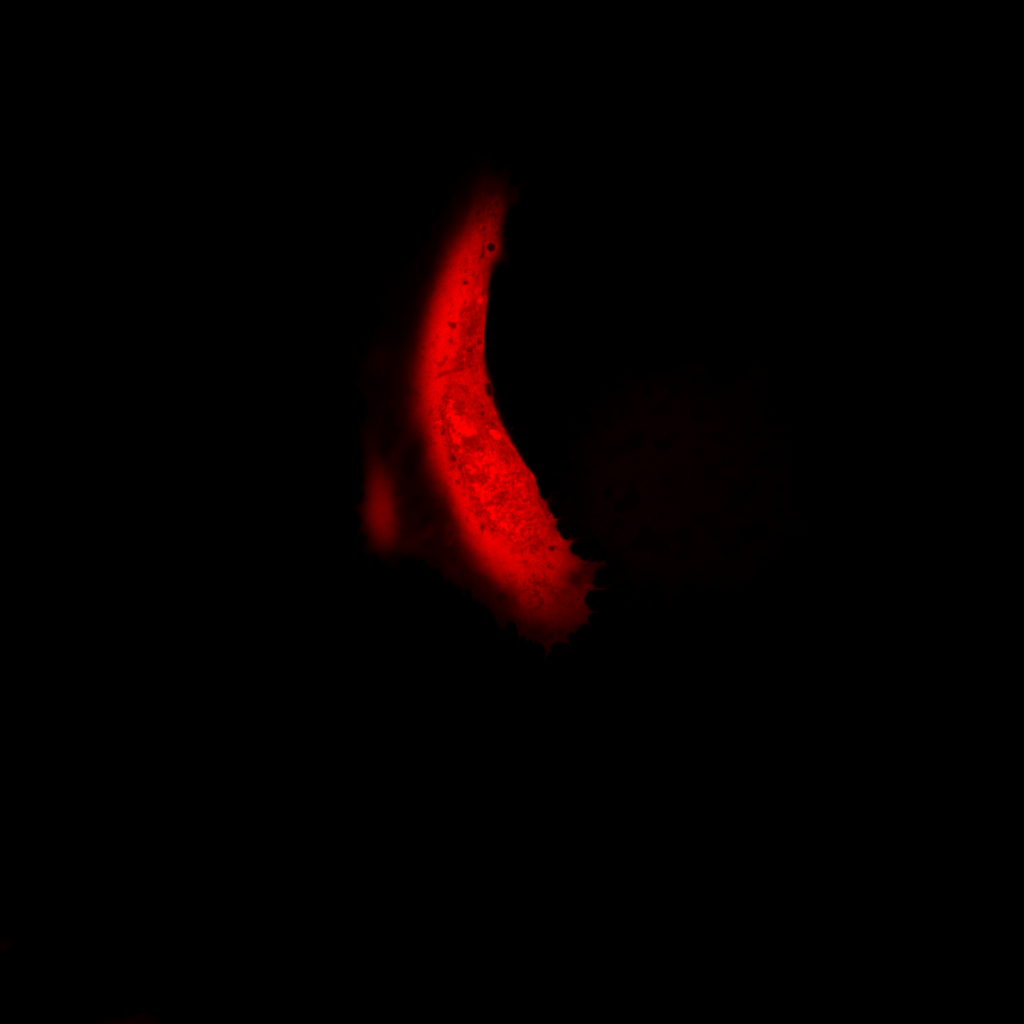

Supplement: Supplementary file 6 — Source data Fig. 4 [file 44319_2024_203_MOESM6_ESM.zip › 4F/RASSF8-delCC_Middle/After wash 20 mins/U2OS_mchRASSF8-RBD-delC-sorbitol_30mins_2023_07_26__18_20_04_s04z14.tif]

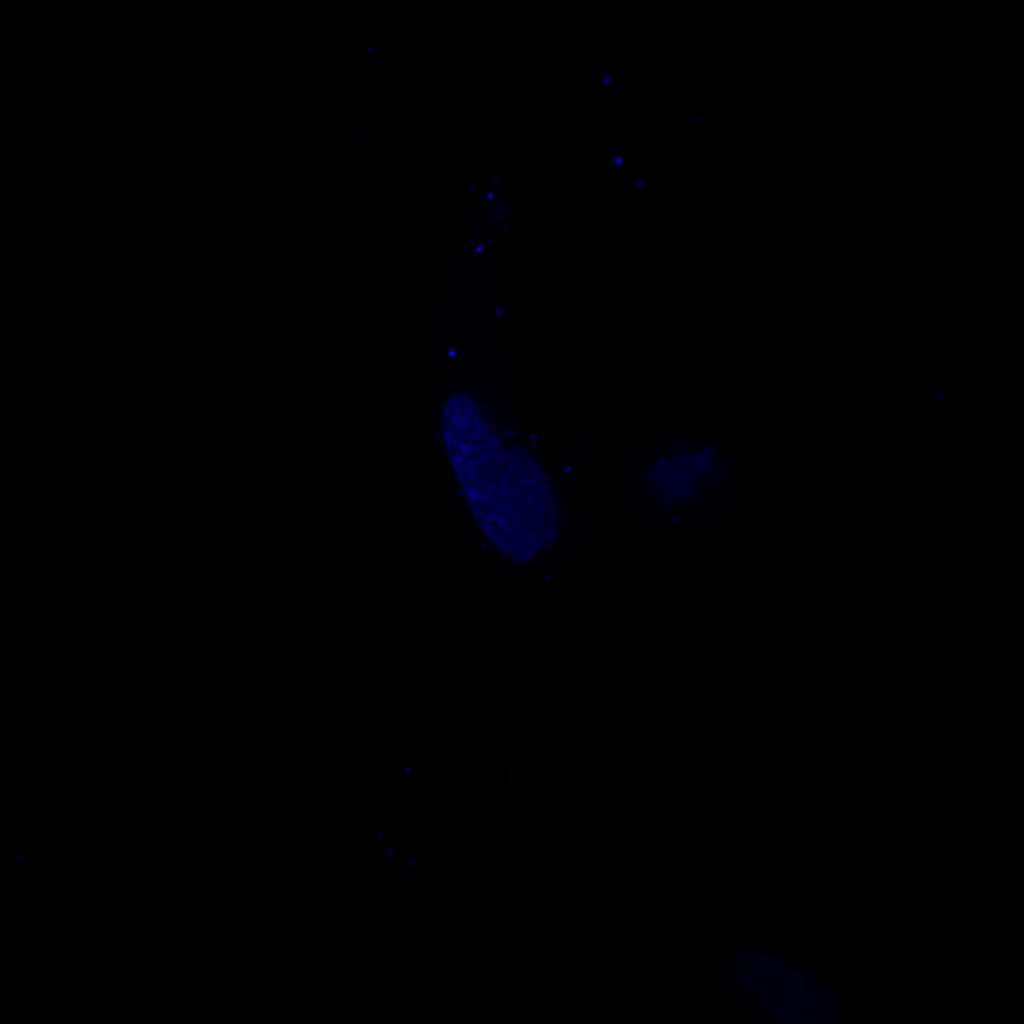

Supplement: Supplementary file 6 — Source data Fig. 4 [file 44319_2024_203_MOESM6_ESM.zip › 4F/RASSF8-delCC_Middle/Pre-treatment/U2OS_mchRASSF8-RBD-delC-nosorbitol_2023_07_26__17_13_54_s04z15c1.tif]

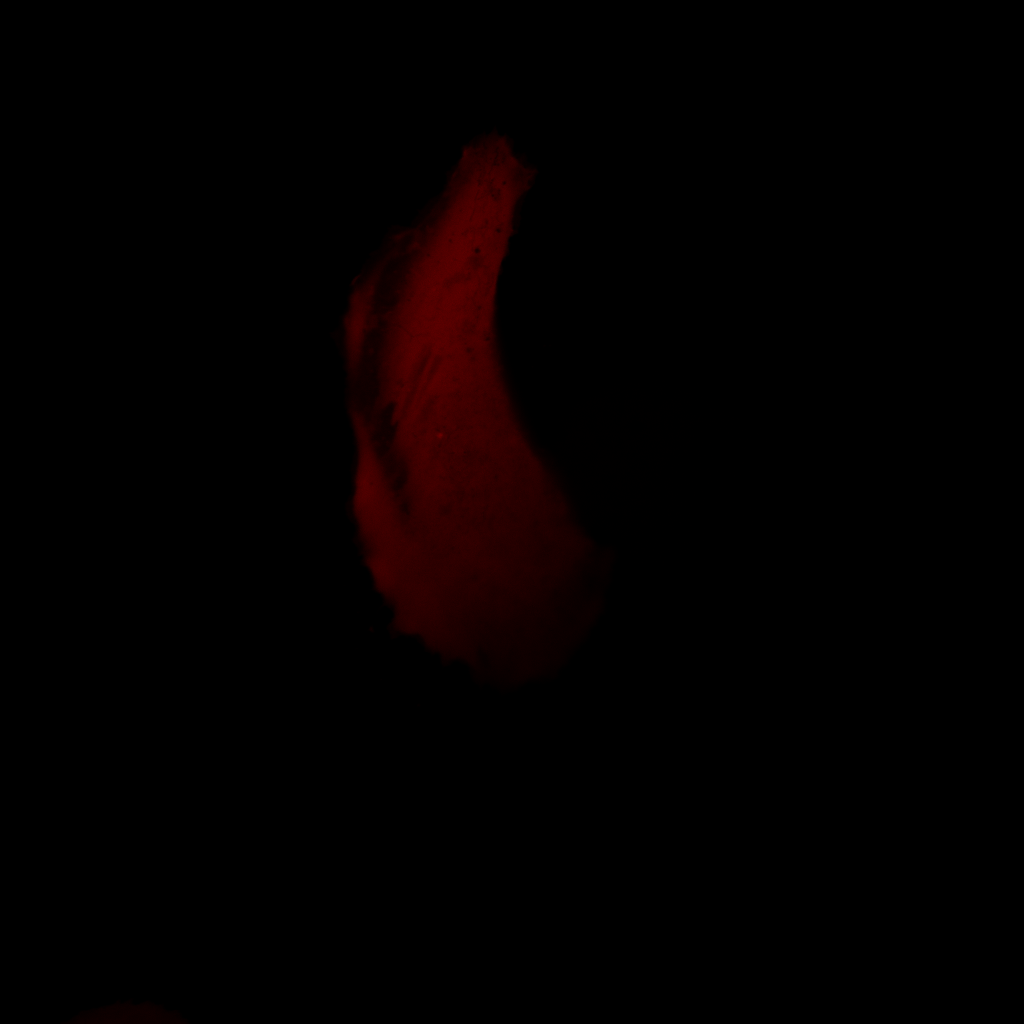

Supplement: Supplementary file 6 — Source data Fig. 4 [file 44319_2024_203_MOESM6_ESM.zip › 4F/RASSF8-delCC_Middle/Pre-treatment/U2OS_mchRASSF8-RBD-delC-nosorbitol_2023_07_26__17_13_54_s04z15c2.tif]

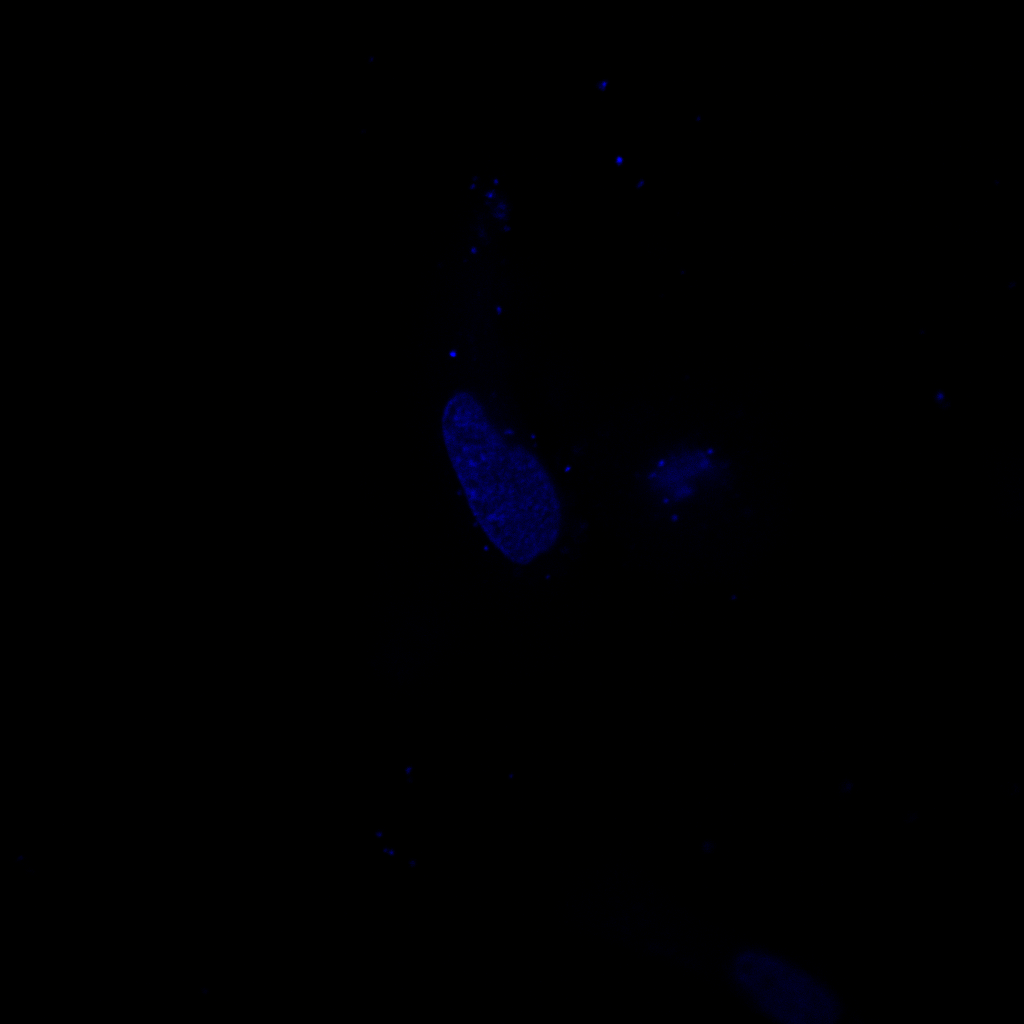

Supplement: Supplementary file 6 — Source data Fig. 4 [file 44319_2024_203_MOESM6_ESM.zip › 4F/RASSF8-delCC_Middle/Pre-treatment/U2OS_mchRASSF8-RBD-delC-nosorbitol_2023_07_26__17_13_54_s04z16c1.tif]

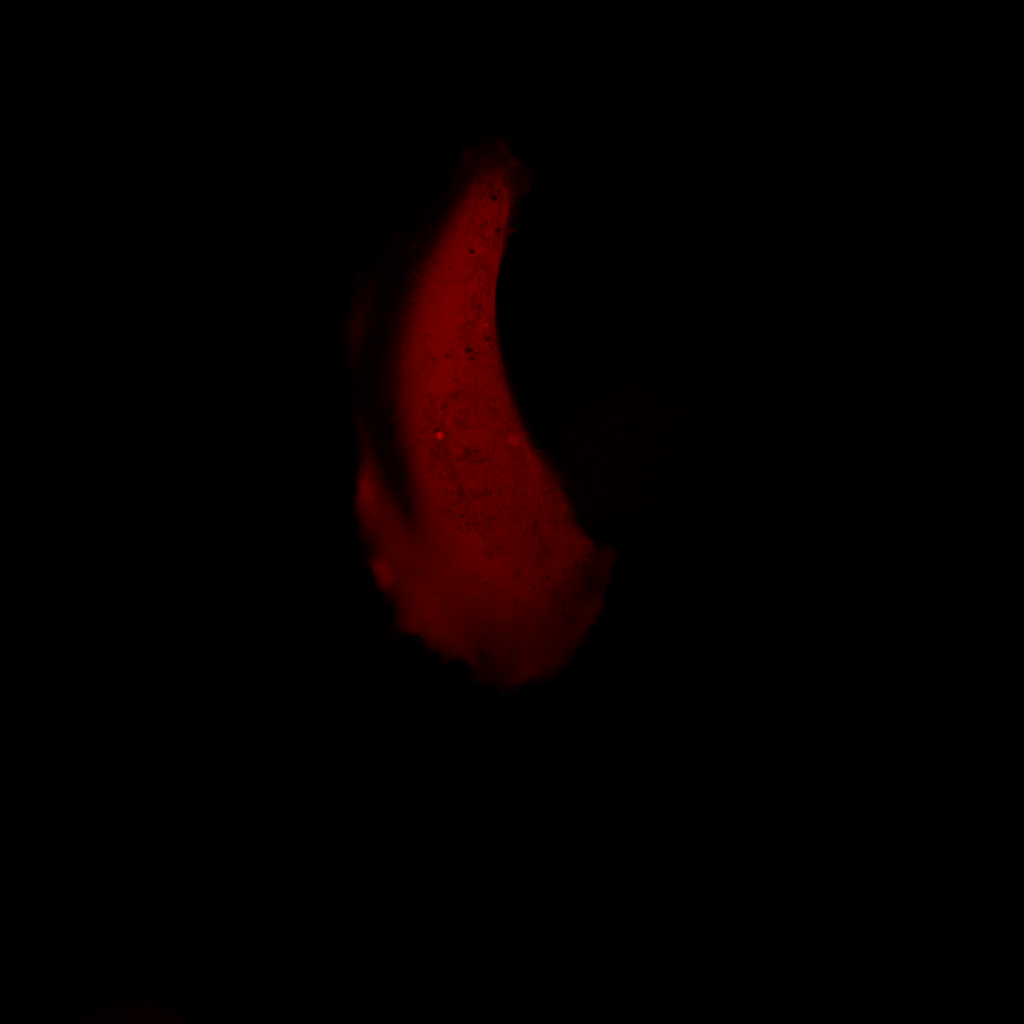

Supplement: Supplementary file 6 — Source data Fig. 4 [file 44319_2024_203_MOESM6_ESM.zip › 4F/RASSF8-delCC_Middle/Pre-treatment/U2OS_mchRASSF8-RBD-delC-nosorbitol_2023_07_26__17_13_54_s04z16c2.tif]

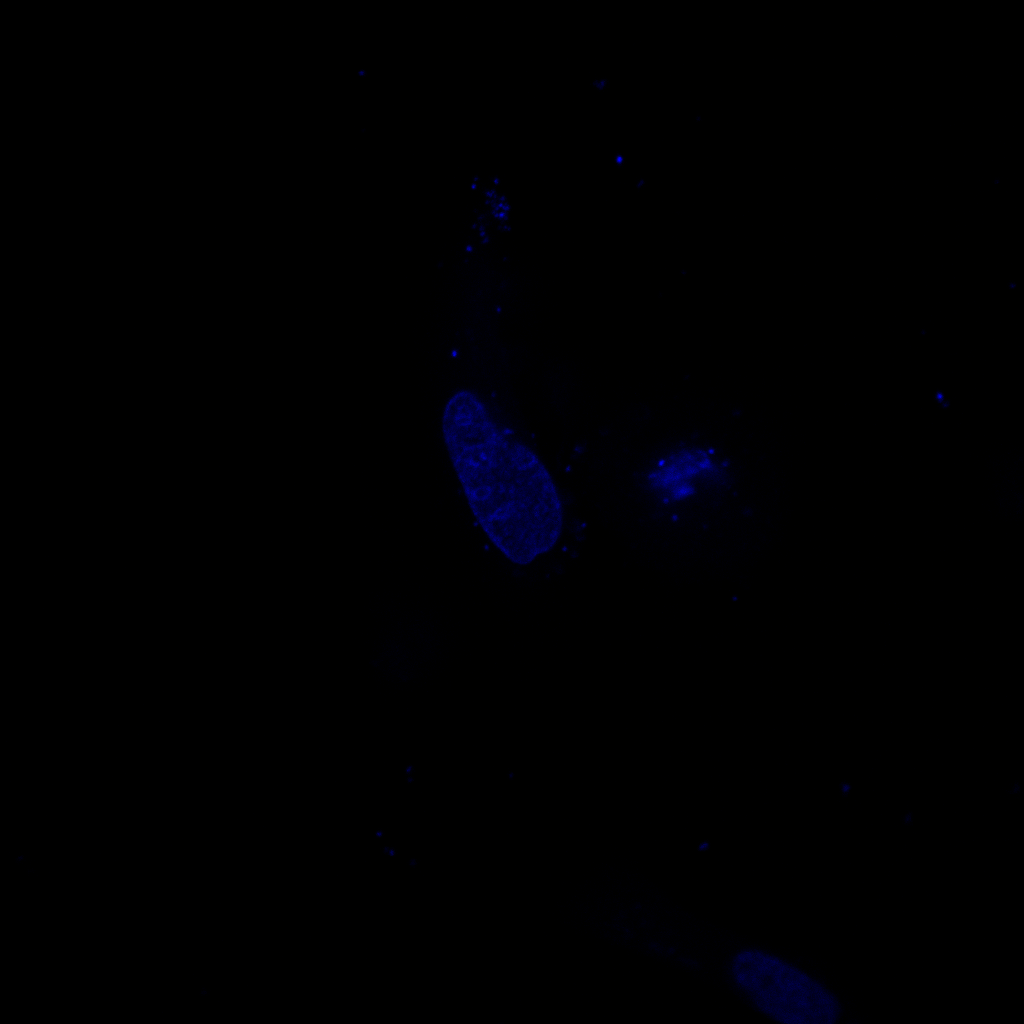

Supplement: Supplementary file 6 — Source data Fig. 4 [file 44319_2024_203_MOESM6_ESM.zip › 4F/RASSF8-delCC_Middle/Pre-treatment/U2OS_mchRASSF8-RBD-delC-nosorbitol_2023_07_26__17_13_54_s04z17c1.tif]

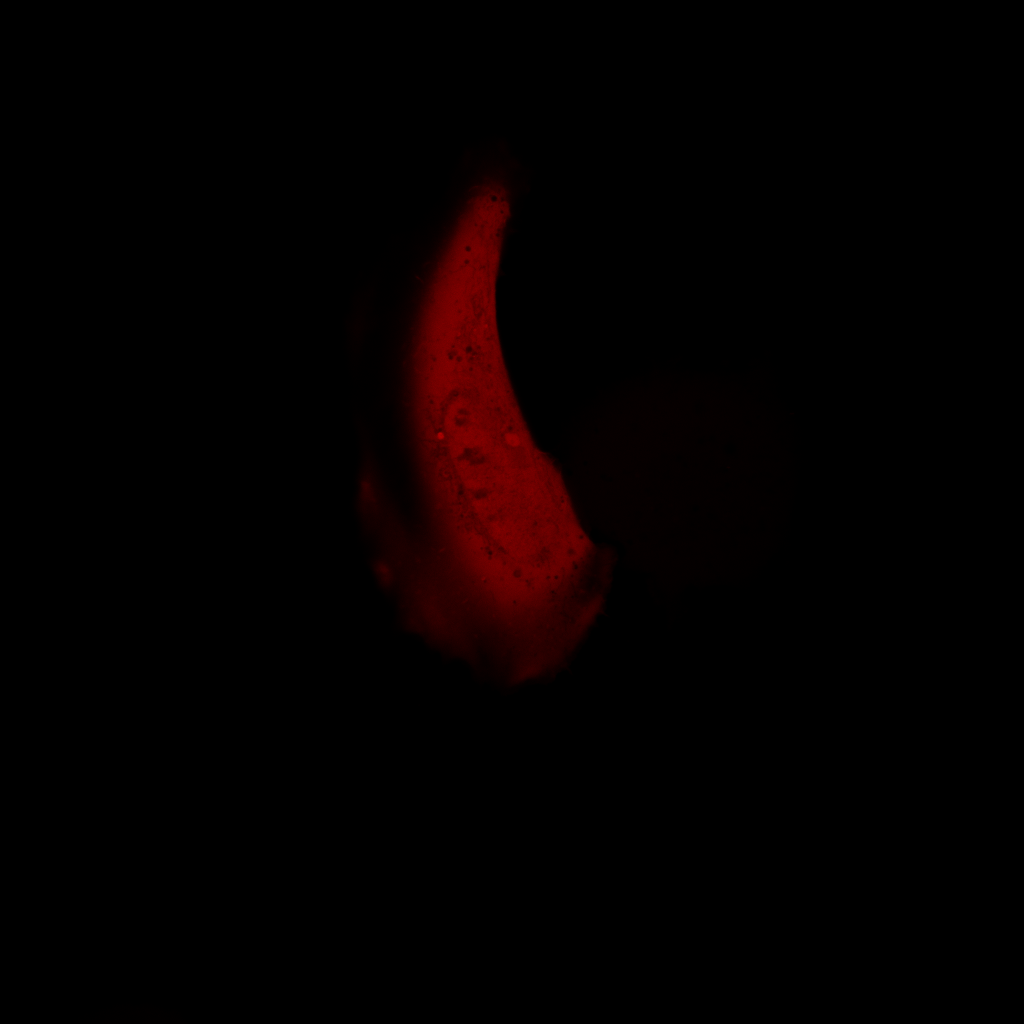

Supplement: Supplementary file 6 — Source data Fig. 4 [file 44319_2024_203_MOESM6_ESM.zip › 4F/RASSF8-delCC_Middle/Pre-treatment/U2OS_mchRASSF8-RBD-delC-nosorbitol_2023_07_26__17_13_54_s04z17c2.tif]

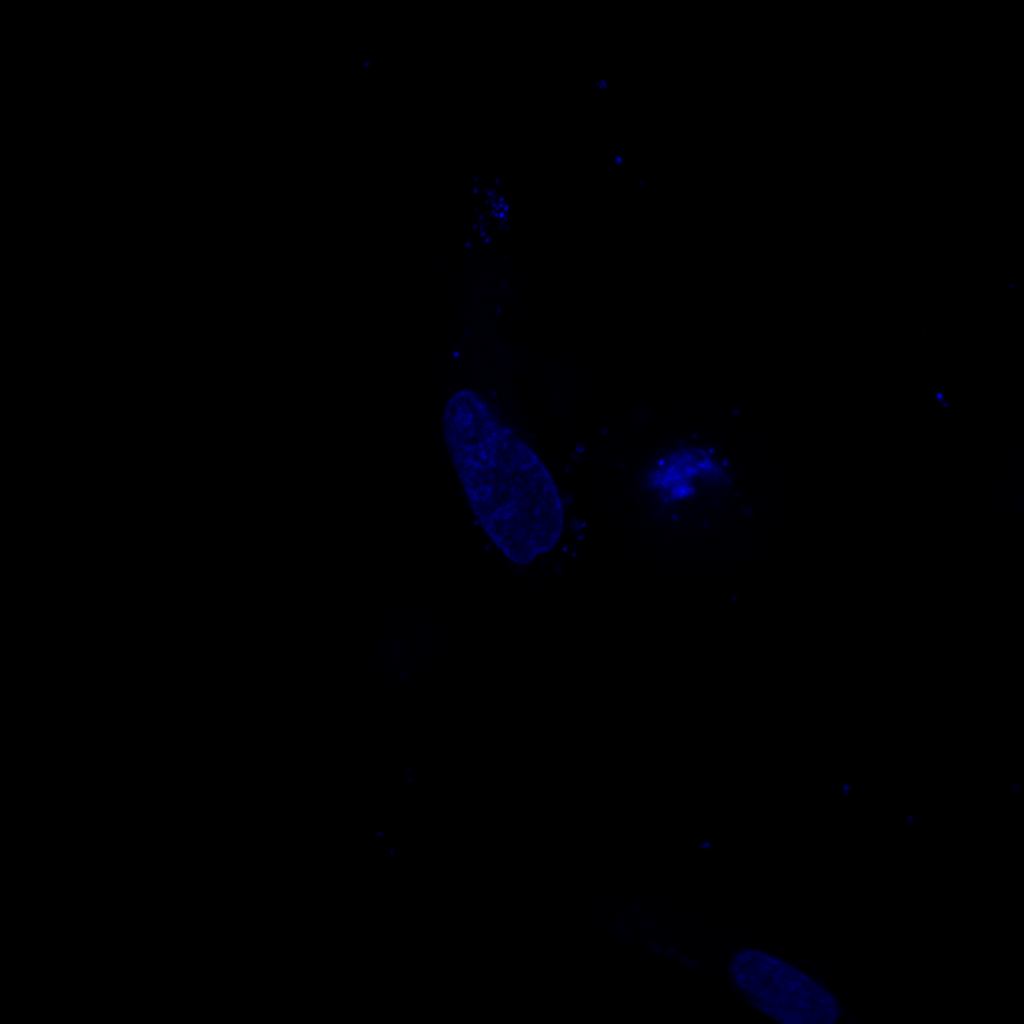

Supplement: Supplementary file 6 — Source data Fig. 4 [file 44319_2024_203_MOESM6_ESM.zip › 4F/RASSF8-delCC_Middle/Pre-treatment/U2OS_mchRASSF8-RBD-delC-nosorbitol_2023_07_26__17_13_54_s04z18c1.tif]

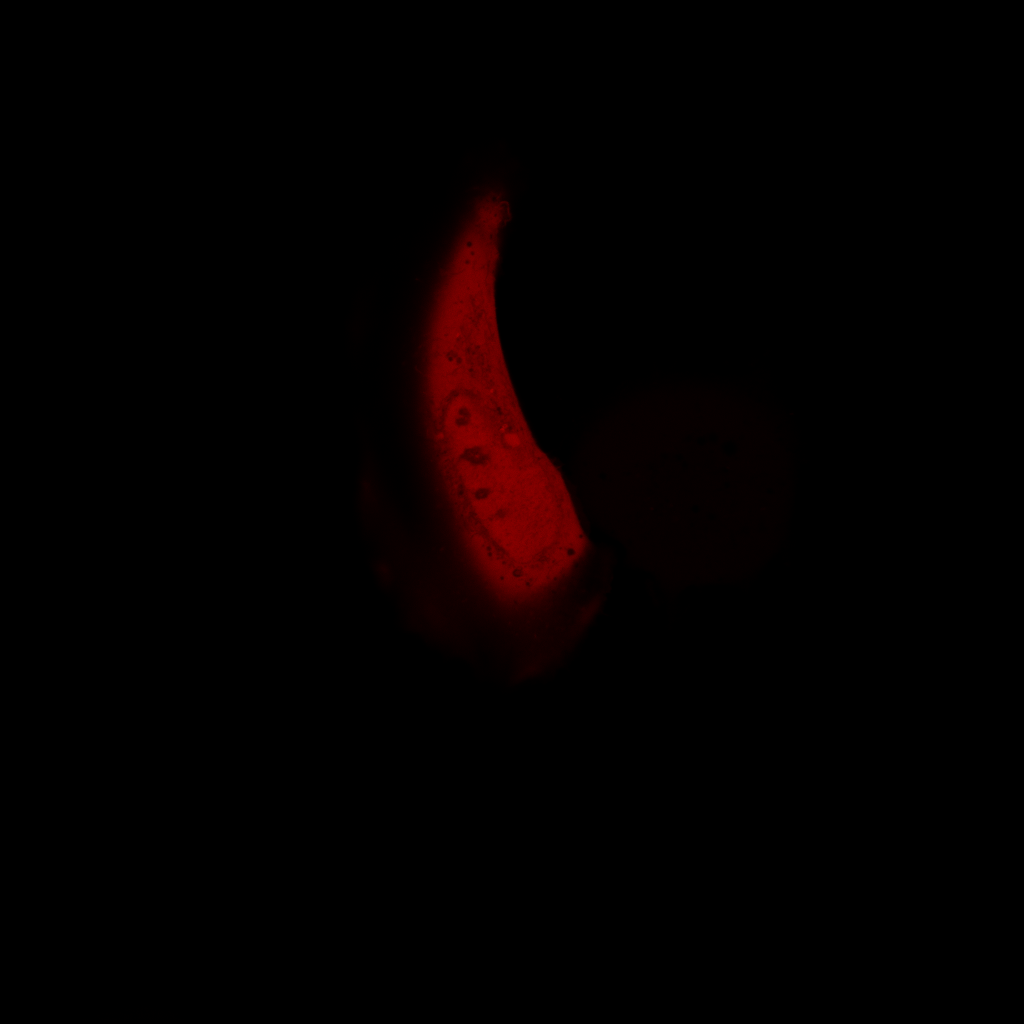

Supplement: Supplementary file 6 — Source data Fig. 4 [file 44319_2024_203_MOESM6_ESM.zip › 4F/RASSF8-delCC_Middle/Pre-treatment/U2OS_mchRASSF8-RBD-delC-nosorbitol_2023_07_26__17_13_54_s04z18c2.tif]

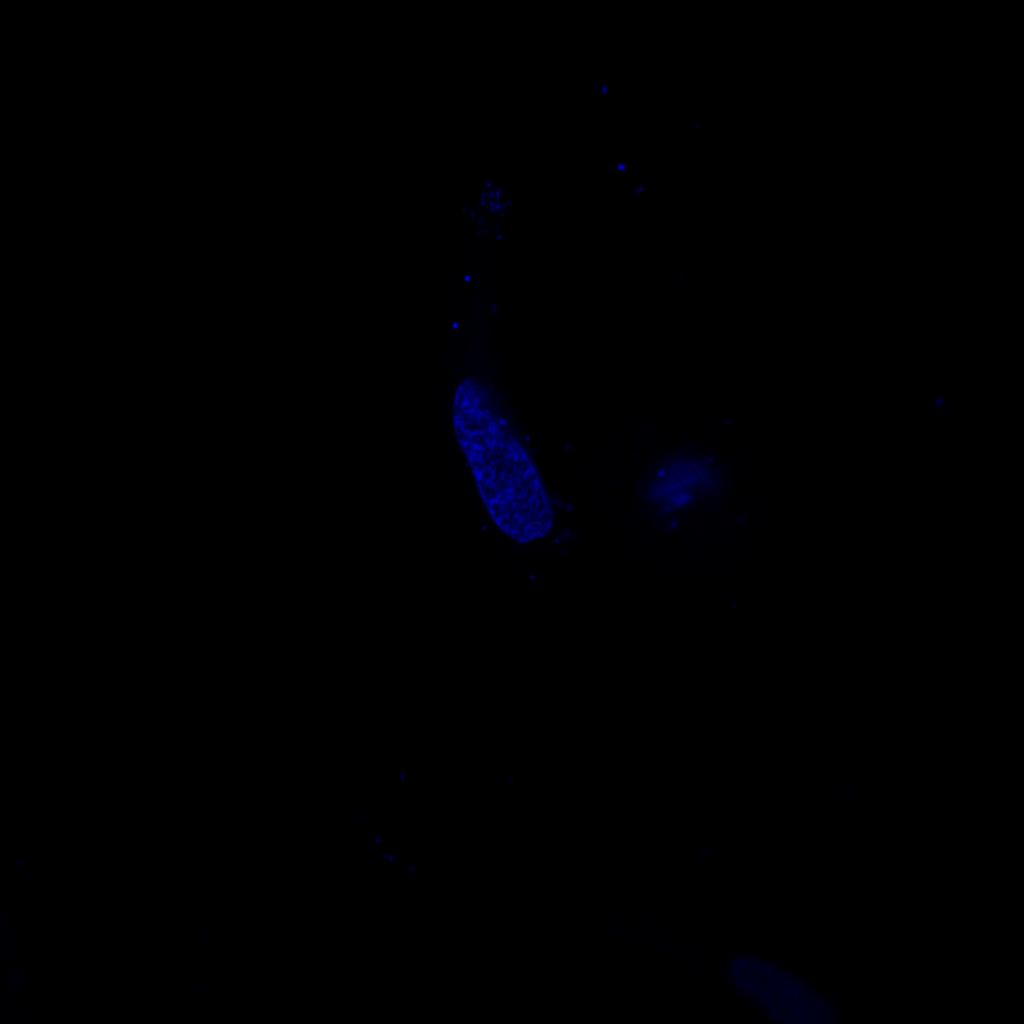

Supplement: Supplementary file 6 — Source data Fig. 4 [file 44319_2024_203_MOESM6_ESM.zip › 4F/RASSF8-delCC_Middle/Sorbitol/U2OS_mchRASSF8-RBD-delC-sorbitol_2mins_2023_07_26__17_49_22_s04z11c1.tif]

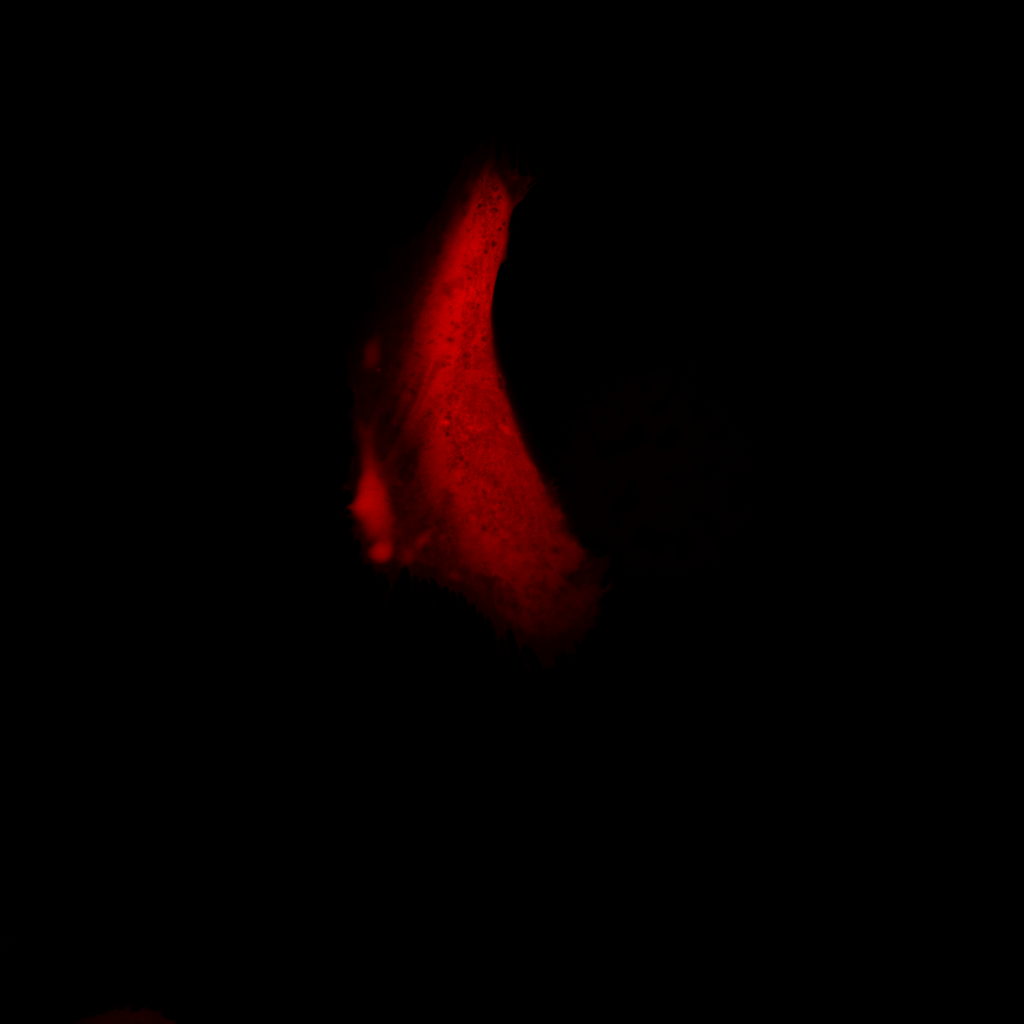

Supplement: Supplementary file 6 — Source data Fig. 4 [file 44319_2024_203_MOESM6_ESM.zip › 4F/RASSF8-delCC_Middle/Sorbitol/U2OS_mchRASSF8-RBD-delC-sorbitol_2mins_2023_07_26__17_49_22_s04z11c2.tif]

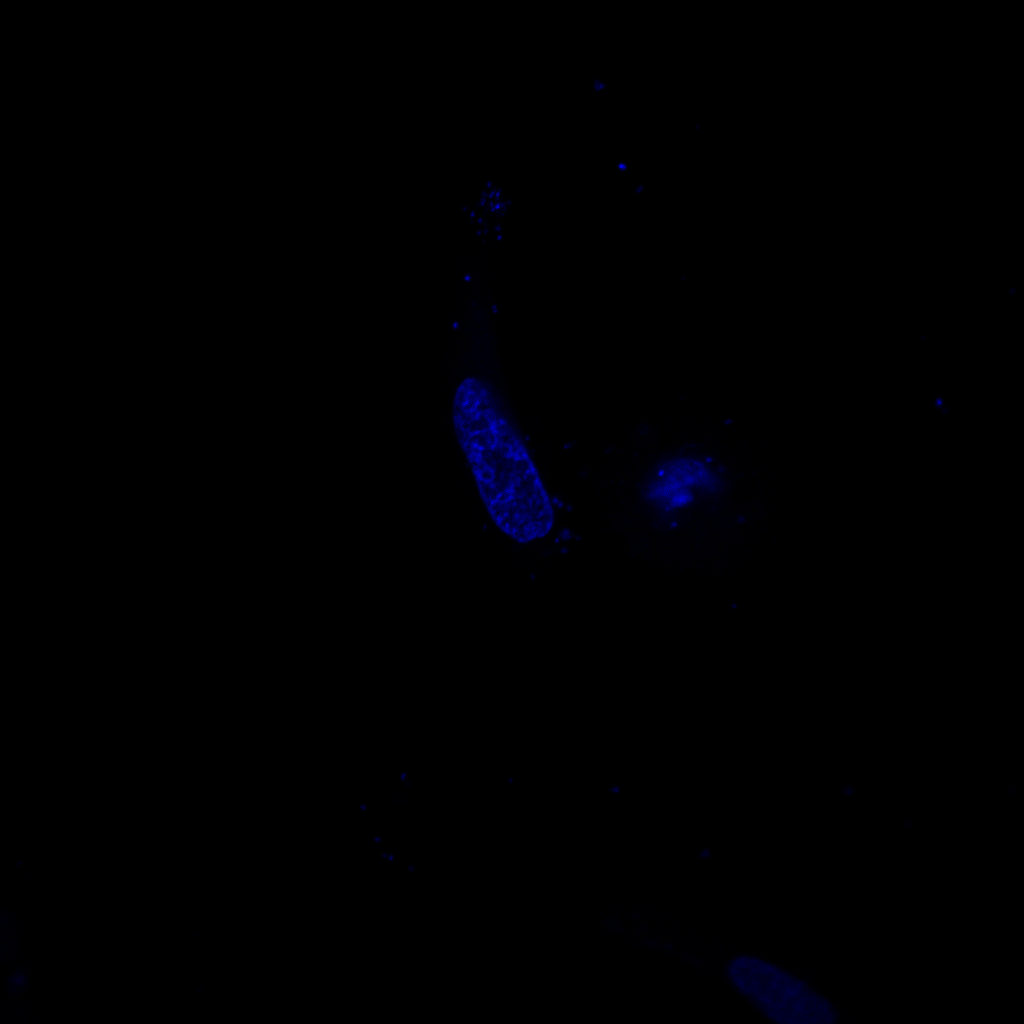

Supplement: Supplementary file 6 — Source data Fig. 4 [file 44319_2024_203_MOESM6_ESM.zip › 4F/RASSF8-delCC_Middle/Sorbitol/U2OS_mchRASSF8-RBD-delC-sorbitol_2mins_2023_07_26__17_49_22_s04z12c1.tif]

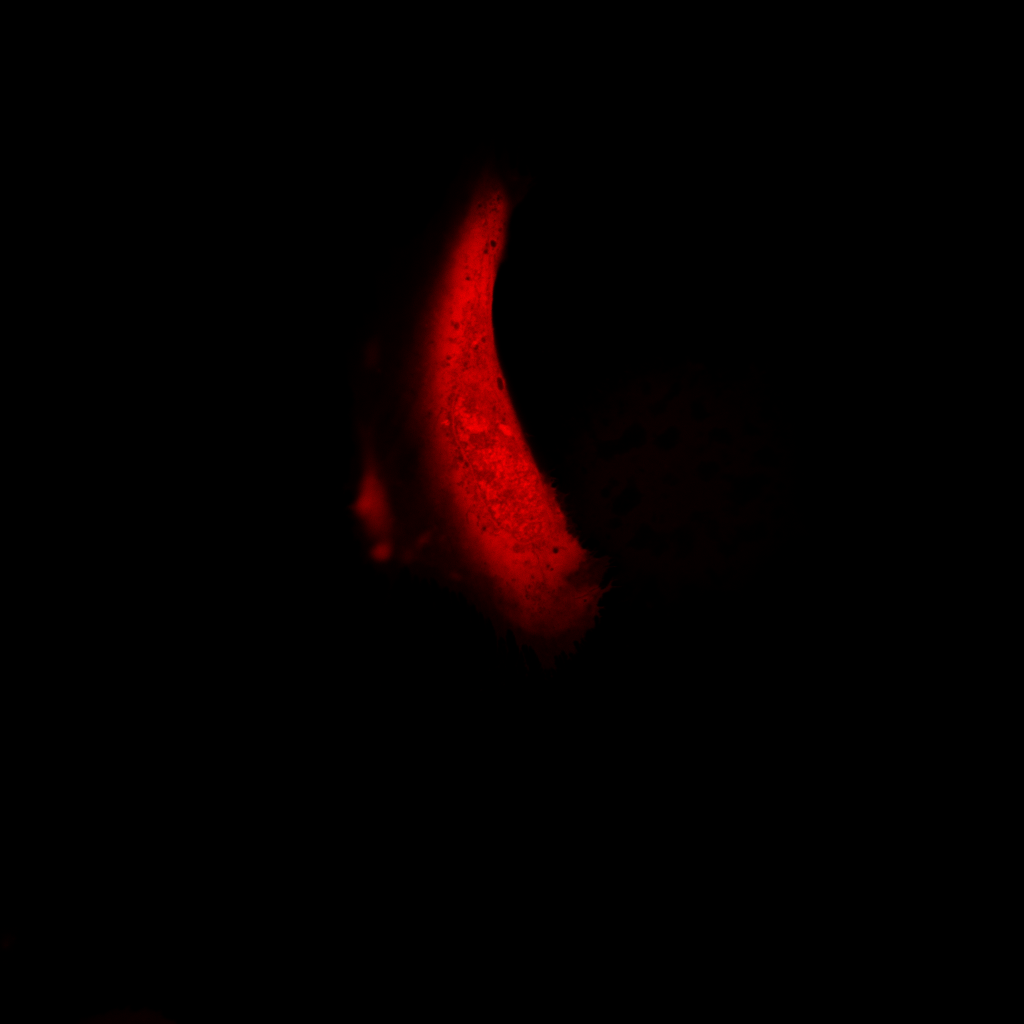

Supplement: Supplementary file 6 — Source data Fig. 4 [file 44319_2024_203_MOESM6_ESM.zip › 4F/RASSF8-delCC_Middle/Sorbitol/U2OS_mchRASSF8-RBD-delC-sorbitol_2mins_2023_07_26__17_49_22_s04z12c2.tif]

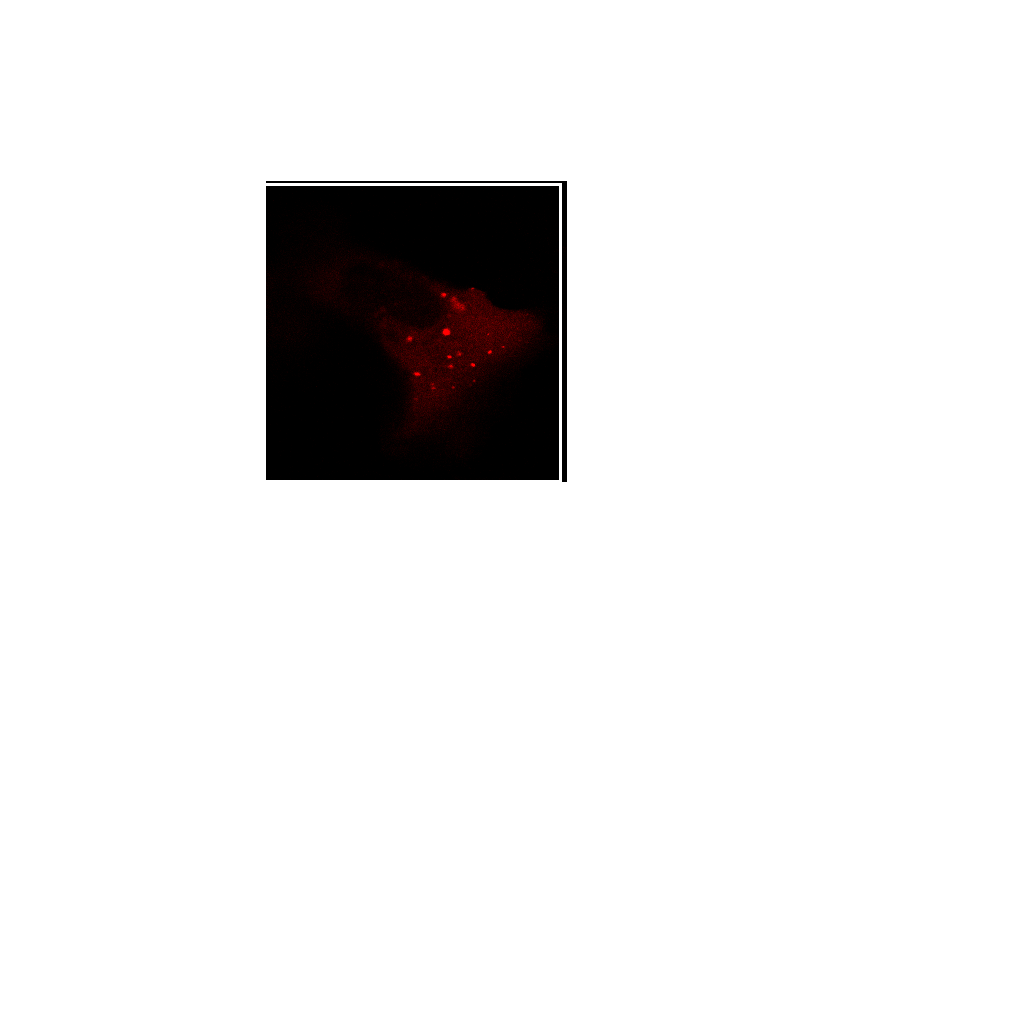

Supplement: Supplementary file 6 — Source data Fig. 4 [file 44319_2024_203_MOESM6_ESM.zip › 4F/RASSF8_Top/After wash 20 mins/mCHRassf8_after20min_zstack16.tif]

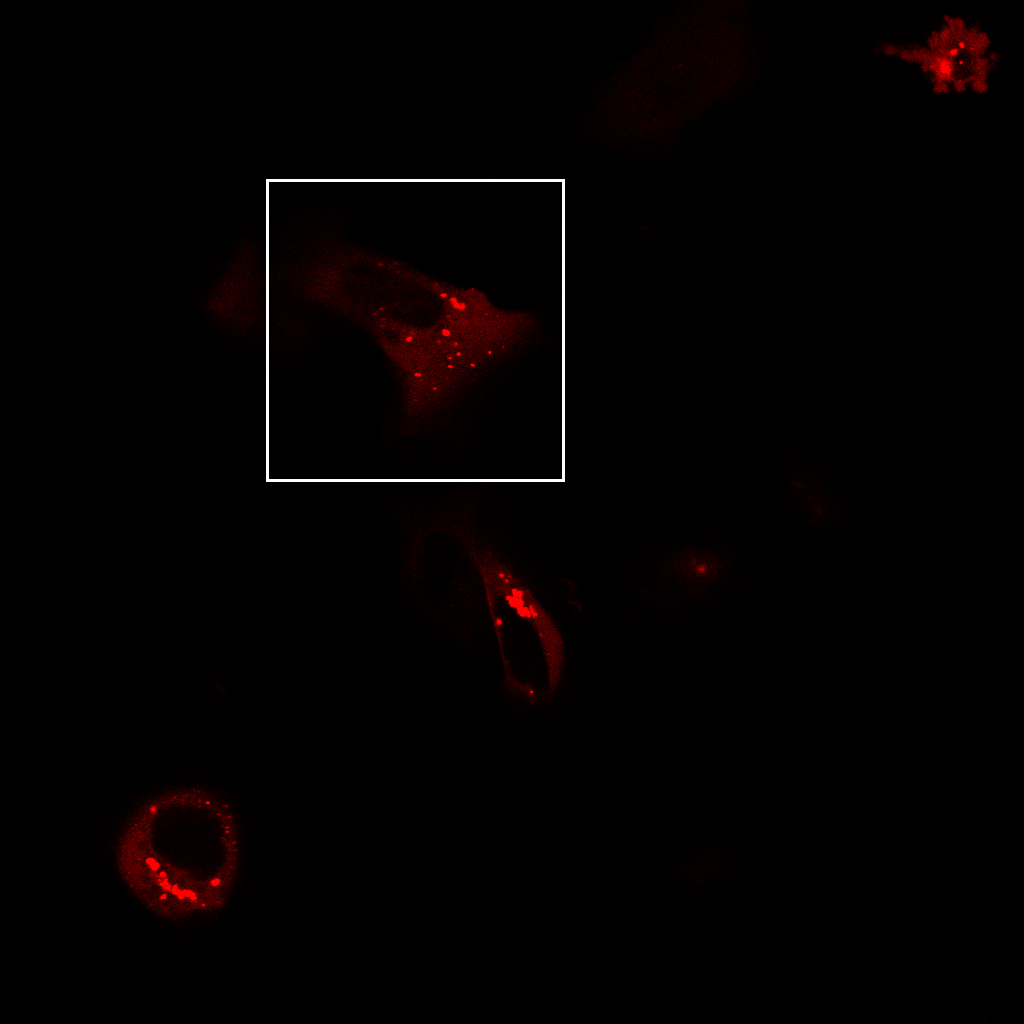

Supplement: Supplementary file 6 — Source data Fig. 4 [file 44319_2024_203_MOESM6_ESM.zip › 4F/RASSF8_Top/After wash 20 mins/mCHRassf8_after20min_zstack17.tif]

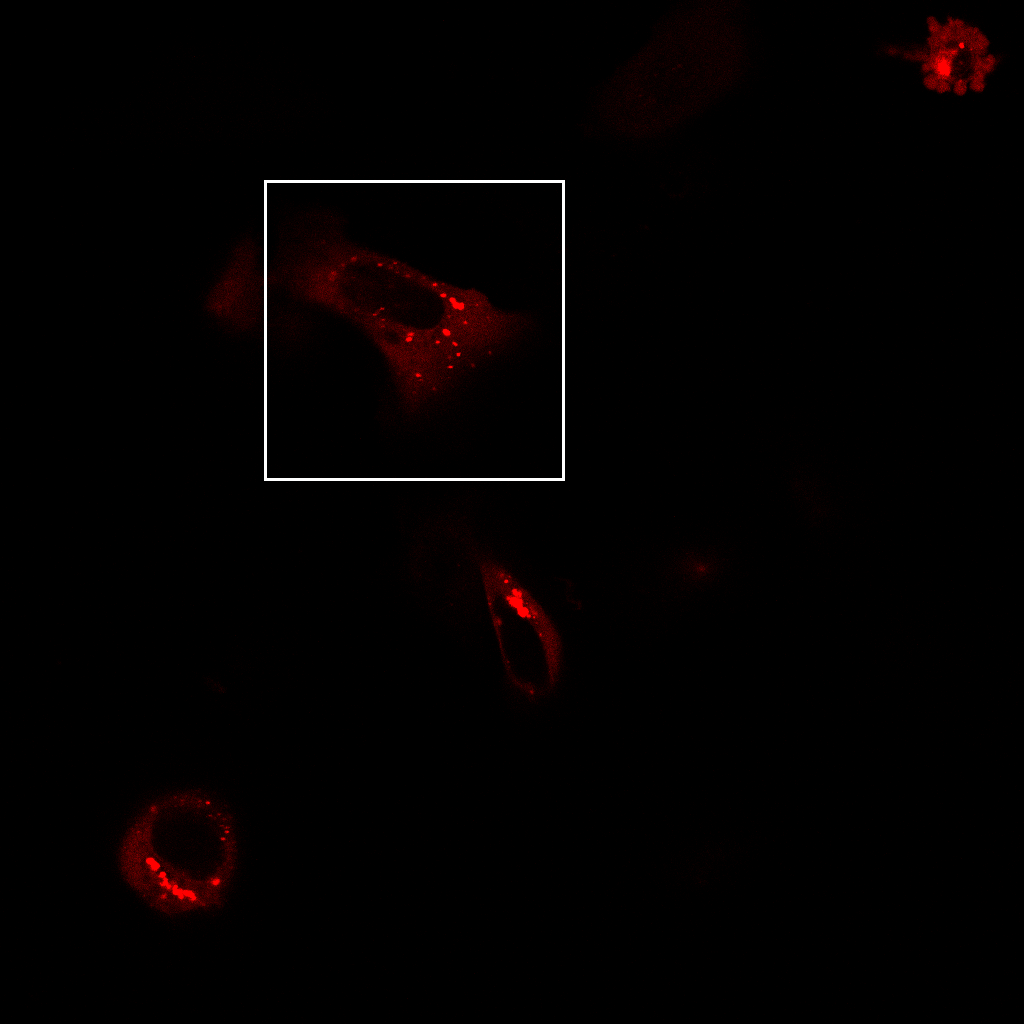

Supplement: Supplementary file 6 — Source data Fig. 4 [file 44319_2024_203_MOESM6_ESM.zip › 4F/RASSF8_Top/After wash 20 mins/mCHRassf8_after20min_zstack18.tif]

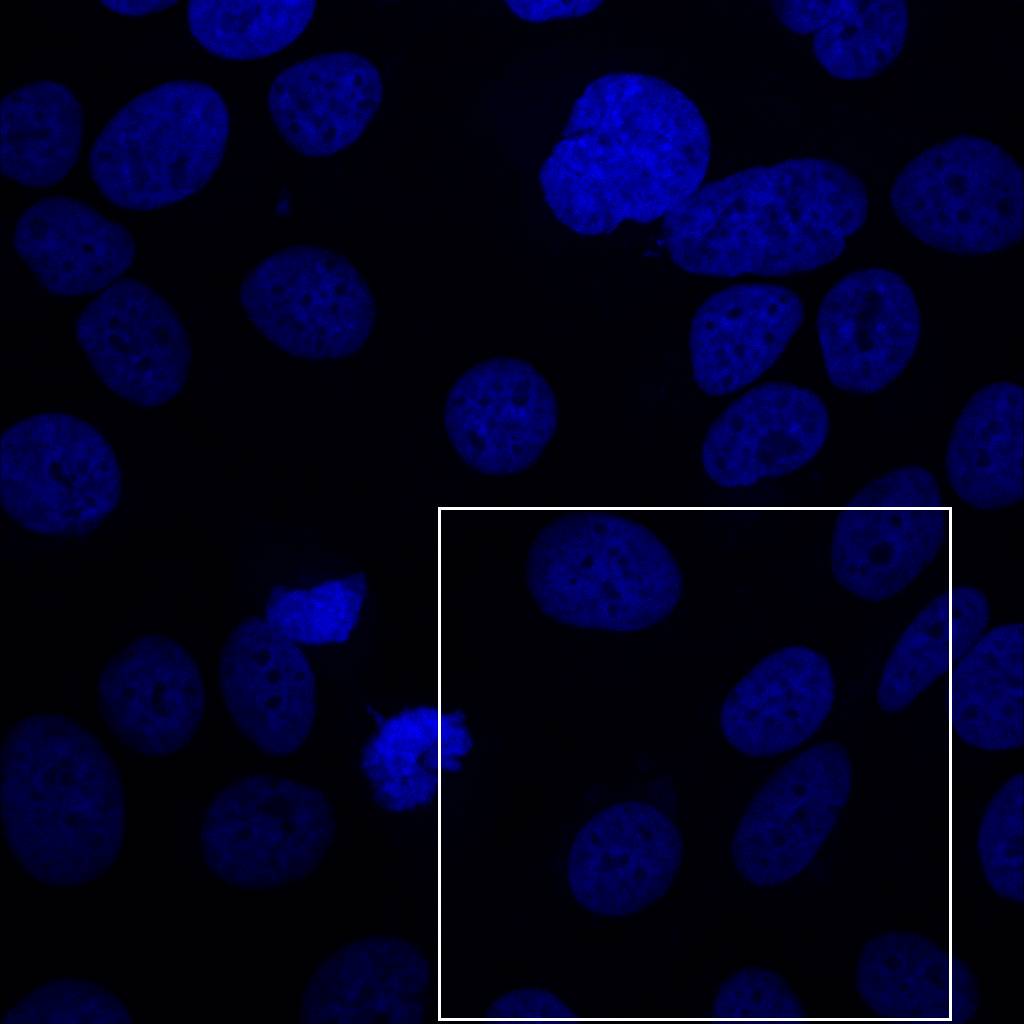

Supplement: Supplementary file 6 — Source data Fig. 4 [file 44319_2024_203_MOESM6_ESM.zip › 4G/Bottom panel_YAP+RASSF8-delCC/Hoechst.tif]

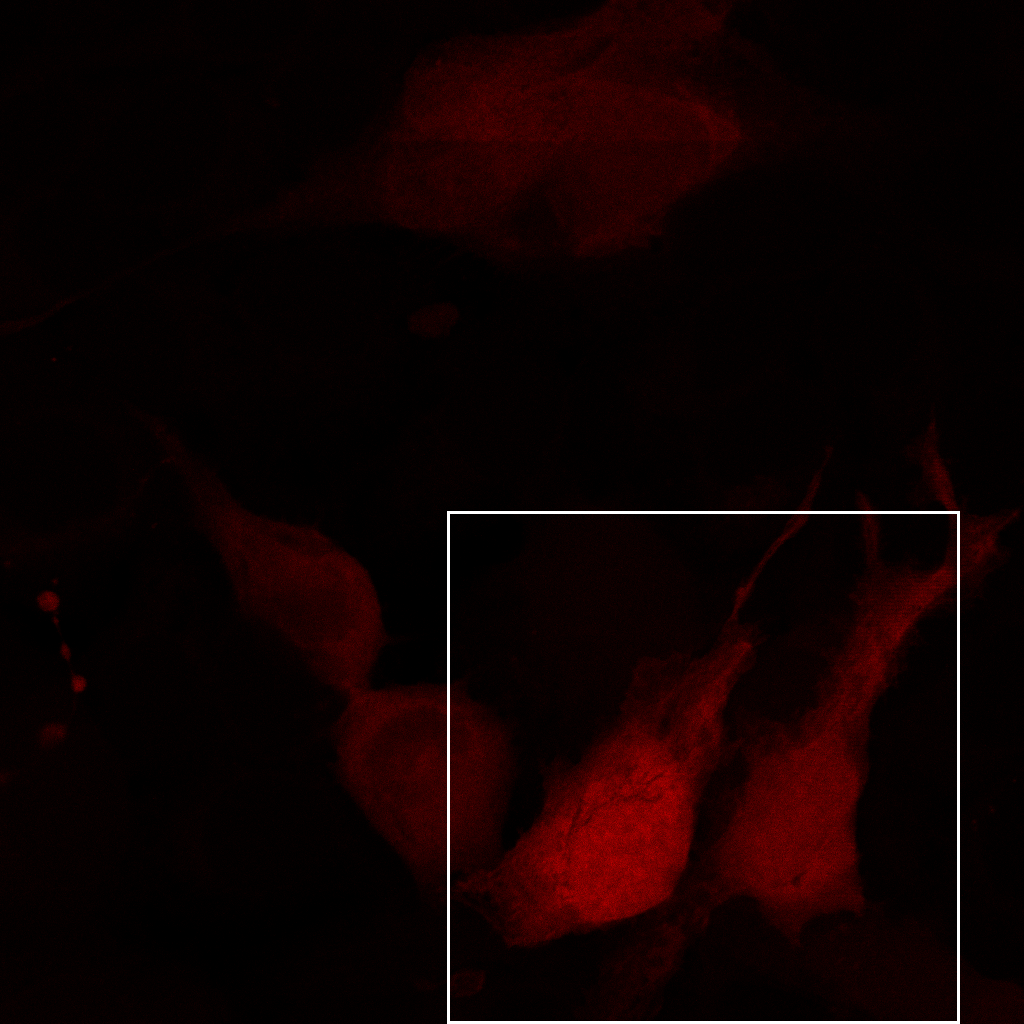

Supplement: Supplementary file 6 — Source data Fig. 4 [file 44319_2024_203_MOESM6_ESM.zip › 4G/Bottom panel_YAP+RASSF8-delCC/Rassf8-delCC.tif]

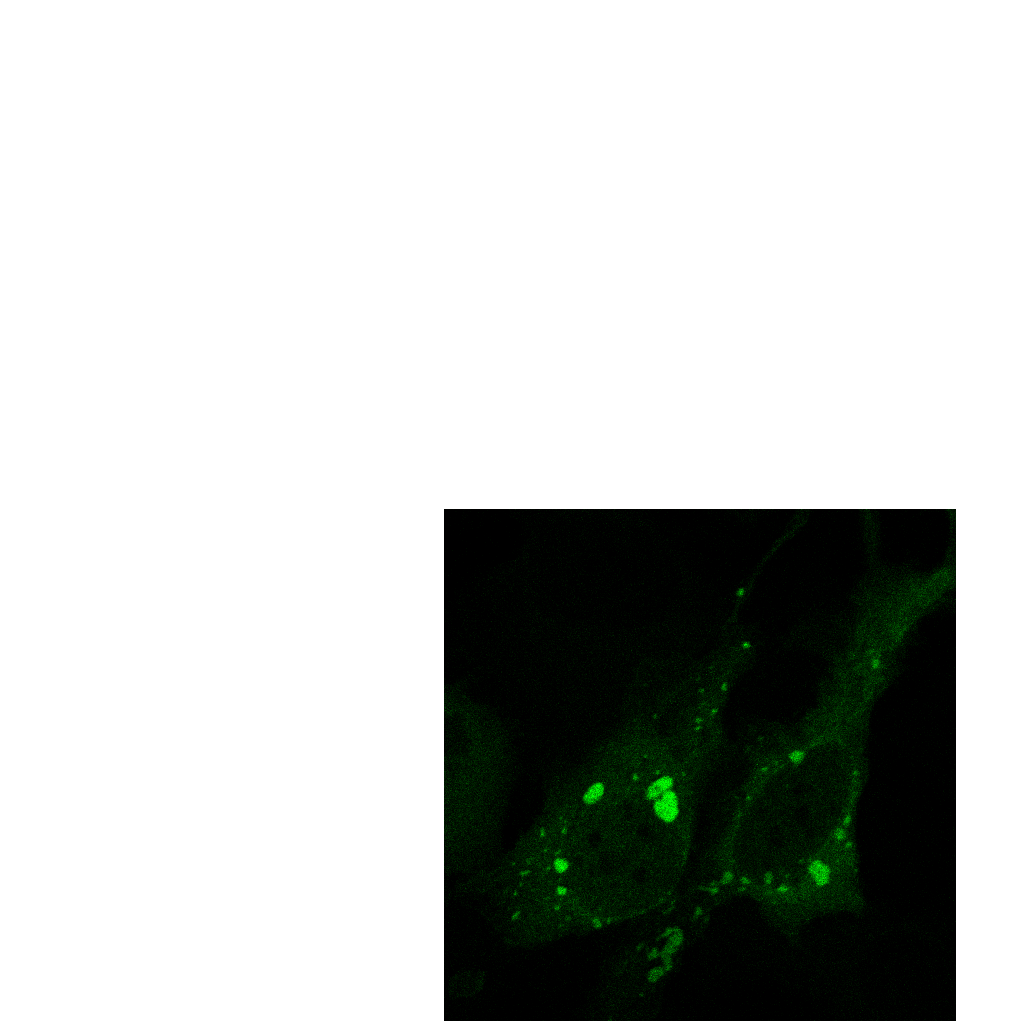

Supplement: Supplementary file 6 — Source data Fig. 4 [file 44319_2024_203_MOESM6_ESM.zip › 4G/Bottom panel_YAP+RASSF8-delCC/YAP.tif]

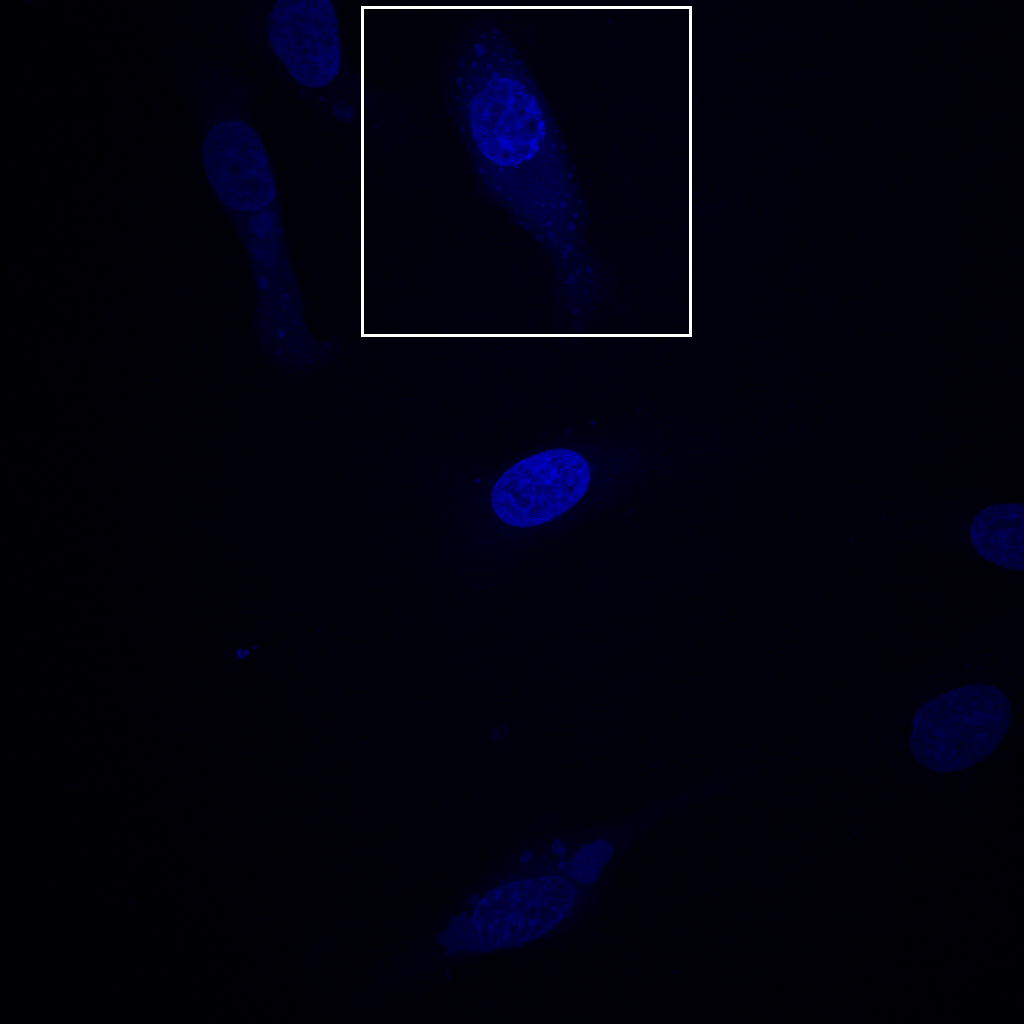

Supplement: Supplementary file 6 — Source data Fig. 4 [file 44319_2024_203_MOESM6_ESM.zip › 4G/Middle panel_YAP+RASSF8/Hoechst.tif]

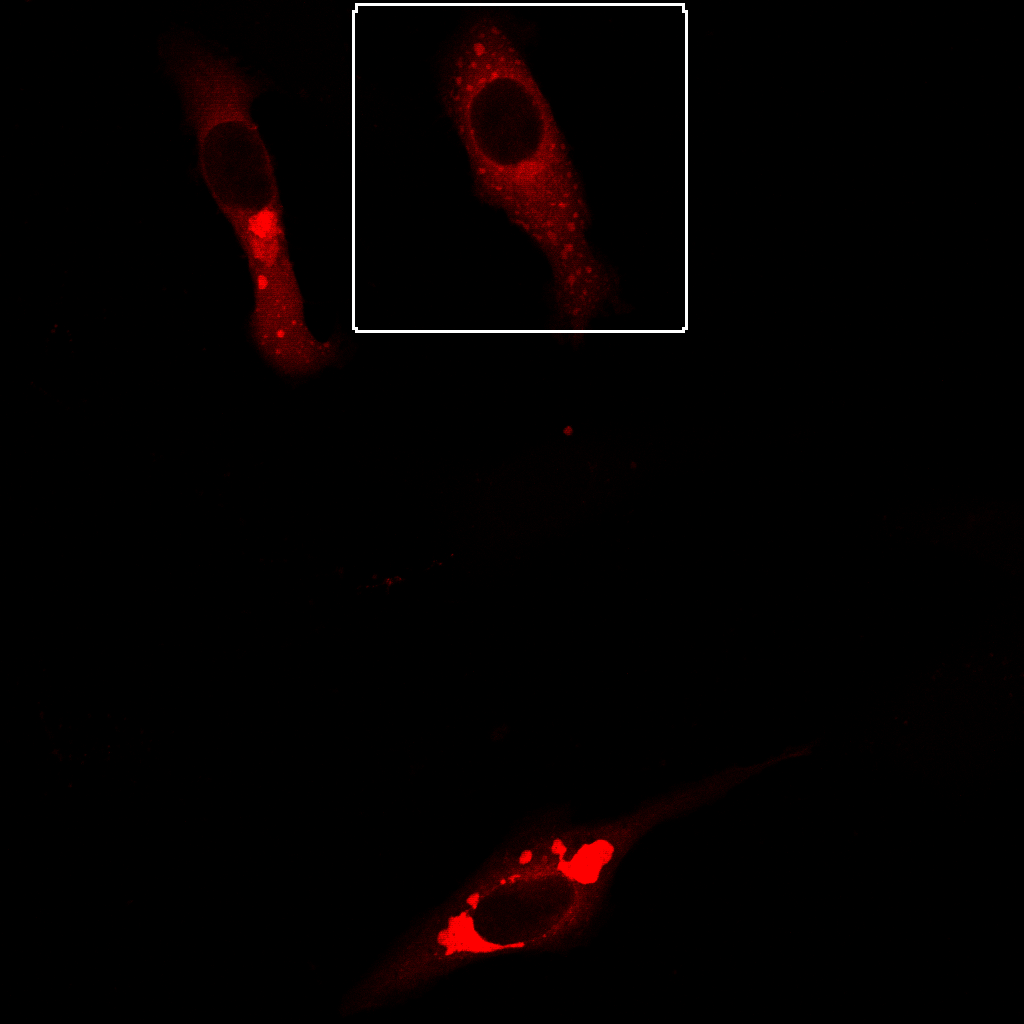

Supplement: Supplementary file 6 — Source data Fig. 4 [file 44319_2024_203_MOESM6_ESM.zip › 4G/Middle panel_YAP+RASSF8/RASSF8.tif]

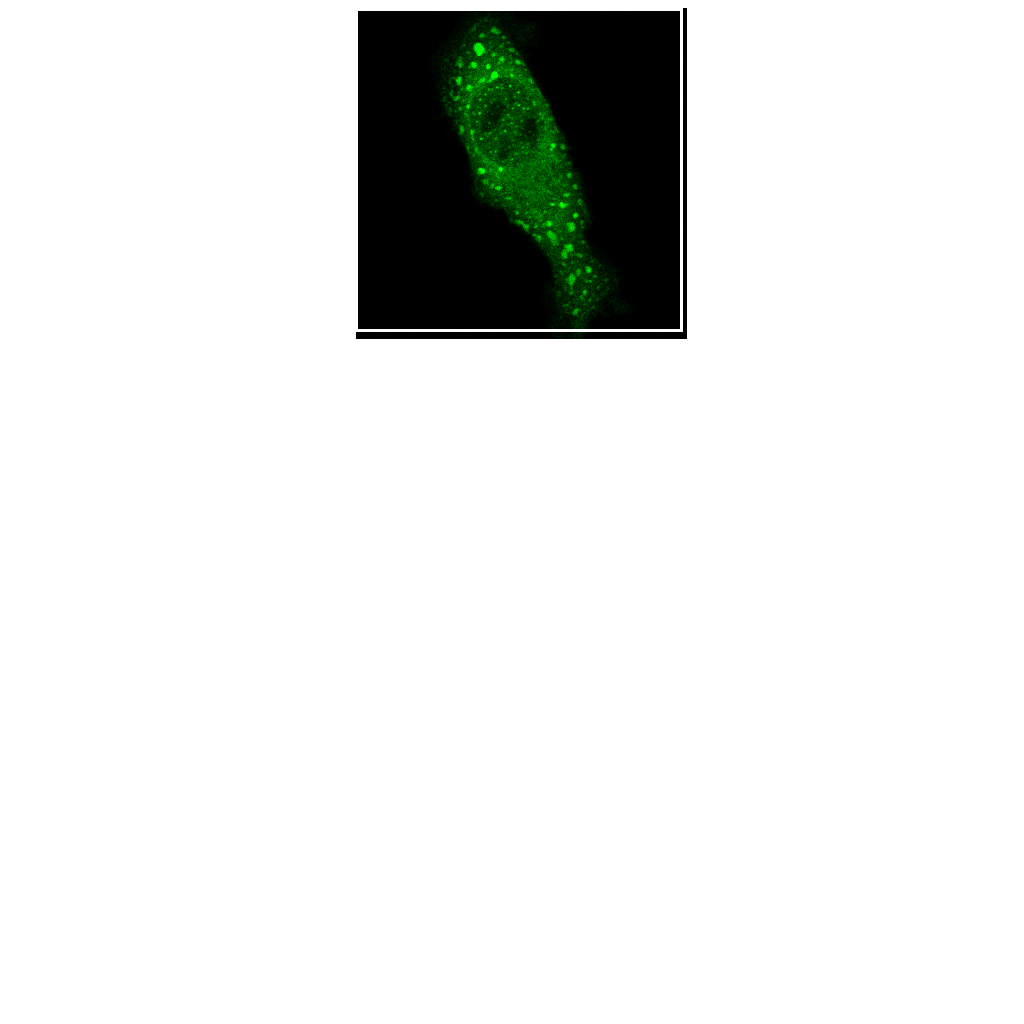

Supplement: Supplementary file 6 — Source data Fig. 4 [file 44319_2024_203_MOESM6_ESM.zip › 4G/Middle panel_YAP+RASSF8/YAP.tif]

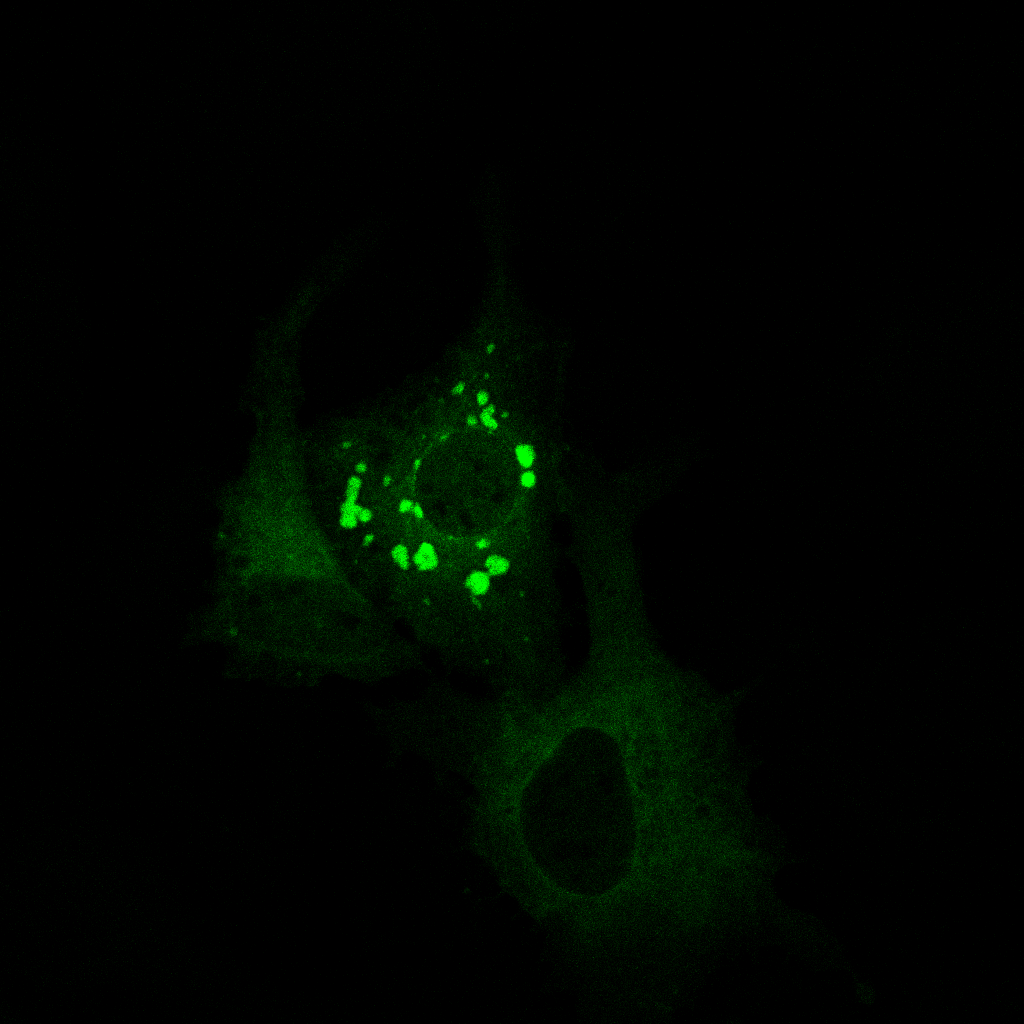

Supplement: Supplementary file 6 — Source data Fig. 4 [file 44319_2024_203_MOESM6_ESM.zip › 4G/Top panel_YAP+mCherry/YAP.tif]

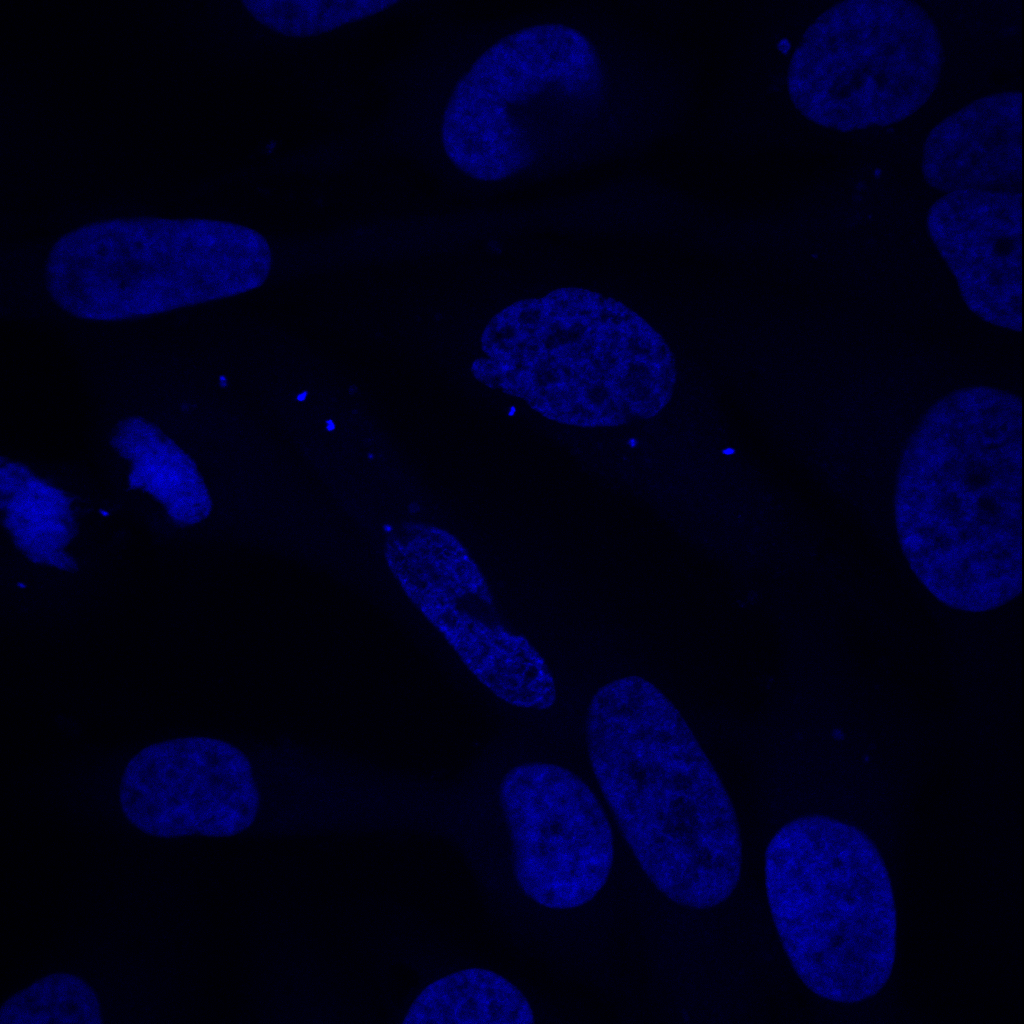

Supplement: Supplementary file 7 — Source data Fig. 5 [file 44319_2024_203_MOESM7_ESM.zip › 5B/5. RHOBTB1 WT+ RASSF8/Hoechst.tif]

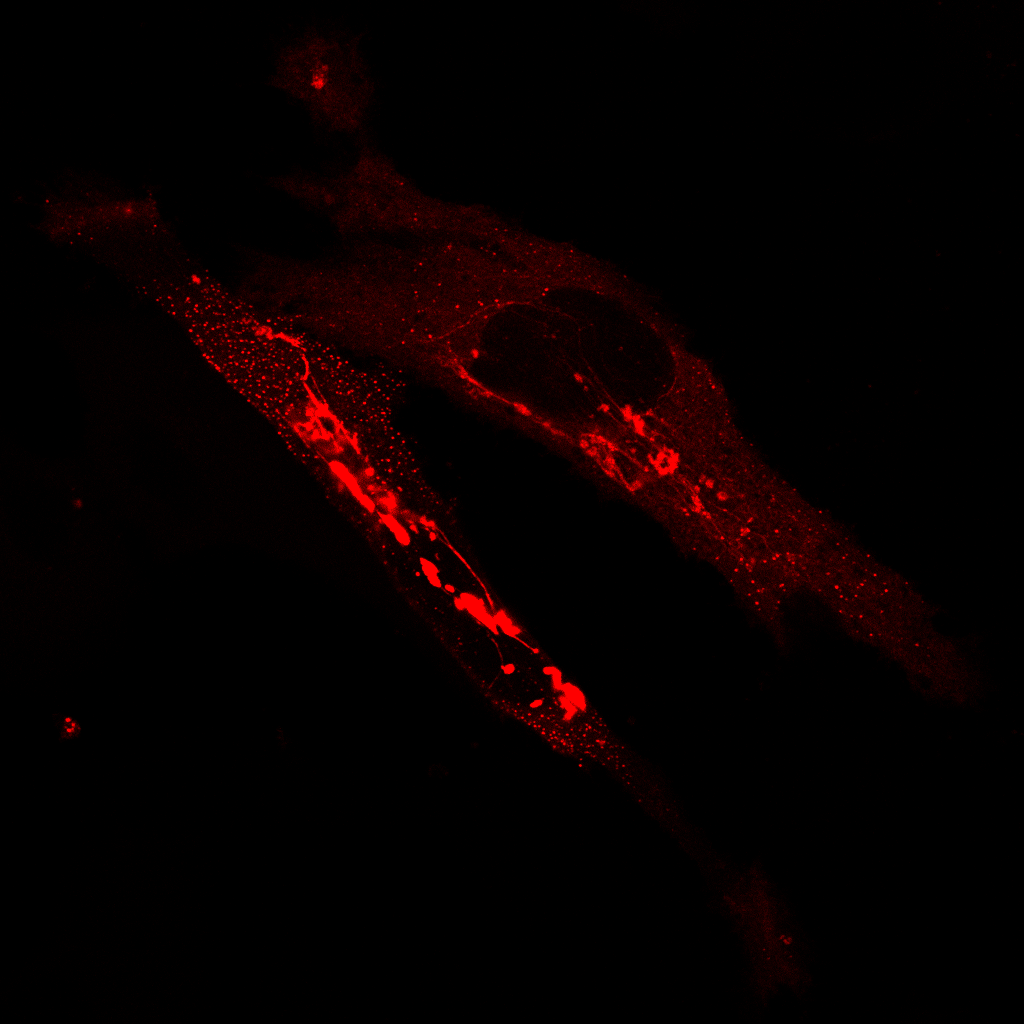

Supplement: Supplementary file 7 — Source data Fig. 5 [file 44319_2024_203_MOESM7_ESM.zip › 5B/5. RHOBTB1 WT+ RASSF8/RASSF8.tif]

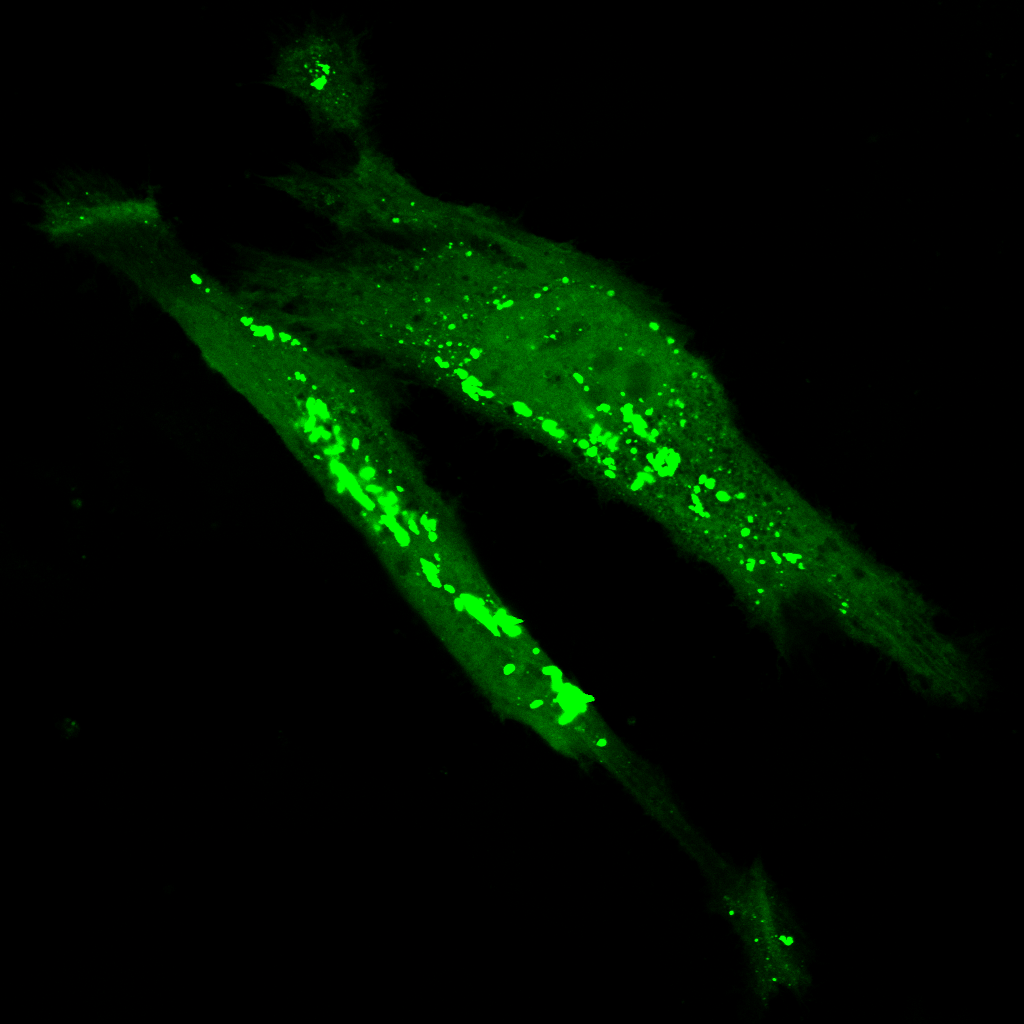

Supplement: Supplementary file 7 — Source data Fig. 5 [file 44319_2024_203_MOESM7_ESM.zip › 5B/5. RHOBTB1 WT+ RASSF8/RHOBTB1.tif]

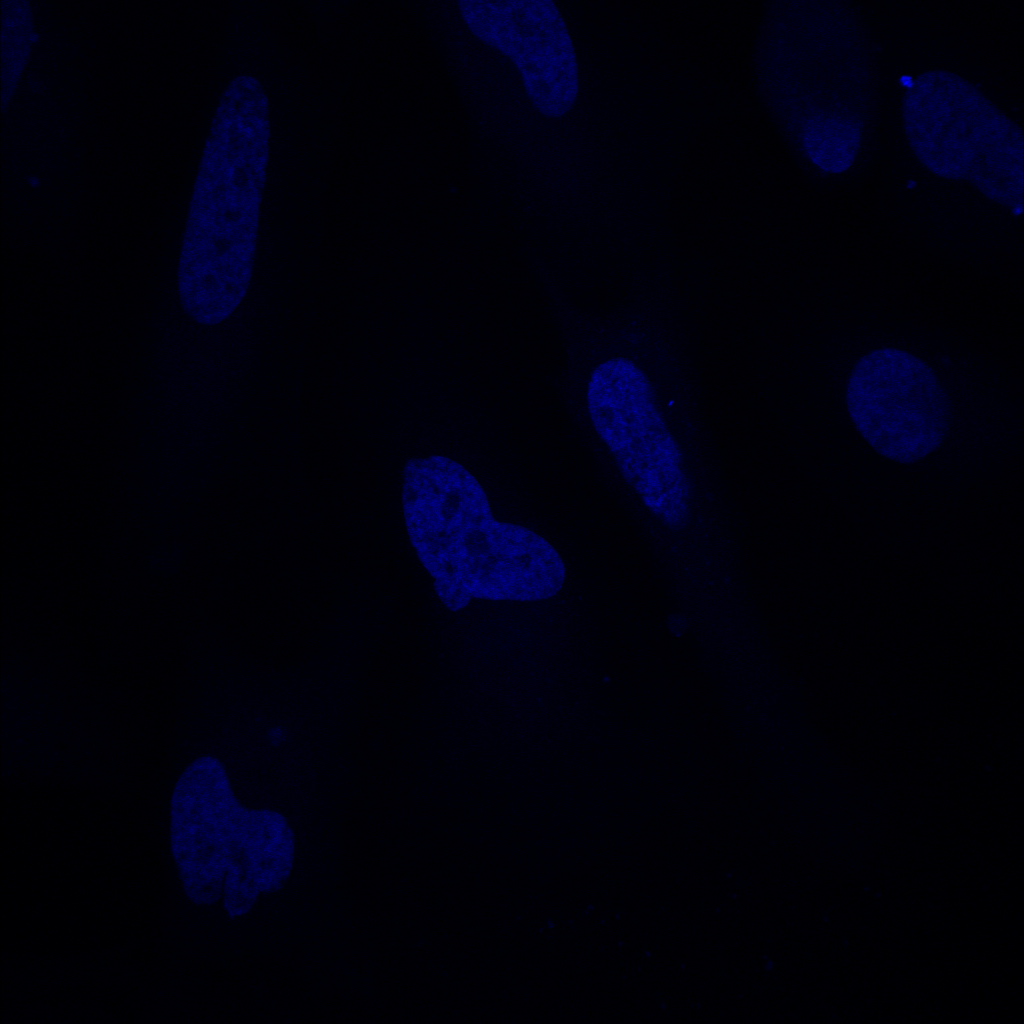

Supplement: Supplementary file 7 — Source data Fig. 5 [file 44319_2024_203_MOESM7_ESM.zip › 5B/6. RHOBTB2 WT+ RASSF8/Hoechst.tif]

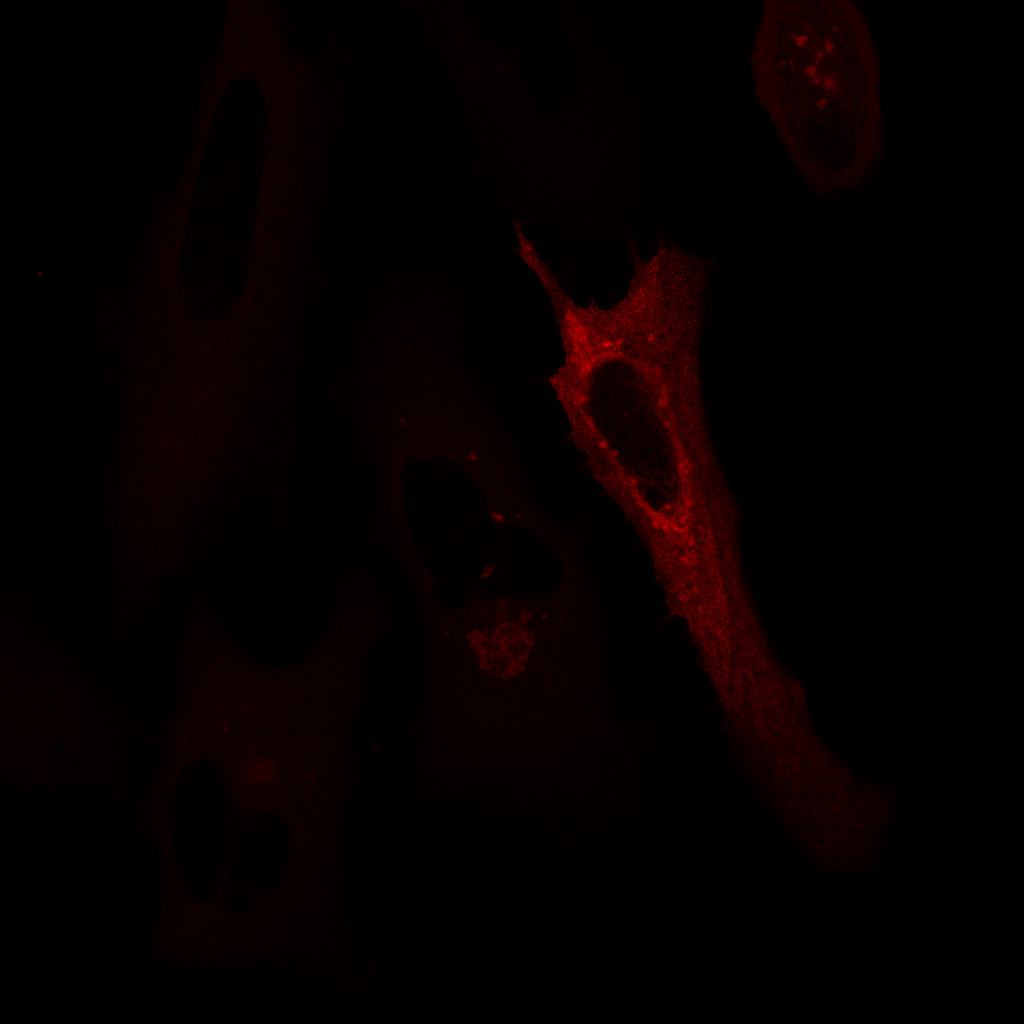

Supplement: Supplementary file 7 — Source data Fig. 5 [file 44319_2024_203_MOESM7_ESM.zip › 5B/6. RHOBTB2 WT+ RASSF8/RASSF8.tif]

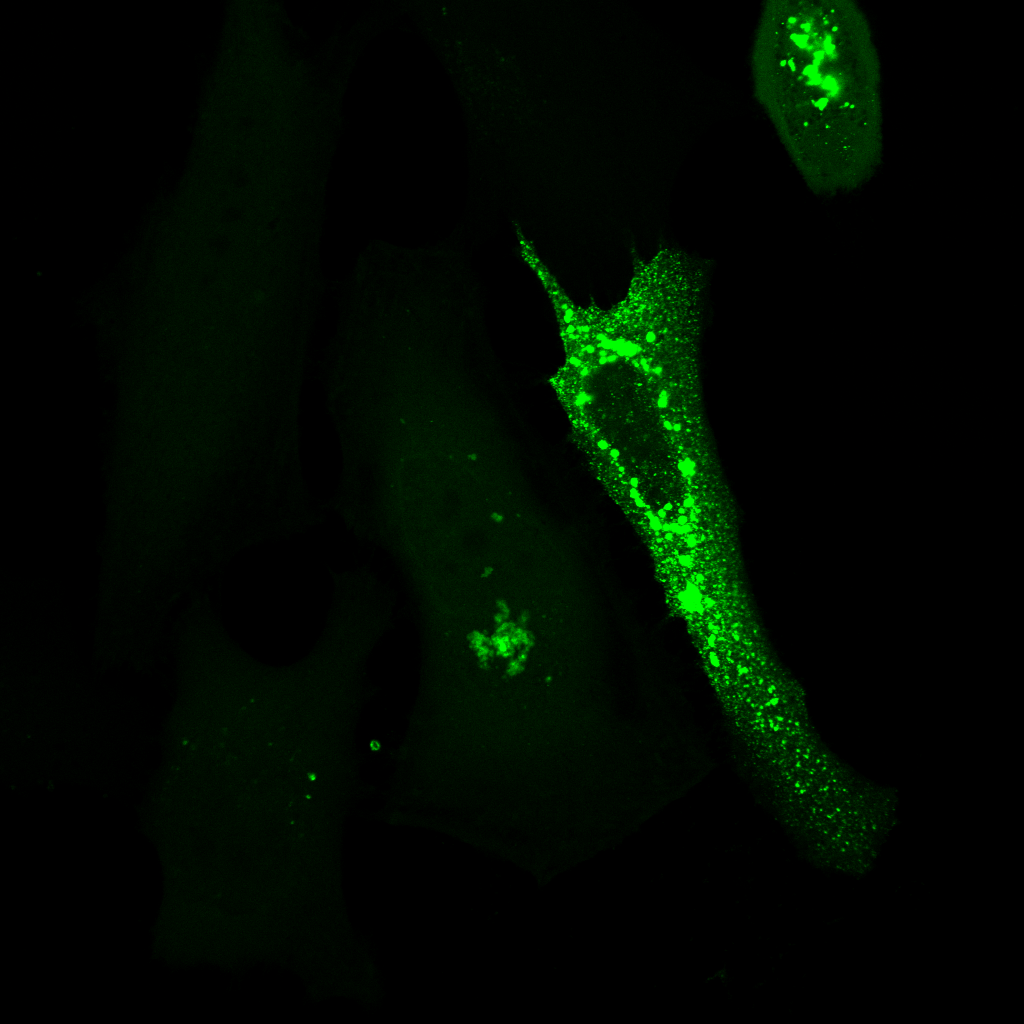

Supplement: Supplementary file 7 — Source data Fig. 5 [file 44319_2024_203_MOESM7_ESM.zip › 5B/6. RHOBTB2 WT+ RASSF8/RHOBTB2.tif]

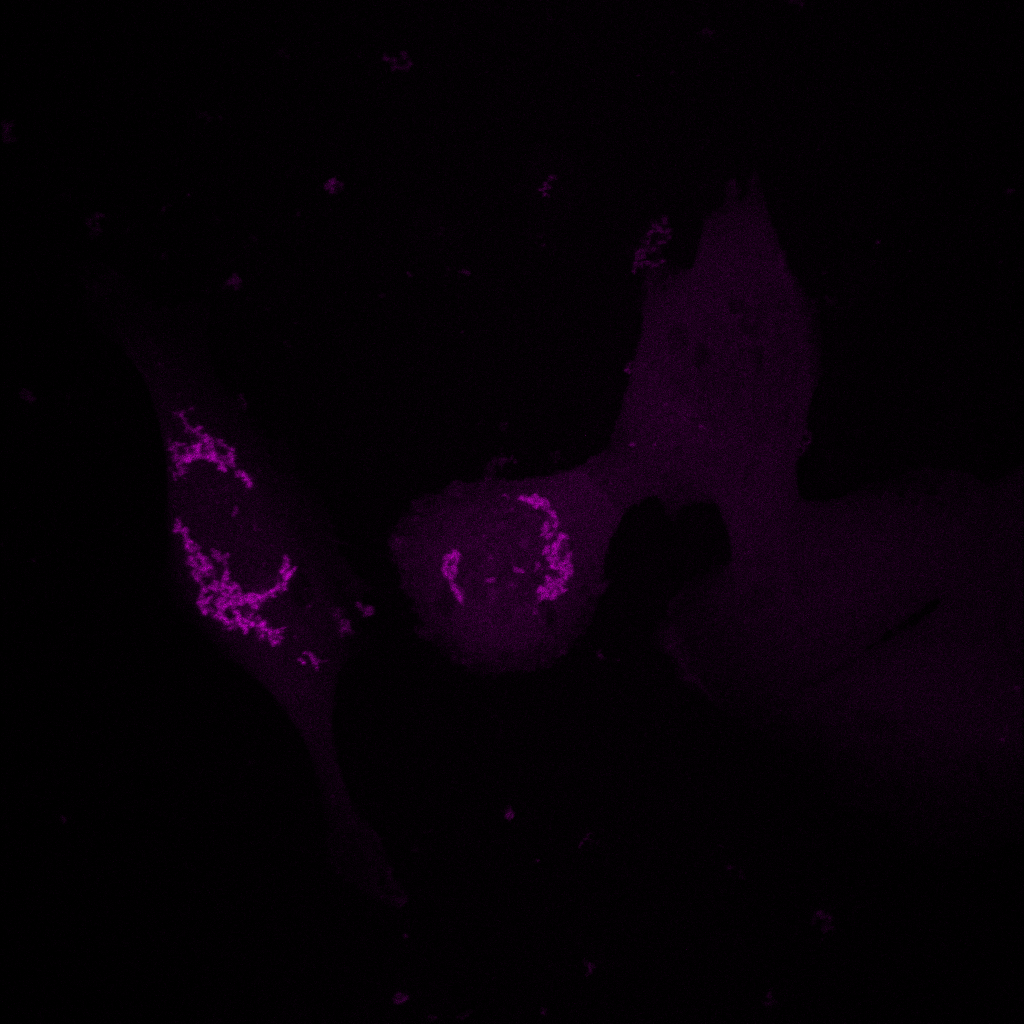

Supplement: Supplementary file 7 — Source data Fig. 5 [file 44319_2024_203_MOESM7_ESM.zip › 5C/1. RASD1+RASSF8+YAP/RASD1.tif]

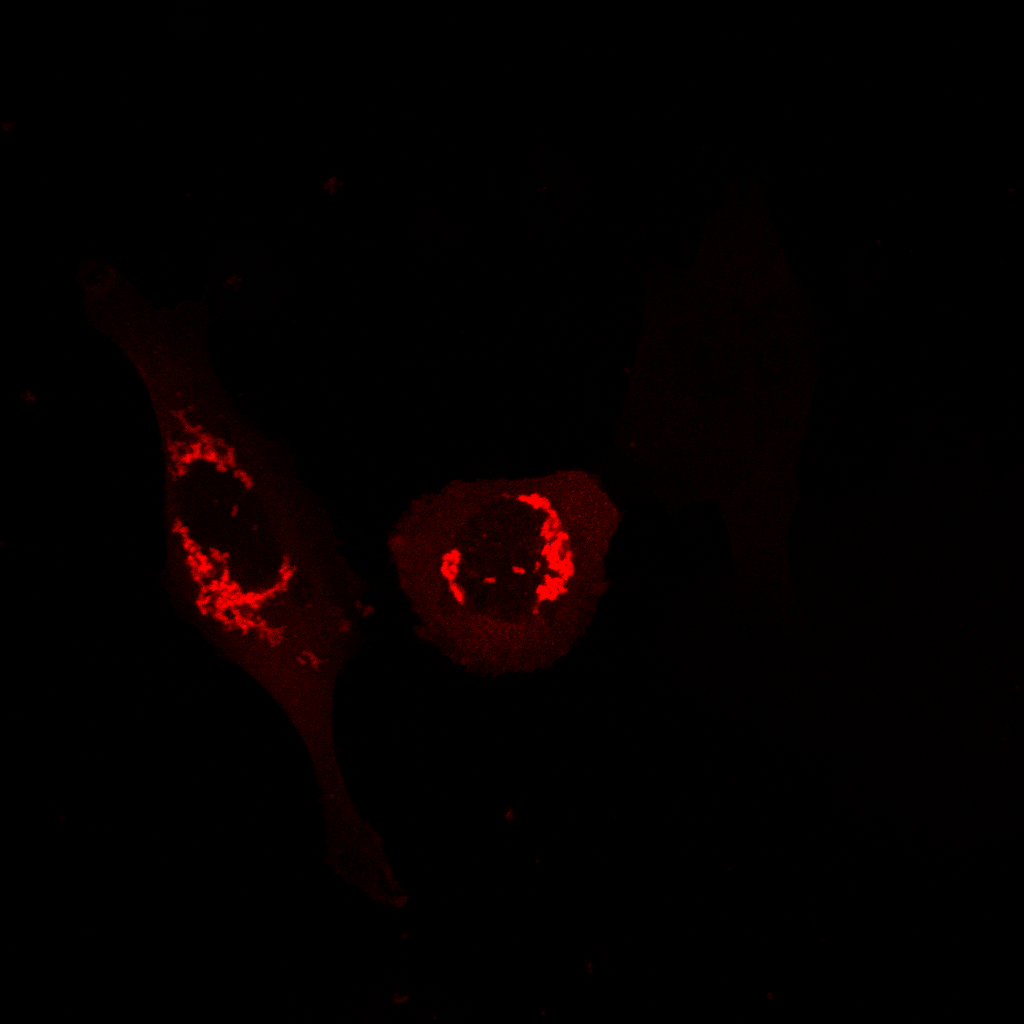

Supplement: Supplementary file 7 — Source data Fig. 5 [file 44319_2024_203_MOESM7_ESM.zip › 5C/1. RASD1+RASSF8+YAP/RASSF8.tif]

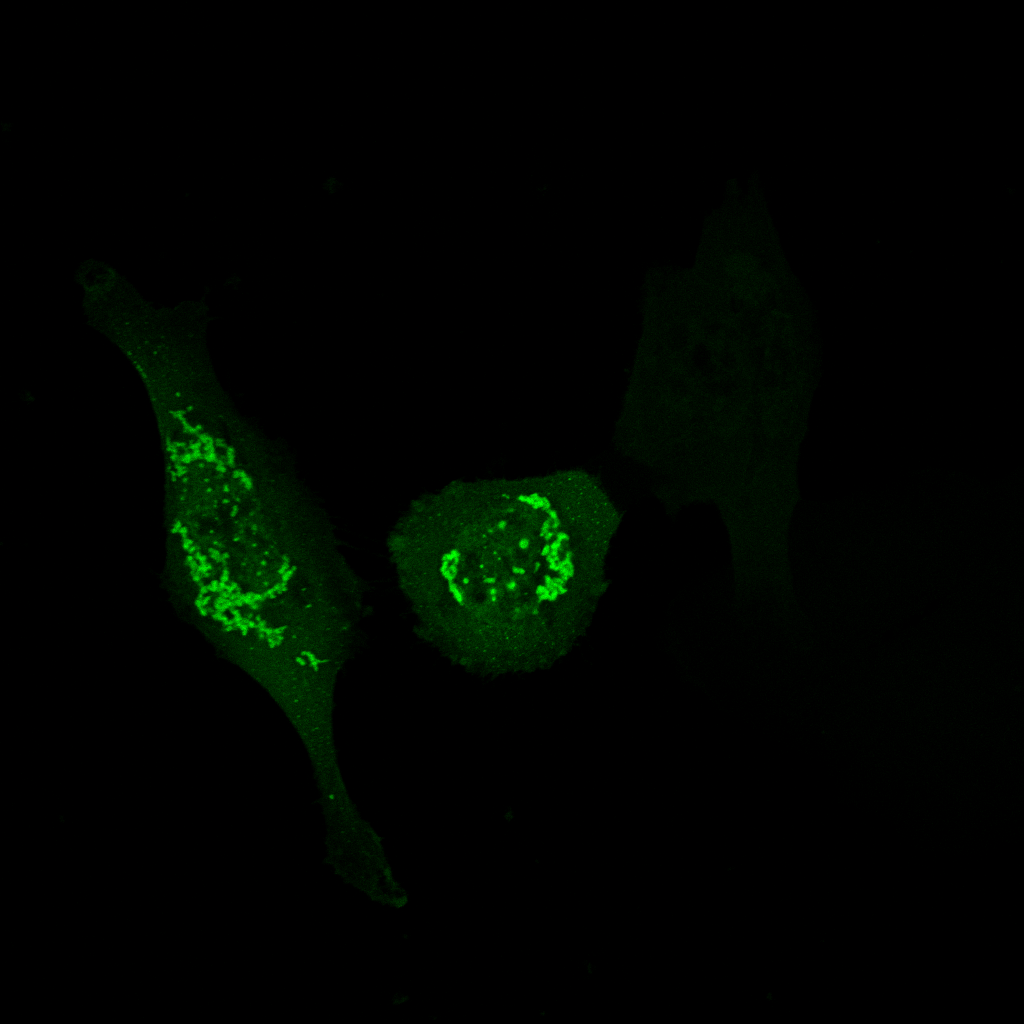

Supplement: Supplementary file 7 — Source data Fig. 5 [file 44319_2024_203_MOESM7_ESM.zip › 5C/1. RASD1+RASSF8+YAP/YAP.tif]

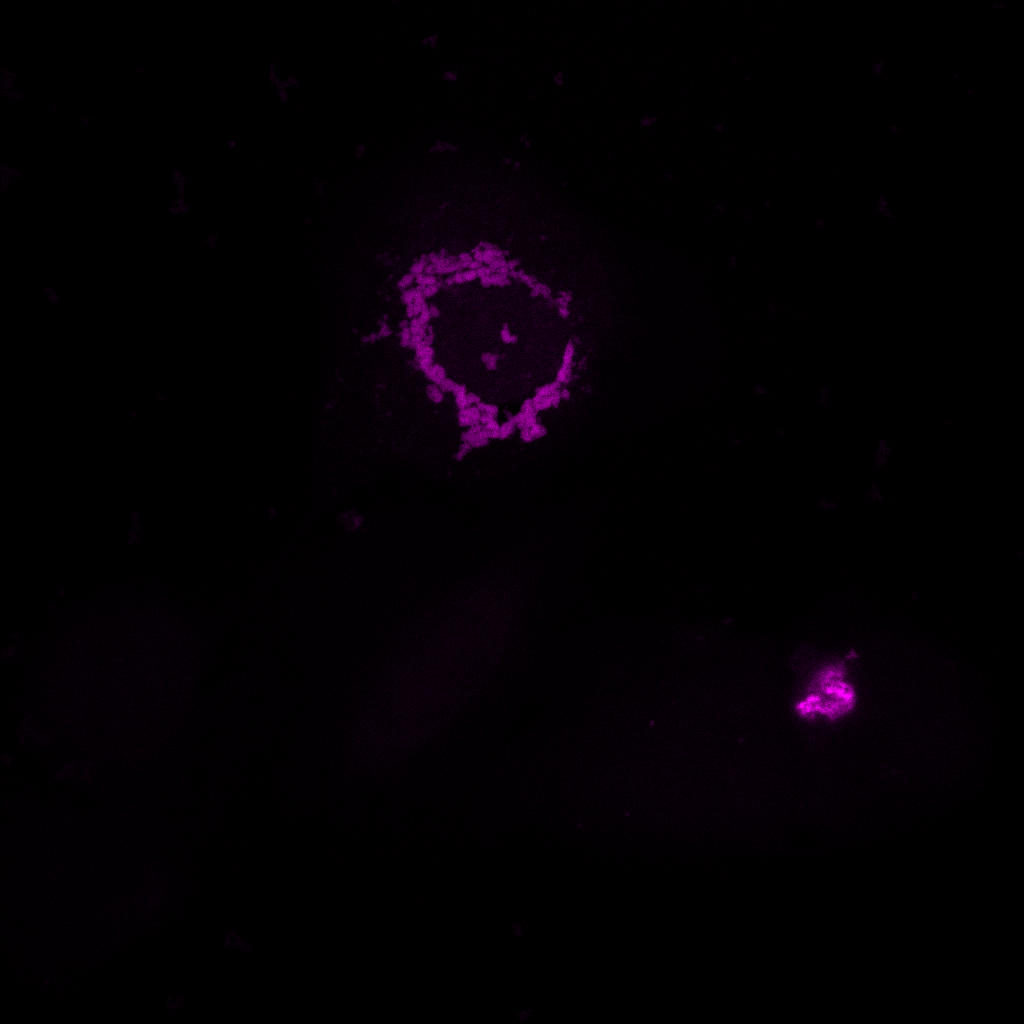

Supplement: Supplementary file 7 — Source data Fig. 5 [file 44319_2024_203_MOESM7_ESM.zip › 5C/2. RASL11B+RASSF8+YAP/RASL11B.tif]

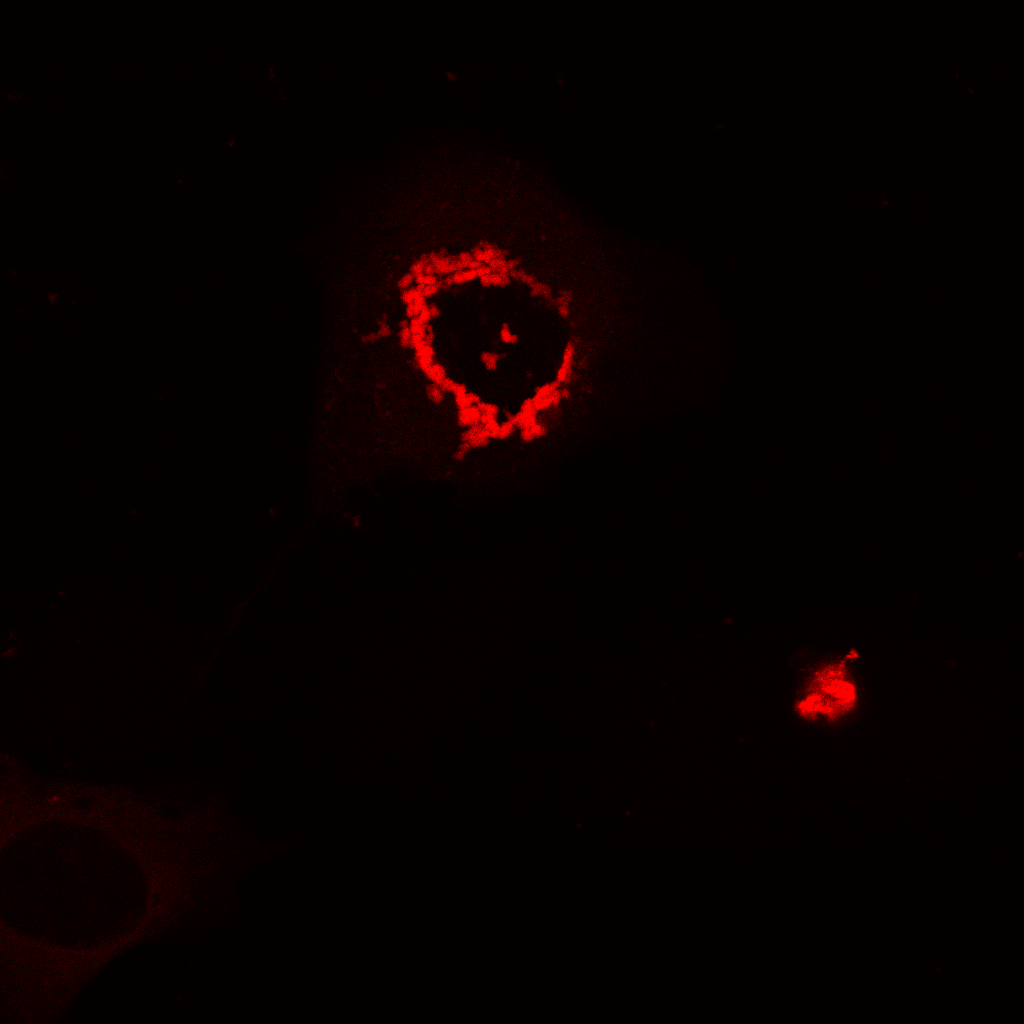

Supplement: Supplementary file 7 — Source data Fig. 5 [file 44319_2024_203_MOESM7_ESM.zip › 5C/2. RASL11B+RASSF8+YAP/RASSF8.tif]

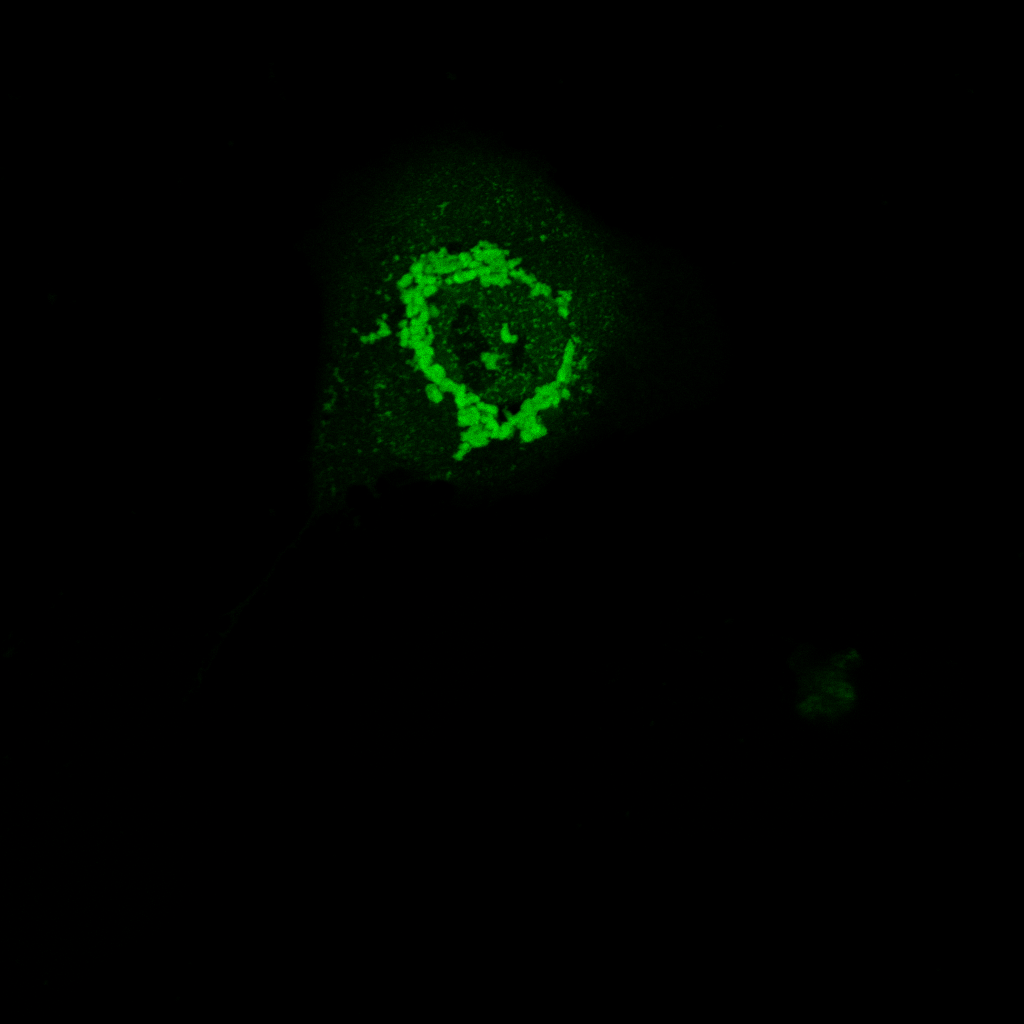

Supplement: Supplementary file 7 — Source data Fig. 5 [file 44319_2024_203_MOESM7_ESM.zip › 5C/2. RASL11B+RASSF8+YAP/YAP.tif]

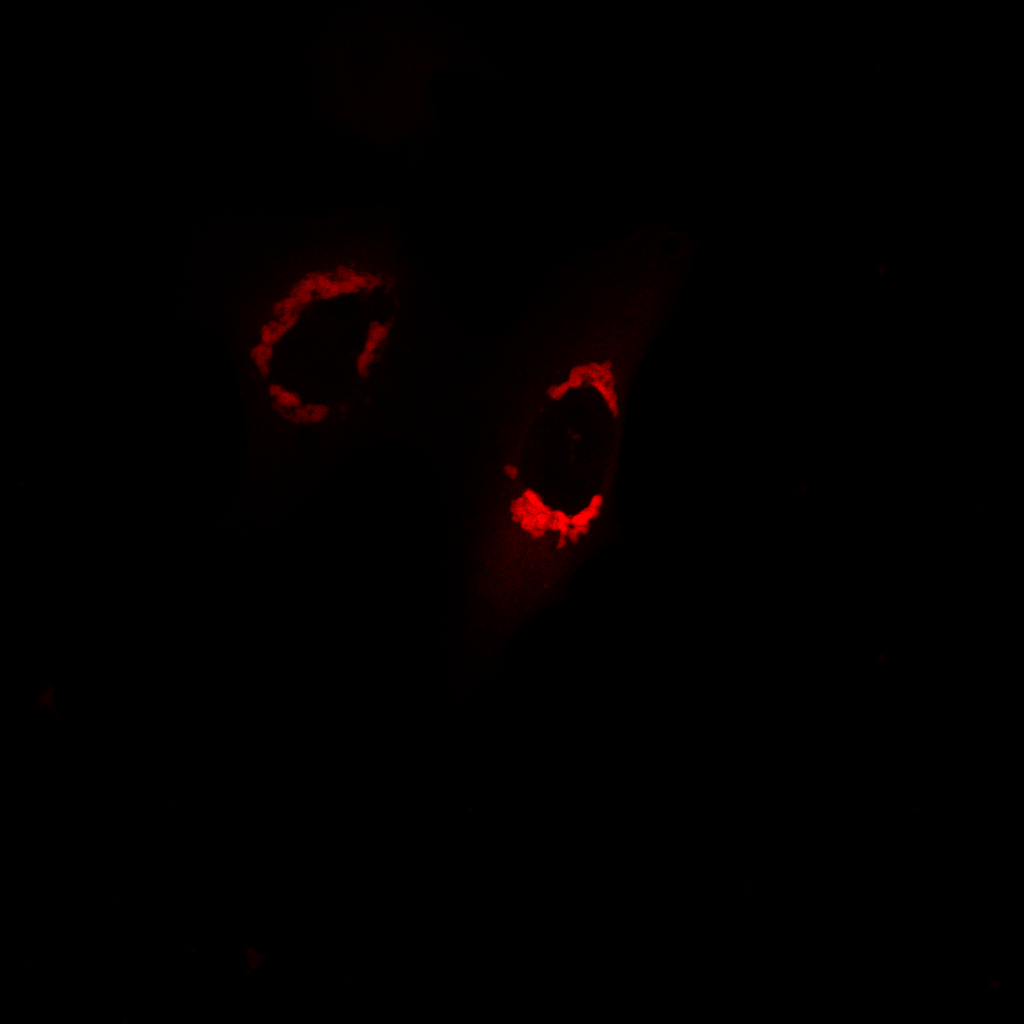

Supplement: Supplementary file 7 — Source data Fig. 5 [file 44319_2024_203_MOESM7_ESM.zip › 5C/3. RHOBTB1+RASSF8+YAP/RASSF8.tif]

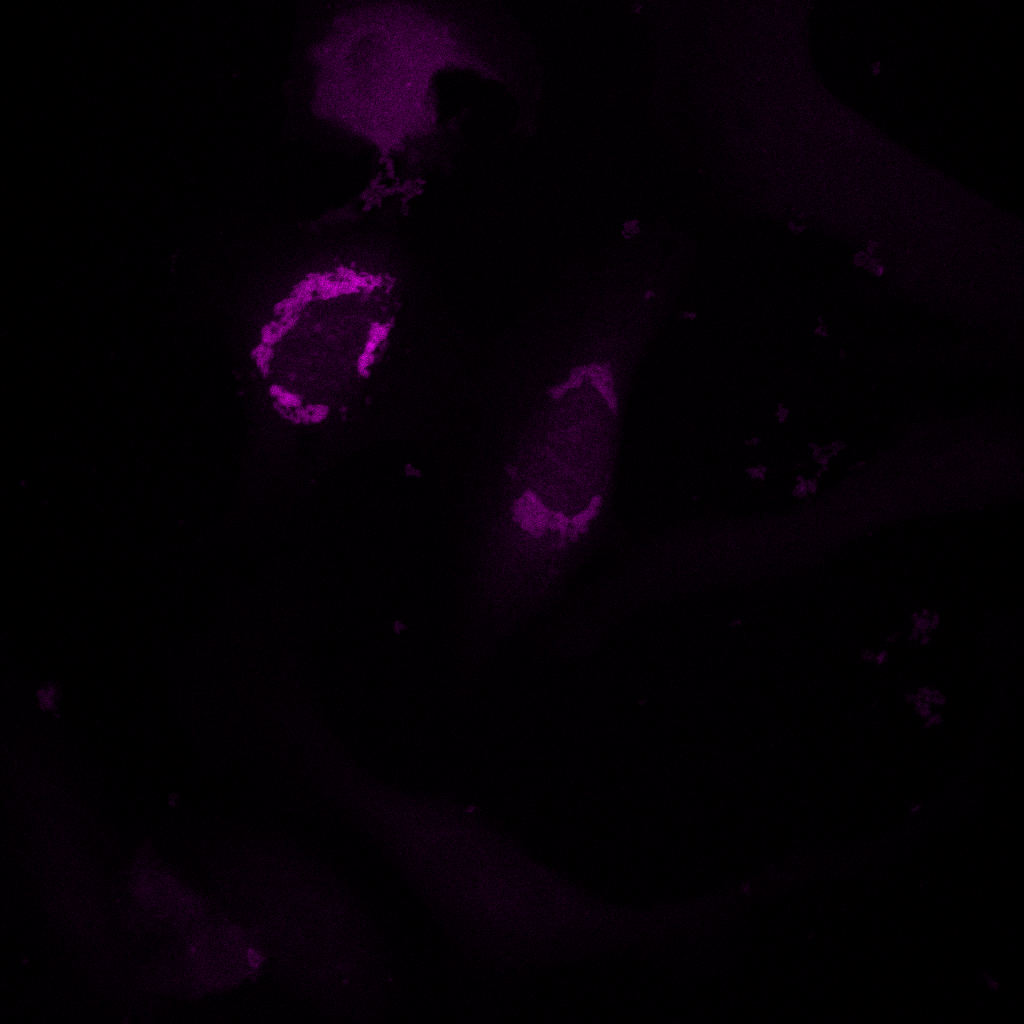

Supplement: Supplementary file 7 — Source data Fig. 5 [file 44319_2024_203_MOESM7_ESM.zip › 5C/3. RHOBTB1+RASSF8+YAP/RHOBTB1.tif]

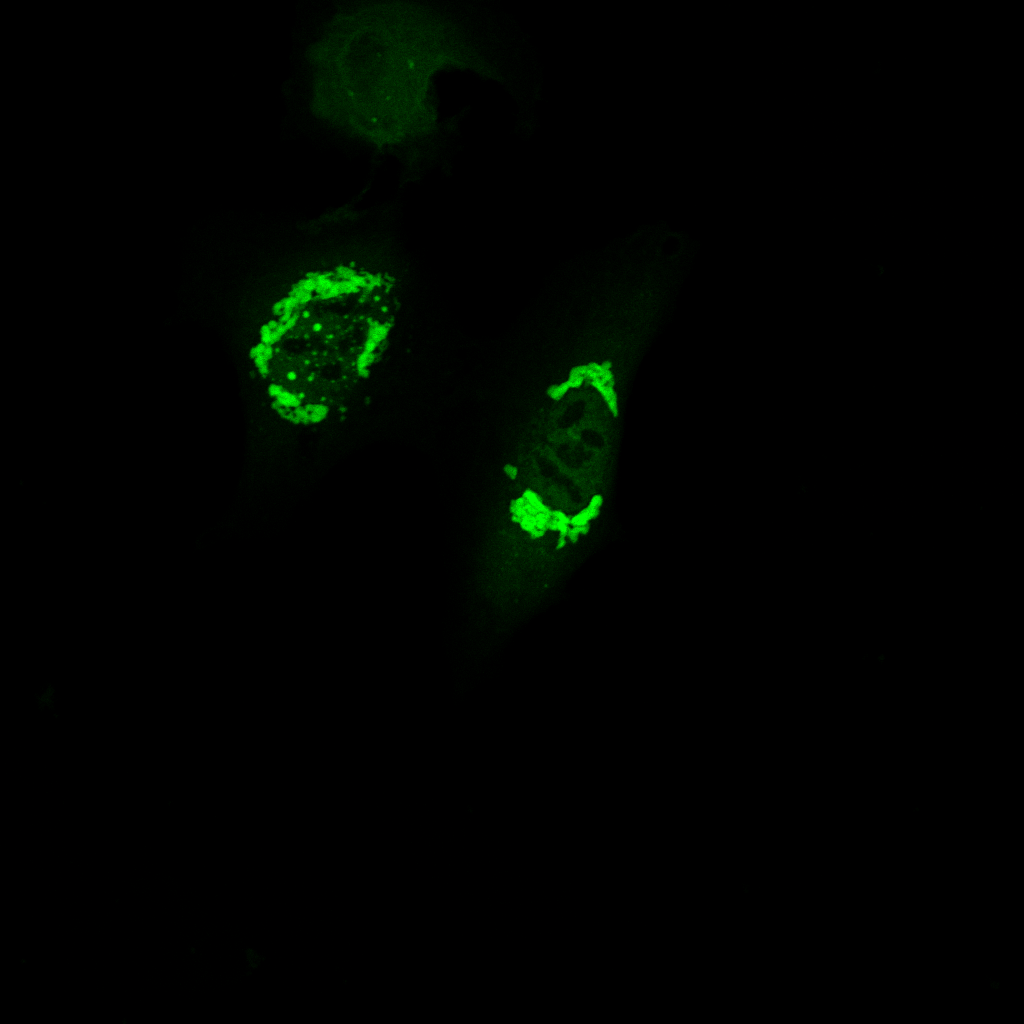

Supplement: Supplementary file 7 — Source data Fig. 5 [file 44319_2024_203_MOESM7_ESM.zip › 5C/3. RHOBTB1+RASSF8+YAP/YAP.tif]

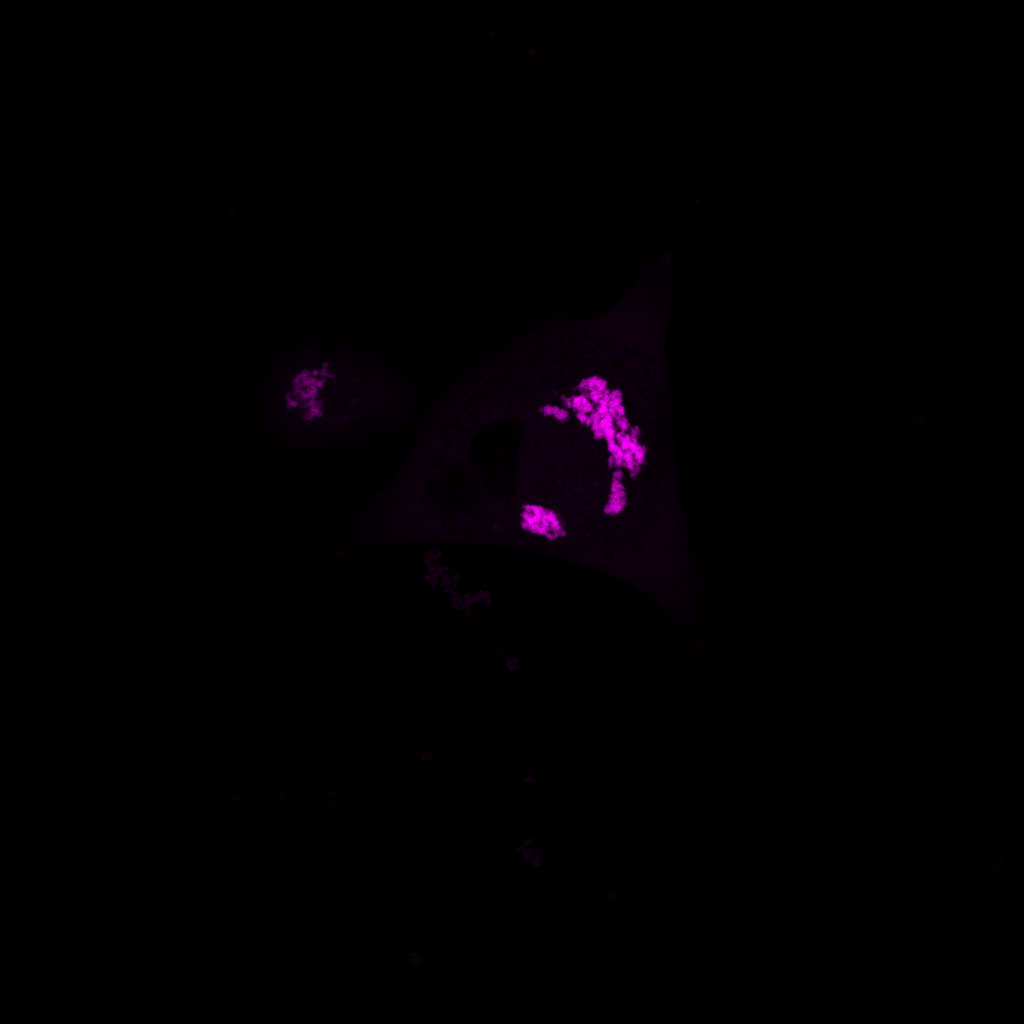

Supplement: Supplementary file 7 — Source data Fig. 5 [file 44319_2024_203_MOESM7_ESM.zip › 5D/1. RASD1+YAP/RASD1.tif]

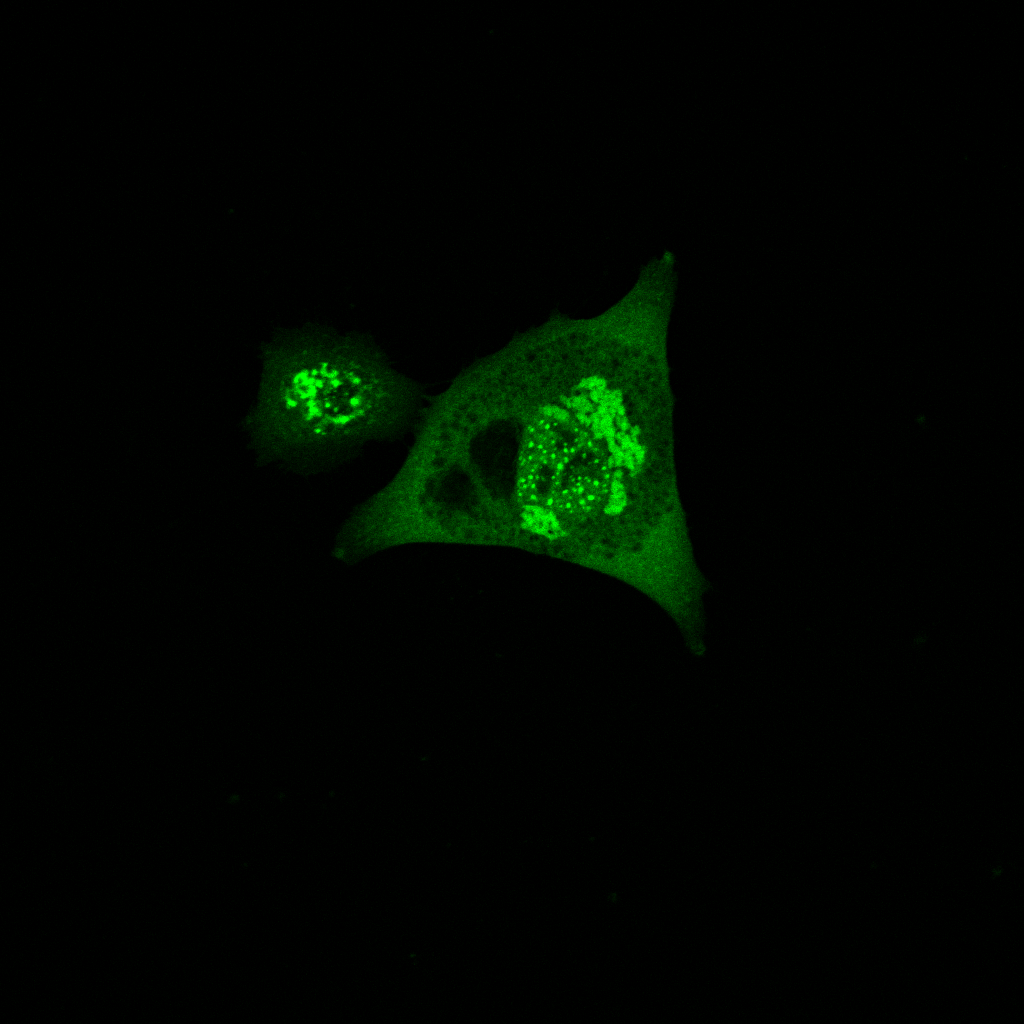

Supplement: Supplementary file 7 — Source data Fig. 5 [file 44319_2024_203_MOESM7_ESM.zip › 5D/1. RASD1+YAP/YAP.tif]

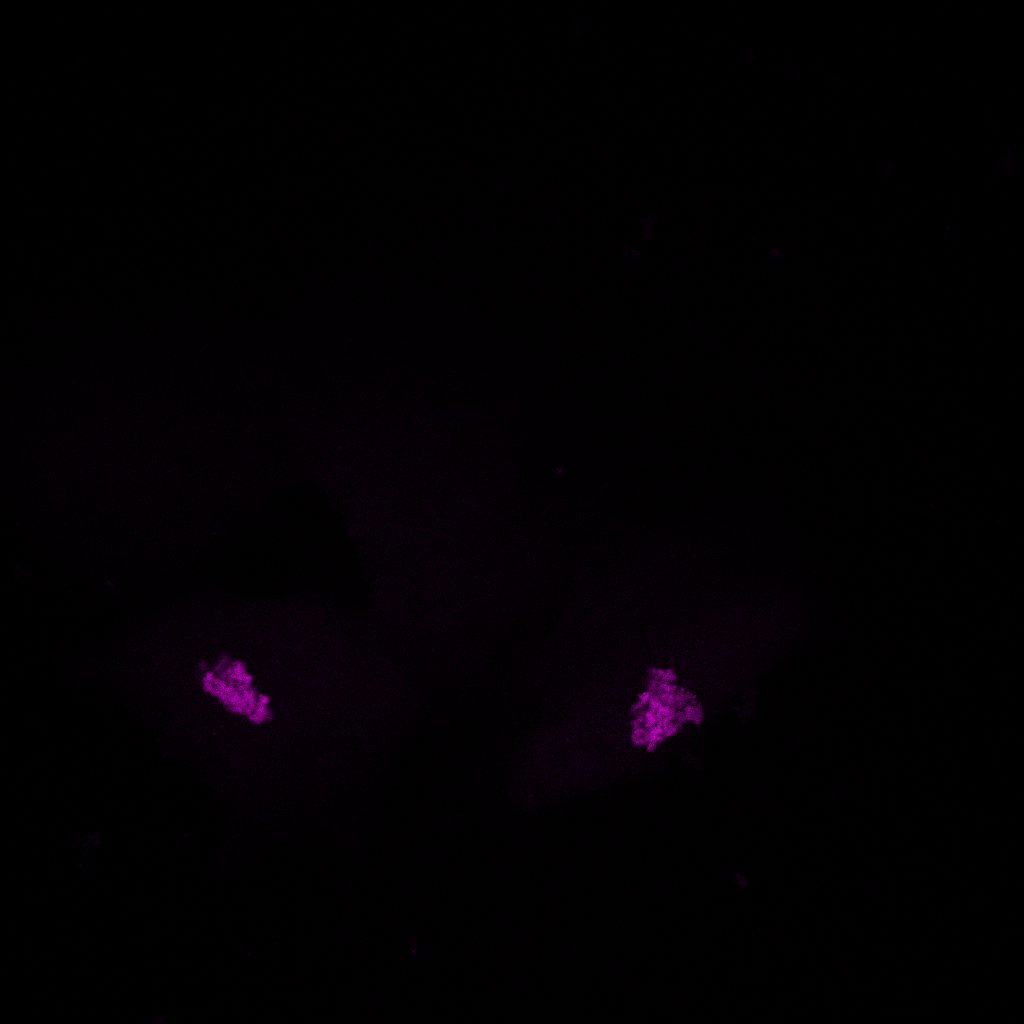

Supplement: Supplementary file 7 — Source data Fig. 5 [file 44319_2024_203_MOESM7_ESM.zip › 5D/2. RASL11B+YAP/RASL11B.tif]

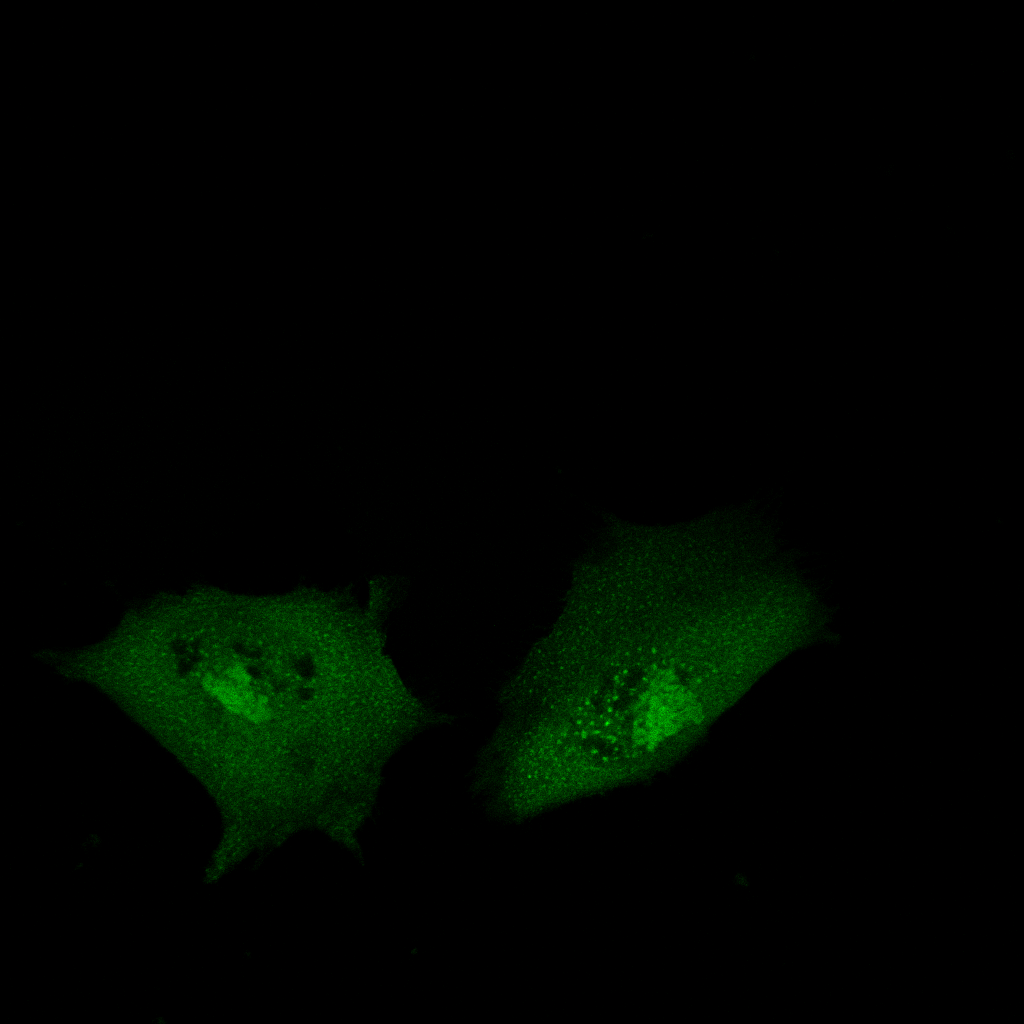

Supplement: Supplementary file 7 — Source data Fig. 5 [file 44319_2024_203_MOESM7_ESM.zip › 5D/2. RASL11B+YAP/YAP.tif]

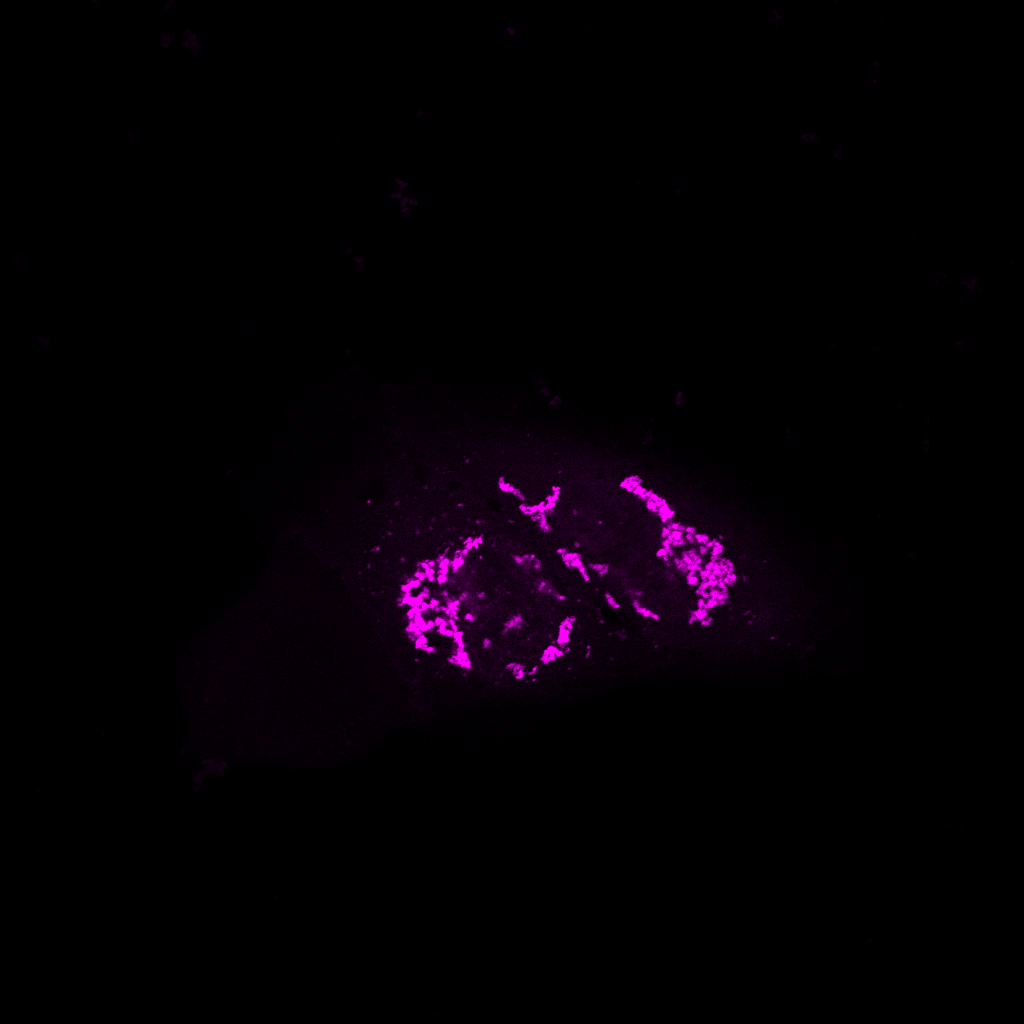

Supplement: Supplementary file 7 — Source data Fig. 5 [file 44319_2024_203_MOESM7_ESM.zip › 5D/3. RHOBTB1+YAP/RHOBTB1.tif]

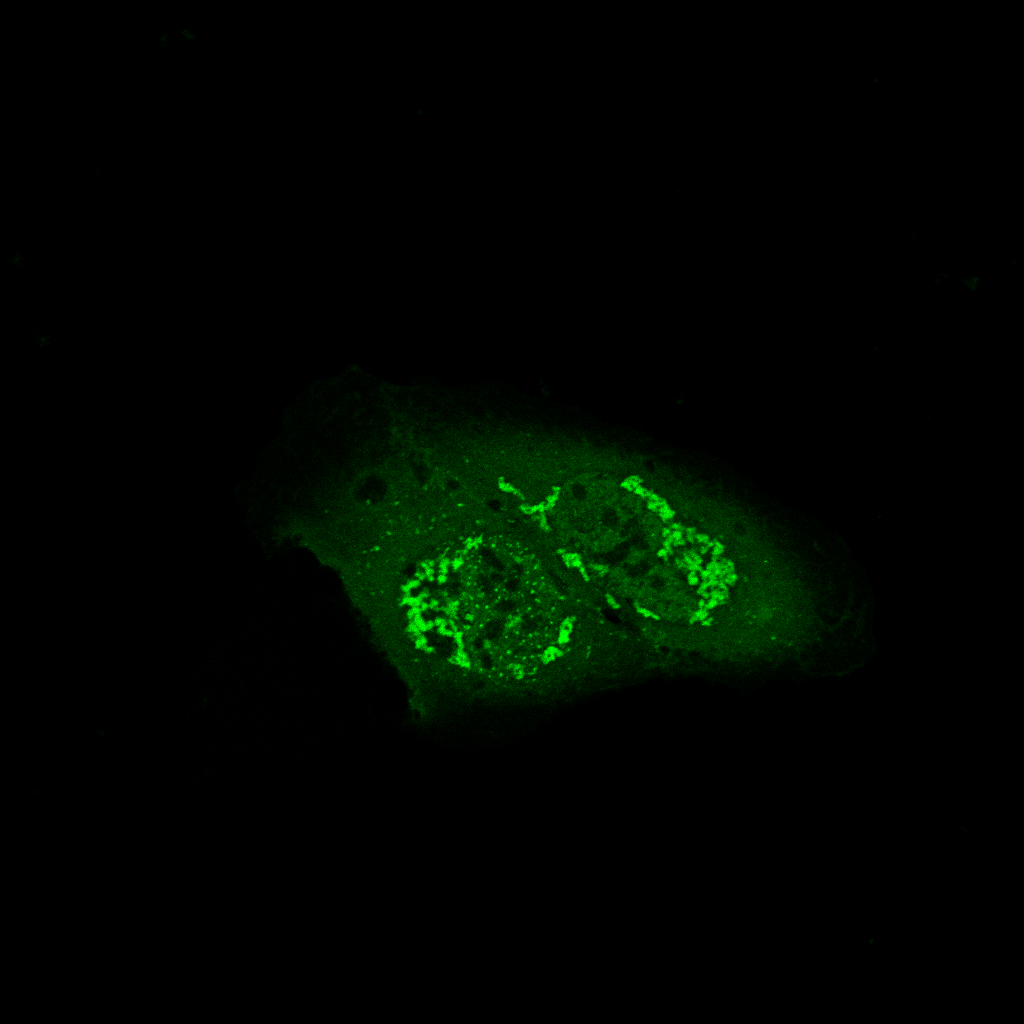

Supplement: Supplementary file 7 — Source data Fig. 5 [file 44319_2024_203_MOESM7_ESM.zip › 5D/3. RHOBTB1+YAP/YAP.tif]

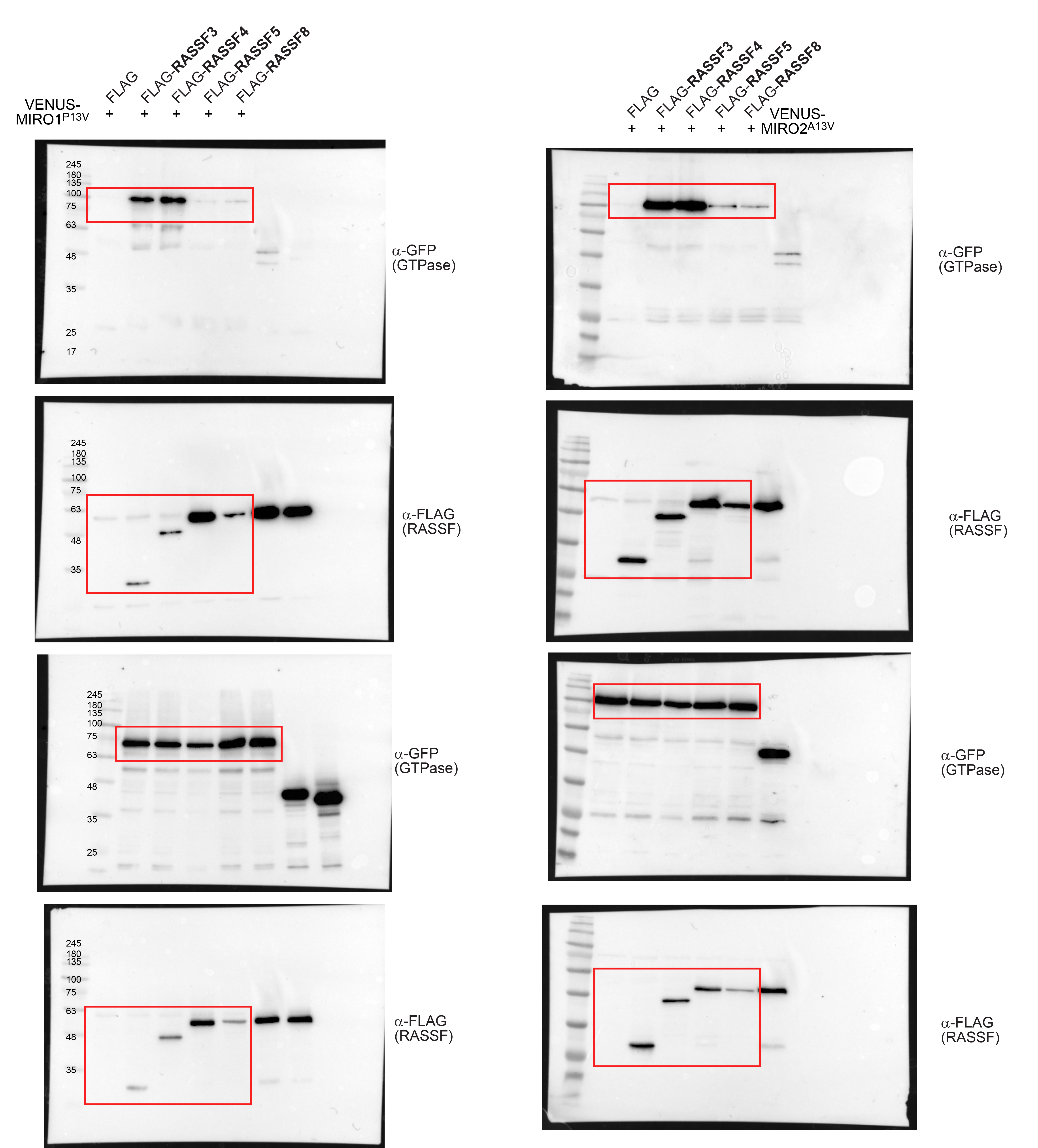

Supplement: Supplementary file 8 — Source data Fig. 6 [file 44319_2024_203_MOESM8_ESM.zip › 6C/6C_MIRO Full length IP.jpg]

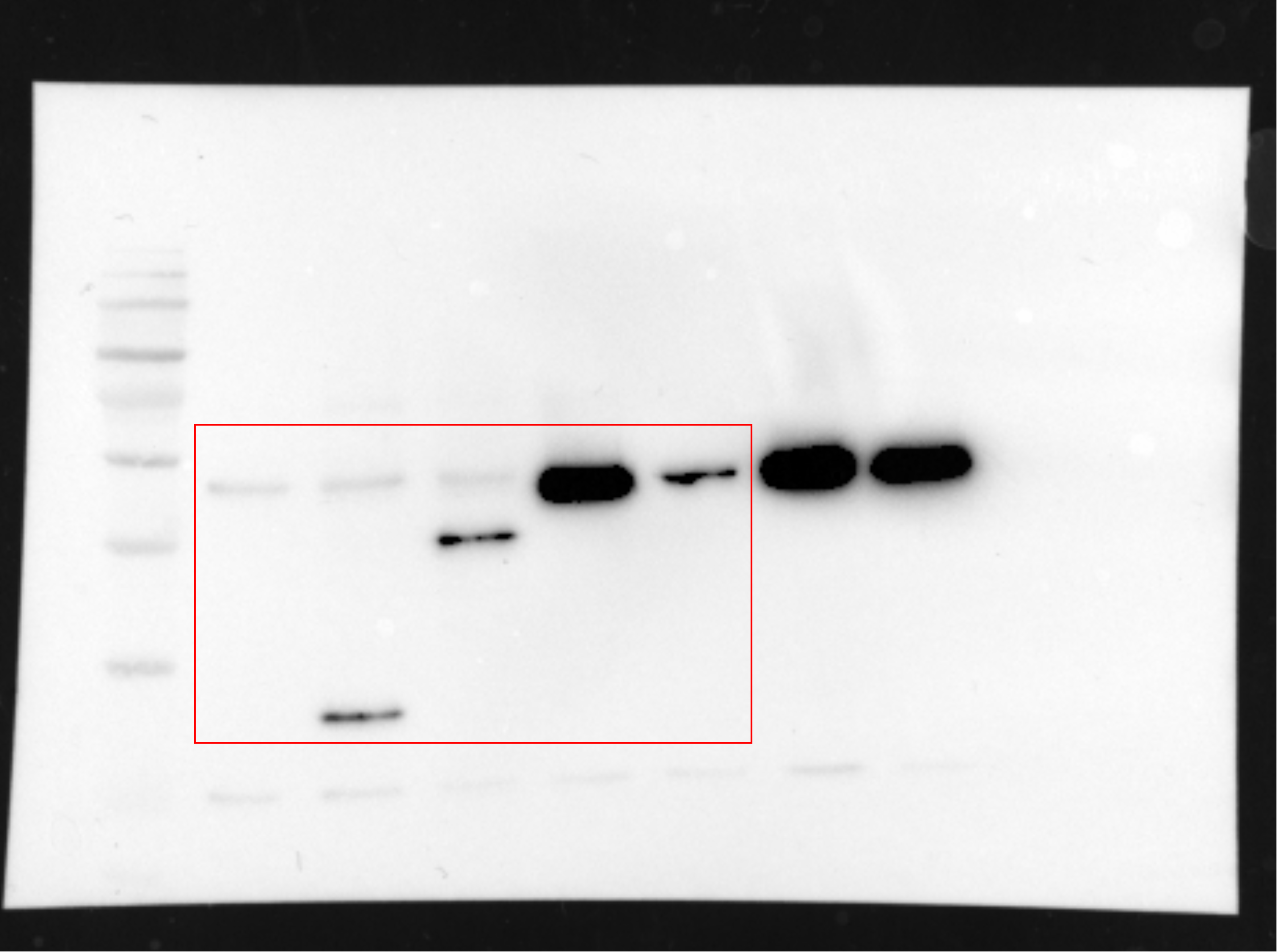

Supplement: Supplementary file 8 — Source data Fig. 6 [file 44319_2024_203_MOESM8_ESM.zip › 6C/MIRO1/IP-AntiFLAG.tif]

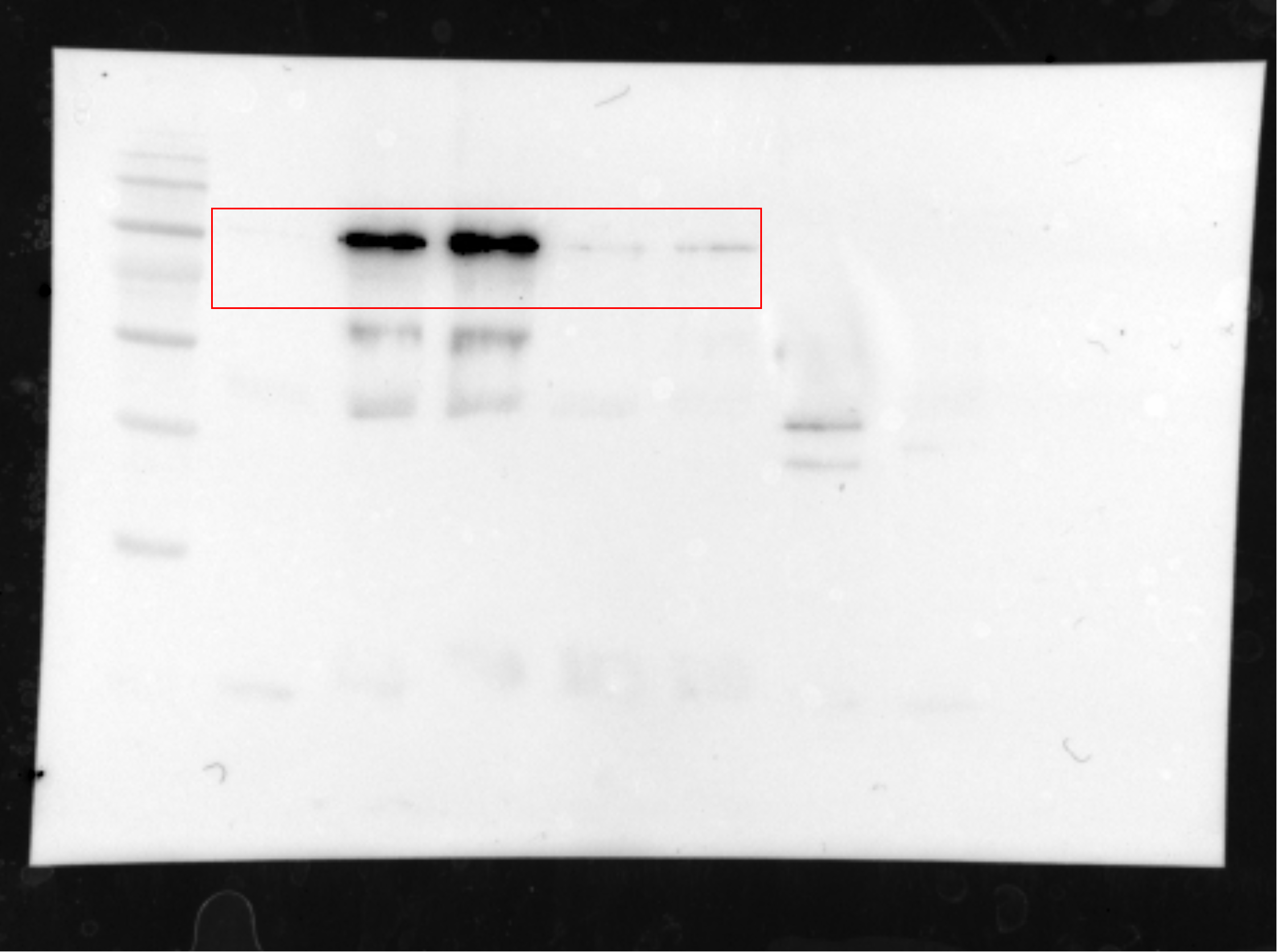

Supplement: Supplementary file 8 — Source data Fig. 6 [file 44319_2024_203_MOESM8_ESM.zip › 6C/MIRO1/IP-AntiGFP.tif]

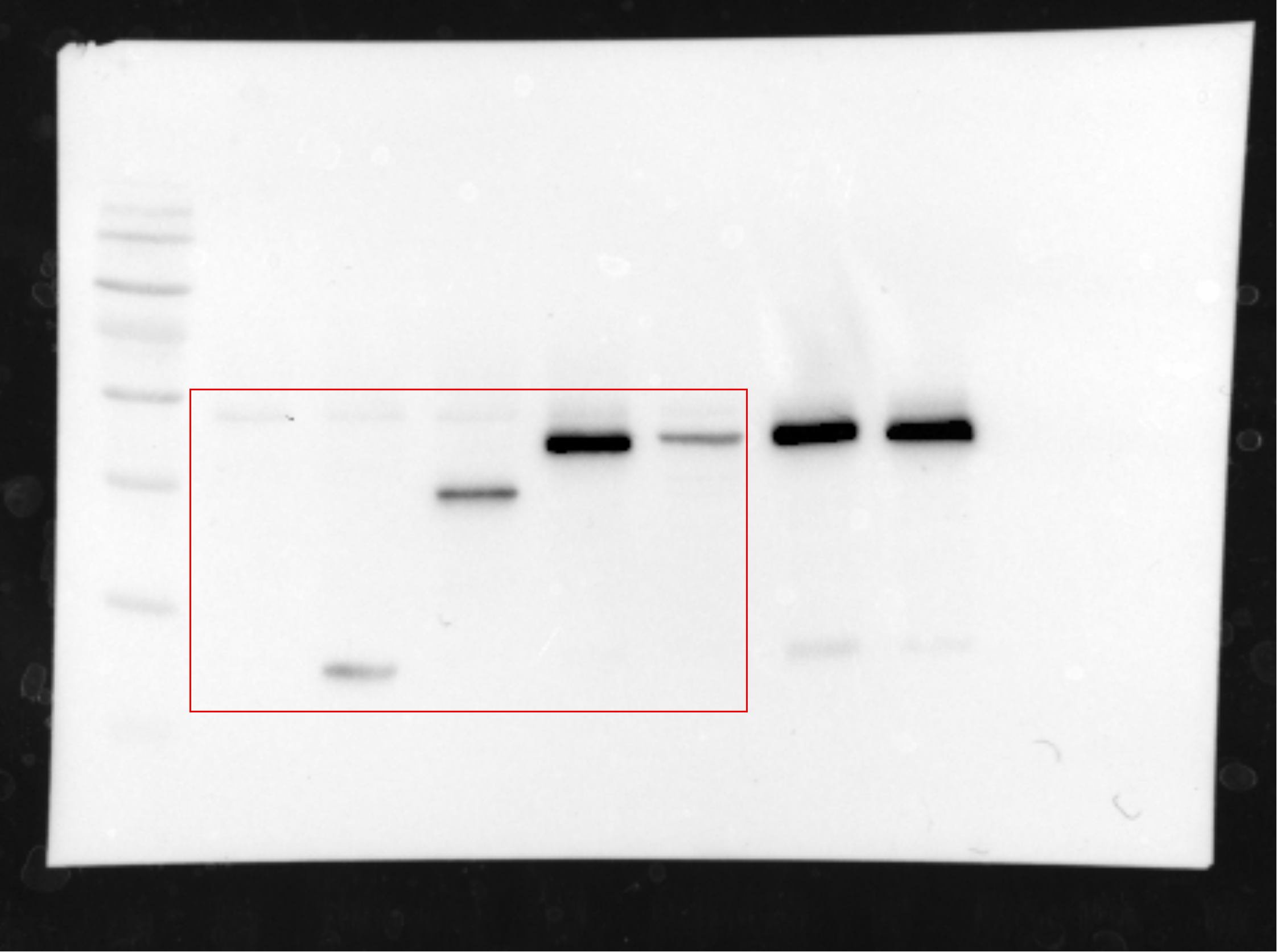

Supplement: Supplementary file 8 — Source data Fig. 6 [file 44319_2024_203_MOESM8_ESM.zip › 6C/MIRO1/Lysate-Anti-FLAG.tif]

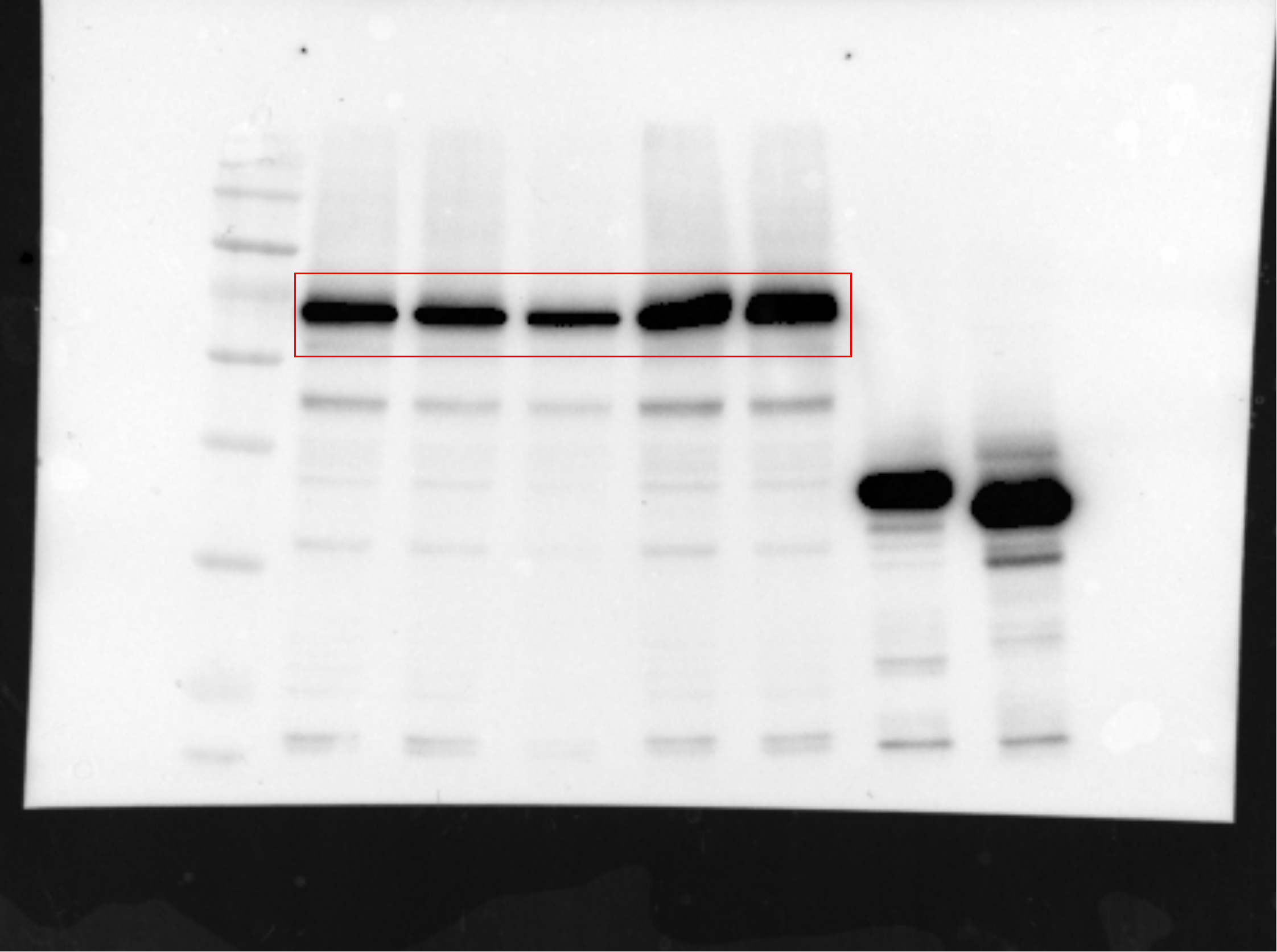

Supplement: Supplementary file 8 — Source data Fig. 6 [file 44319_2024_203_MOESM8_ESM.zip › 6C/MIRO1/Lysate-Anti-GFP.tif]

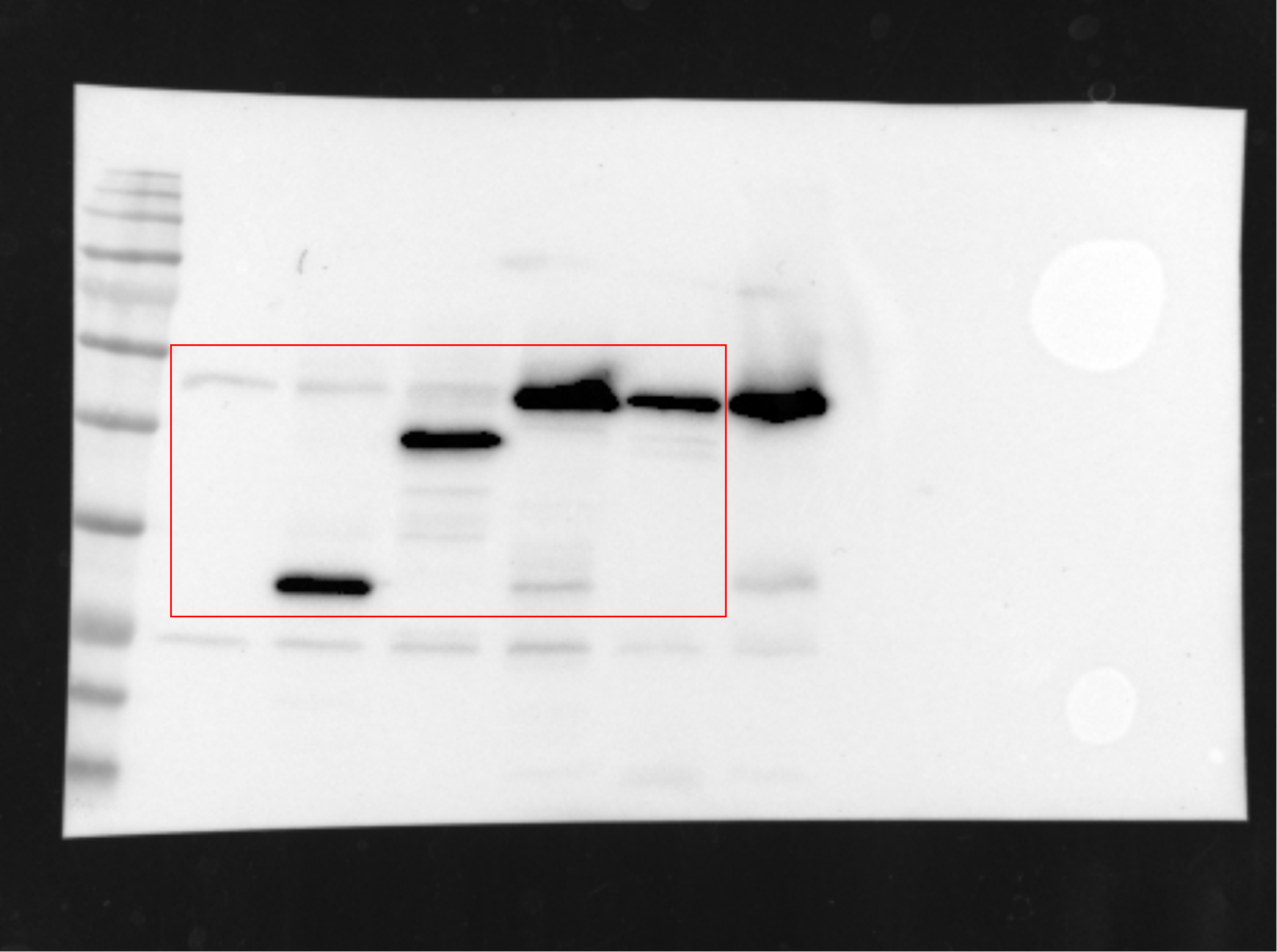

Supplement: Supplementary file 8 — Source data Fig. 6 [file 44319_2024_203_MOESM8_ESM.zip › 6C/MIRO2/IP-Anti-FLAG.tif]

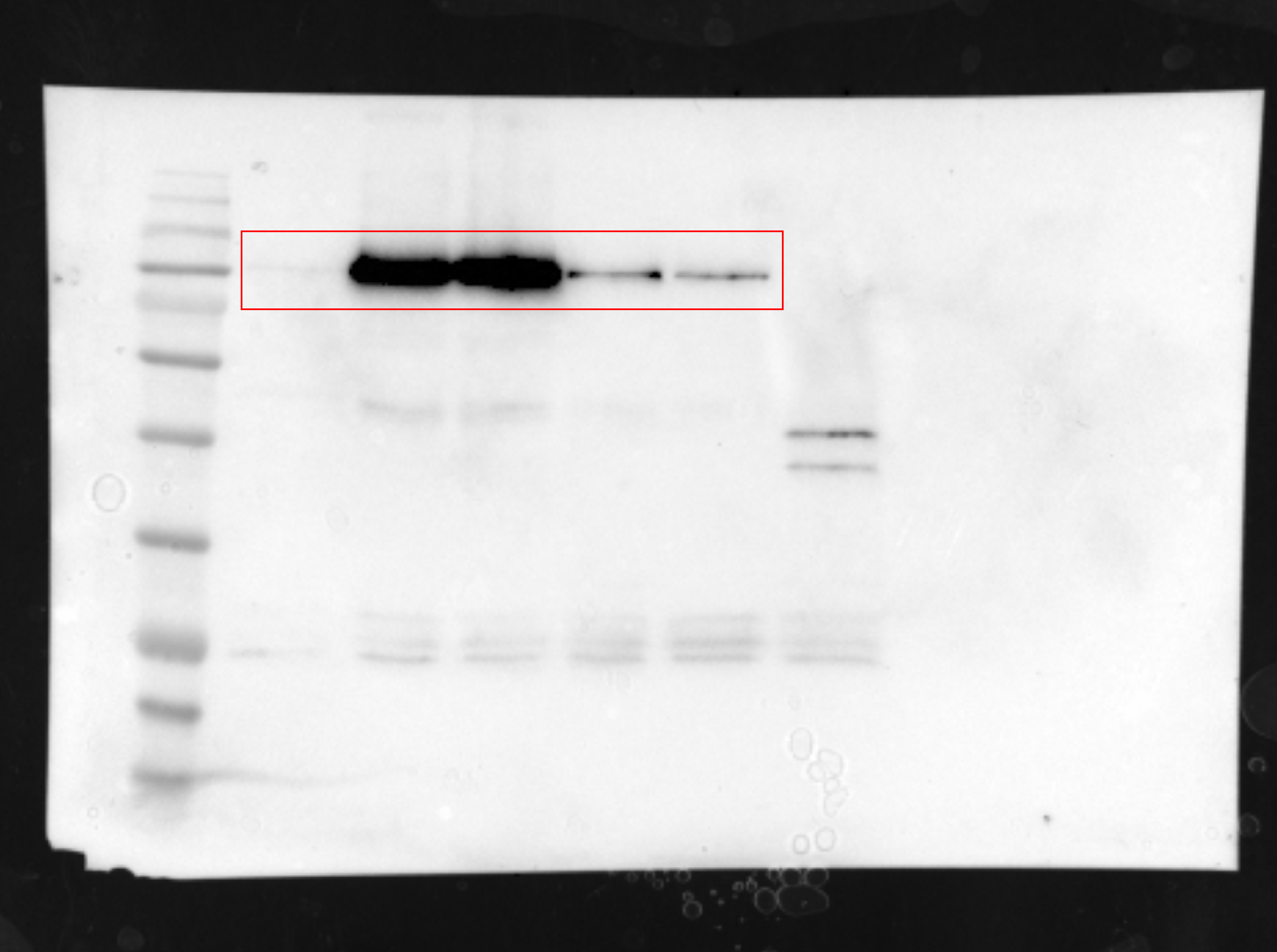

Supplement: Supplementary file 8 — Source data Fig. 6 [file 44319_2024_203_MOESM8_ESM.zip › 6C/MIRO2/IP-Anti-GFP.tif]

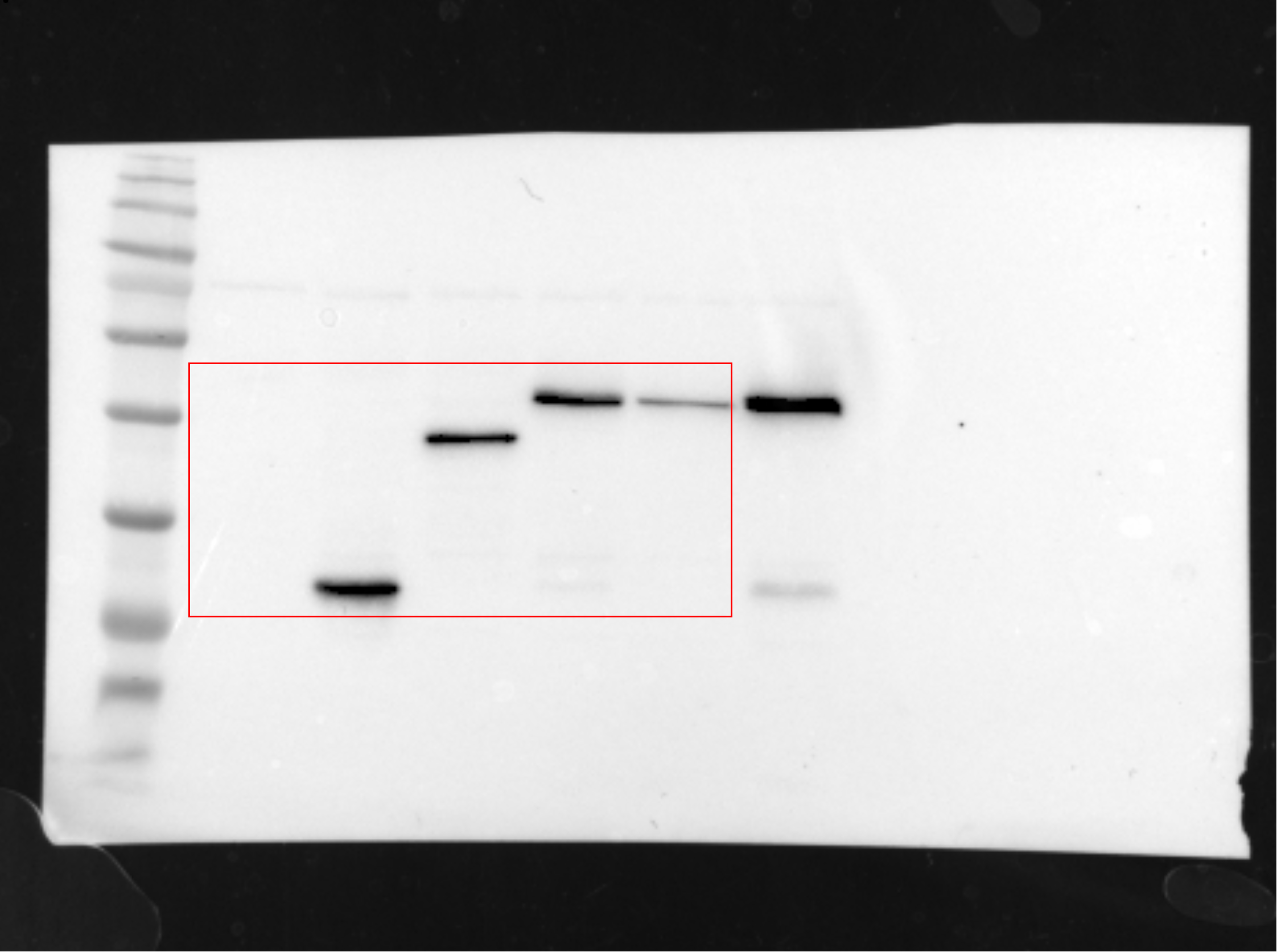

Supplement: Supplementary file 8 — Source data Fig. 6 [file 44319_2024_203_MOESM8_ESM.zip › 6C/MIRO2/Lysate-Anti-FLAG.tif]

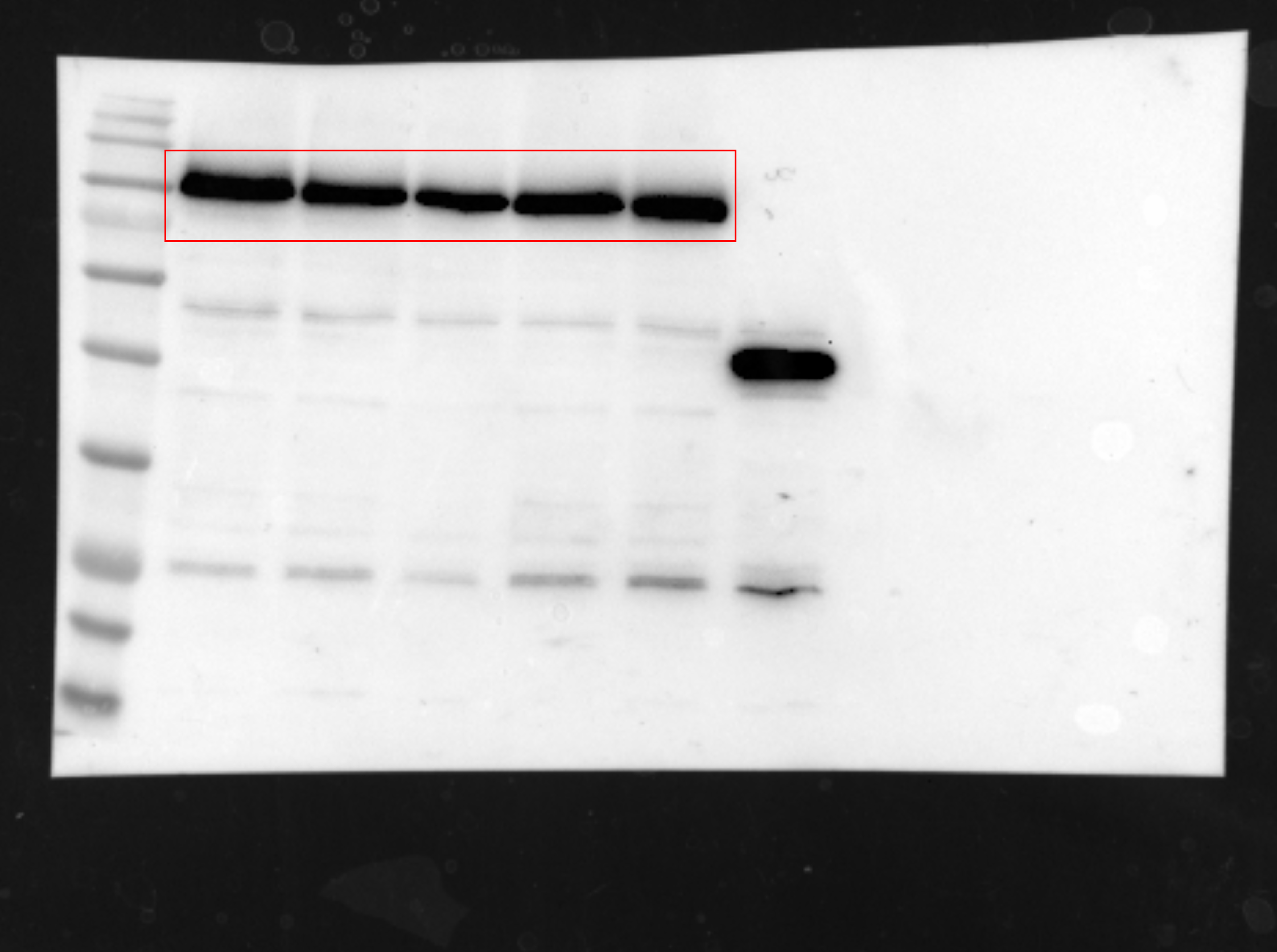

Supplement: Supplementary file 8 — Source data Fig. 6 [file 44319_2024_203_MOESM8_ESM.zip › 6C/MIRO2/Lysate-Anti-GFP.tif]

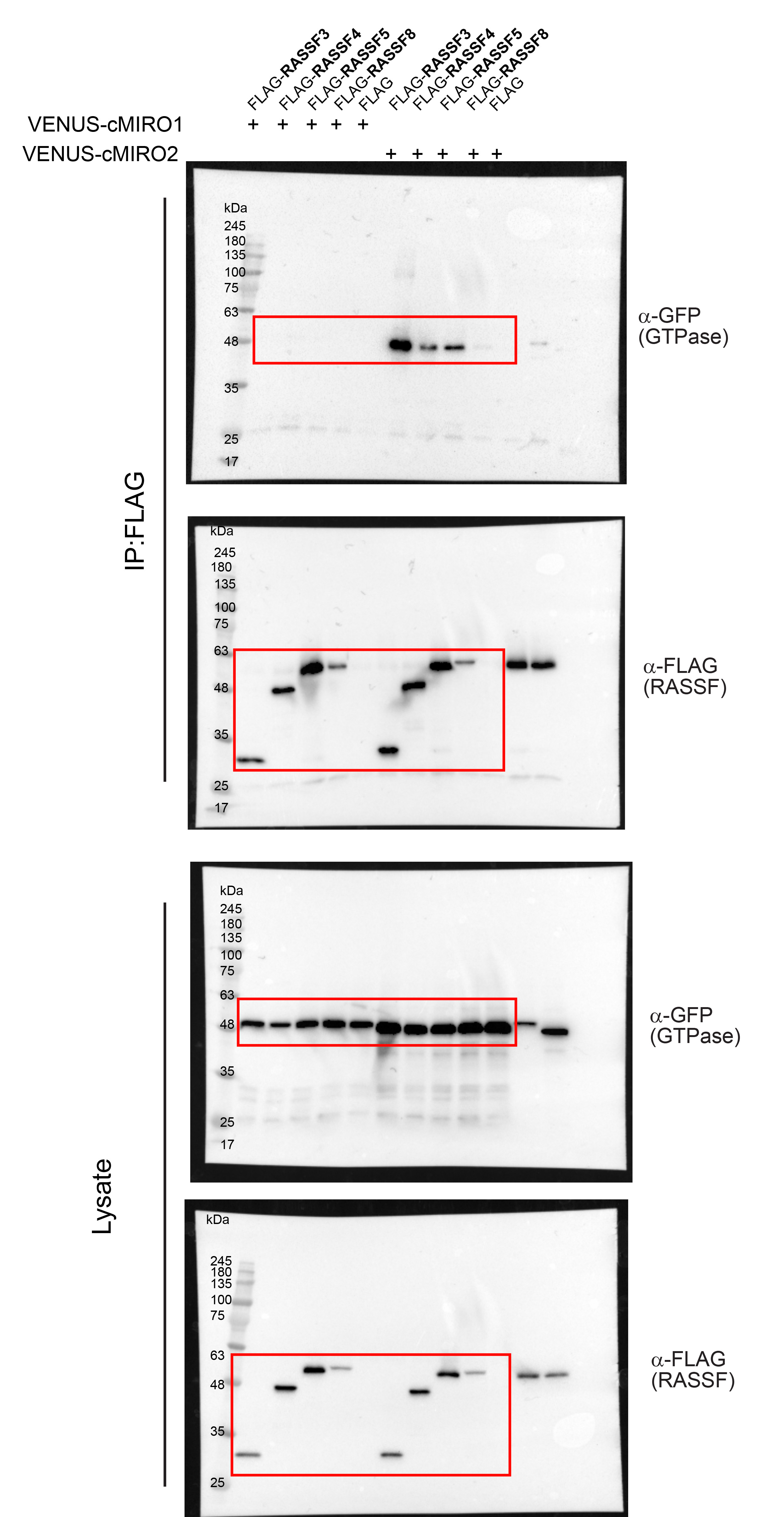

Supplement: Supplementary file 8 — Source data Fig. 6 [file 44319_2024_203_MOESM8_ESM.zip › 6D/6D_Blots.jpg]

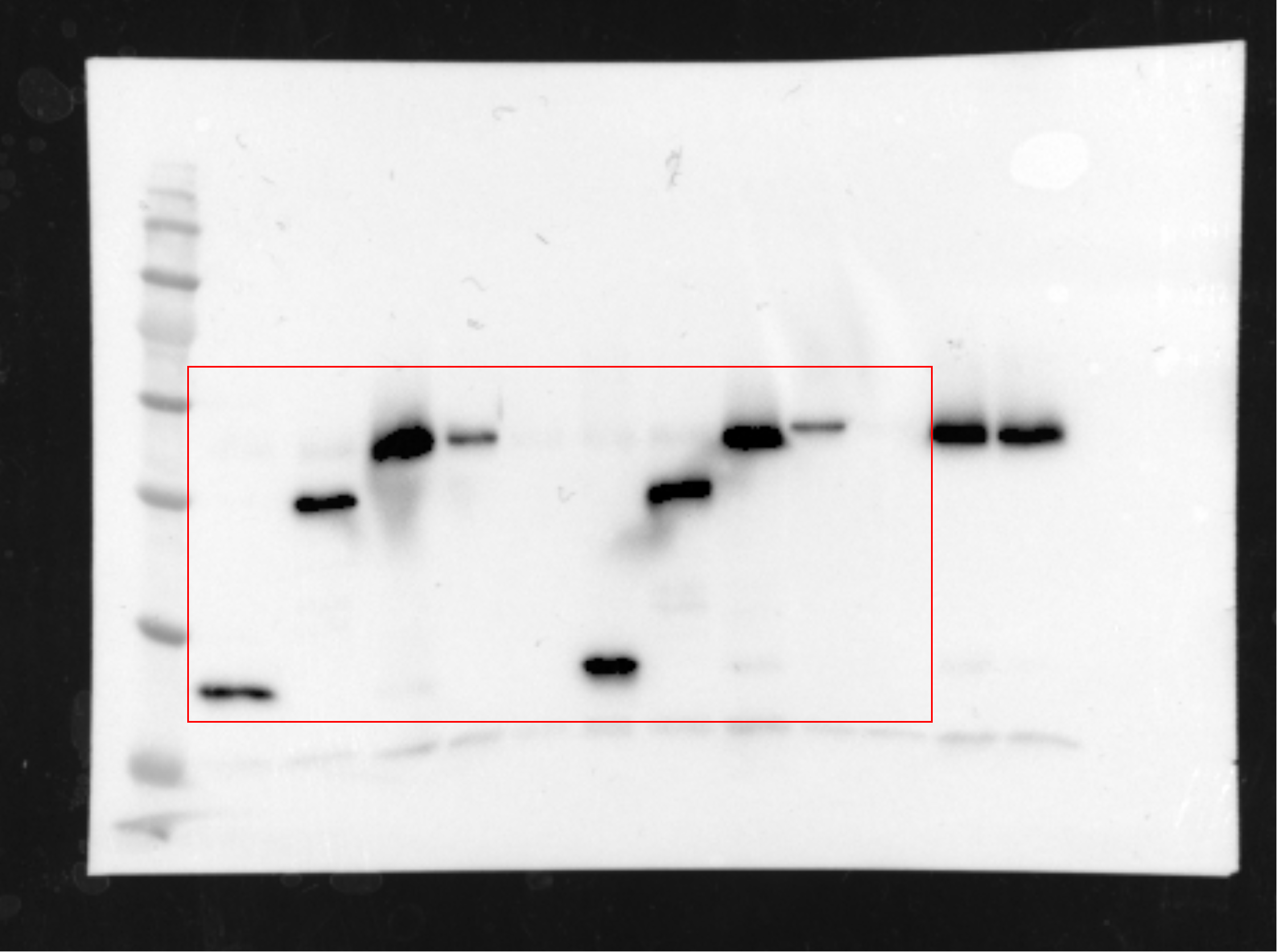

Supplement: Supplementary file 8 — Source data Fig. 6 [file 44319_2024_203_MOESM8_ESM.zip › 6D/IP_Anti-FLAG.tif]

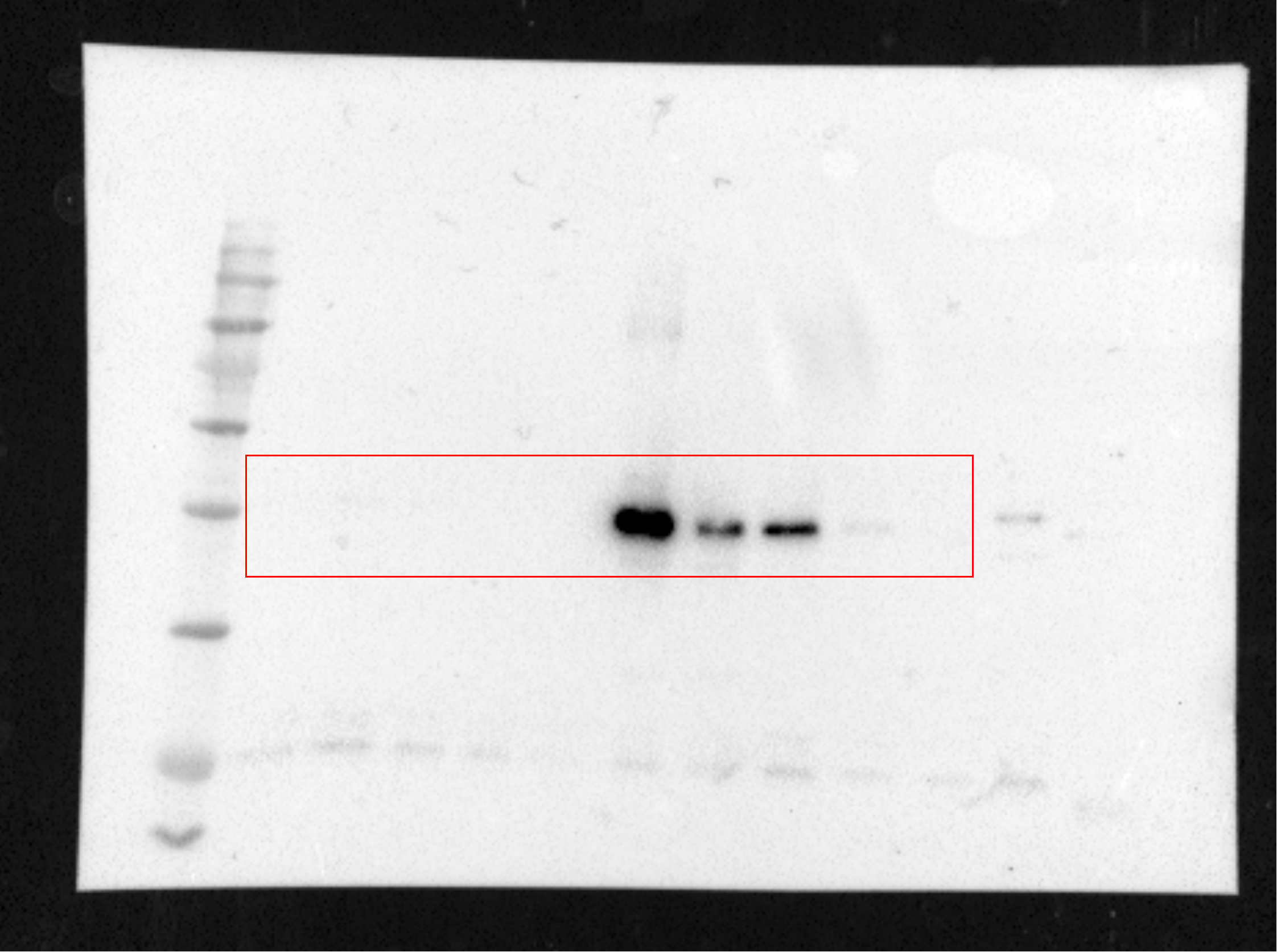

Supplement: Supplementary file 8 — Source data Fig. 6 [file 44319_2024_203_MOESM8_ESM.zip › 6D/IP_Anti-GFP.tif]

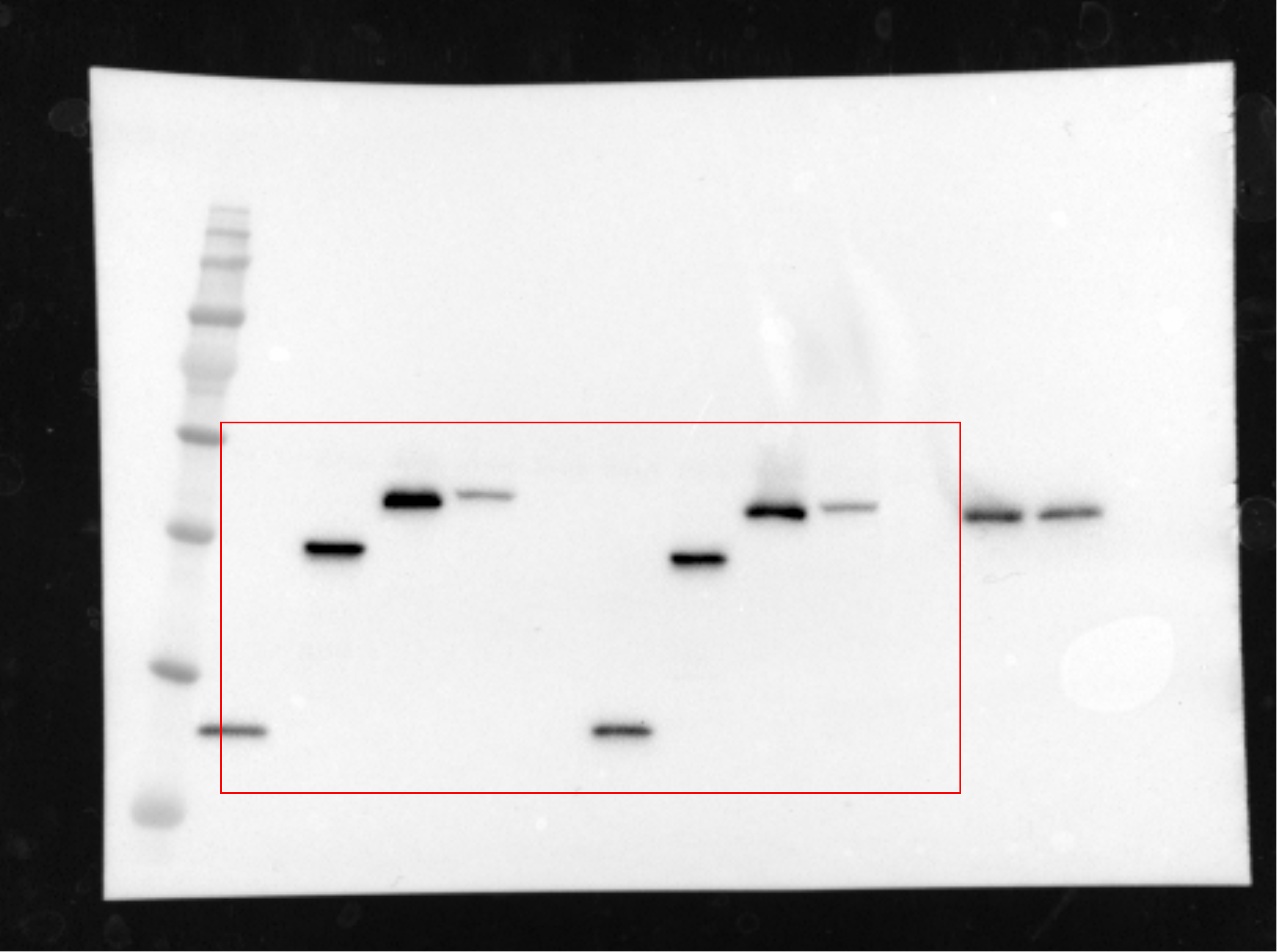

Supplement: Supplementary file 8 — Source data Fig. 6 [file 44319_2024_203_MOESM8_ESM.zip › 6D/Lysate_Anti-FLAG.tif]

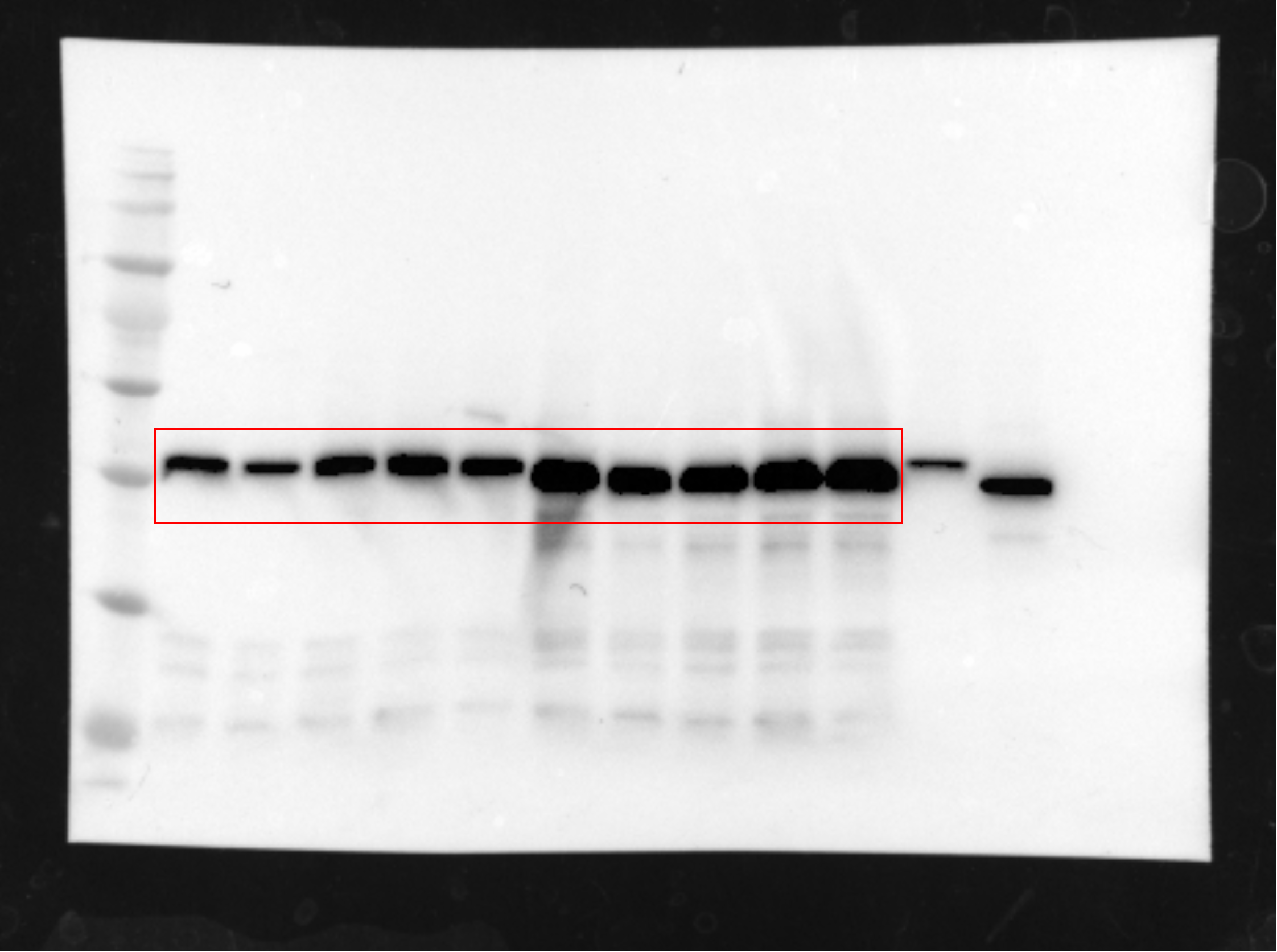

Supplement: Supplementary file 8 — Source data Fig. 6 [file 44319_2024_203_MOESM8_ESM.zip › 6D/Lysate_Anti-GFP.tif]

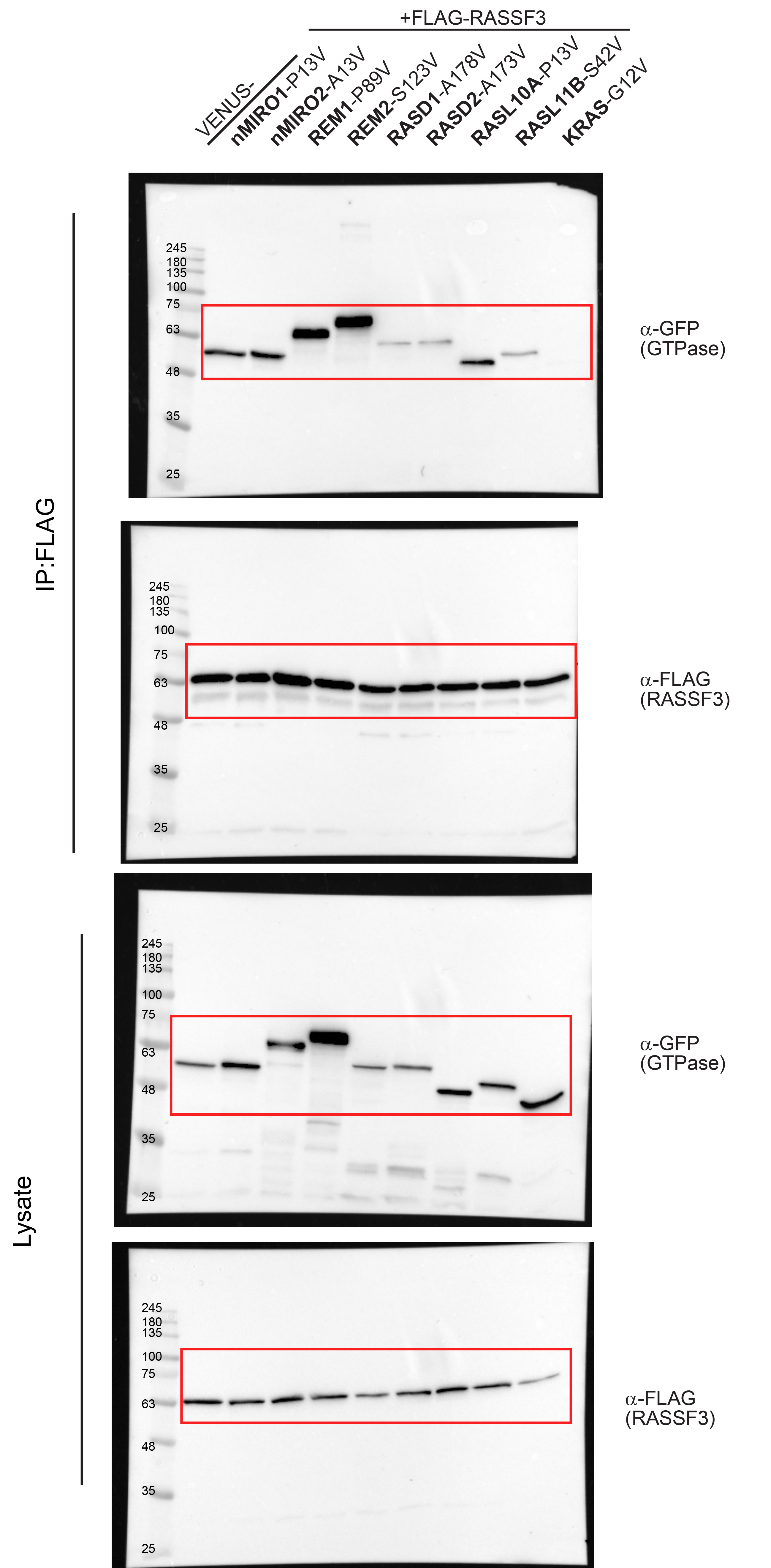

Supplement: Supplementary file 8 — Source data Fig. 6 [file 44319_2024_203_MOESM8_ESM.zip › 6A/RASSF3_IP/6A_RASSF3 IP.jpg]

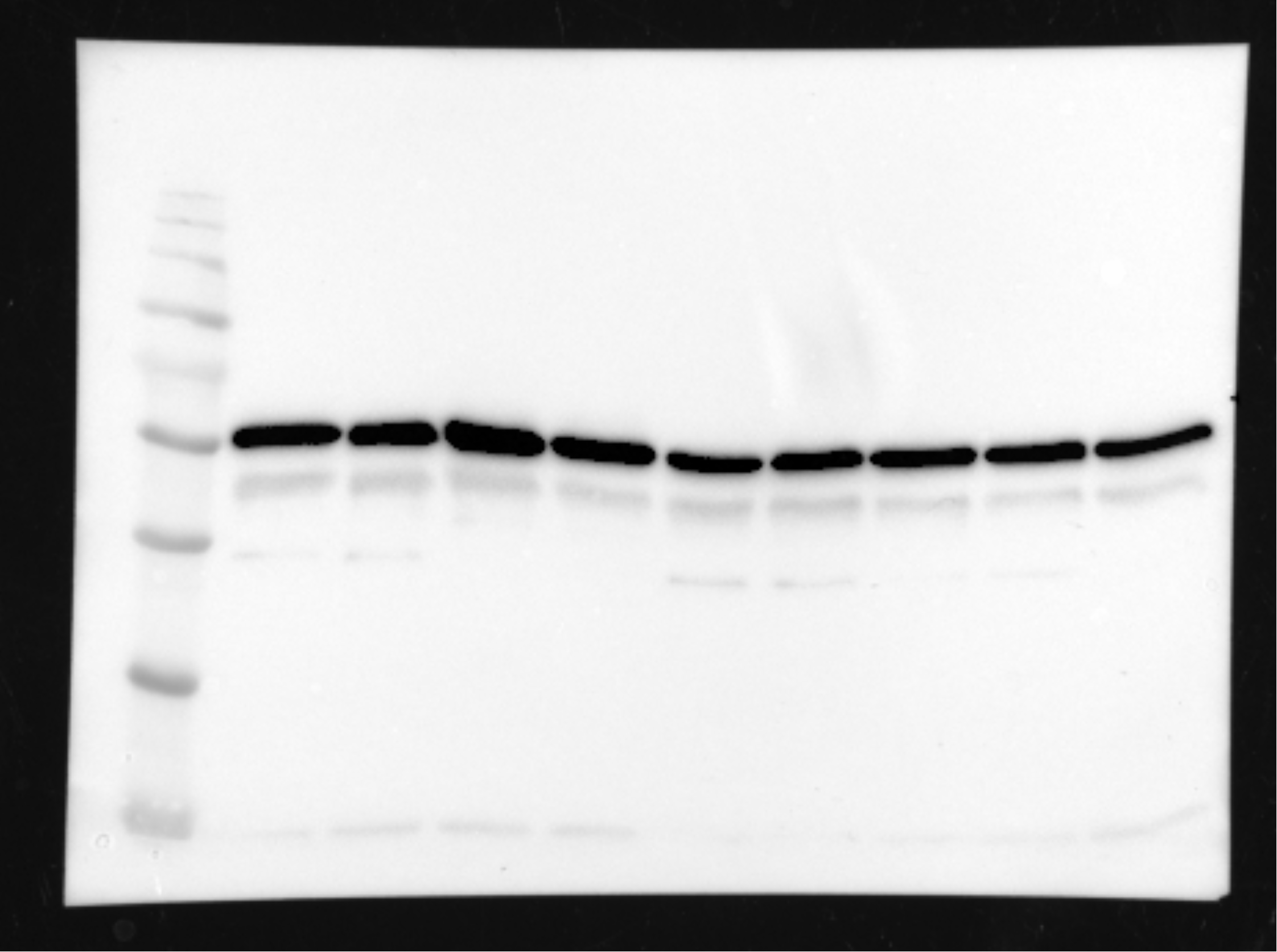

Supplement: Supplementary file 8 — Source data Fig. 6 [file 44319_2024_203_MOESM8_ESM.zip › 6A/RASSF3_IP/Ladder+5sec-IP-Flag-R3.tif]

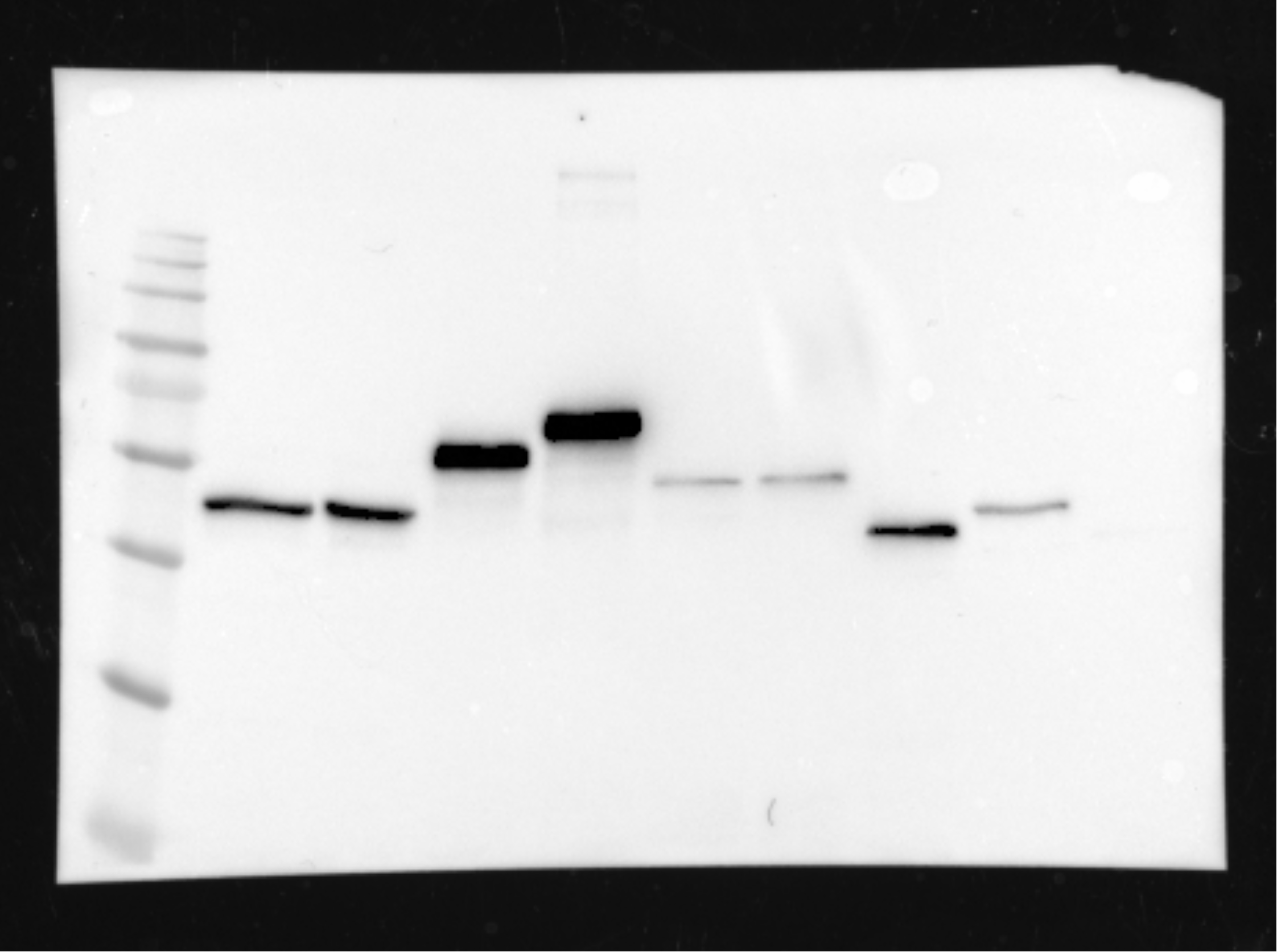

Supplement: Supplementary file 8 — Source data Fig. 6 [file 44319_2024_203_MOESM8_ESM.zip › 6A/RASSF3_IP/Ladder+5sec-IP-GFP-R3.tif]

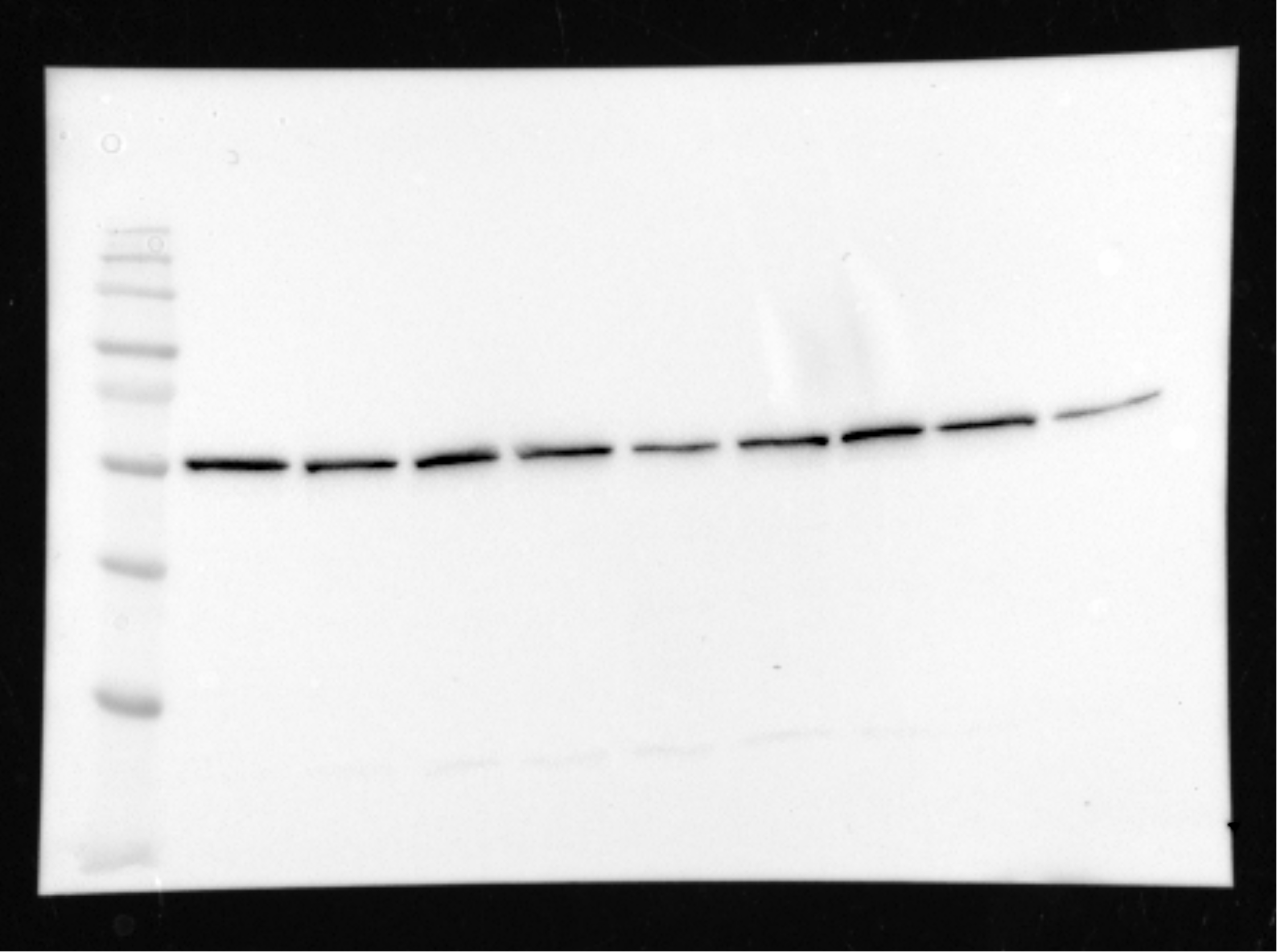

Supplement: Supplementary file 8 — Source data Fig. 6 [file 44319_2024_203_MOESM8_ESM.zip › 6A/RASSF3_IP/Ladder+5sec-Lysate-Flag-R3.tif]

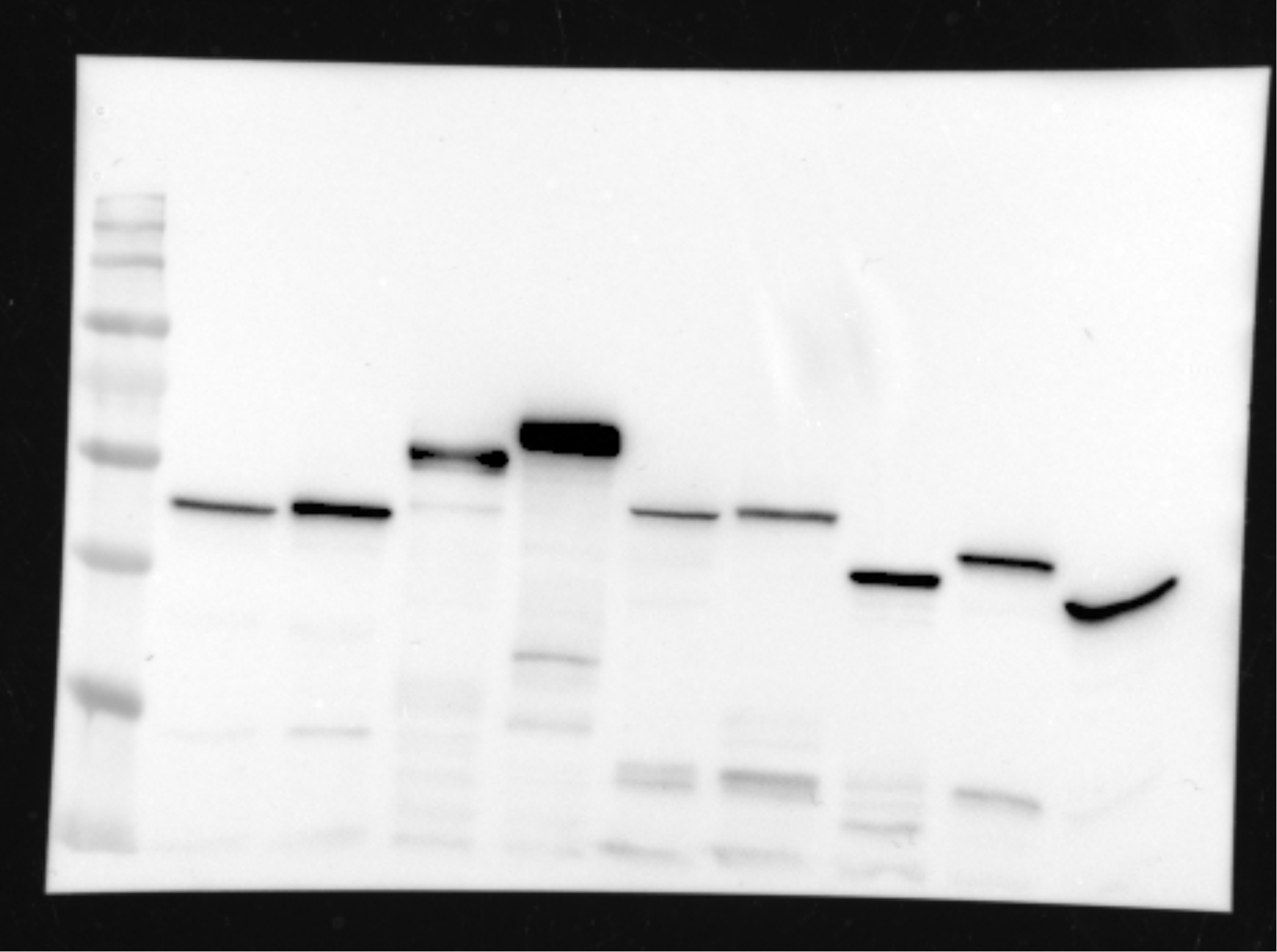

Supplement: Supplementary file 8 — Source data Fig. 6 [file 44319_2024_203_MOESM8_ESM.zip › 6A/RASSF3_IP/Ladder+5sec-Lysate-GFP-R3.tif]

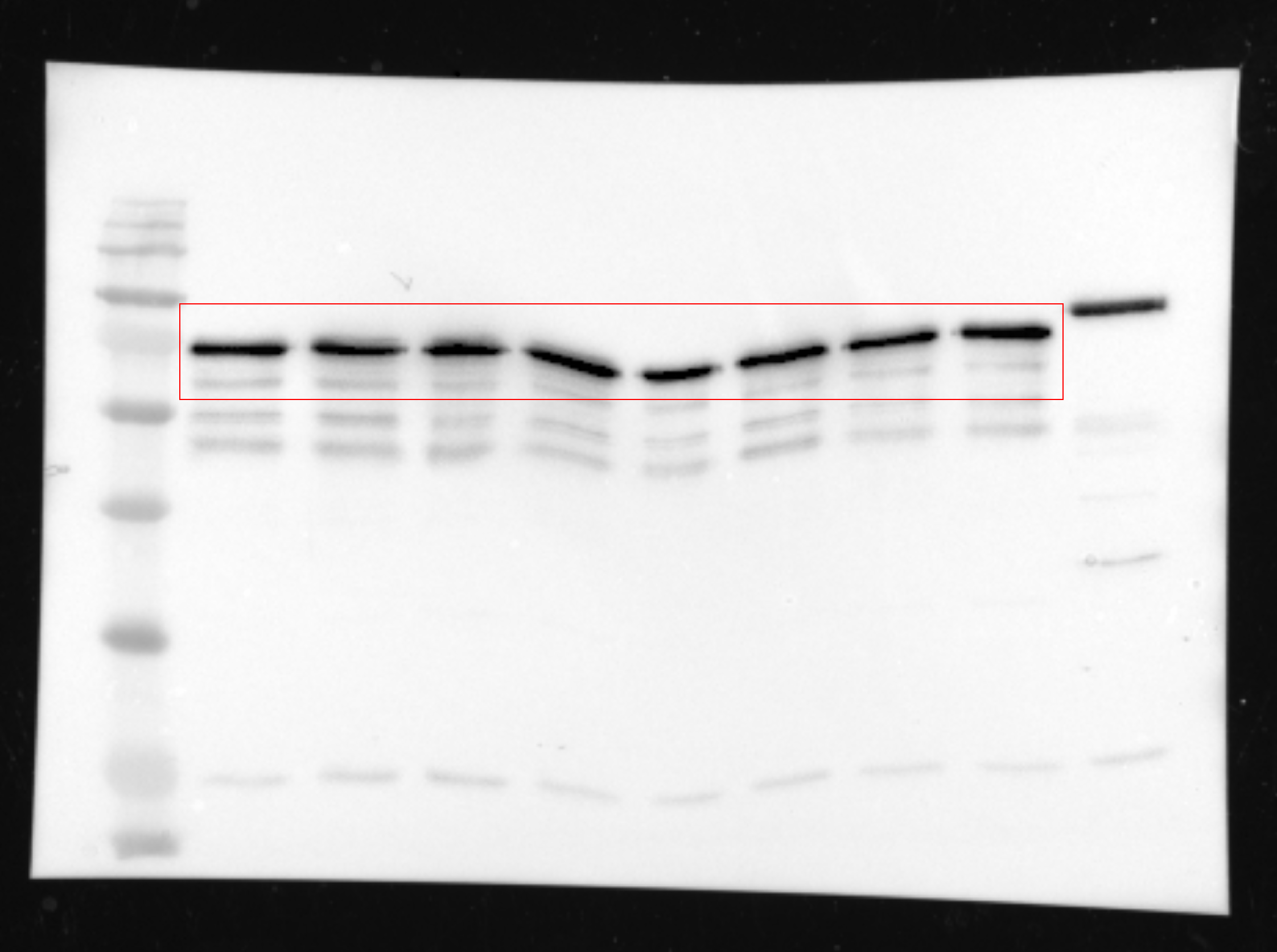

Supplement: Supplementary file 8 — Source data Fig. 6 [file 44319_2024_203_MOESM8_ESM.zip › 6A/RASSF4_IP/5sec+Ladder-IP-Flag-R4.tif]

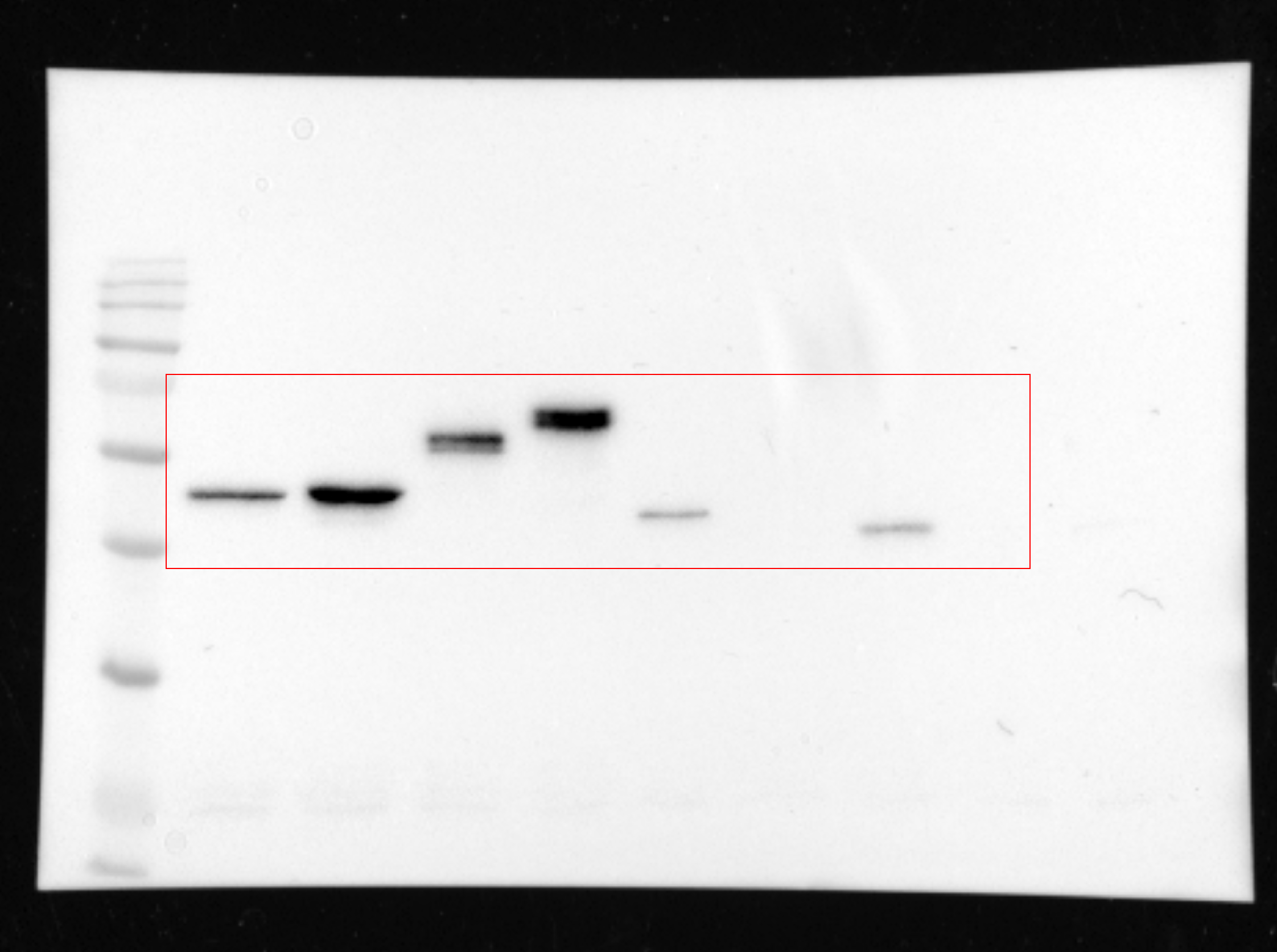

Supplement: Supplementary file 8 — Source data Fig. 6 [file 44319_2024_203_MOESM8_ESM.zip › 6A/RASSF4_IP/5sec+Ladder-IP-GFP-R4.tif]

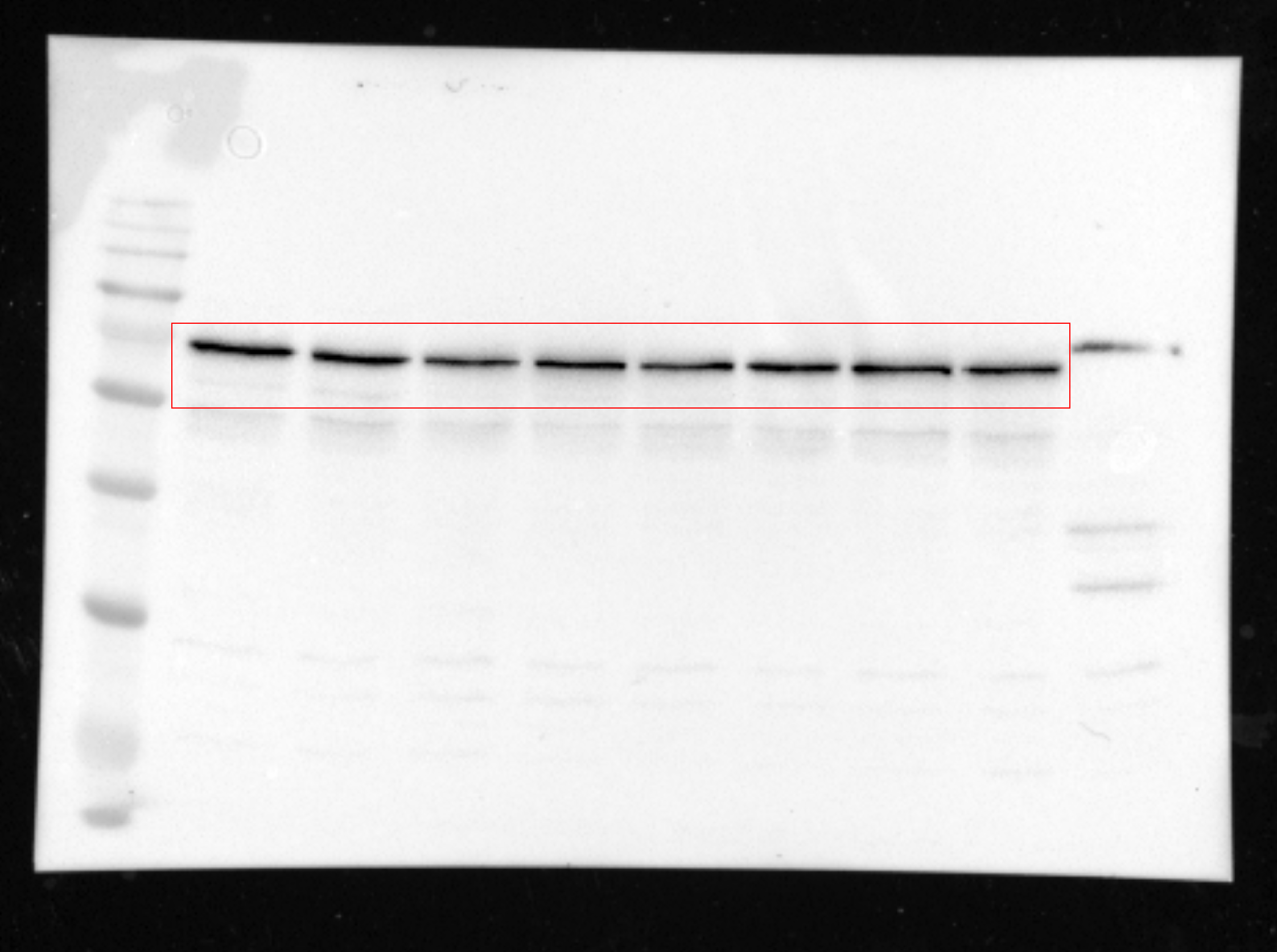

Supplement: Supplementary file 8 — Source data Fig. 6 [file 44319_2024_203_MOESM8_ESM.zip › 6A/RASSF4_IP/5sec+Ladder-Lysate-Flag-R4.tif]

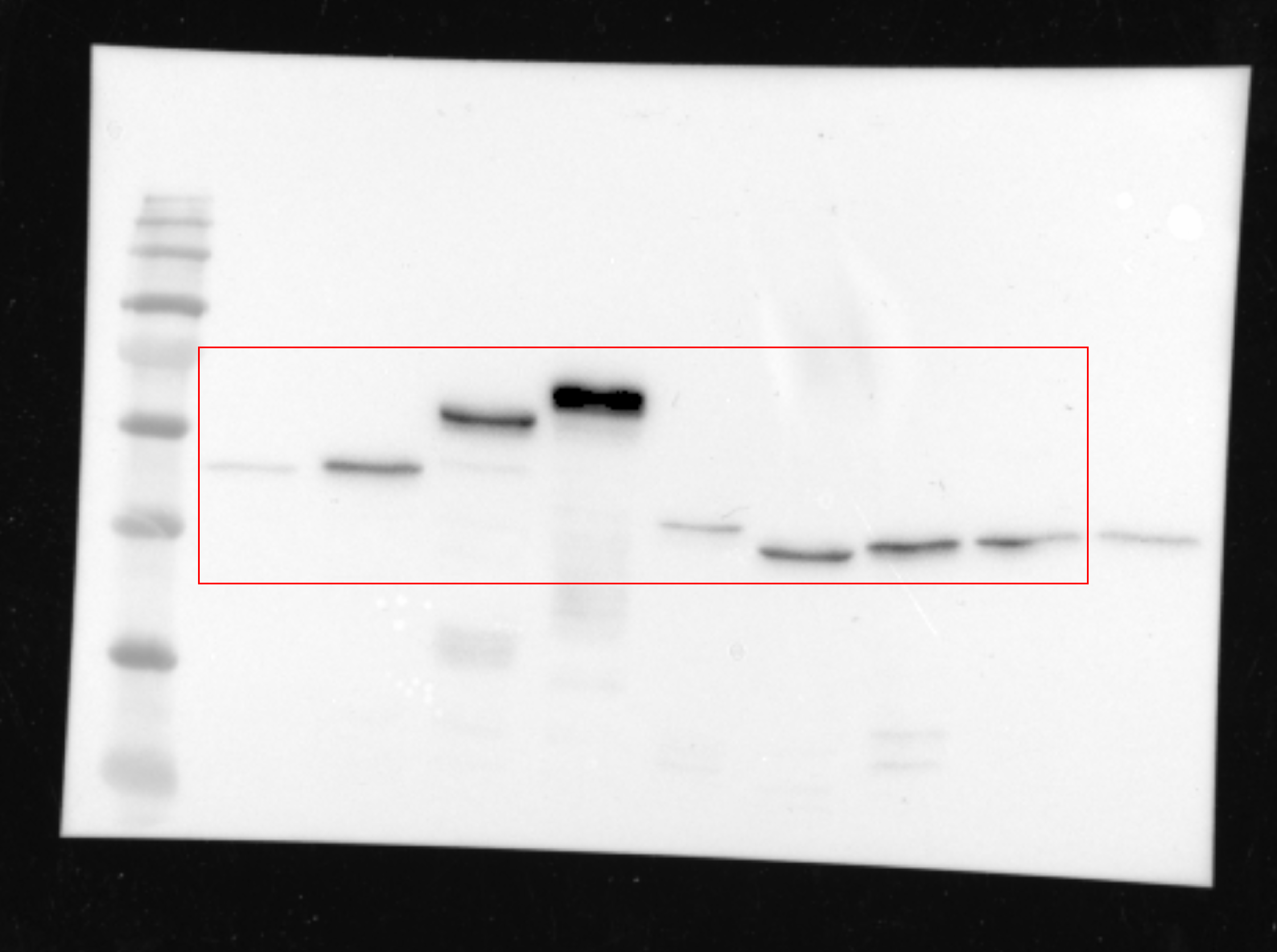

Supplement: Supplementary file 8 — Source data Fig. 6 [file 44319_2024_203_MOESM8_ESM.zip › 6A/RASSF4_IP/5sec+Ladder-Lysate-GFP-R4.tif]

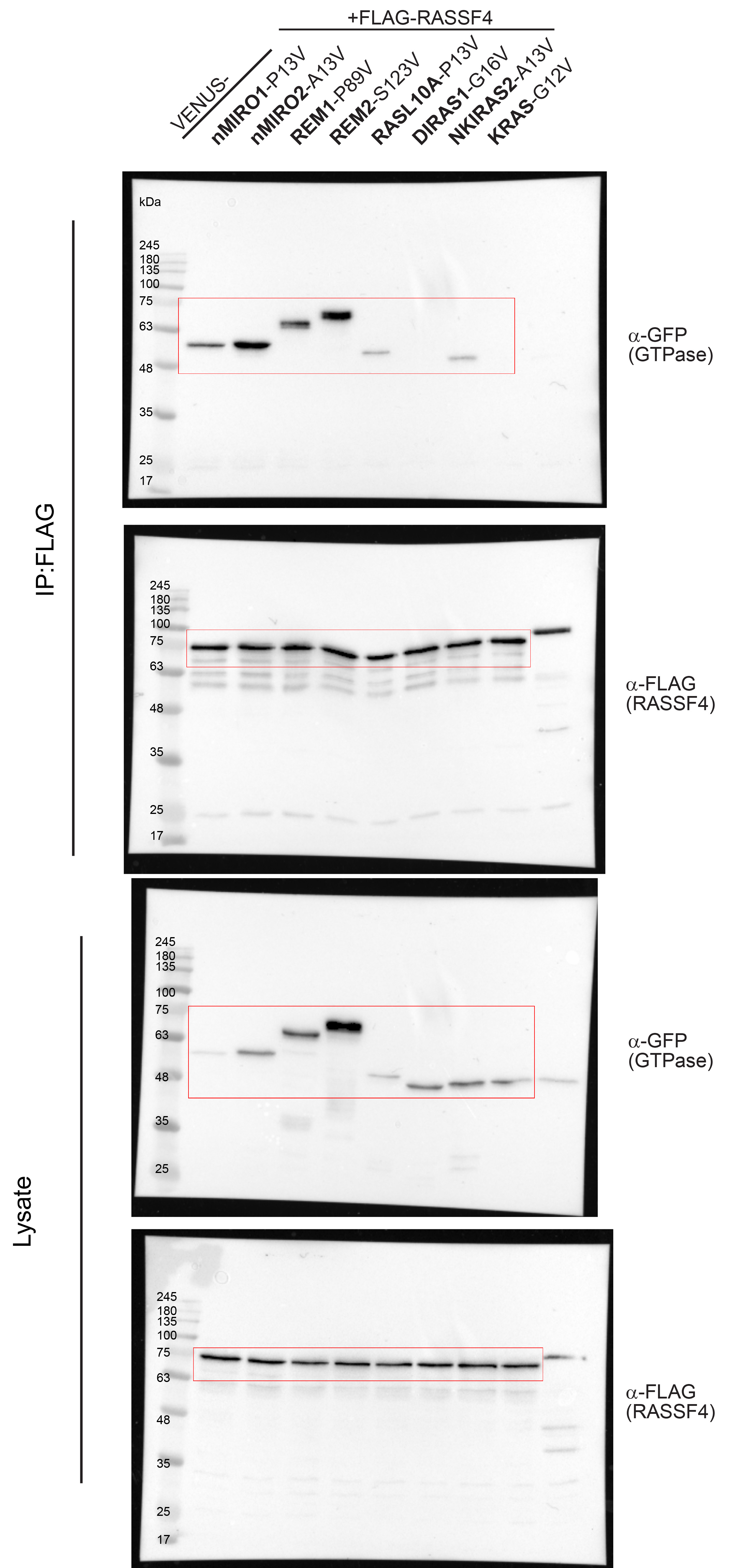

Supplement: Supplementary file 8 — Source data Fig. 6 [file 44319_2024_203_MOESM8_ESM.zip › 6A/RASSF4_IP/6A_RASSF4 IP.jpg]

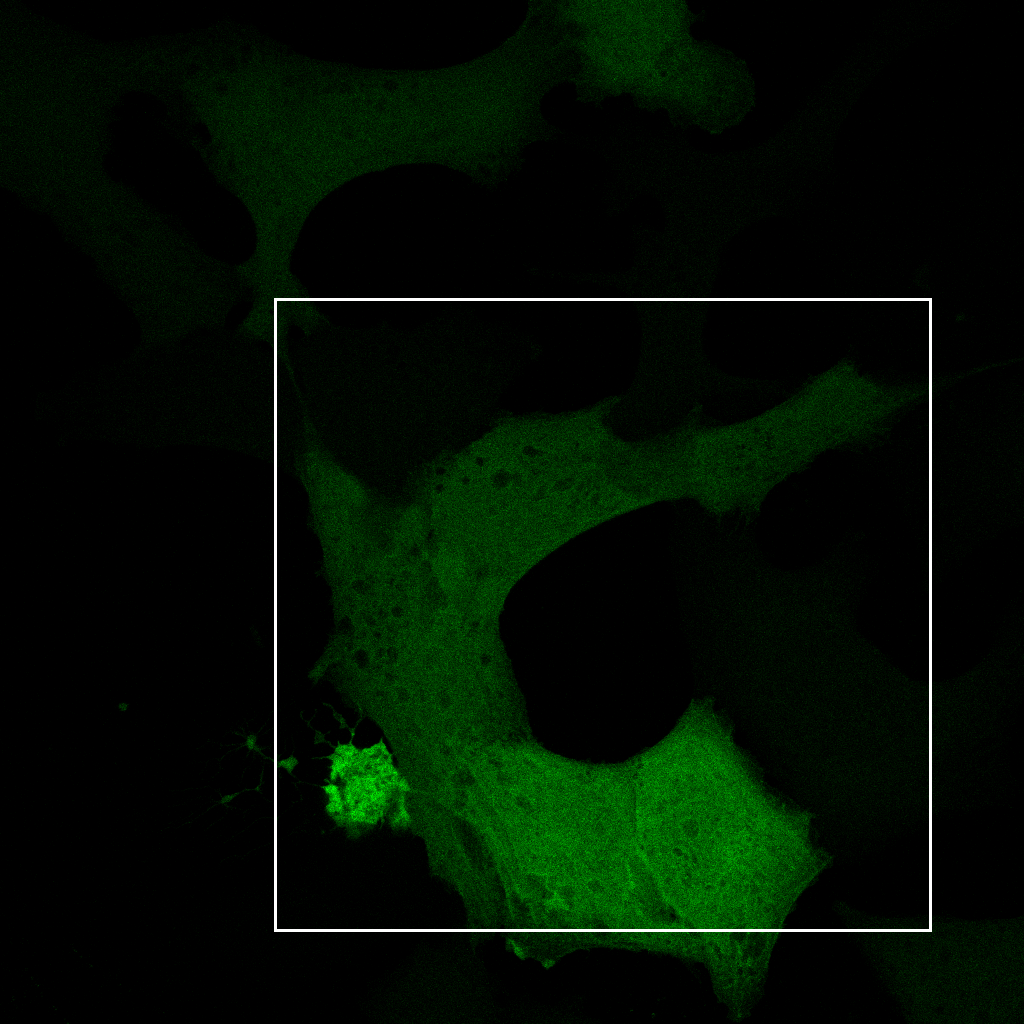

Supplement: Supplementary file 9 — Source data Fig. 7 [file 44319_2024_203_MOESM9_ESM.zip › 7D/GFPalone_mCherry-R3_Pex14/GFPalone.tif]

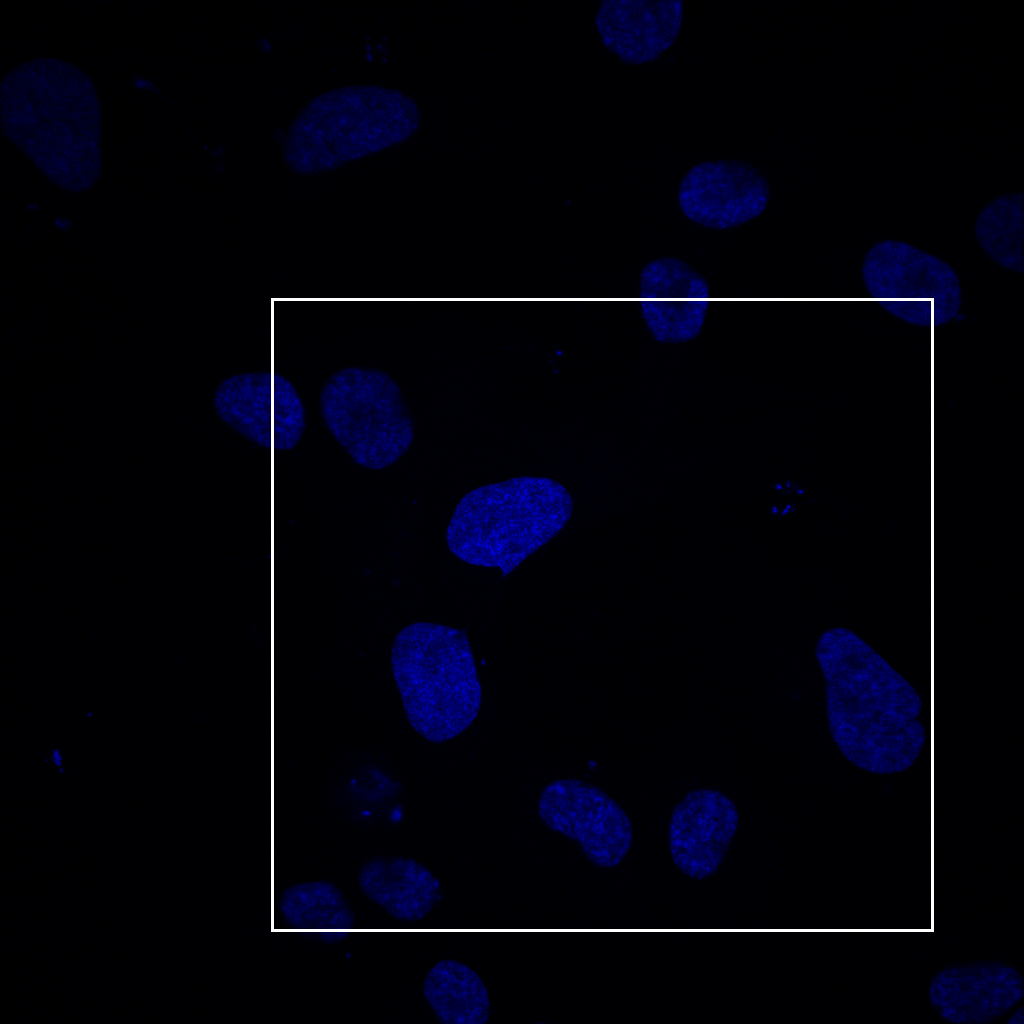

Supplement: Supplementary file 9 — Source data Fig. 7 [file 44319_2024_203_MOESM9_ESM.zip › 7D/GFPalone_mCherry-R3_Pex14/Hoechst.tif]

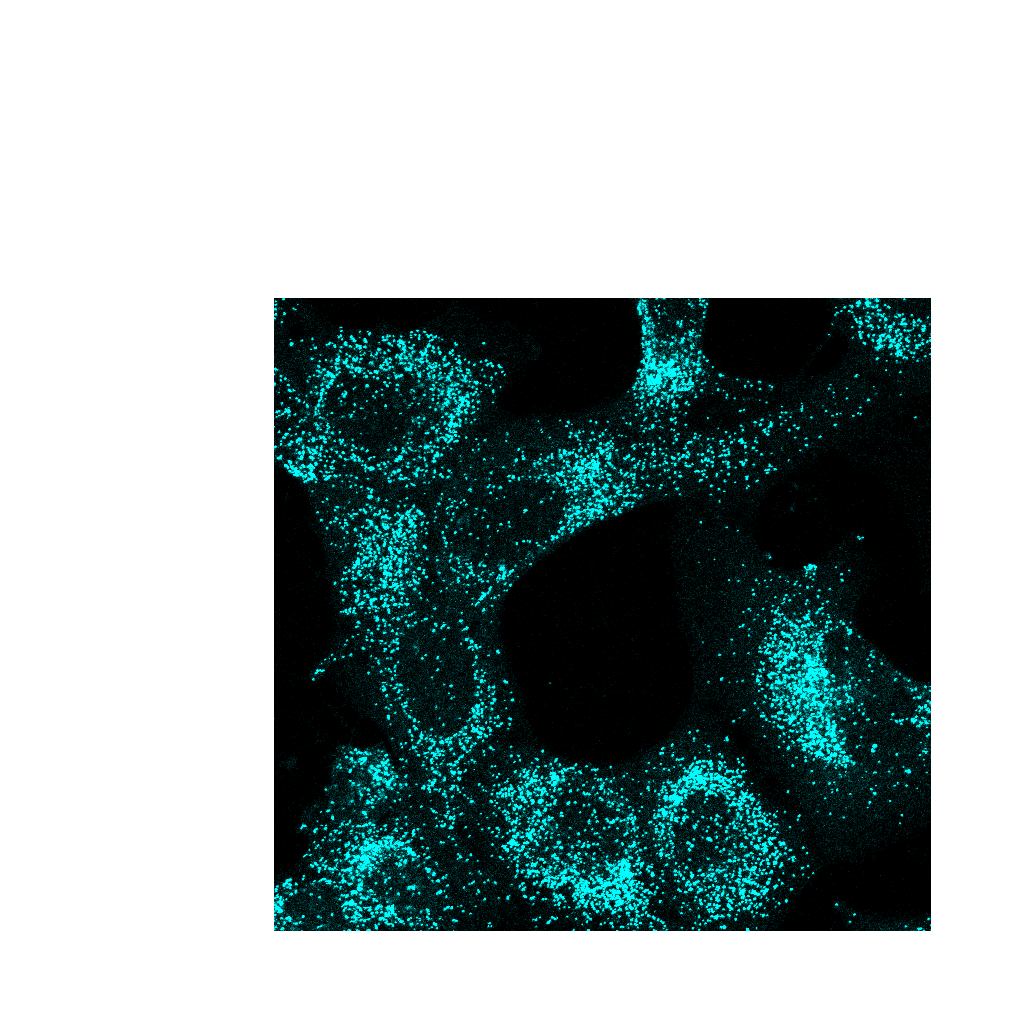

Supplement: Supplementary file 9 — Source data Fig. 7 [file 44319_2024_203_MOESM9_ESM.zip › 7D/GFPalone_mCherry-R3_Pex14/Pex14.tif]

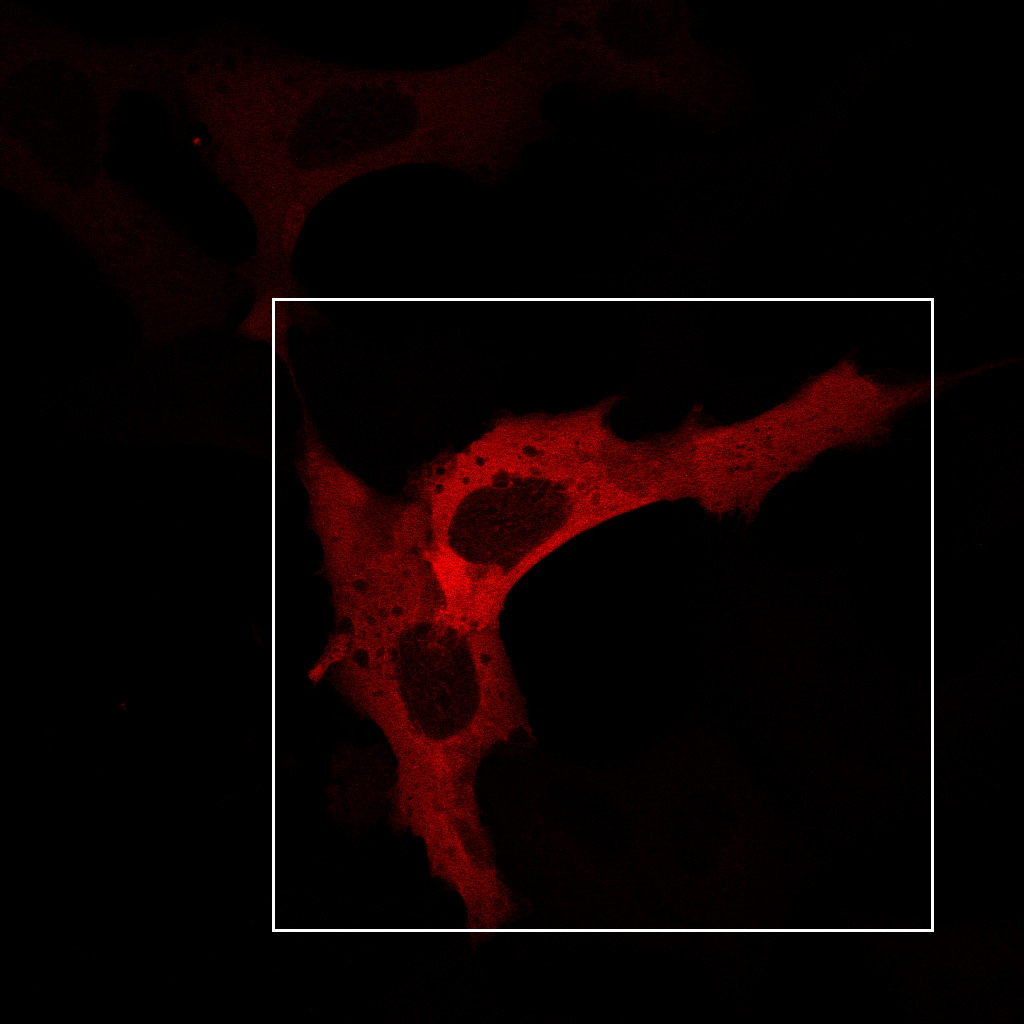

Supplement: Supplementary file 9 — Source data Fig. 7 [file 44319_2024_203_MOESM9_ESM.zip › 7D/GFPalone_mCherry-R3_Pex14/RASSF3.tif]

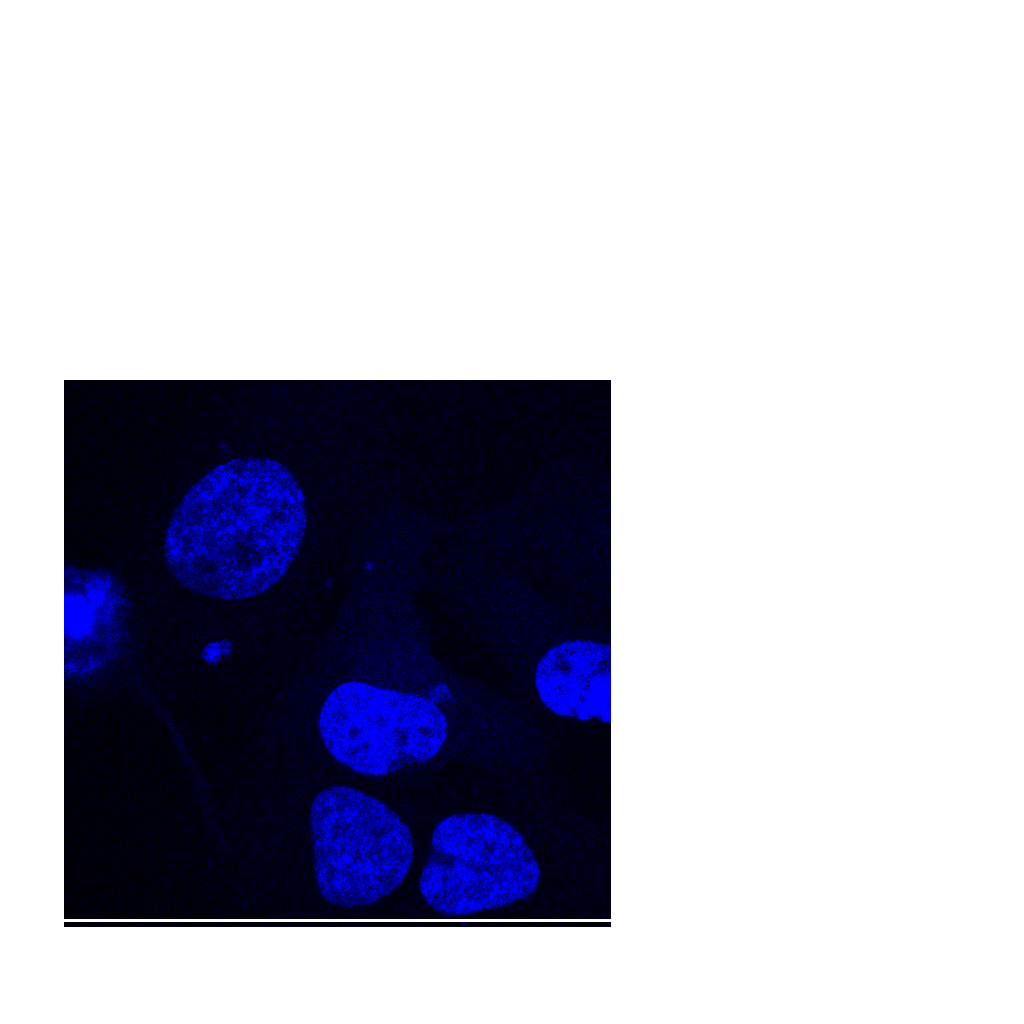

Supplement: Supplementary file 9 — Source data Fig. 7 [file 44319_2024_203_MOESM9_ESM.zip › 7D/MIRO1variant_mCherry-R3_Pex14/Hoechst.tif]

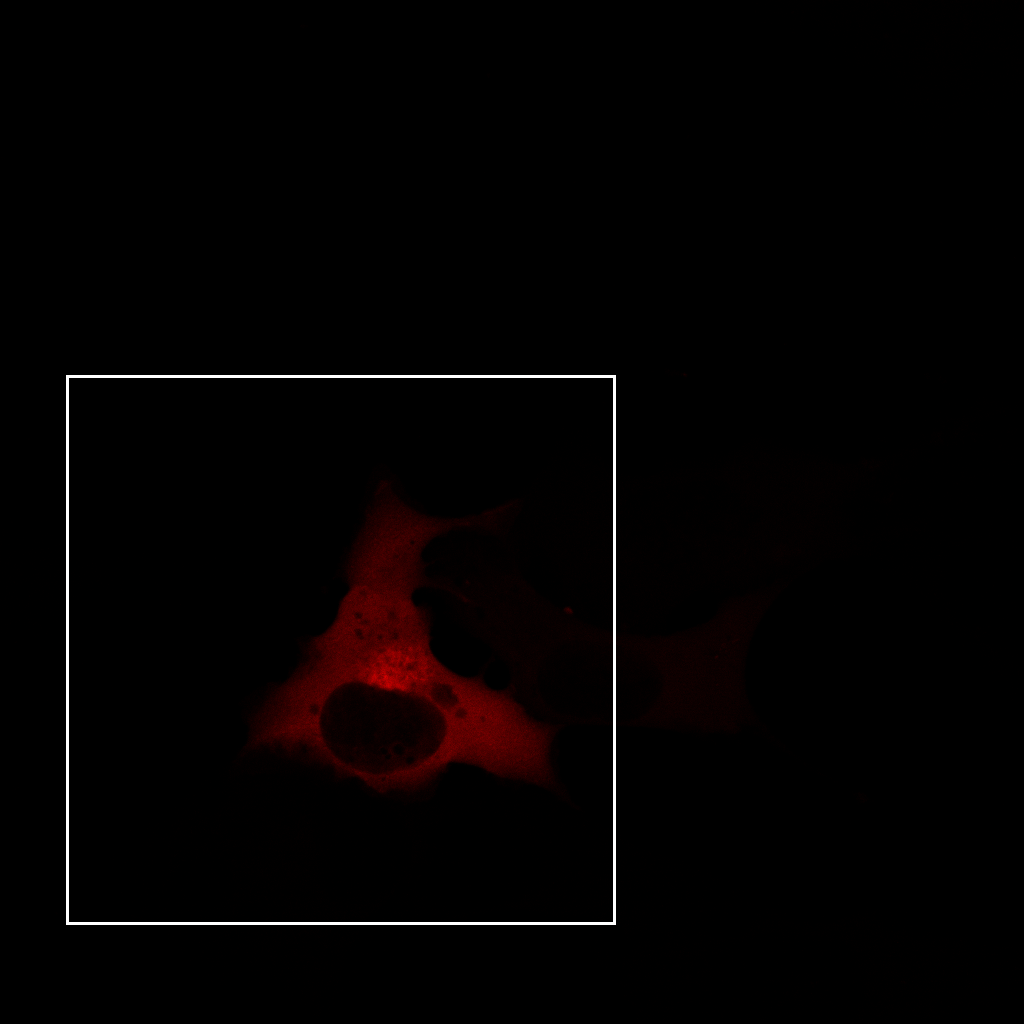

Supplement: Supplementary file 9 — Source data Fig. 7 [file 44319_2024_203_MOESM9_ESM.zip › 7D/MIRO1variant_mCherry-R3_Pex14/mCherry-RASSF3.tif]

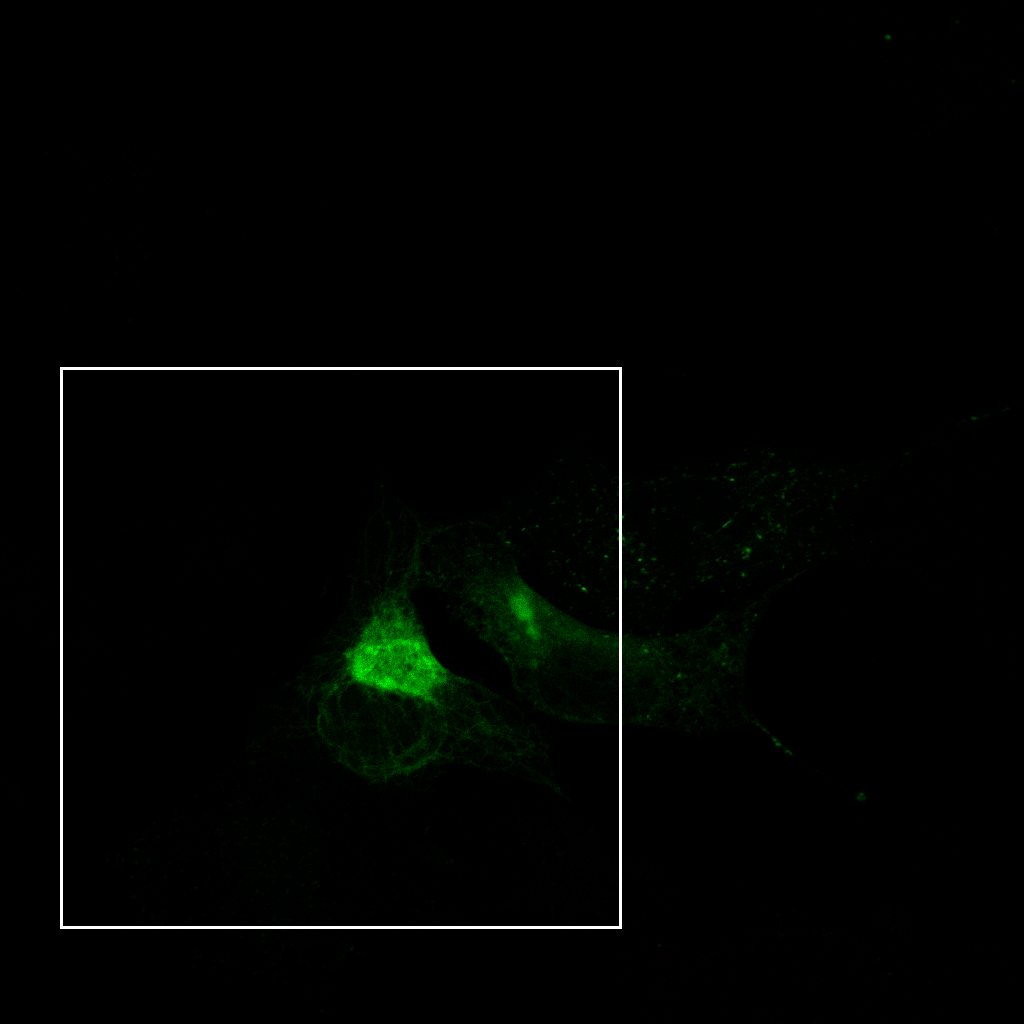

Supplement: Supplementary file 9 — Source data Fig. 7 [file 44319_2024_203_MOESM9_ESM.zip › 7D/MIRO1variant_mCherry-R3_Pex14/MIRO1variant.tif]

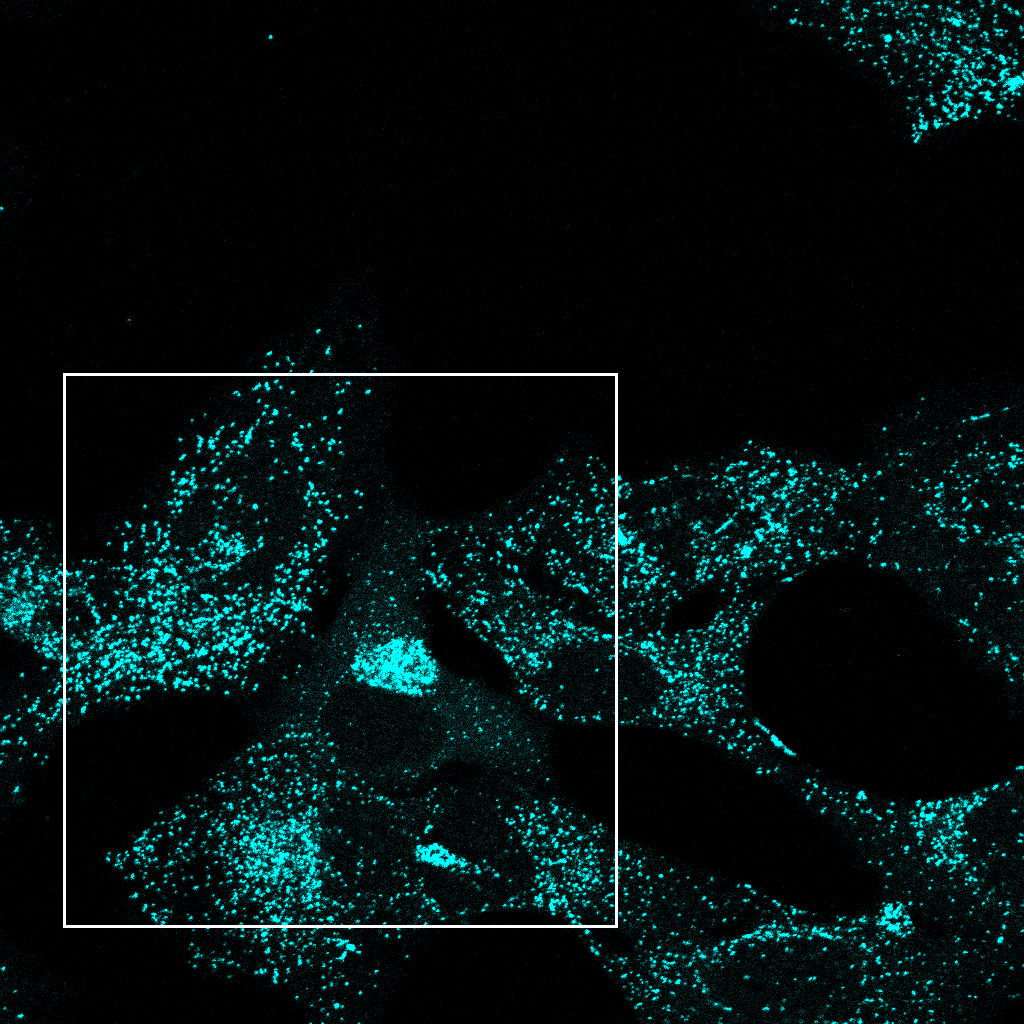

Supplement: Supplementary file 9 — Source data Fig. 7 [file 44319_2024_203_MOESM9_ESM.zip › 7D/MIRO1variant_mCherry-R3_Pex14/Pex14.tif]

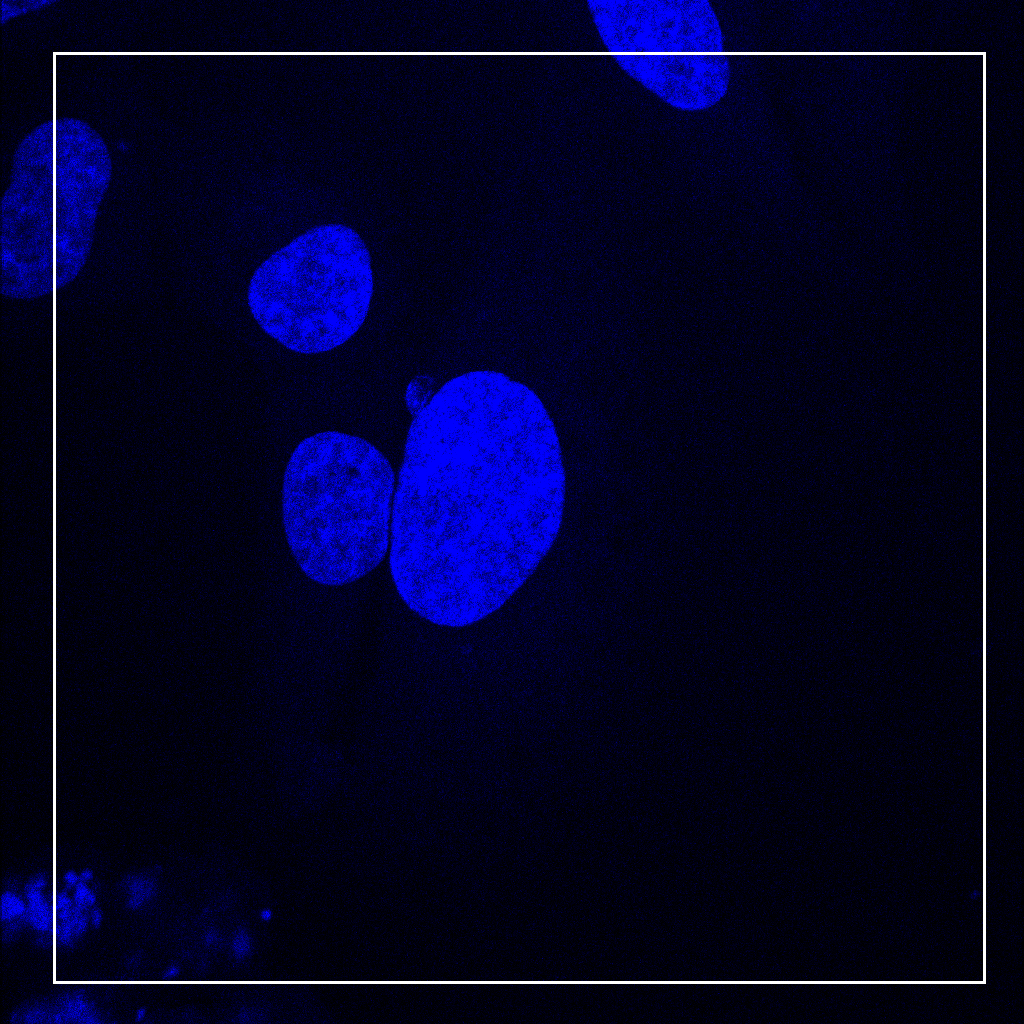

Supplement: Supplementary file 9 — Source data Fig. 7 [file 44319_2024_203_MOESM9_ESM.zip › 7D/MIRO1variant_mCherryAlone_Pex14/Hoechst.tif]

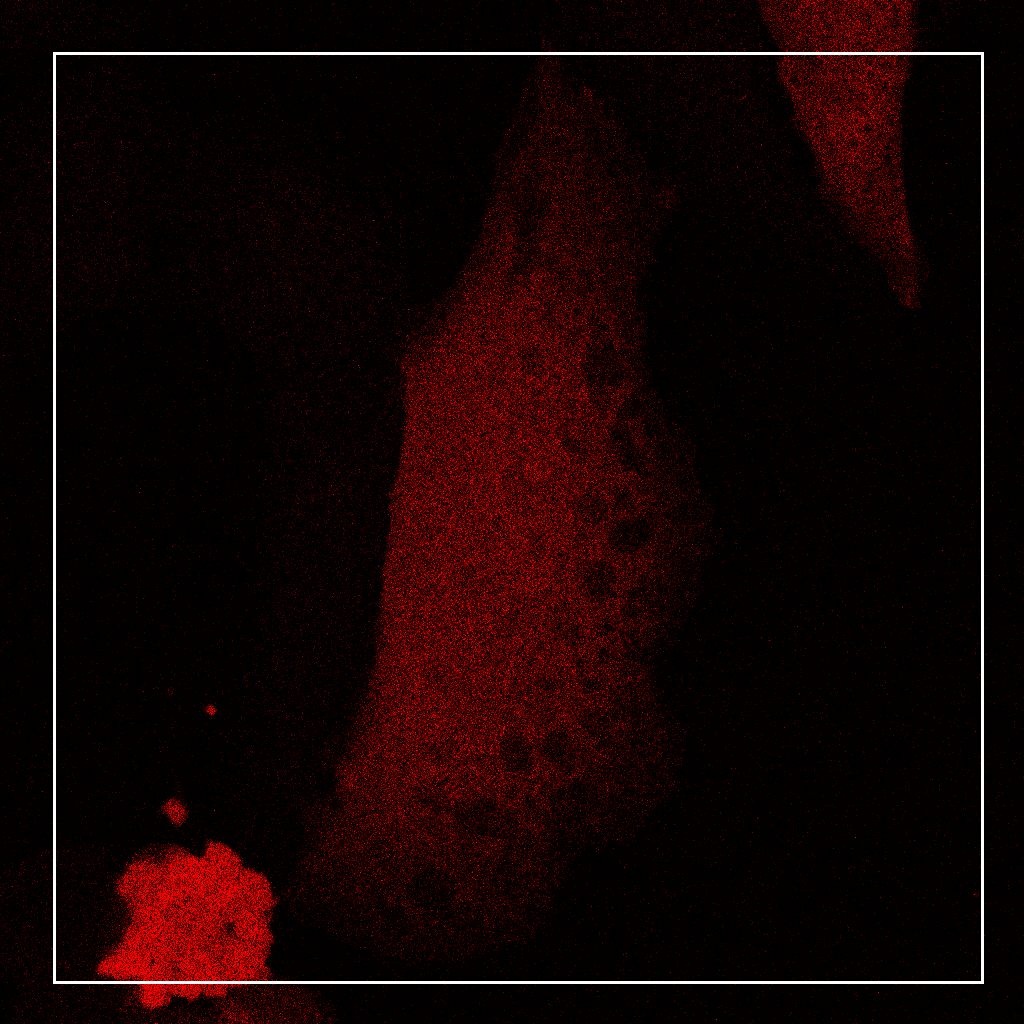

Supplement: Supplementary file 9 — Source data Fig. 7 [file 44319_2024_203_MOESM9_ESM.zip › 7D/MIRO1variant_mCherryAlone_Pex14/mCherry-alone.tif]

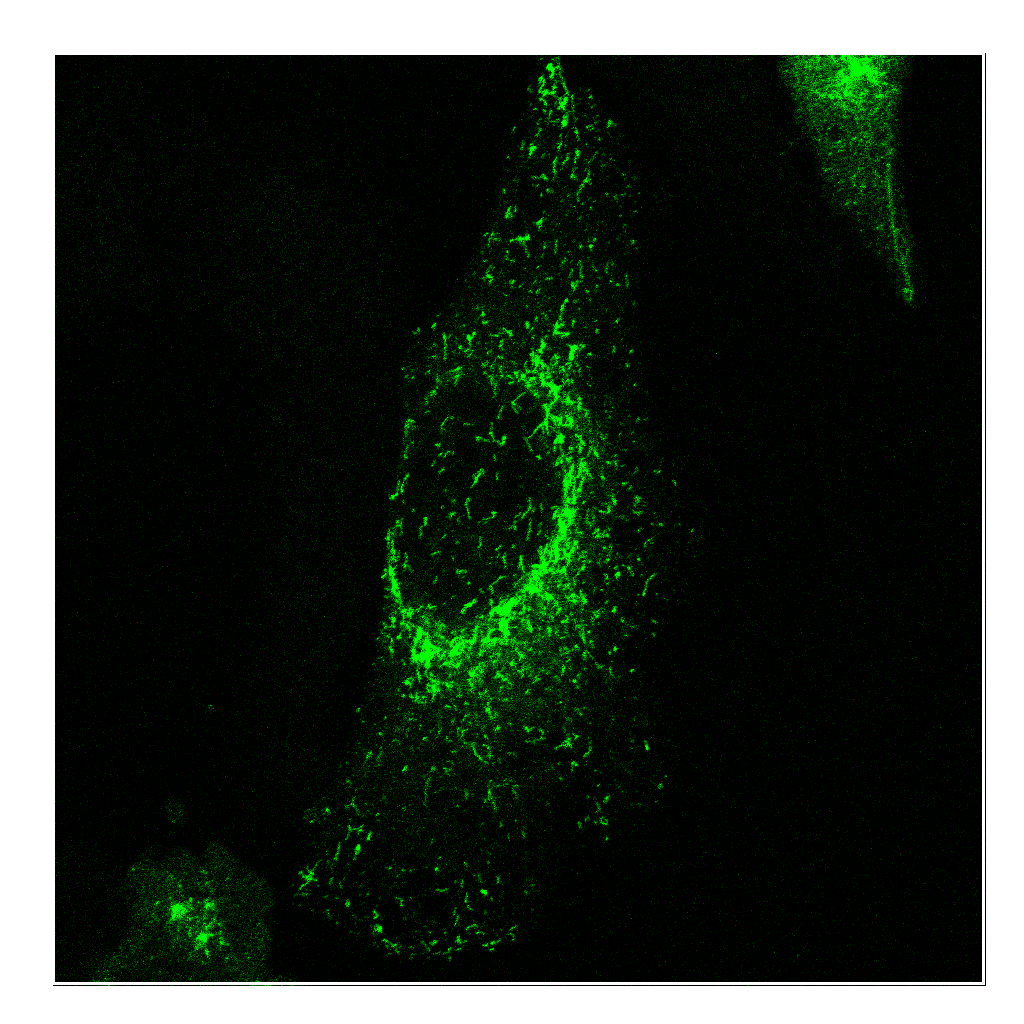

Supplement: Supplementary file 9 — Source data Fig. 7 [file 44319_2024_203_MOESM9_ESM.zip › 7D/MIRO1variant_mCherryAlone_Pex14/MIRO1variant.tif]

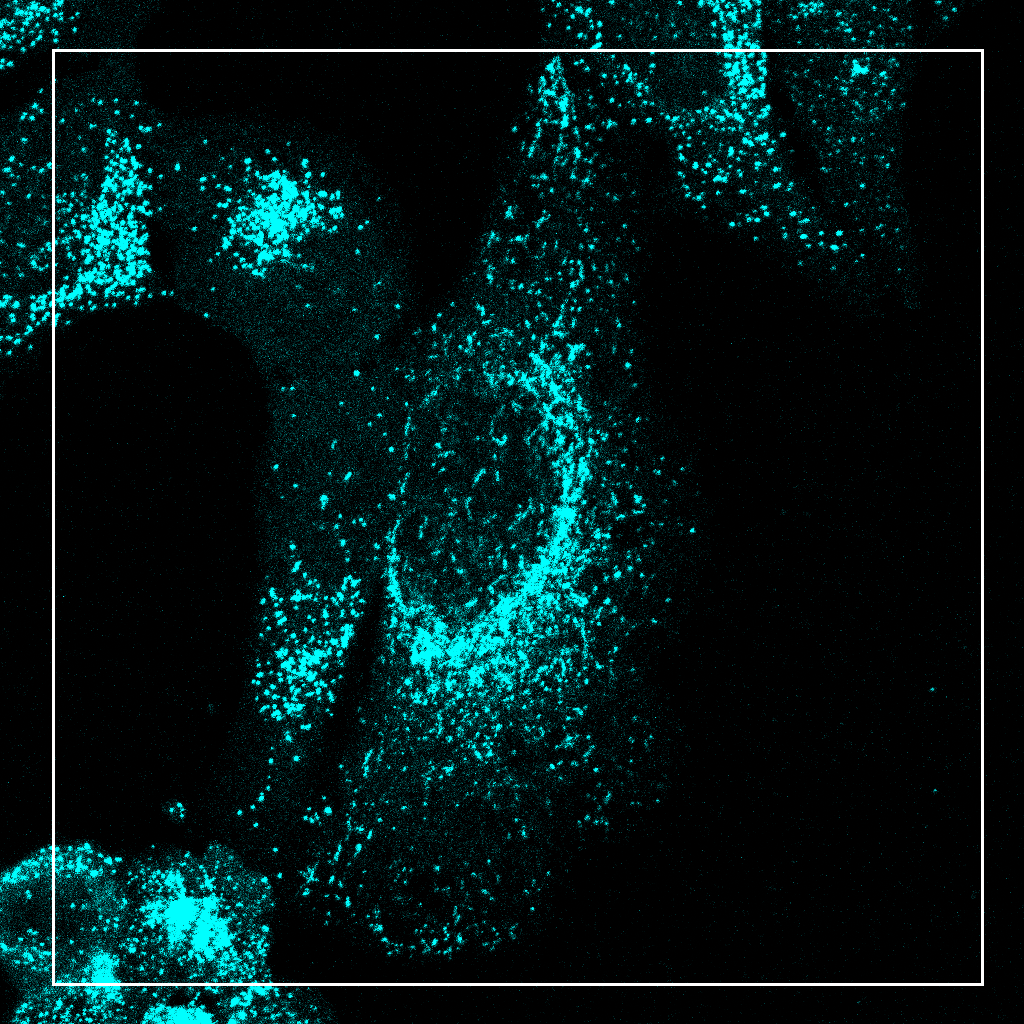

Supplement: Supplementary file 9 — Source data Fig. 7 [file 44319_2024_203_MOESM9_ESM.zip › 7D/MIRO1variant_mCherryAlone_Pex14/Pex14.tif]

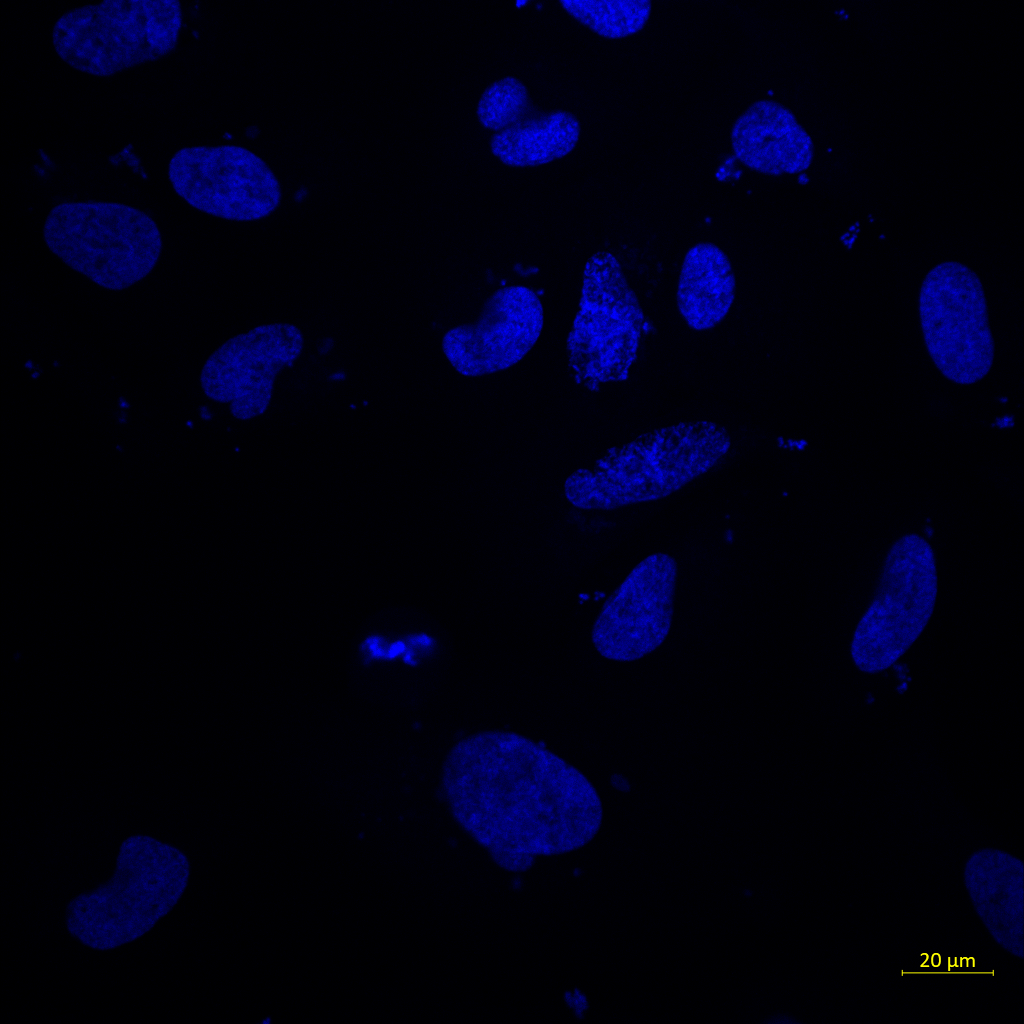

Supplement: Supplementary file 9 — Source data Fig. 7 [file 44319_2024_203_MOESM9_ESM.zip › 7A/Venus-alone+mCh-RASSF3/Hoechst.tif]

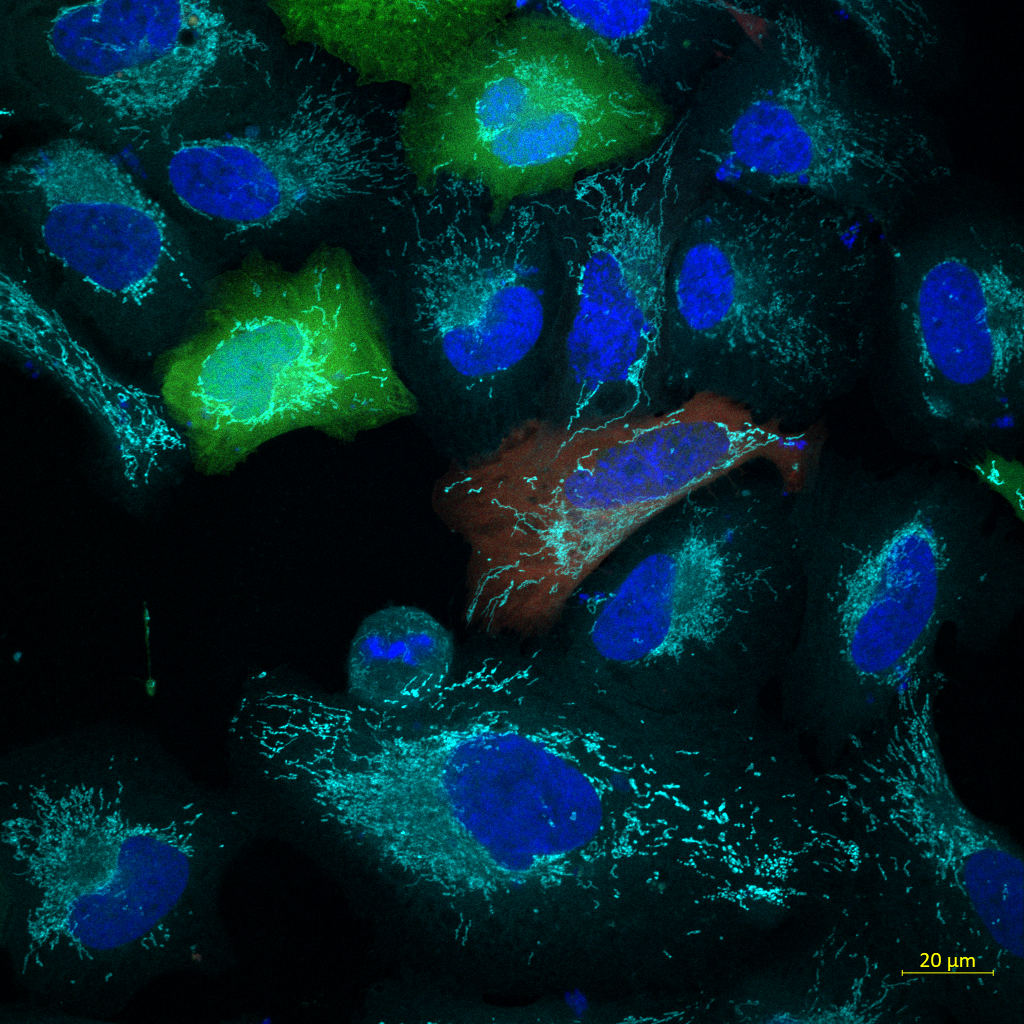

Supplement: Supplementary file 9 — Source data Fig. 7 [file 44319_2024_203_MOESM9_ESM.zip › 7A/Venus-alone+mCh-RASSF3/Merge.tif]

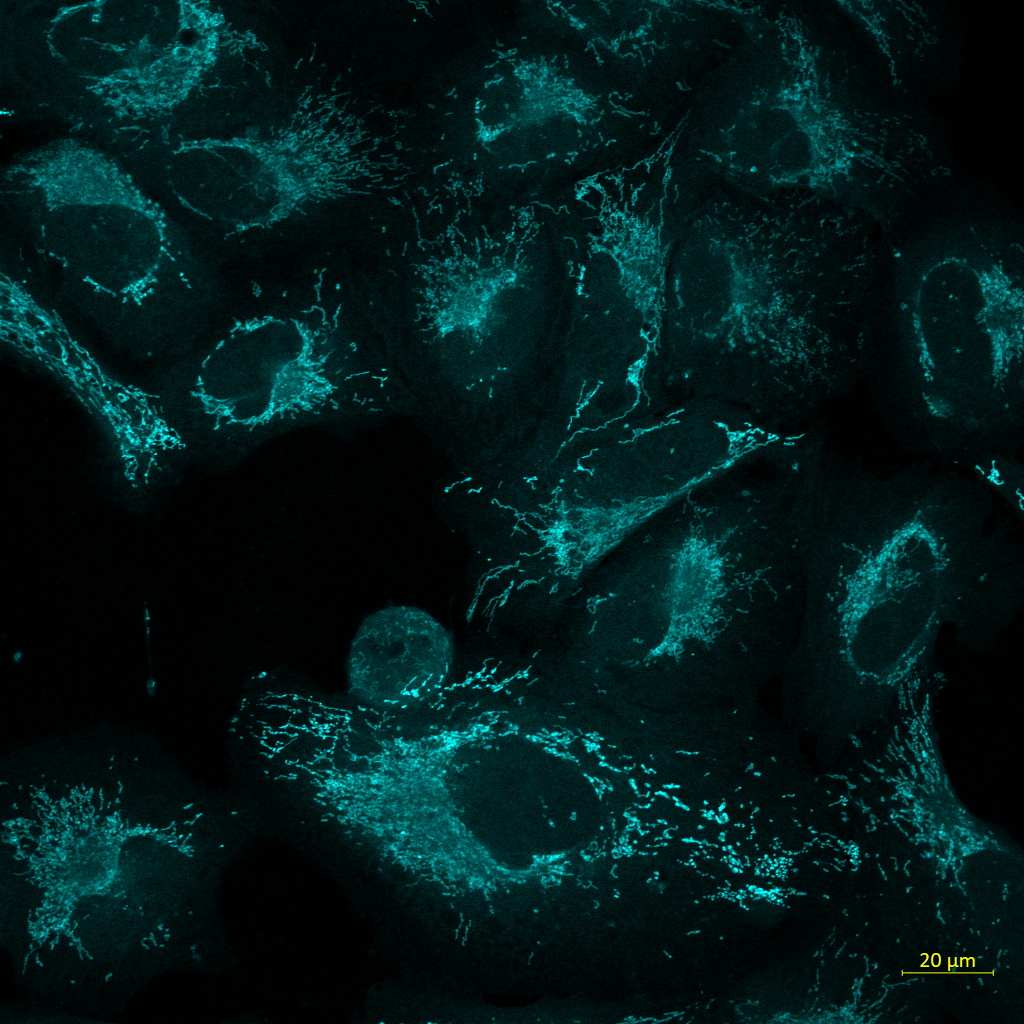

Supplement: Supplementary file 9 — Source data Fig. 7 [file 44319_2024_203_MOESM9_ESM.zip › 7A/Venus-alone+mCh-RASSF3/MitoTracker.tif]

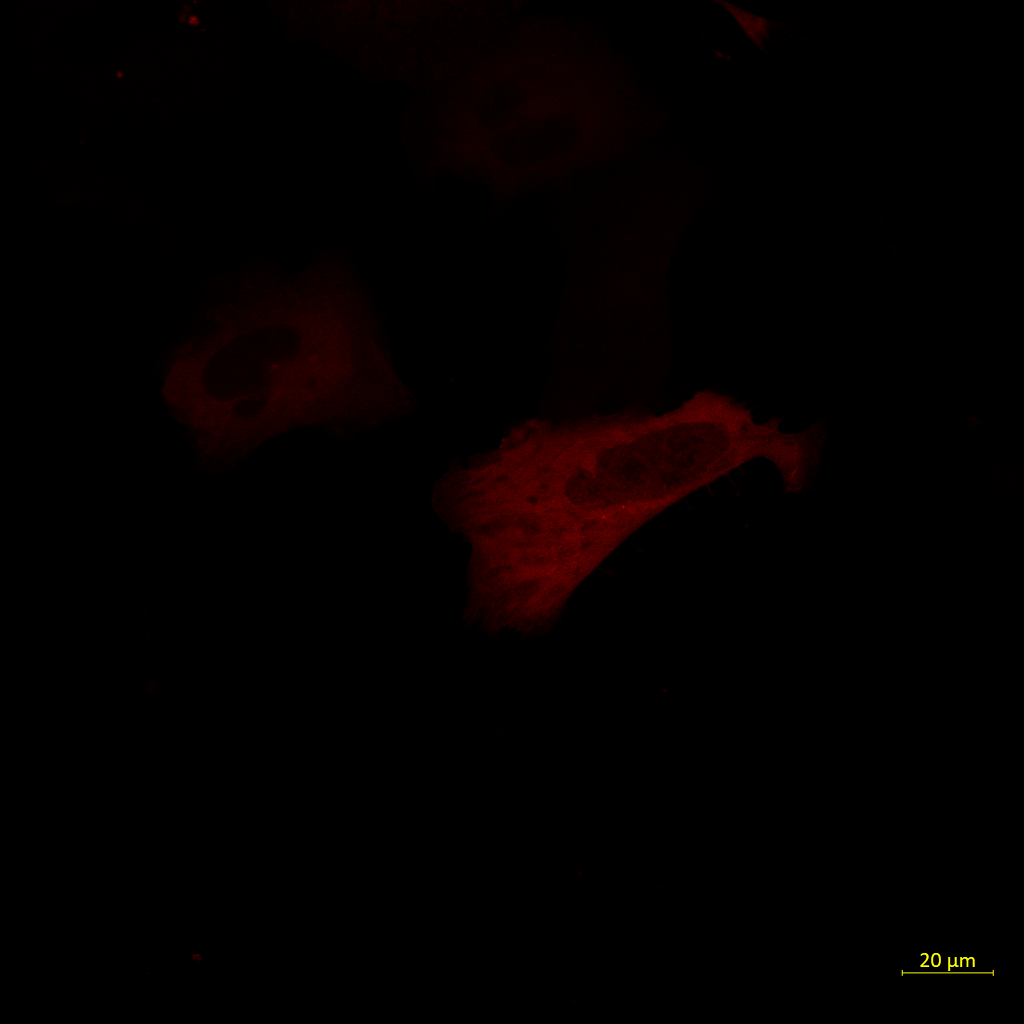

Supplement: Supplementary file 9 — Source data Fig. 7 [file 44319_2024_203_MOESM9_ESM.zip › 7A/Venus-alone+mCh-RASSF3/RASSF3.tif]

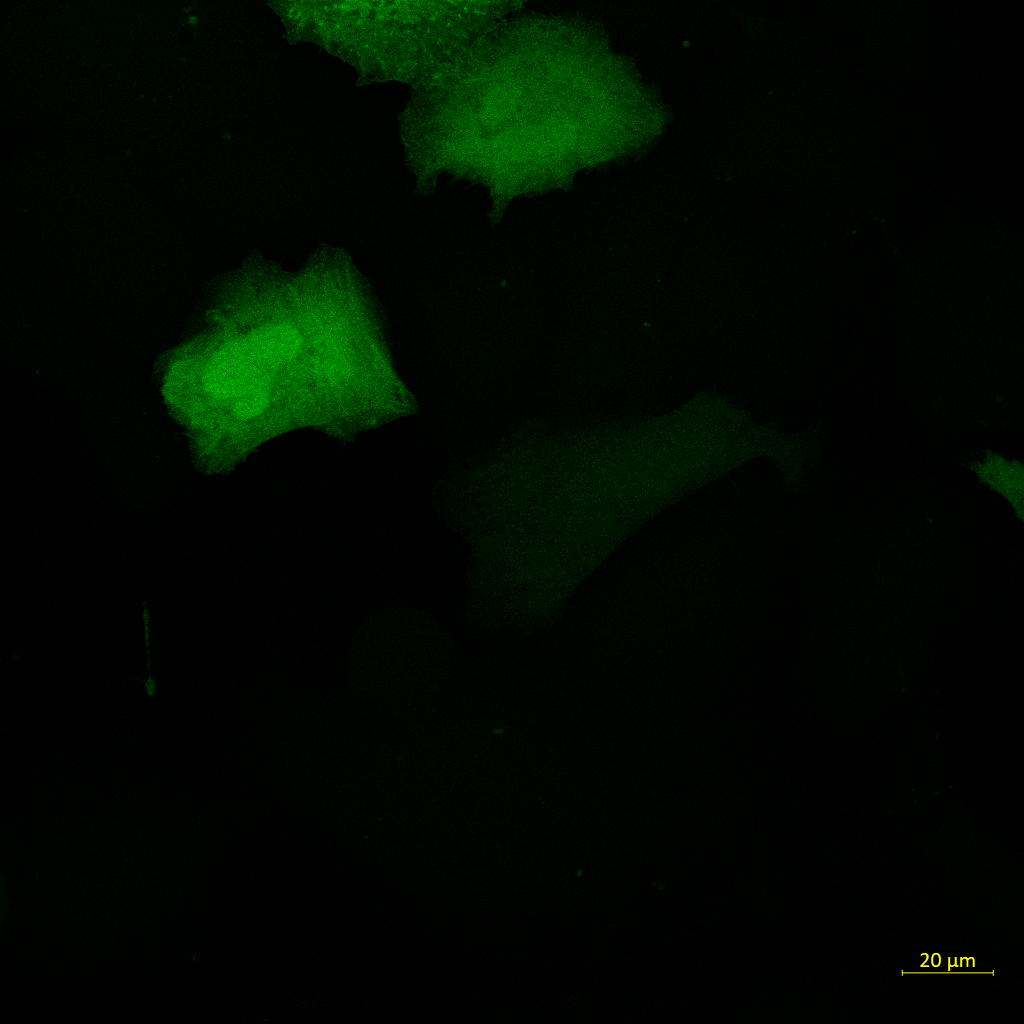

Supplement: Supplementary file 9 — Source data Fig. 7 [file 44319_2024_203_MOESM9_ESM.zip › 7A/Venus-alone+mCh-RASSF3/Venus alone.tif]

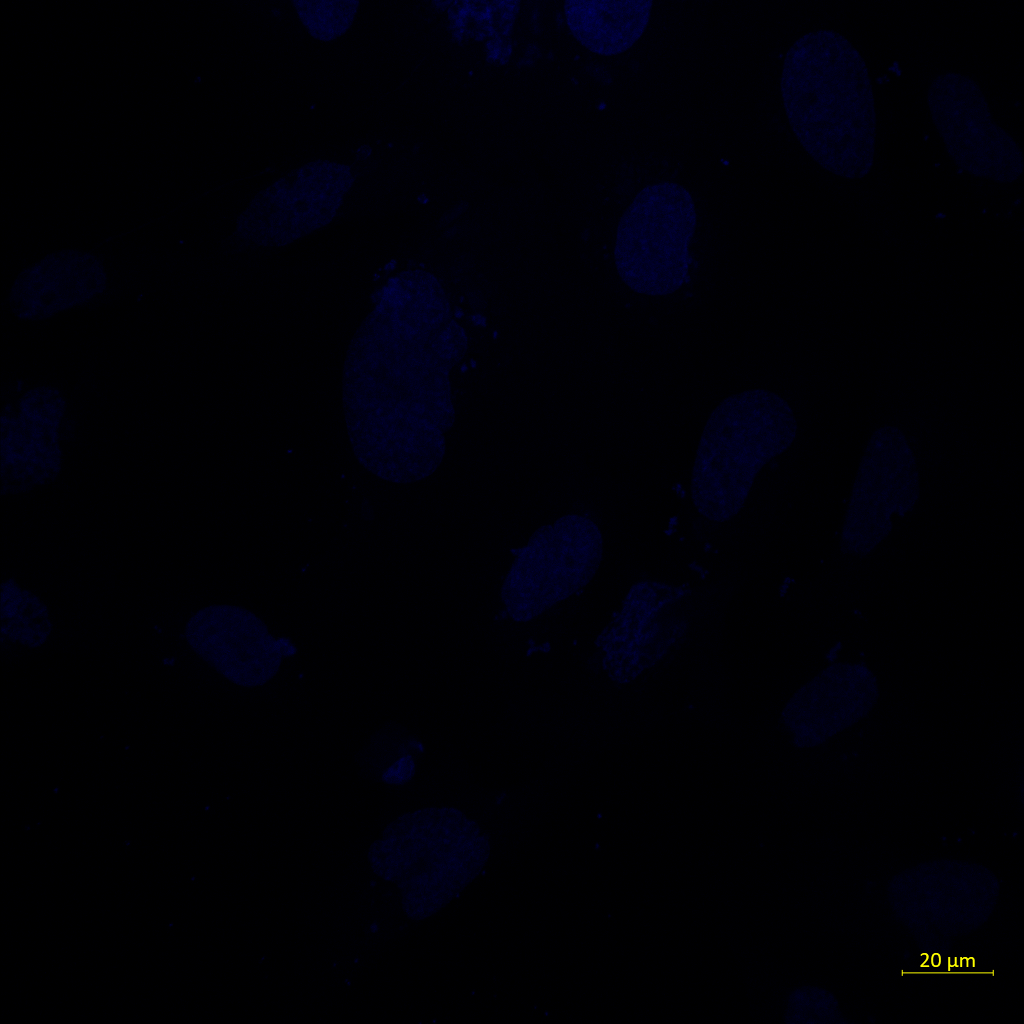

Supplement: Supplementary file 9 — Source data Fig. 7 [file 44319_2024_203_MOESM9_ESM.zip › 7A/Venus-MIRO1+mCherry/Hoechst.tif]

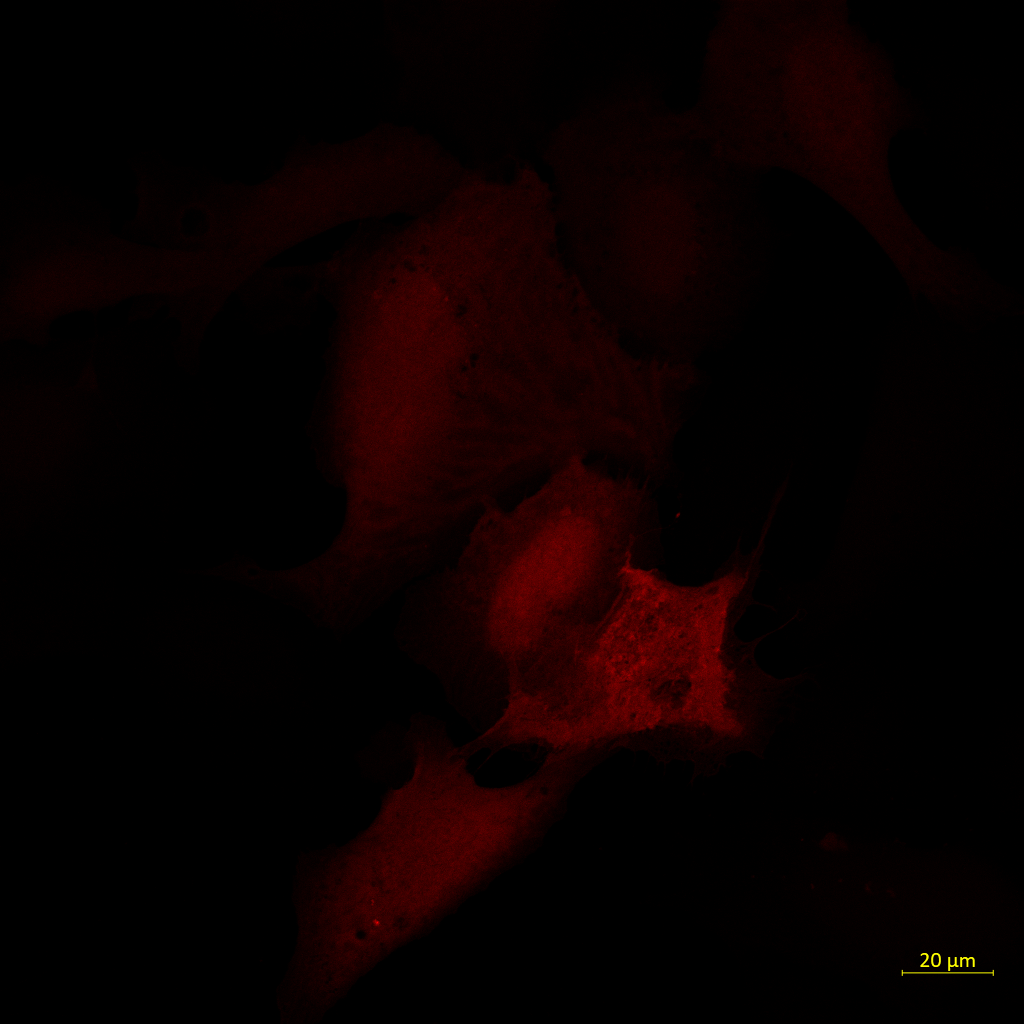

Supplement: Supplementary file 9 — Source data Fig. 7 [file 44319_2024_203_MOESM9_ESM.zip › 7A/Venus-MIRO1+mCherry/mCherry alone.tif]

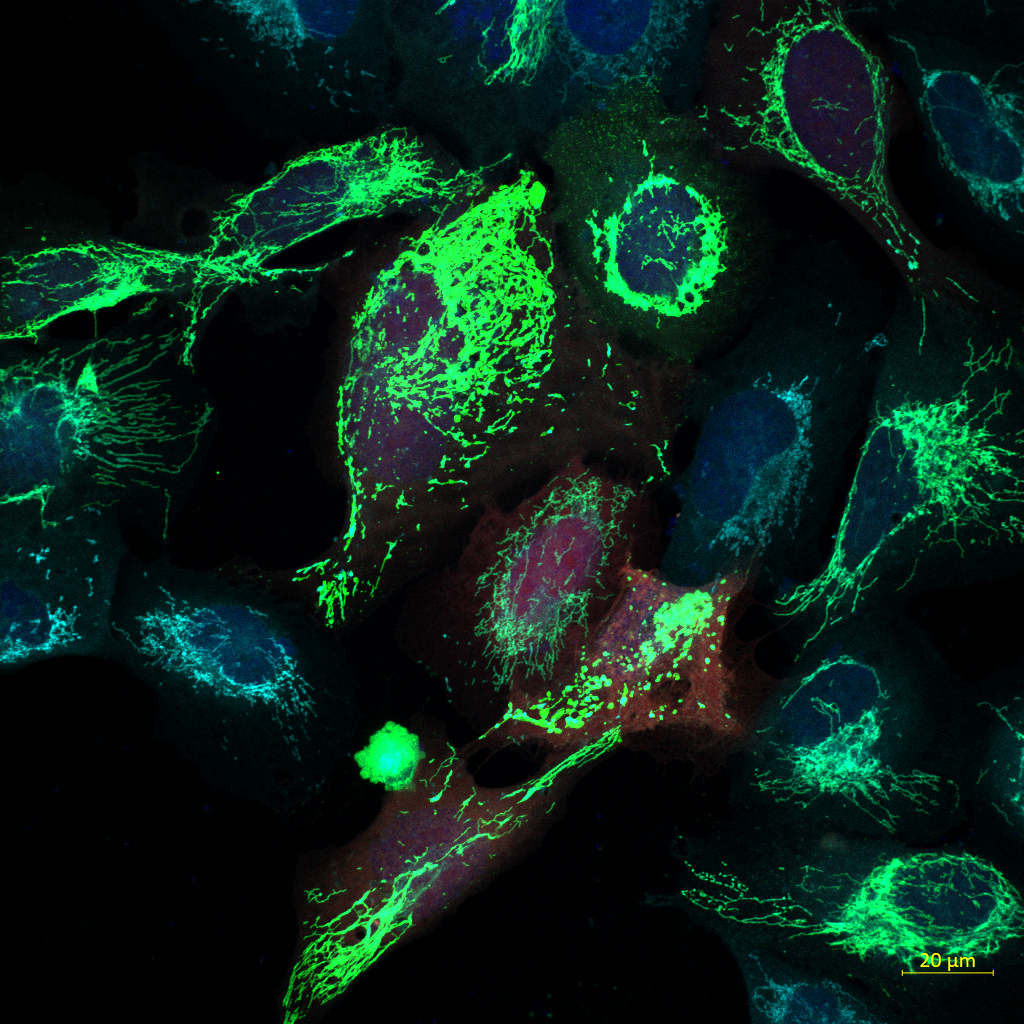

Supplement: Supplementary file 9 — Source data Fig. 7 [file 44319_2024_203_MOESM9_ESM.zip › 7A/Venus-MIRO1+mCherry/Merge.tif]

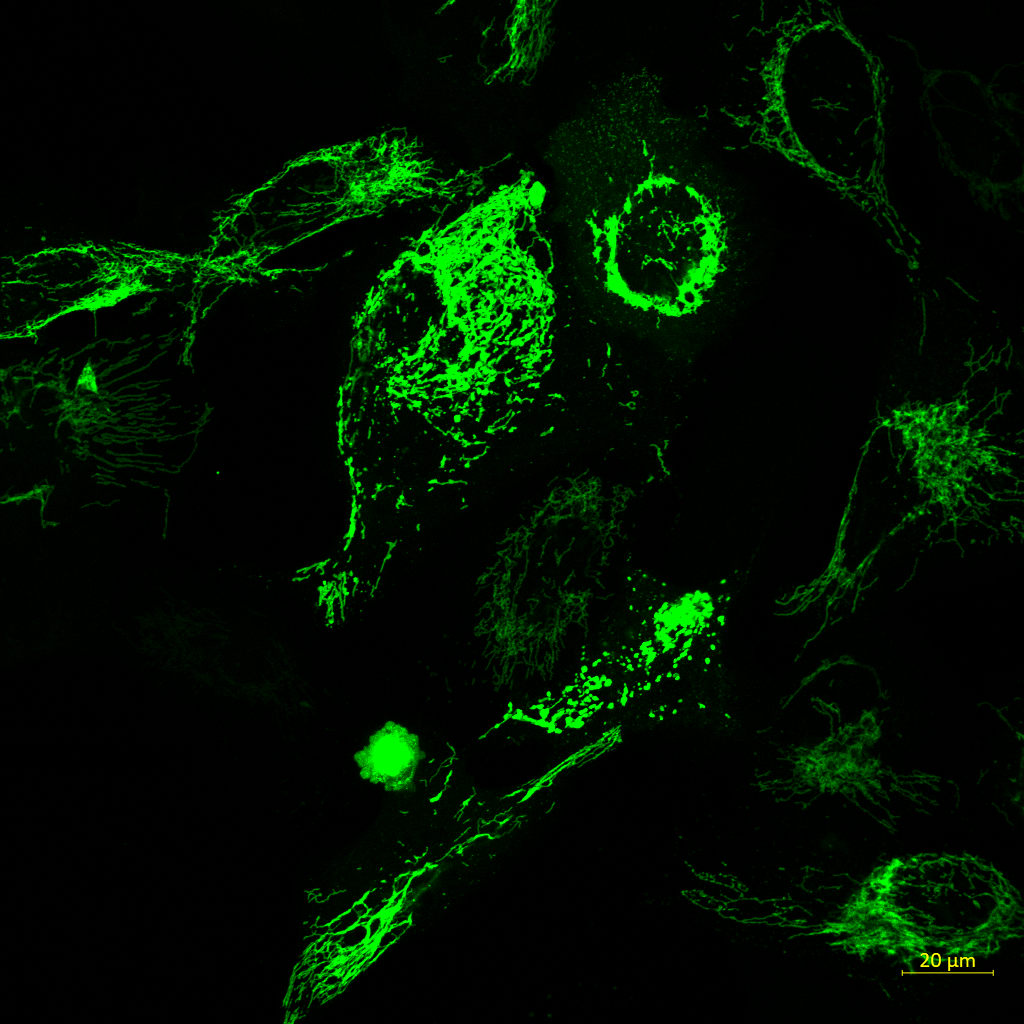

Supplement: Supplementary file 9 — Source data Fig. 7 [file 44319_2024_203_MOESM9_ESM.zip › 7A/Venus-MIRO1+mCherry/MIRO1.tif]

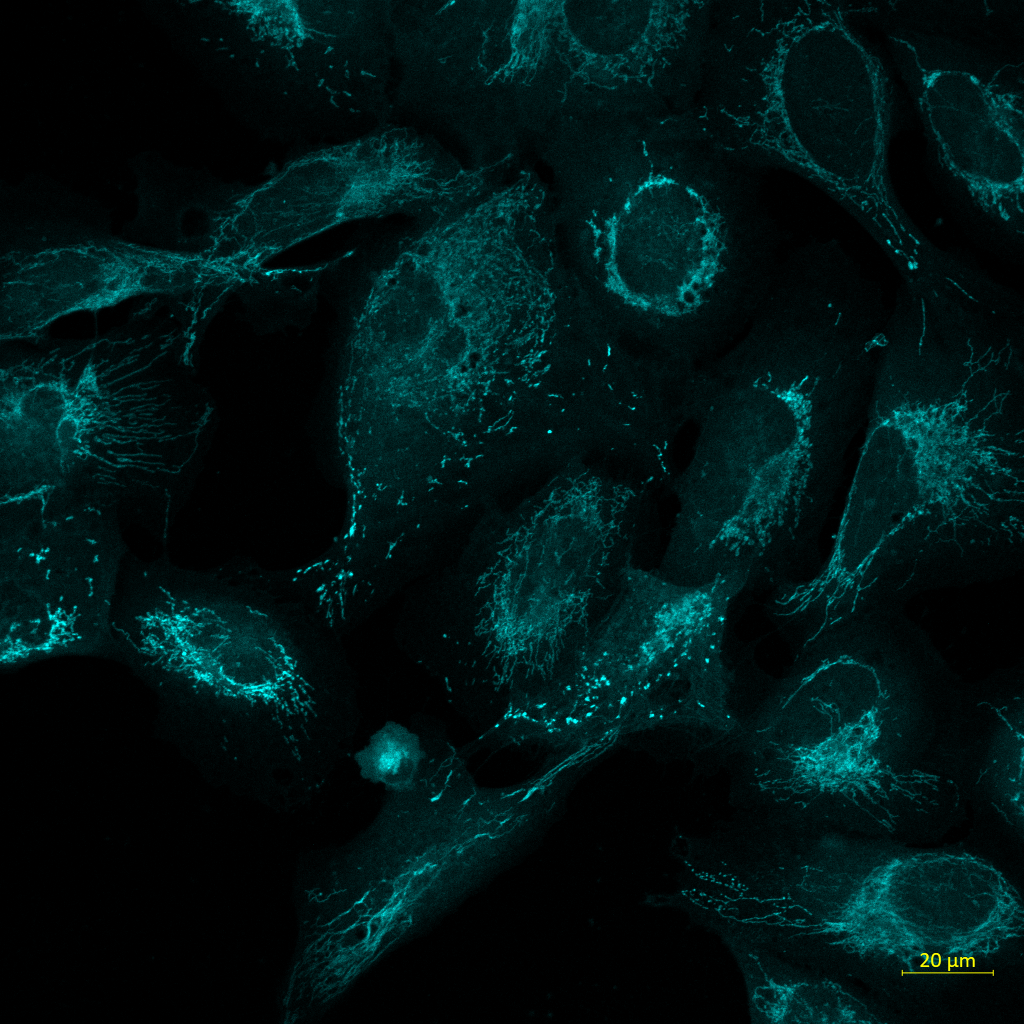

Supplement: Supplementary file 9 — Source data Fig. 7 [file 44319_2024_203_MOESM9_ESM.zip › 7A/Venus-MIRO1+mCherry/MitoTracker.tif]

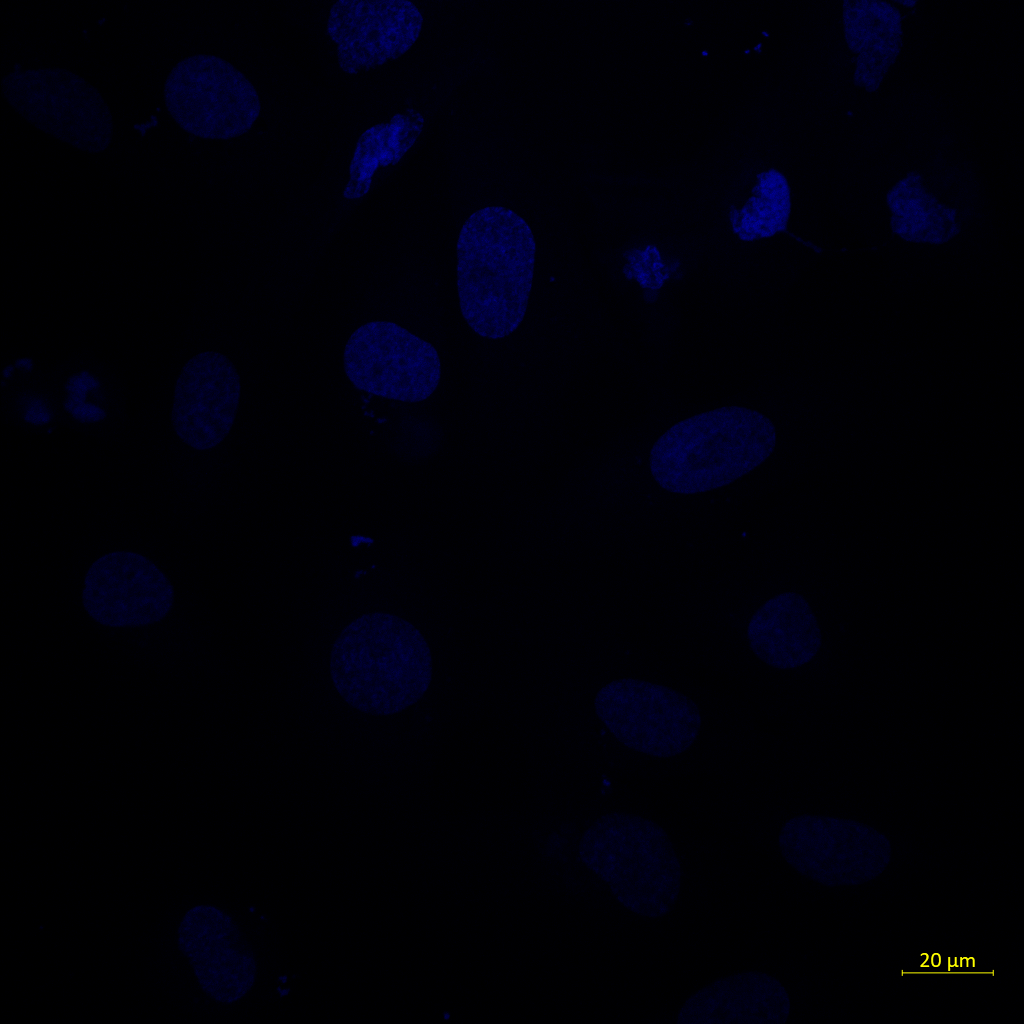

Supplement: Supplementary file 9 — Source data Fig. 7 [file 44319_2024_203_MOESM9_ESM.zip › 7A/Venus-MIRO2+mCH-RASSF3/Hoechst.tif]

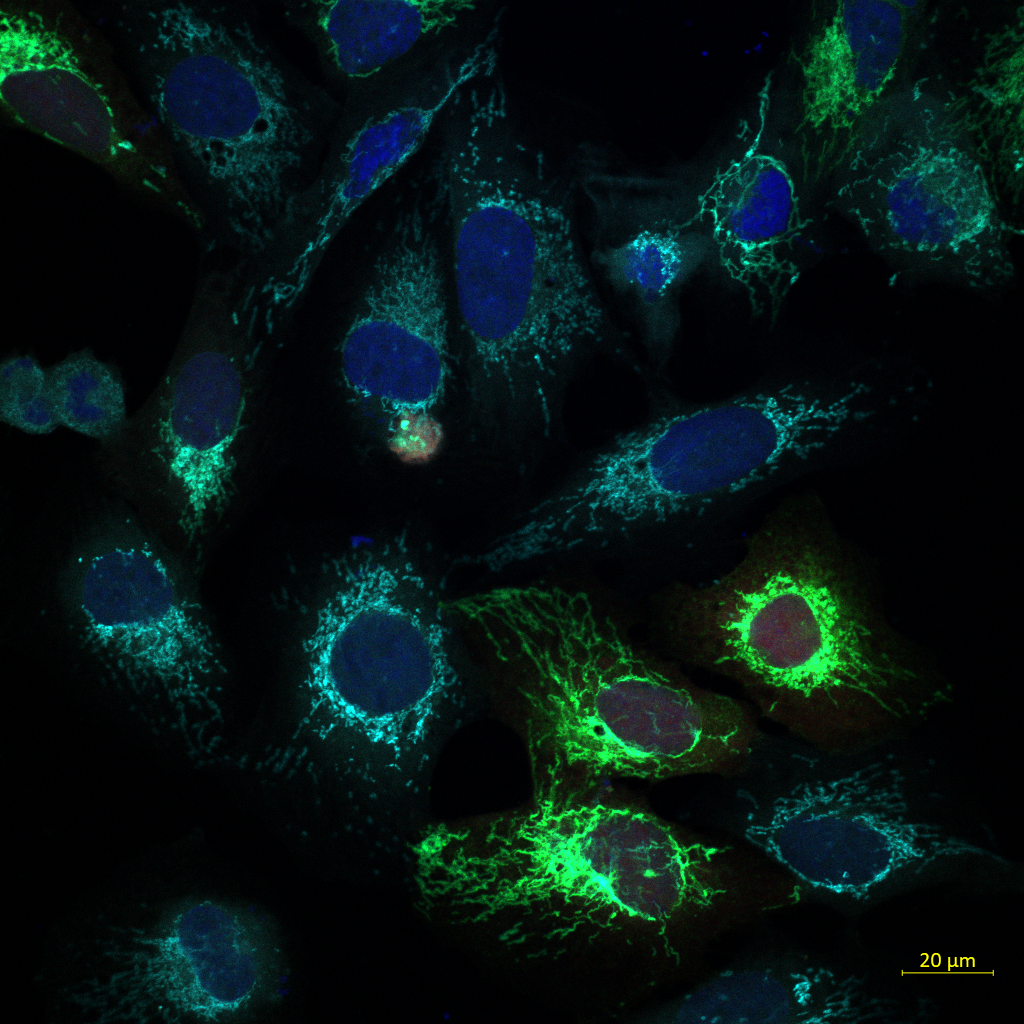

Supplement: Supplementary file 9 — Source data Fig. 7 [file 44319_2024_203_MOESM9_ESM.zip › 7A/Venus-MIRO2+mCH-RASSF3/Merge.tif]

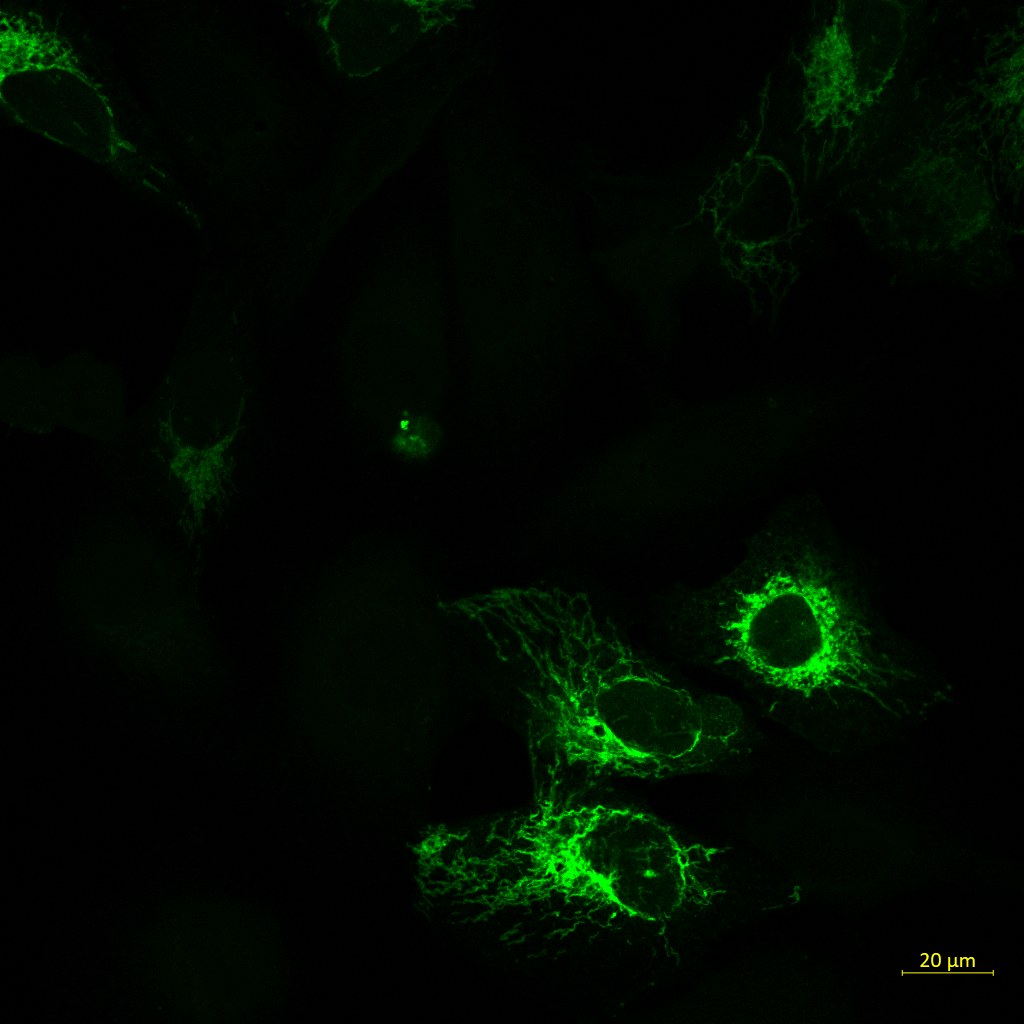

Supplement: Supplementary file 9 — Source data Fig. 7 [file 44319_2024_203_MOESM9_ESM.zip › 7A/Venus-MIRO2+mCH-RASSF3/MIRO2.tif]

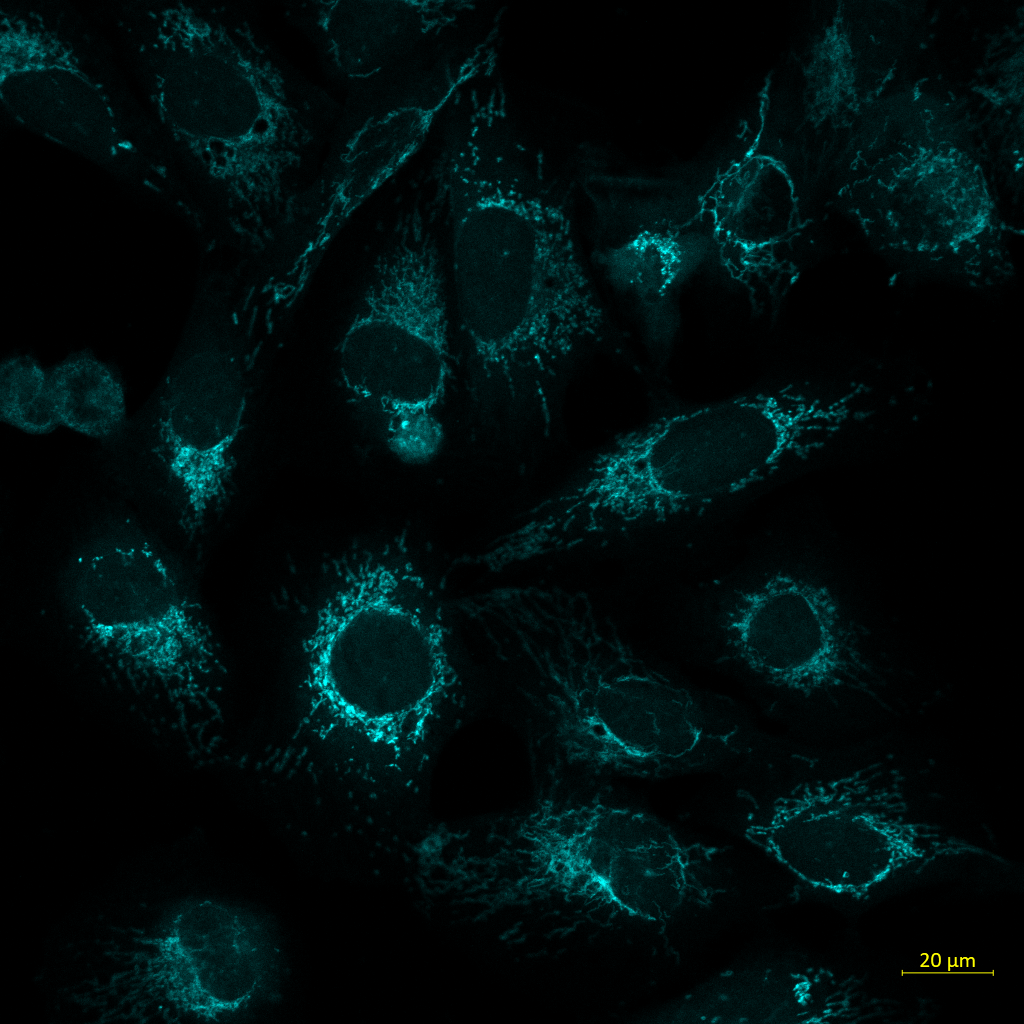

Supplement: Supplementary file 9 — Source data Fig. 7 [file 44319_2024_203_MOESM9_ESM.zip › 7A/Venus-MIRO2+mCH-RASSF3/MitoTracker.tif]

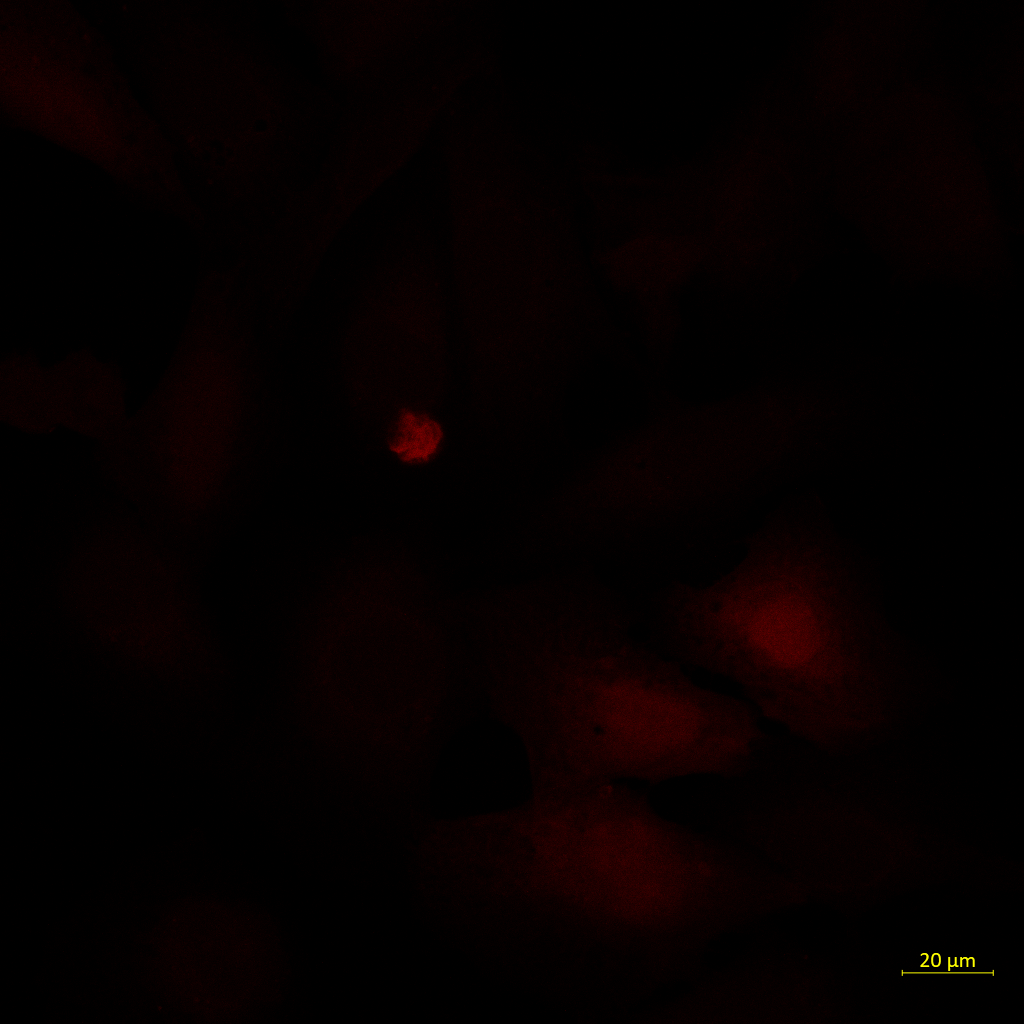

Supplement: Supplementary file 9 — Source data Fig. 7 [file 44319_2024_203_MOESM9_ESM.zip › 7A/Venus-MIRO2+mCH-RASSF3/RASSF3.tif]

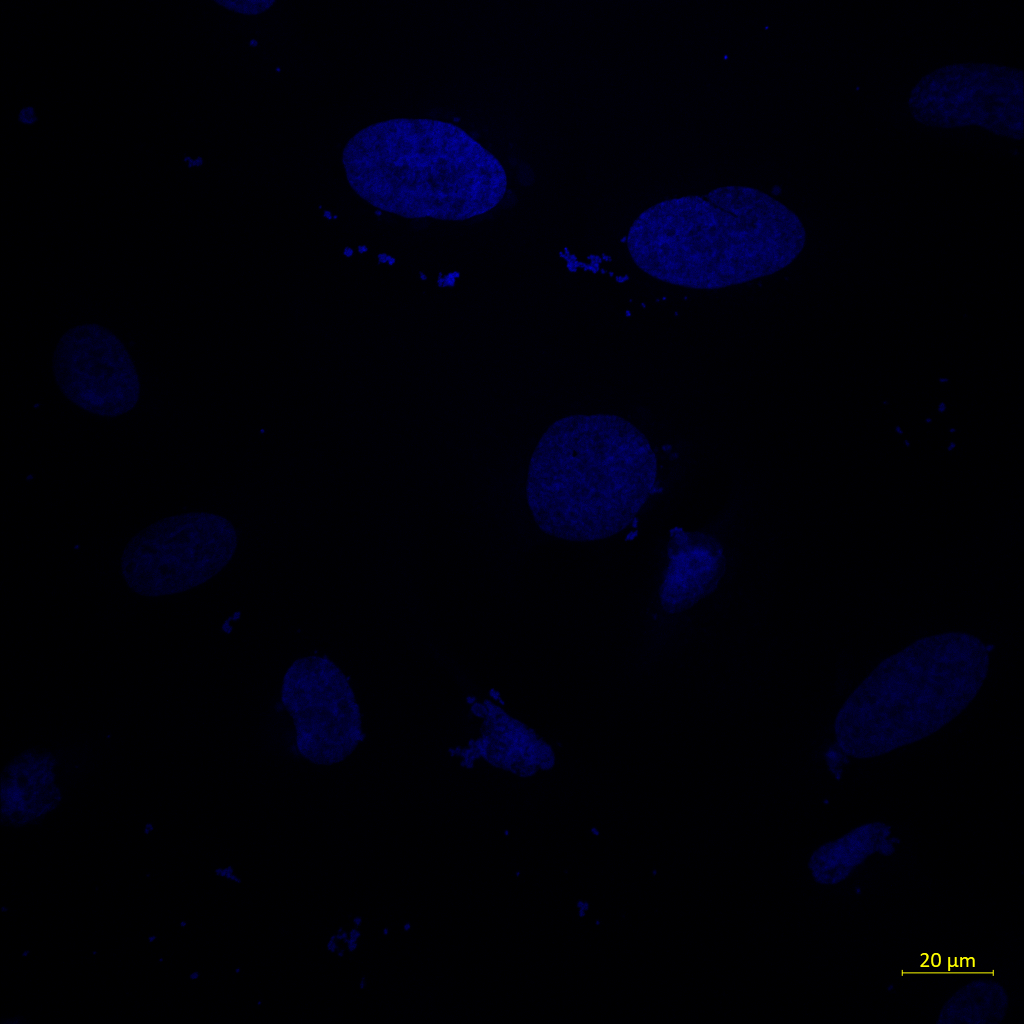

Supplement: Supplementary file 9 — Source data Fig. 7 [file 44319_2024_203_MOESM9_ESM.zip › 7A/VenusMiro1WT+mCh-RASFF3/Hoechst.tif]
