# Supplementary figures and images for: Complex interplay between RAS GTPases and RASSF effectors regulates subcellular localization of YAP (part 3 of 4)
Source: EMBO Rep. 2024 Jul 15;25(8):22. doi: 10.1038/s44319-024-00203-9 (PMC11316025; doi:10.1038/s44319-024-00203-9)

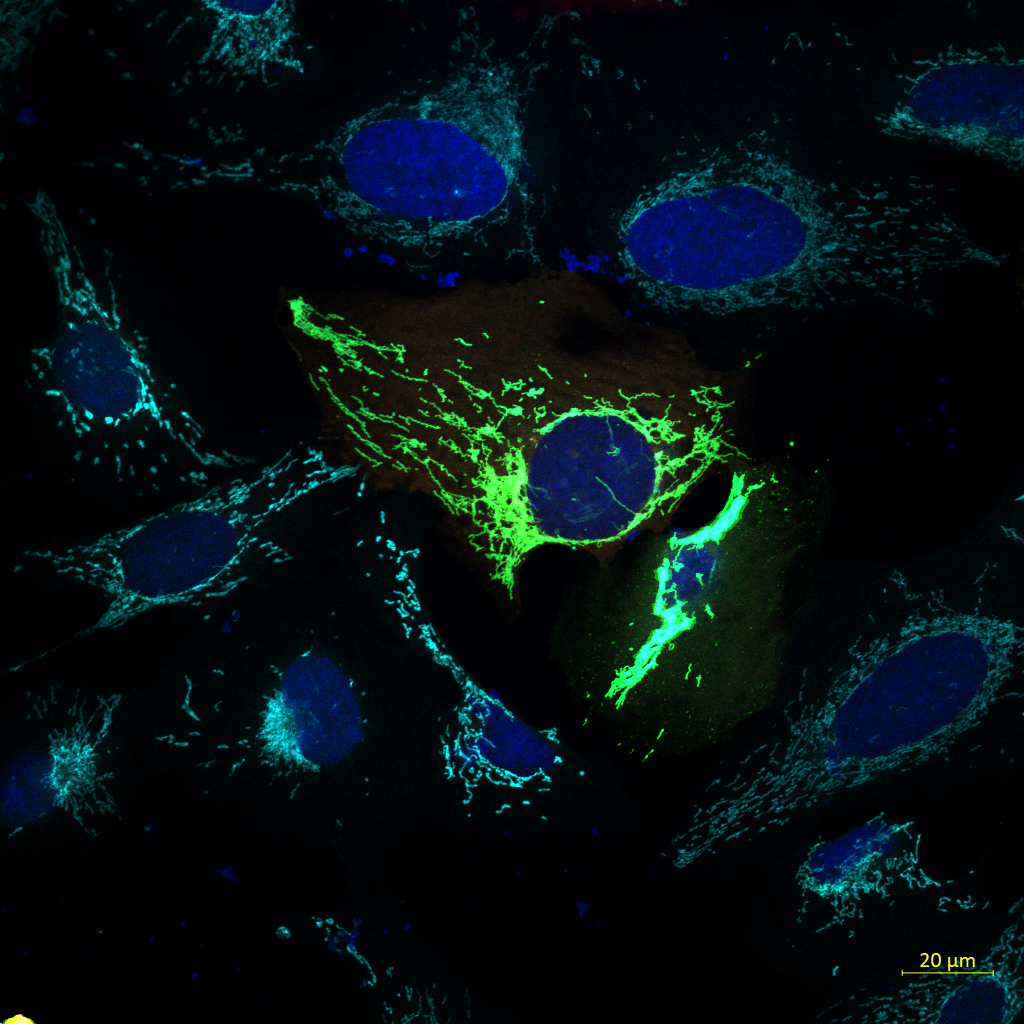

Supplement: Supplementary file 9 — Source data Fig. 7 [file 44319_2024_203_MOESM9_ESM.zip › 7A/VenusMiro1WT+mCh-RASFF3/Merge.tif]

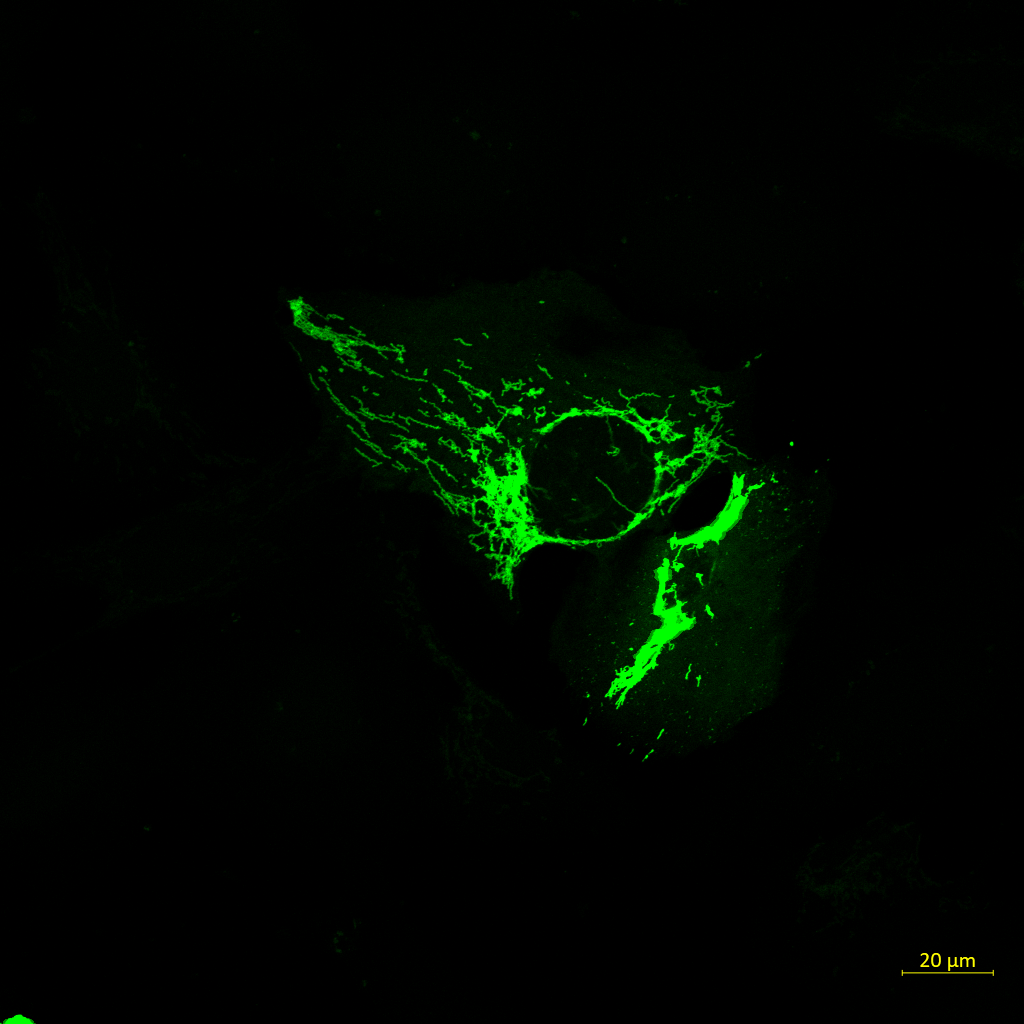

Supplement: Supplementary file 9 — Source data Fig. 7 [file 44319_2024_203_MOESM9_ESM.zip › 7A/VenusMiro1WT+mCh-RASFF3/MIRO1.tif]

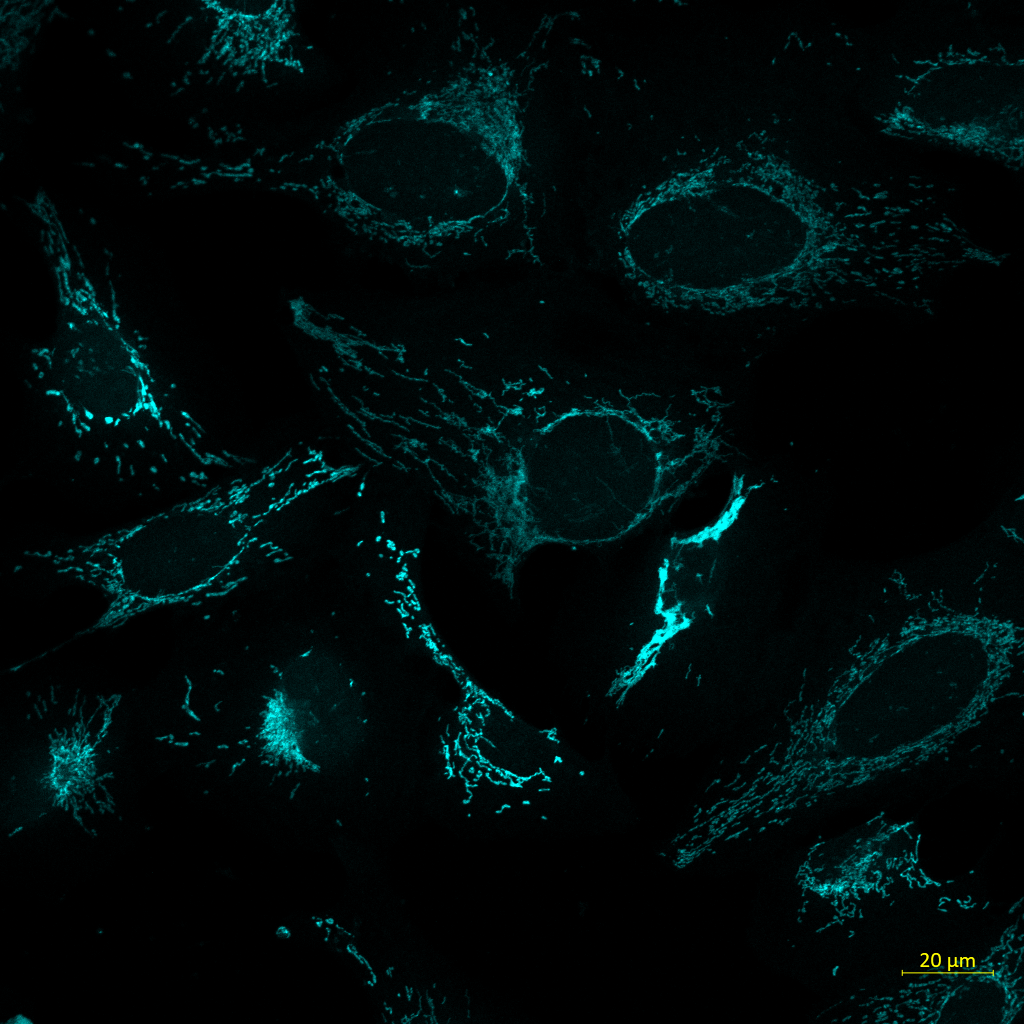

Supplement: Supplementary file 9 — Source data Fig. 7 [file 44319_2024_203_MOESM9_ESM.zip › 7A/VenusMiro1WT+mCh-RASFF3/MitoTracker.tif]

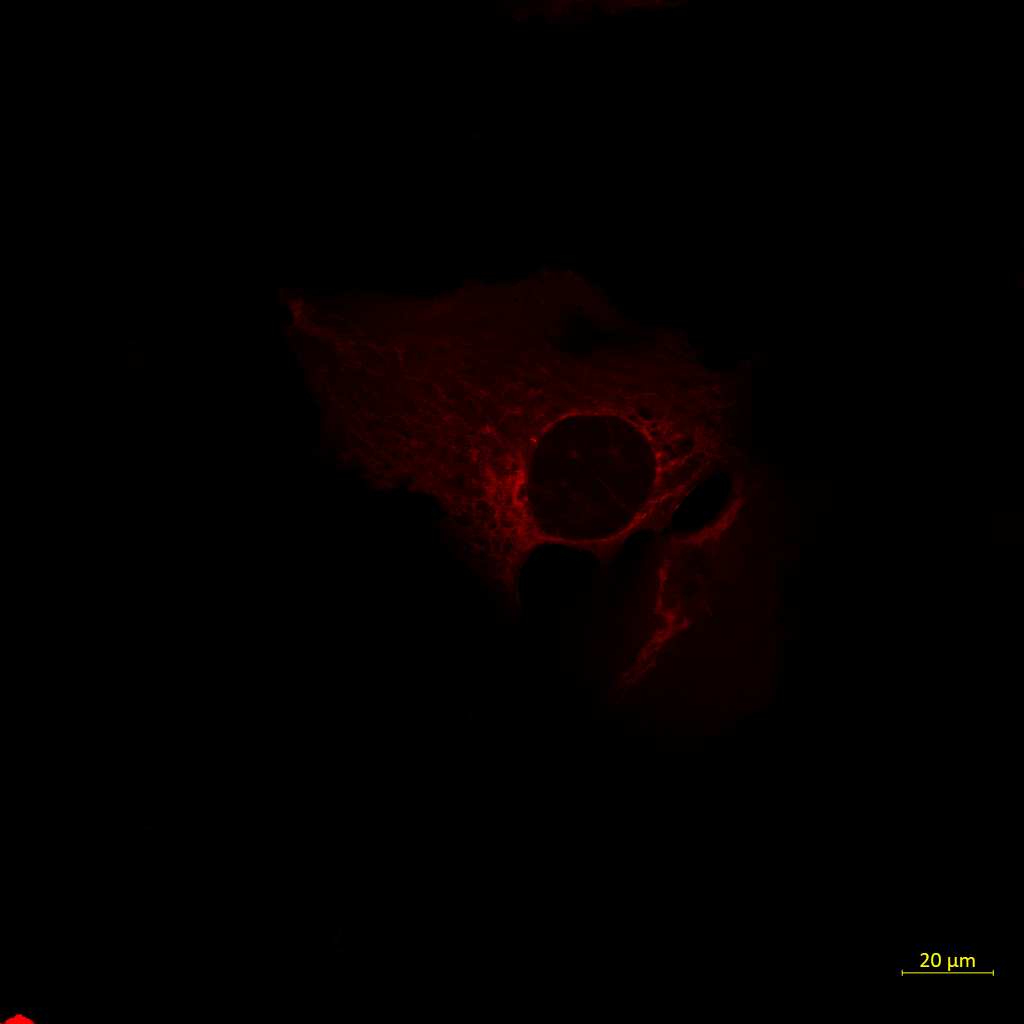

Supplement: Supplementary file 9 — Source data Fig. 7 [file 44319_2024_203_MOESM9_ESM.zip › 7A/VenusMiro1WT+mCh-RASFF3/RASSF3.tif]

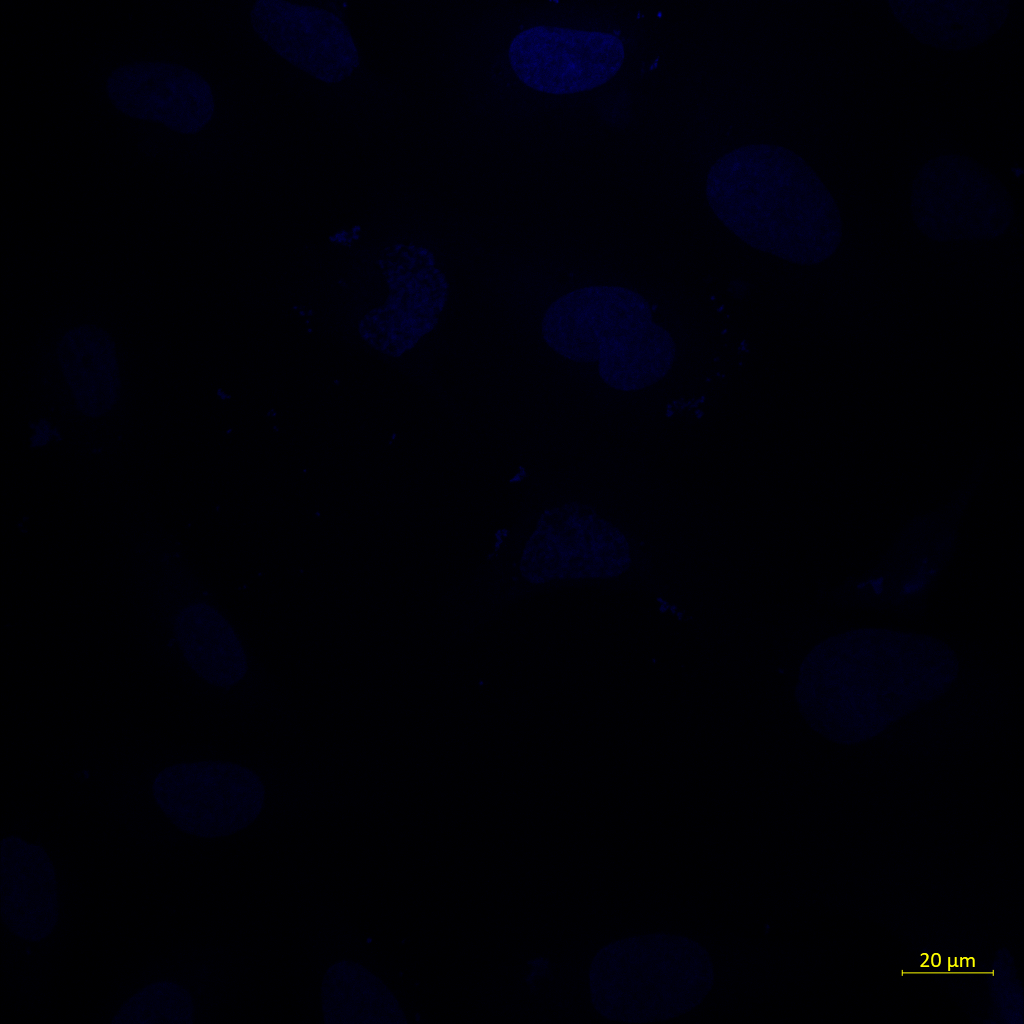

Supplement: Supplementary file 9 — Source data Fig. 7 [file 44319_2024_203_MOESM9_ESM.zip › 7A/VenusMiro2+mCh-RASSF3/Hoechst.tif]

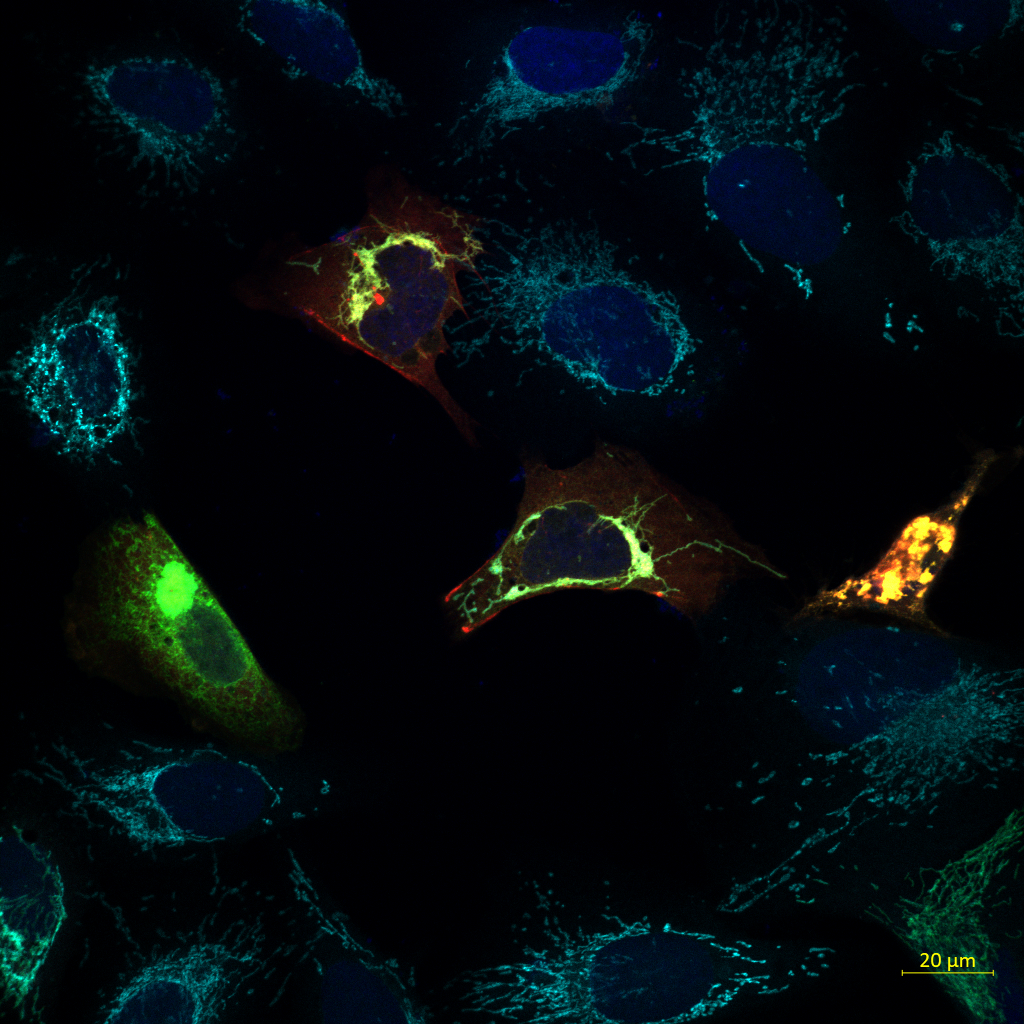

Supplement: Supplementary file 9 — Source data Fig. 7 [file 44319_2024_203_MOESM9_ESM.zip › 7A/VenusMiro2+mCh-RASSF3/Merge.tif]

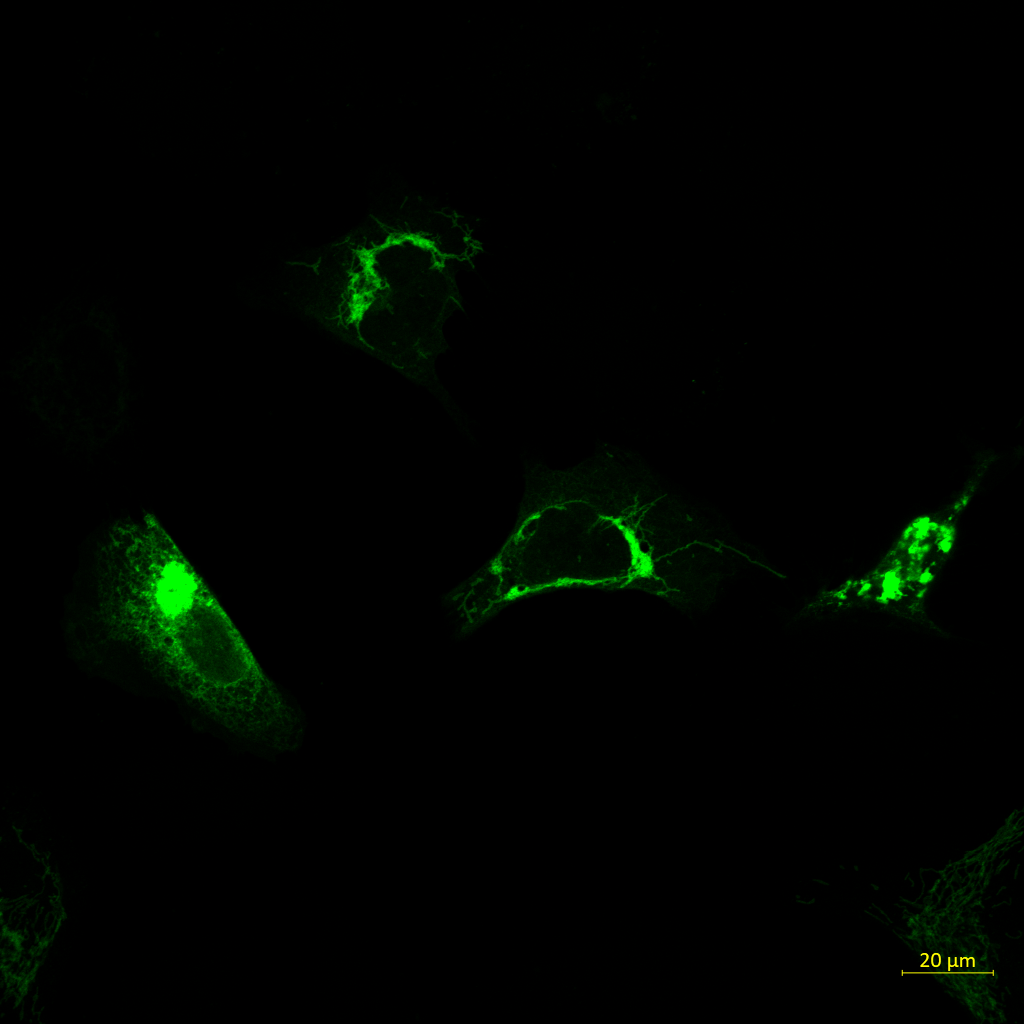

Supplement: Supplementary file 9 — Source data Fig. 7 [file 44319_2024_203_MOESM9_ESM.zip › 7A/VenusMiro2+mCh-RASSF3/MIRO2.tif]

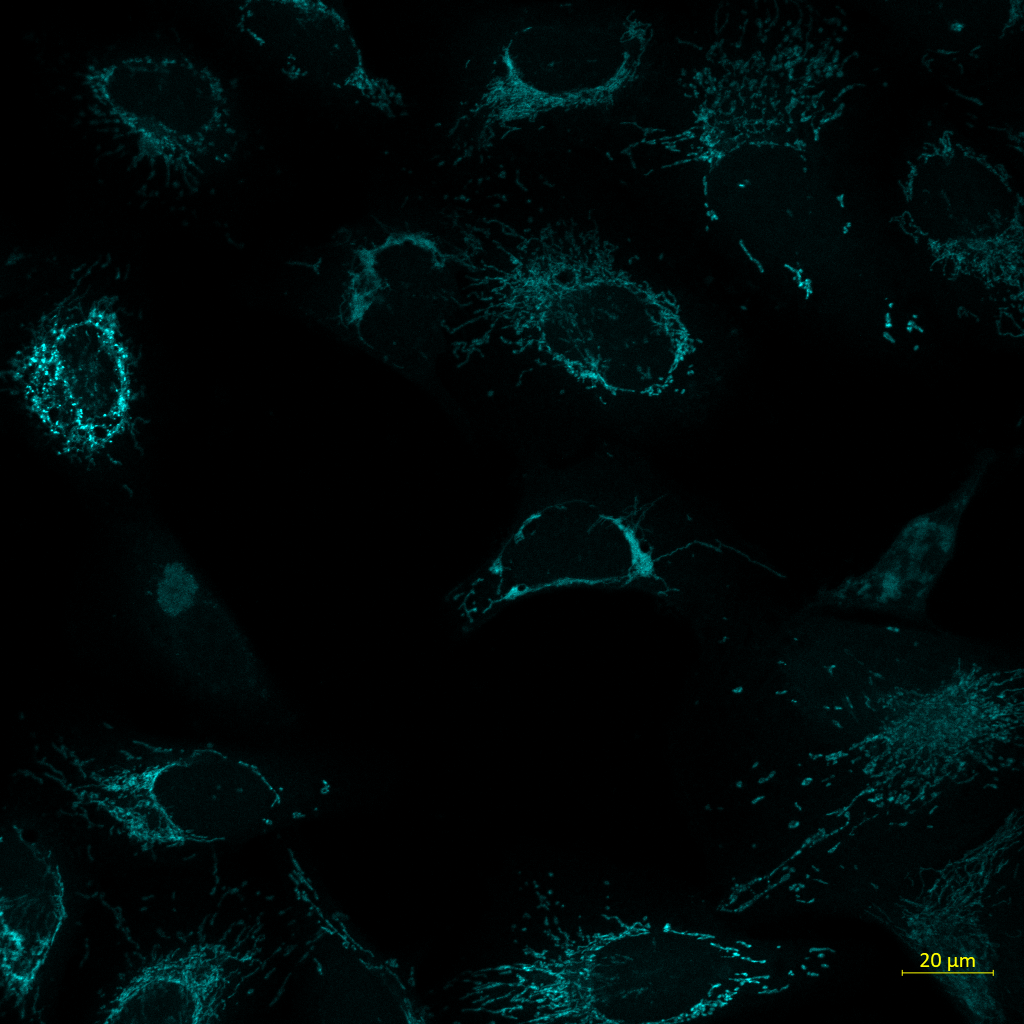

Supplement: Supplementary file 9 — Source data Fig. 7 [file 44319_2024_203_MOESM9_ESM.zip › 7A/VenusMiro2+mCh-RASSF3/MitoTracker.tif]

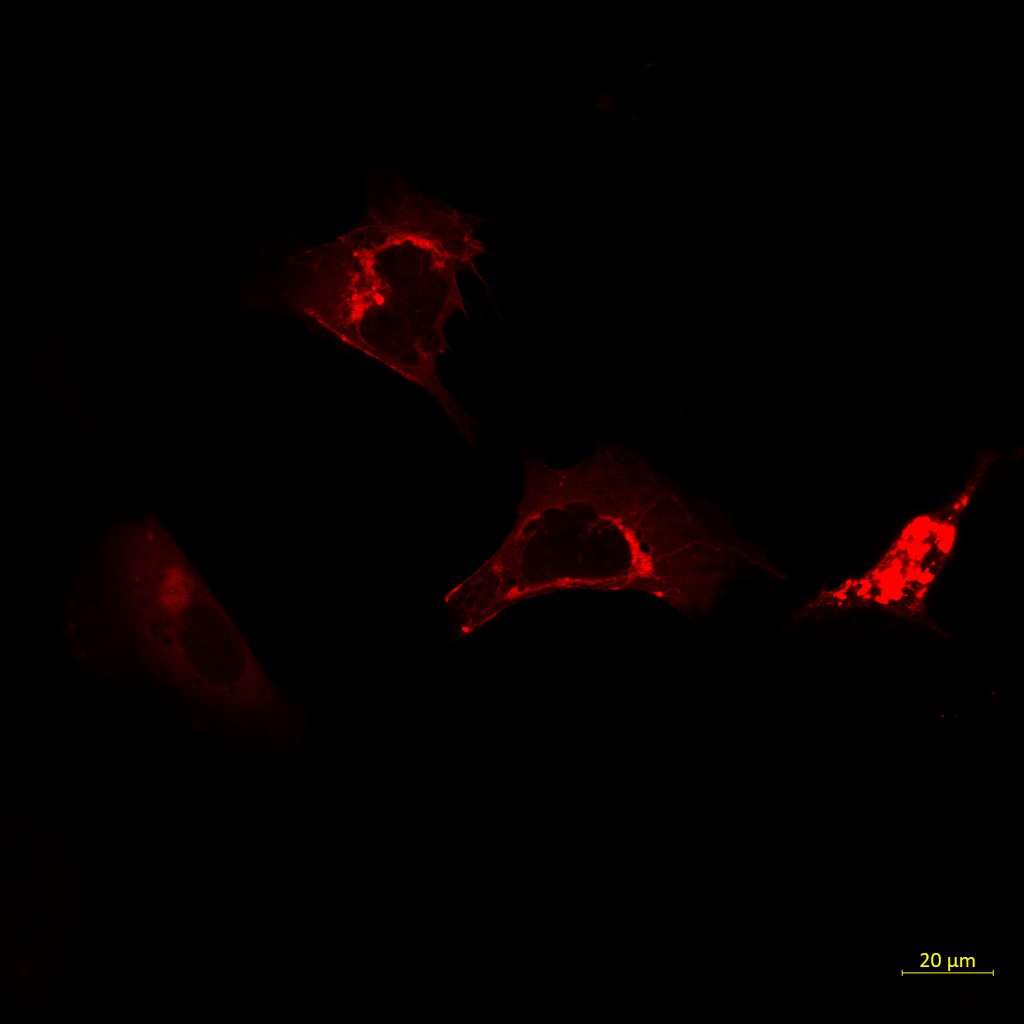

Supplement: Supplementary file 9 — Source data Fig. 7 [file 44319_2024_203_MOESM9_ESM.zip › 7A/VenusMiro2+mCh-RASSF3/RASSF3.tif]

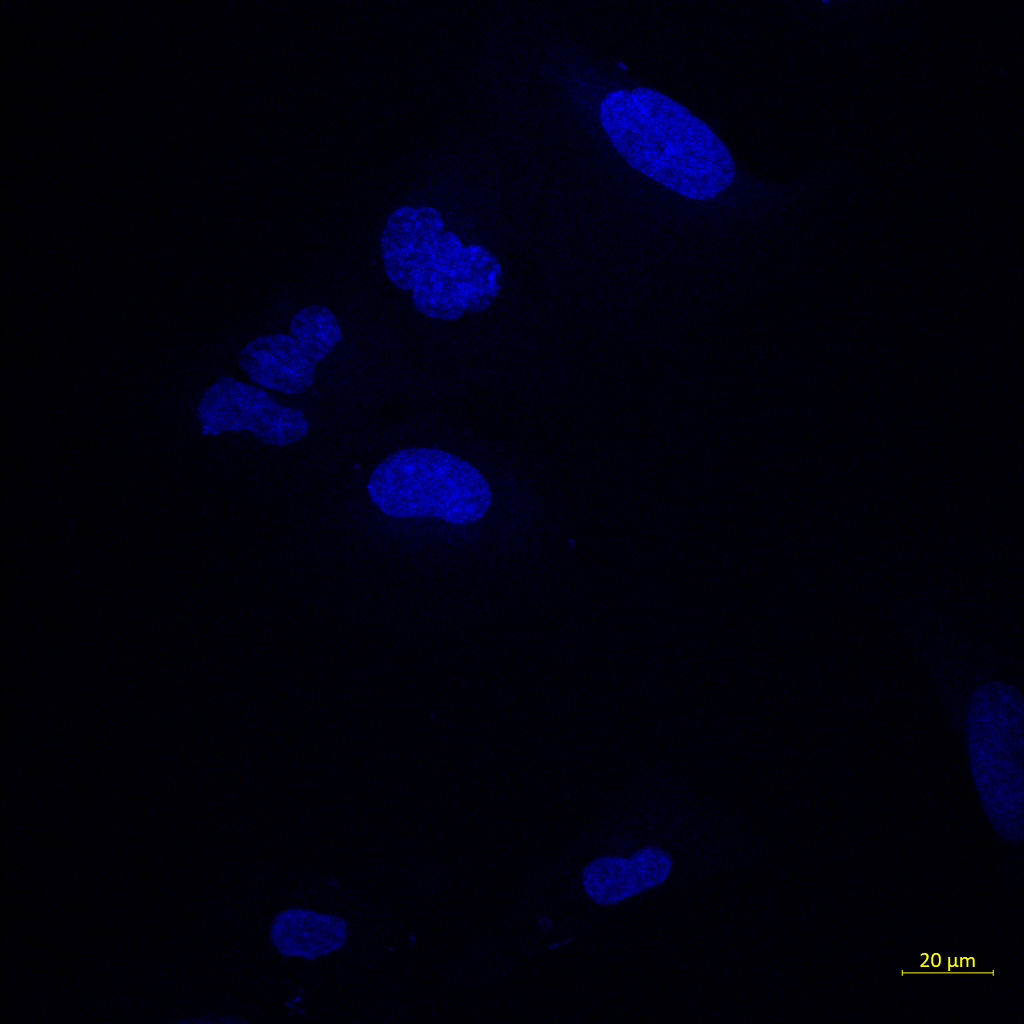

Supplement: Supplementary file 9 — Source data Fig. 7 [file 44319_2024_203_MOESM9_ESM.zip › 7E/EGFP-MIRO1v4+mCherry/Hoechst.tif]

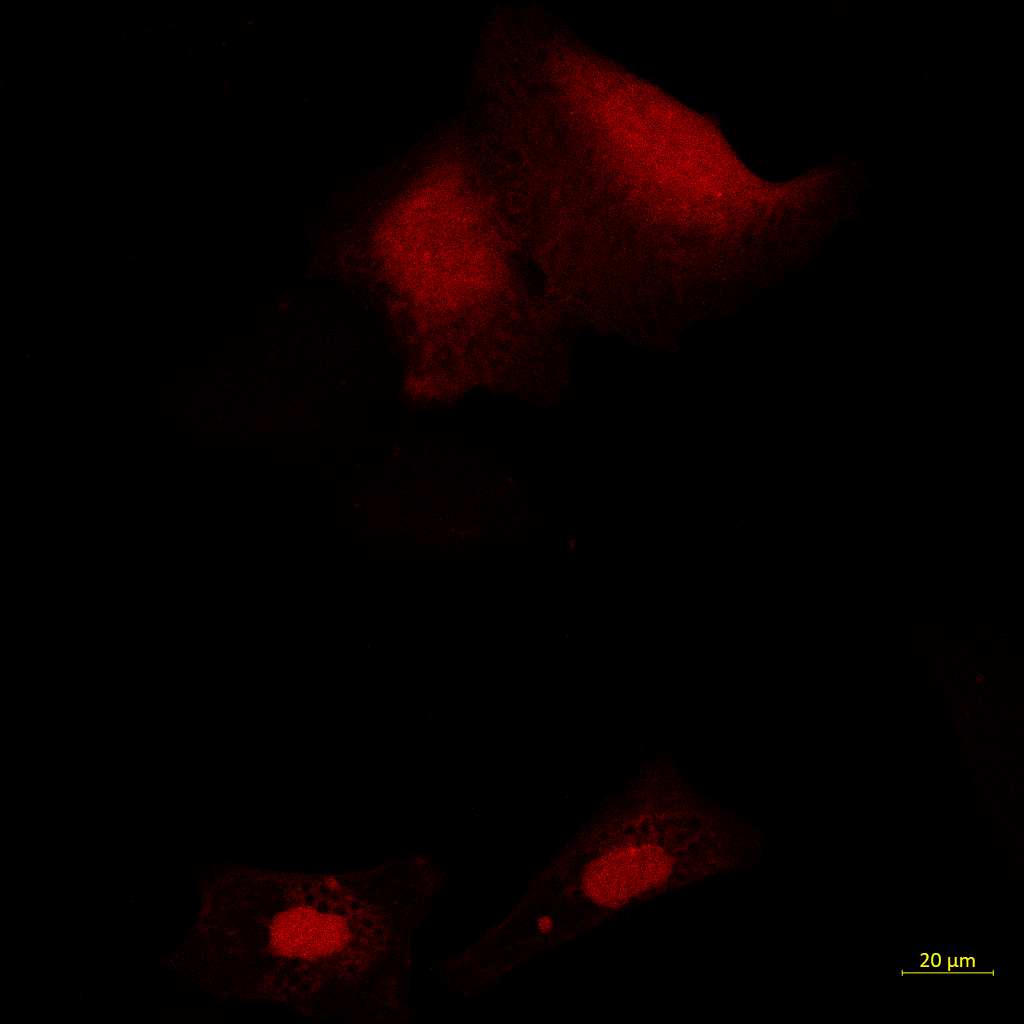

Supplement: Supplementary file 9 — Source data Fig. 7 [file 44319_2024_203_MOESM9_ESM.zip › 7E/EGFP-MIRO1v4+mCherry/mCherry.tif]

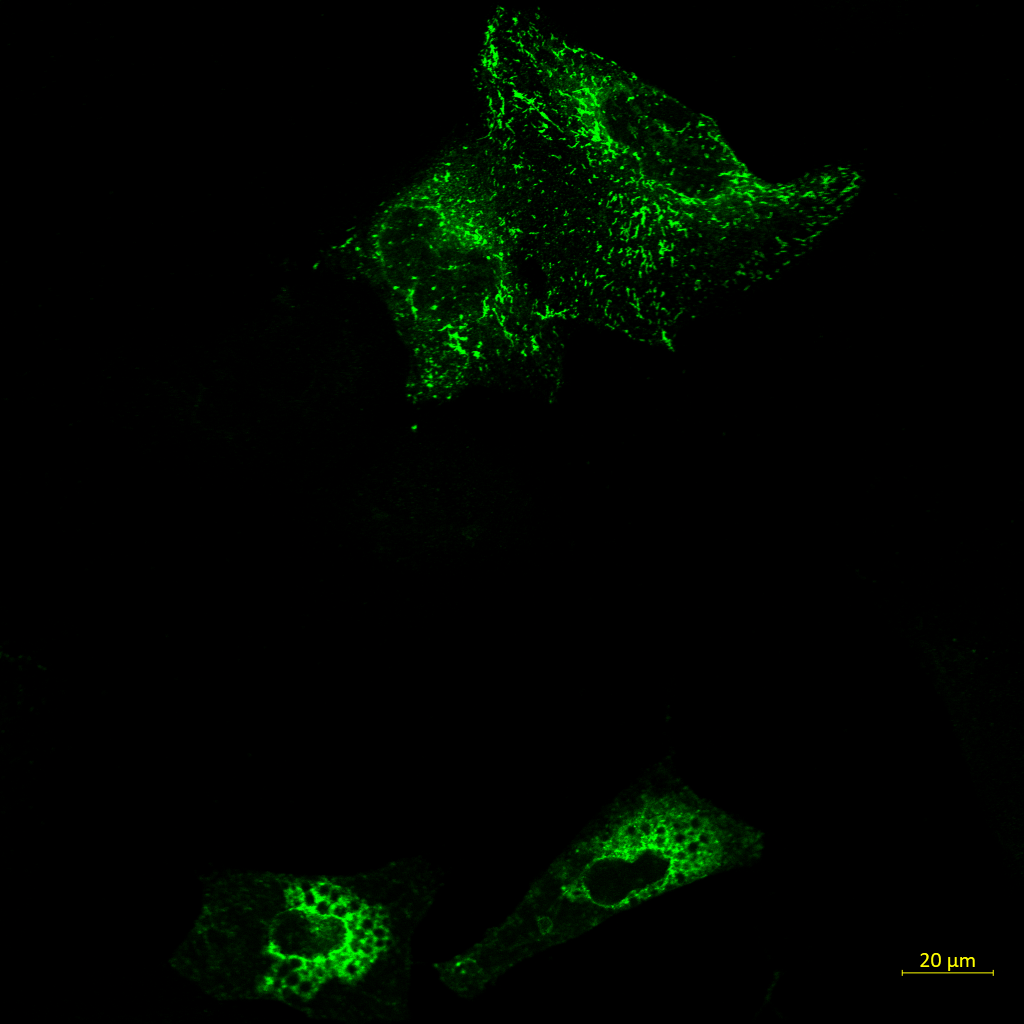

Supplement: Supplementary file 9 — Source data Fig. 7 [file 44319_2024_203_MOESM9_ESM.zip › 7E/EGFP-MIRO1v4+mCherry/MIRO1v4.tif]

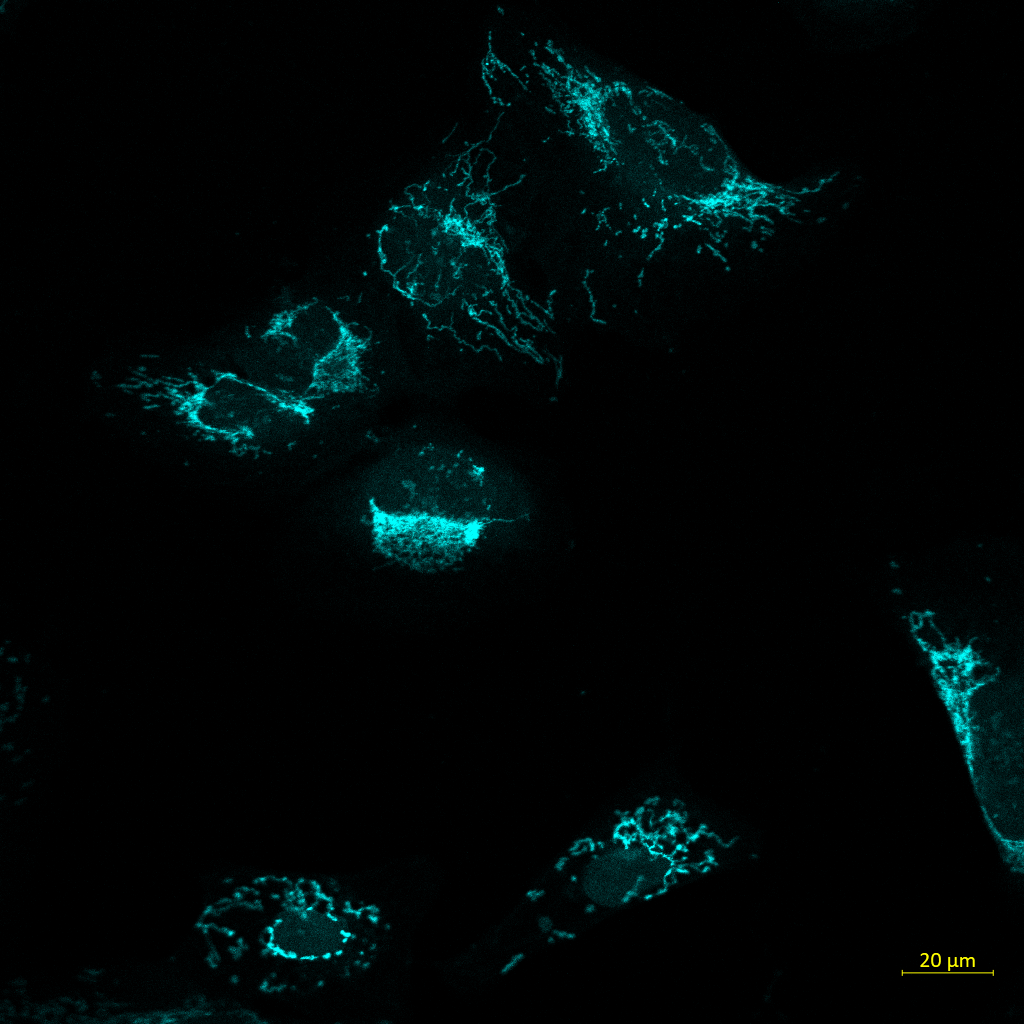

Supplement: Supplementary file 9 — Source data Fig. 7 [file 44319_2024_203_MOESM9_ESM.zip › 7E/EGFP-MIRO1v4+mCherry/MitoTracker.tif]

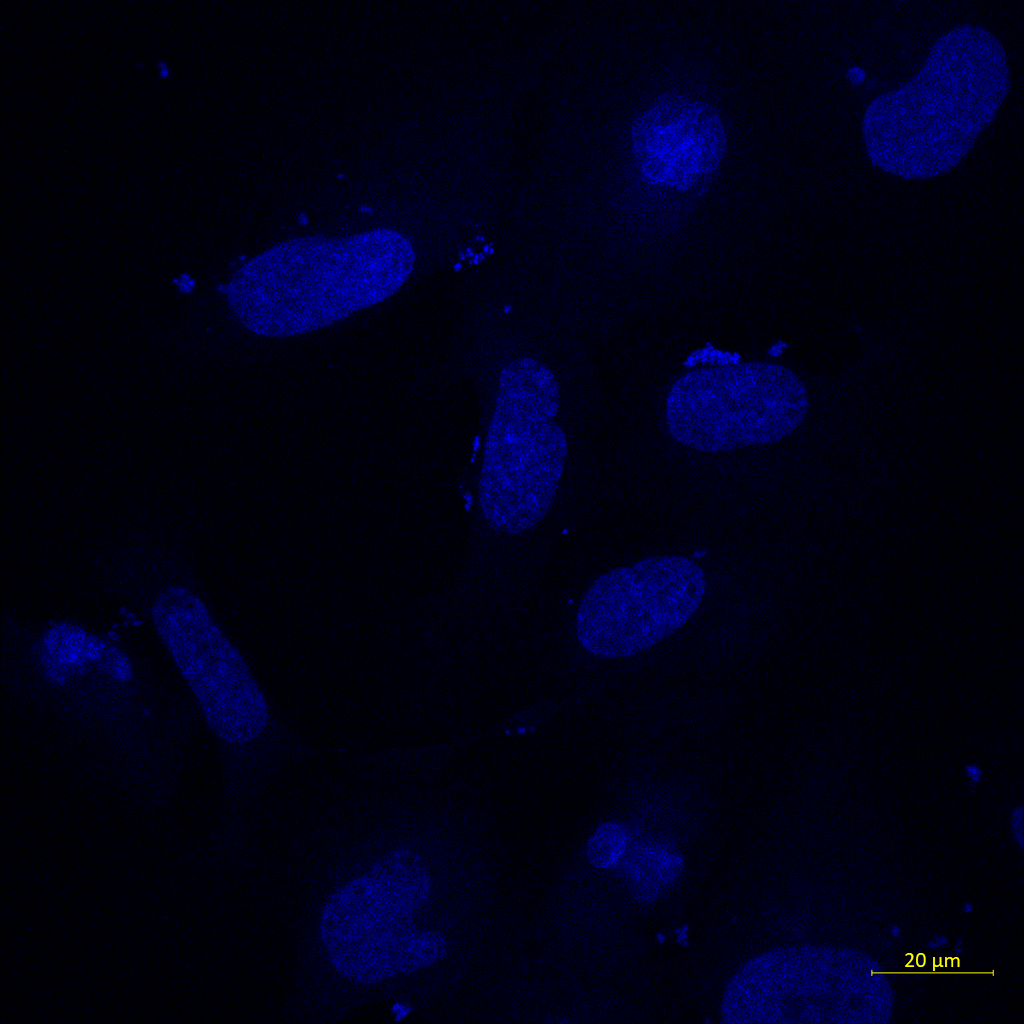

Supplement: Supplementary file 9 — Source data Fig. 7 [file 44319_2024_203_MOESM9_ESM.zip › 7E/EGFP-MIRO1v4+mCherry-RASSF3/Hoechst.tif]

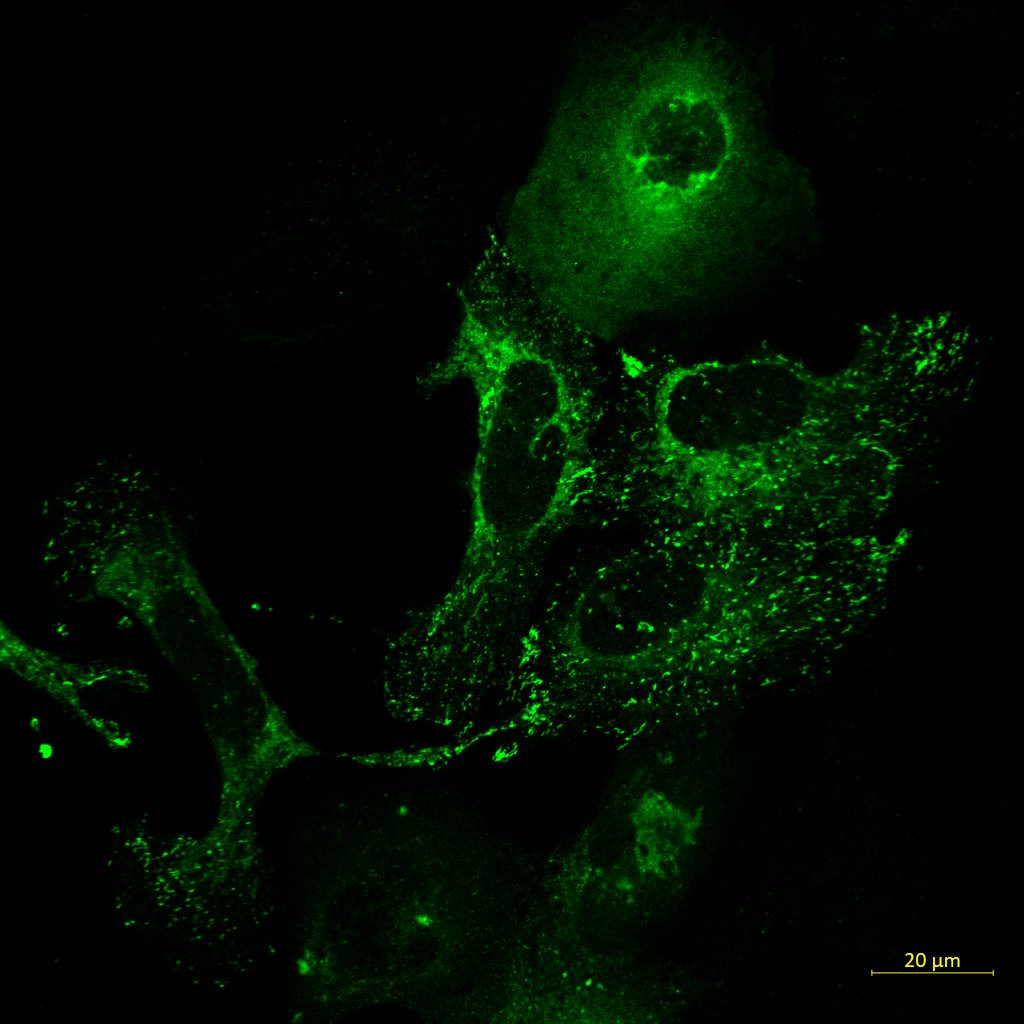

Supplement: Supplementary file 9 — Source data Fig. 7 [file 44319_2024_203_MOESM9_ESM.zip › 7E/EGFP-MIRO1v4+mCherry-RASSF3/MIRO1v4.tif]

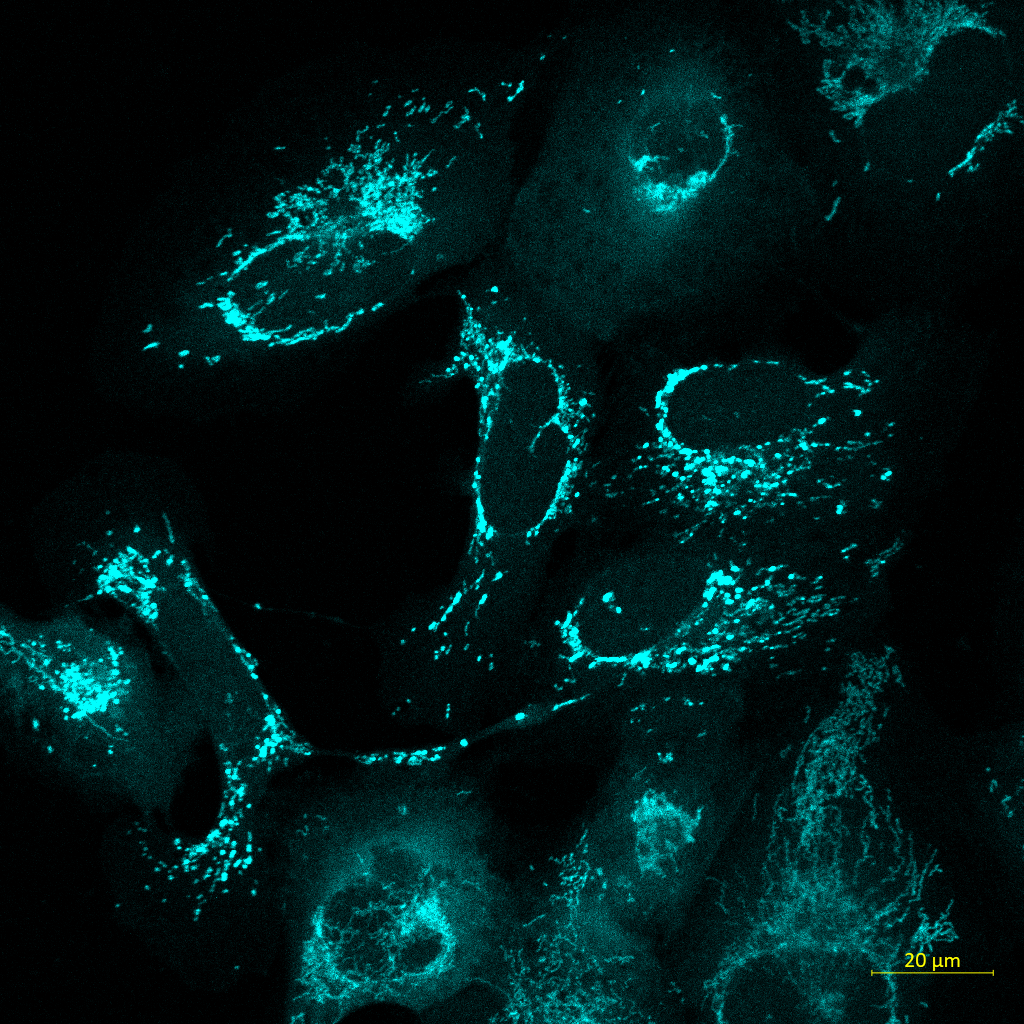

Supplement: Supplementary file 9 — Source data Fig. 7 [file 44319_2024_203_MOESM9_ESM.zip › 7E/EGFP-MIRO1v4+mCherry-RASSF3/MitoTracker.tif]

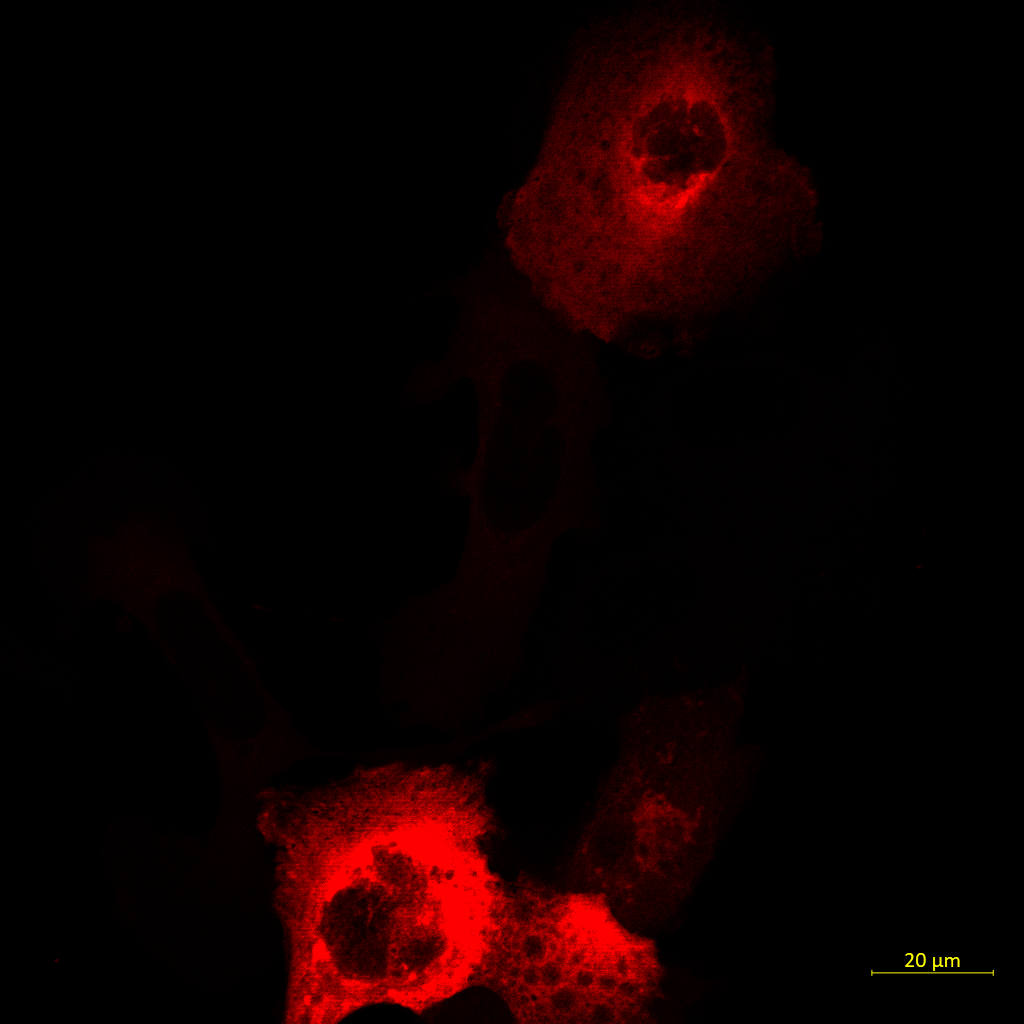

Supplement: Supplementary file 9 — Source data Fig. 7 [file 44319_2024_203_MOESM9_ESM.zip › 7E/EGFP-MIRO1v4+mCherry-RASSF3/RASSF3.tif]

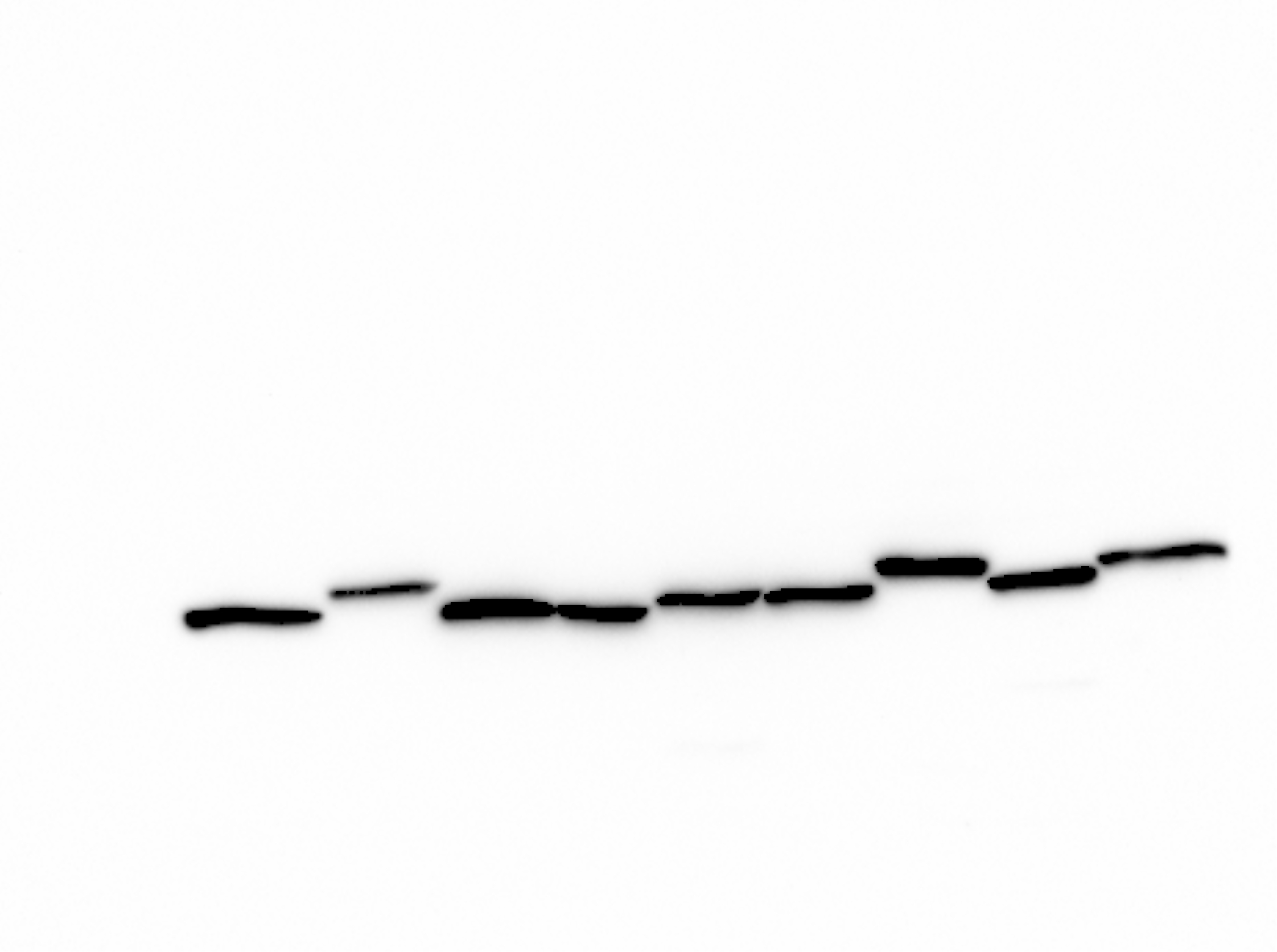

Supplement: Supplementary file 10 — Appendix Figures Source Data [file 44319_2024_203_MOESM10_ESM.zip › Appendix4_RASSF3/Fourthrow/Left/Lysate.jpg]

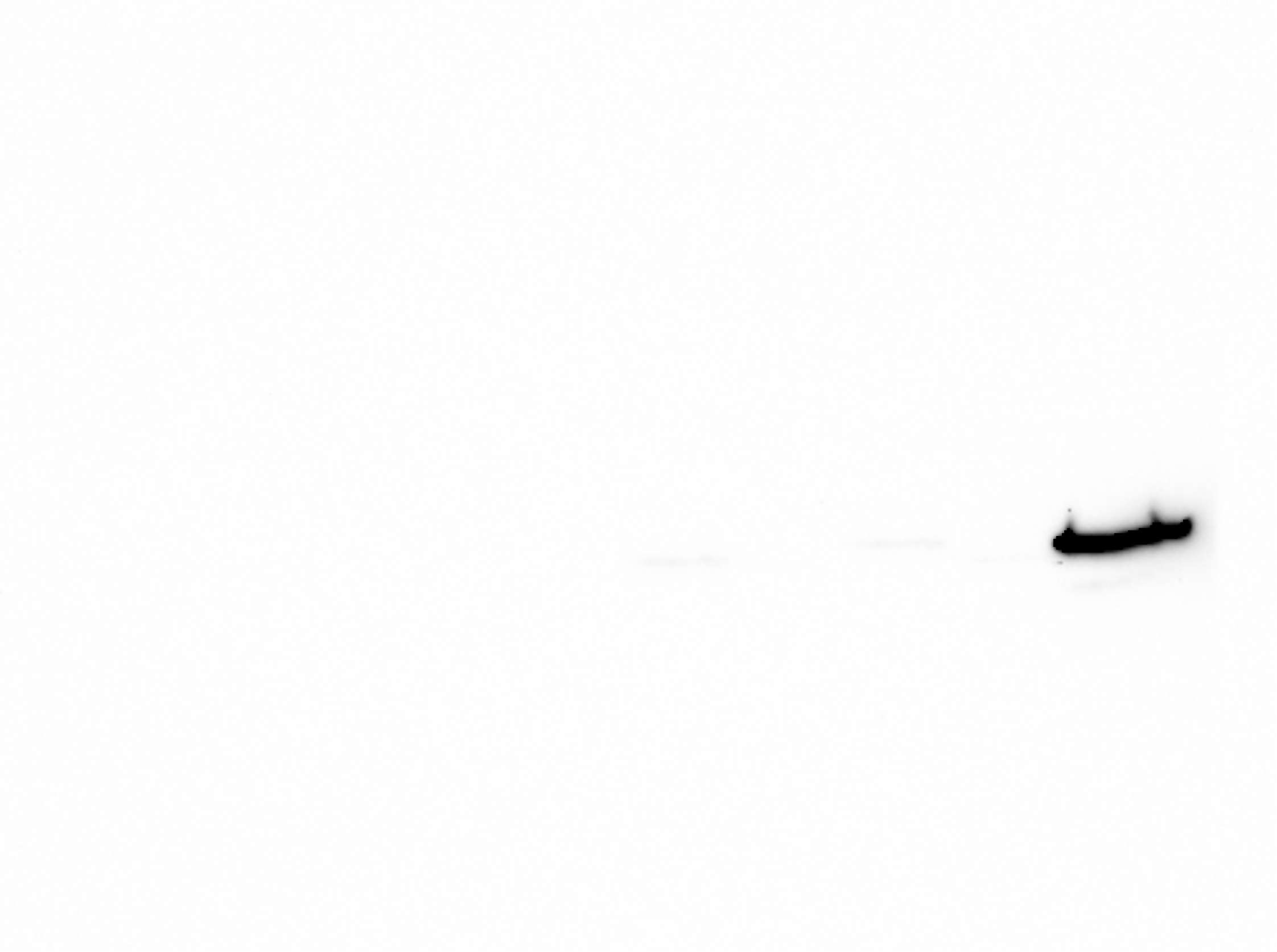

Supplement: Supplementary file 10 — Appendix Figures Source Data [file 44319_2024_203_MOESM10_ESM.zip › Appendix4_RASSF3/Fourthrow/Left/Pulldown.jpg]

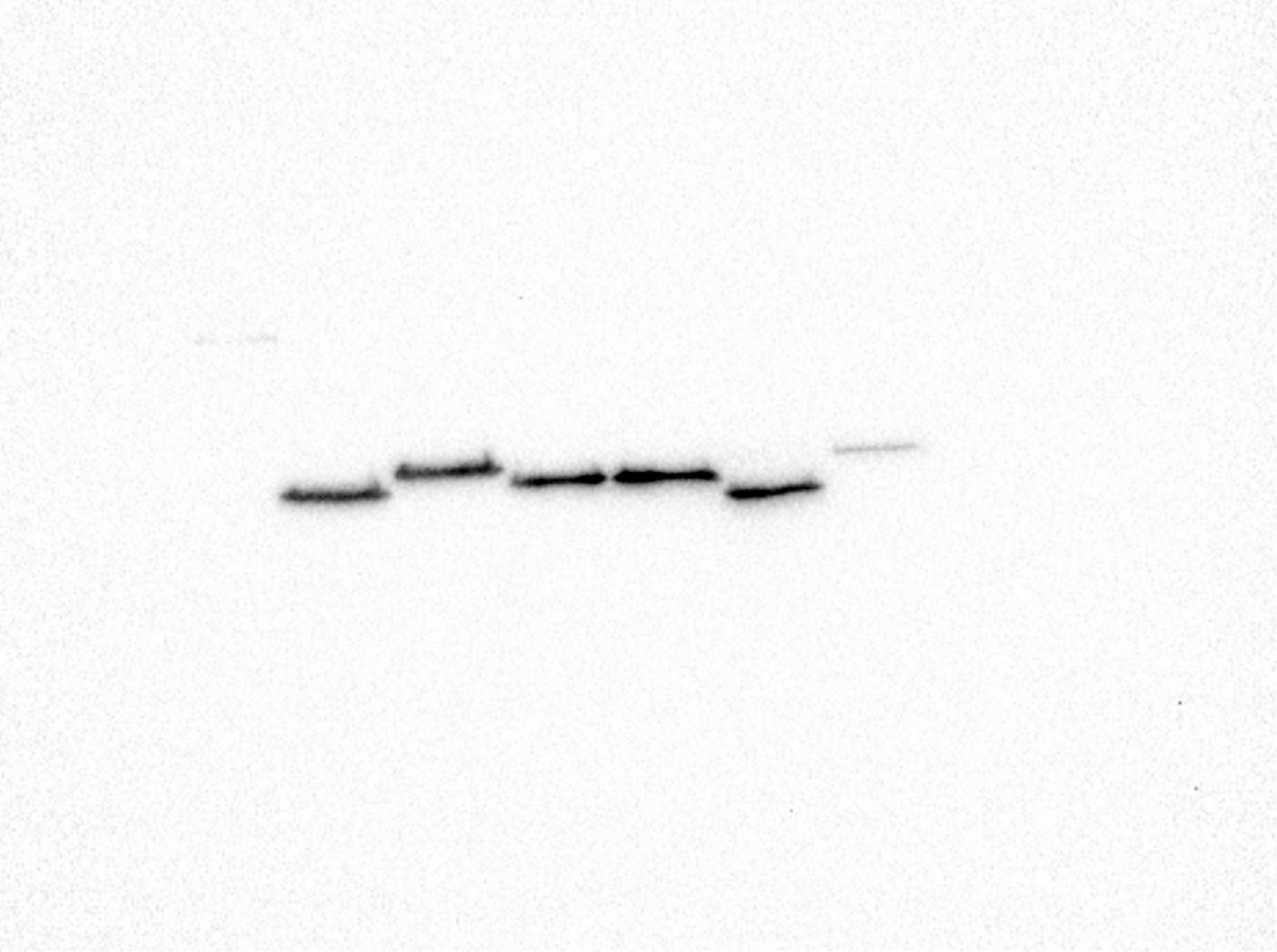

Supplement: Supplementary file 10 — Appendix Figures Source Data [file 44319_2024_203_MOESM10_ESM.zip › Appendix4_RASSF3/Fourthrow/Middle/Lysate.jpg]

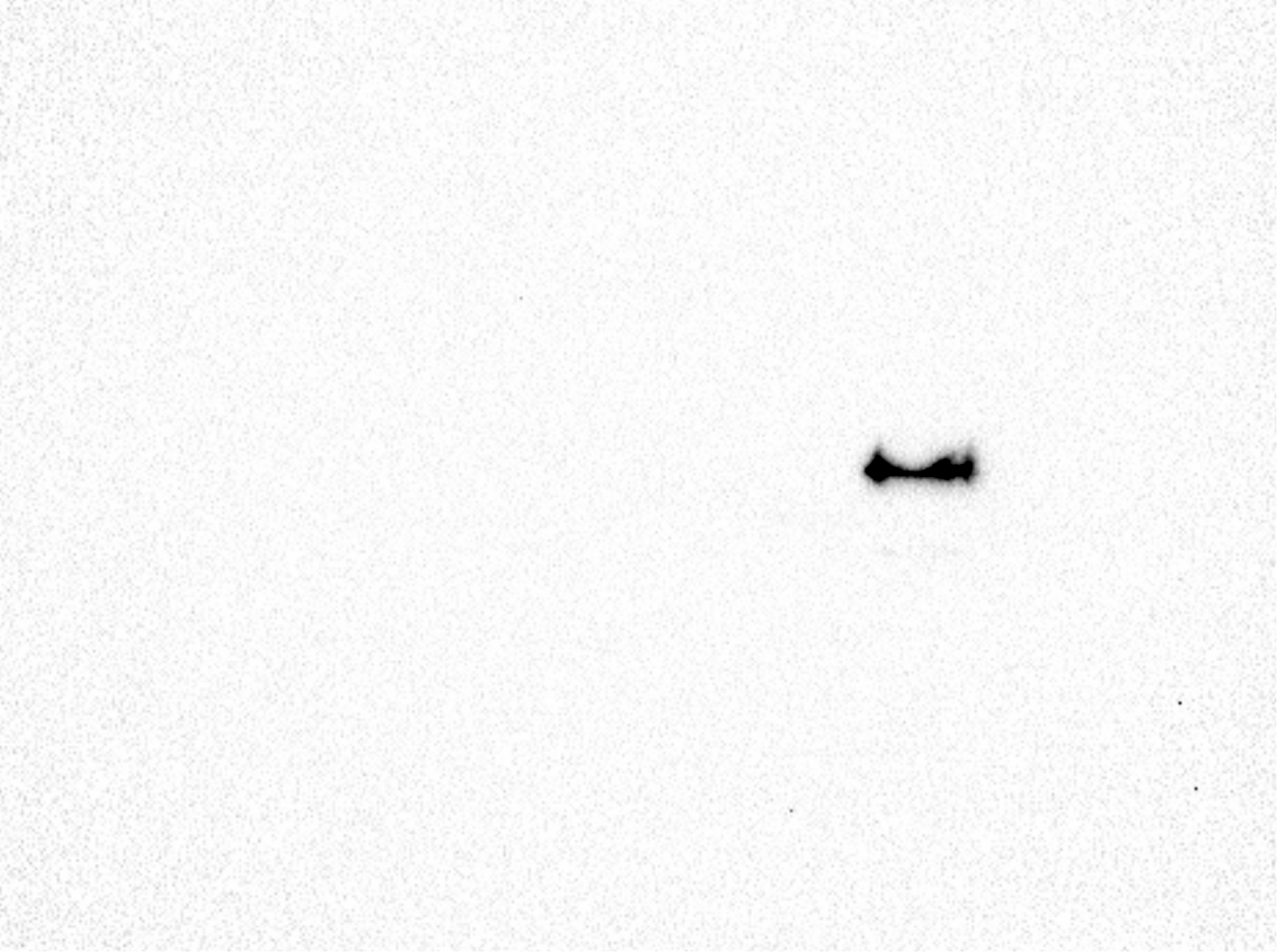

Supplement: Supplementary file 10 — Appendix Figures Source Data [file 44319_2024_203_MOESM10_ESM.zip › Appendix4_RASSF3/Fourthrow/Middle/Pulldown.jpg]

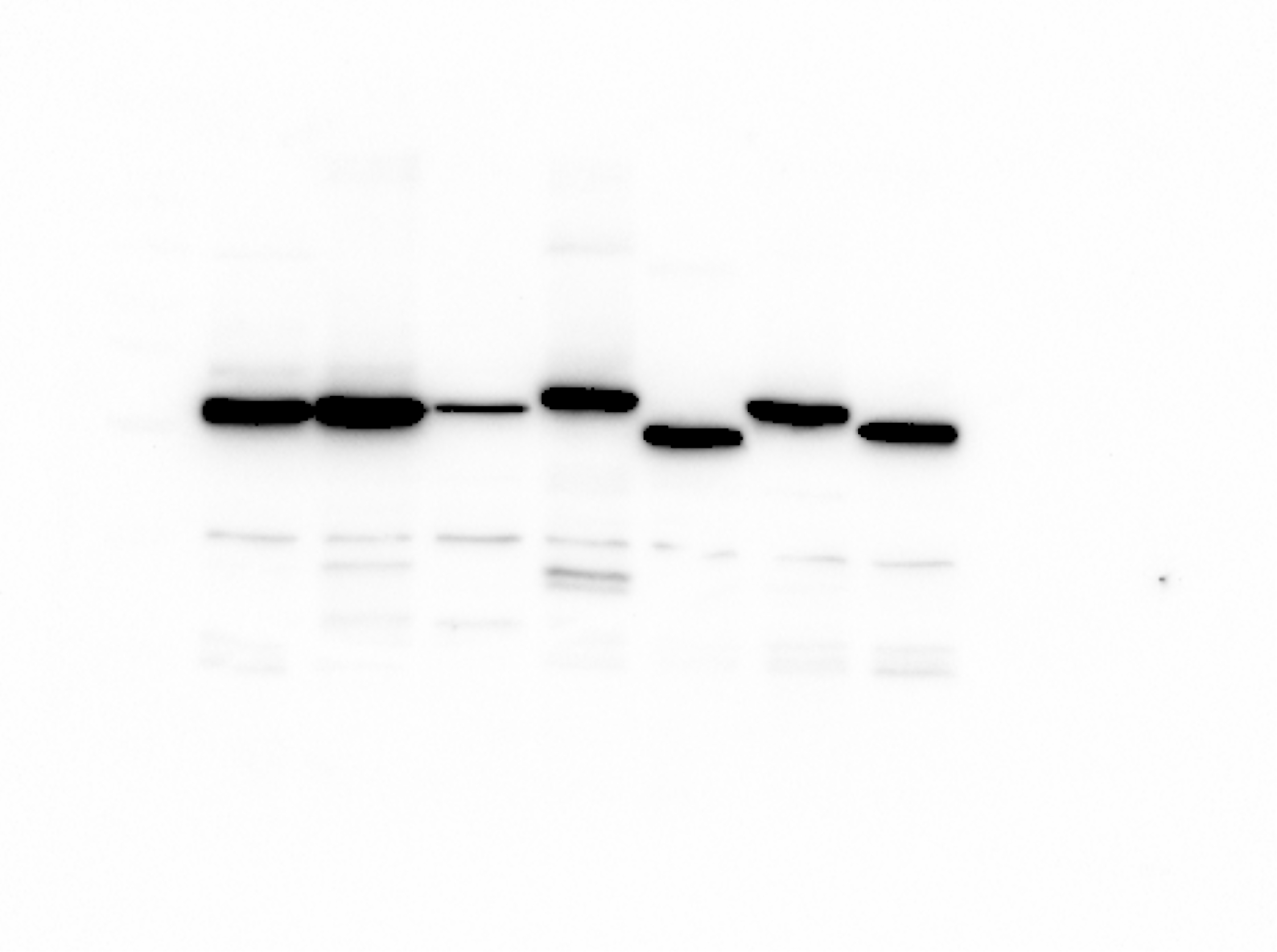

Supplement: Supplementary file 10 — Appendix Figures Source Data [file 44319_2024_203_MOESM10_ESM.zip › Appendix4_RASSF3/Fourthrow/Right/Lysate.jpg]

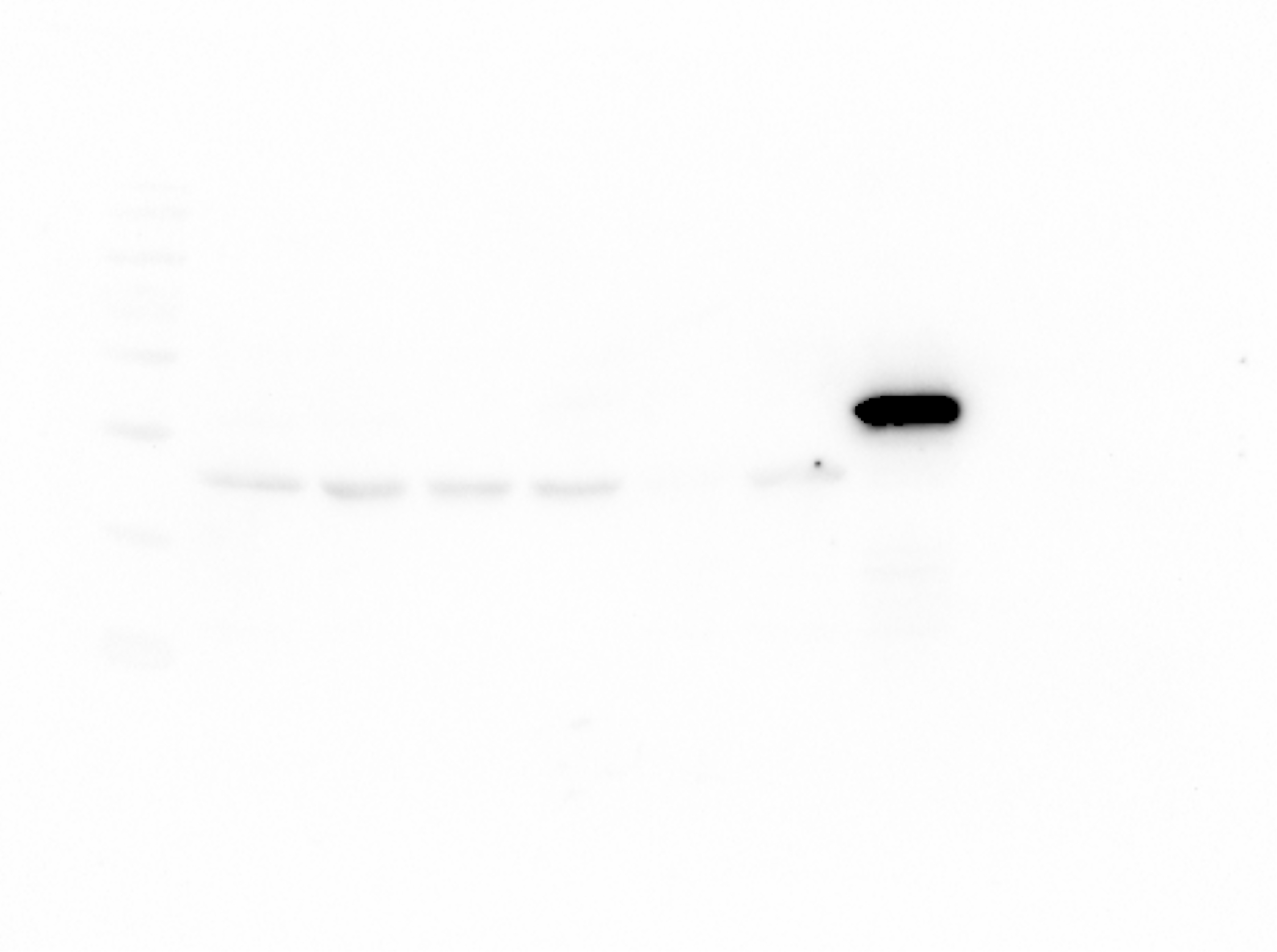

Supplement: Supplementary file 10 — Appendix Figures Source Data [file 44319_2024_203_MOESM10_ESM.zip › Appendix4_RASSF3/Fourthrow/Right/Pulldown.jpg]

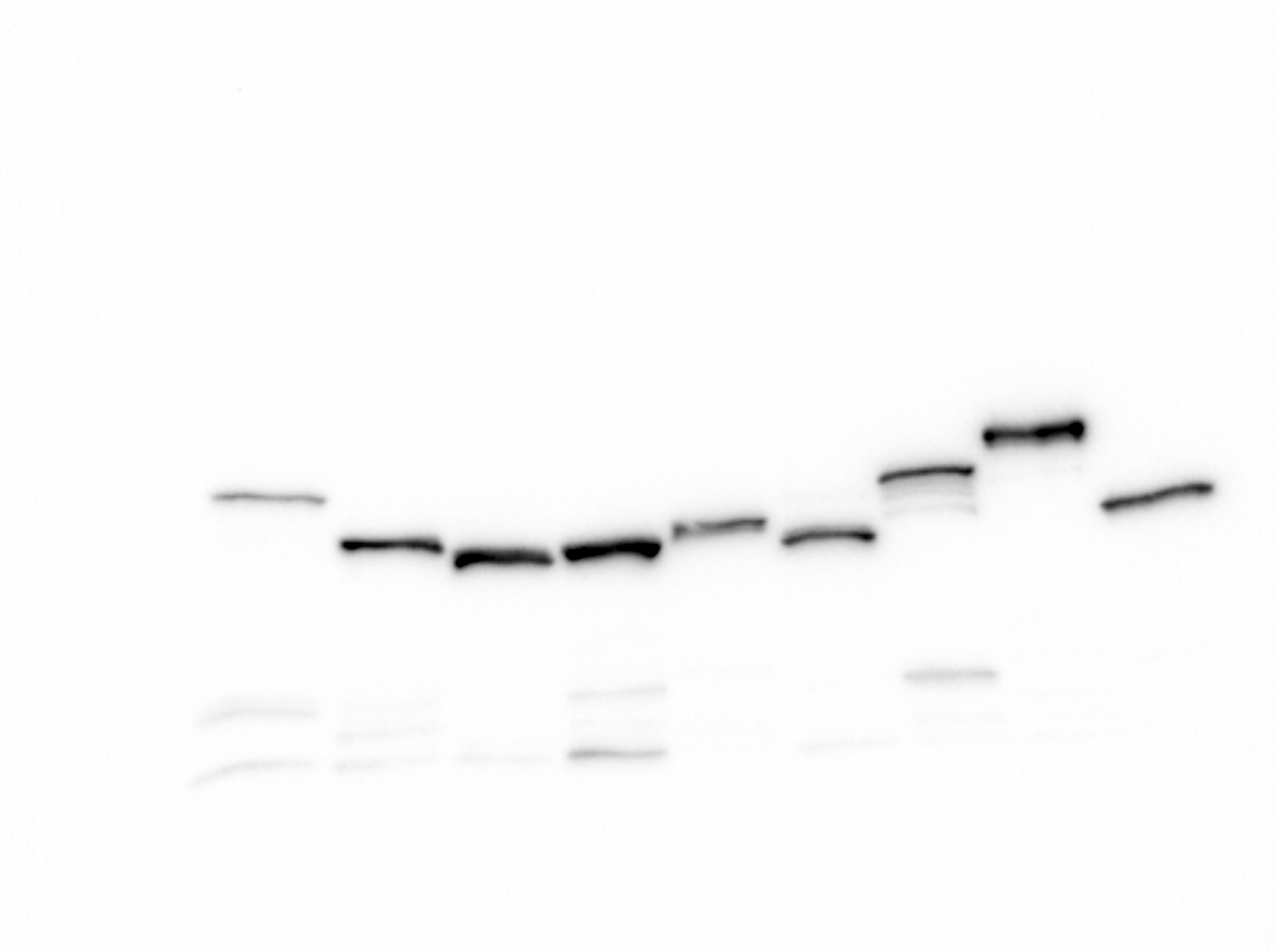

Supplement: Supplementary file 10 — Appendix Figures Source Data [file 44319_2024_203_MOESM10_ESM.zip › Appendix4_RASSF3/Secondrow/Leftmost/Lysate.jpg]

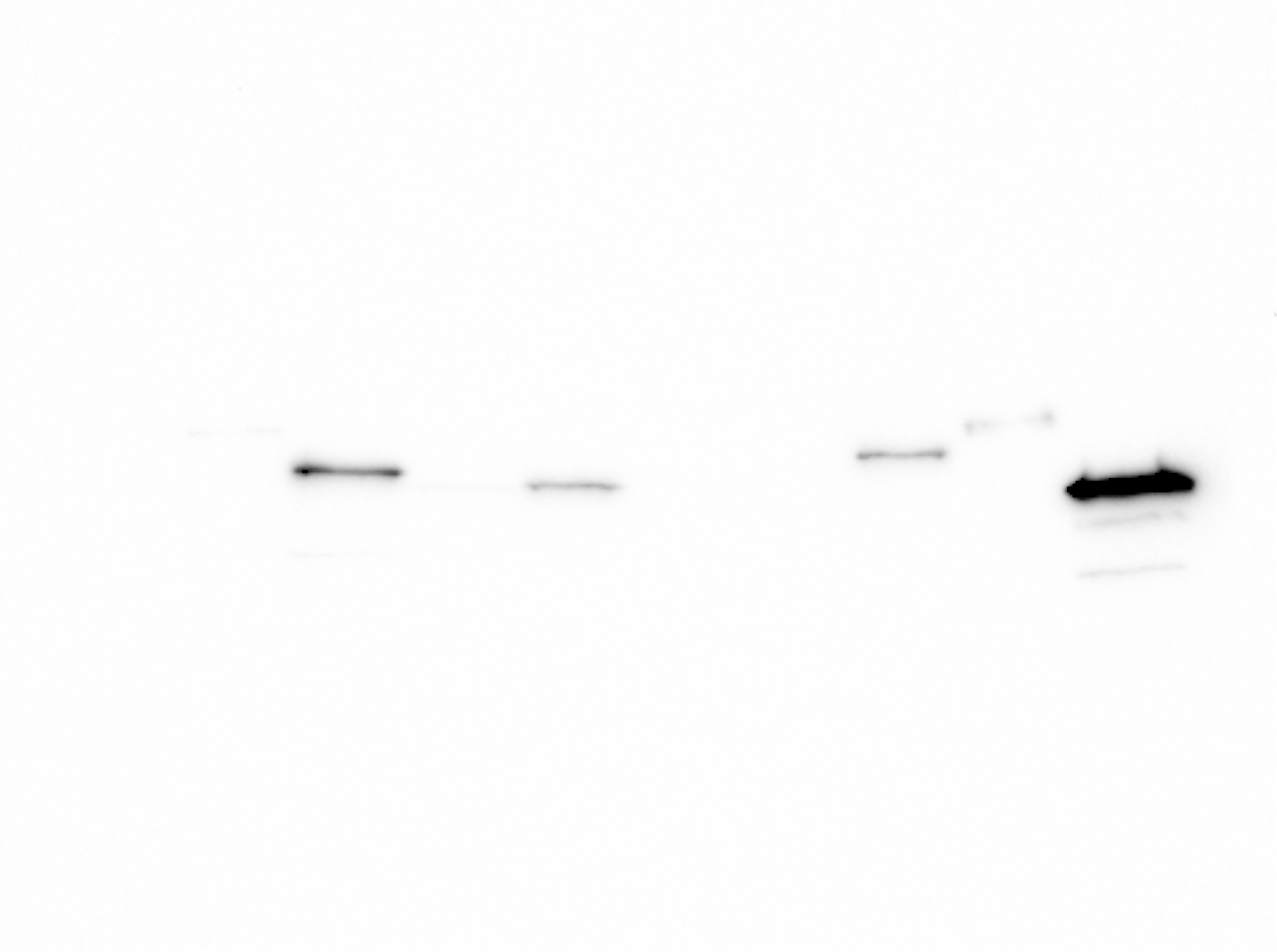

Supplement: Supplementary file 10 — Appendix Figures Source Data [file 44319_2024_203_MOESM10_ESM.zip › Appendix4_RASSF3/Secondrow/Leftmost/Pulldown.jpg]

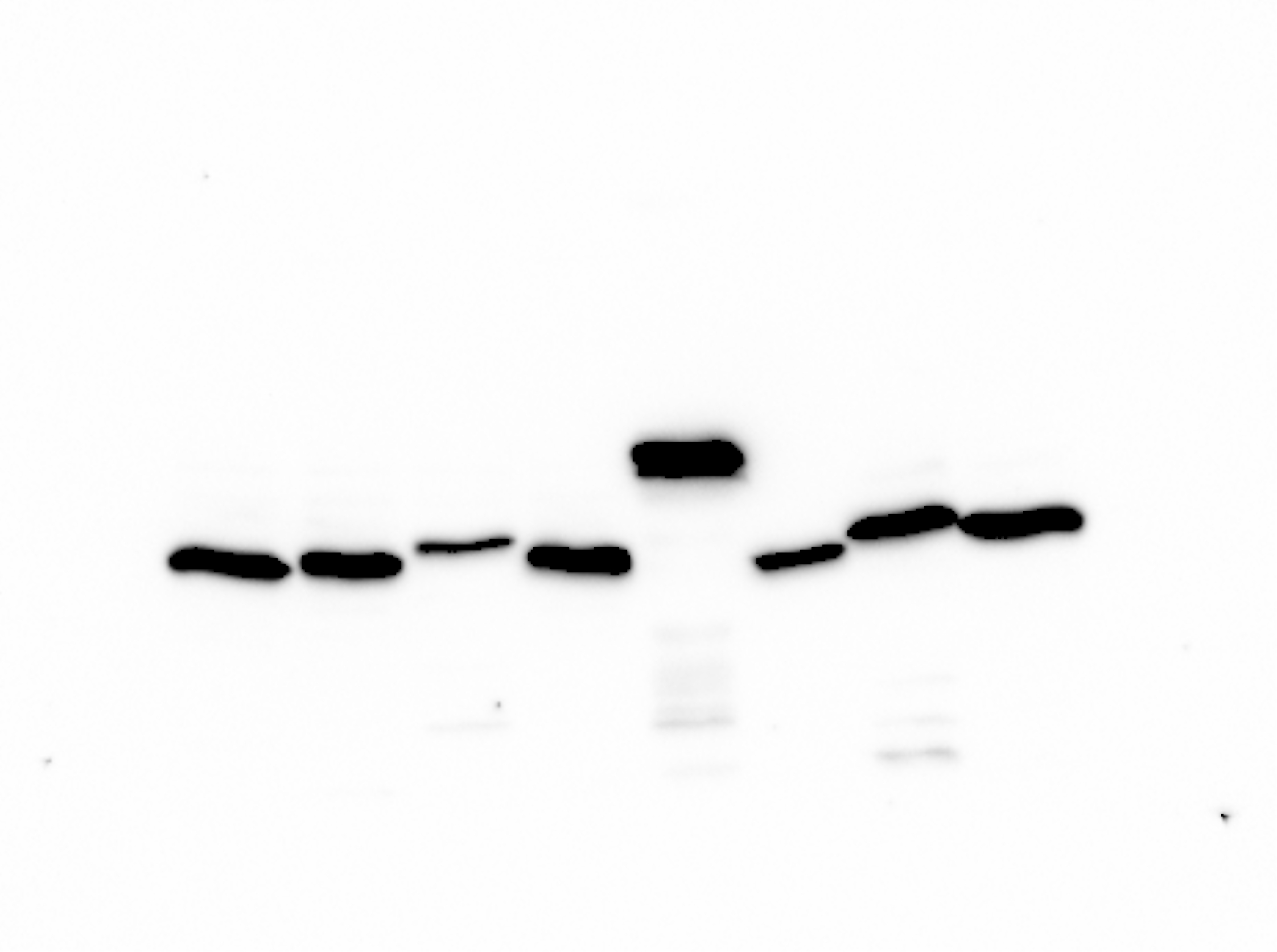

Supplement: Supplementary file 10 — Appendix Figures Source Data [file 44319_2024_203_MOESM10_ESM.zip › Appendix4_RASSF3/Secondrow/Middle/Lysate.jpg]

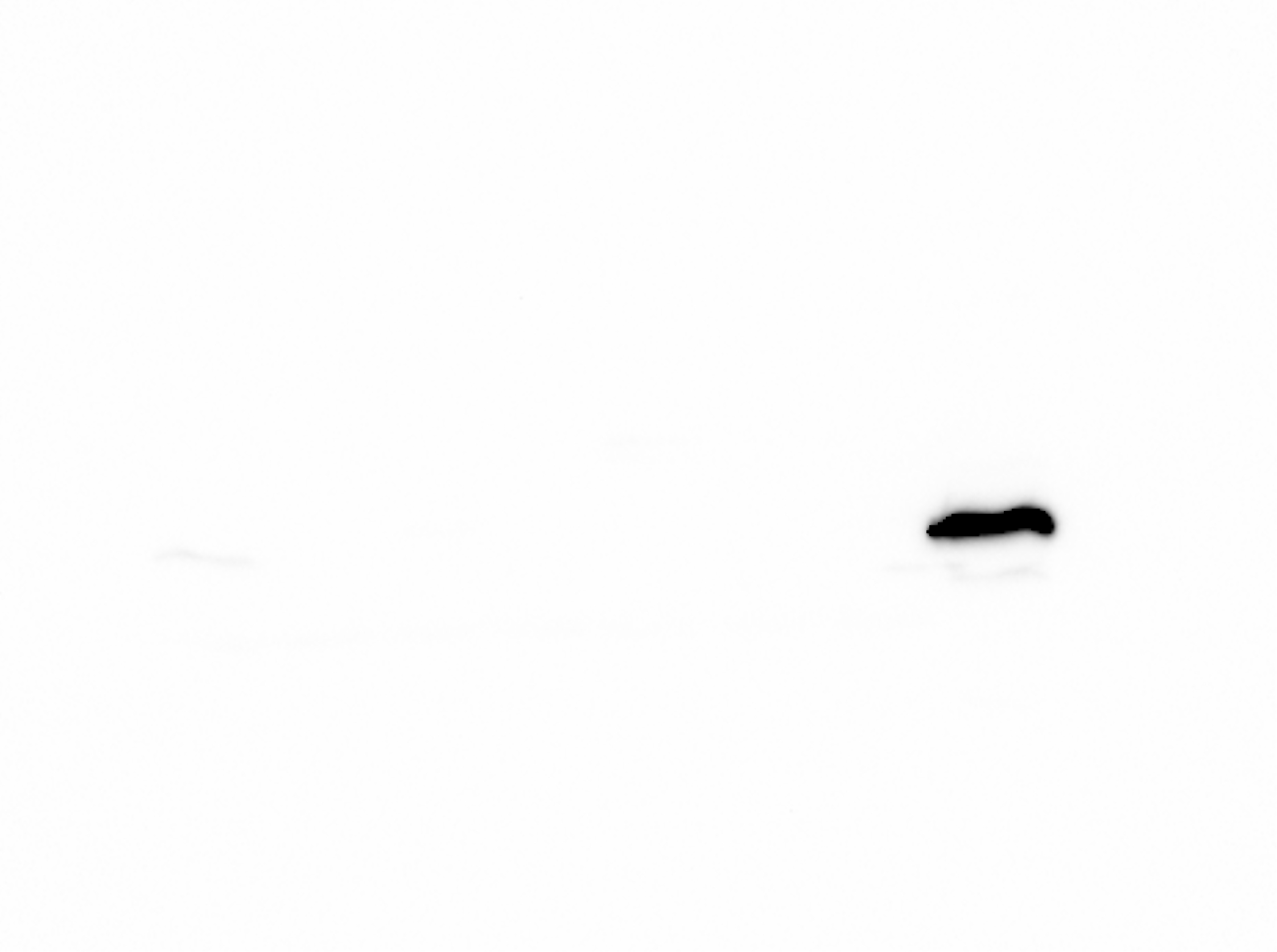

Supplement: Supplementary file 10 — Appendix Figures Source Data [file 44319_2024_203_MOESM10_ESM.zip › Appendix4_RASSF3/Secondrow/Middle/Pulldown.jpg]

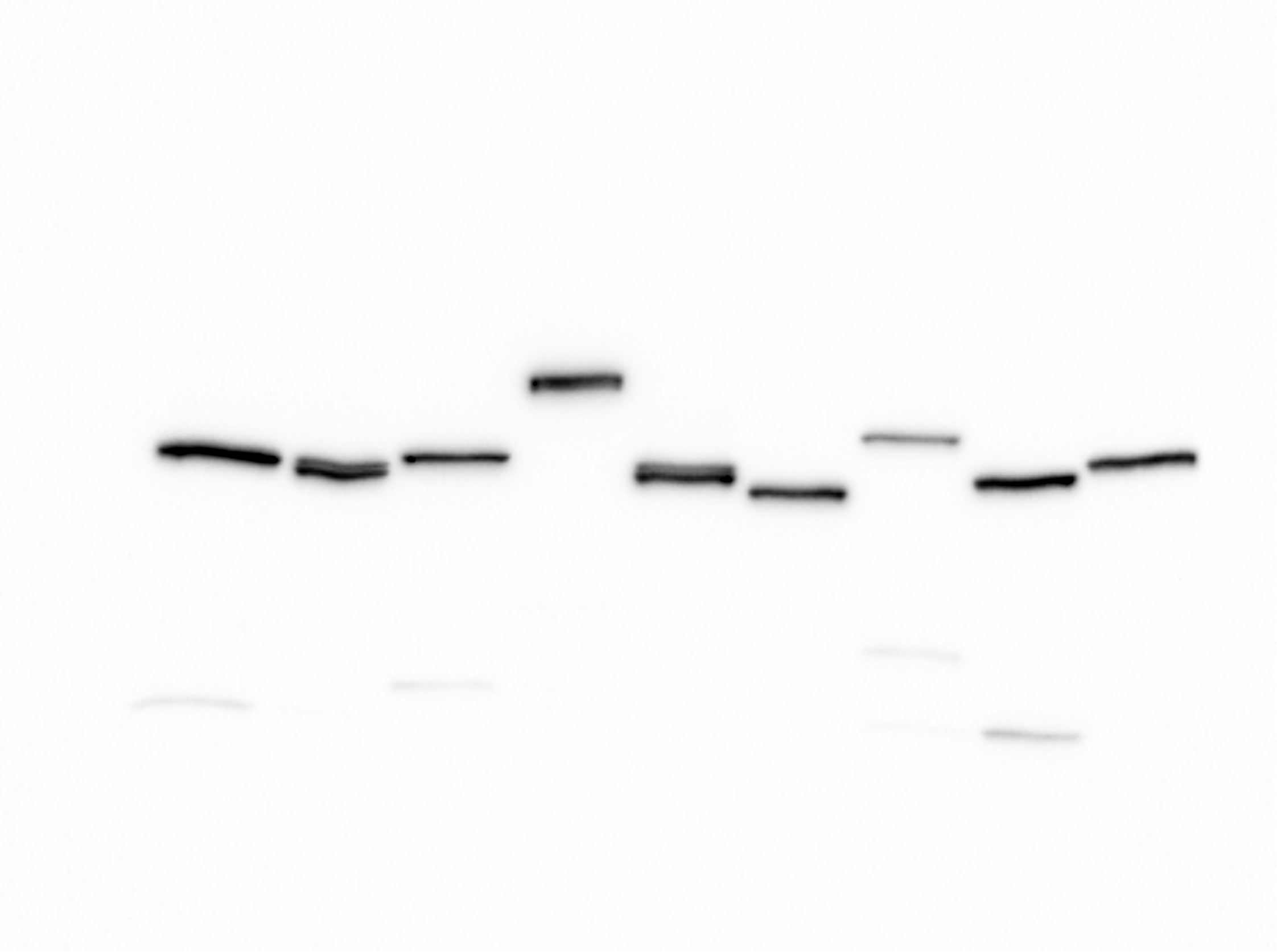

Supplement: Supplementary file 10 — Appendix Figures Source Data [file 44319_2024_203_MOESM10_ESM.zip › Appendix4_RASSF3/Secondrow/Rightmost/Lysate.jpg]

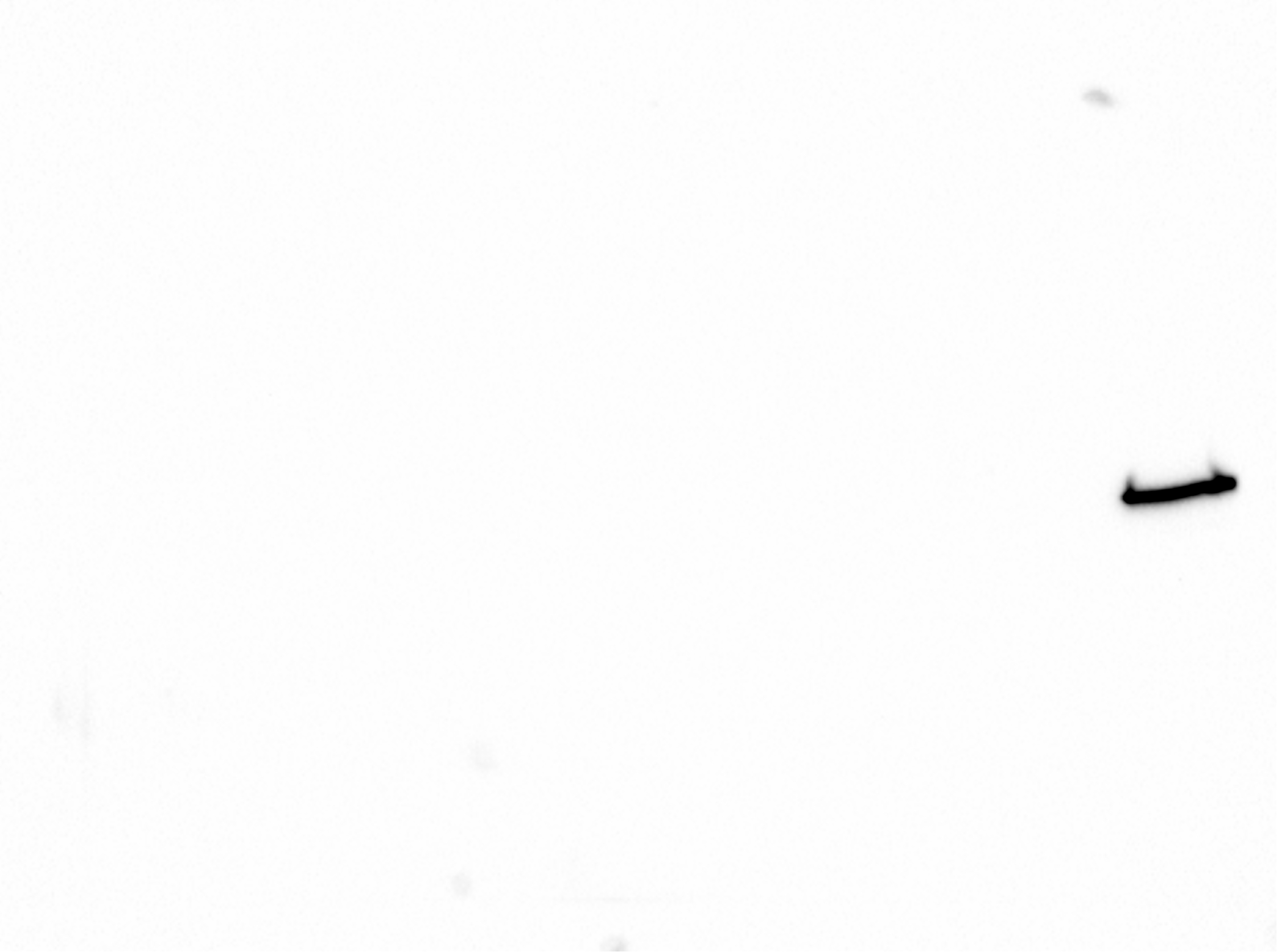

Supplement: Supplementary file 10 — Appendix Figures Source Data [file 44319_2024_203_MOESM10_ESM.zip › Appendix4_RASSF3/Secondrow/Rightmost/Pulldowm.jpg]

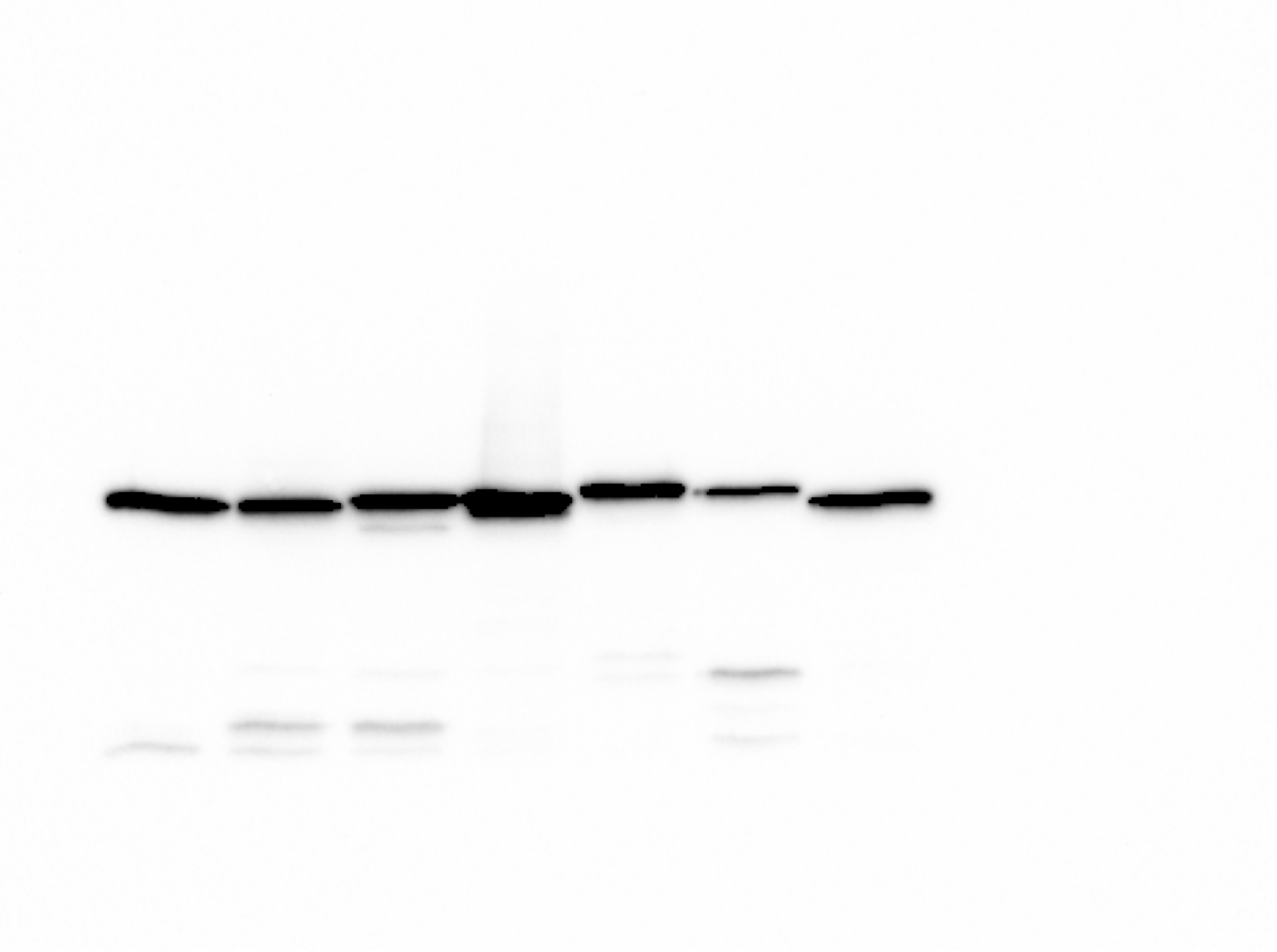

Supplement: Supplementary file 10 — Appendix Figures Source Data [file 44319_2024_203_MOESM10_ESM.zip › Appendix4_RASSF3/Thirdrow/Left/Lysate.jpg]

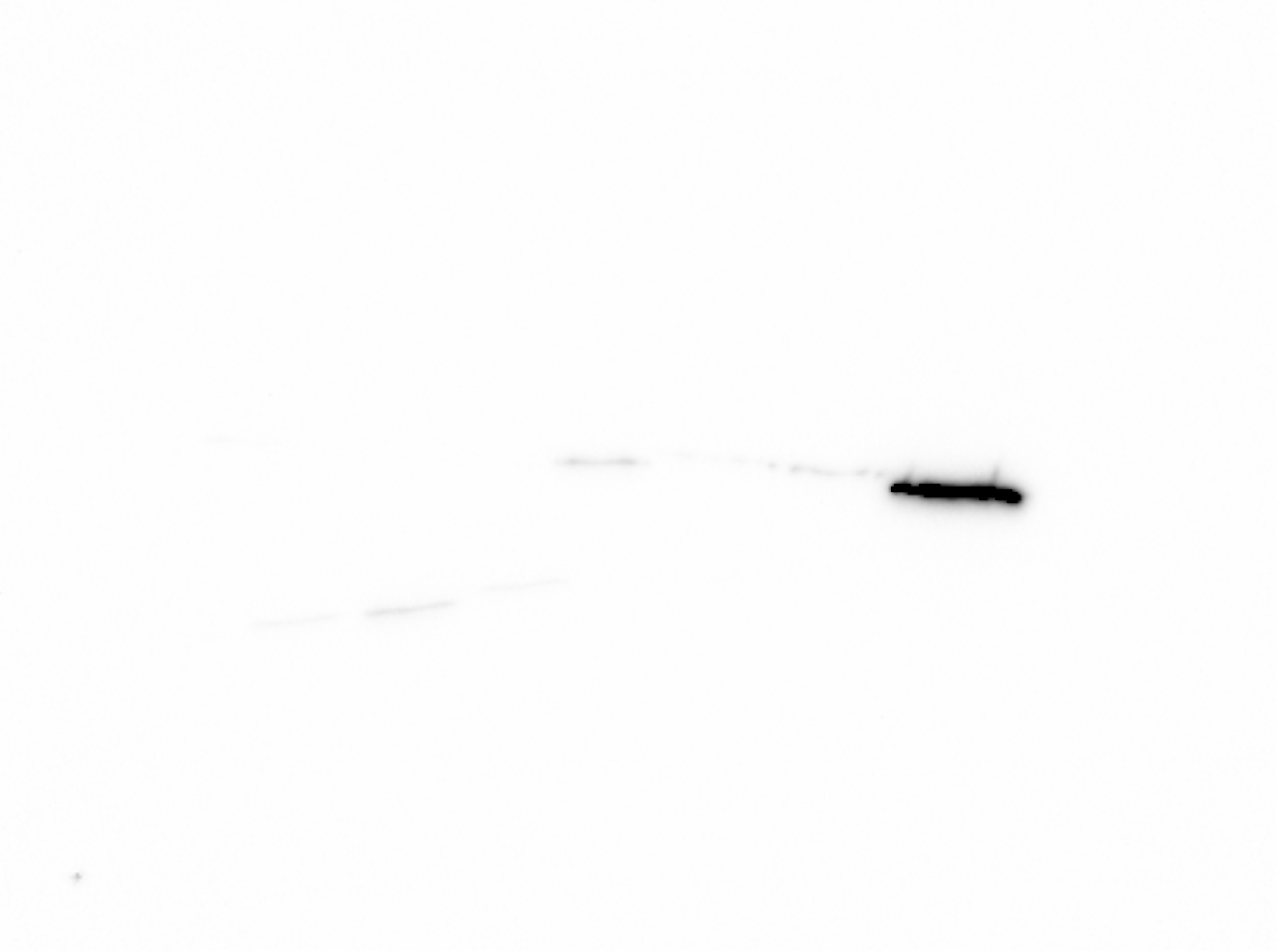

Supplement: Supplementary file 10 — Appendix Figures Source Data [file 44319_2024_203_MOESM10_ESM.zip › Appendix4_RASSF3/Thirdrow/Left/Pulldown.jpg]

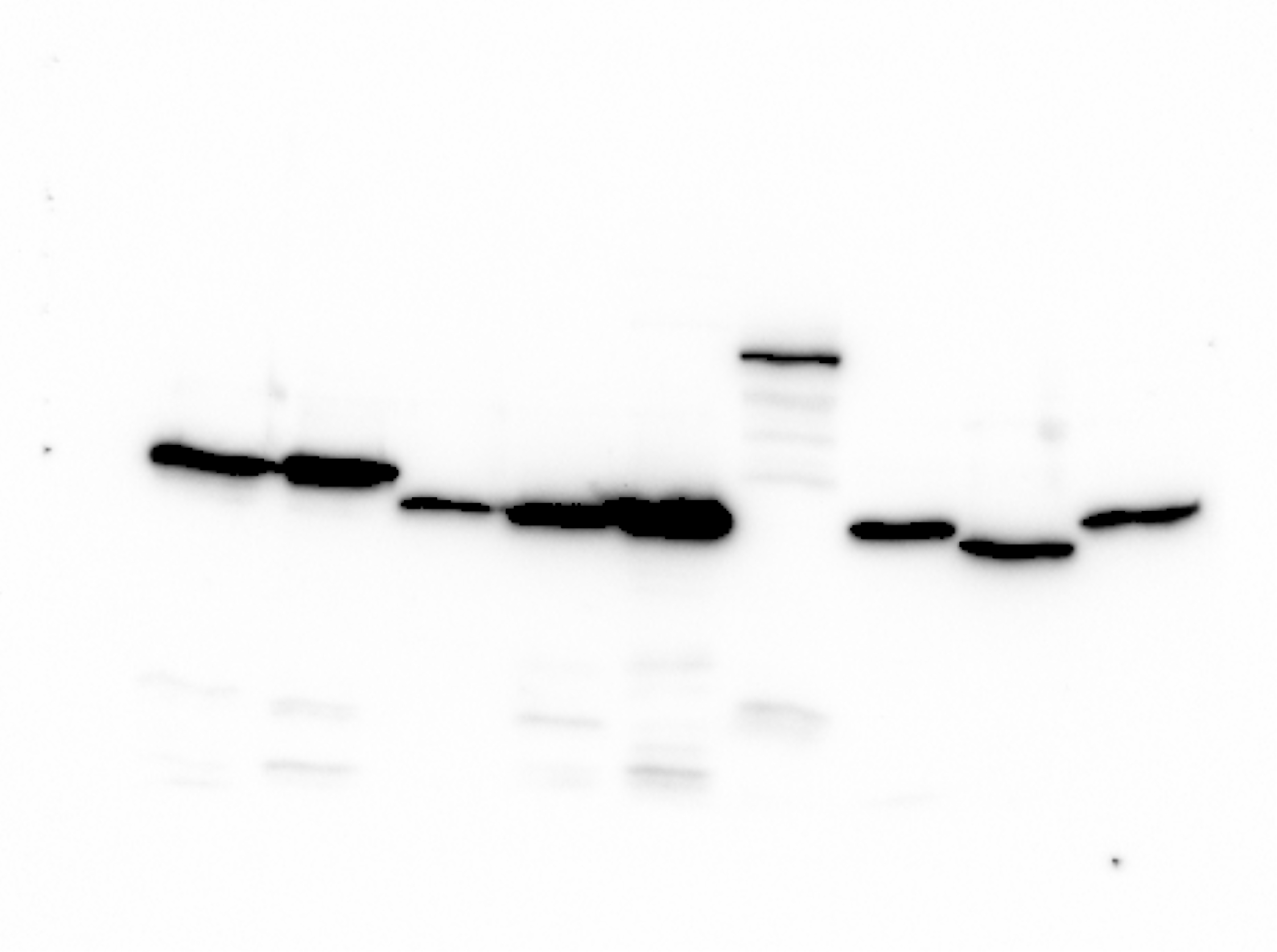

Supplement: Supplementary file 10 — Appendix Figures Source Data [file 44319_2024_203_MOESM10_ESM.zip › Appendix4_RASSF3/Thirdrow/Middle/Lysate.jpg]

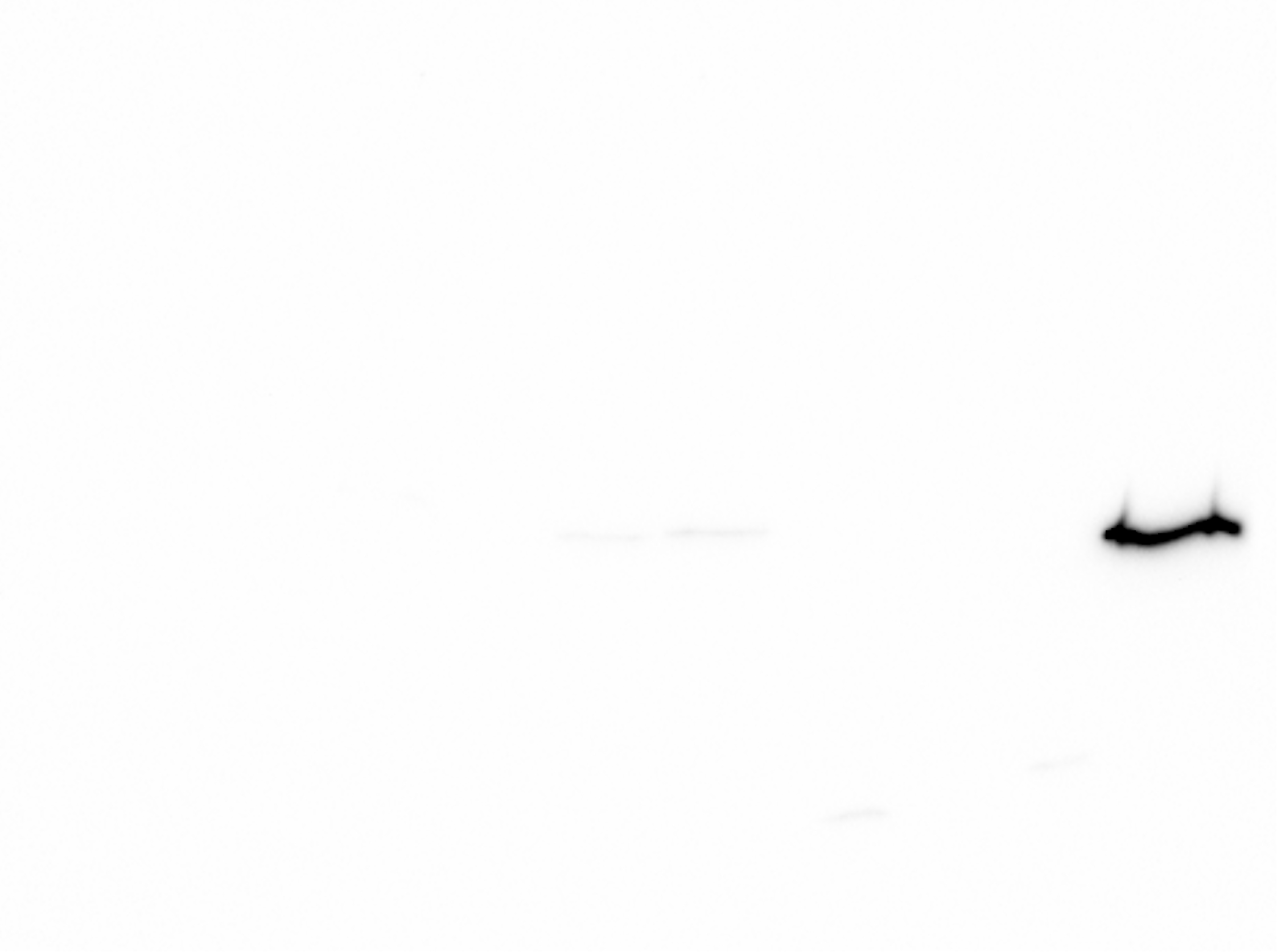

Supplement: Supplementary file 10 — Appendix Figures Source Data [file 44319_2024_203_MOESM10_ESM.zip › Appendix4_RASSF3/Thirdrow/Middle/Pulldown.jpg]

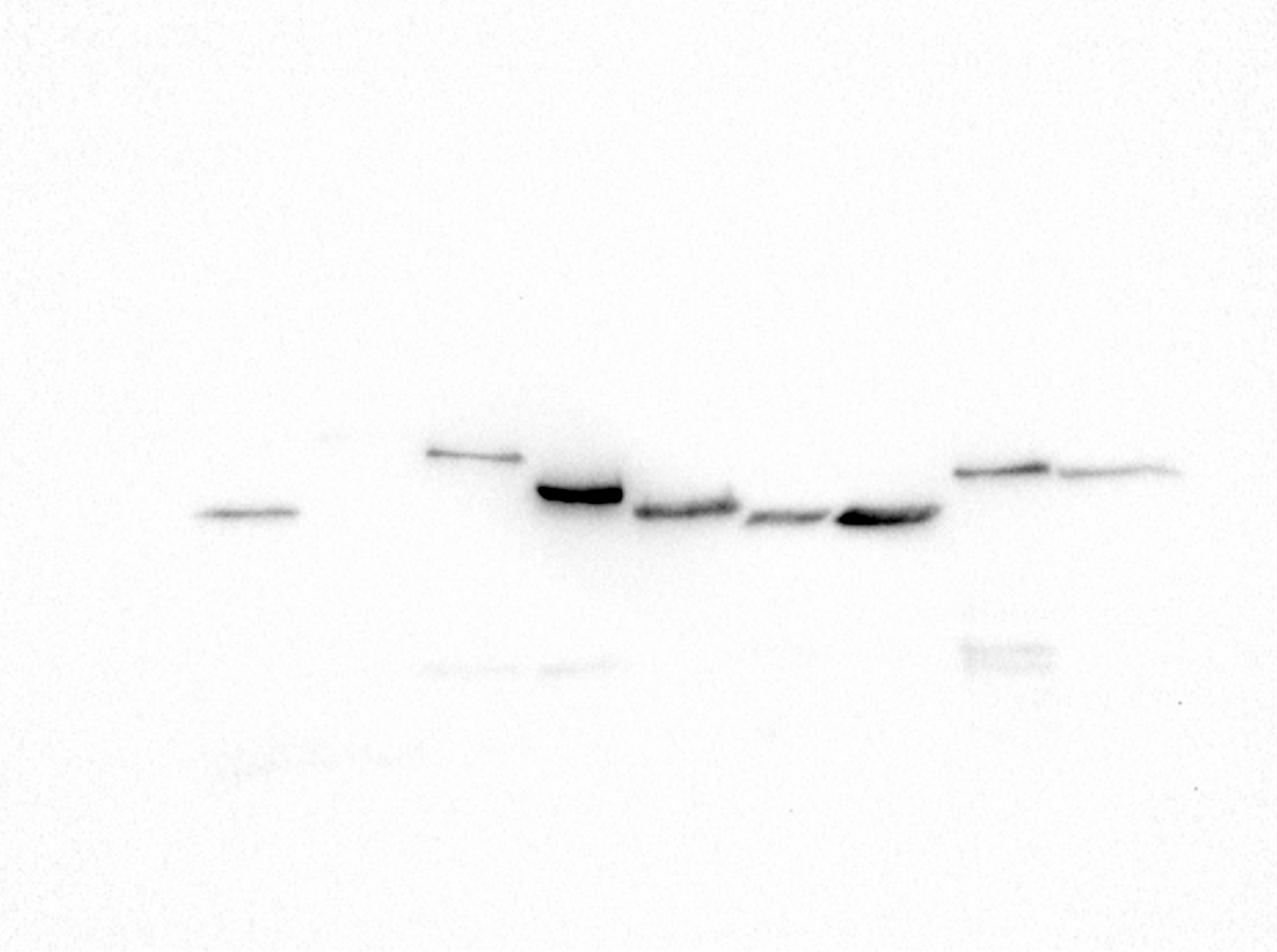

Supplement: Supplementary file 10 — Appendix Figures Source Data [file 44319_2024_203_MOESM10_ESM.zip › Appendix4_RASSF3/Thirdrow/Right/Lysate.jpg]

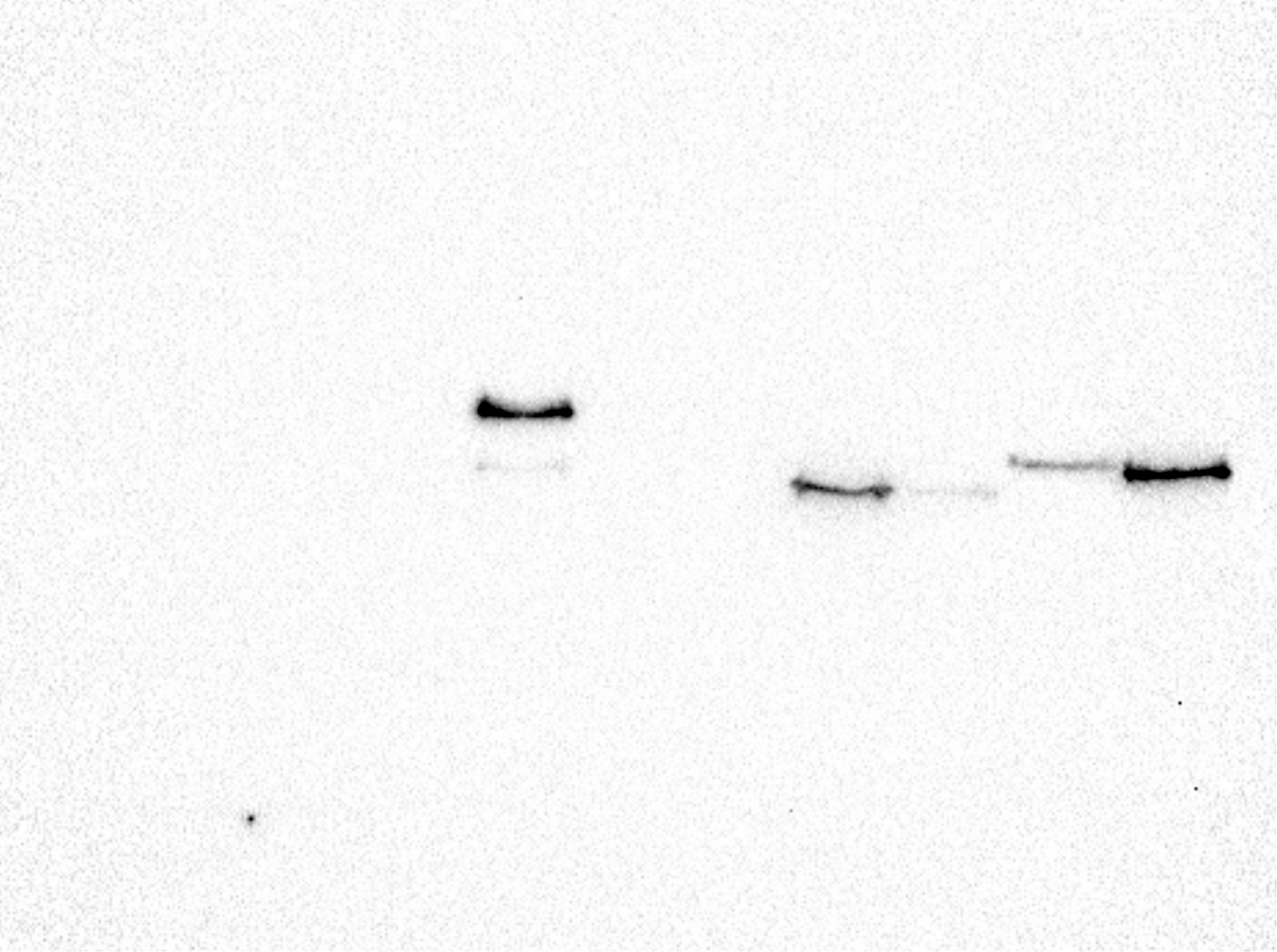

Supplement: Supplementary file 10 — Appendix Figures Source Data [file 44319_2024_203_MOESM10_ESM.zip › Appendix4_RASSF3/Thirdrow/Right/Pulldown.jpg]

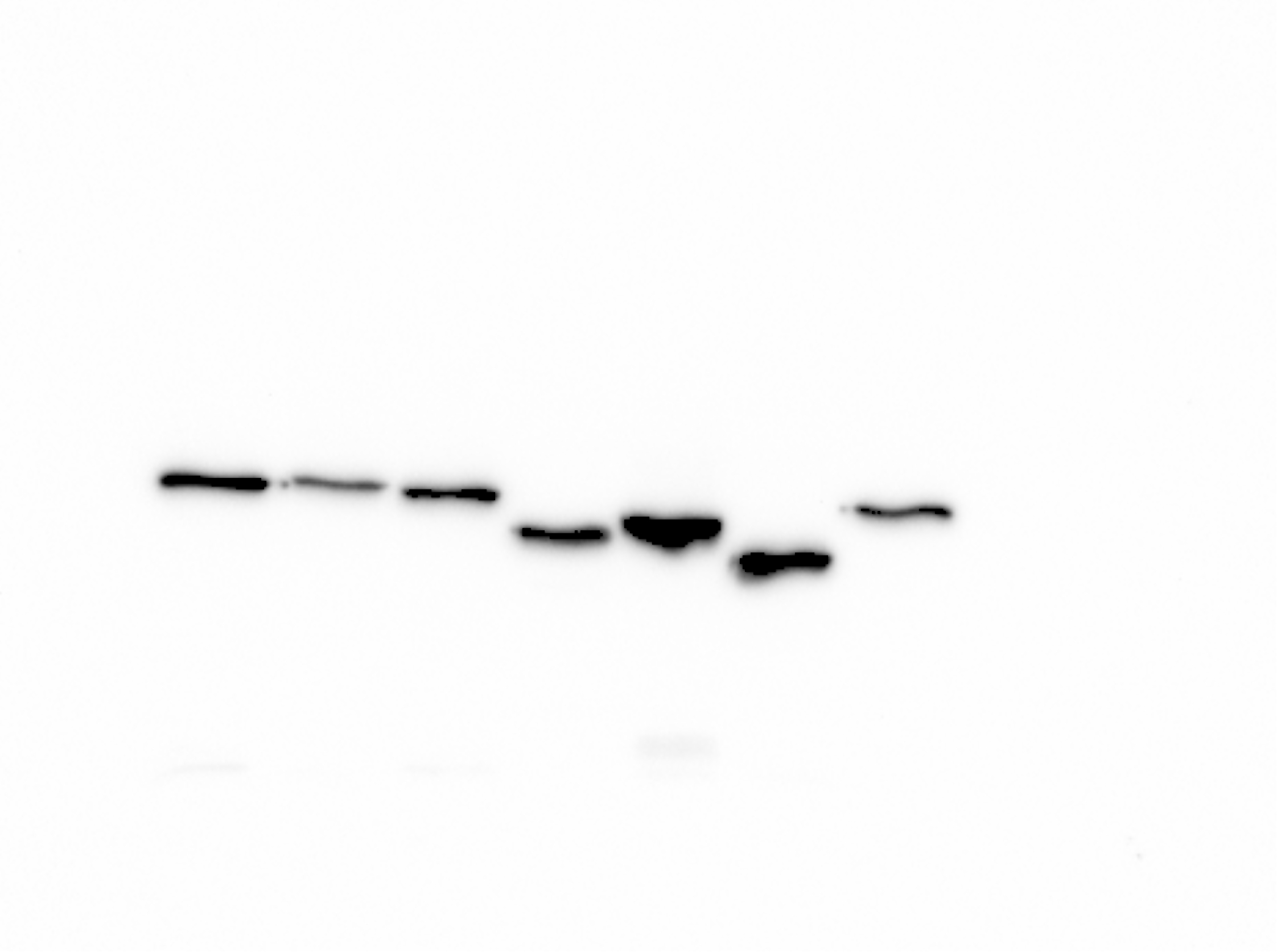

Supplement: Supplementary file 10 — Appendix Figures Source Data [file 44319_2024_203_MOESM10_ESM.zip › Appendix4_RASSF3/Toprow/Leftmost/Lysate.jpg]

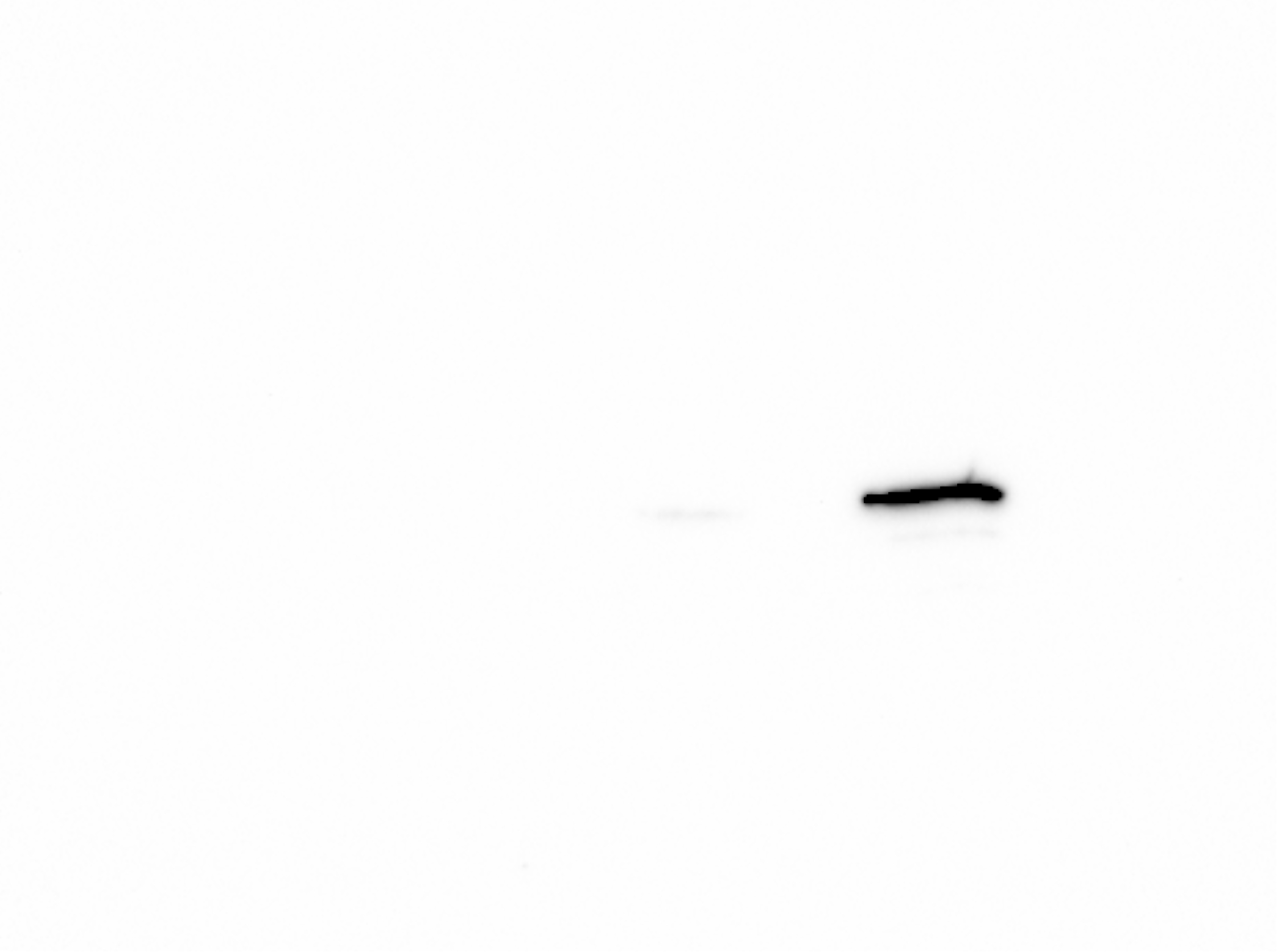

Supplement: Supplementary file 10 — Appendix Figures Source Data [file 44319_2024_203_MOESM10_ESM.zip › Appendix4_RASSF3/Toprow/Leftmost/Pulldown.jpg]

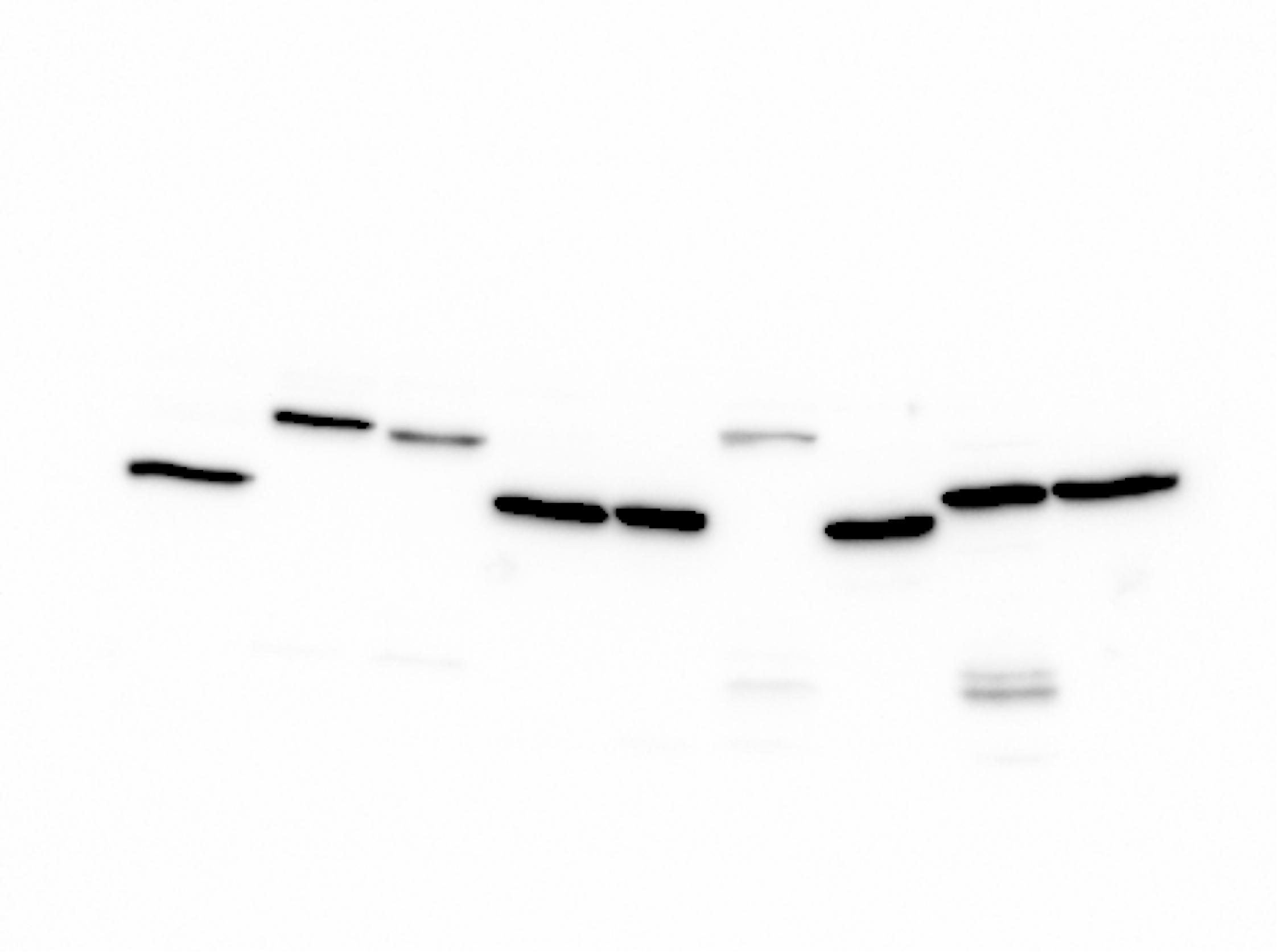

Supplement: Supplementary file 10 — Appendix Figures Source Data [file 44319_2024_203_MOESM10_ESM.zip › Appendix4_RASSF3/Toprow/Middle/Lysate.jpg]

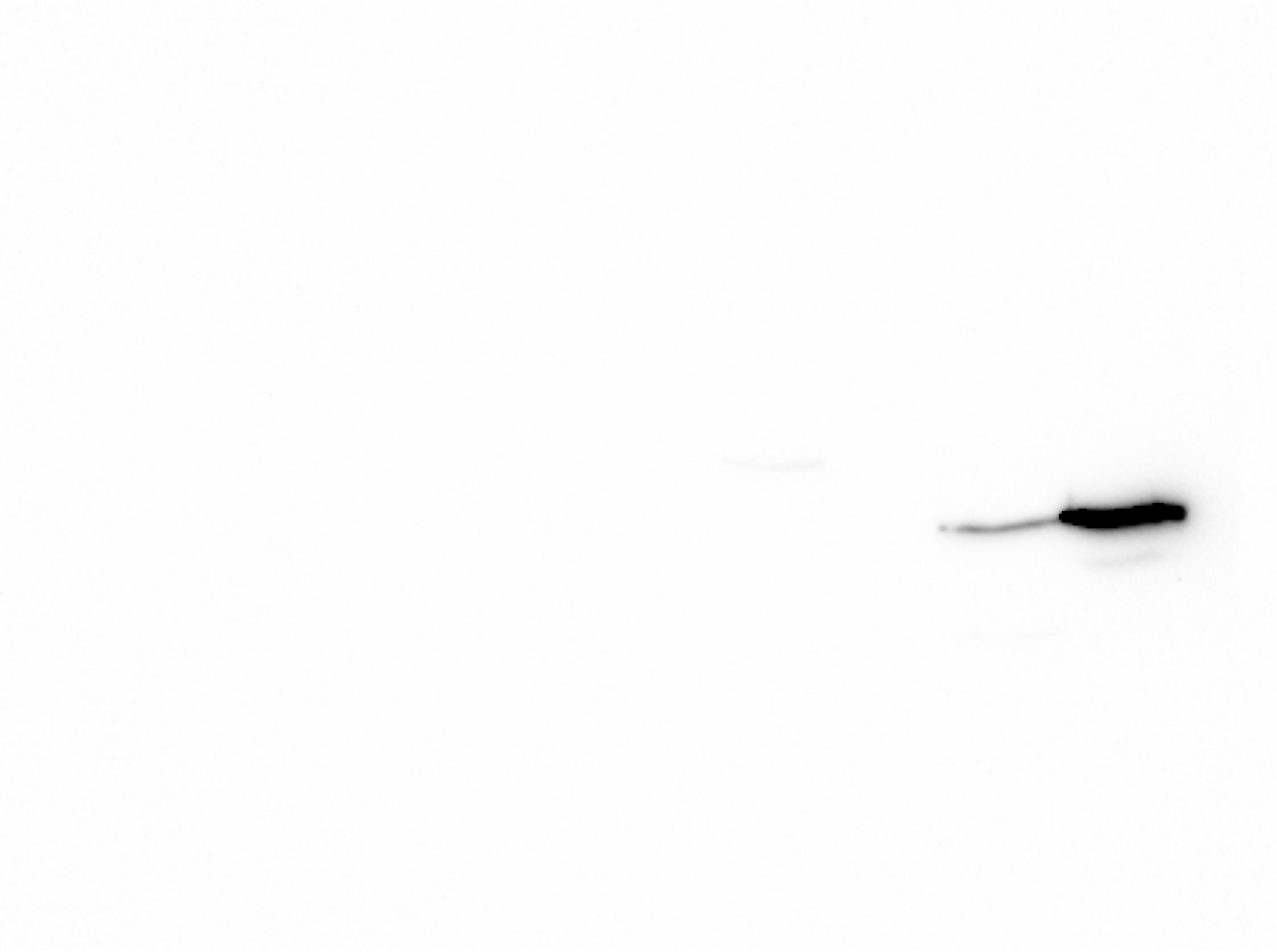

Supplement: Supplementary file 10 — Appendix Figures Source Data [file 44319_2024_203_MOESM10_ESM.zip › Appendix4_RASSF3/Toprow/Middle/Pulldown.jpg]

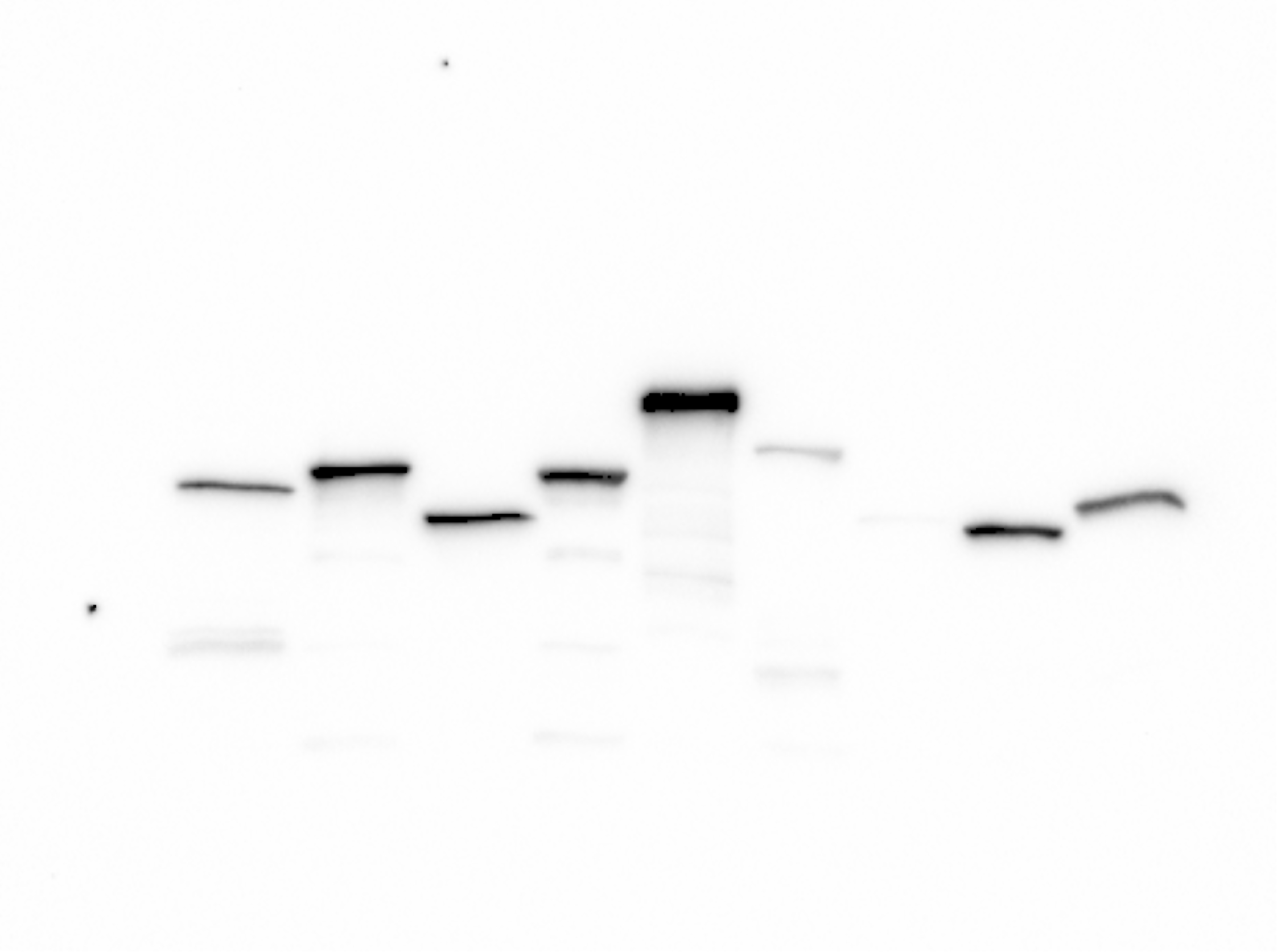

Supplement: Supplementary file 10 — Appendix Figures Source Data [file 44319_2024_203_MOESM10_ESM.zip › Appendix4_RASSF3/Toprow/Rightmost/Lysate.jpg]

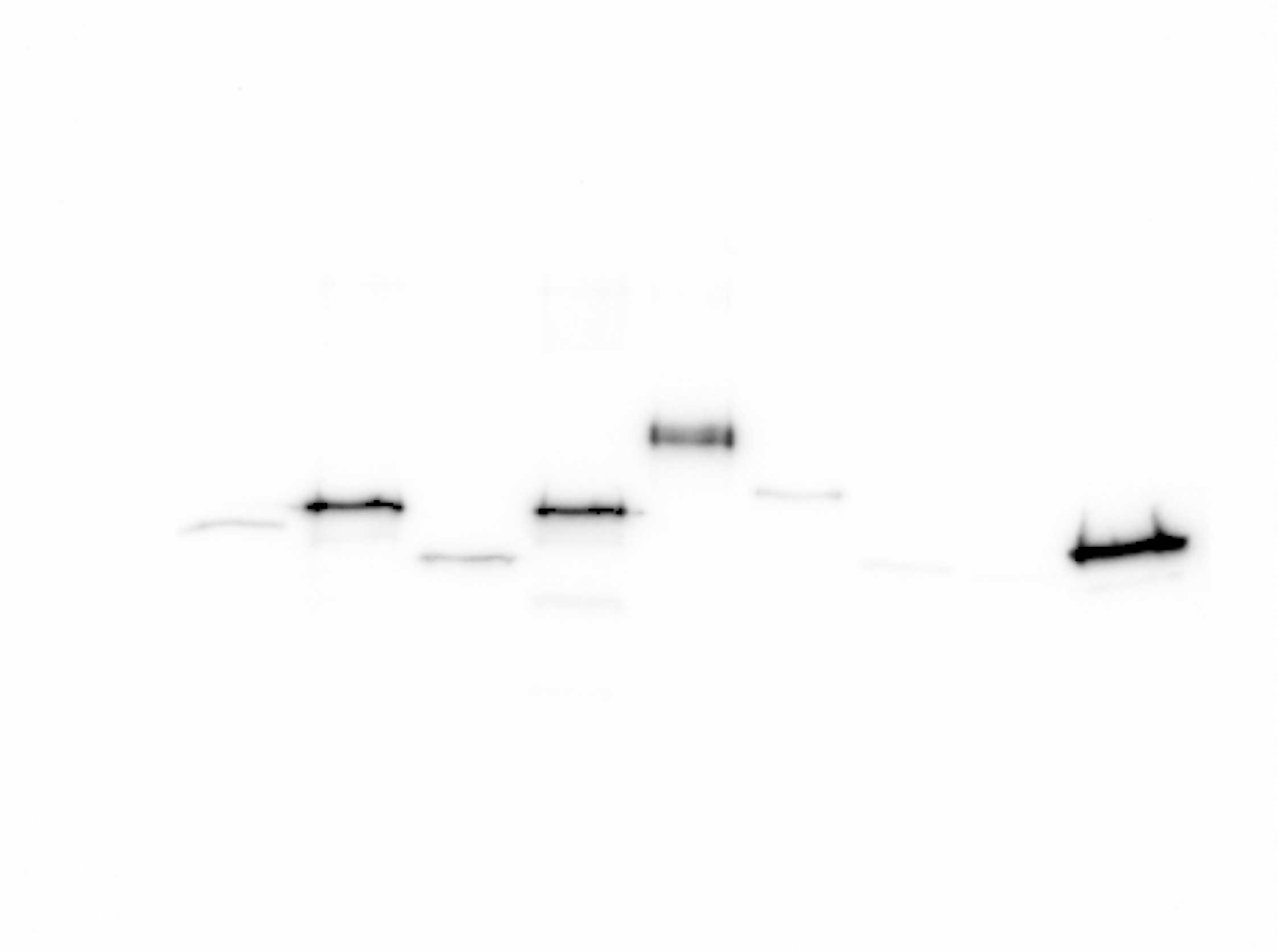

Supplement: Supplementary file 10 — Appendix Figures Source Data [file 44319_2024_203_MOESM10_ESM.zip › Appendix4_RASSF3/Toprow/Rightmost/Pulldown.jpg]

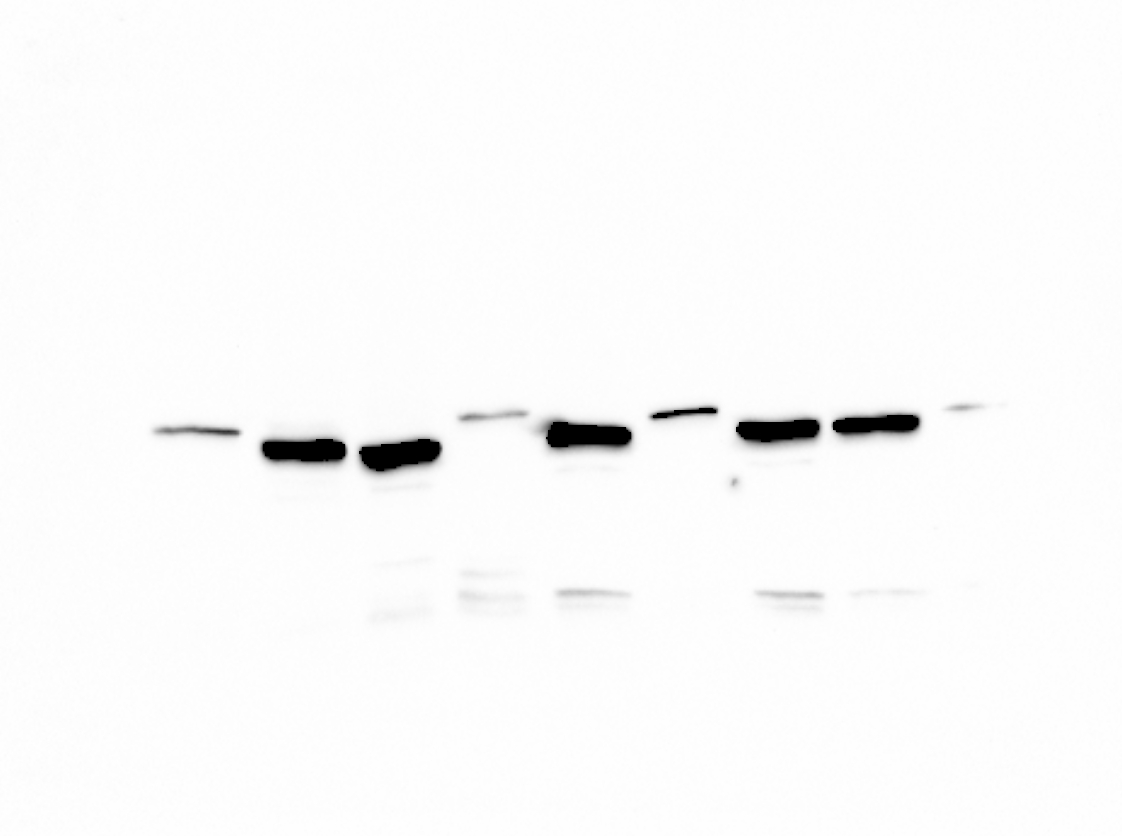

Supplement: Supplementary file 10 — Appendix Figures Source Data [file 44319_2024_203_MOESM10_ESM.zip › Appendix2_BRAF/fourthrow/leftmost/Lysate.jpg]

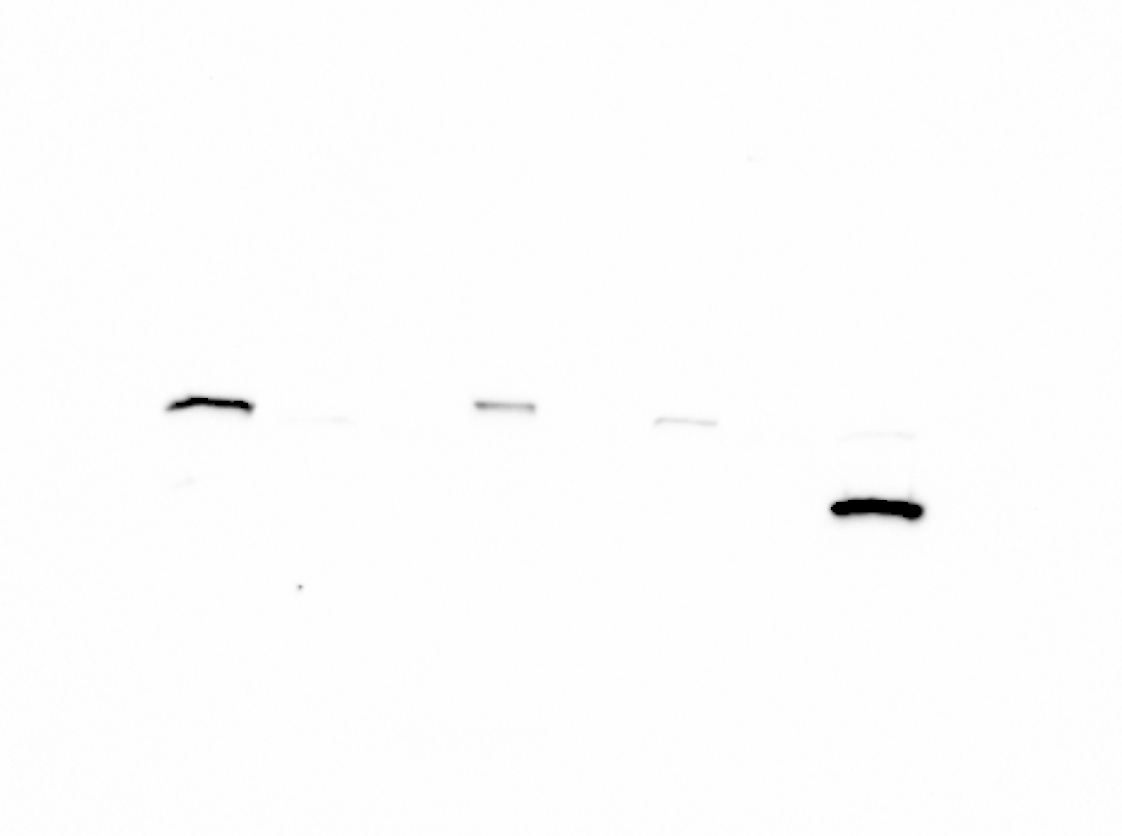

Supplement: Supplementary file 10 — Appendix Figures Source Data [file 44319_2024_203_MOESM10_ESM.zip › Appendix2_BRAF/fourthrow/leftmost/Pulldown.jpg]

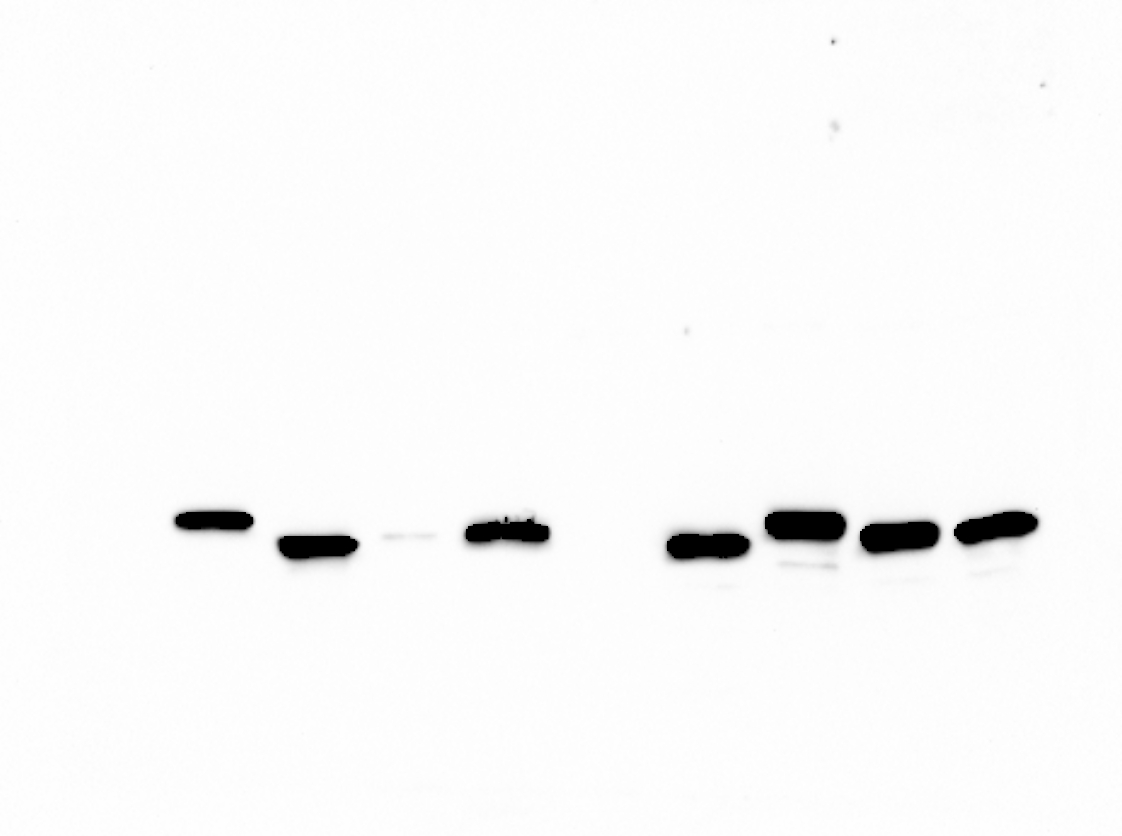

Supplement: Supplementary file 10 — Appendix Figures Source Data [file 44319_2024_203_MOESM10_ESM.zip › Appendix2_BRAF/fourthrow/middle/Lysate.jpg]

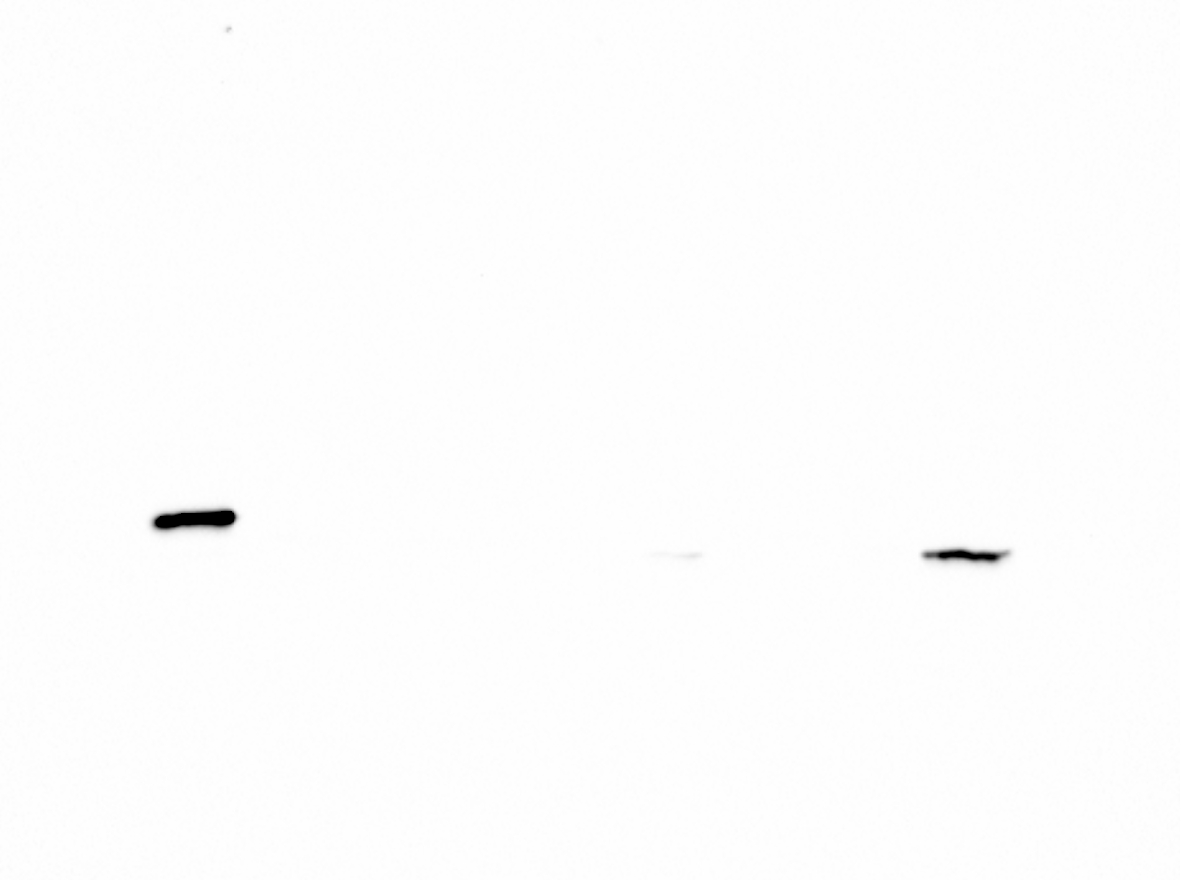

Supplement: Supplementary file 10 — Appendix Figures Source Data [file 44319_2024_203_MOESM10_ESM.zip › Appendix2_BRAF/fourthrow/middle/Pulldown.jpg]

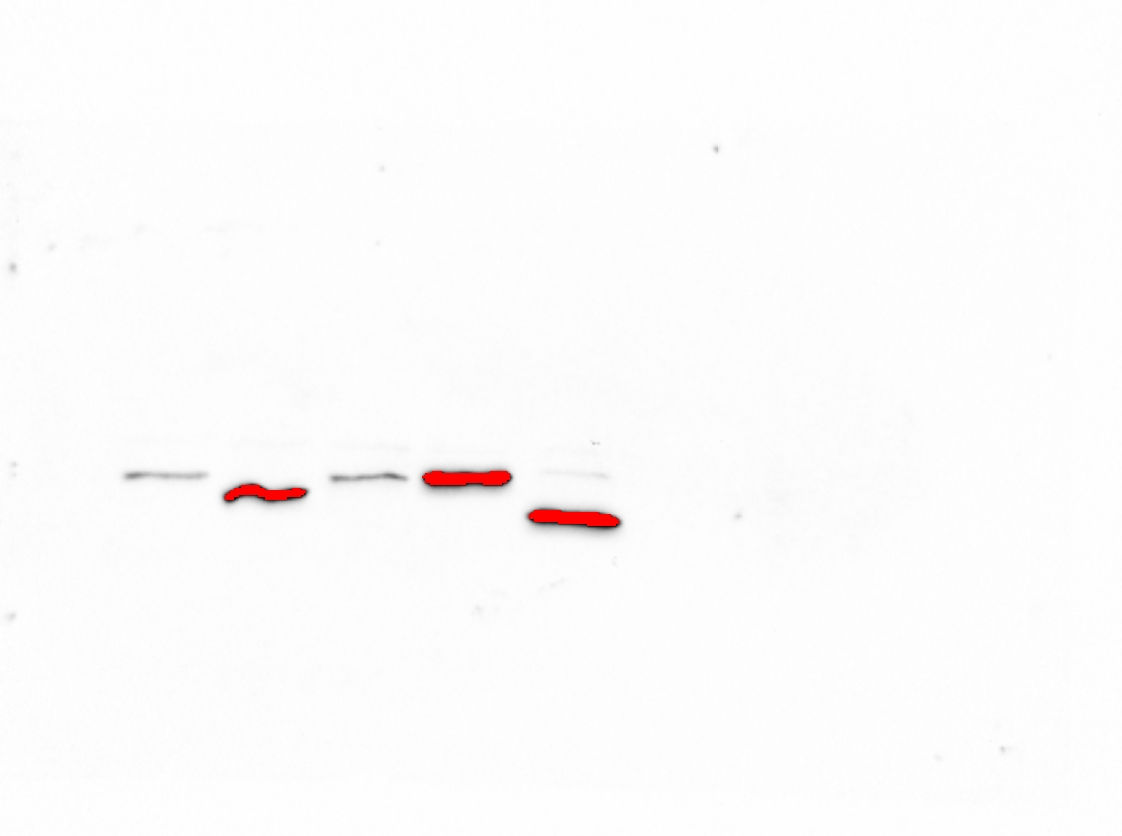

Supplement: Supplementary file 10 — Appendix Figures Source Data [file 44319_2024_203_MOESM10_ESM.zip › Appendix2_BRAF/fourthrow/rightmost/Lysate.jpg]

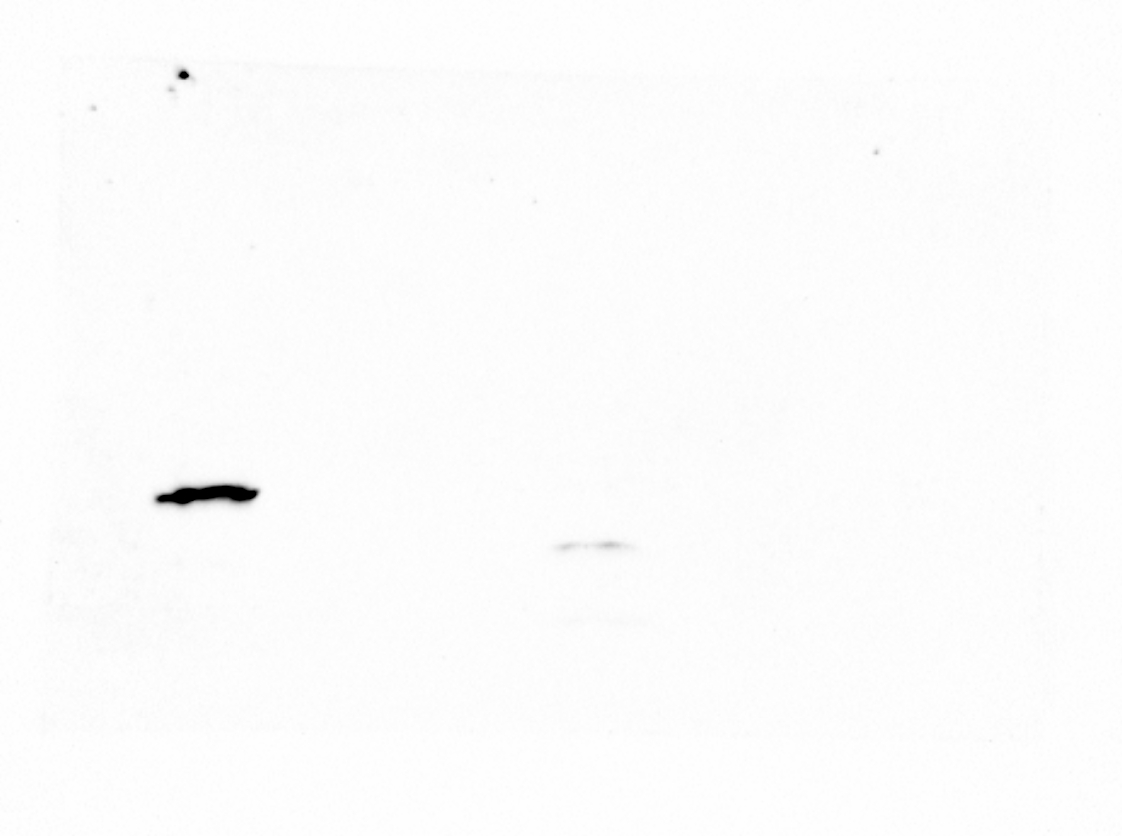

Supplement: Supplementary file 10 — Appendix Figures Source Data [file 44319_2024_203_MOESM10_ESM.zip › Appendix2_BRAF/fourthrow/rightmost/Pulldown.jpg]

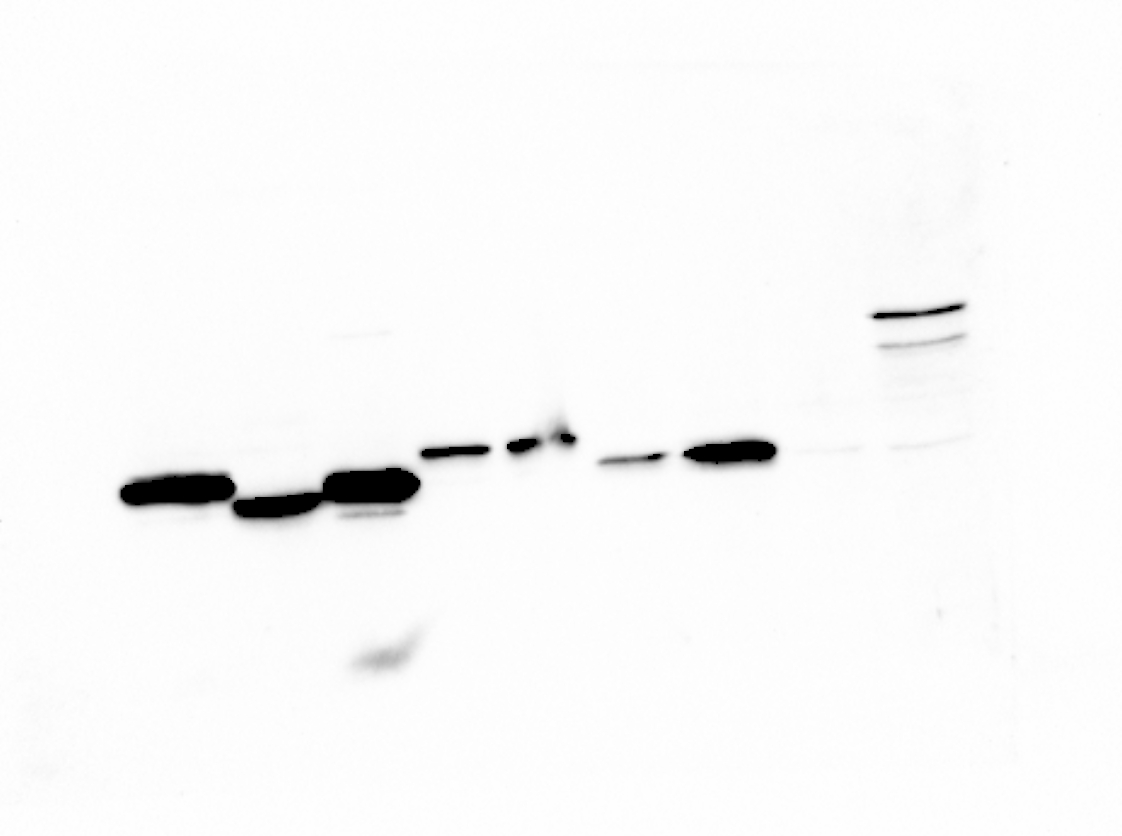

Supplement: Supplementary file 10 — Appendix Figures Source Data [file 44319_2024_203_MOESM10_ESM.zip › Appendix2_BRAF/secondrow/leftmost/Lysate.jpg]

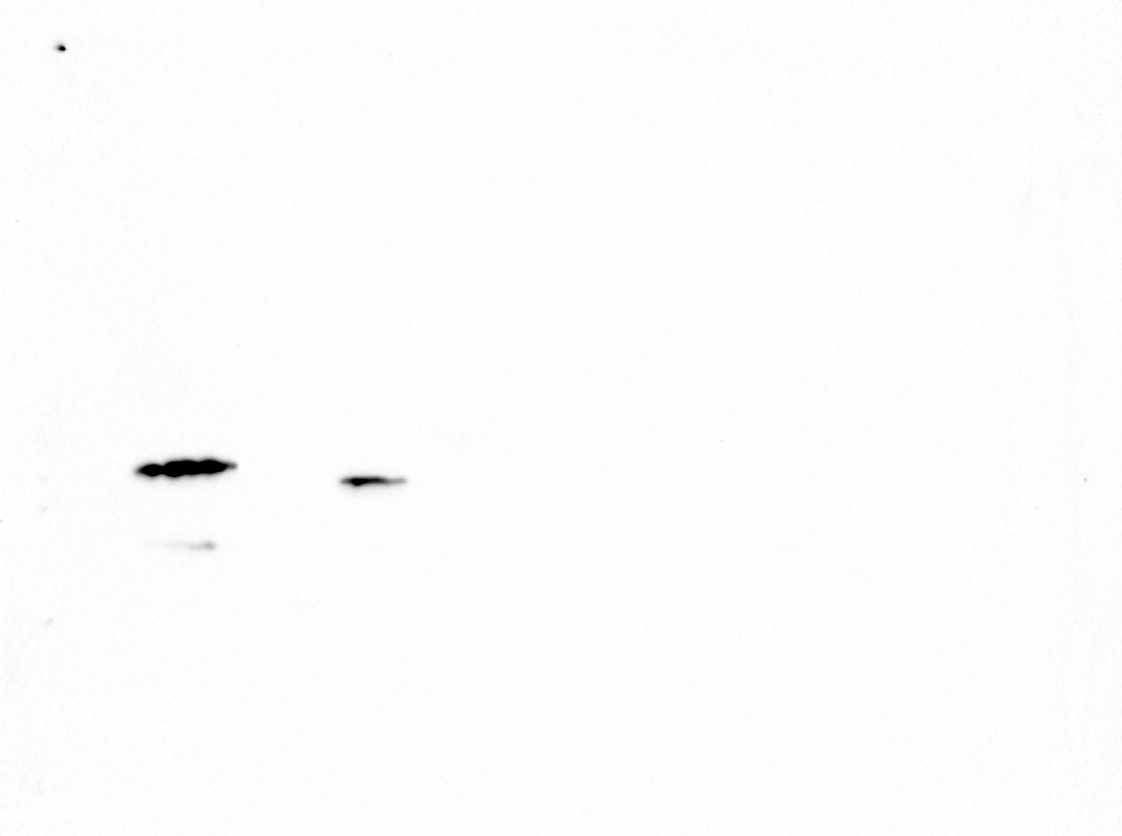

Supplement: Supplementary file 10 — Appendix Figures Source Data [file 44319_2024_203_MOESM10_ESM.zip › Appendix2_BRAF/secondrow/leftmost/Pulldown.jpg]

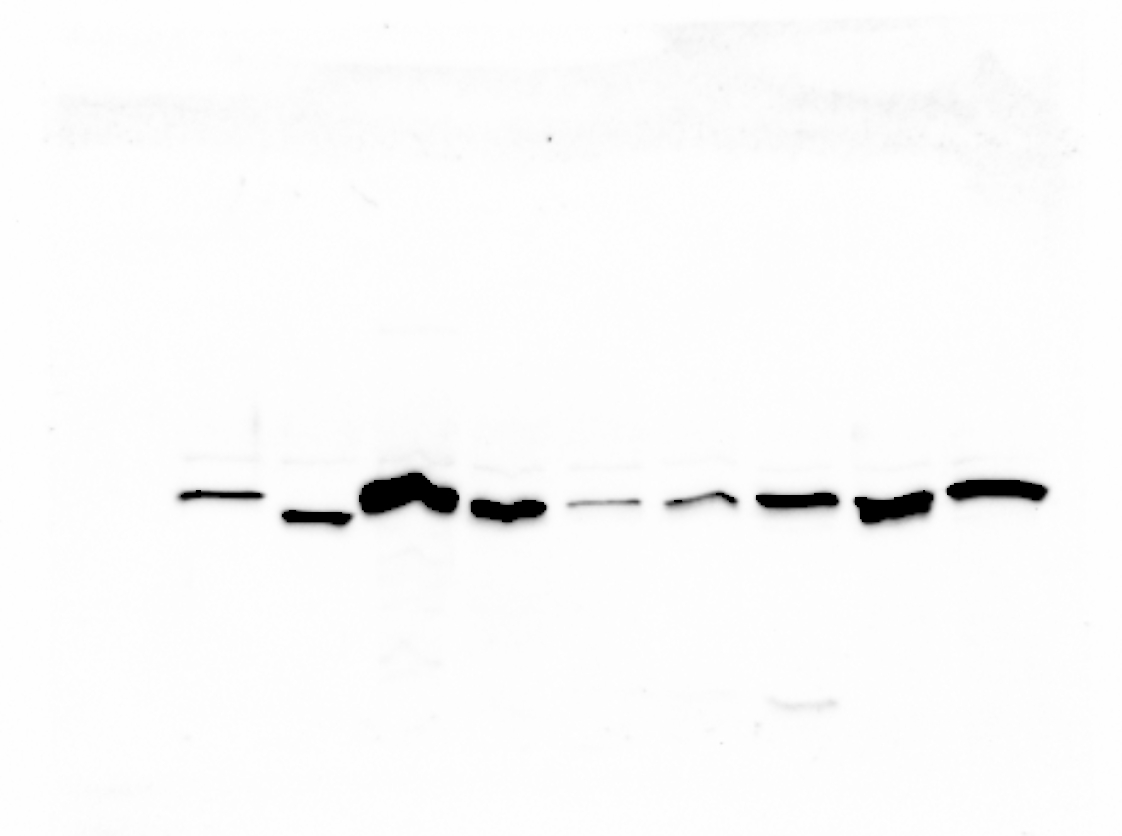

Supplement: Supplementary file 10 — Appendix Figures Source Data [file 44319_2024_203_MOESM10_ESM.zip › Appendix2_BRAF/secondrow/middle/Lysate.jpg]

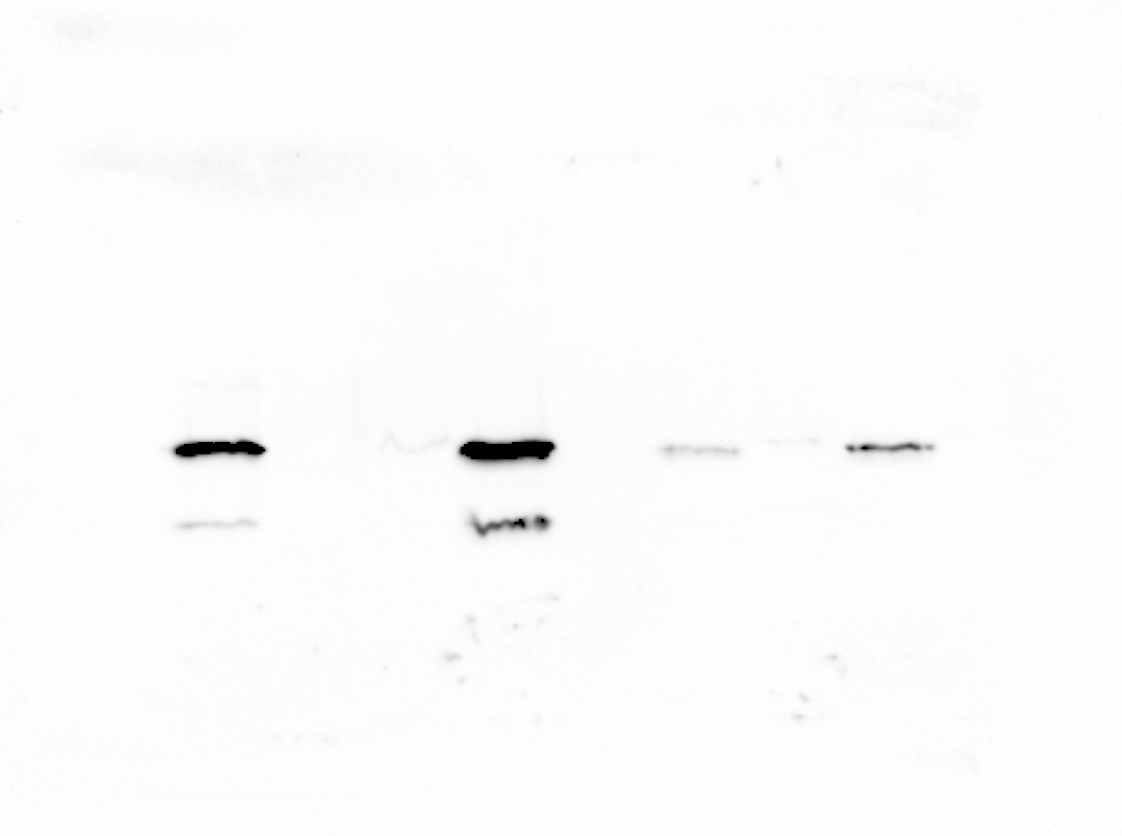

Supplement: Supplementary file 10 — Appendix Figures Source Data [file 44319_2024_203_MOESM10_ESM.zip › Appendix2_BRAF/secondrow/middle/Pulldown.jpg]

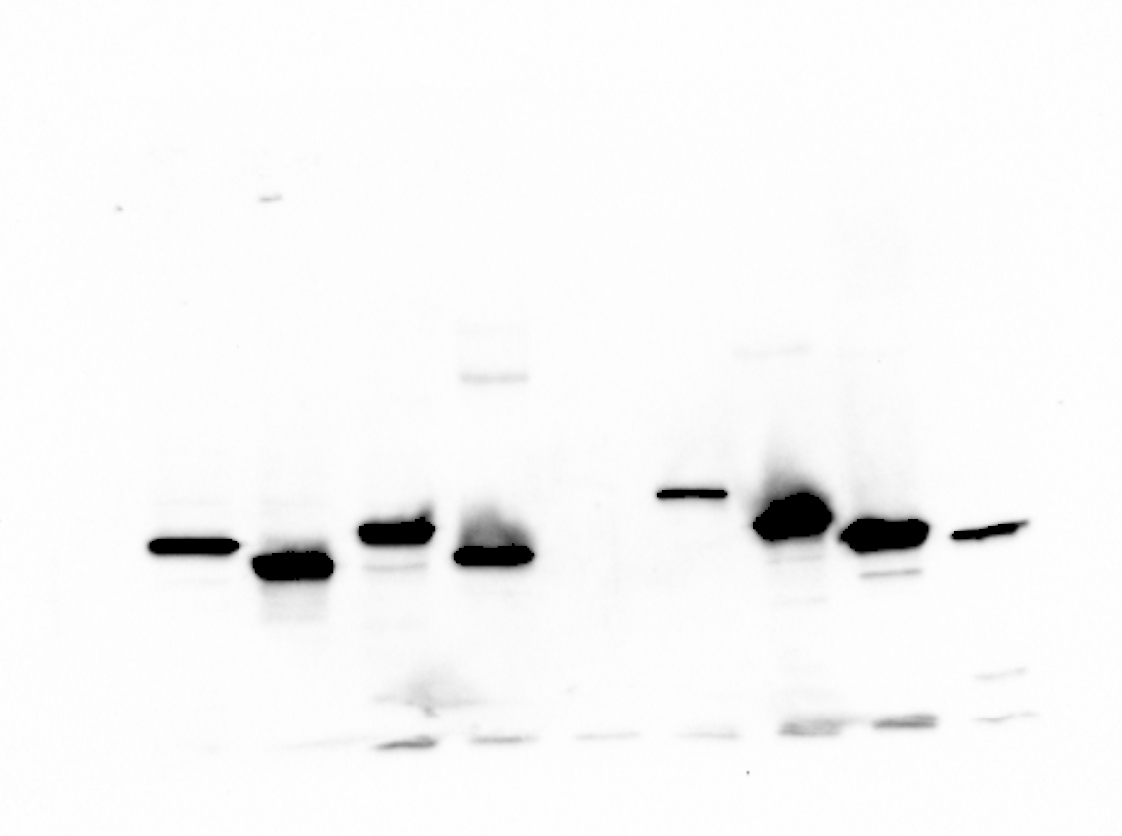

Supplement: Supplementary file 10 — Appendix Figures Source Data [file 44319_2024_203_MOESM10_ESM.zip › Appendix2_BRAF/secondrow/rightmost/Lysate.jpg]

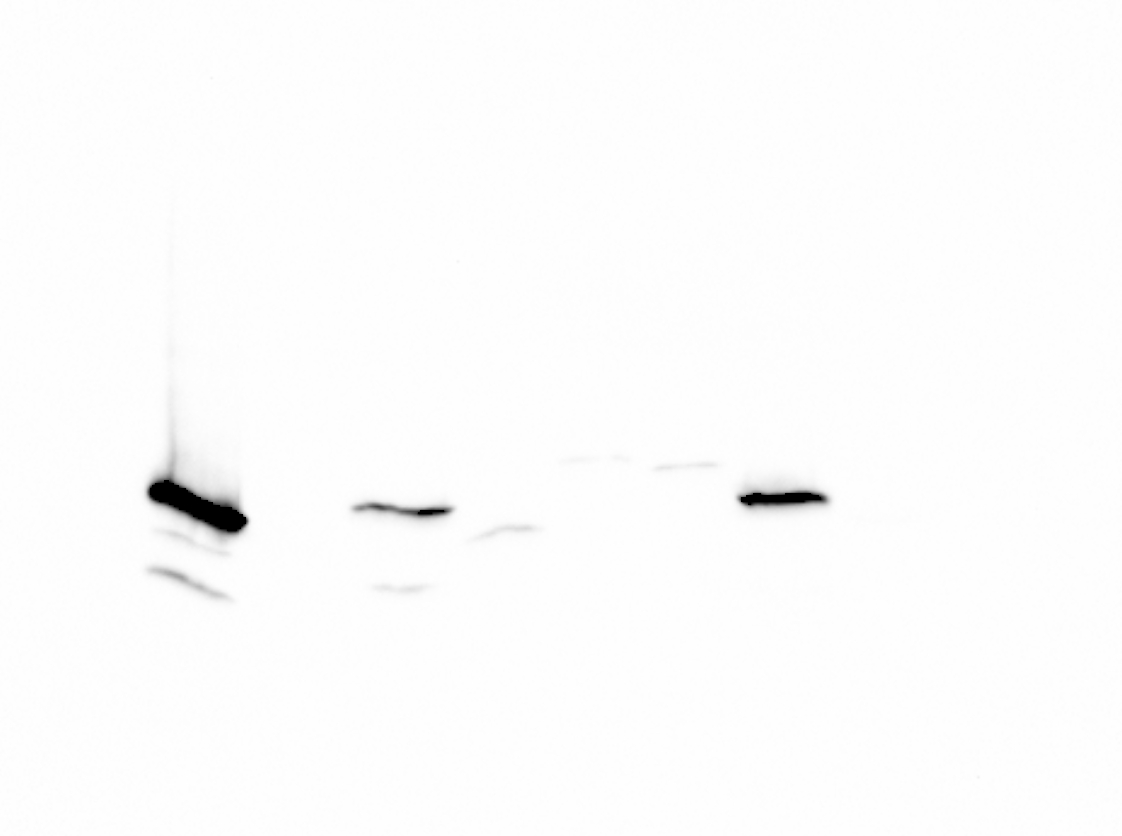

Supplement: Supplementary file 10 — Appendix Figures Source Data [file 44319_2024_203_MOESM10_ESM.zip › Appendix2_BRAF/secondrow/rightmost/Pulldown.jpg]

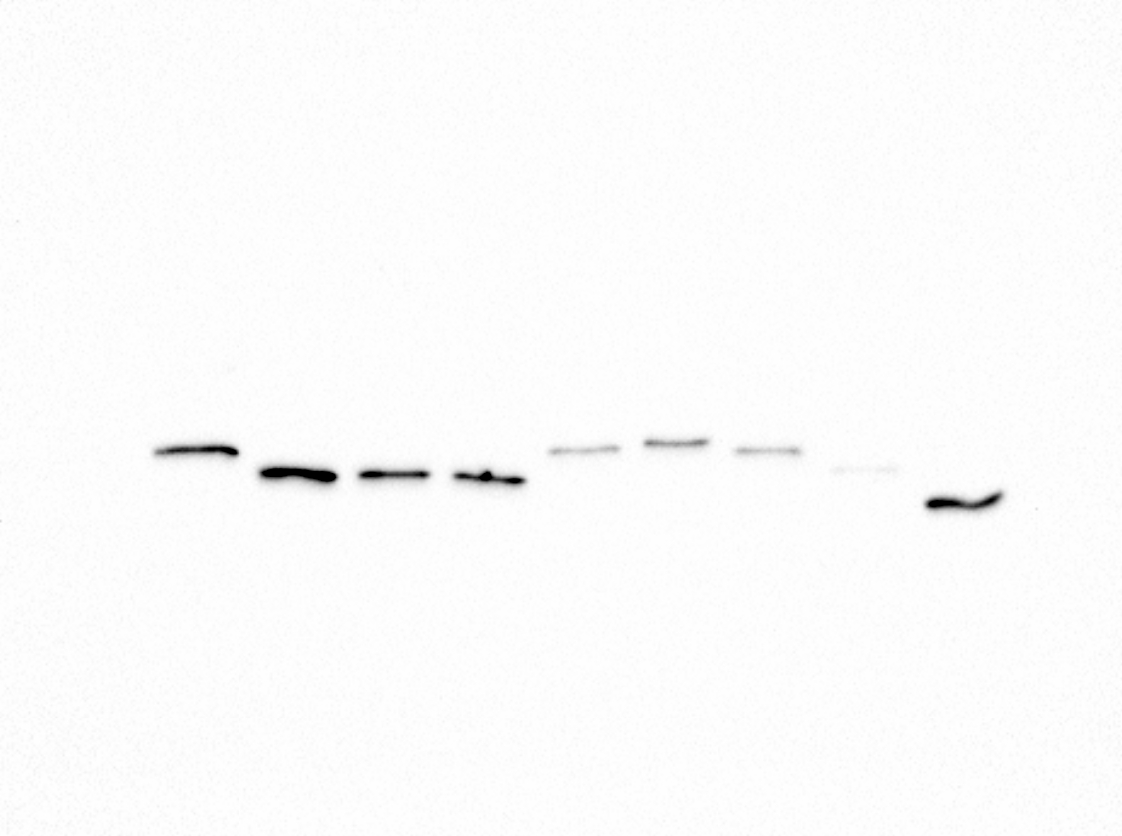

Supplement: Supplementary file 10 — Appendix Figures Source Data [file 44319_2024_203_MOESM10_ESM.zip › Appendix2_BRAF/thirdrow/leftmost/Lysate.jpg]

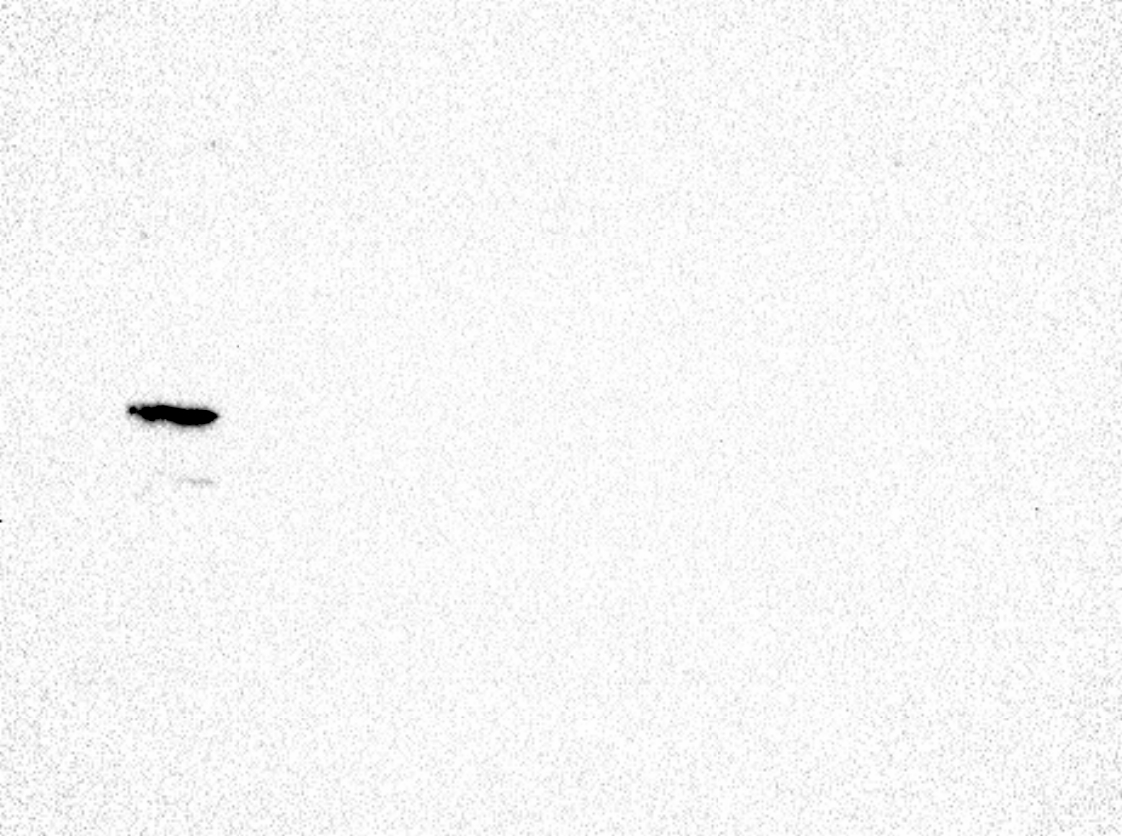

Supplement: Supplementary file 10 — Appendix Figures Source Data [file 44319_2024_203_MOESM10_ESM.zip › Appendix2_BRAF/thirdrow/leftmost/Pulldown.jpg]

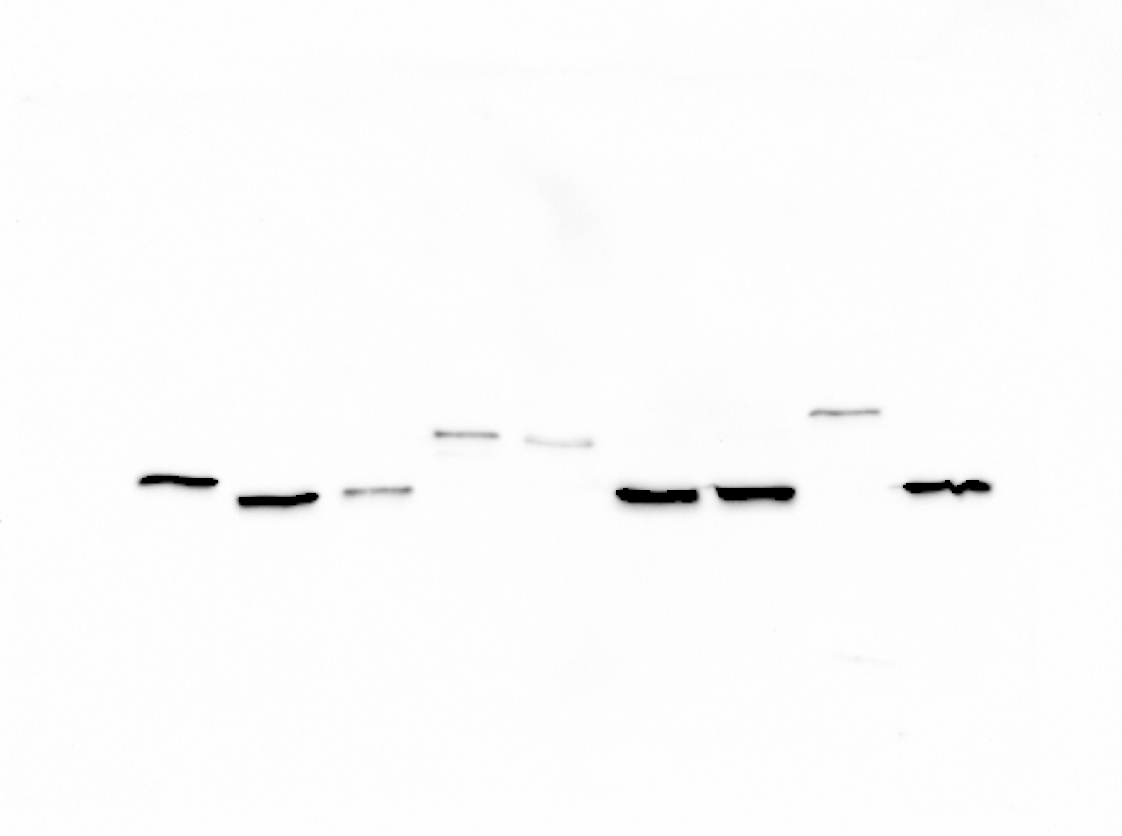

Supplement: Supplementary file 10 — Appendix Figures Source Data [file 44319_2024_203_MOESM10_ESM.zip › Appendix2_BRAF/thirdrow/middle/lysate.jpg]

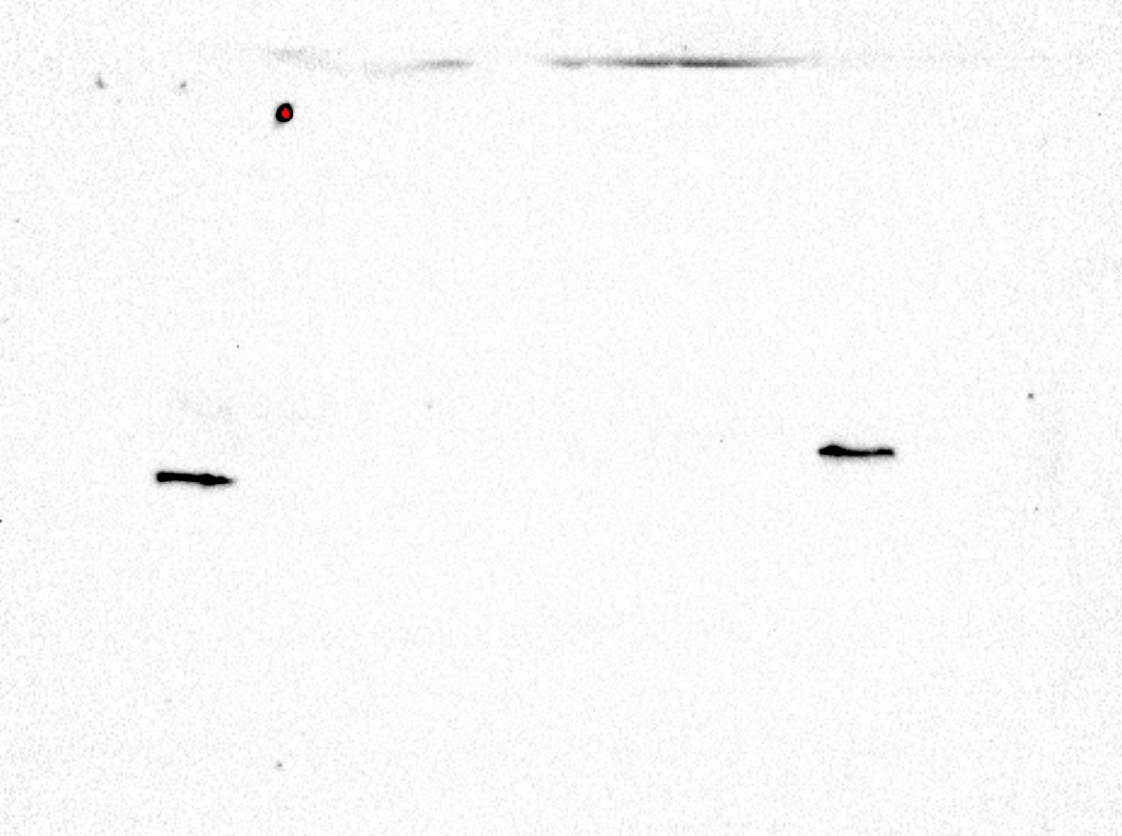

Supplement: Supplementary file 10 — Appendix Figures Source Data [file 44319_2024_203_MOESM10_ESM.zip › Appendix2_BRAF/thirdrow/middle/Pulldown.jpg]

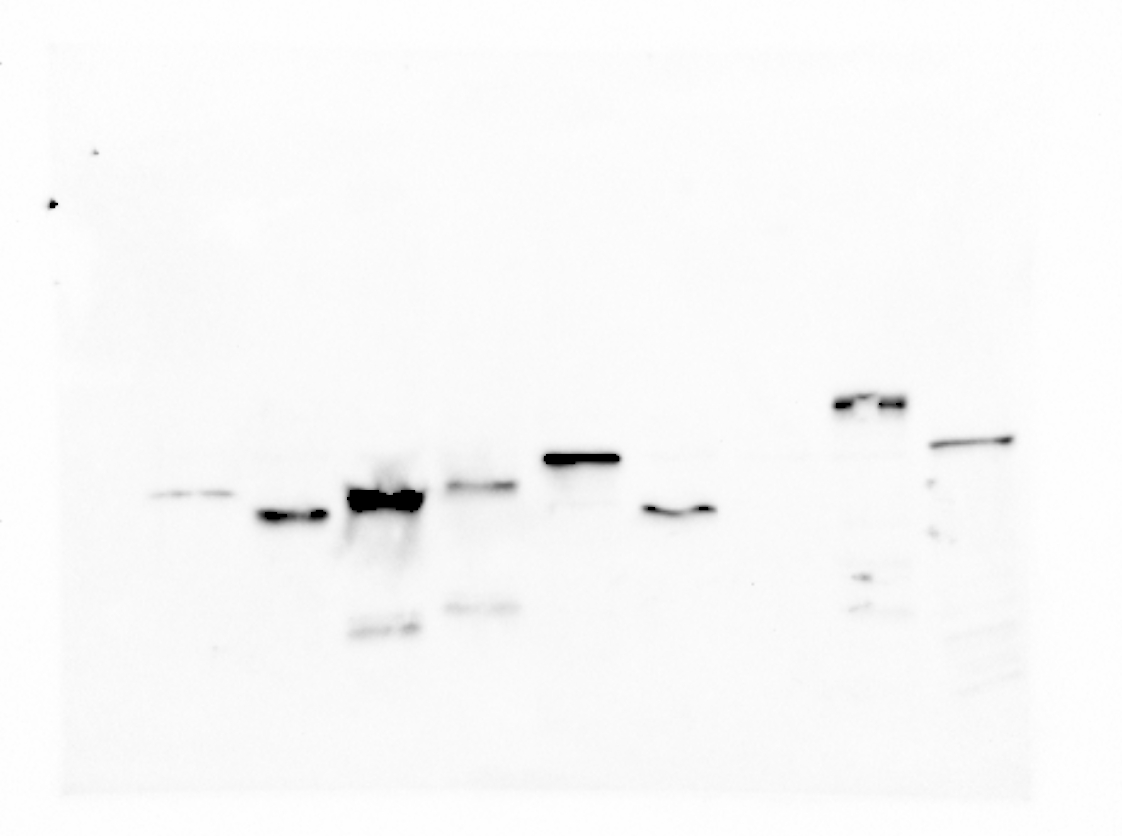

Supplement: Supplementary file 10 — Appendix Figures Source Data [file 44319_2024_203_MOESM10_ESM.zip › Appendix2_BRAF/thirdrow/rightmost/Lysate.jpg]

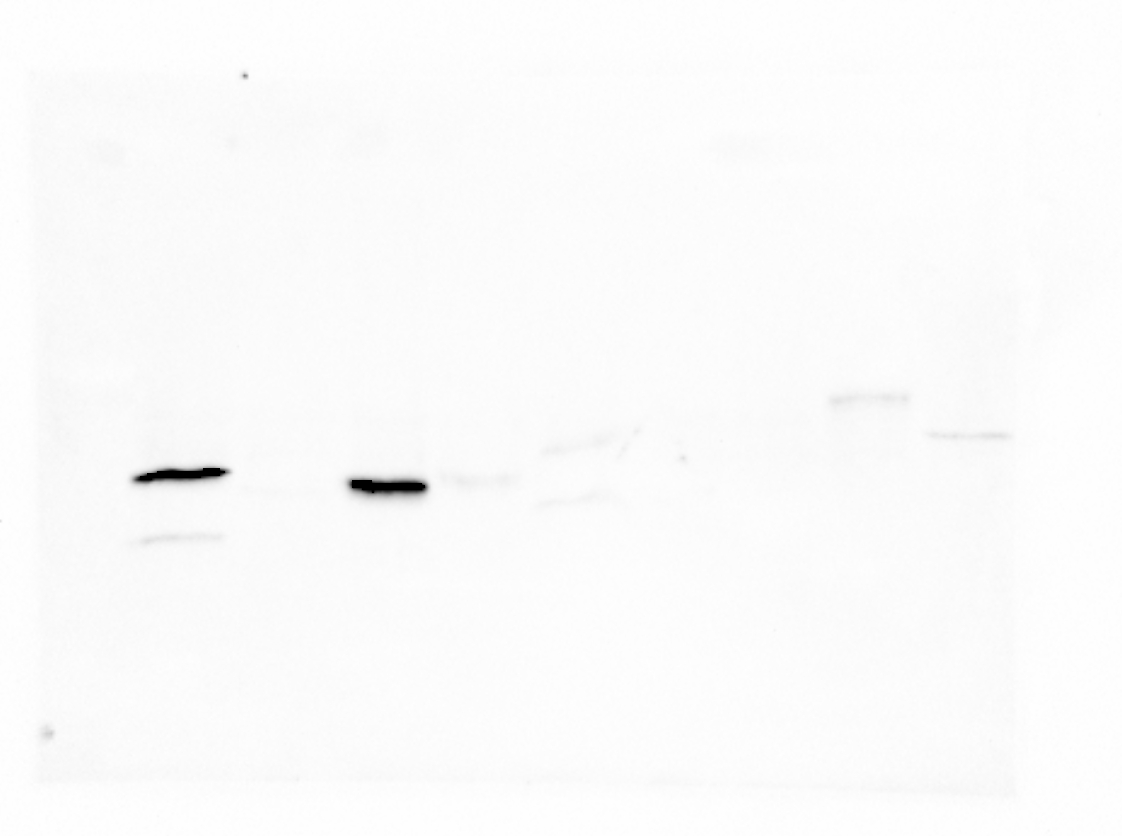

Supplement: Supplementary file 10 — Appendix Figures Source Data [file 44319_2024_203_MOESM10_ESM.zip › Appendix2_BRAF/thirdrow/rightmost/Pulldown.jpg]

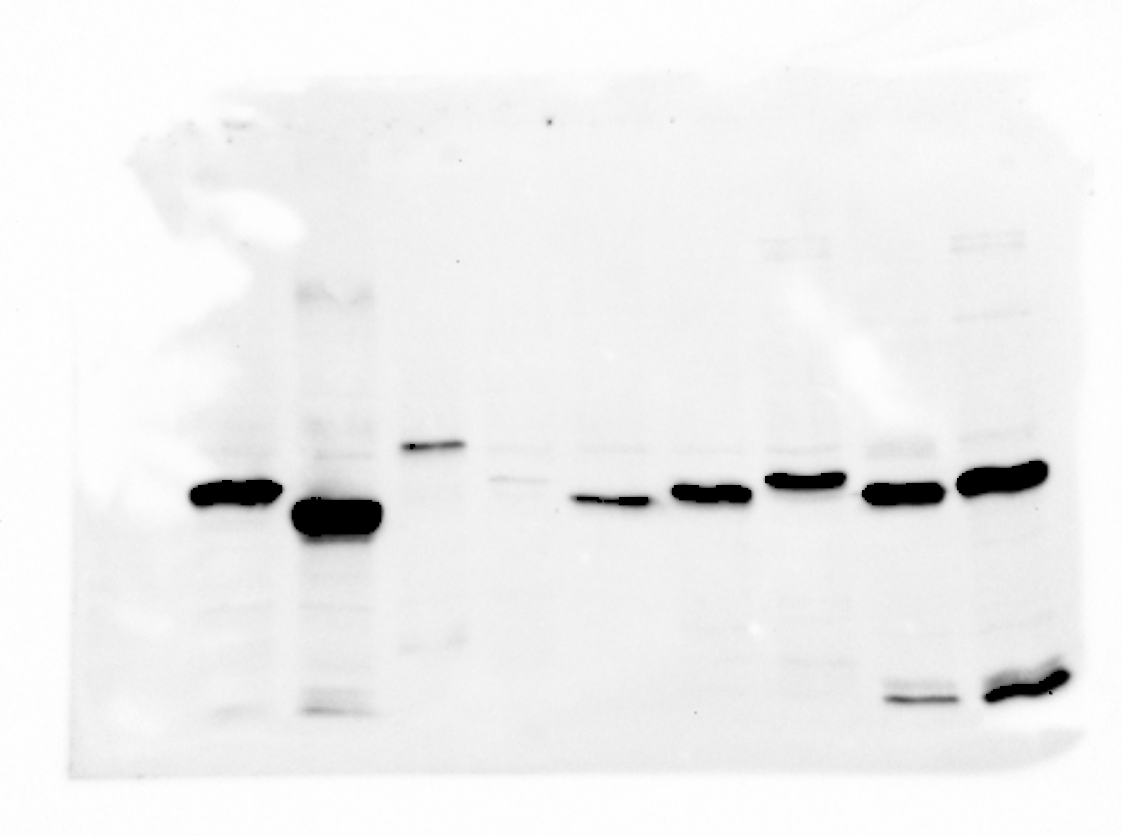

Supplement: Supplementary file 10 — Appendix Figures Source Data [file 44319_2024_203_MOESM10_ESM.zip › Appendix2_BRAF/toprow/leftmost/lysate.jpg]

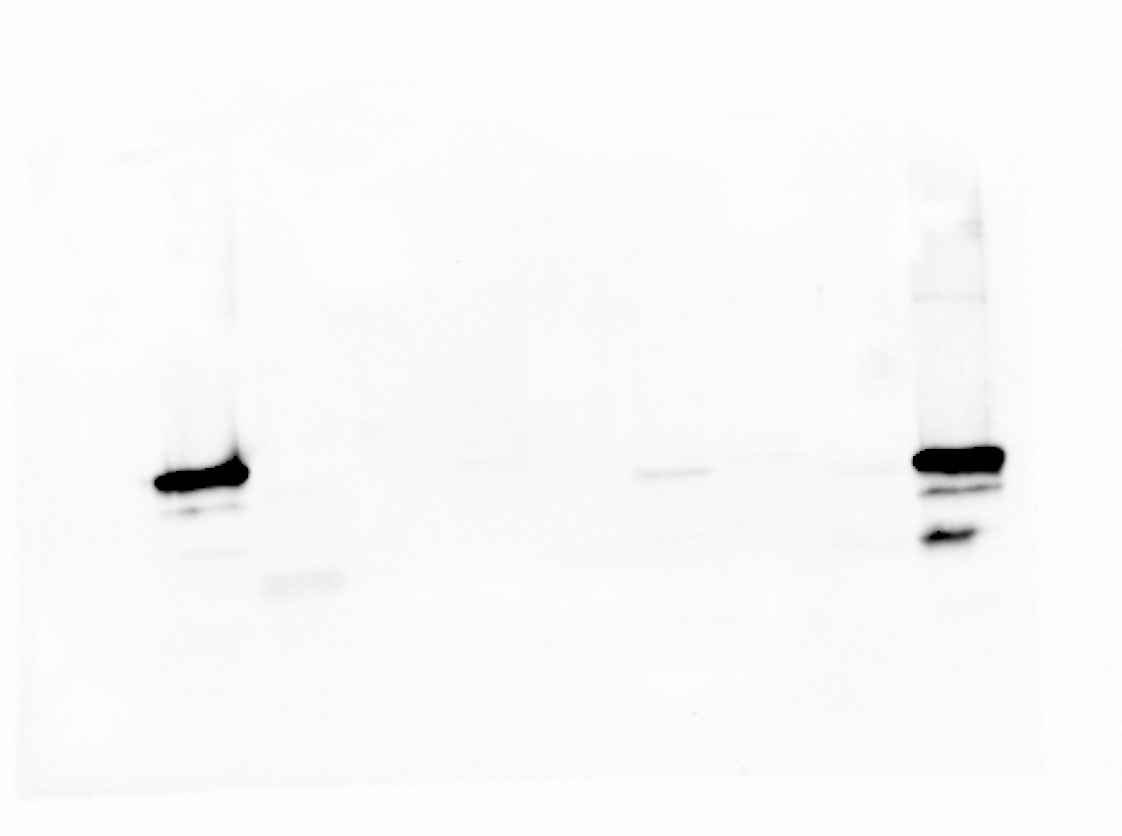

Supplement: Supplementary file 10 — Appendix Figures Source Data [file 44319_2024_203_MOESM10_ESM.zip › Appendix2_BRAF/toprow/leftmost/pulldown.jpg]

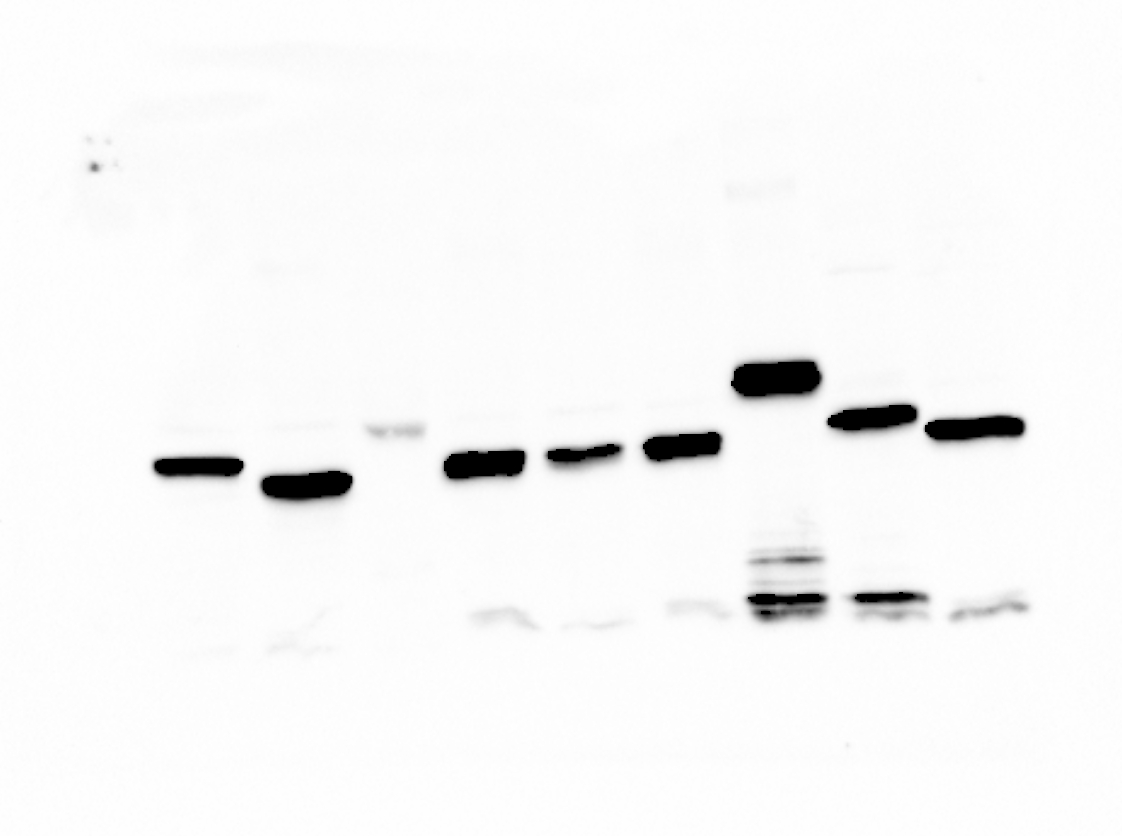

Supplement: Supplementary file 10 — Appendix Figures Source Data [file 44319_2024_203_MOESM10_ESM.zip › Appendix2_BRAF/toprow/middle/Lysate.jpg]

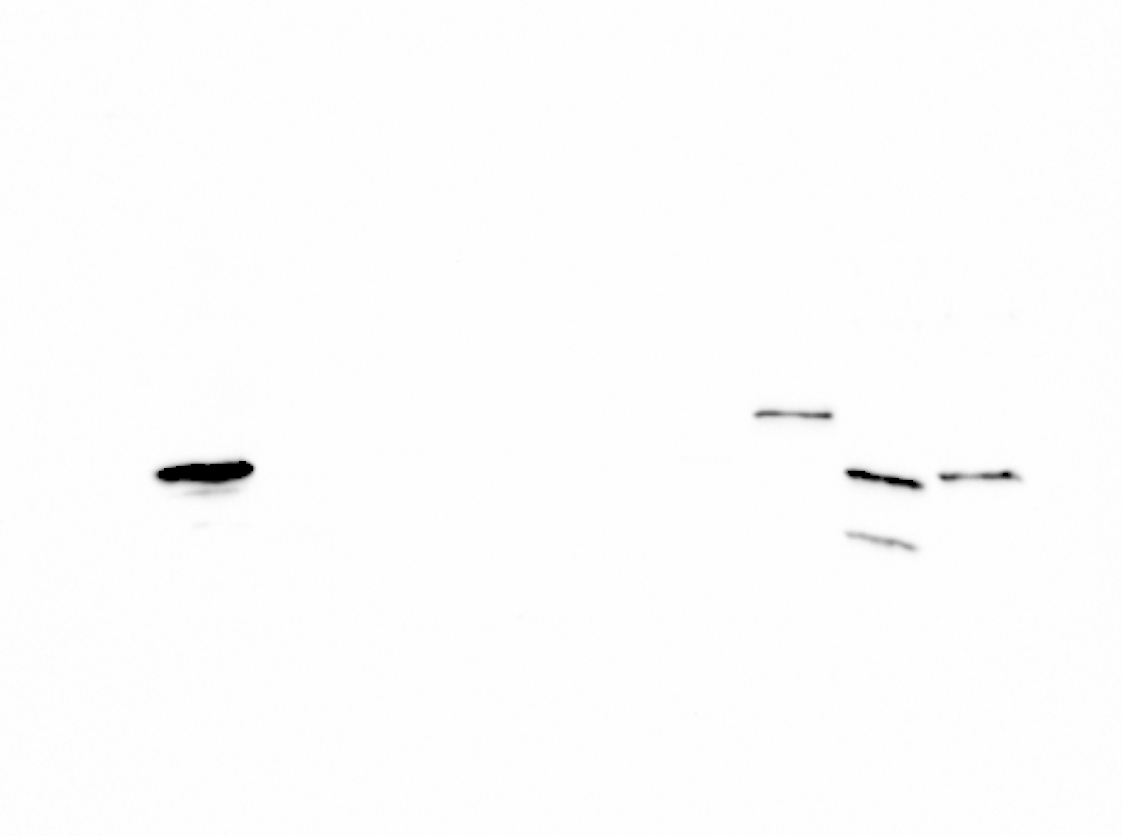

Supplement: Supplementary file 10 — Appendix Figures Source Data [file 44319_2024_203_MOESM10_ESM.zip › Appendix2_BRAF/toprow/middle/Pulldown.jpg]

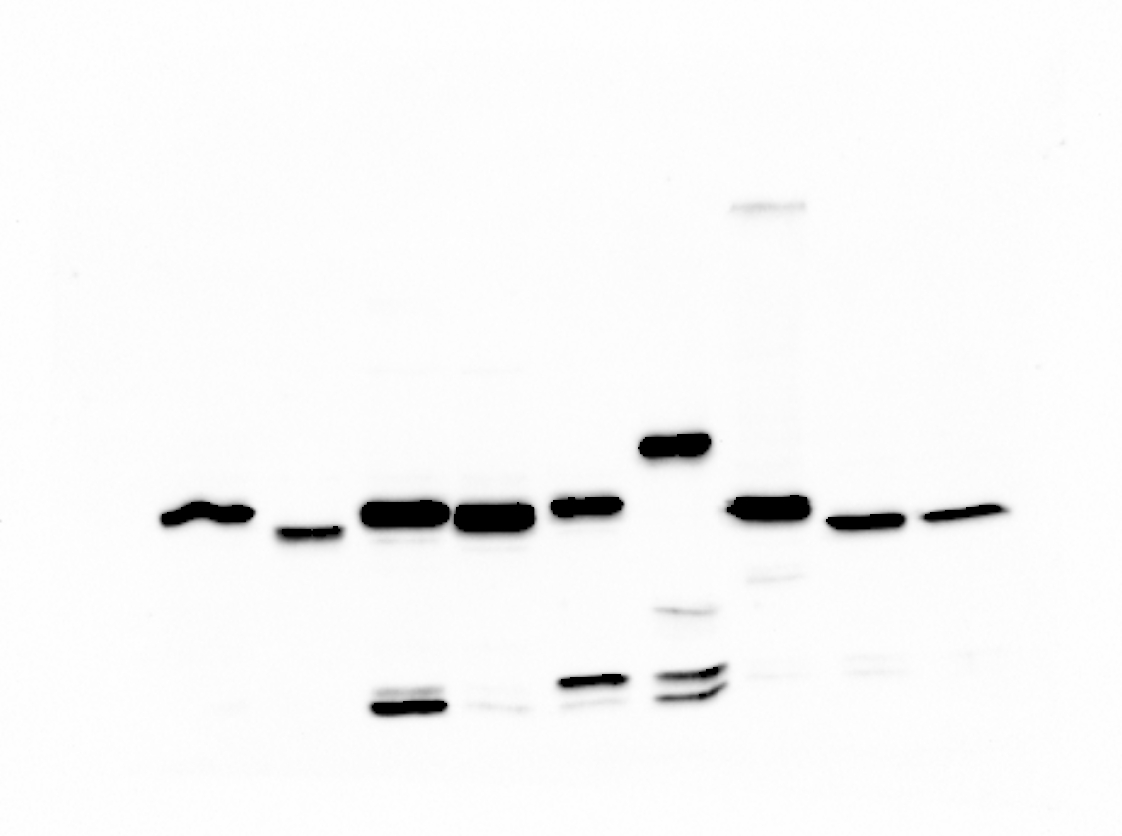

Supplement: Supplementary file 10 — Appendix Figures Source Data [file 44319_2024_203_MOESM10_ESM.zip › Appendix2_BRAF/toprow/rightmost/Lysate.jpg]

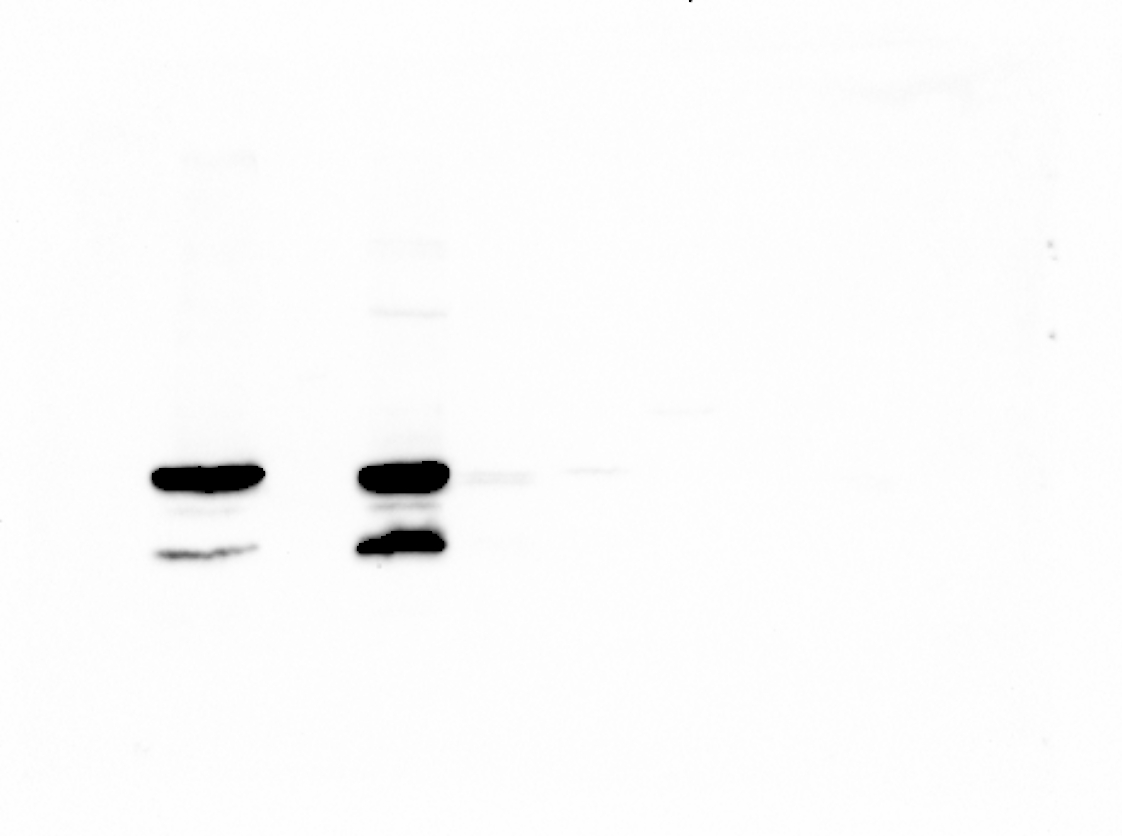

Supplement: Supplementary file 10 — Appendix Figures Source Data [file 44319_2024_203_MOESM10_ESM.zip › Appendix2_BRAF/toprow/rightmost/Pulldown.jpg]

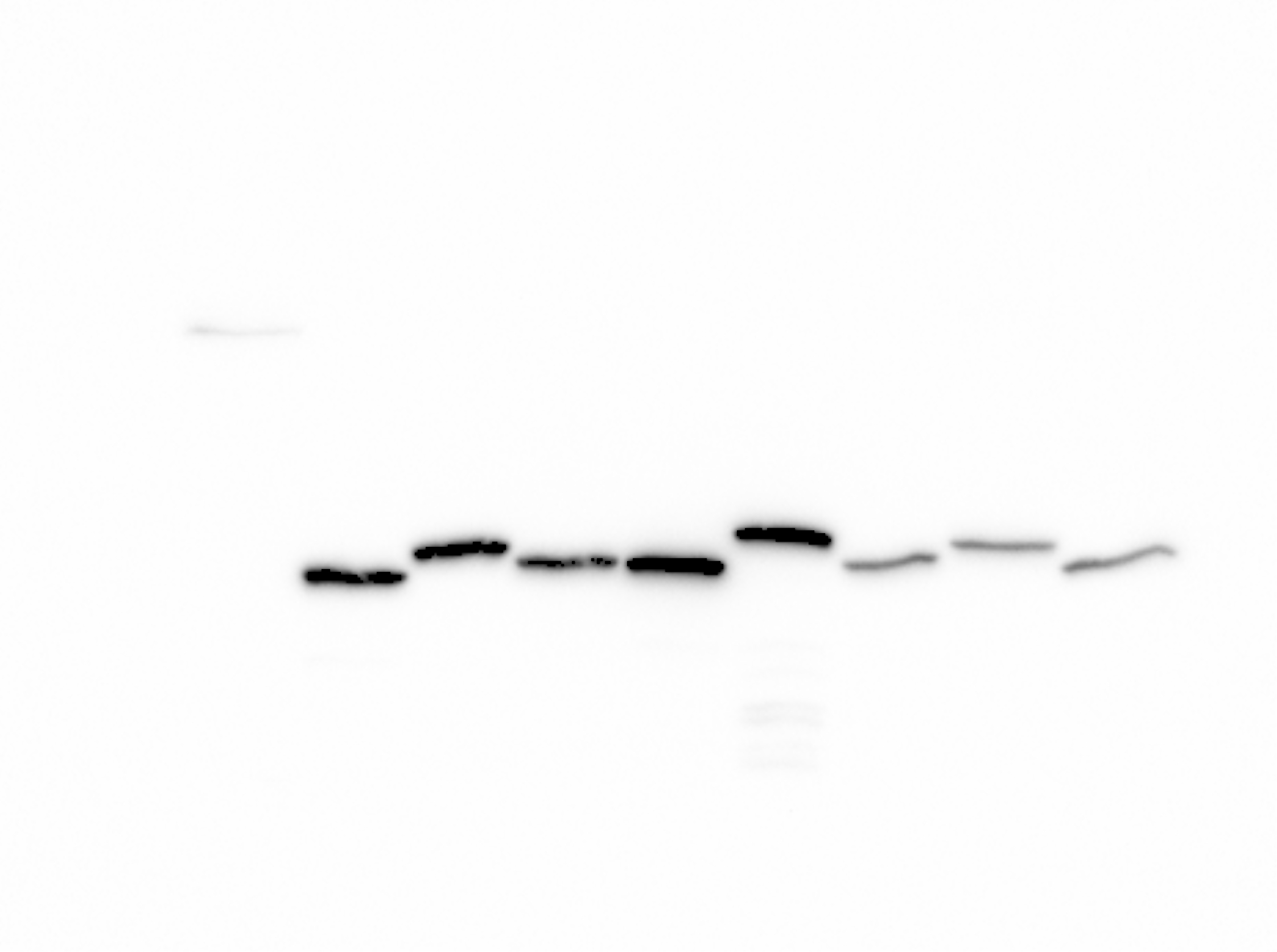

Supplement: Supplementary file 10 — Appendix Figures Source Data [file 44319_2024_203_MOESM10_ESM.zip › Appendix3_RASSF5/Fifthrow/Lysate.jpg]

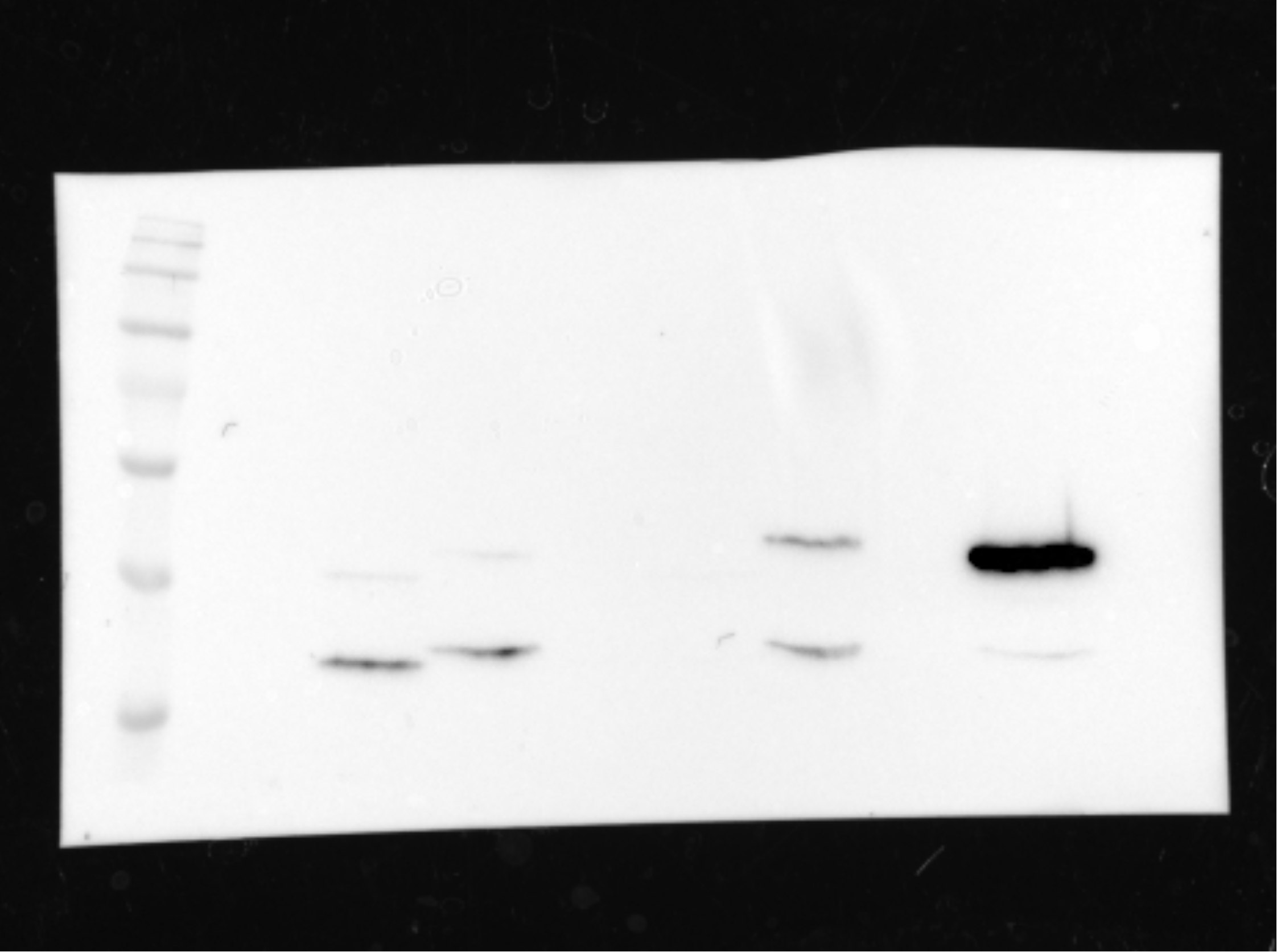

Supplement: Supplementary file 10 — Appendix Figures Source Data [file 44319_2024_203_MOESM10_ESM.zip › Appendix3_RASSF5/Fifthrow/Pulldown.jpg]

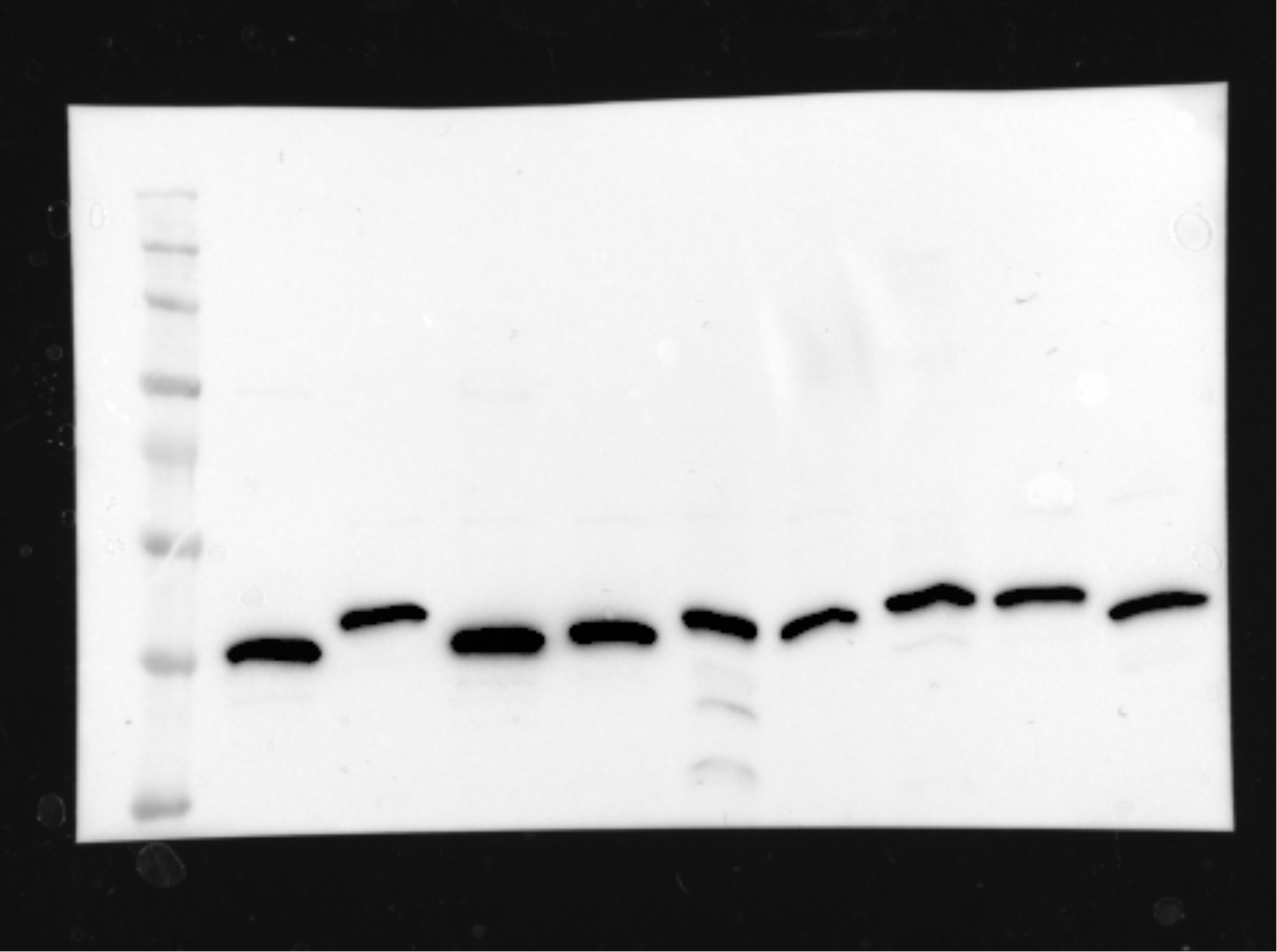

Supplement: Supplementary file 10 — Appendix Figures Source Data [file 44319_2024_203_MOESM10_ESM.zip › Appendix3_RASSF5/Fourthrow/Leftmost/Lysate.jpg]

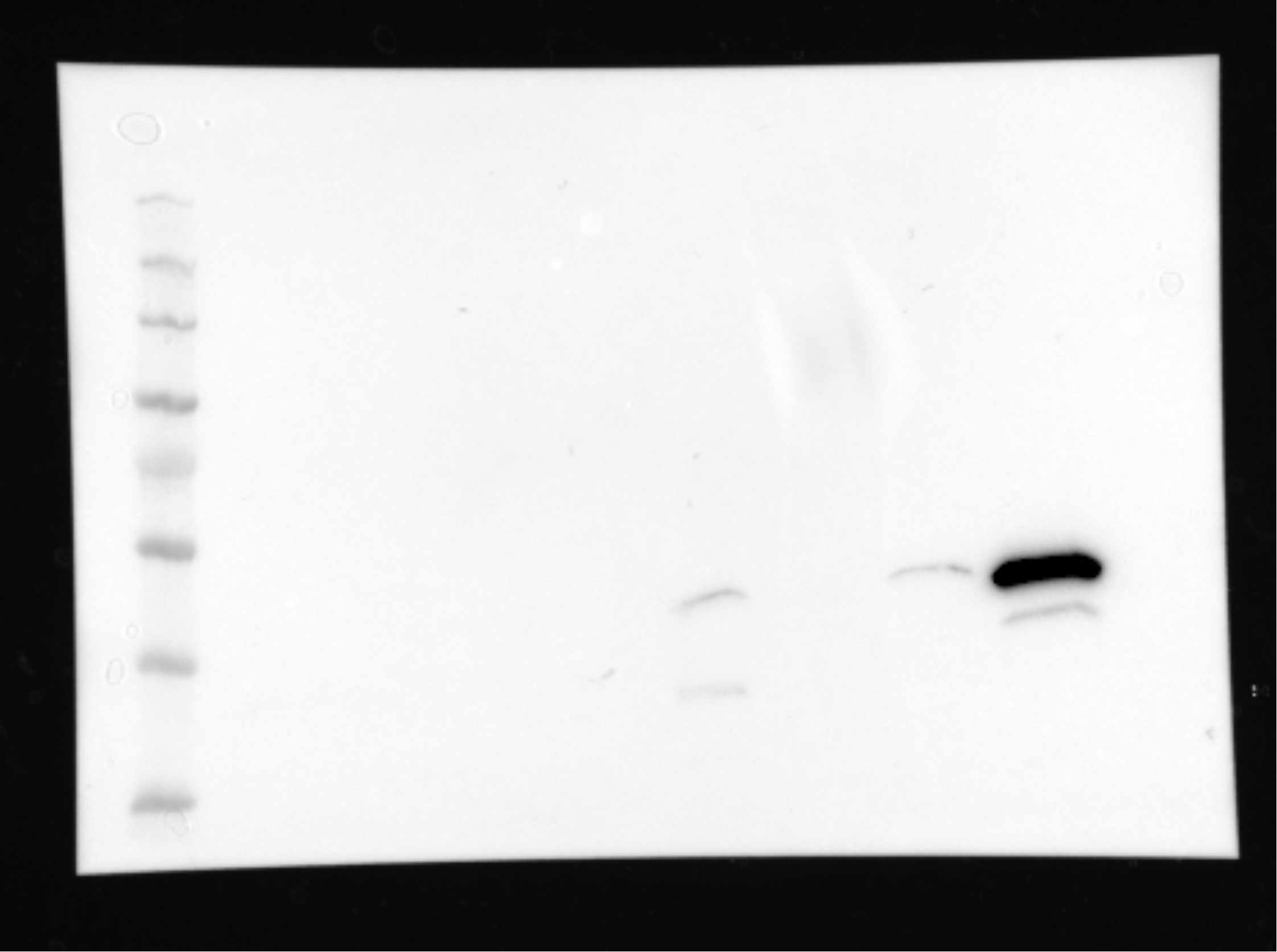

Supplement: Supplementary file 10 — Appendix Figures Source Data [file 44319_2024_203_MOESM10_ESM.zip › Appendix3_RASSF5/Fourthrow/Leftmost/Pulldown.jpg]

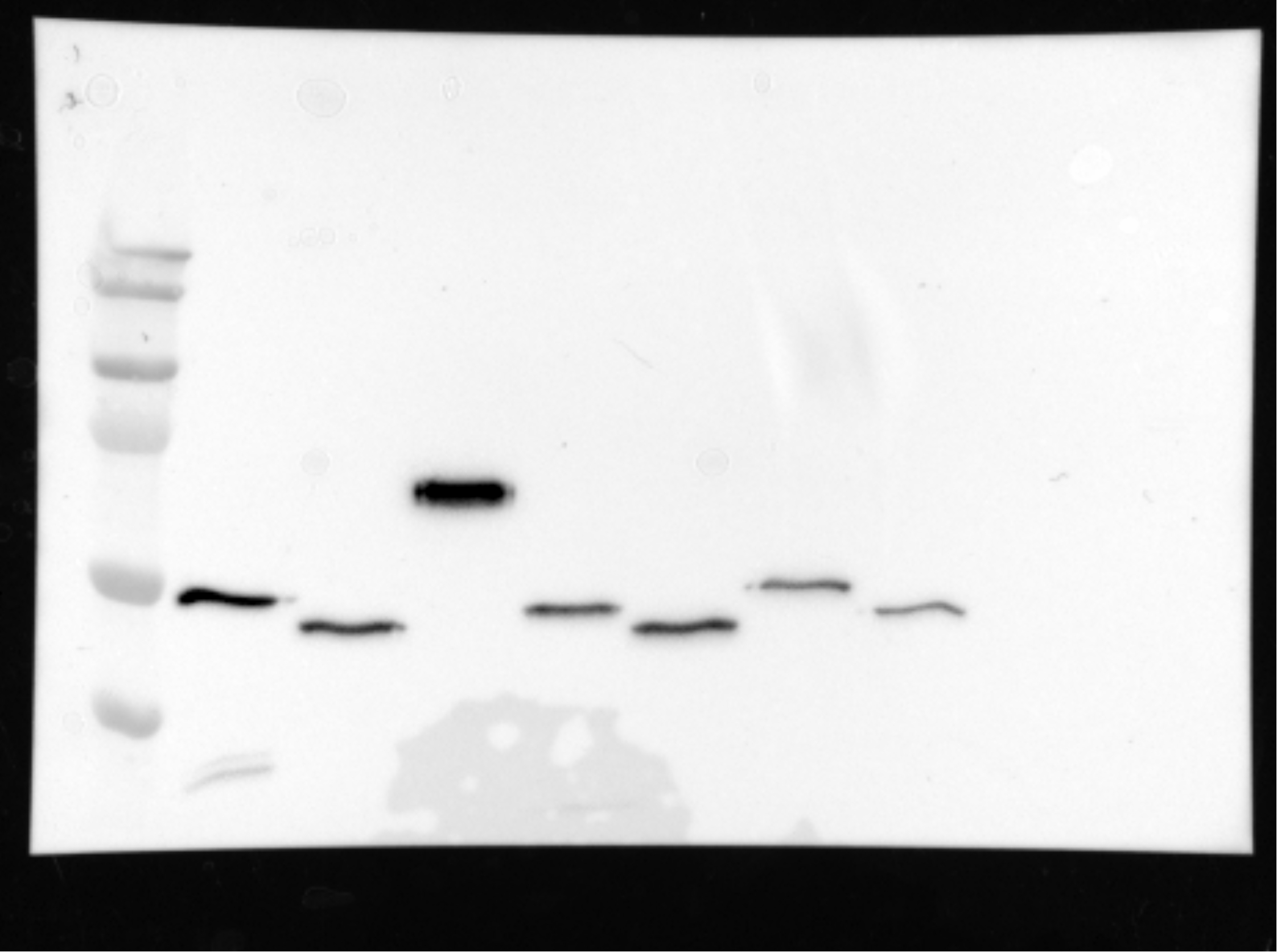

Supplement: Supplementary file 10 — Appendix Figures Source Data [file 44319_2024_203_MOESM10_ESM.zip › Appendix3_RASSF5/Fourthrow/Middle/Lysate.jpg]

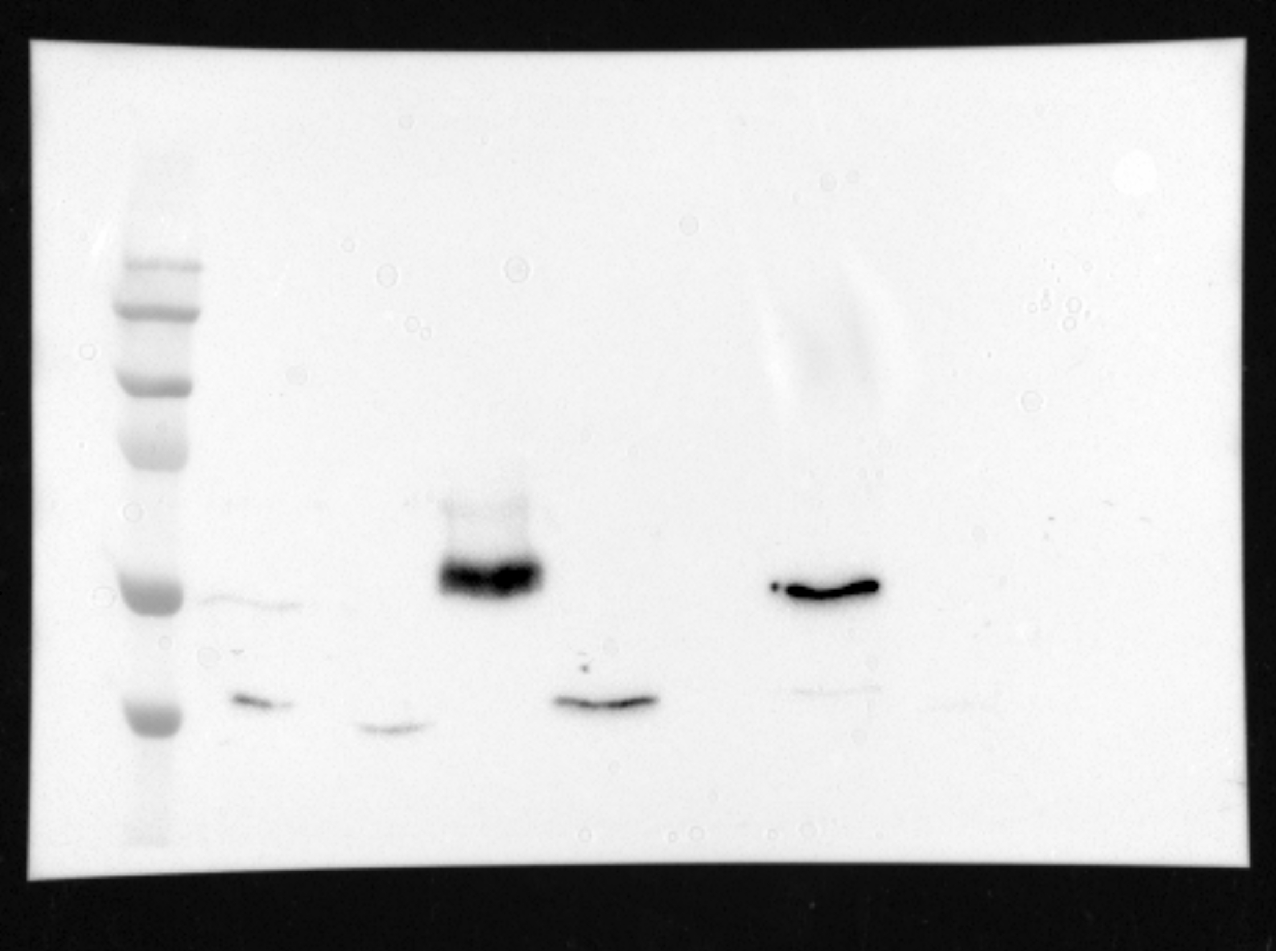

Supplement: Supplementary file 10 — Appendix Figures Source Data [file 44319_2024_203_MOESM10_ESM.zip › Appendix3_RASSF5/Fourthrow/Middle/Pulldown.jpg]

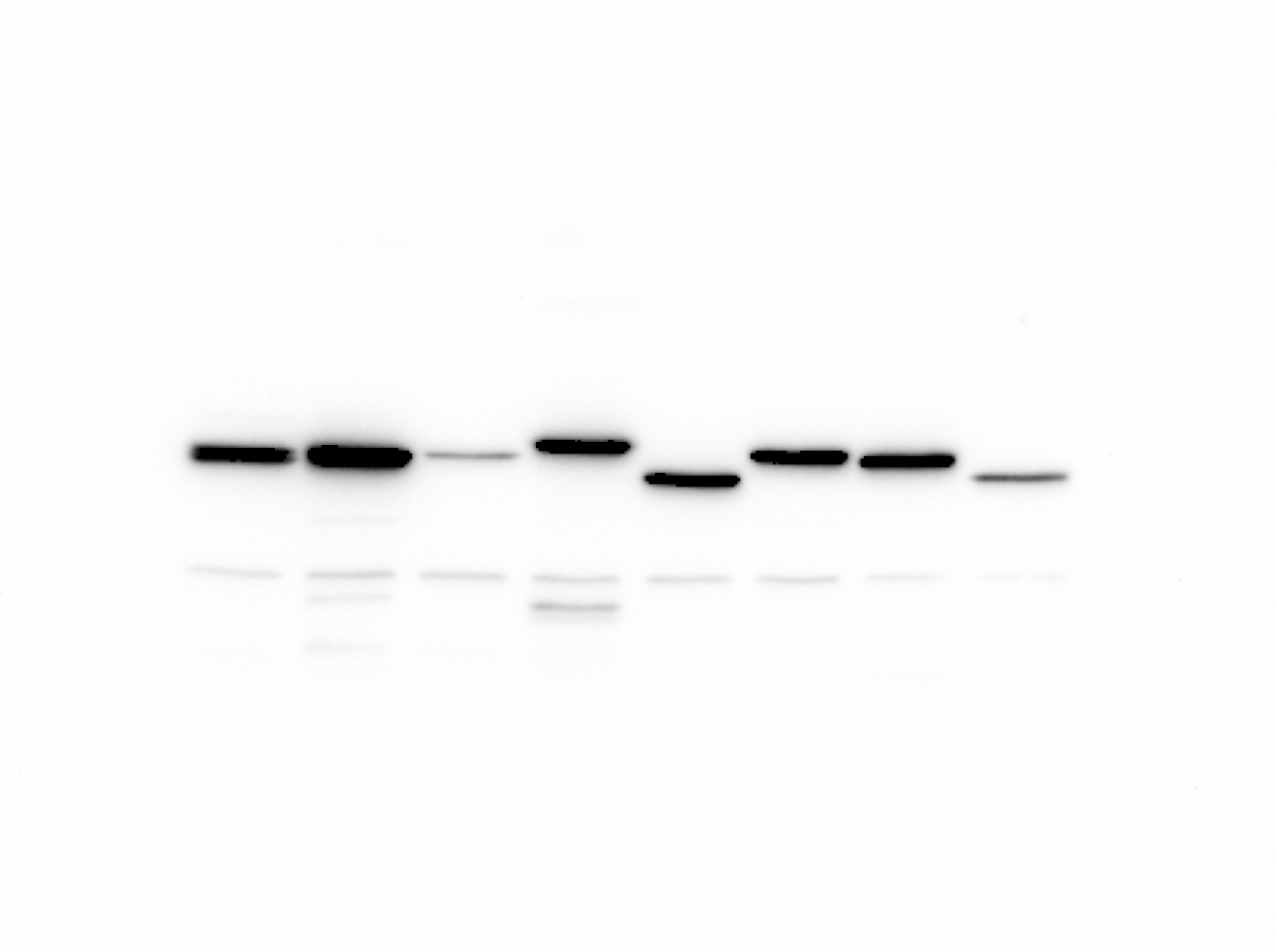

Supplement: Supplementary file 10 — Appendix Figures Source Data [file 44319_2024_203_MOESM10_ESM.zip › Appendix3_RASSF5/Fourthrow/Rightmost/Lysate.jpg]

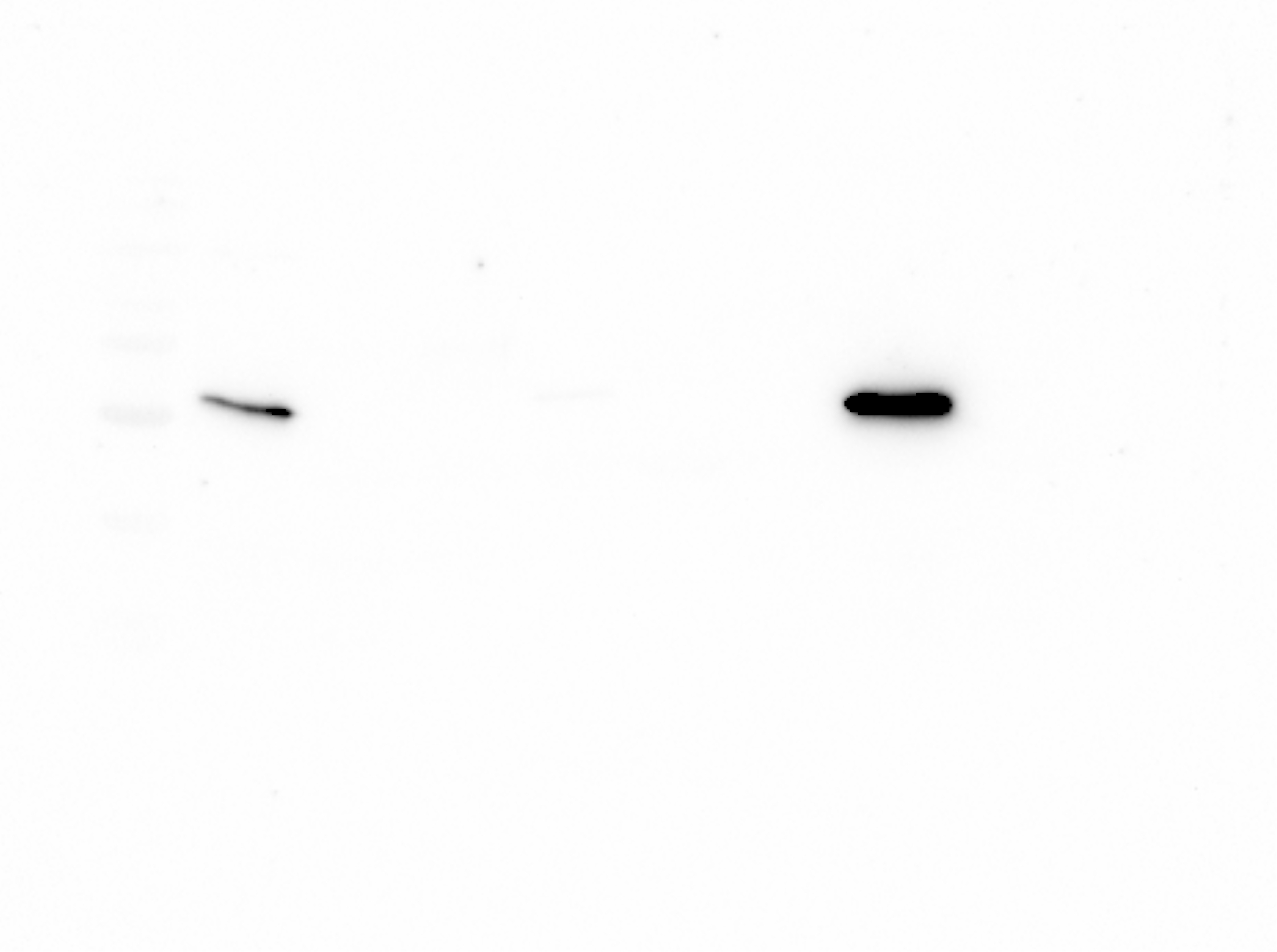

Supplement: Supplementary file 10 — Appendix Figures Source Data [file 44319_2024_203_MOESM10_ESM.zip › Appendix3_RASSF5/Fourthrow/Rightmost/Pulldown.jpg]

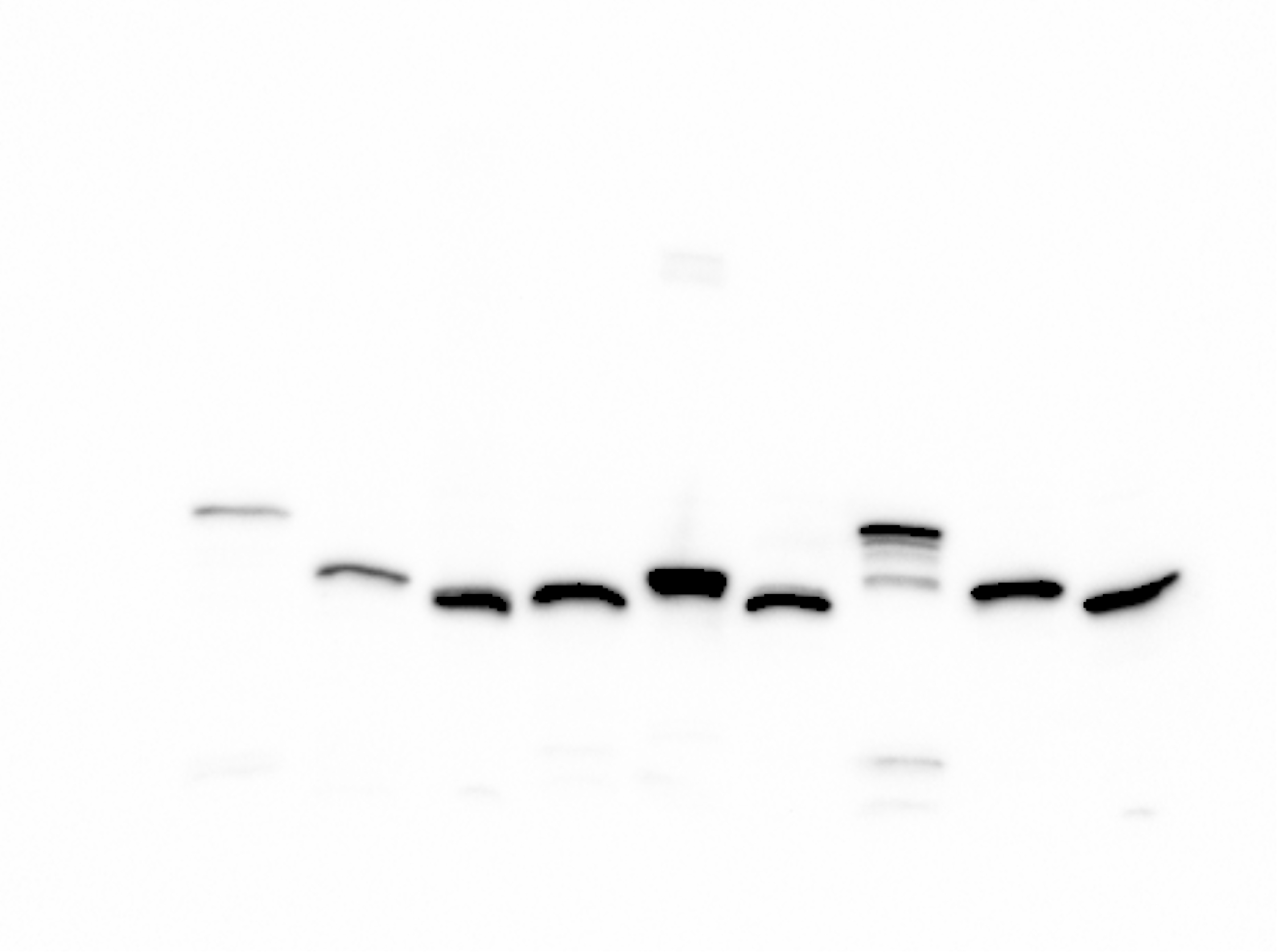

Supplement: Supplementary file 10 — Appendix Figures Source Data [file 44319_2024_203_MOESM10_ESM.zip › Appendix3_RASSF5/Secondrow/Leftmost/Lysate.jpg]

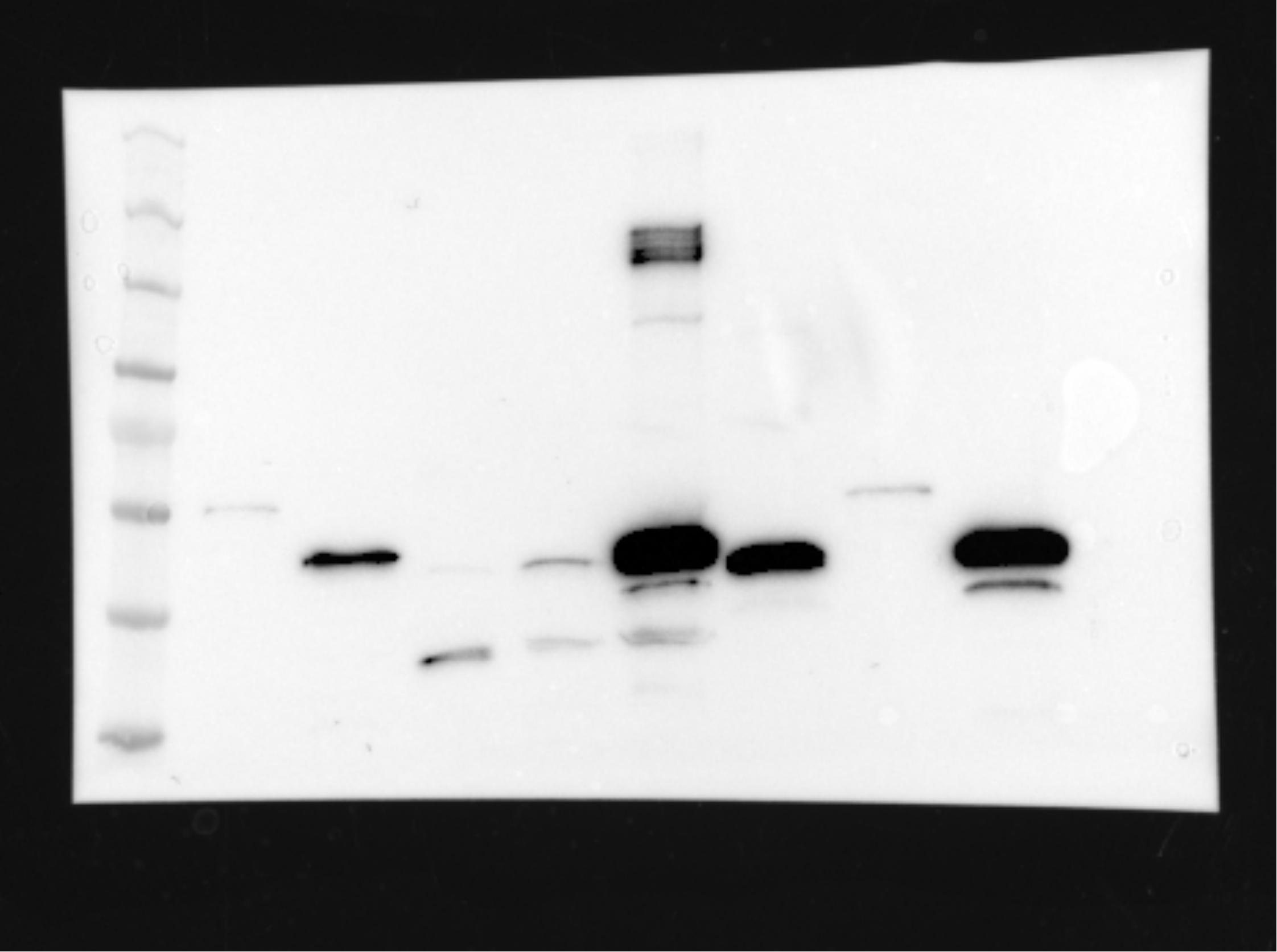

Supplement: Supplementary file 10 — Appendix Figures Source Data [file 44319_2024_203_MOESM10_ESM.zip › Appendix3_RASSF5/Secondrow/Leftmost/Pulldown.jpg]

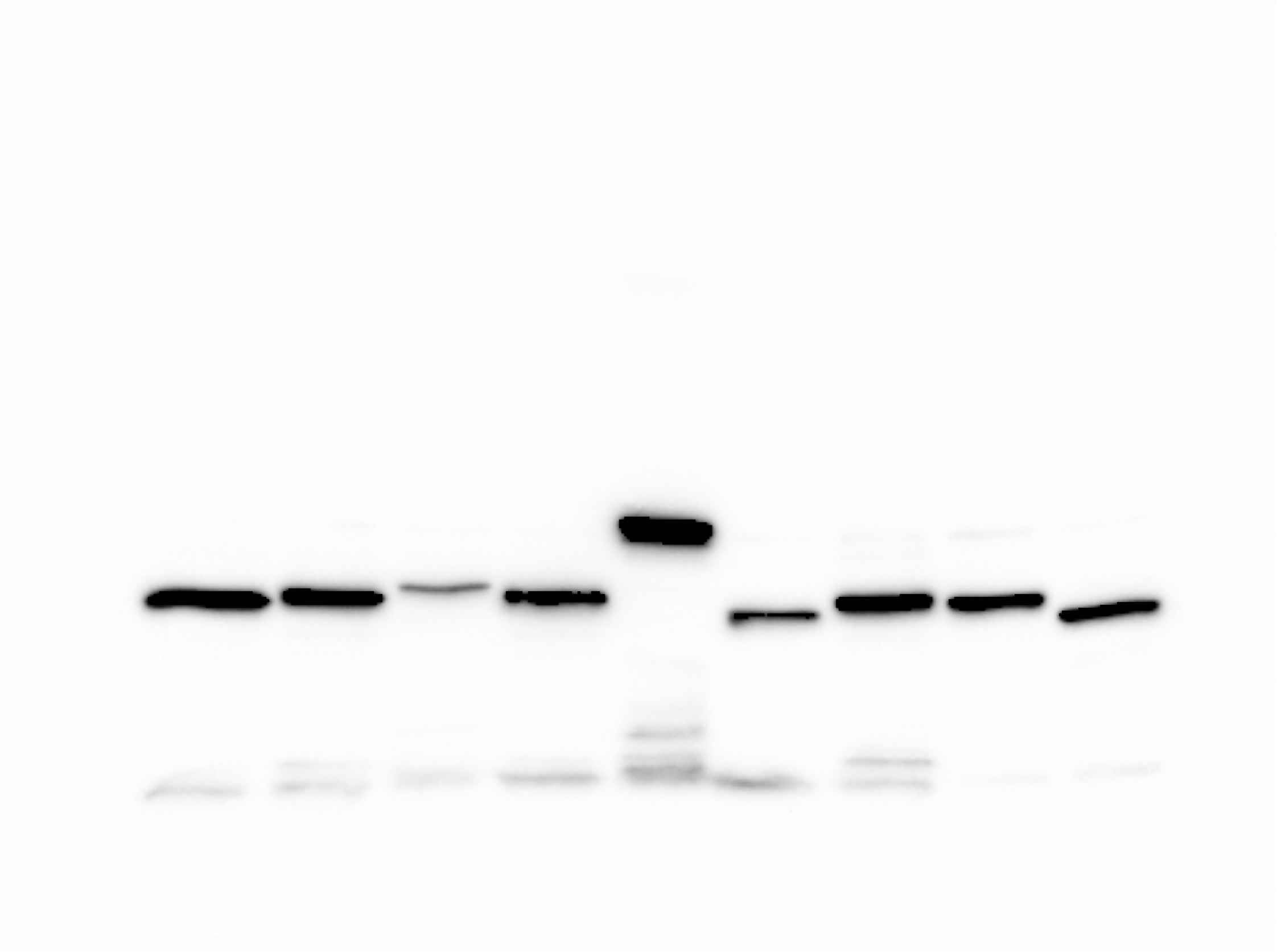

Supplement: Supplementary file 10 — Appendix Figures Source Data [file 44319_2024_203_MOESM10_ESM.zip › Appendix3_RASSF5/Secondrow/Middle/Lysate.jpg]

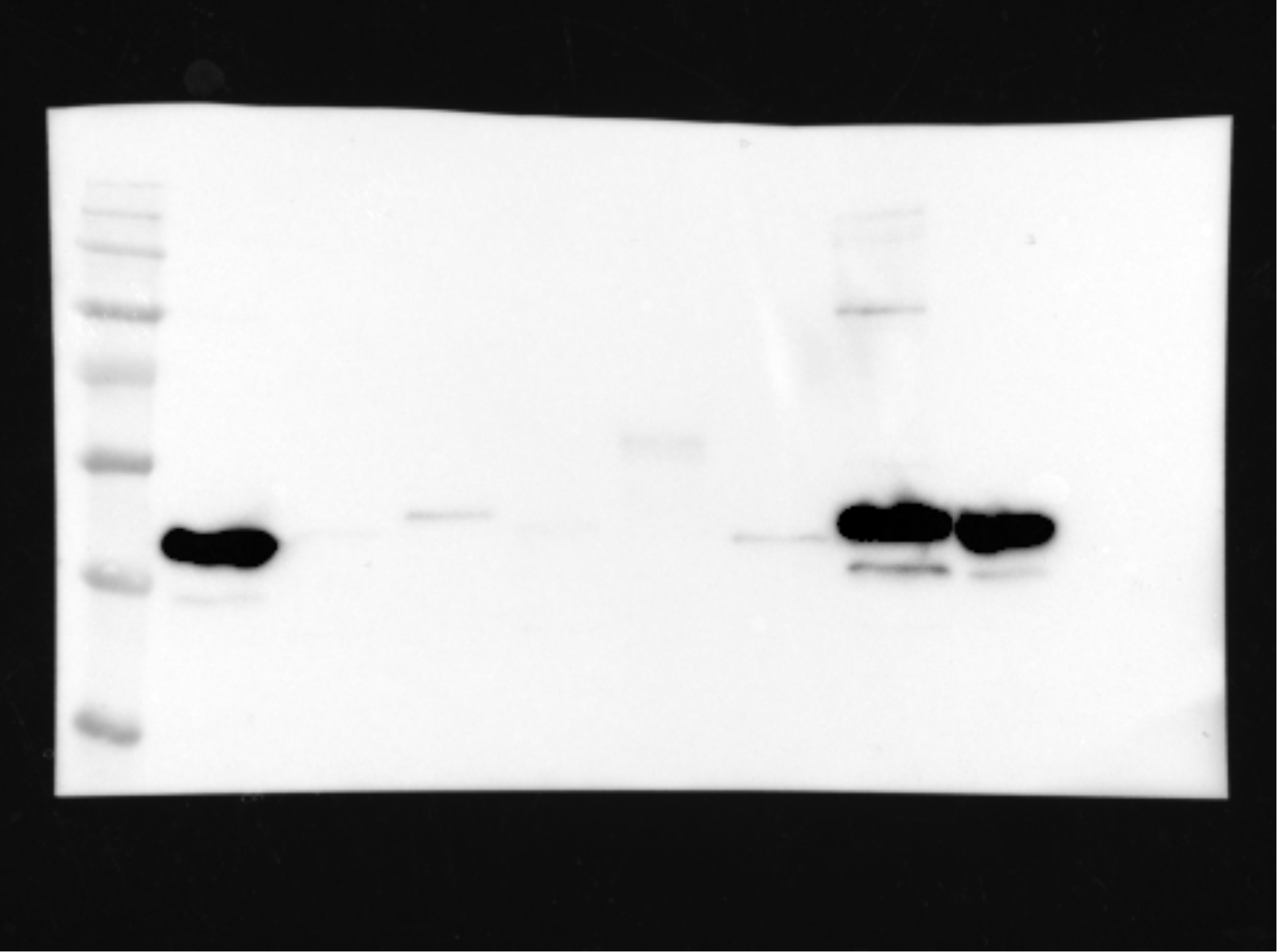

Supplement: Supplementary file 10 — Appendix Figures Source Data [file 44319_2024_203_MOESM10_ESM.zip › Appendix3_RASSF5/Secondrow/Middle/Pulldown.jpg]

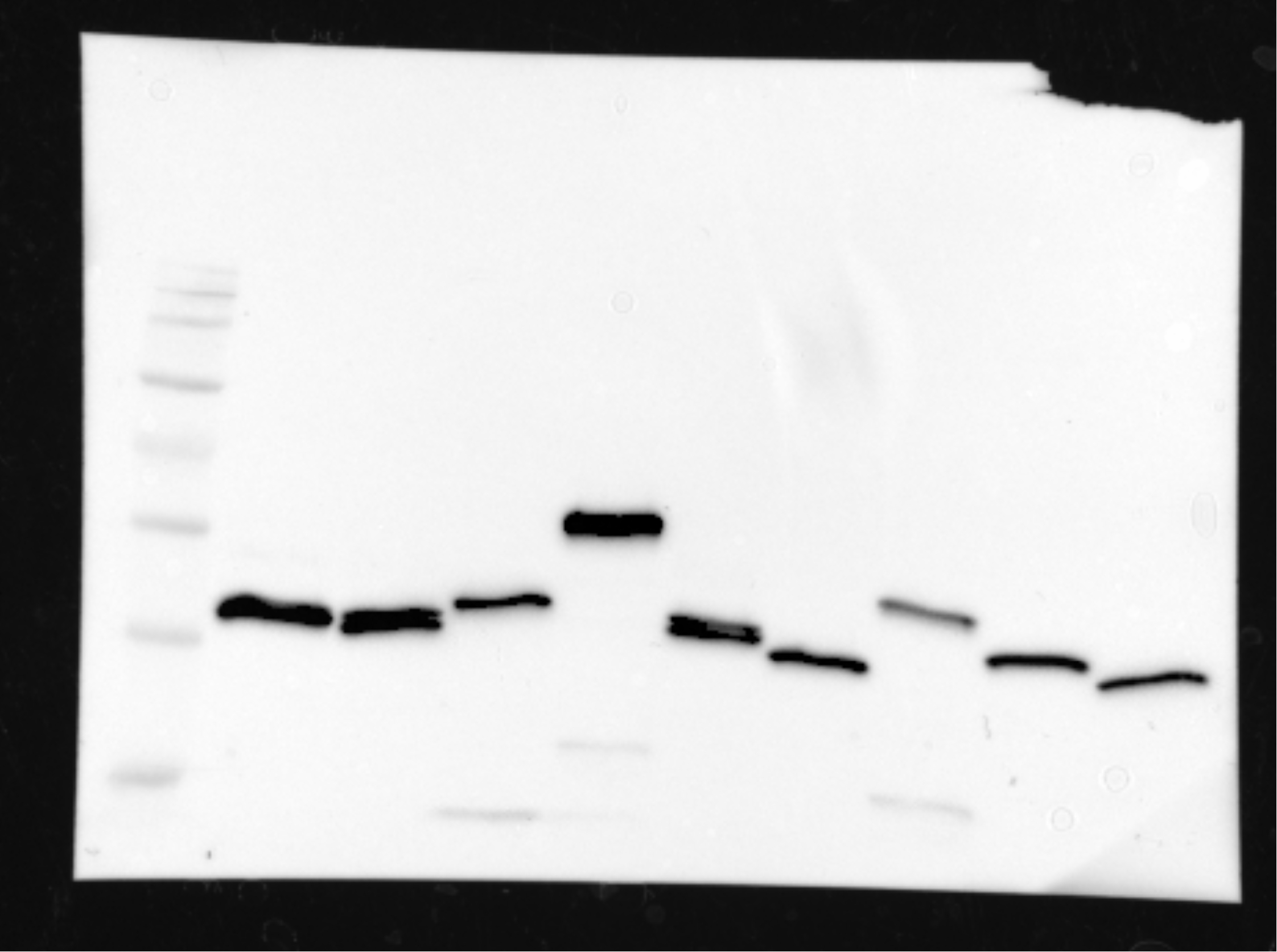

Supplement: Supplementary file 10 — Appendix Figures Source Data [file 44319_2024_203_MOESM10_ESM.zip › Appendix3_RASSF5/Secondrow/Rightmost/Lysate.jpg]

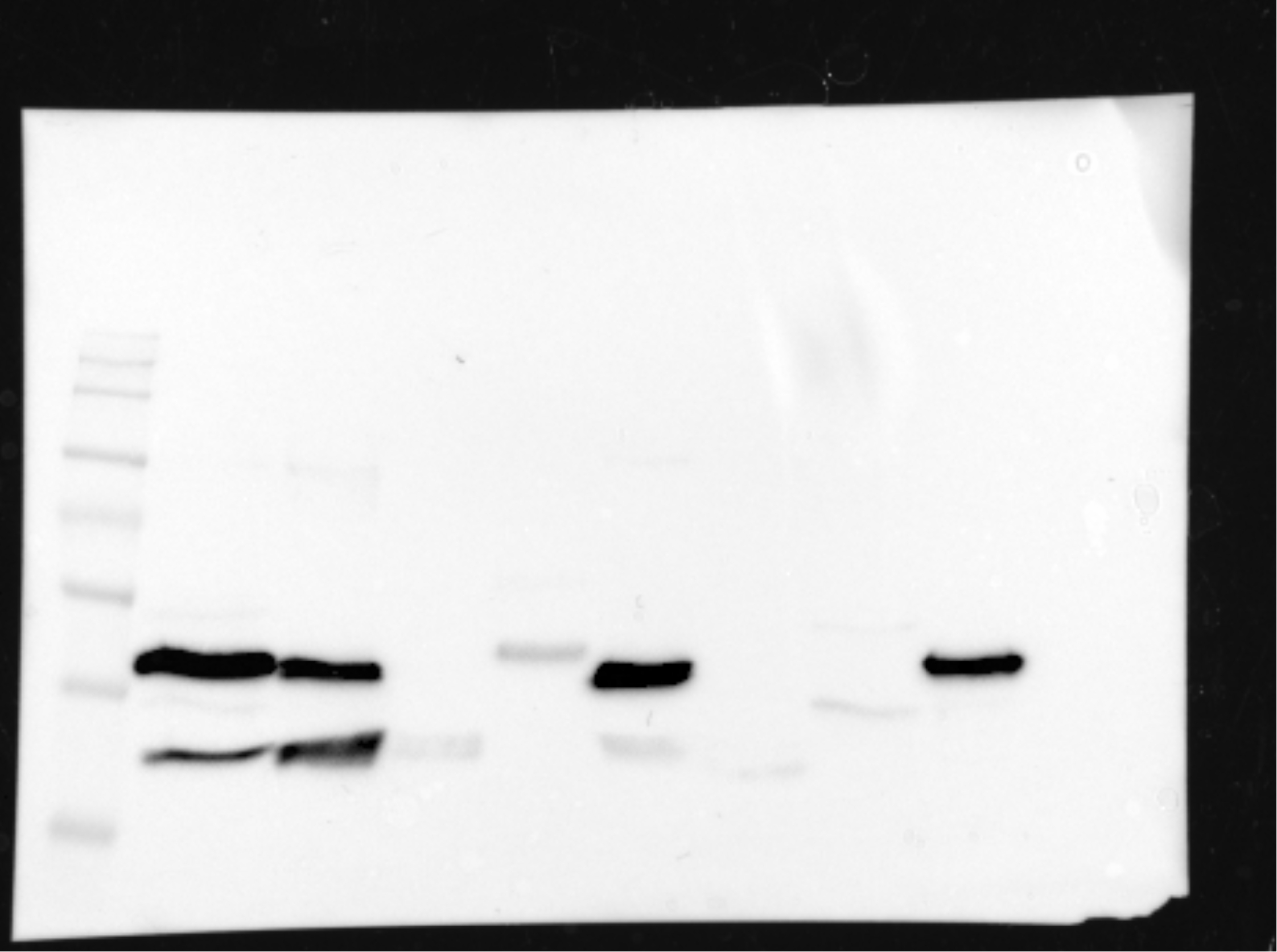

Supplement: Supplementary file 10 — Appendix Figures Source Data [file 44319_2024_203_MOESM10_ESM.zip › Appendix3_RASSF5/Secondrow/Rightmost/Pulldown.jpg]

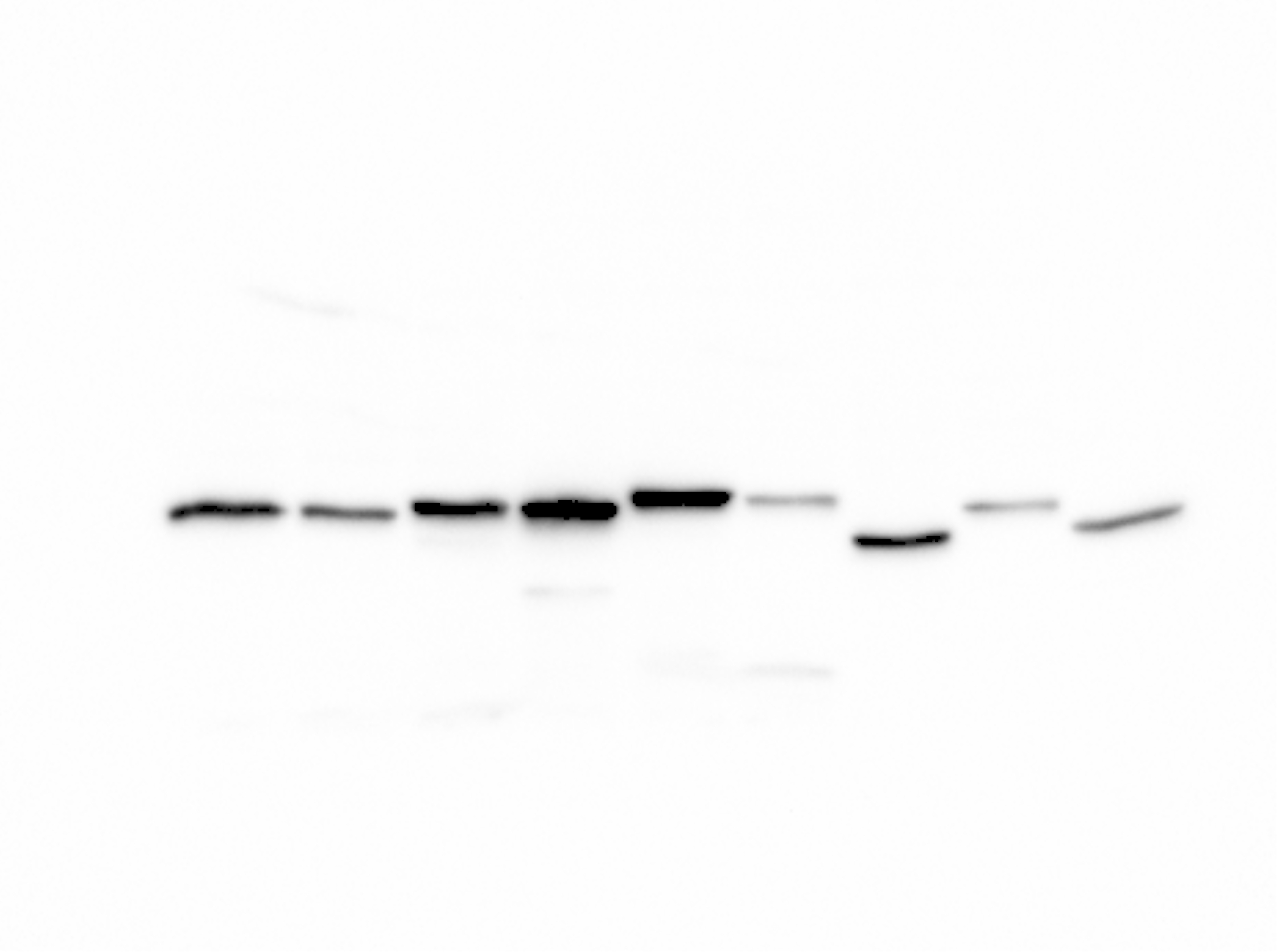

Supplement: Supplementary file 10 — Appendix Figures Source Data [file 44319_2024_203_MOESM10_ESM.zip › Appendix3_RASSF5/Thirdrow/Leftmost/Lysate.jpg]

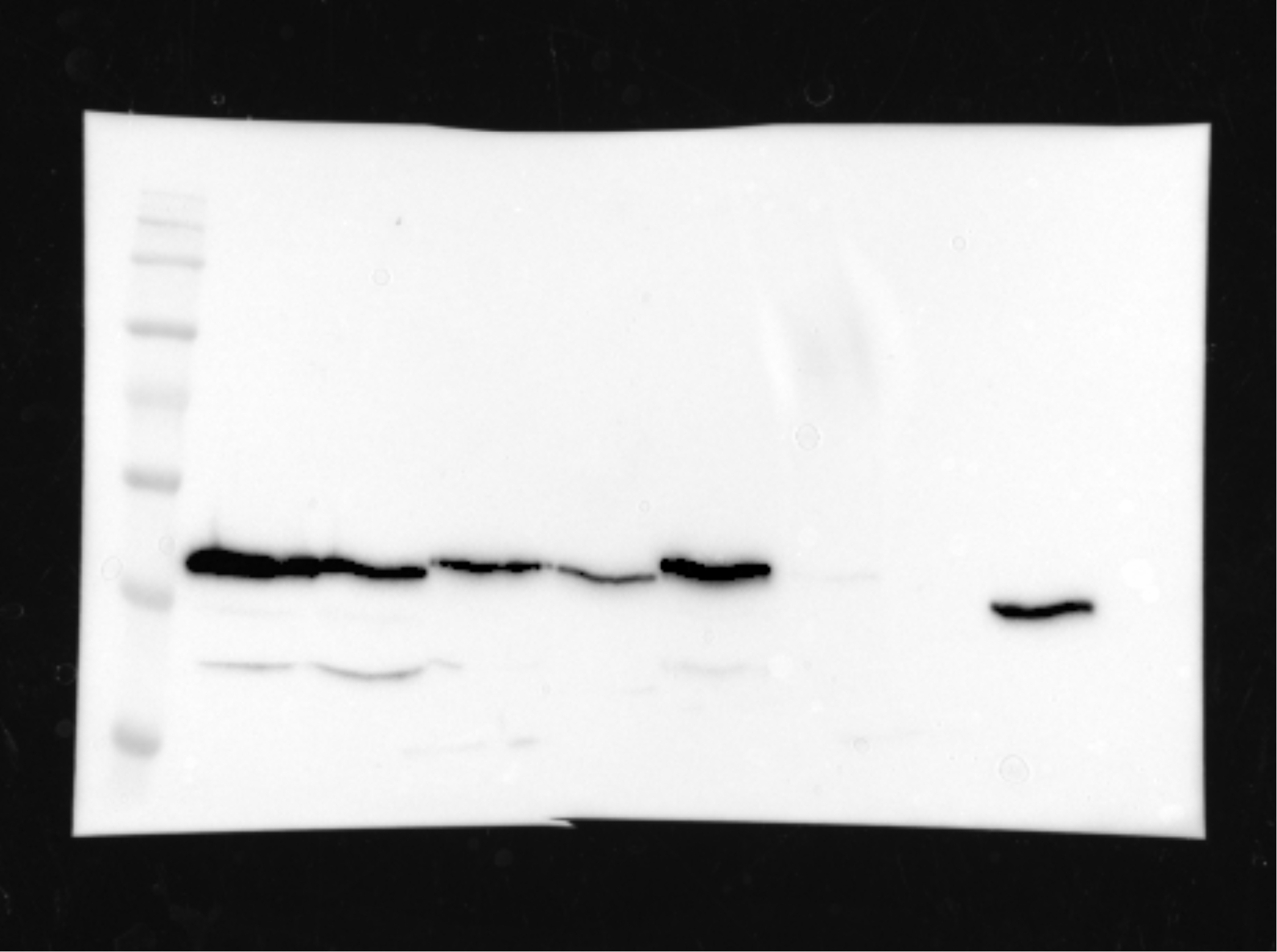

Supplement: Supplementary file 10 — Appendix Figures Source Data [file 44319_2024_203_MOESM10_ESM.zip › Appendix3_RASSF5/Thirdrow/Leftmost/Pulldown.jpg]

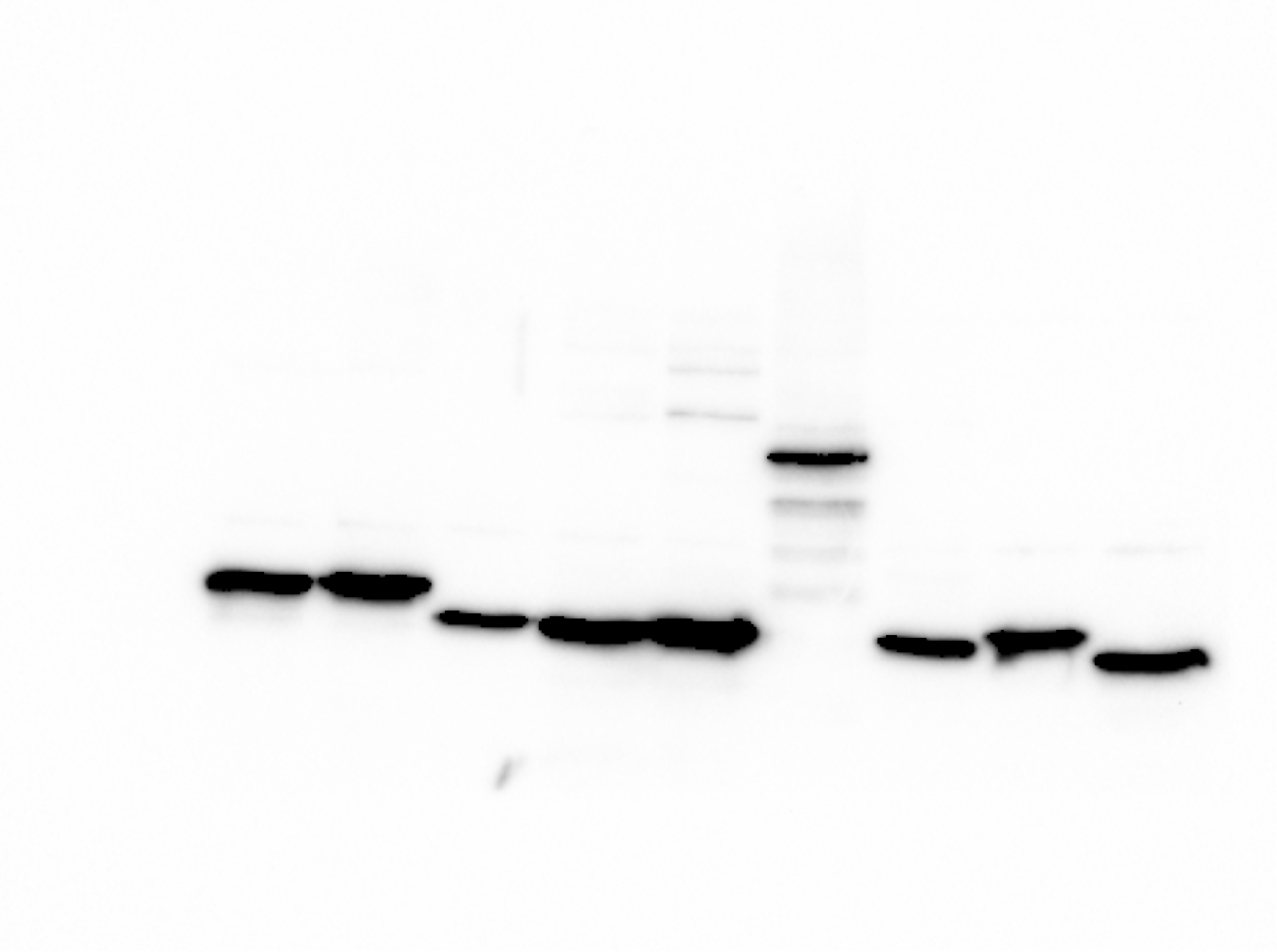

Supplement: Supplementary file 10 — Appendix Figures Source Data [file 44319_2024_203_MOESM10_ESM.zip › Appendix3_RASSF5/Thirdrow/Middle/Lysate.jpg]

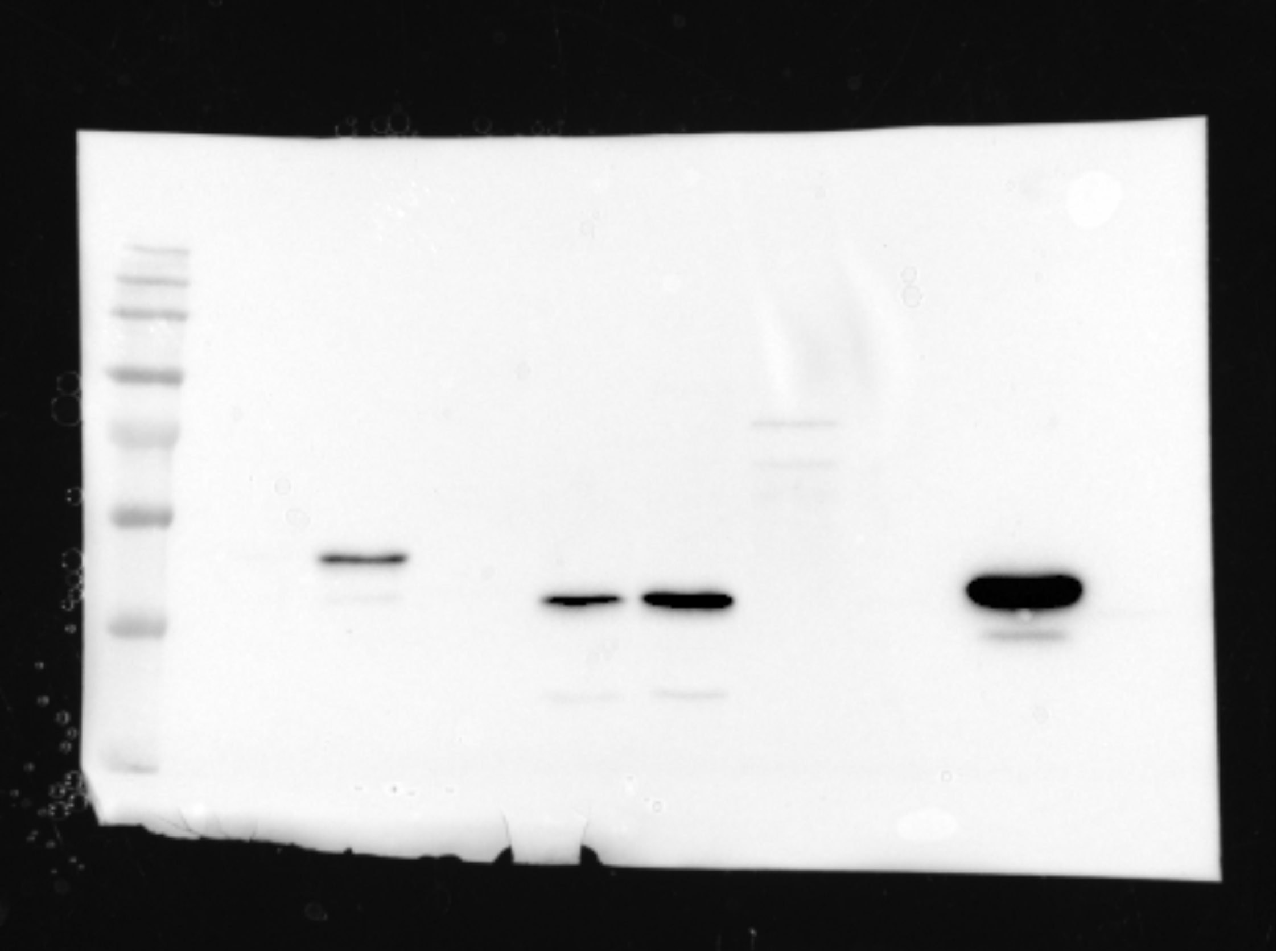

Supplement: Supplementary file 10 — Appendix Figures Source Data [file 44319_2024_203_MOESM10_ESM.zip › Appendix3_RASSF5/Thirdrow/Middle/Pulldown.jpg]

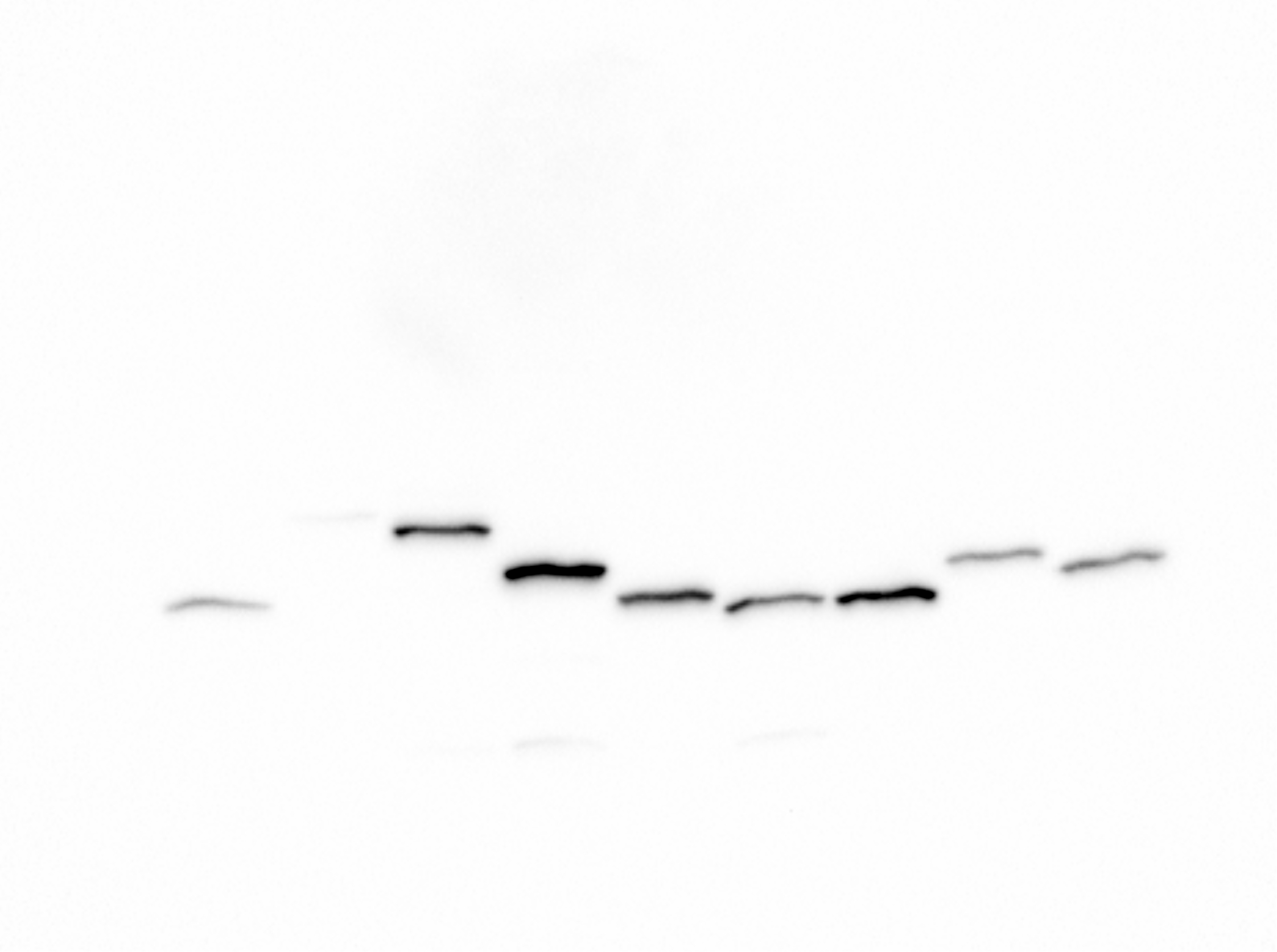

Supplement: Supplementary file 10 — Appendix Figures Source Data [file 44319_2024_203_MOESM10_ESM.zip › Appendix3_RASSF5/Thirdrow/Rightmost/Lysate.jpg]

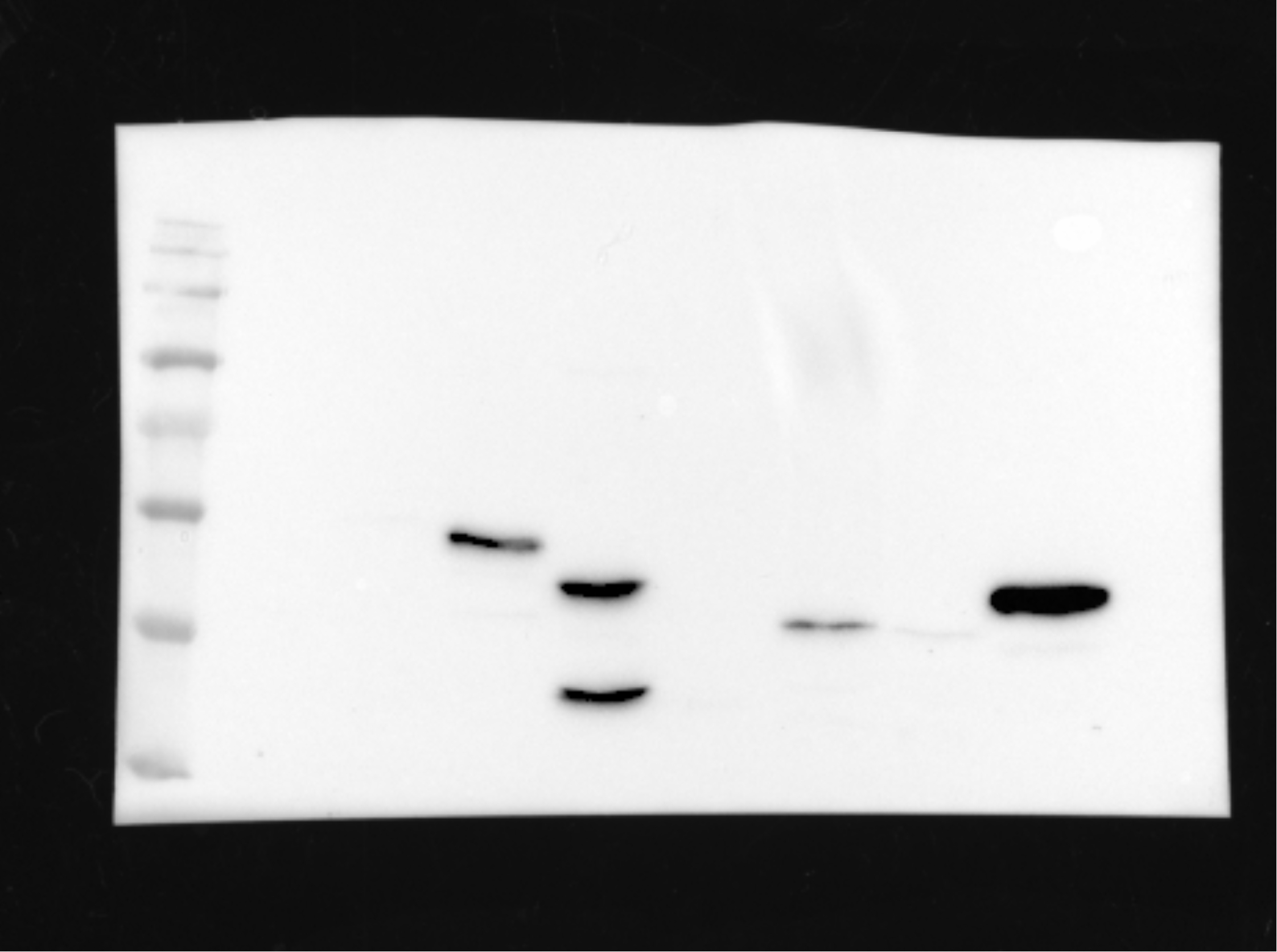

Supplement: Supplementary file 10 — Appendix Figures Source Data [file 44319_2024_203_MOESM10_ESM.zip › Appendix3_RASSF5/Thirdrow/Rightmost/Pulldown.jpg]

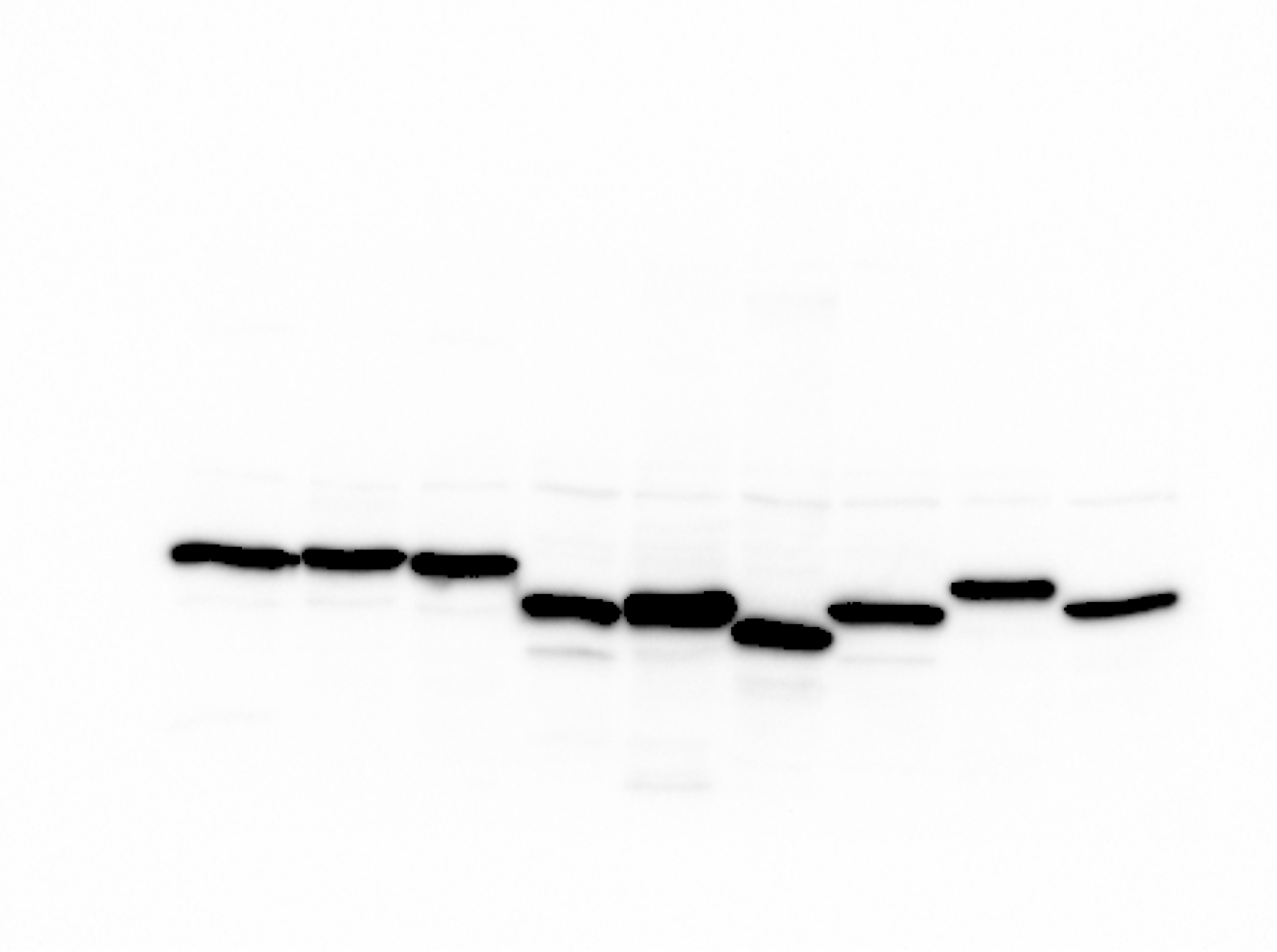

Supplement: Supplementary file 10 — Appendix Figures Source Data [file 44319_2024_203_MOESM10_ESM.zip › Appendix3_RASSF5/Toprow/Leftmost/Lysate.jpg]

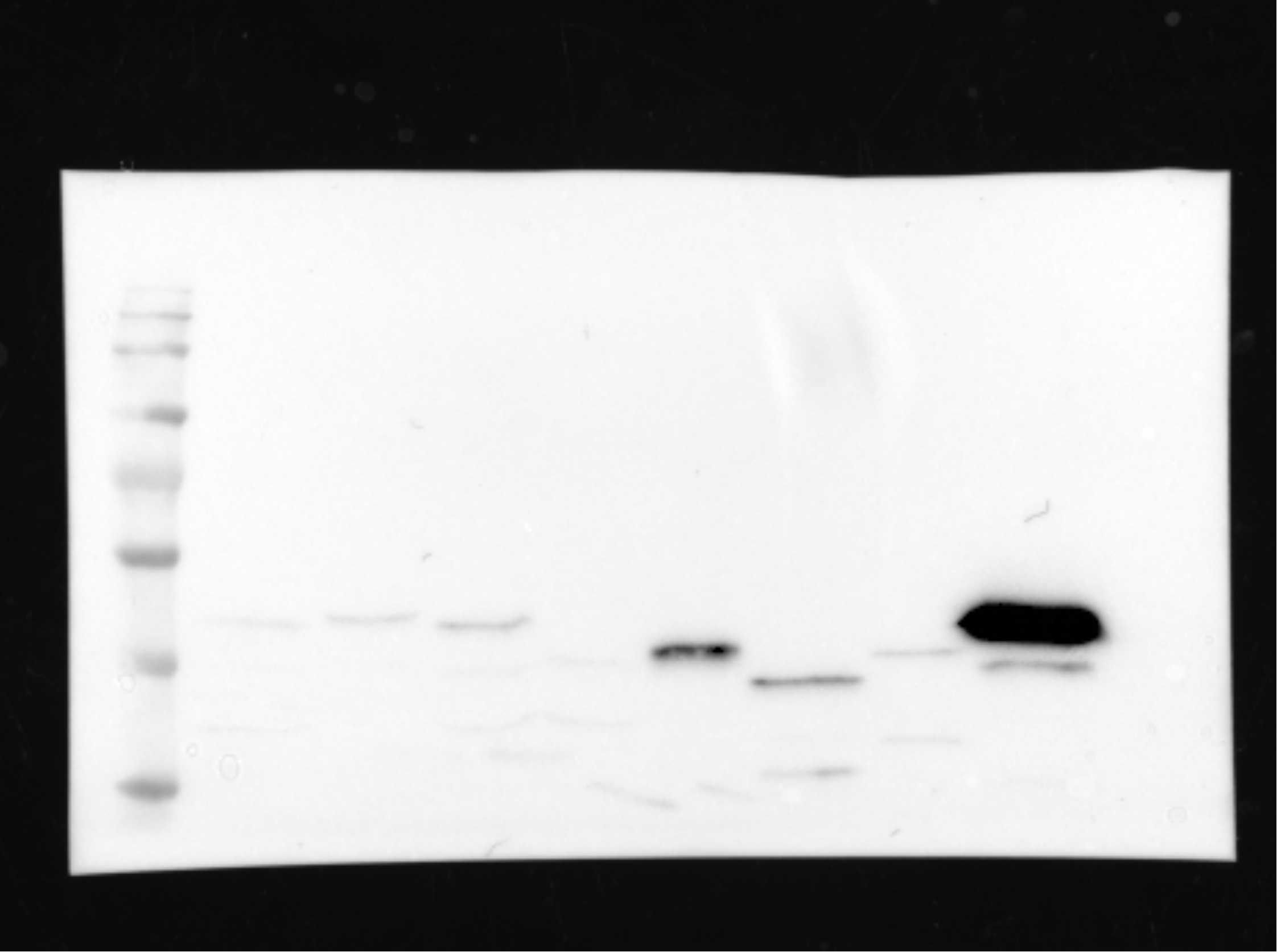

Supplement: Supplementary file 10 — Appendix Figures Source Data [file 44319_2024_203_MOESM10_ESM.zip › Appendix3_RASSF5/Toprow/Leftmost/Pulldown.jpg]

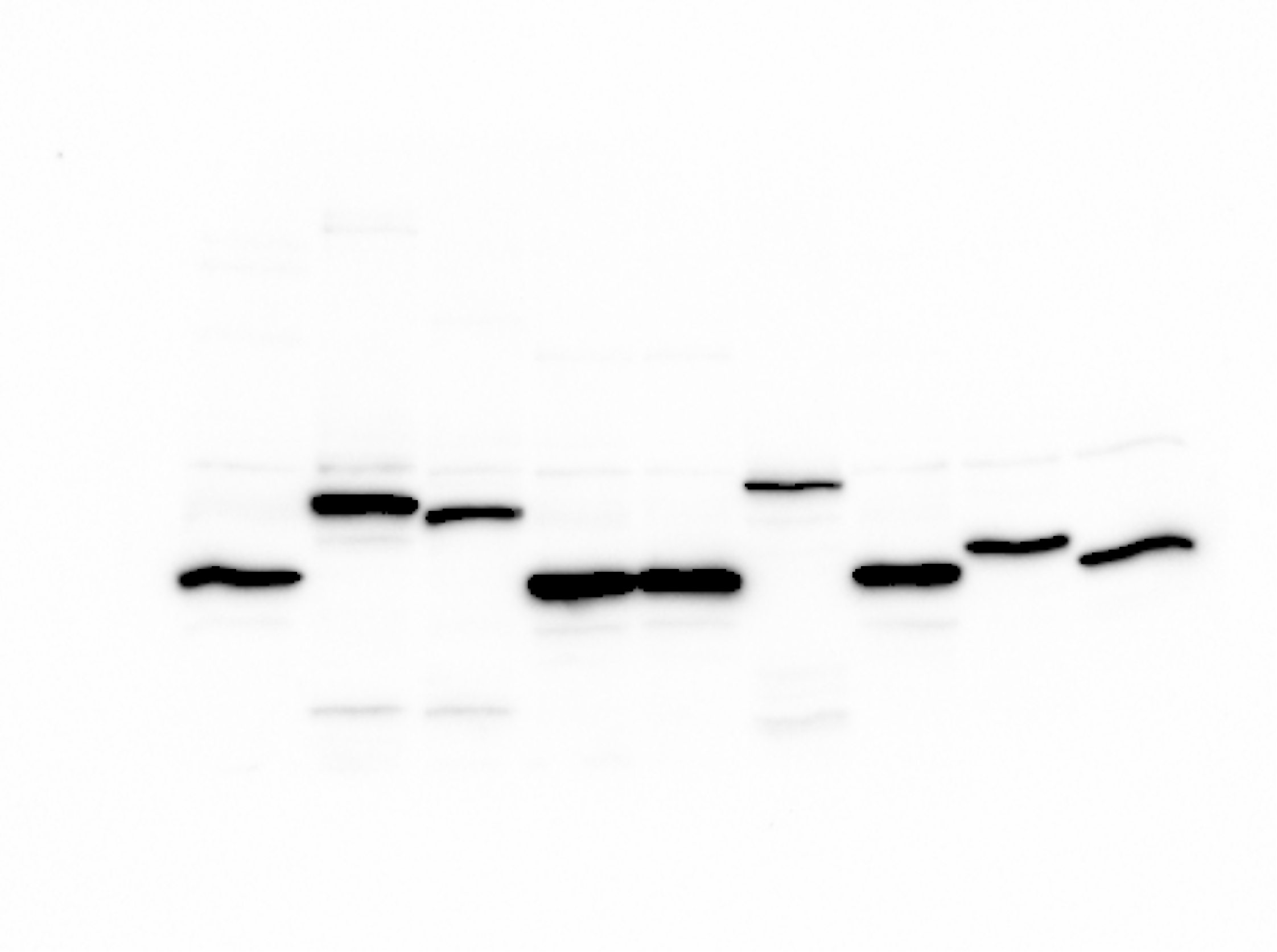

Supplement: Supplementary file 10 — Appendix Figures Source Data [file 44319_2024_203_MOESM10_ESM.zip › Appendix3_RASSF5/Toprow/Middle/Lysate.jpg]

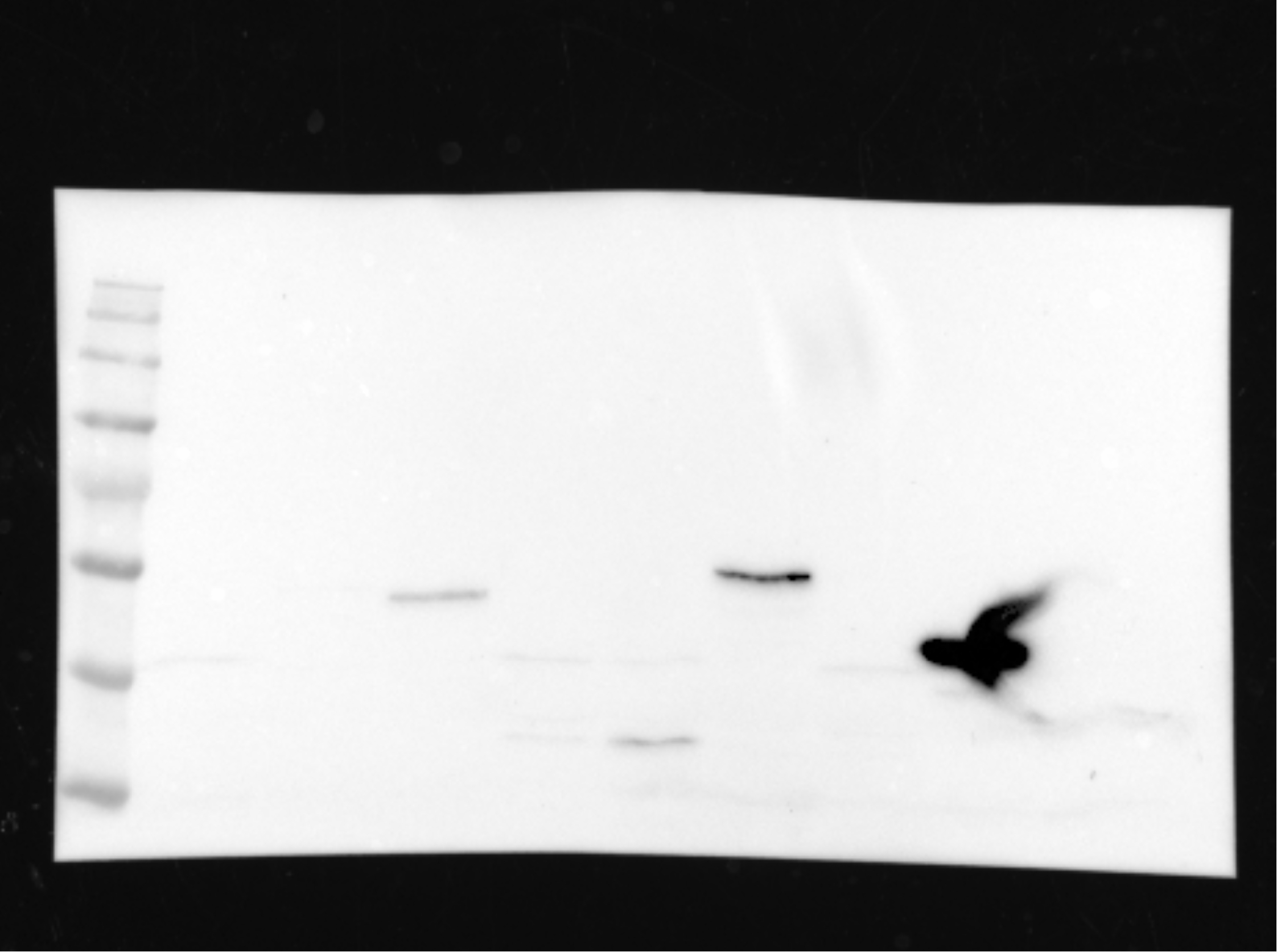

Supplement: Supplementary file 10 — Appendix Figures Source Data [file 44319_2024_203_MOESM10_ESM.zip › Appendix3_RASSF5/Toprow/Middle/Pulldown.jpg]

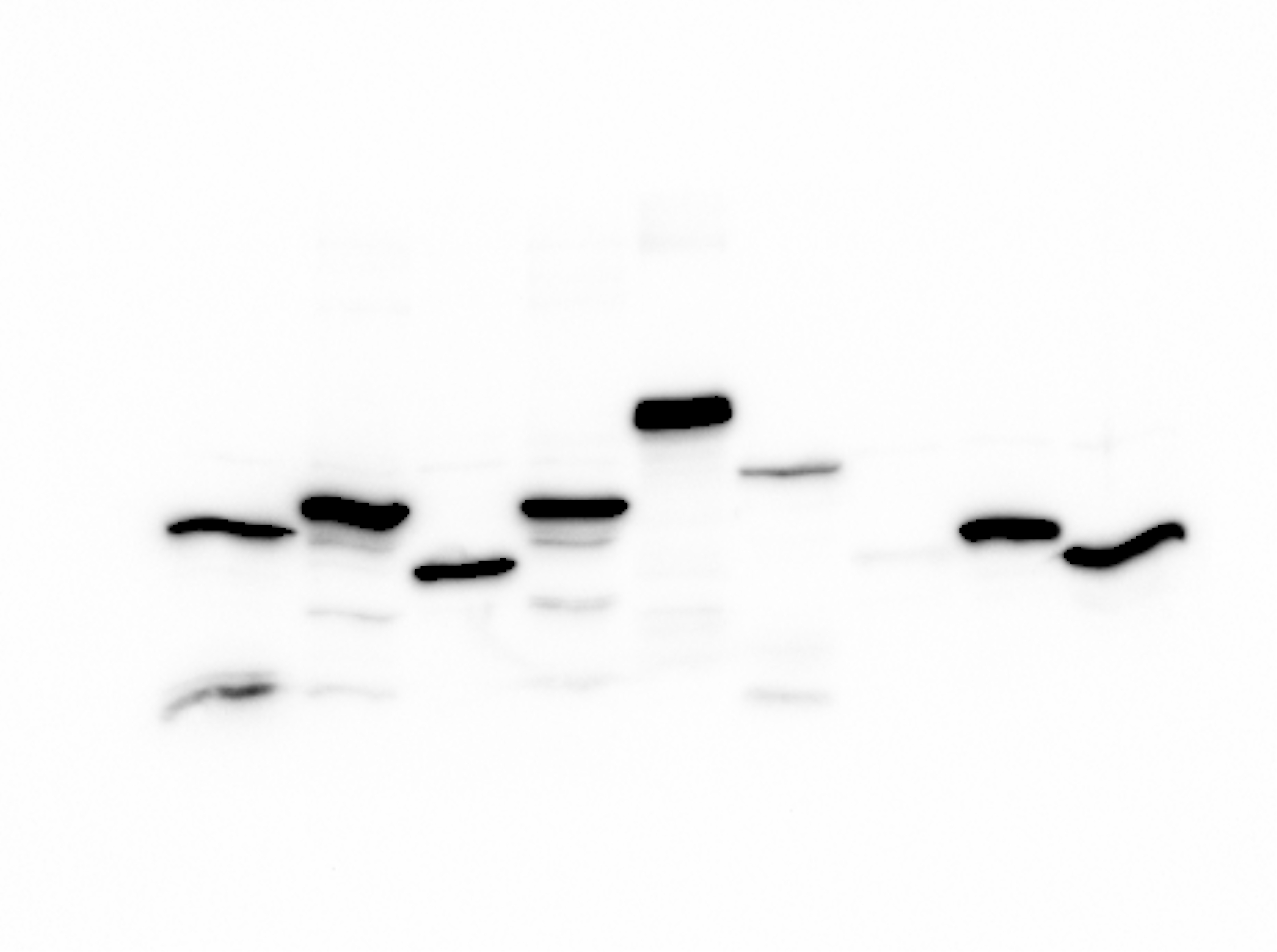

Supplement: Supplementary file 10 — Appendix Figures Source Data [file 44319_2024_203_MOESM10_ESM.zip › Appendix3_RASSF5/Toprow/Rightmost/Lysate.jpg]

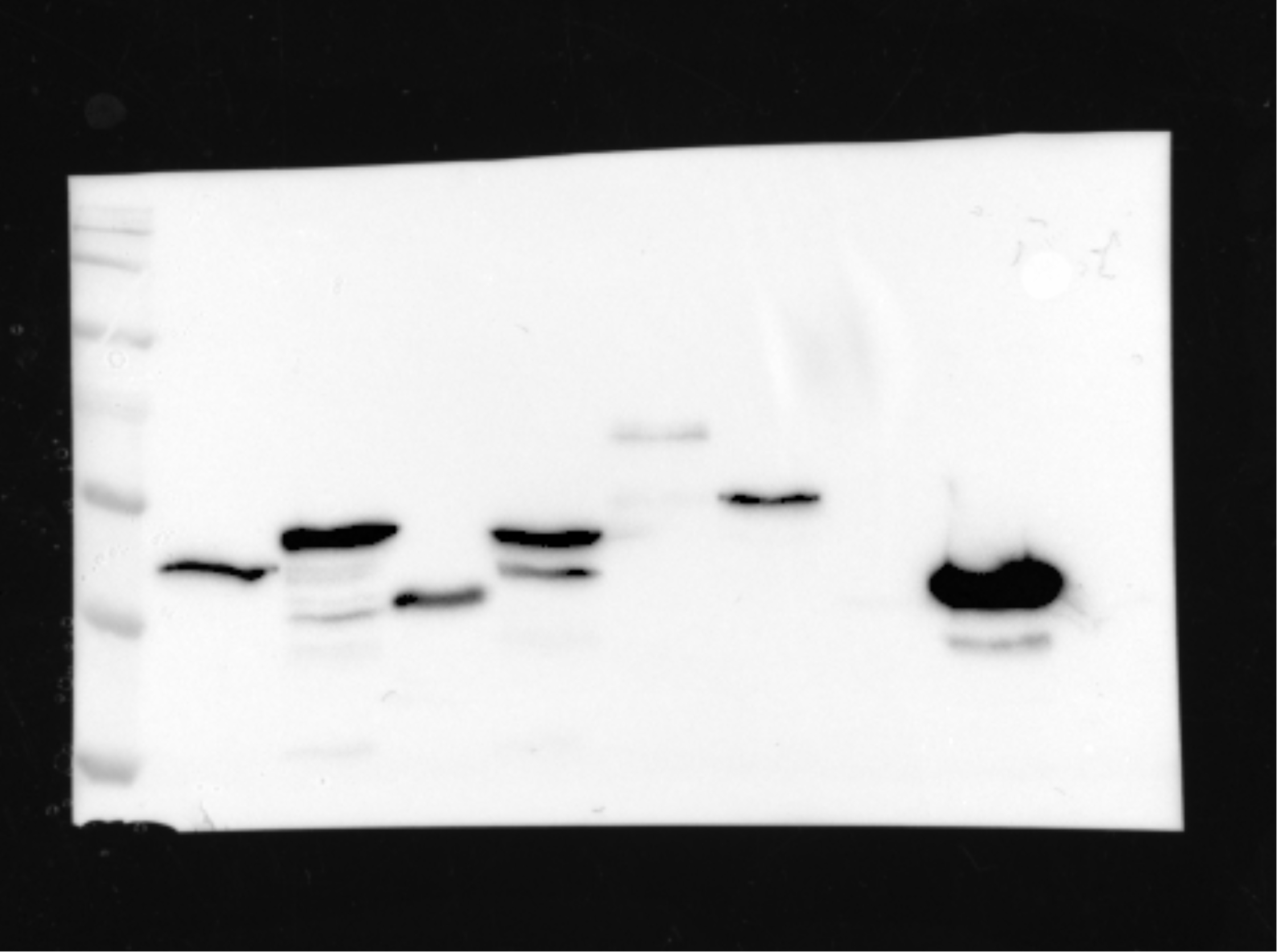

Supplement: Supplementary file 10 — Appendix Figures Source Data [file 44319_2024_203_MOESM10_ESM.zip › Appendix3_RASSF5/Toprow/Rightmost/Pulldown.jpg]

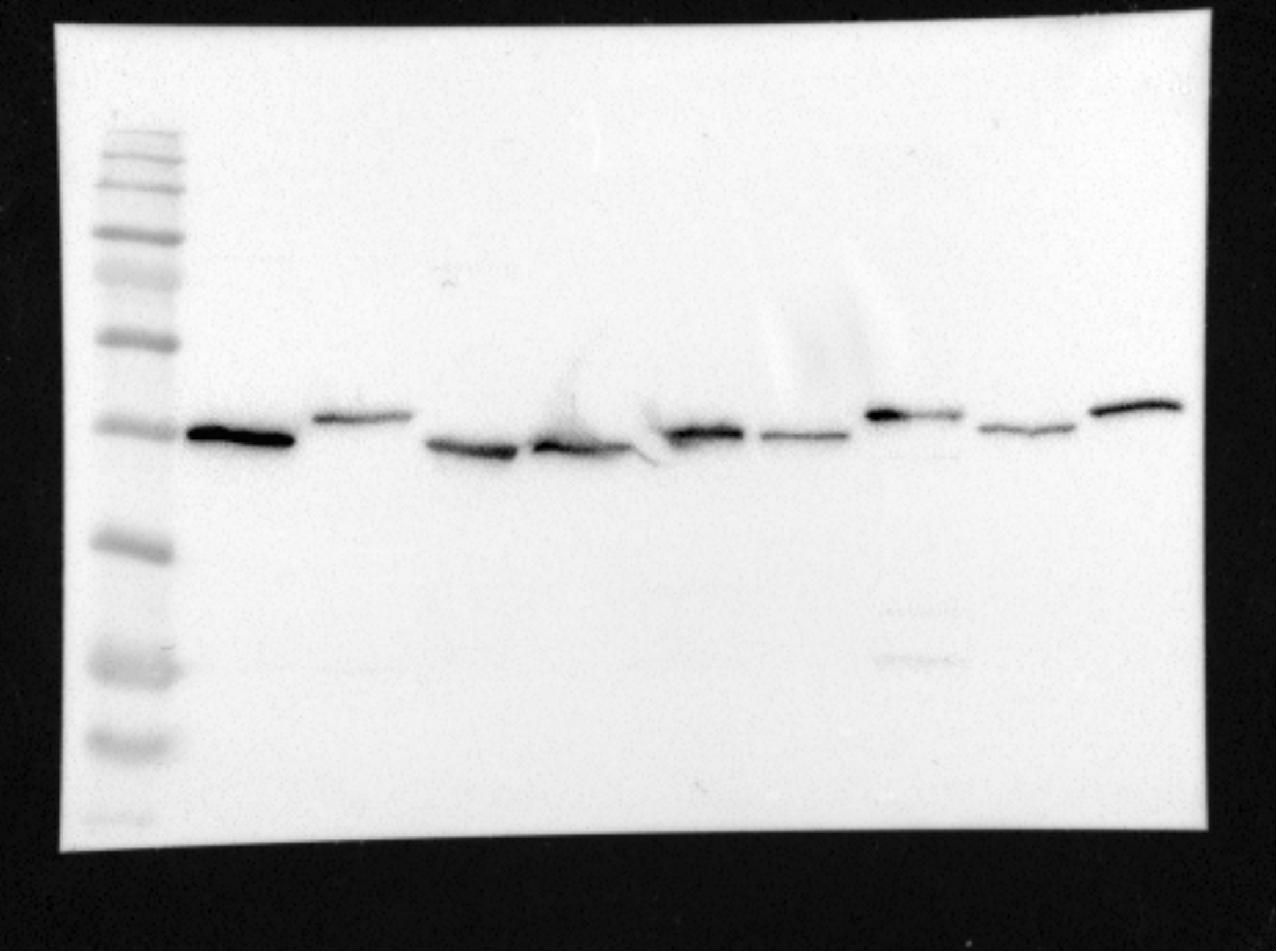

Supplement: Supplementary file 10 — Appendix Figures Source Data [file 44319_2024_203_MOESM10_ESM.zip › Appendix1_GST/Fourthrow/Leftblot/Lysate.jpg]

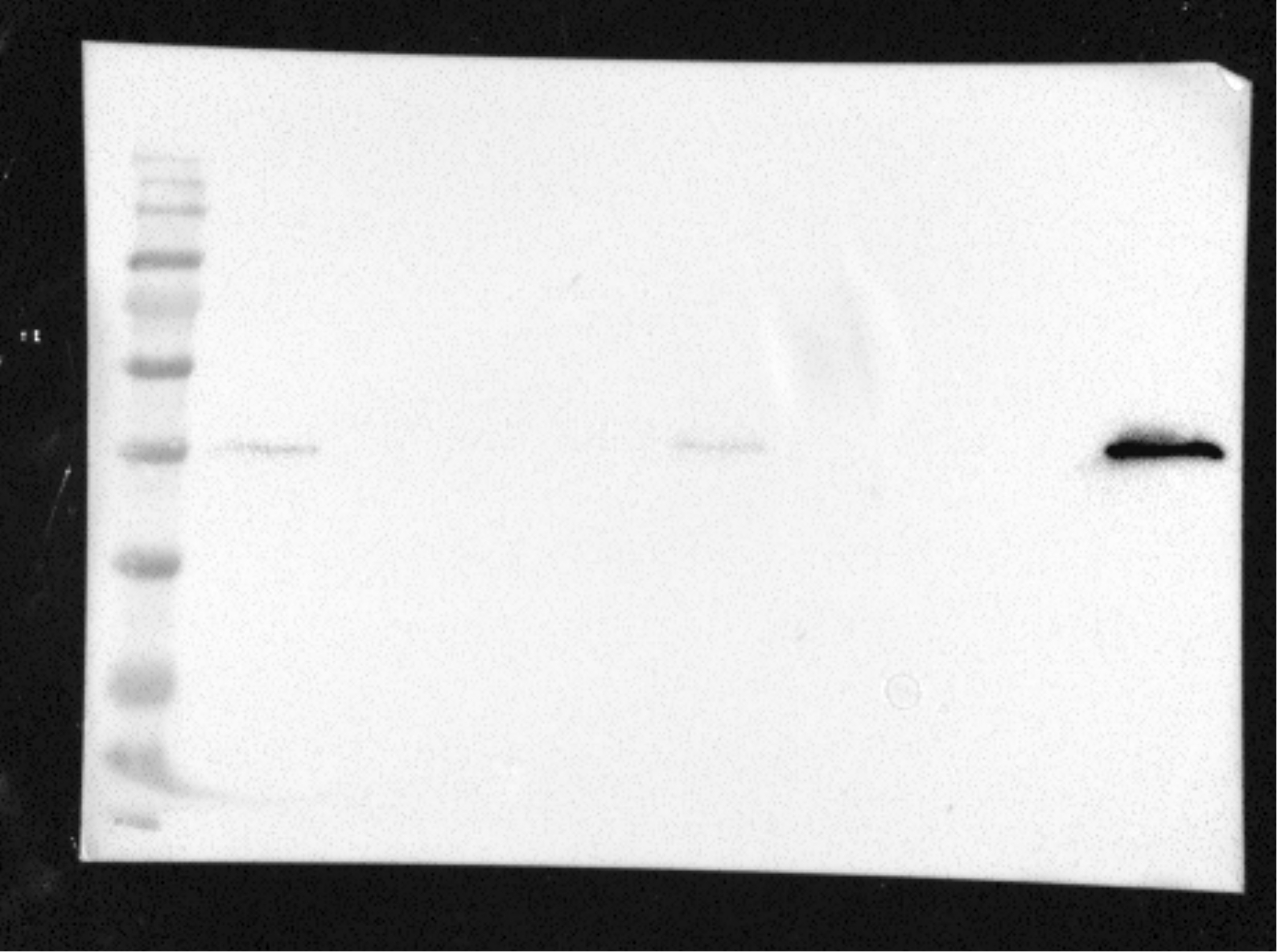

Supplement: Supplementary file 10 — Appendix Figures Source Data [file 44319_2024_203_MOESM10_ESM.zip › Appendix1_GST/Fourthrow/Leftblot/Pulldown.jpg]

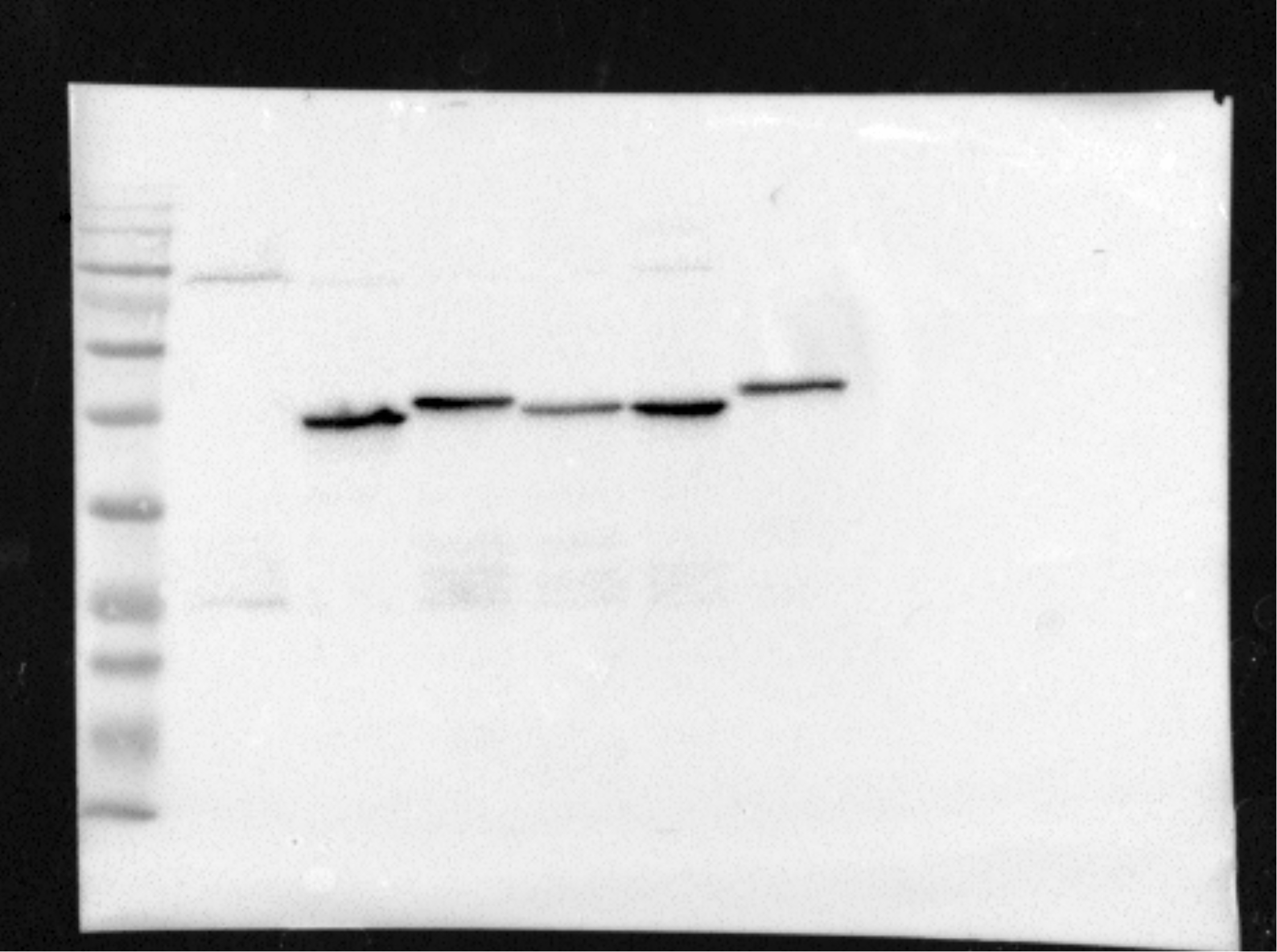

Supplement: Supplementary file 10 — Appendix Figures Source Data [file 44319_2024_203_MOESM10_ESM.zip › Appendix1_GST/Fourthrow/Rightblot/Lysate.jpg]

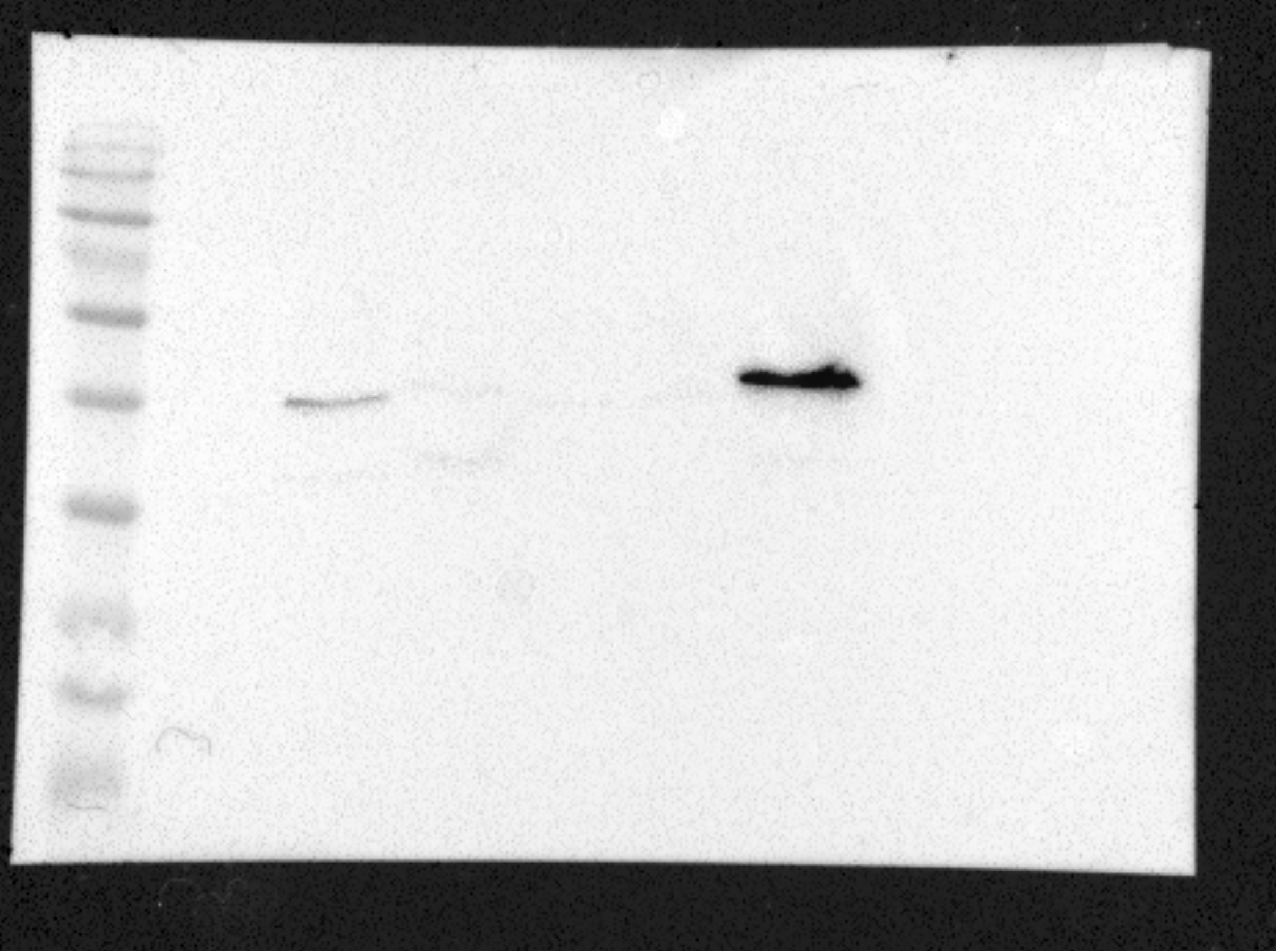

Supplement: Supplementary file 10 — Appendix Figures Source Data [file 44319_2024_203_MOESM10_ESM.zip › Appendix1_GST/Fourthrow/Rightblot/Pulldown.jpg]

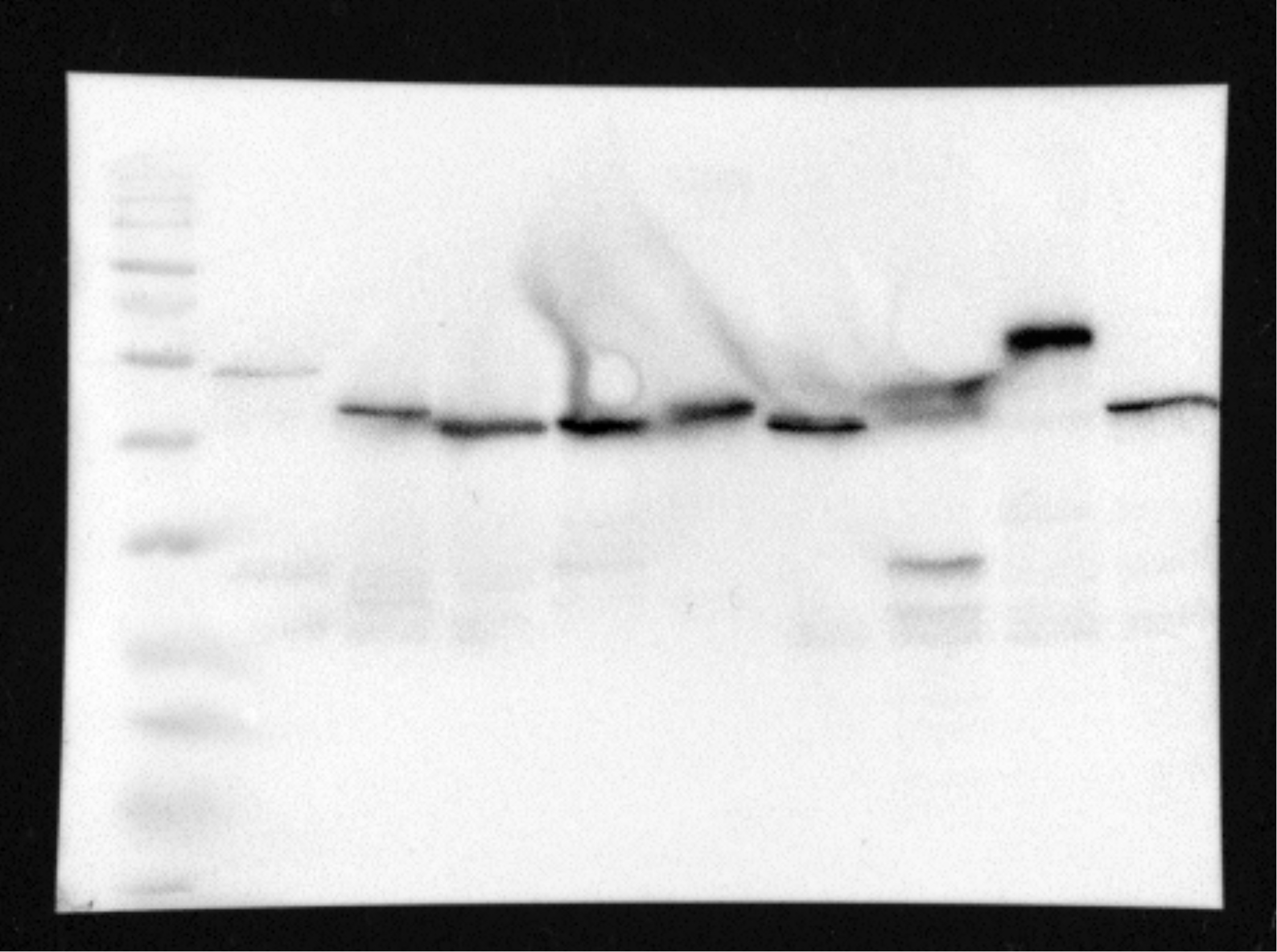

Supplement: Supplementary file 10 — Appendix Figures Source Data [file 44319_2024_203_MOESM10_ESM.zip › Appendix1_GST/Secondrow/Leftmost/Lysate.jpg]

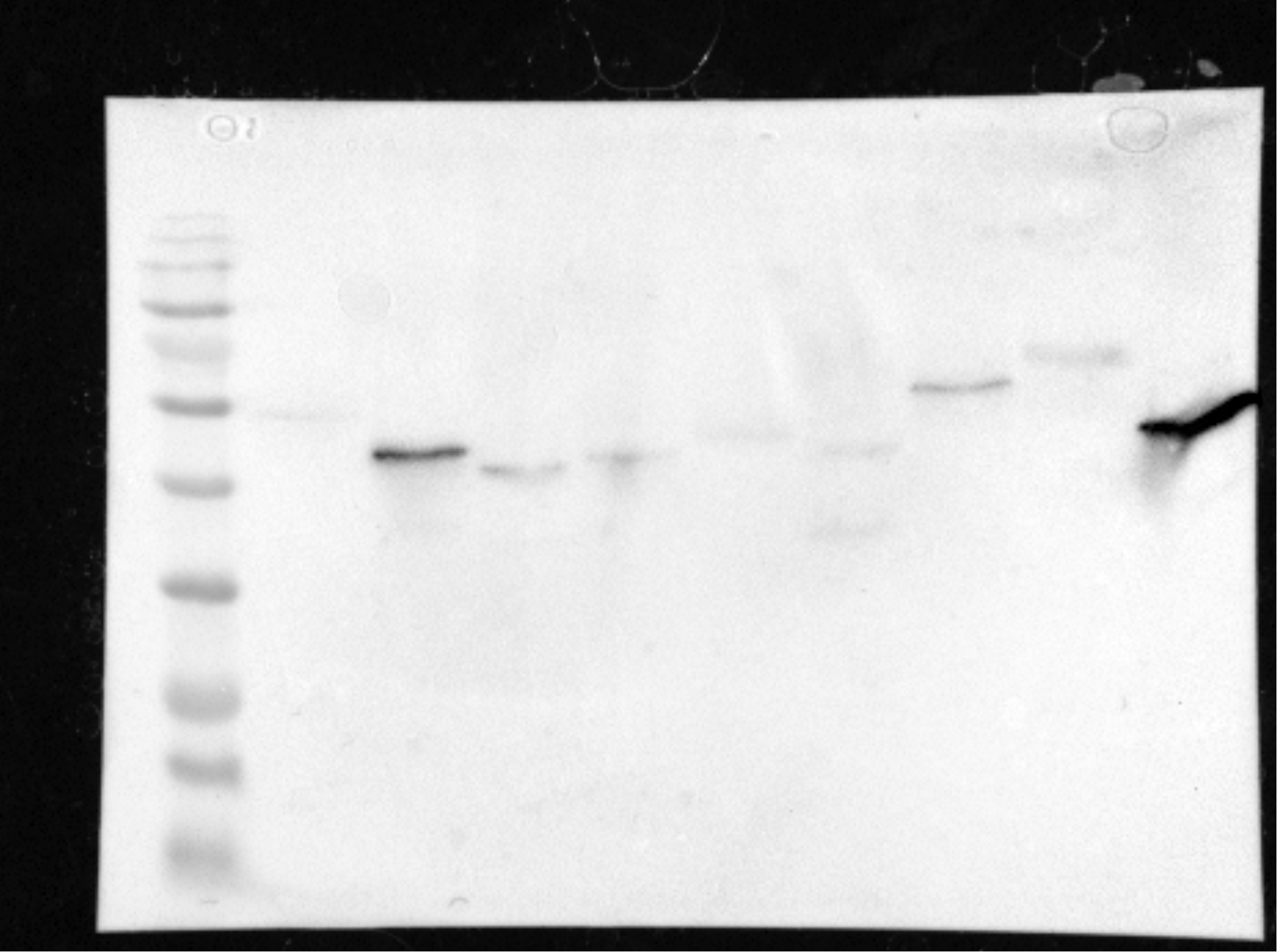

Supplement: Supplementary file 10 — Appendix Figures Source Data [file 44319_2024_203_MOESM10_ESM.zip › Appendix1_GST/Secondrow/Leftmost/Pulldown.jpg]

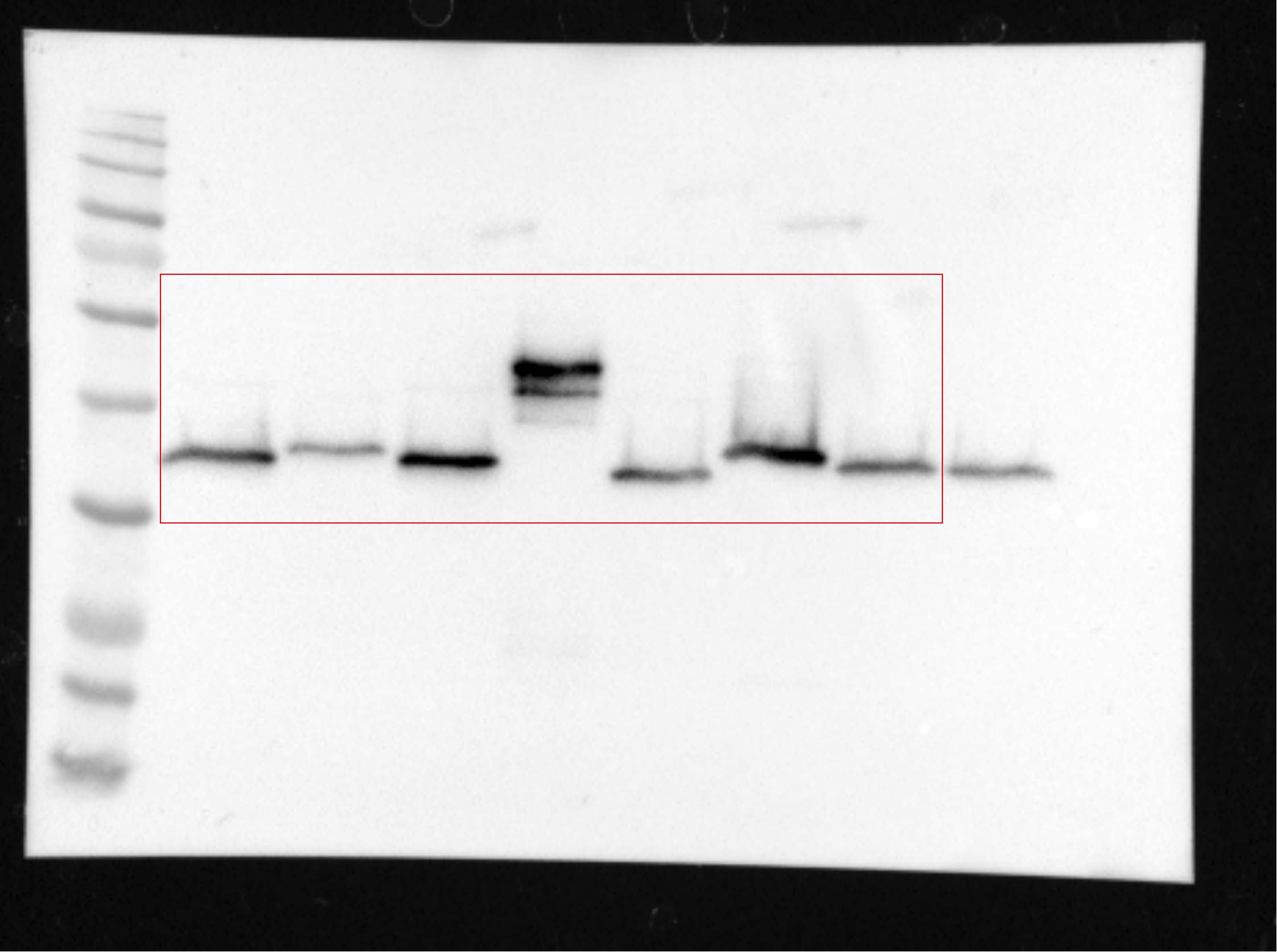

Supplement: Supplementary file 10 — Appendix Figures Source Data [file 44319_2024_203_MOESM10_ESM.zip › Appendix1_GST/Secondrow/Middle/Lysate.jpg]

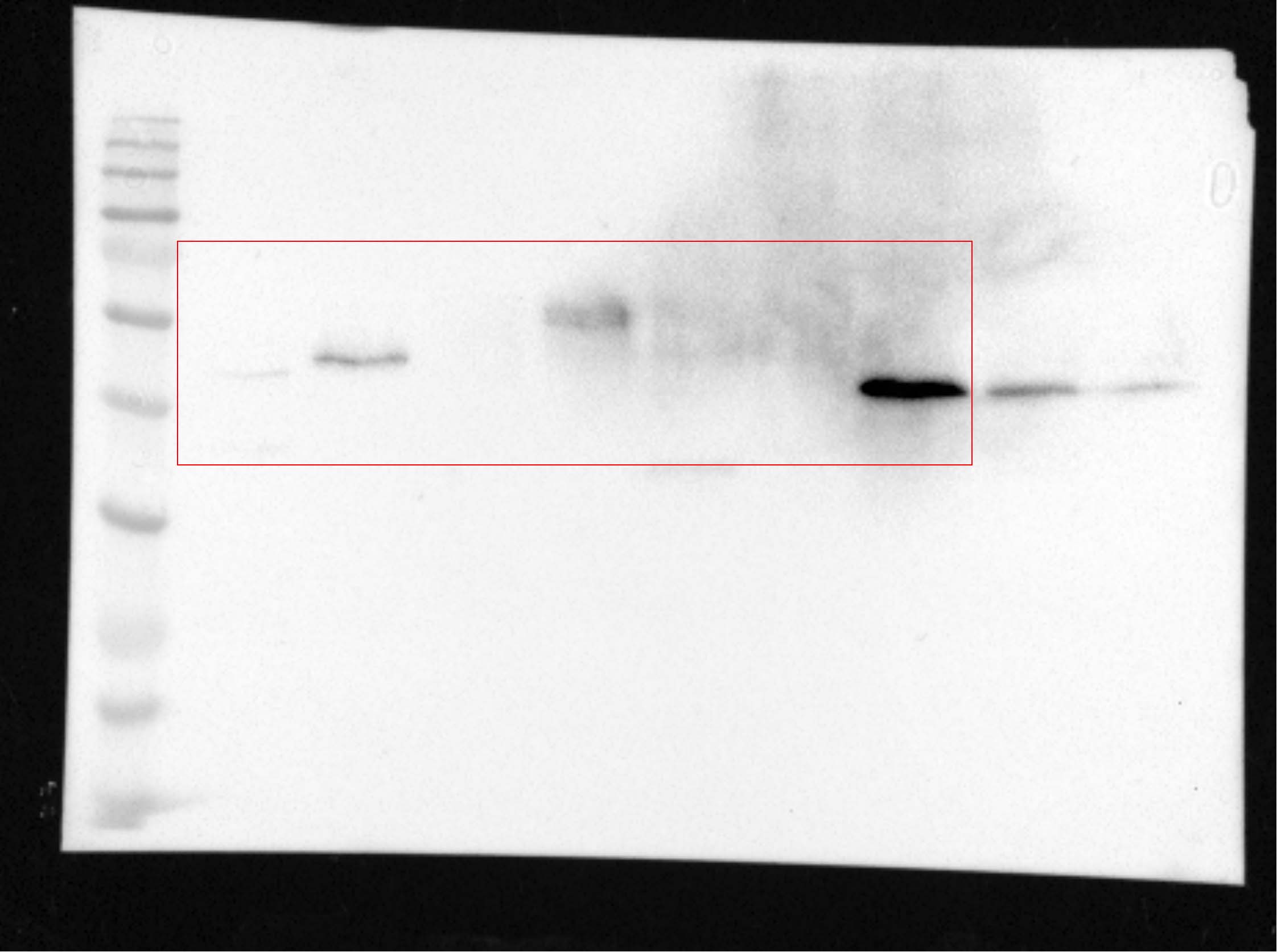

Supplement: Supplementary file 10 — Appendix Figures Source Data [file 44319_2024_203_MOESM10_ESM.zip › Appendix1_GST/Secondrow/Middle/Pulldown.jpg]

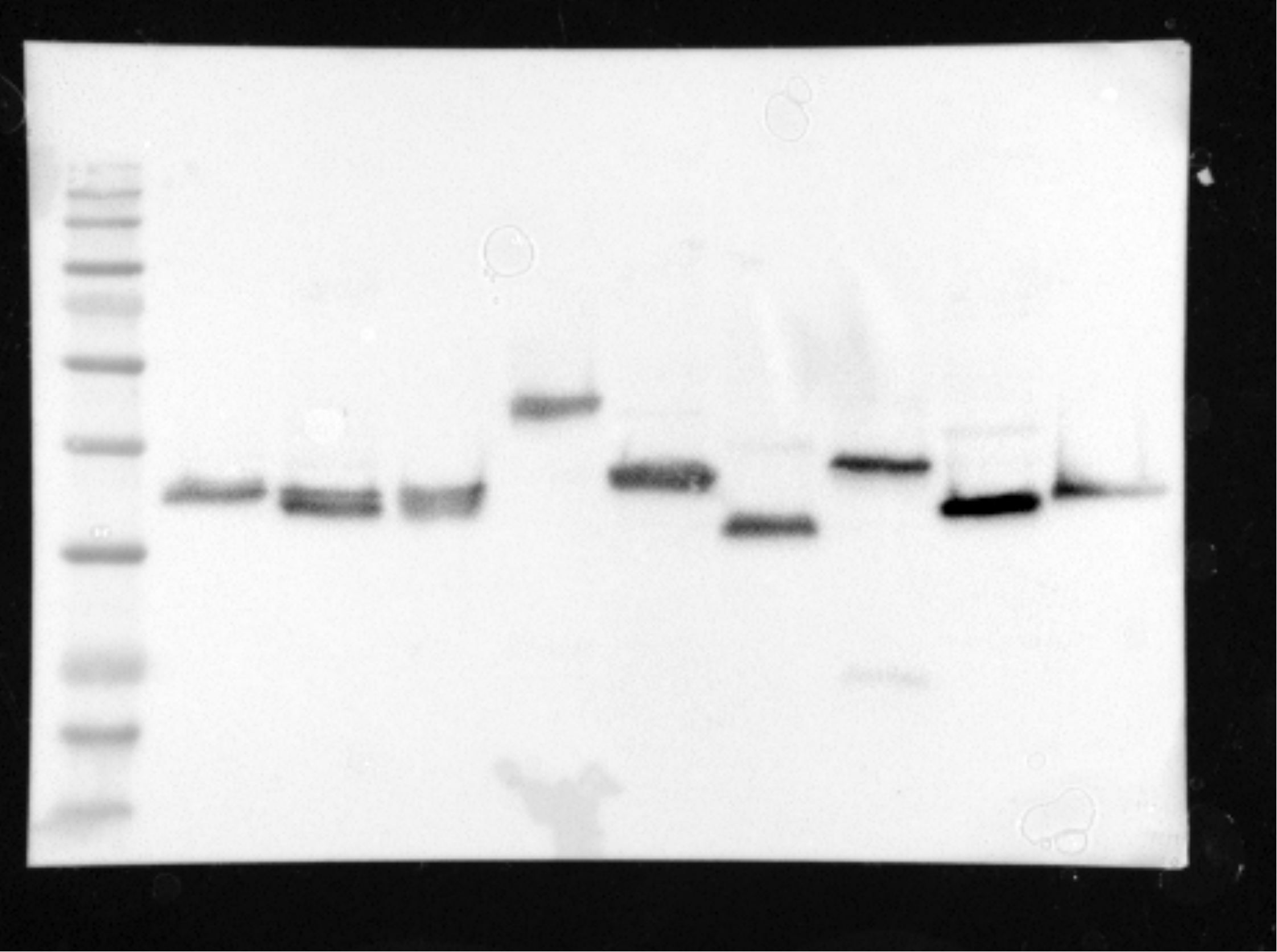

Supplement: Supplementary file 10 — Appendix Figures Source Data [file 44319_2024_203_MOESM10_ESM.zip › Appendix1_GST/Secondrow/Rightmost/Lysate.jpg]
