# Supplementary figures and images for: Complex interplay between RAS GTPases and RASSF effectors regulates subcellular localization of YAP (part 4 of 4)
Source: EMBO Rep. 2024 Jul 15;25(8):22. doi: 10.1038/s44319-024-00203-9 (PMC11316025; doi:10.1038/s44319-024-00203-9)

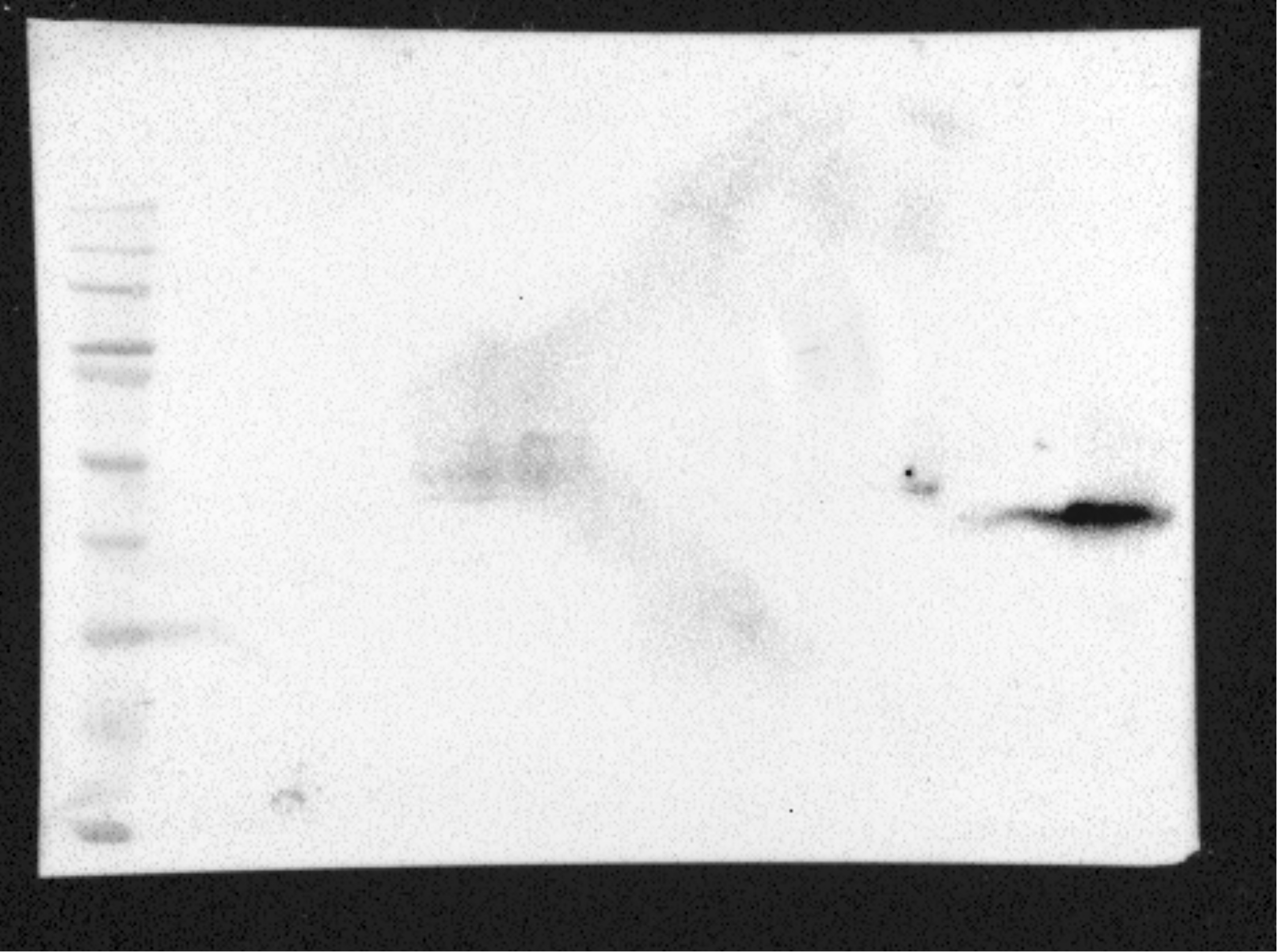

Supplement: Supplementary file 10 — Appendix Figures Source Data [file 44319_2024_203_MOESM10_ESM.zip › Appendix1_GST/Secondrow/Rightmost/Pulldown.jpg]

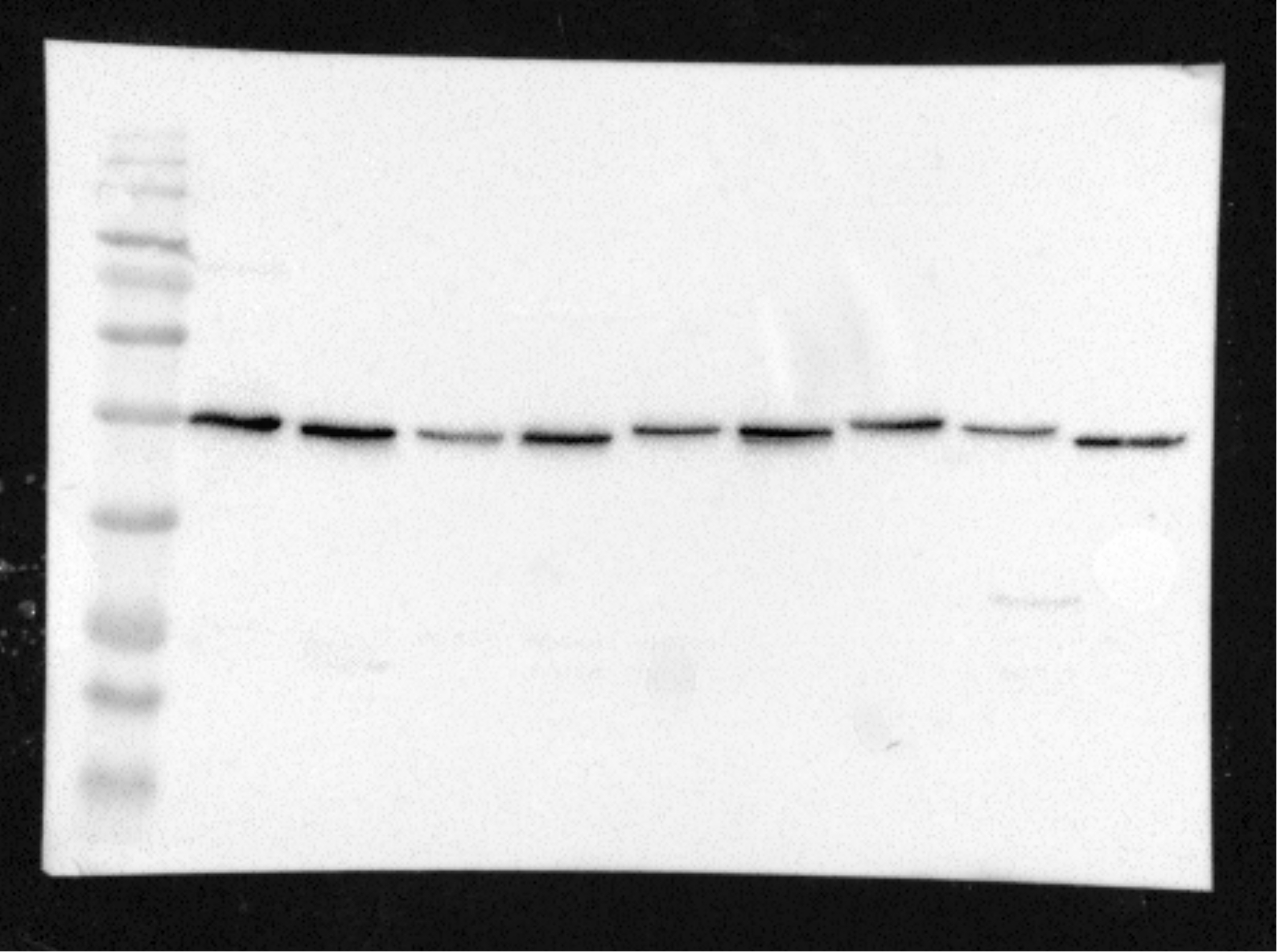

Supplement: Supplementary file 10 — Appendix Figures Source Data [file 44319_2024_203_MOESM10_ESM.zip › Appendix1_GST/Thirdrow/Leftmost/Lysate.jpg]

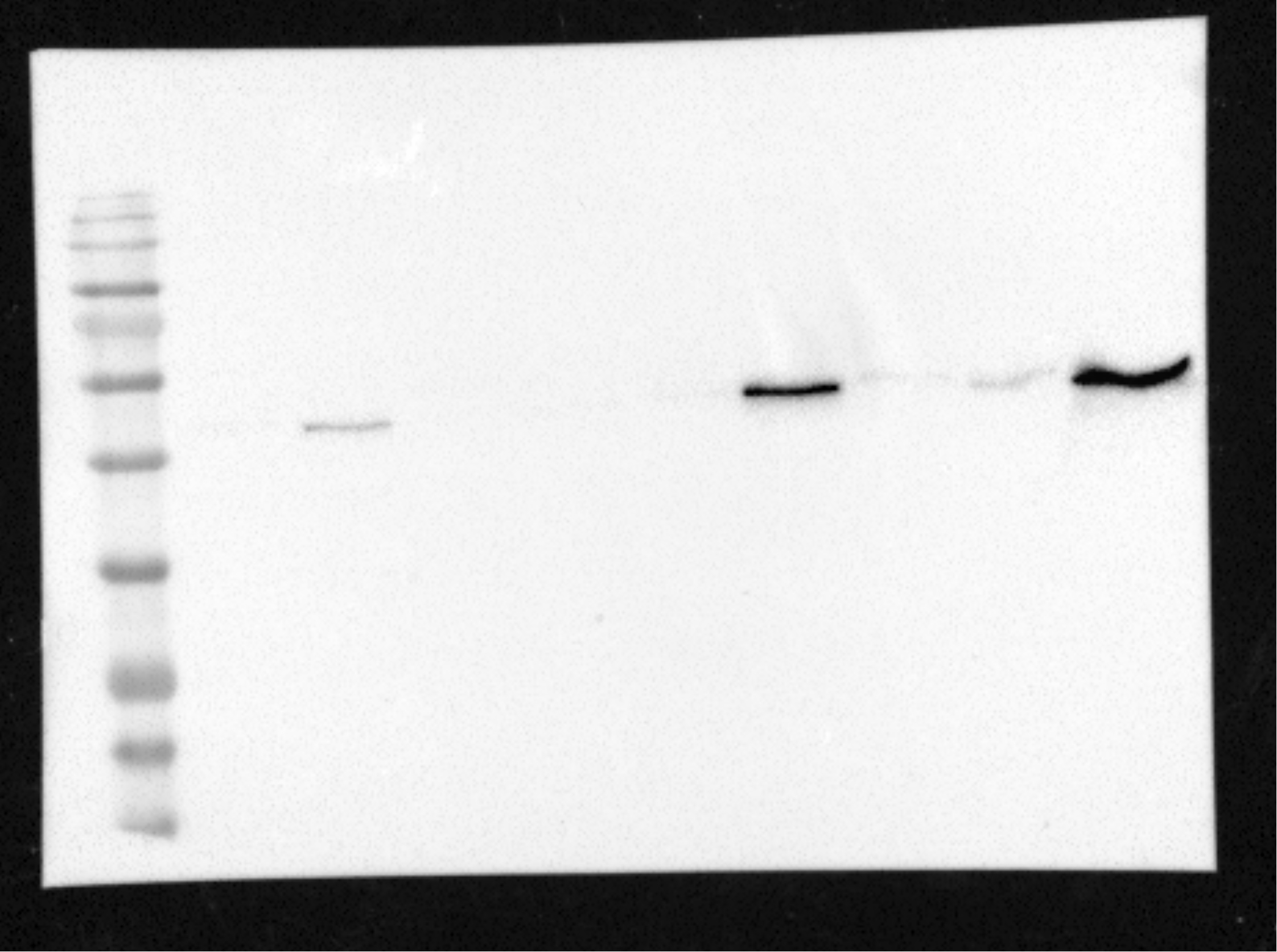

Supplement: Supplementary file 10 — Appendix Figures Source Data [file 44319_2024_203_MOESM10_ESM.zip › Appendix1_GST/Thirdrow/Leftmost/Pulldown.jpg]

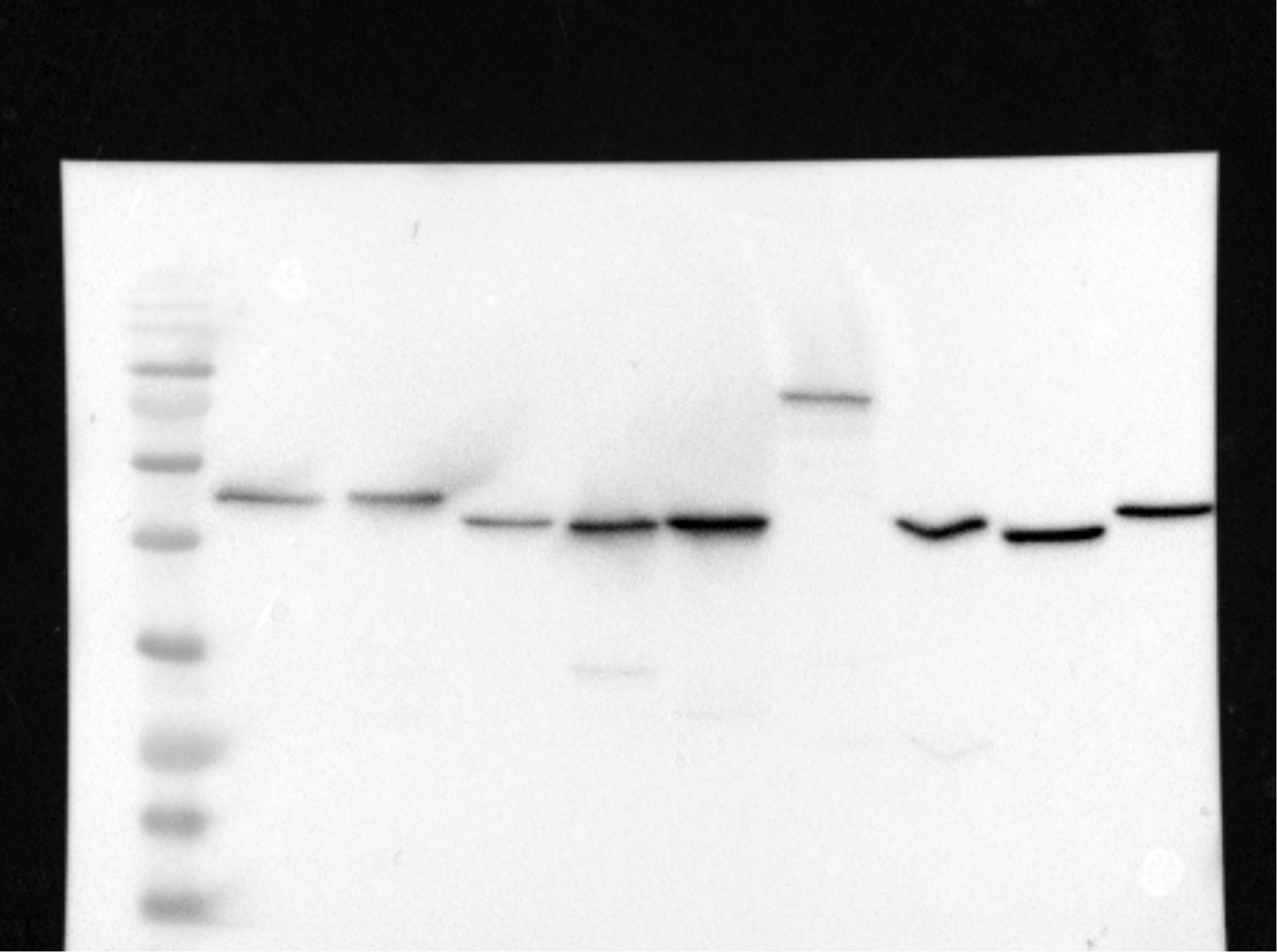

Supplement: Supplementary file 10 — Appendix Figures Source Data [file 44319_2024_203_MOESM10_ESM.zip › Appendix1_GST/Thirdrow/Middle/Lysate.jpg]

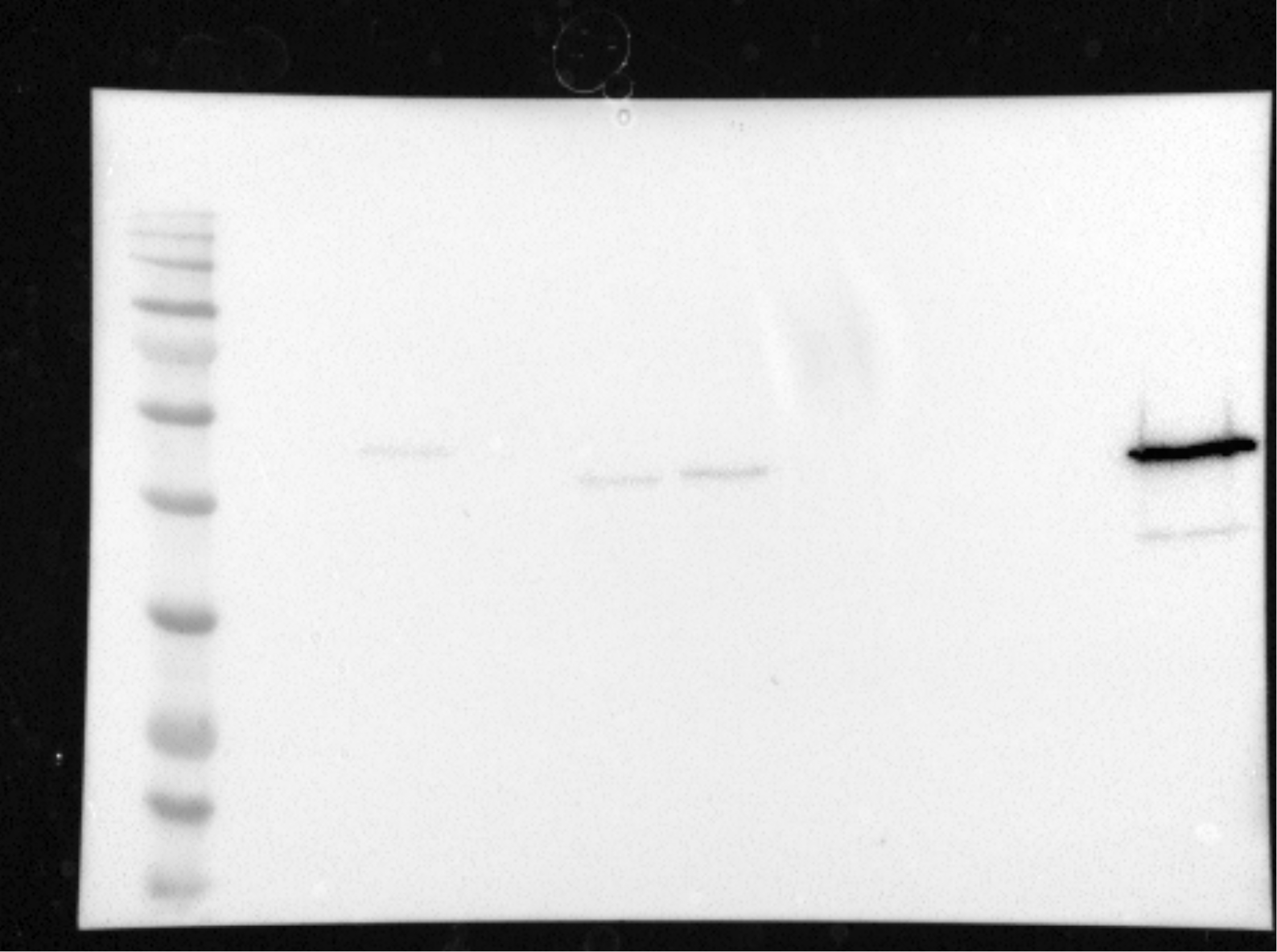

Supplement: Supplementary file 10 — Appendix Figures Source Data [file 44319_2024_203_MOESM10_ESM.zip › Appendix1_GST/Thirdrow/Middle/Pulldown.jpg]

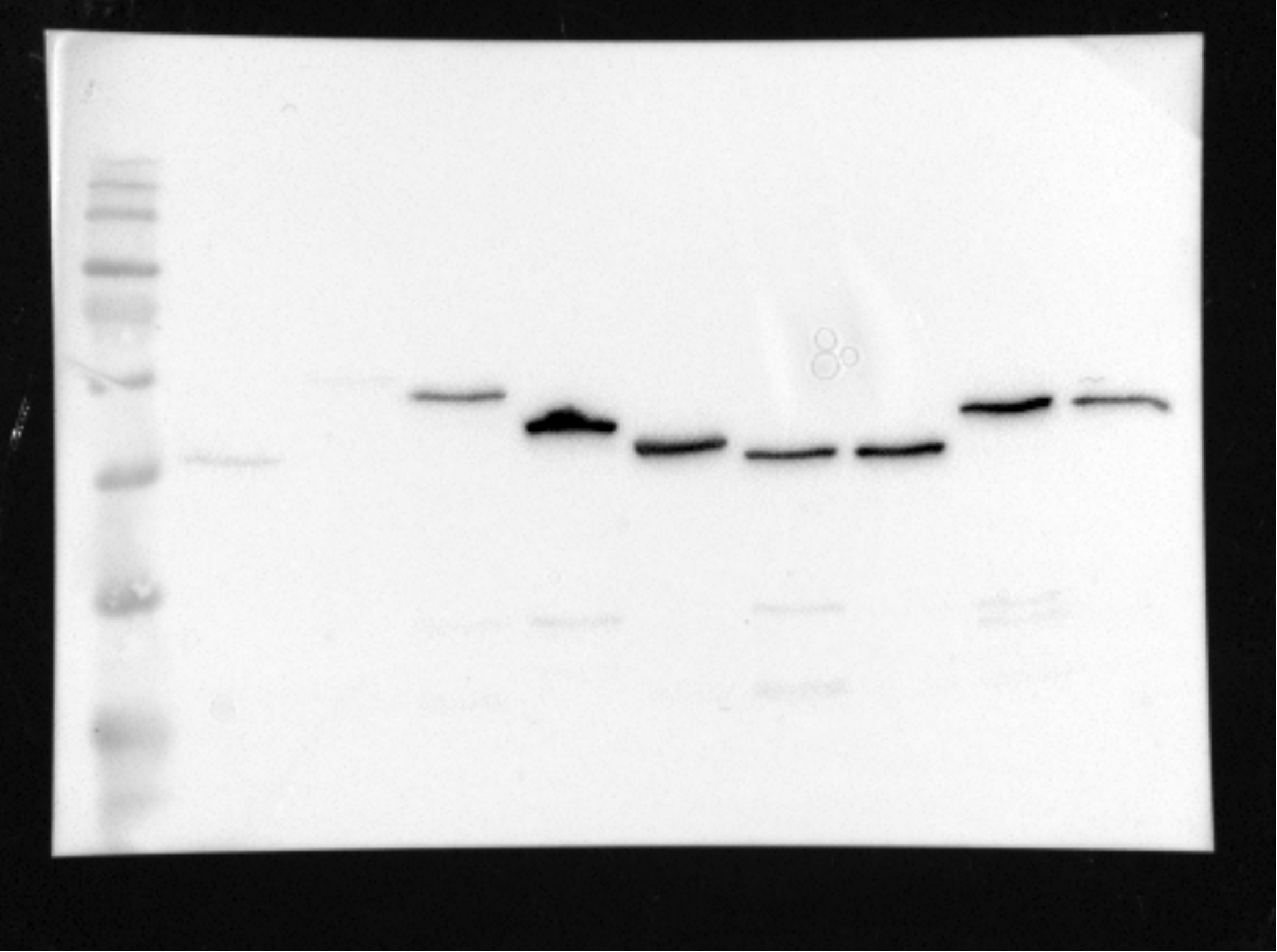

Supplement: Supplementary file 10 — Appendix Figures Source Data [file 44319_2024_203_MOESM10_ESM.zip › Appendix1_GST/Thirdrow/Rightmost/Lysate.jpg]

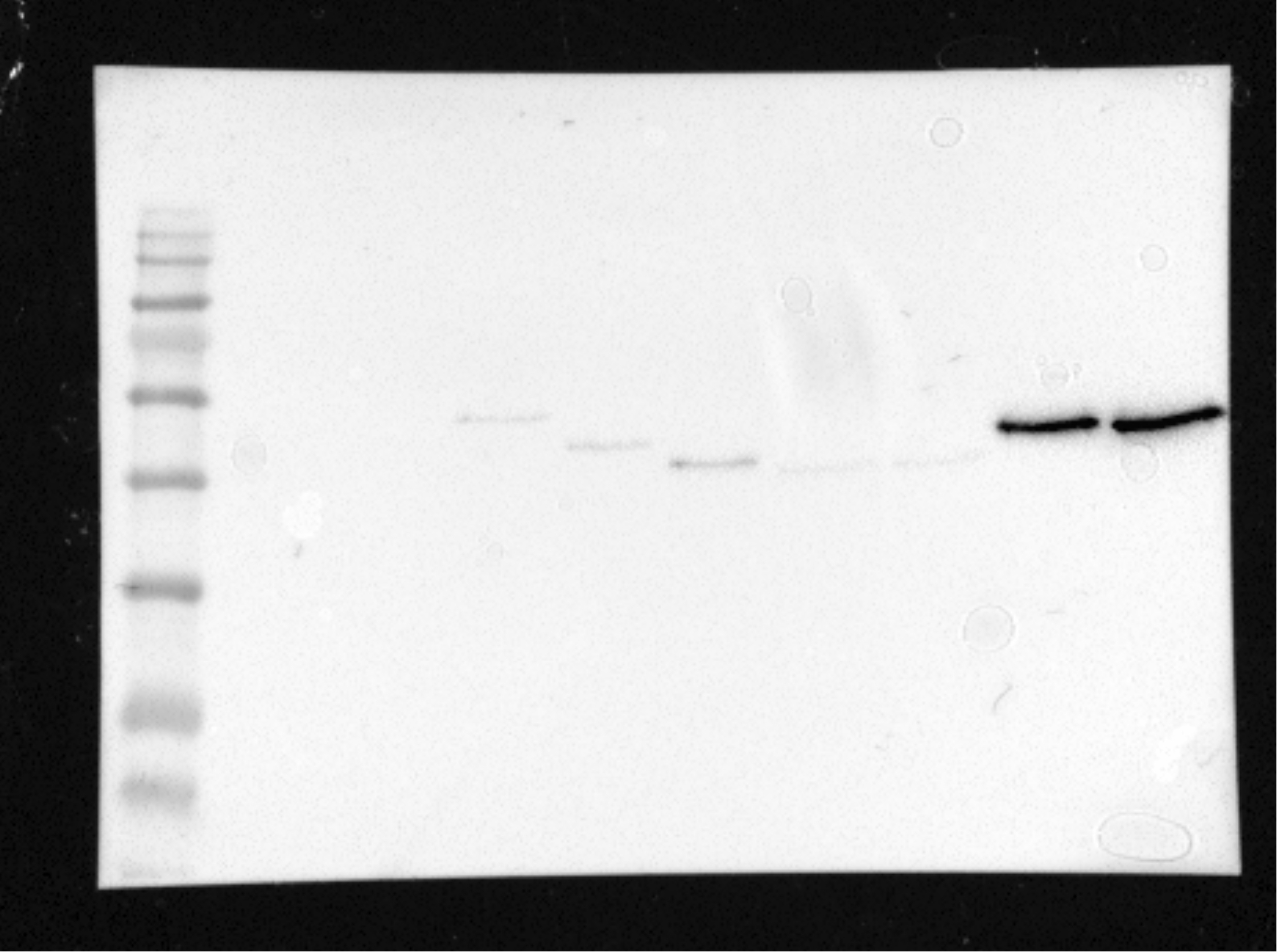

Supplement: Supplementary file 10 — Appendix Figures Source Data [file 44319_2024_203_MOESM10_ESM.zip › Appendix1_GST/Thirdrow/Rightmost/Pulldown.jpg]

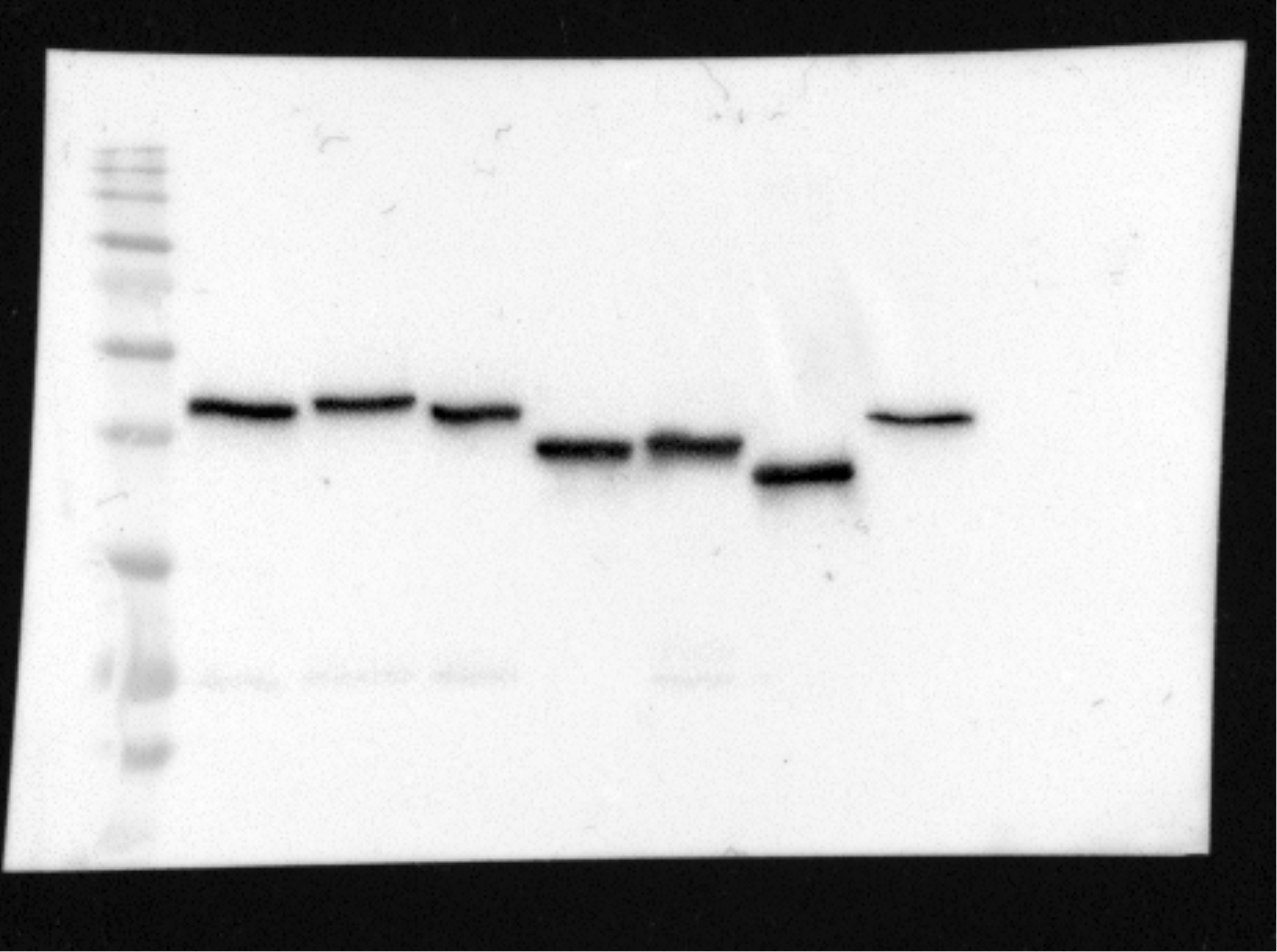

Supplement: Supplementary file 10 — Appendix Figures Source Data [file 44319_2024_203_MOESM10_ESM.zip › Appendix1_GST/Toprow/Leftmost/Lysate.jpg]

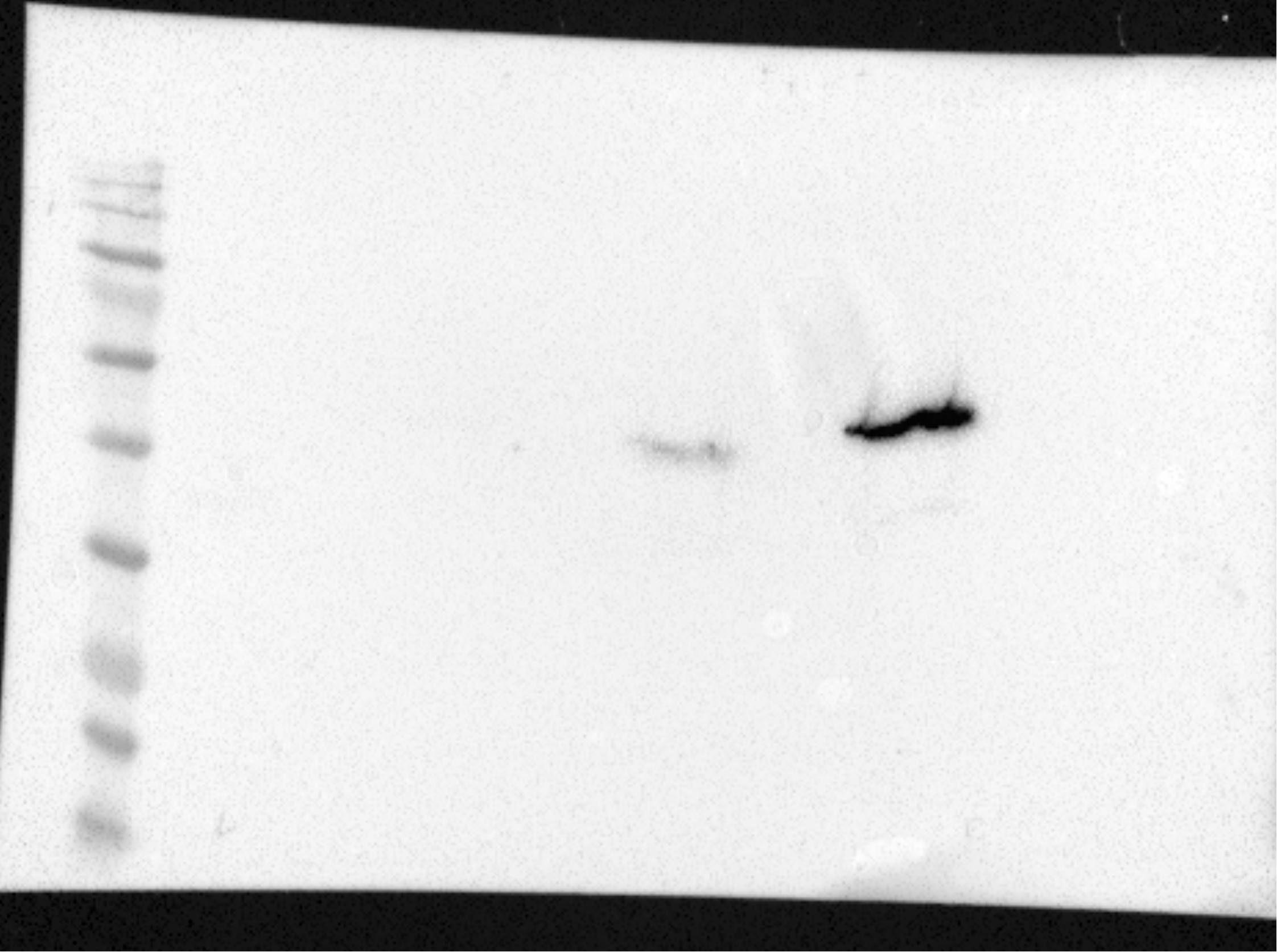

Supplement: Supplementary file 10 — Appendix Figures Source Data [file 44319_2024_203_MOESM10_ESM.zip › Appendix1_GST/Toprow/Leftmost/Pulldown.jpg]

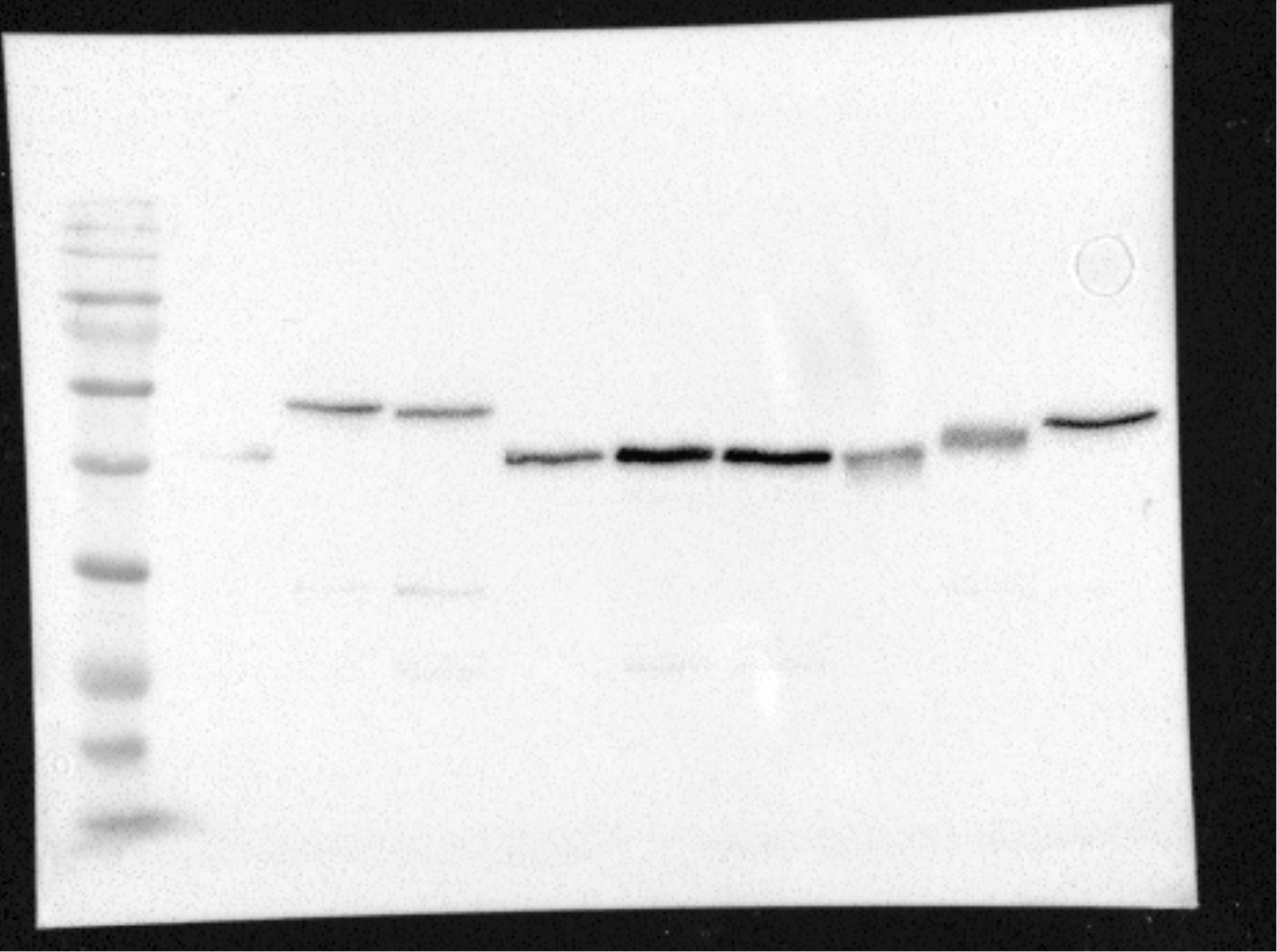

Supplement: Supplementary file 10 — Appendix Figures Source Data [file 44319_2024_203_MOESM10_ESM.zip › Appendix1_GST/Toprow/Middle/Lysate.jpg]

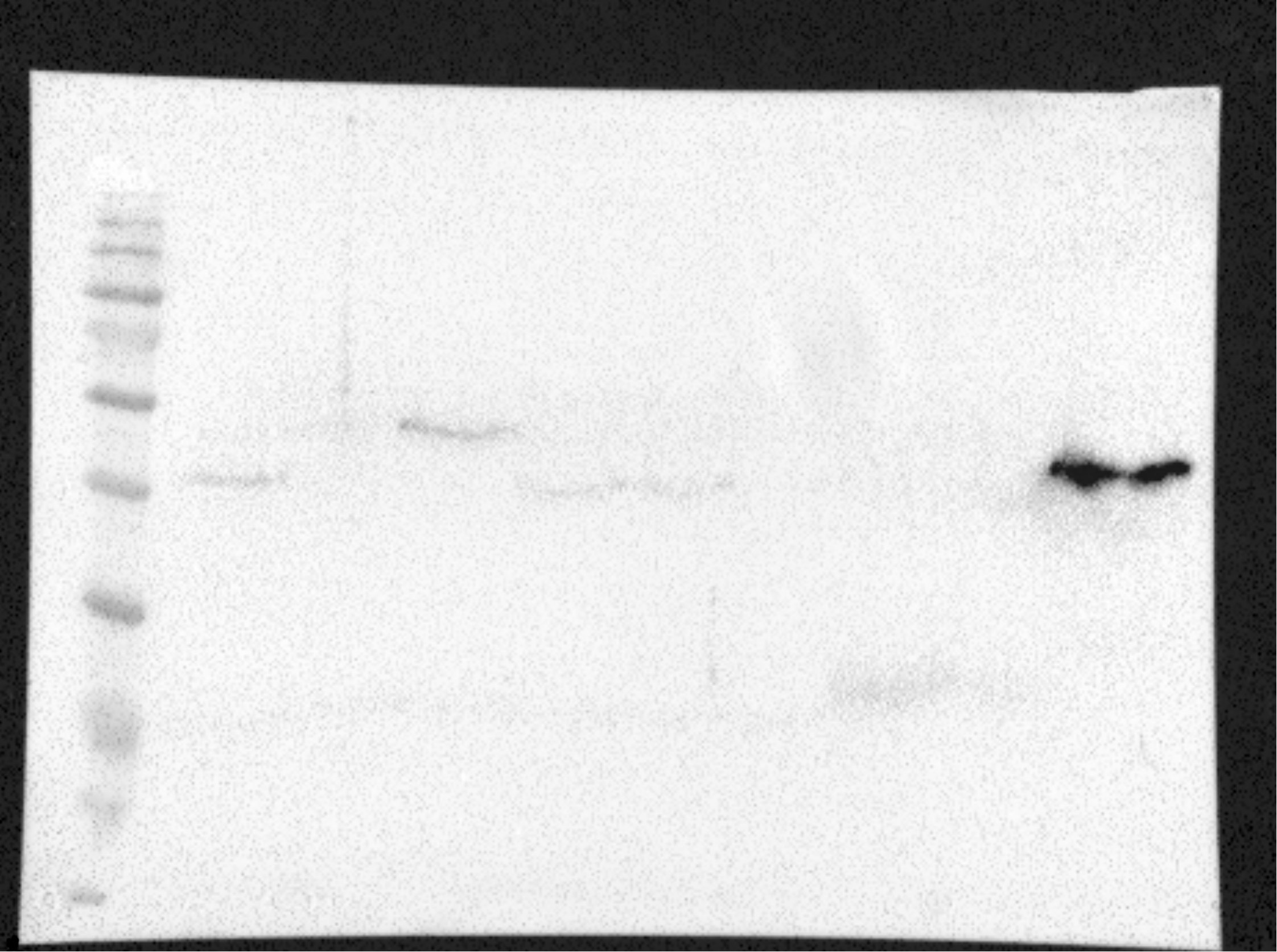

Supplement: Supplementary file 10 — Appendix Figures Source Data [file 44319_2024_203_MOESM10_ESM.zip › Appendix1_GST/Toprow/Middle/Pulldown.jpg]

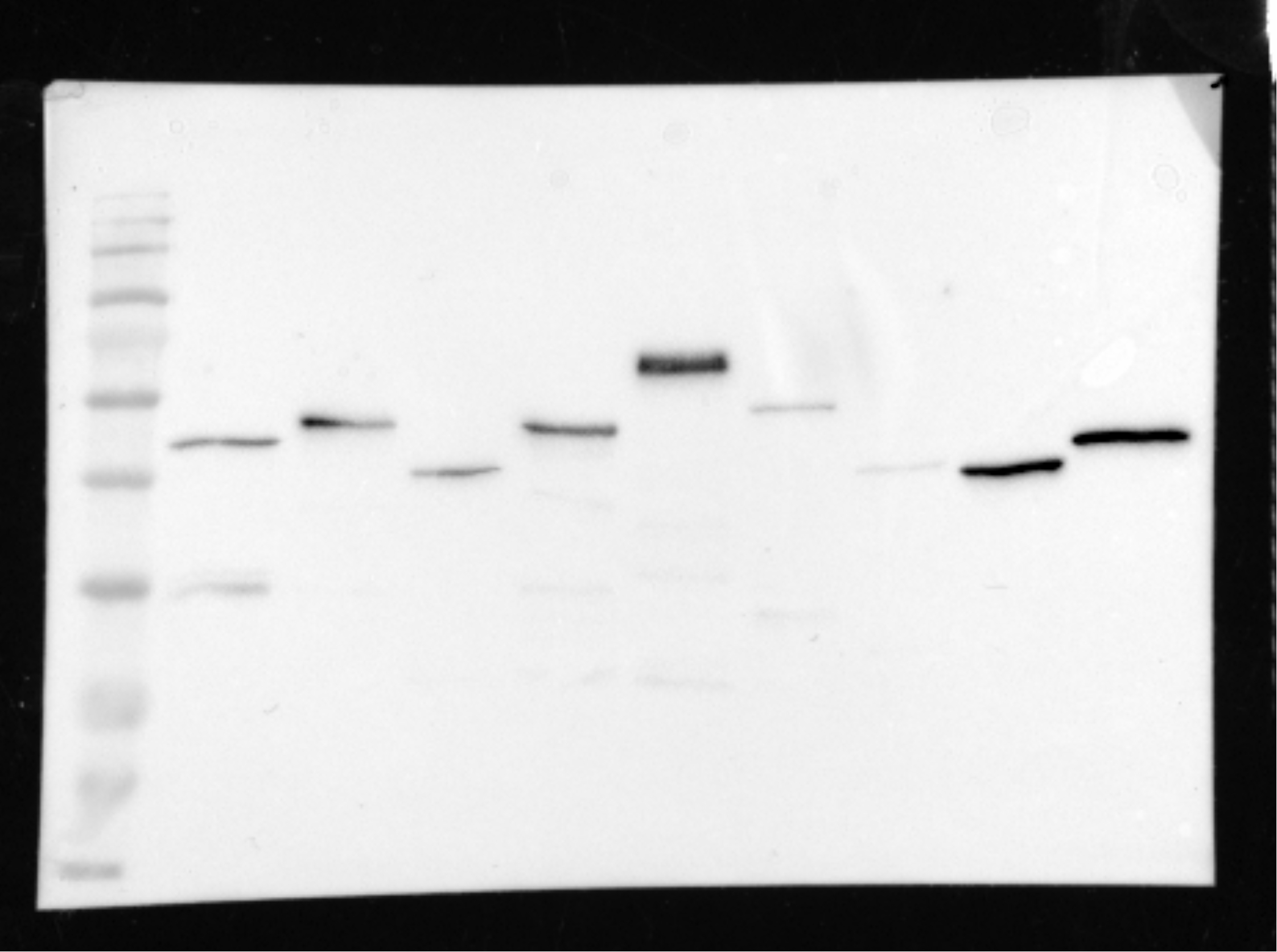

Supplement: Supplementary file 10 — Appendix Figures Source Data [file 44319_2024_203_MOESM10_ESM.zip › Appendix1_GST/Toprow/Rightmost/Lysate.jpg]

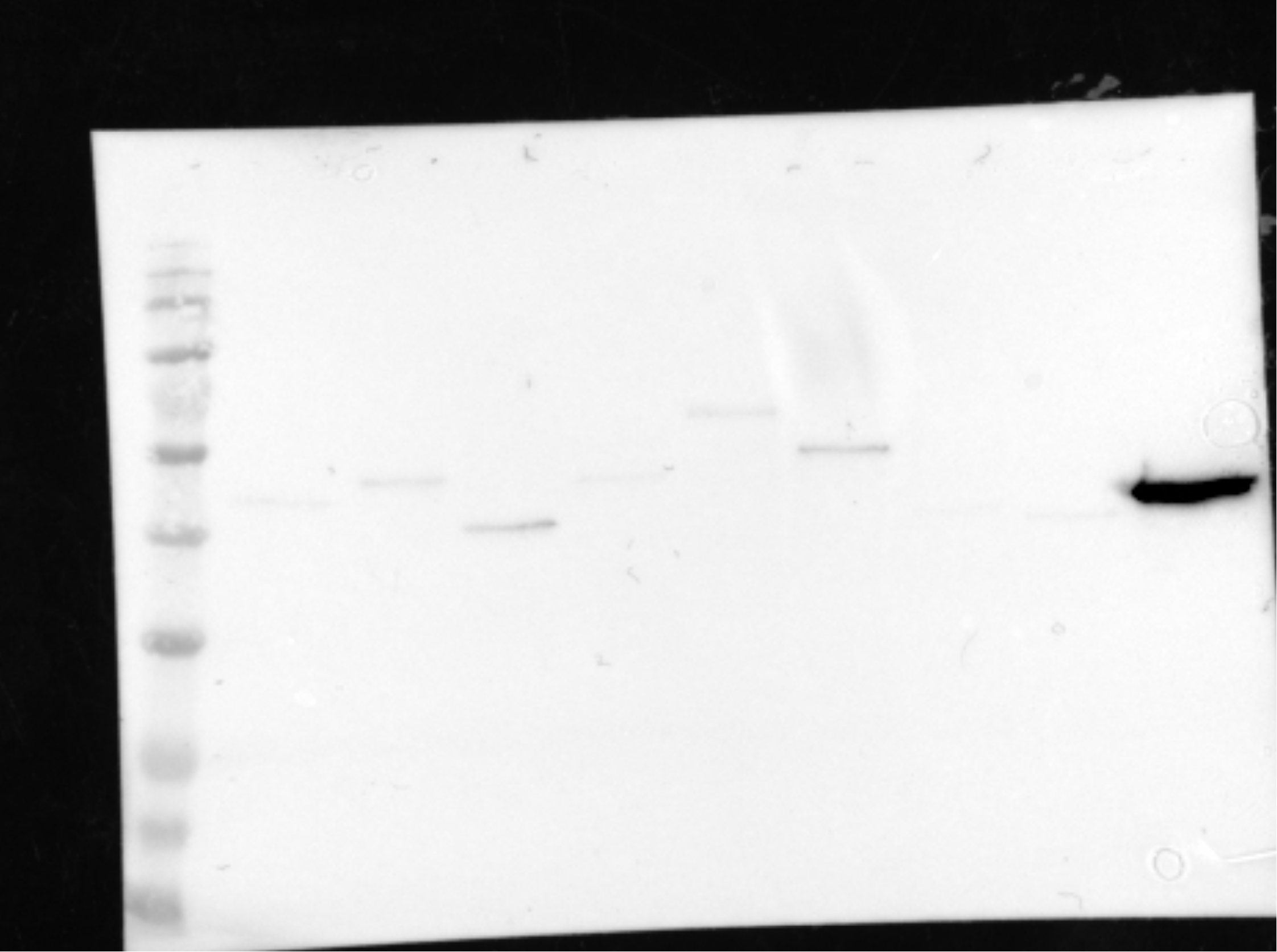

Supplement: Supplementary file 10 — Appendix Figures Source Data [file 44319_2024_203_MOESM10_ESM.zip › Appendix1_GST/Toprow/Rightmost/Pulldown.jpg]

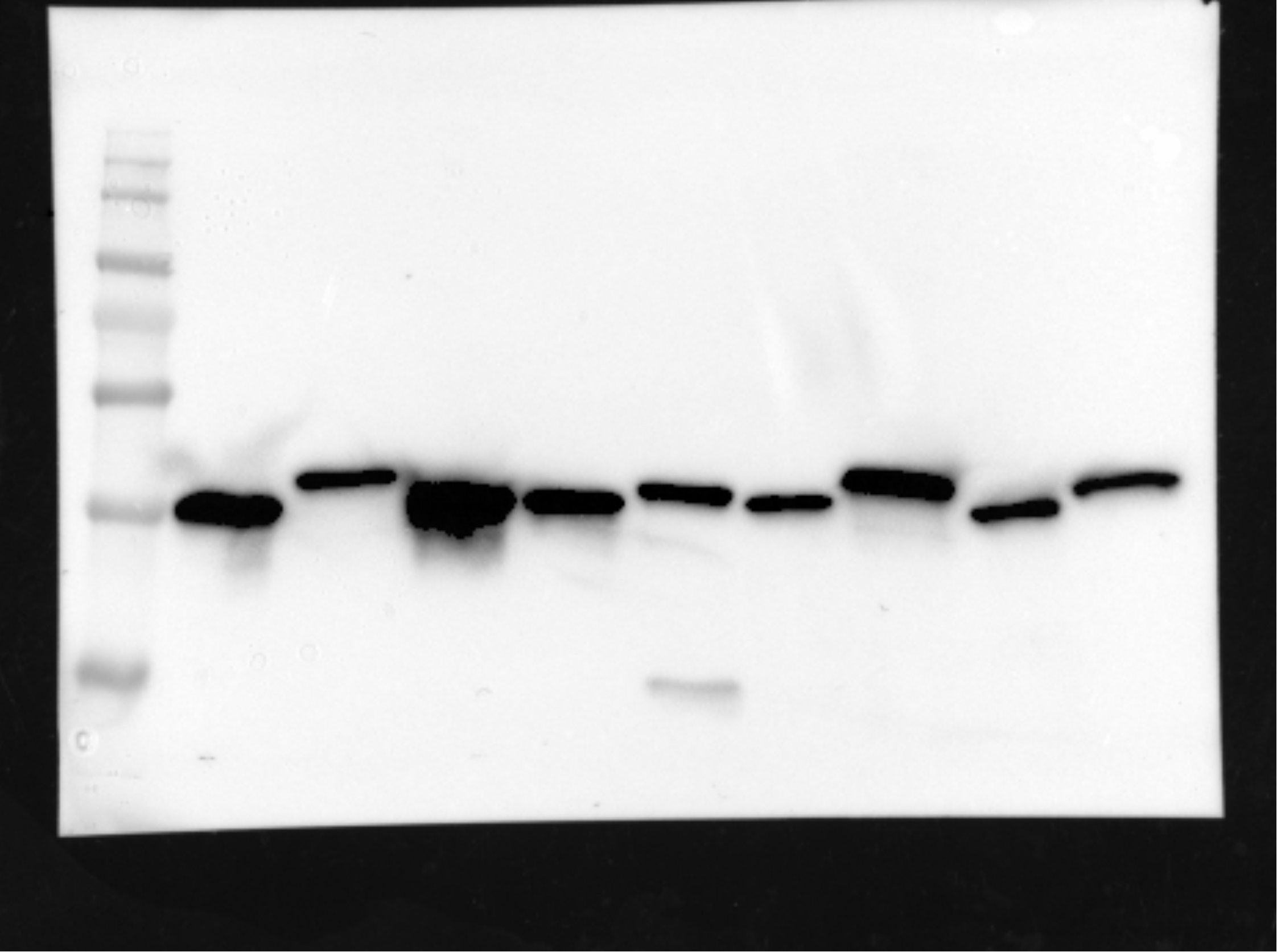

Supplement: Supplementary file 10 — Appendix Figures Source Data [file 44319_2024_203_MOESM10_ESM.zip › Appendix6_RASSF8/Fourthrow/Left/Lysate.jpg]

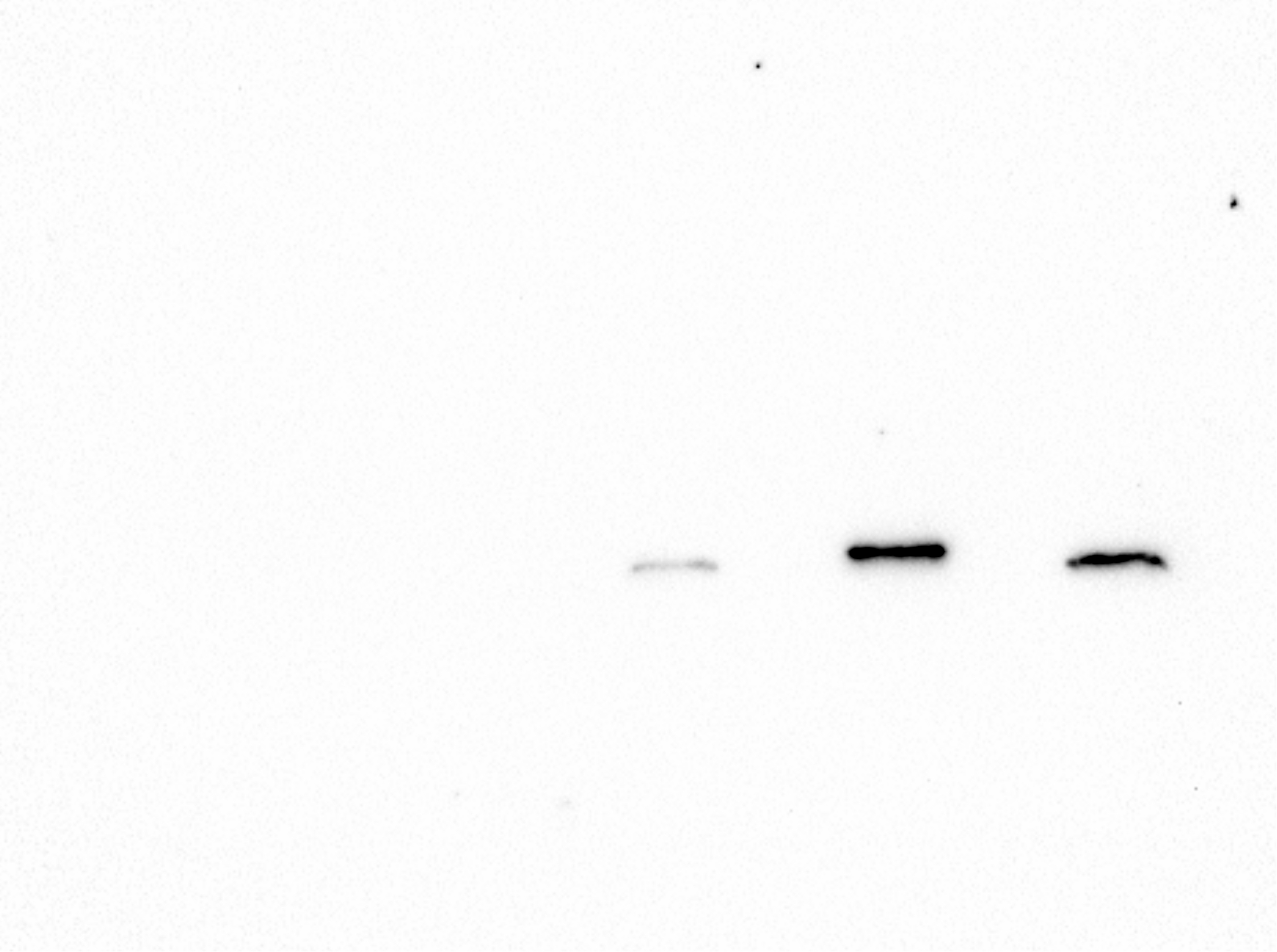

Supplement: Supplementary file 10 — Appendix Figures Source Data [file 44319_2024_203_MOESM10_ESM.zip › Appendix6_RASSF8/Fourthrow/Left/Pulldown.jpg]

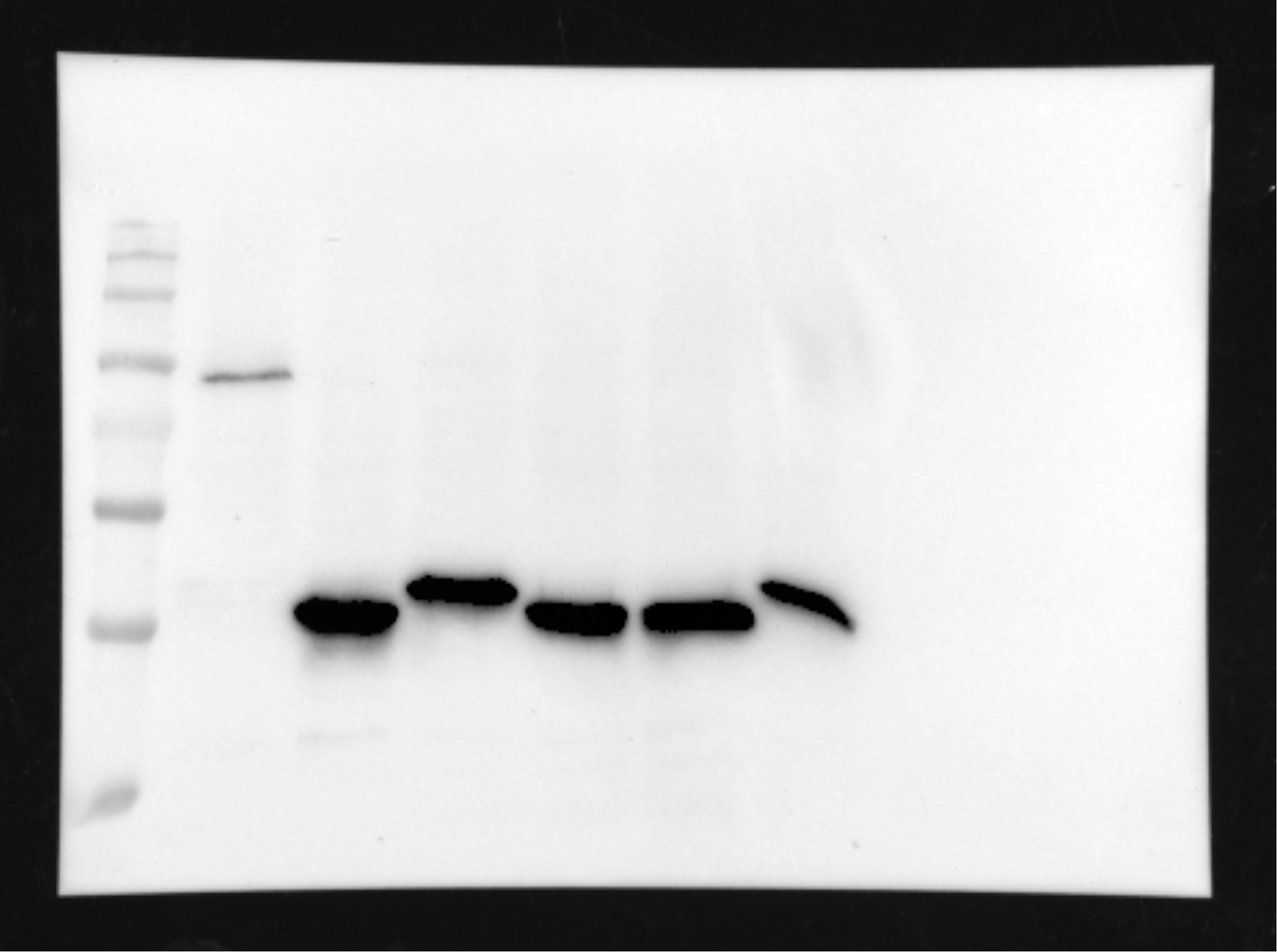

Supplement: Supplementary file 10 — Appendix Figures Source Data [file 44319_2024_203_MOESM10_ESM.zip › Appendix6_RASSF8/Fourthrow/Middle/Lysate.jpg]

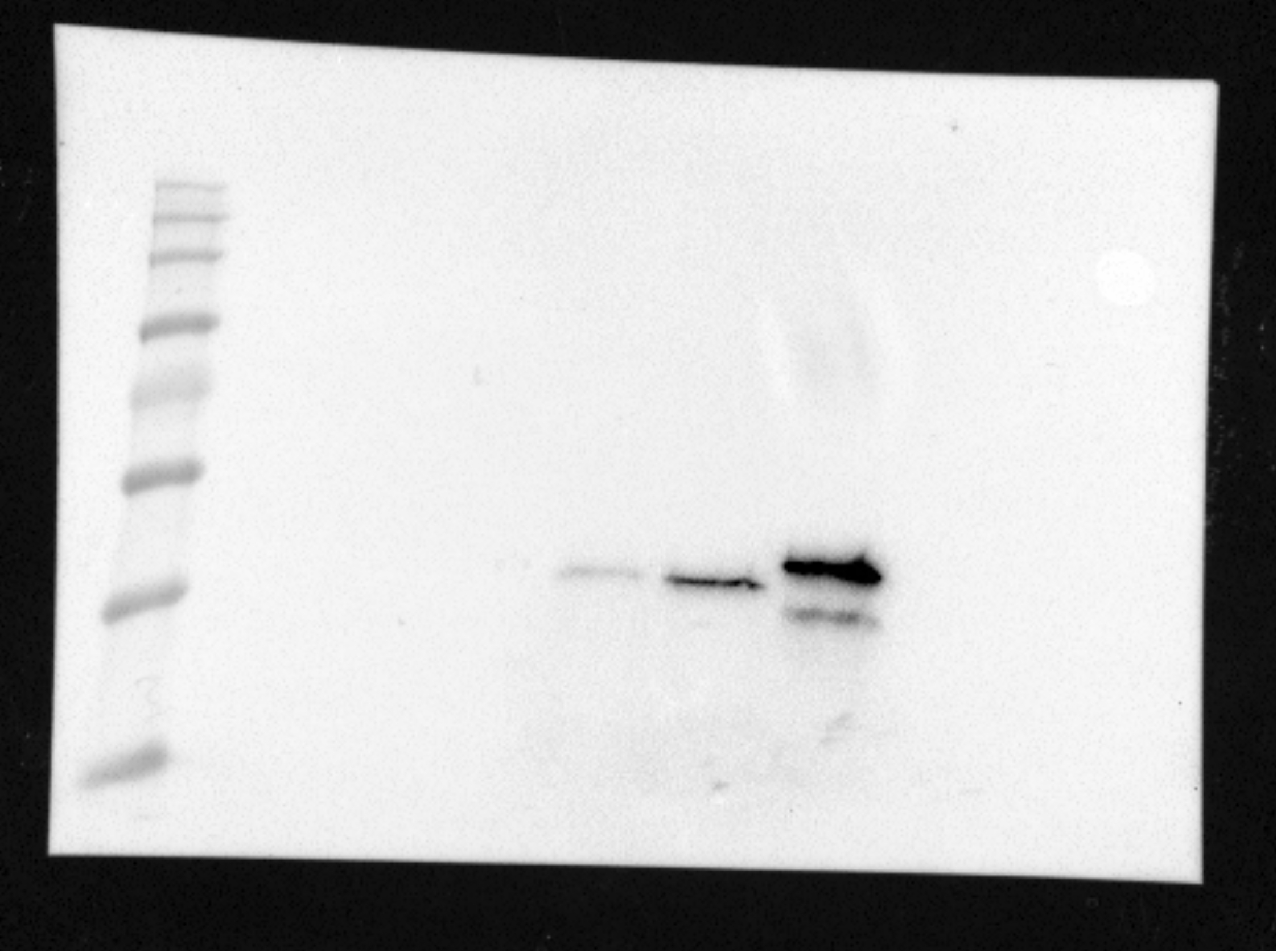

Supplement: Supplementary file 10 — Appendix Figures Source Data [file 44319_2024_203_MOESM10_ESM.zip › Appendix6_RASSF8/Fourthrow/Middle/Pulldown.jpg]

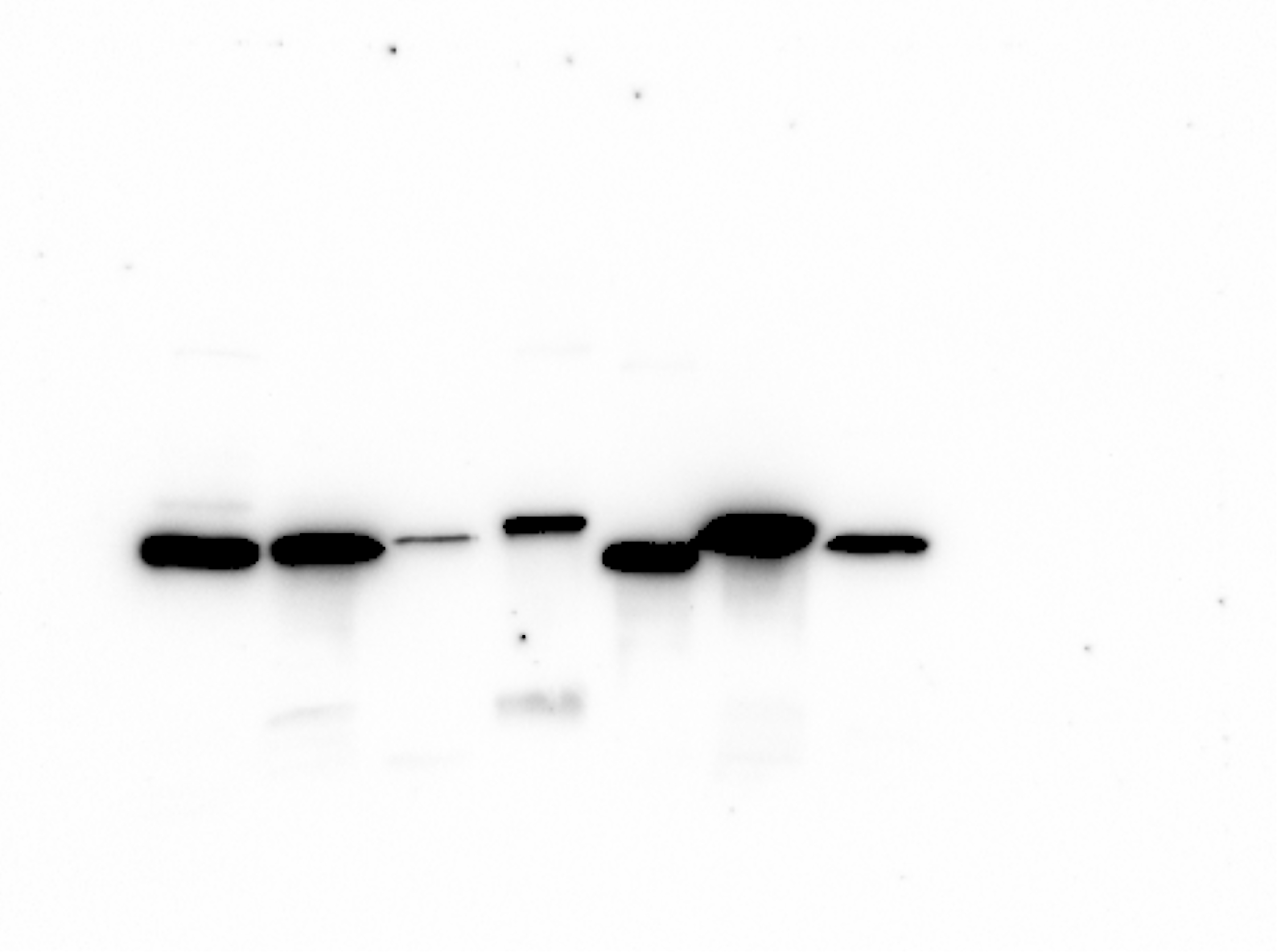

Supplement: Supplementary file 10 — Appendix Figures Source Data [file 44319_2024_203_MOESM10_ESM.zip › Appendix6_RASSF8/Fourthrow/Right/Lysate.jpg]

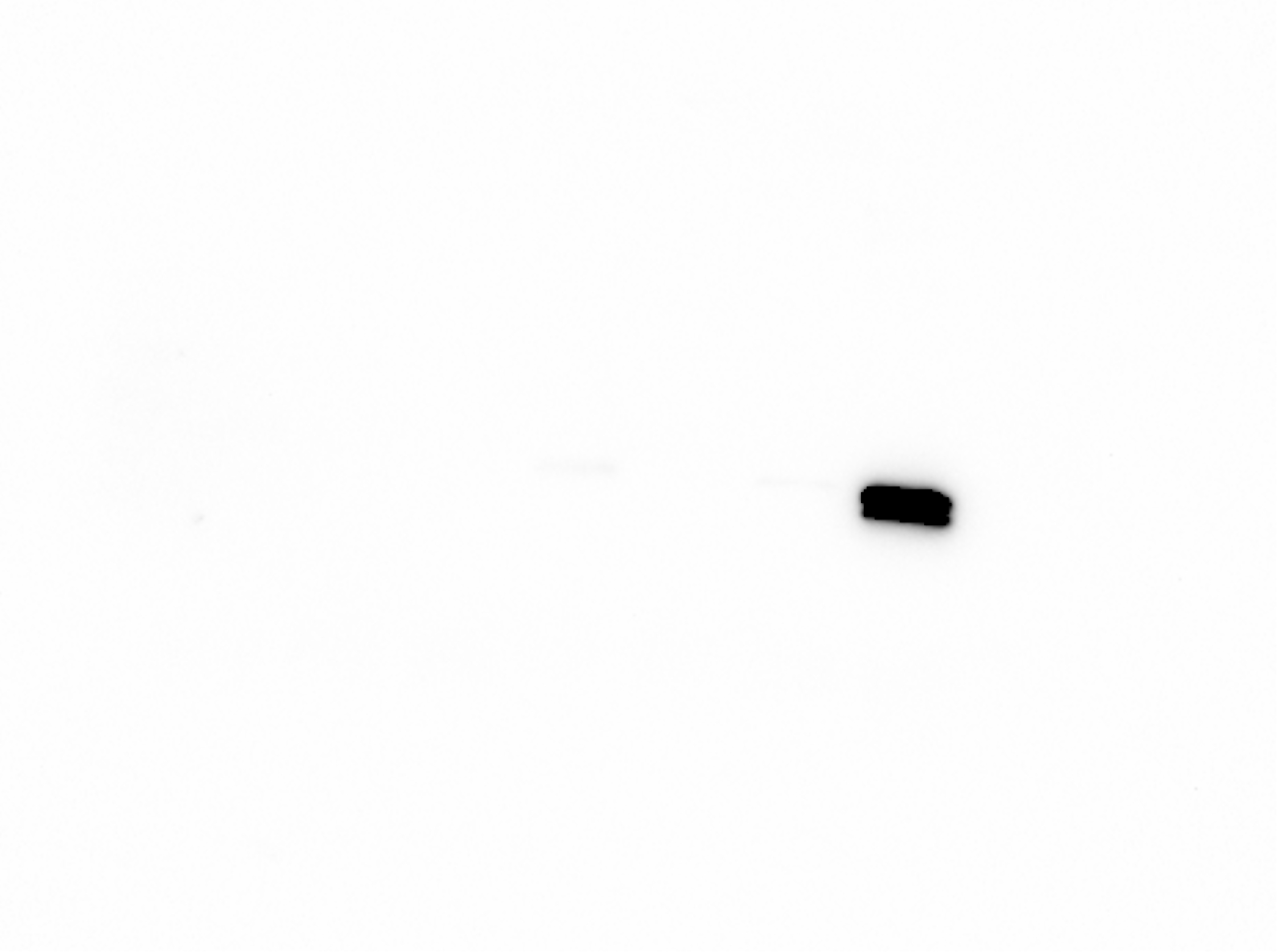

Supplement: Supplementary file 10 — Appendix Figures Source Data [file 44319_2024_203_MOESM10_ESM.zip › Appendix6_RASSF8/Fourthrow/Right/Pulldown.jpg]

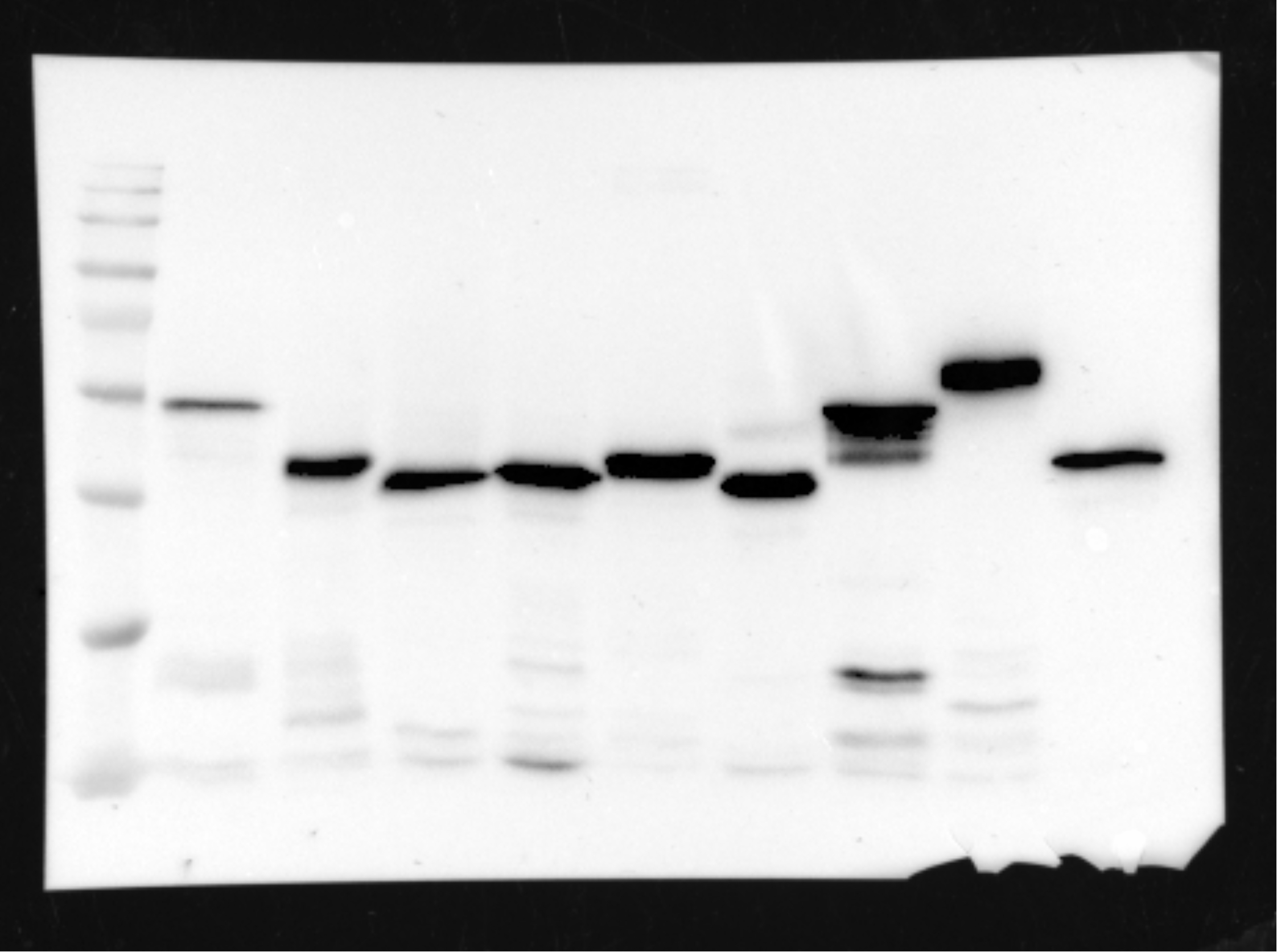

Supplement: Supplementary file 10 — Appendix Figures Source Data [file 44319_2024_203_MOESM10_ESM.zip › Appendix6_RASSF8/Secondrow/Left/Lysate.jpg]

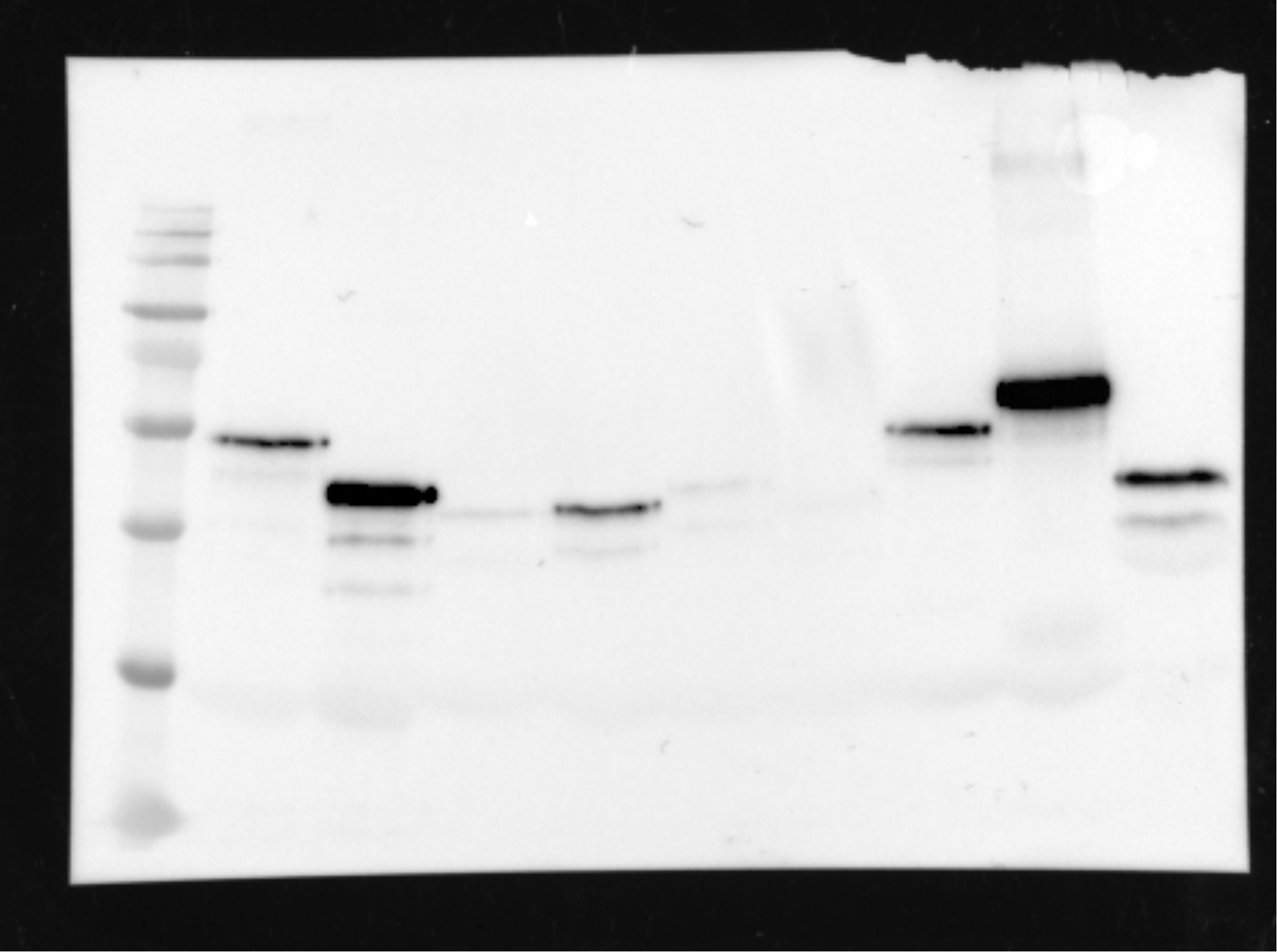

Supplement: Supplementary file 10 — Appendix Figures Source Data [file 44319_2024_203_MOESM10_ESM.zip › Appendix6_RASSF8/Secondrow/Left/Pulldown.jpg]

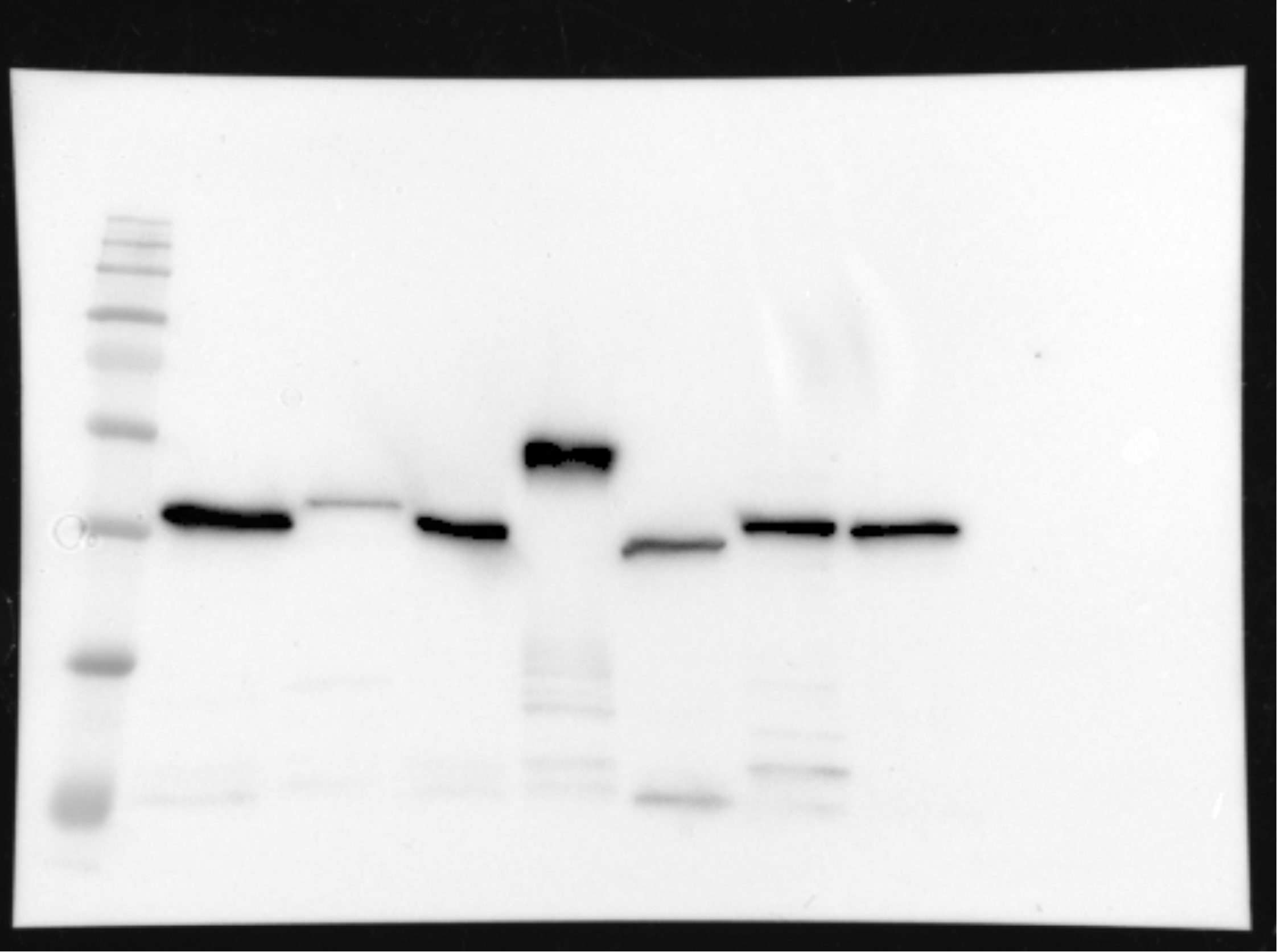

Supplement: Supplementary file 10 — Appendix Figures Source Data [file 44319_2024_203_MOESM10_ESM.zip › Appendix6_RASSF8/Secondrow/Middle/Lysate.jpg]

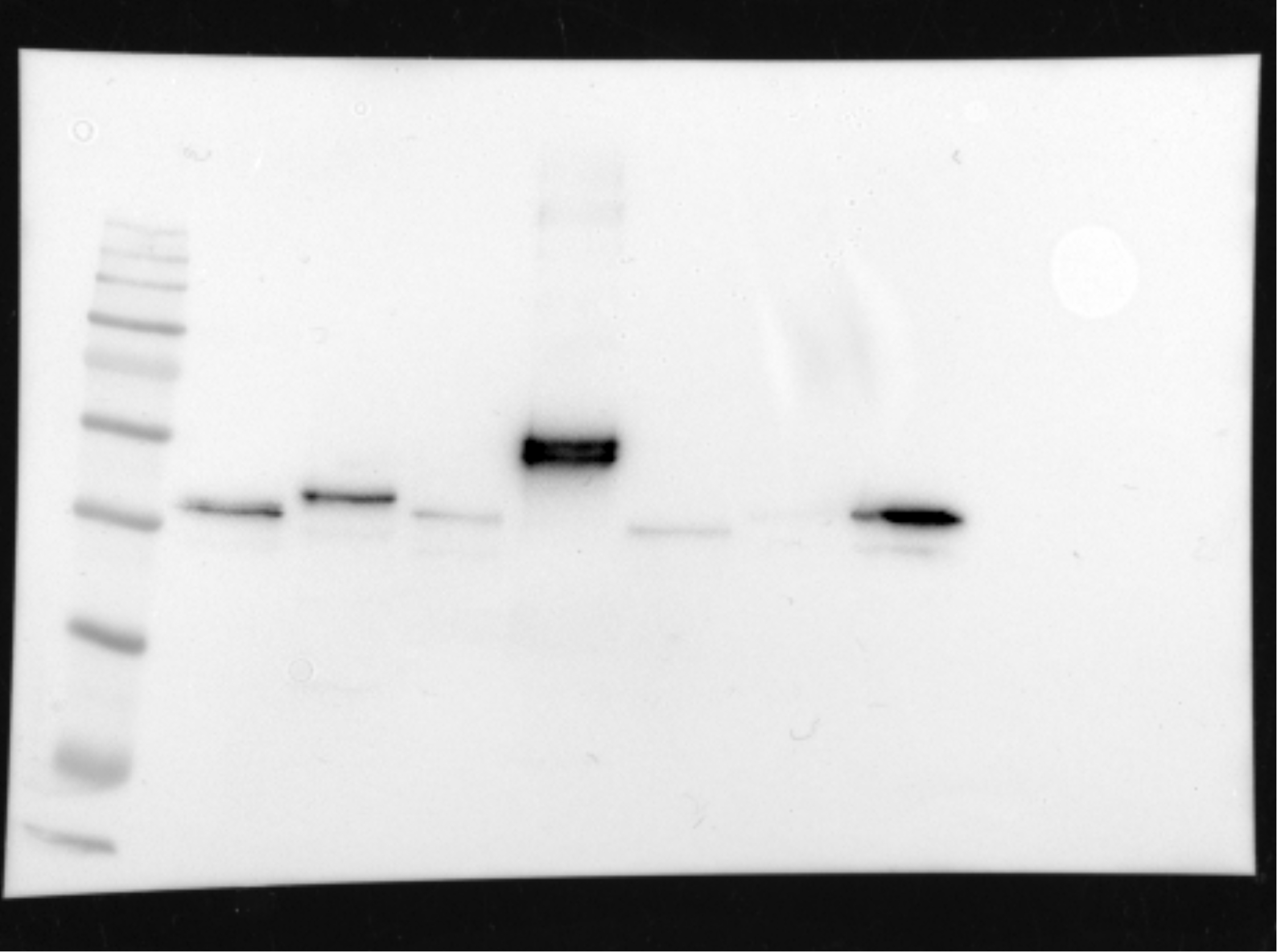

Supplement: Supplementary file 10 — Appendix Figures Source Data [file 44319_2024_203_MOESM10_ESM.zip › Appendix6_RASSF8/Secondrow/Middle/Pulldown.jpg]

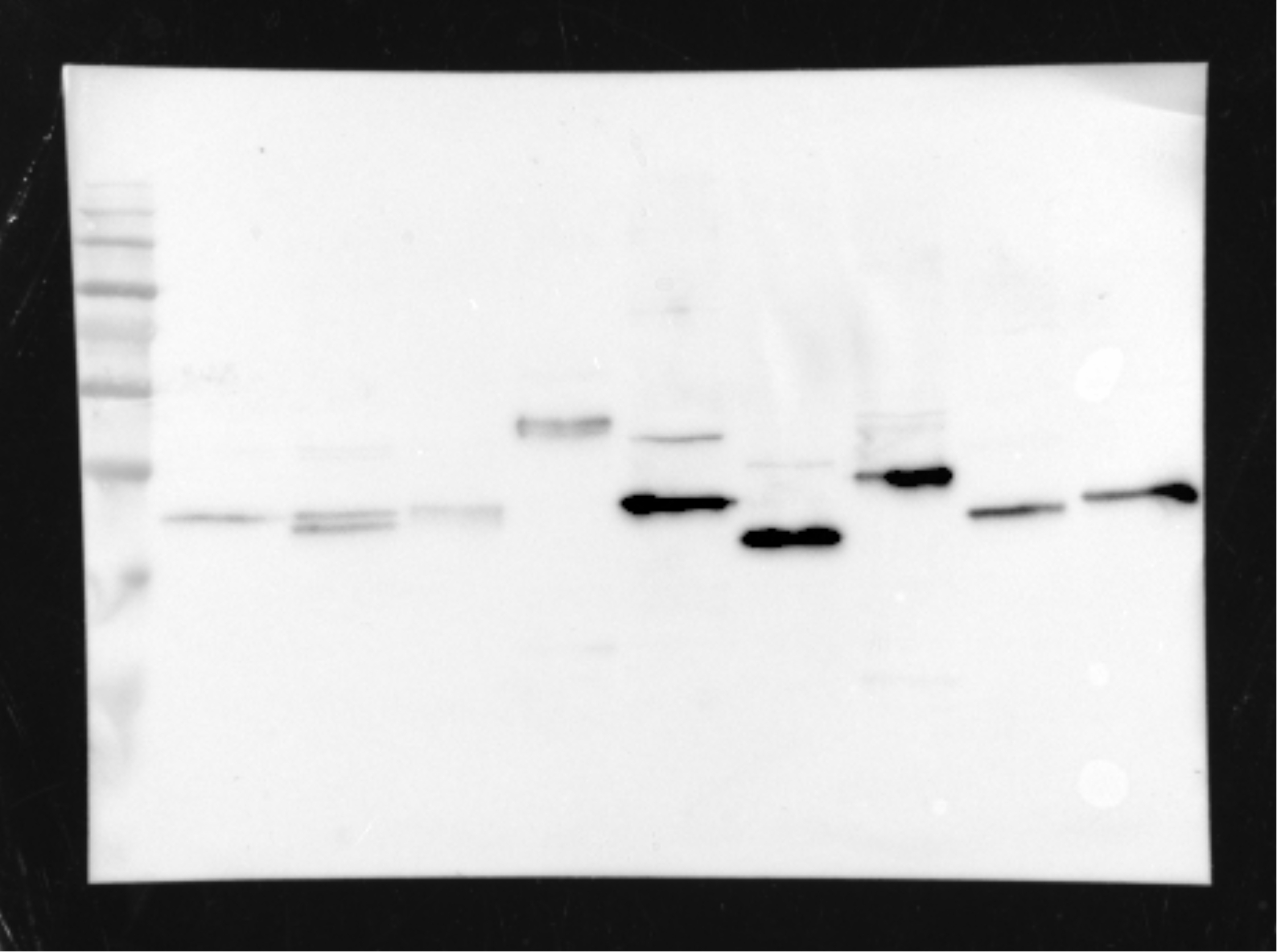

Supplement: Supplementary file 10 — Appendix Figures Source Data [file 44319_2024_203_MOESM10_ESM.zip › Appendix6_RASSF8/Secondrow/Right/Lysate.jpg]

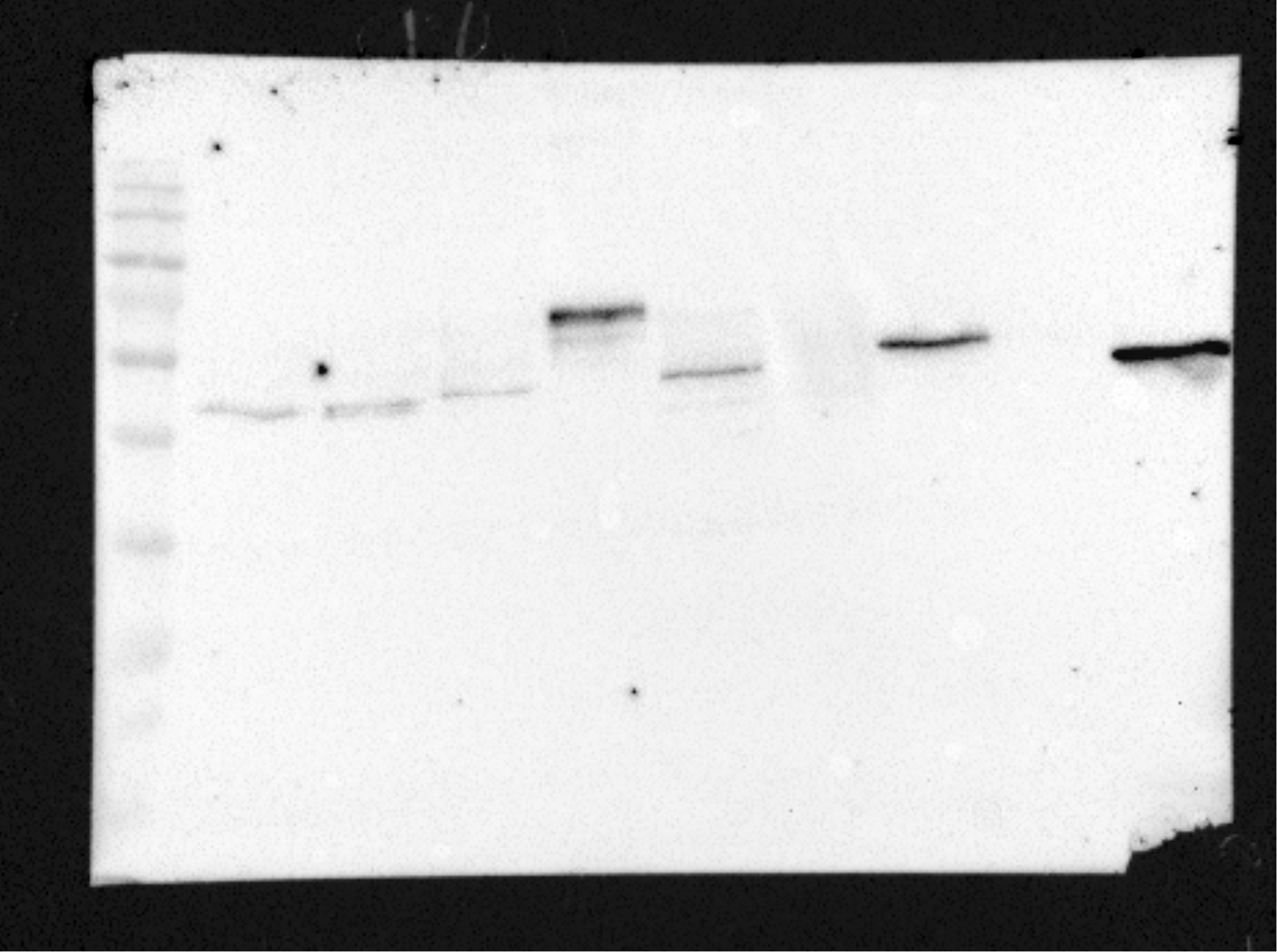

Supplement: Supplementary file 10 — Appendix Figures Source Data [file 44319_2024_203_MOESM10_ESM.zip › Appendix6_RASSF8/Secondrow/Right/Pulldown.jpg]

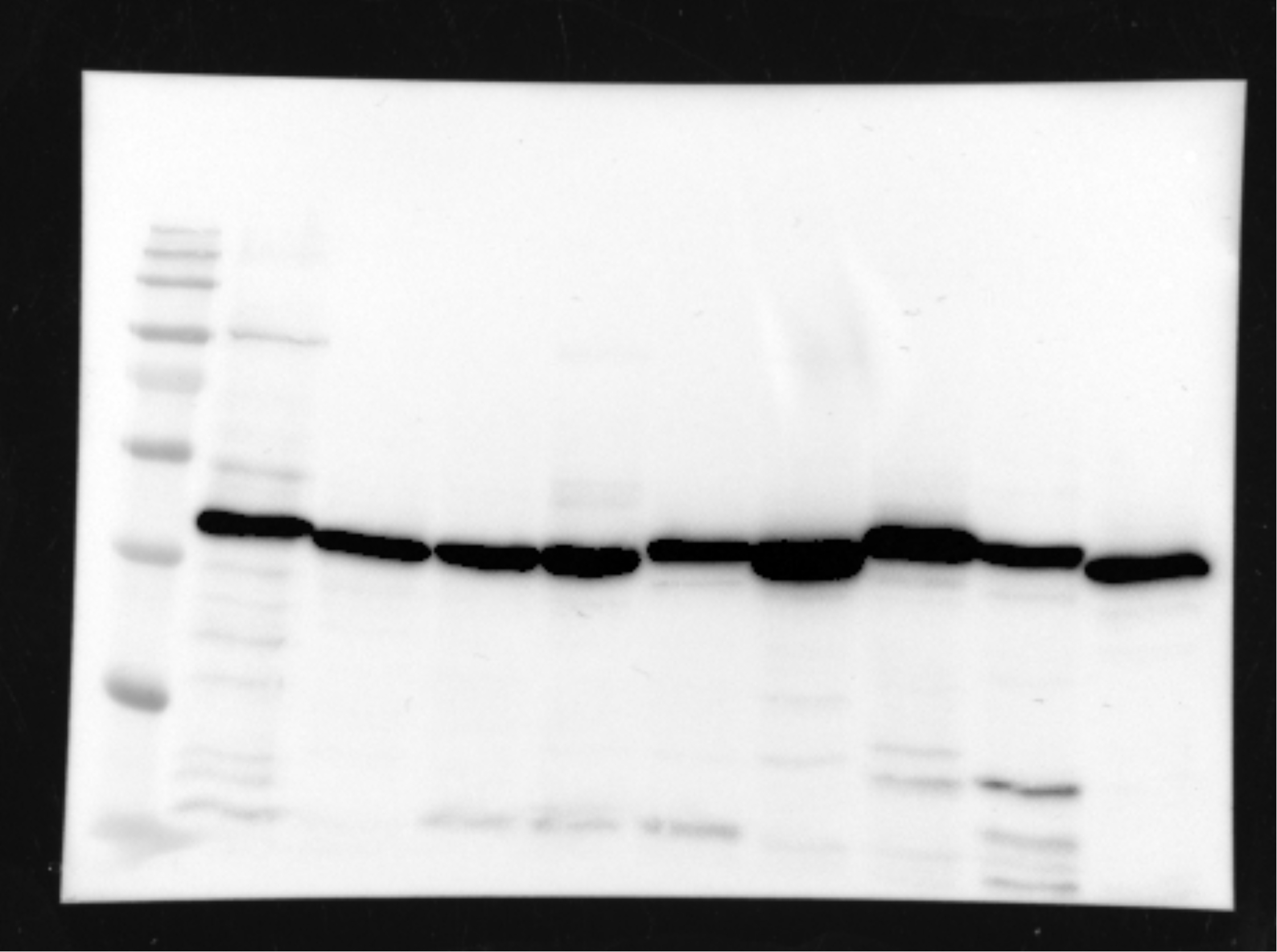

Supplement: Supplementary file 10 — Appendix Figures Source Data [file 44319_2024_203_MOESM10_ESM.zip › Appendix6_RASSF8/Thirdrow/Left/Lysate.jpg]

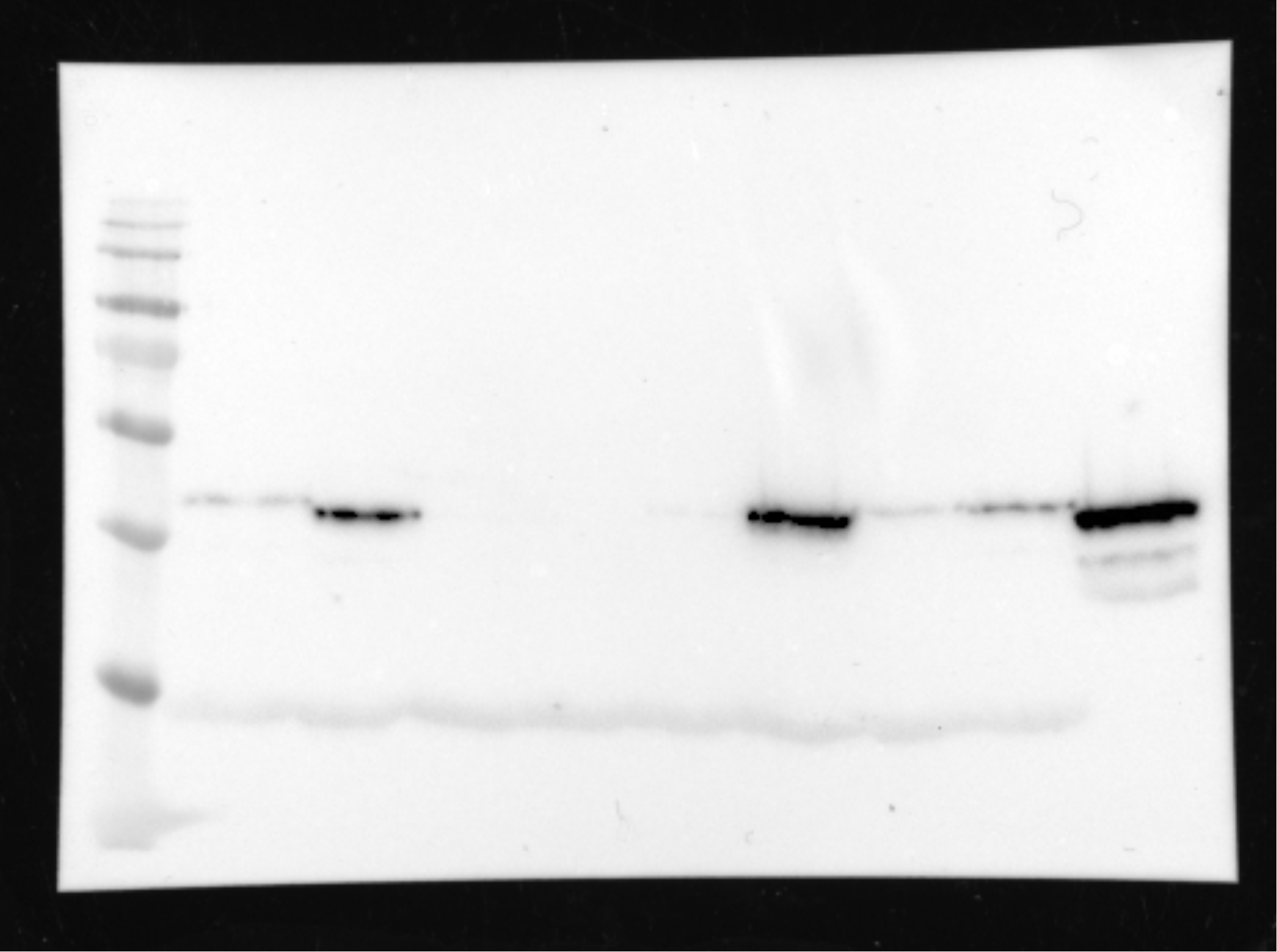

Supplement: Supplementary file 10 — Appendix Figures Source Data [file 44319_2024_203_MOESM10_ESM.zip › Appendix6_RASSF8/Thirdrow/Left/Pulldown.jpg]

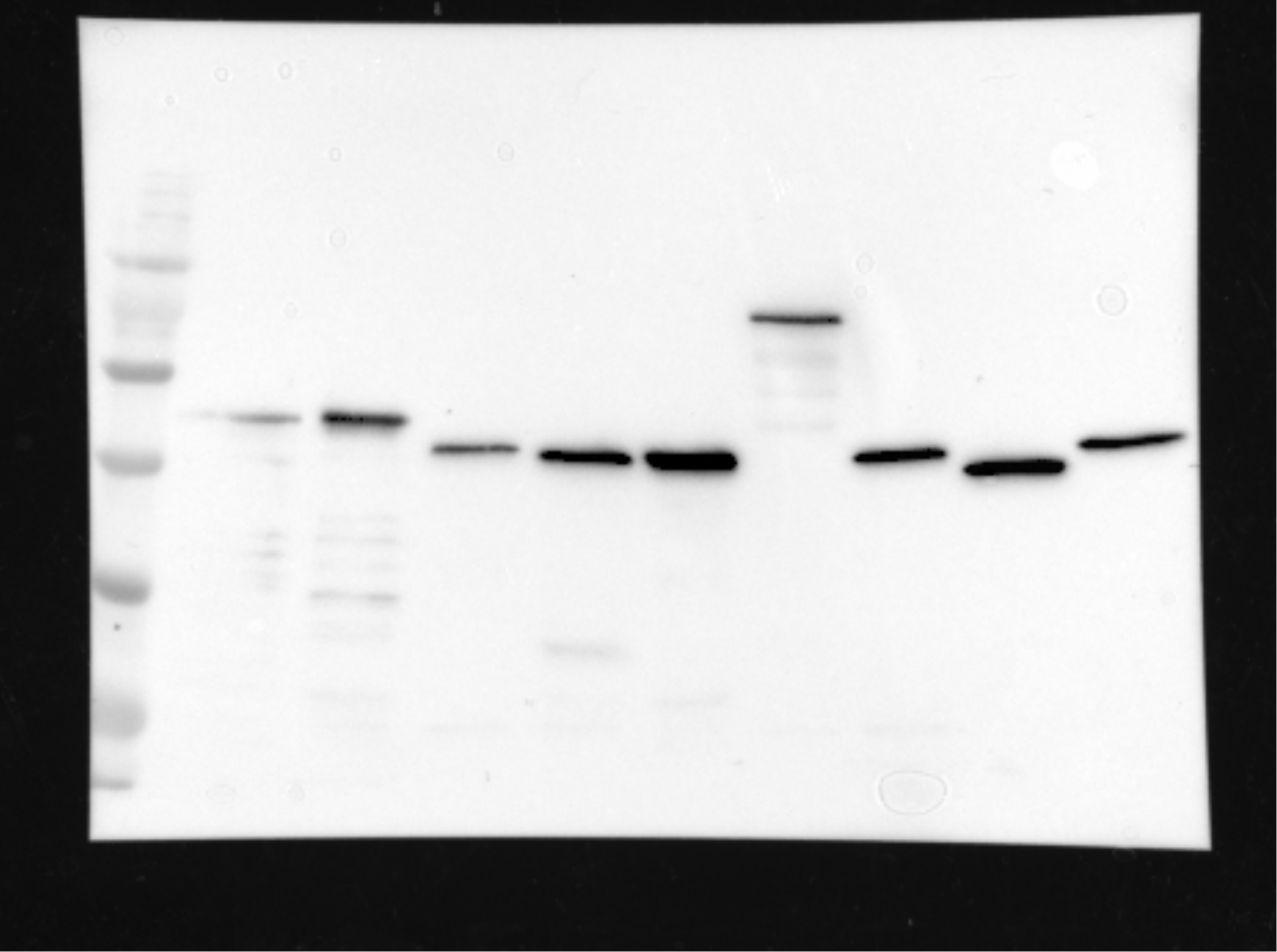

Supplement: Supplementary file 10 — Appendix Figures Source Data [file 44319_2024_203_MOESM10_ESM.zip › Appendix6_RASSF8/Thirdrow/Middle/Lysate.jpg]

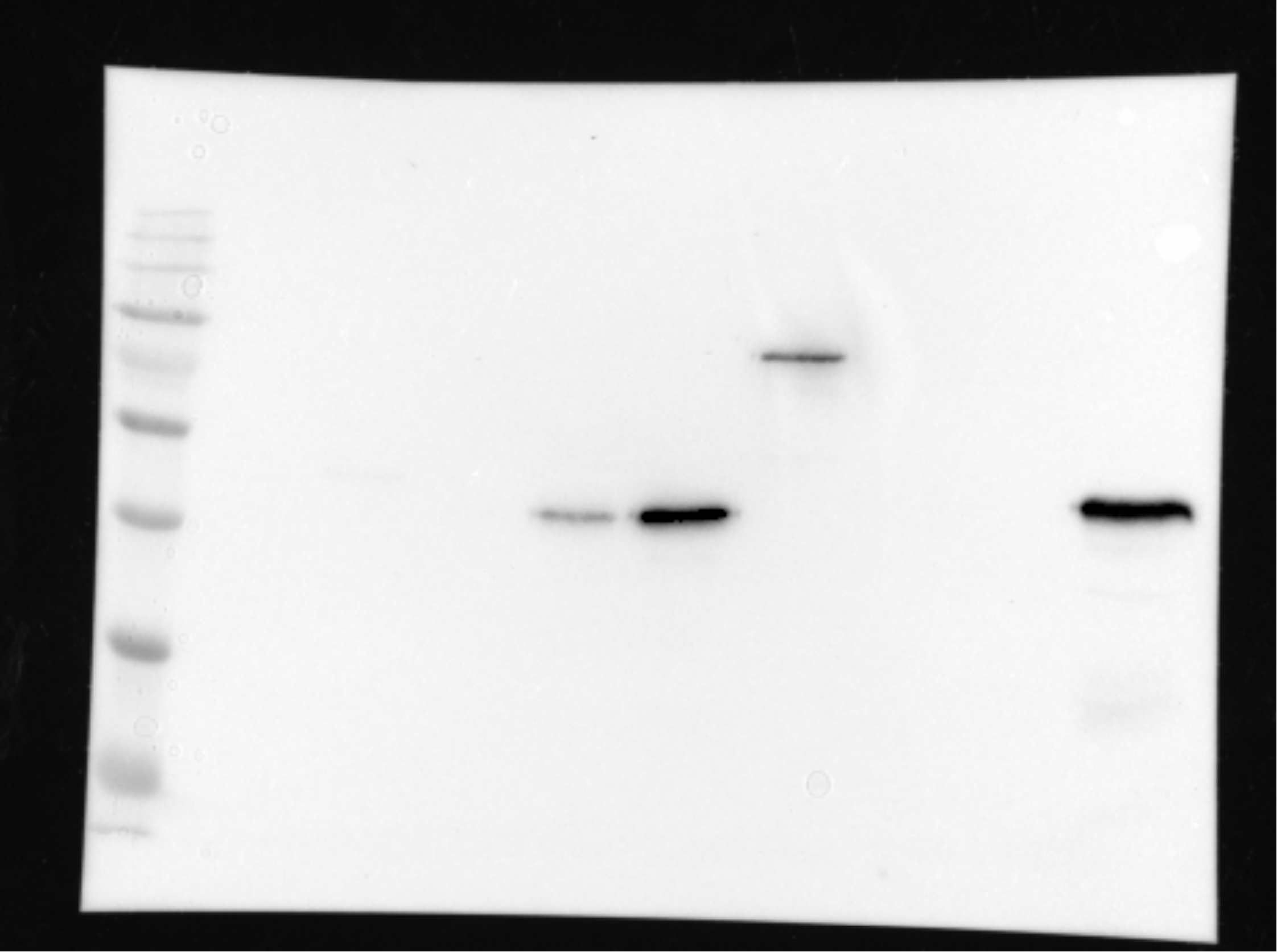

Supplement: Supplementary file 10 — Appendix Figures Source Data [file 44319_2024_203_MOESM10_ESM.zip › Appendix6_RASSF8/Thirdrow/Middle/Pulldown.jpg]

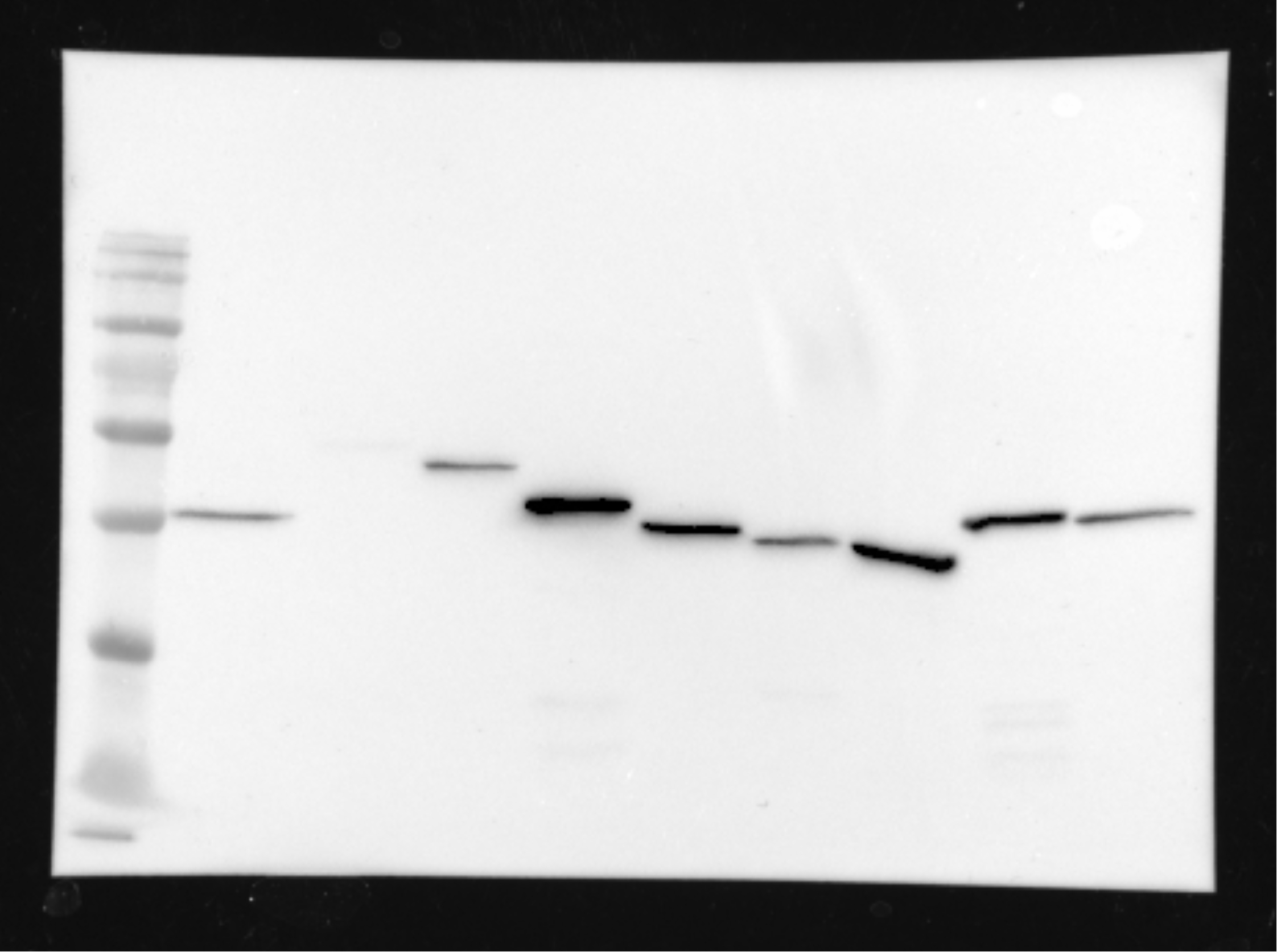

Supplement: Supplementary file 10 — Appendix Figures Source Data [file 44319_2024_203_MOESM10_ESM.zip › Appendix6_RASSF8/Thirdrow/Right/Lysate.jpg]

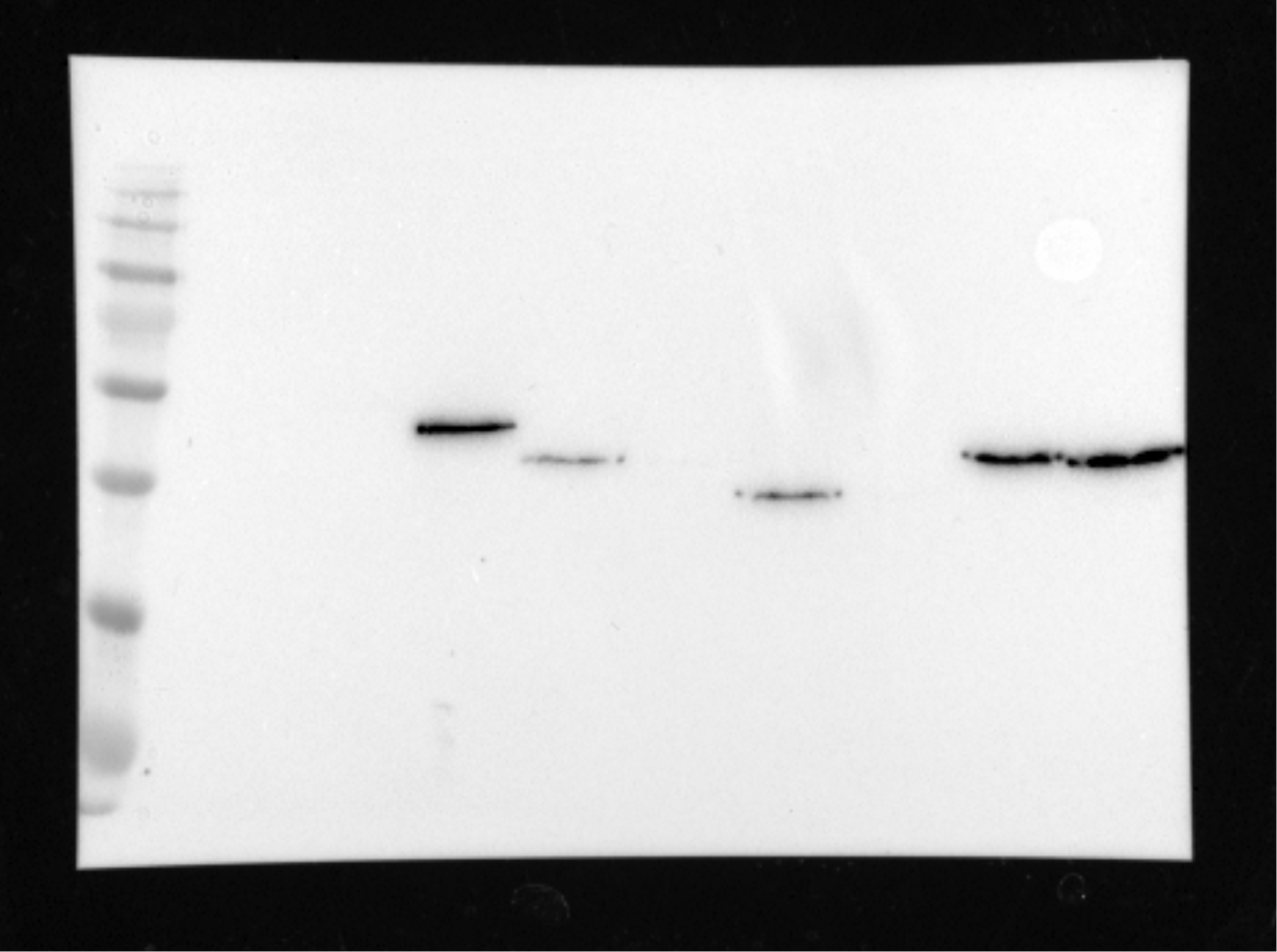

Supplement: Supplementary file 10 — Appendix Figures Source Data [file 44319_2024_203_MOESM10_ESM.zip › Appendix6_RASSF8/Thirdrow/Right/Pulldown.jpg]

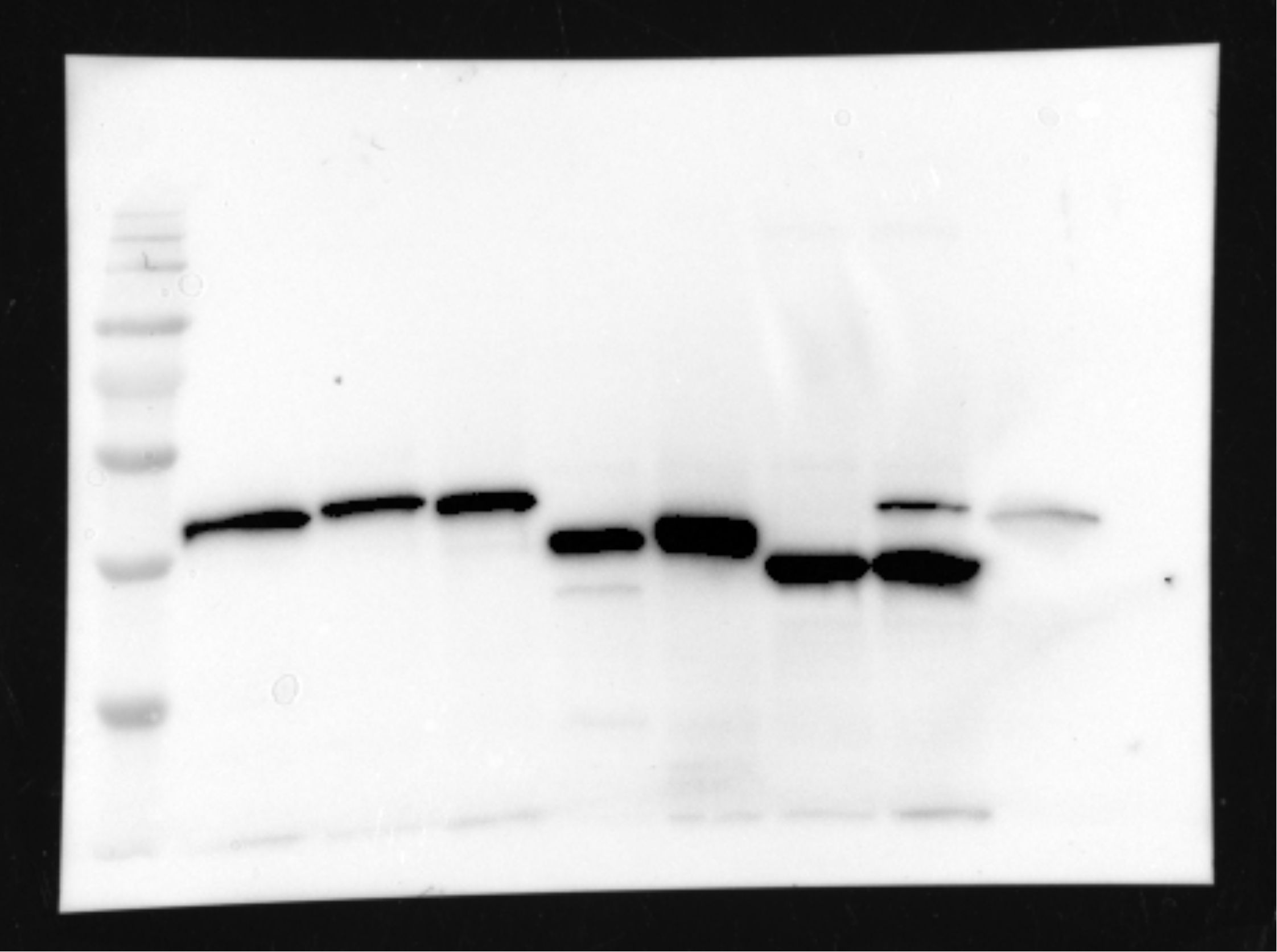

Supplement: Supplementary file 10 — Appendix Figures Source Data [file 44319_2024_203_MOESM10_ESM.zip › Appendix6_RASSF8/Toprow/Left/Lysate.jpg]

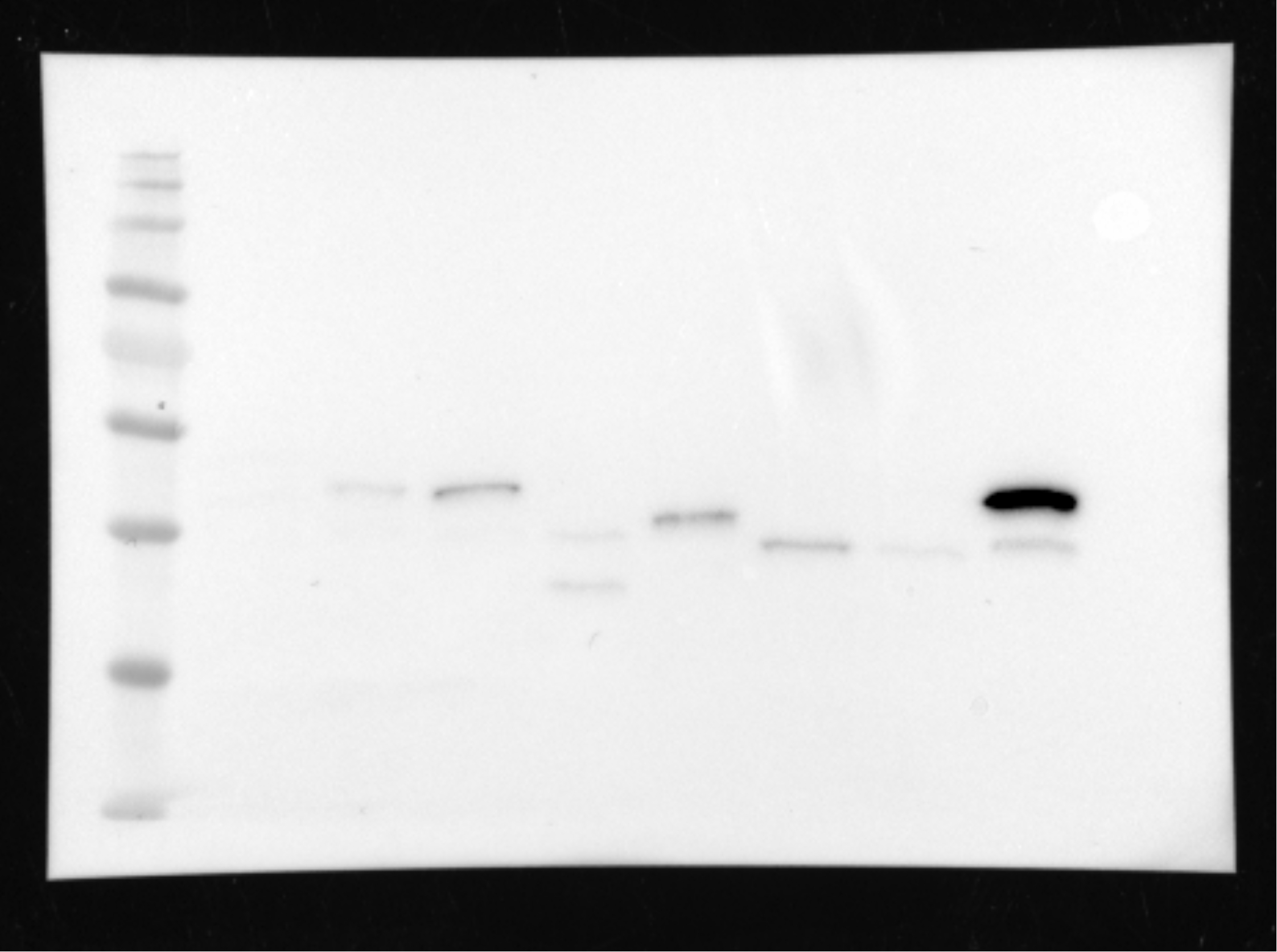

Supplement: Supplementary file 10 — Appendix Figures Source Data [file 44319_2024_203_MOESM10_ESM.zip › Appendix6_RASSF8/Toprow/Left/Pulldown.jpg]

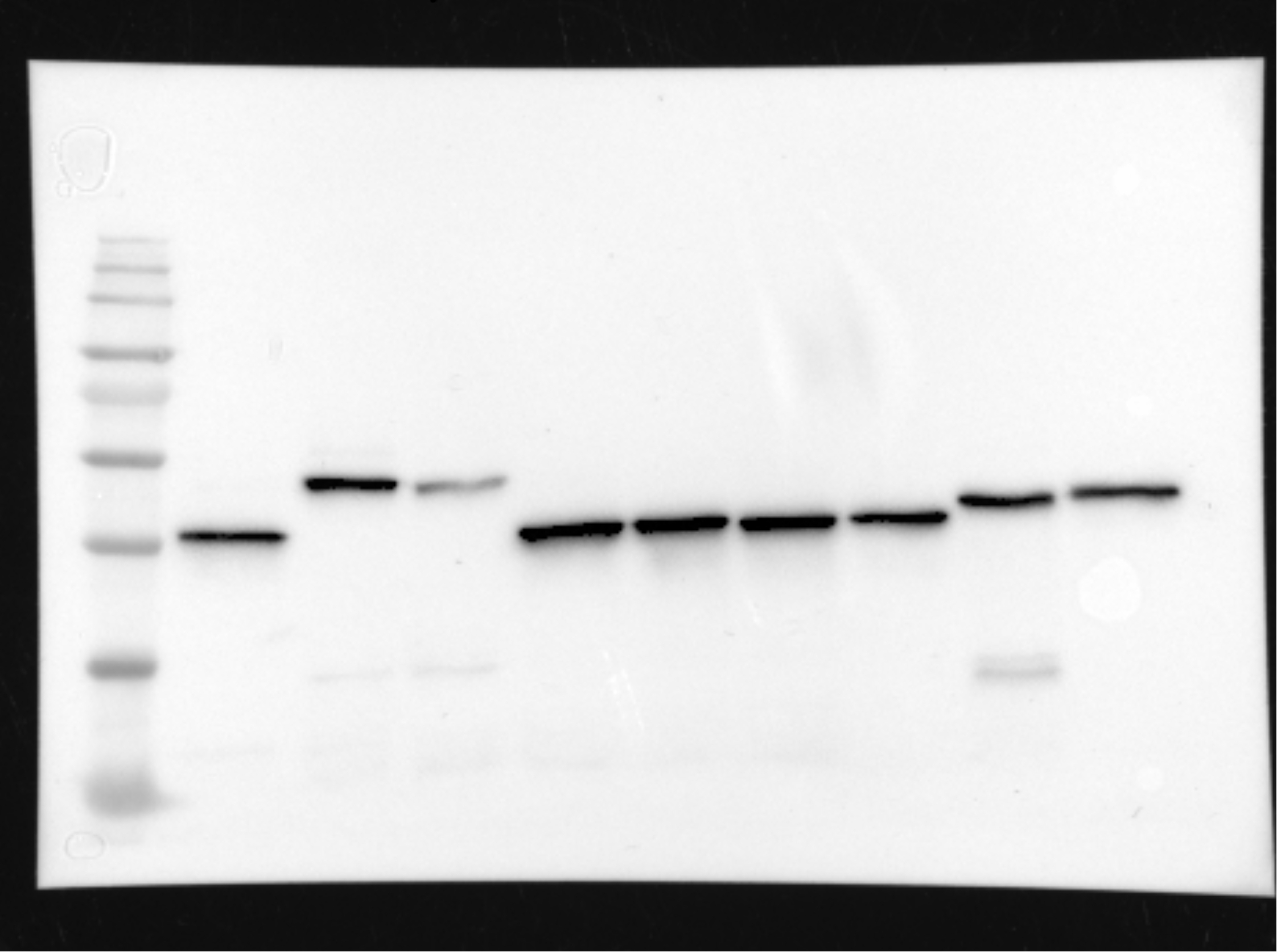

Supplement: Supplementary file 10 — Appendix Figures Source Data [file 44319_2024_203_MOESM10_ESM.zip › Appendix6_RASSF8/Toprow/Middle/Lysate.jpg]

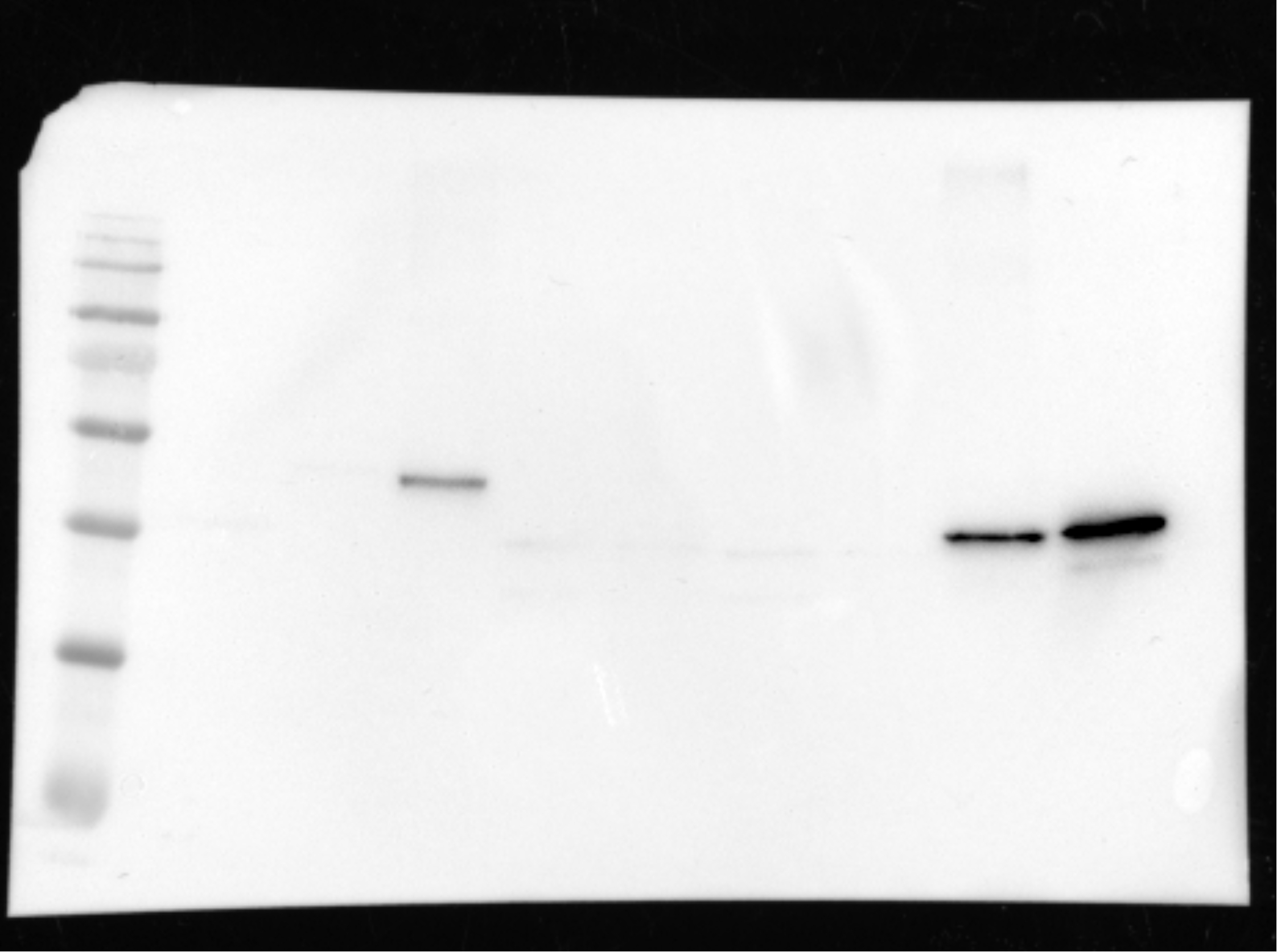

Supplement: Supplementary file 10 — Appendix Figures Source Data [file 44319_2024_203_MOESM10_ESM.zip › Appendix6_RASSF8/Toprow/Middle/Pulldown.jpg]

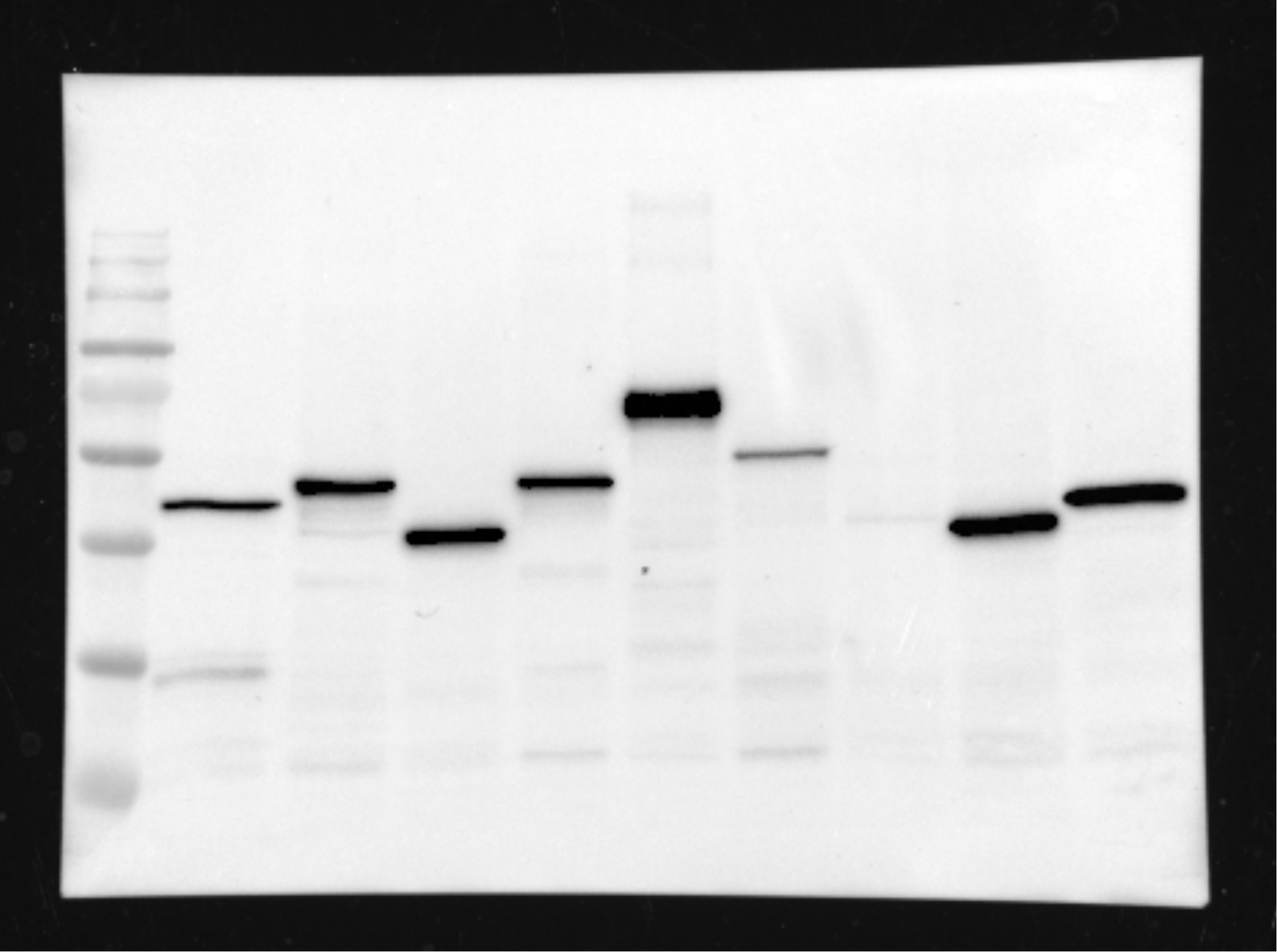

Supplement: Supplementary file 10 — Appendix Figures Source Data [file 44319_2024_203_MOESM10_ESM.zip › Appendix6_RASSF8/Toprow/Right/Lysate.jpg]

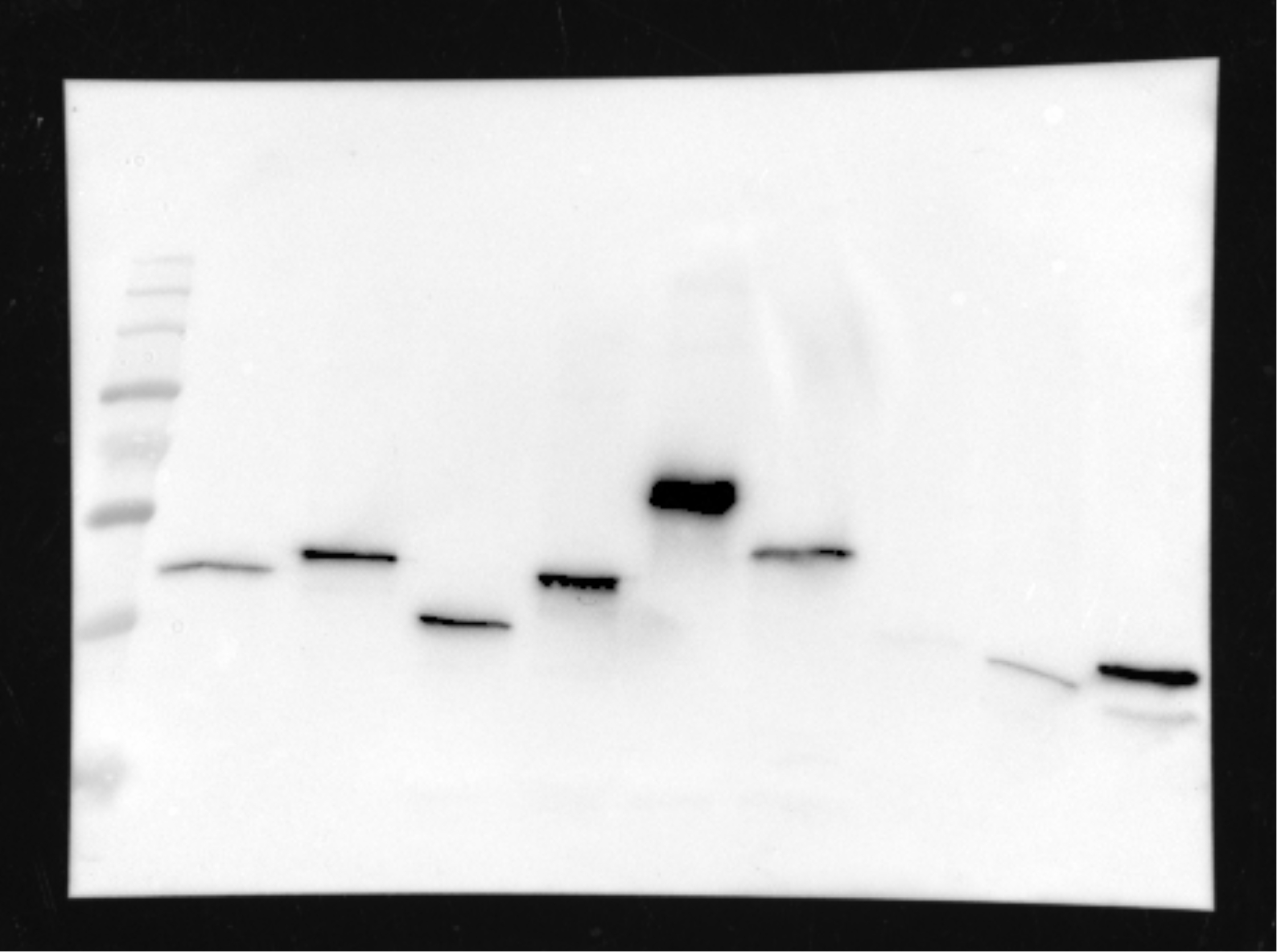

Supplement: Supplementary file 10 — Appendix Figures Source Data [file 44319_2024_203_MOESM10_ESM.zip › Appendix6_RASSF8/Toprow/Right/Pulldown.jpg]

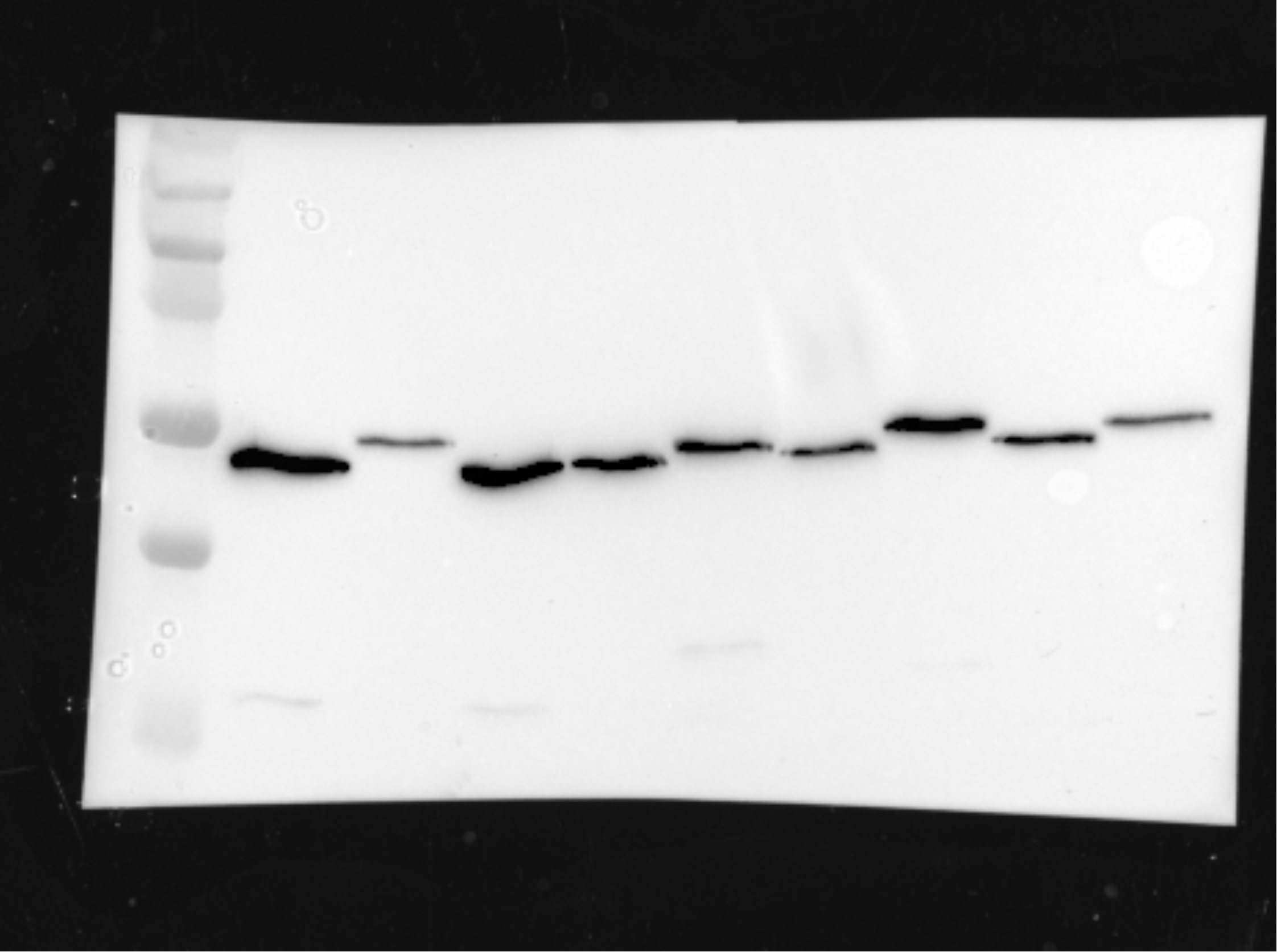

Supplement: Supplementary file 10 — Appendix Figures Source Data [file 44319_2024_203_MOESM10_ESM.zip › Appendix5_RASSF4/Fourthrow/Left/Lysate.jpg]

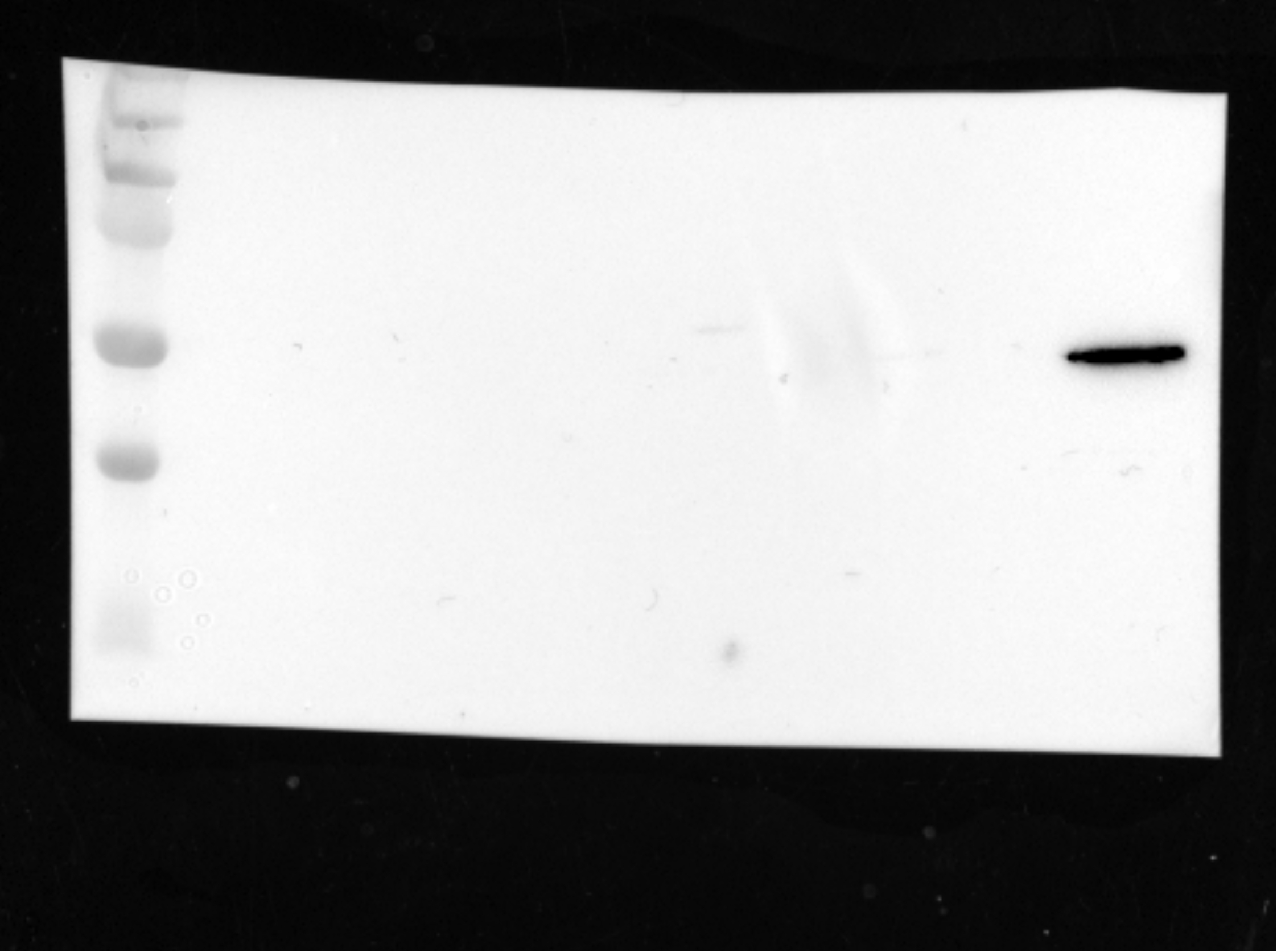

Supplement: Supplementary file 10 — Appendix Figures Source Data [file 44319_2024_203_MOESM10_ESM.zip › Appendix5_RASSF4/Fourthrow/Left/Pulldown.jpg]

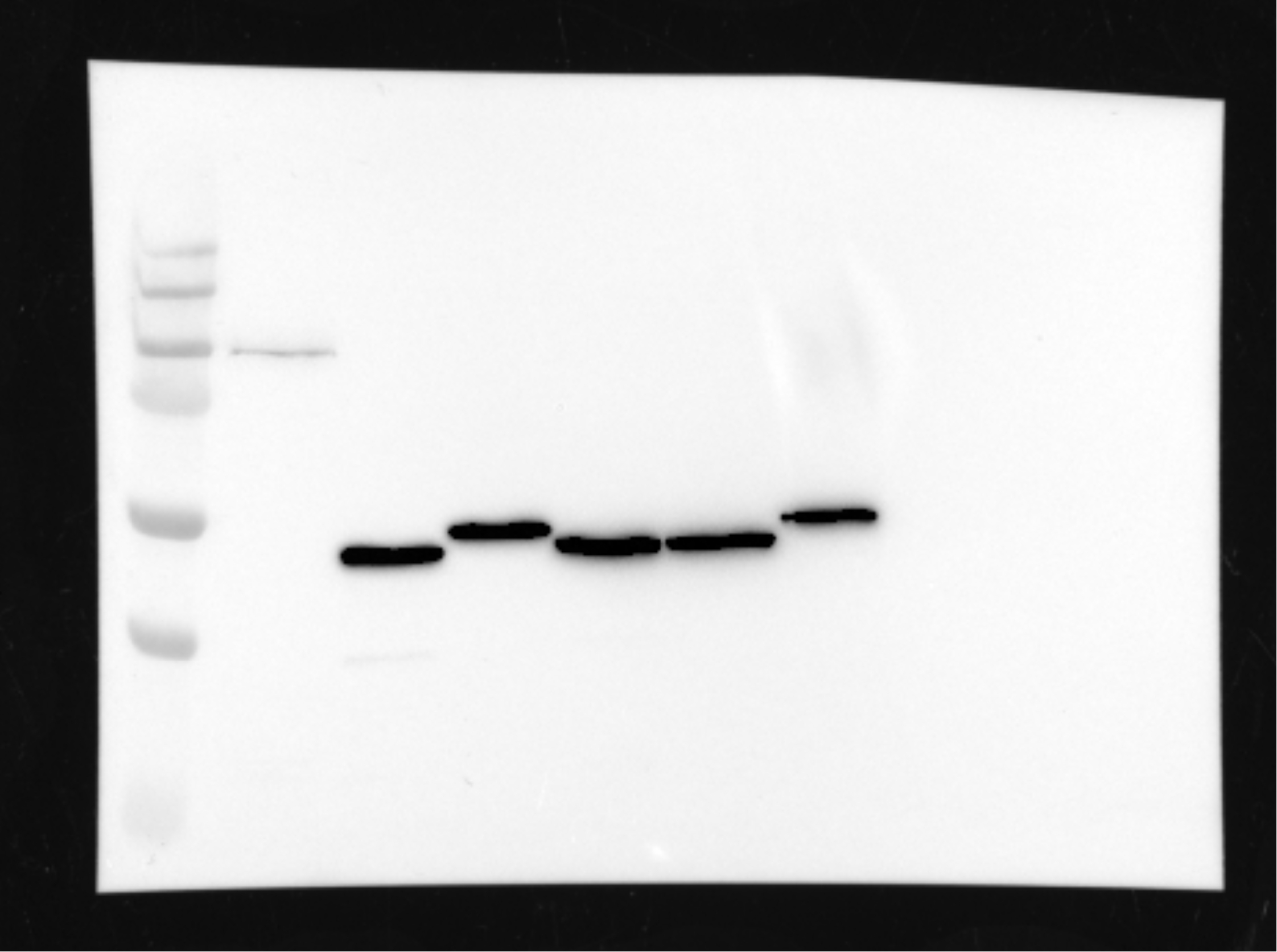

Supplement: Supplementary file 10 — Appendix Figures Source Data [file 44319_2024_203_MOESM10_ESM.zip › Appendix5_RASSF4/Fourthrow/Middle/Lysate.jpg]

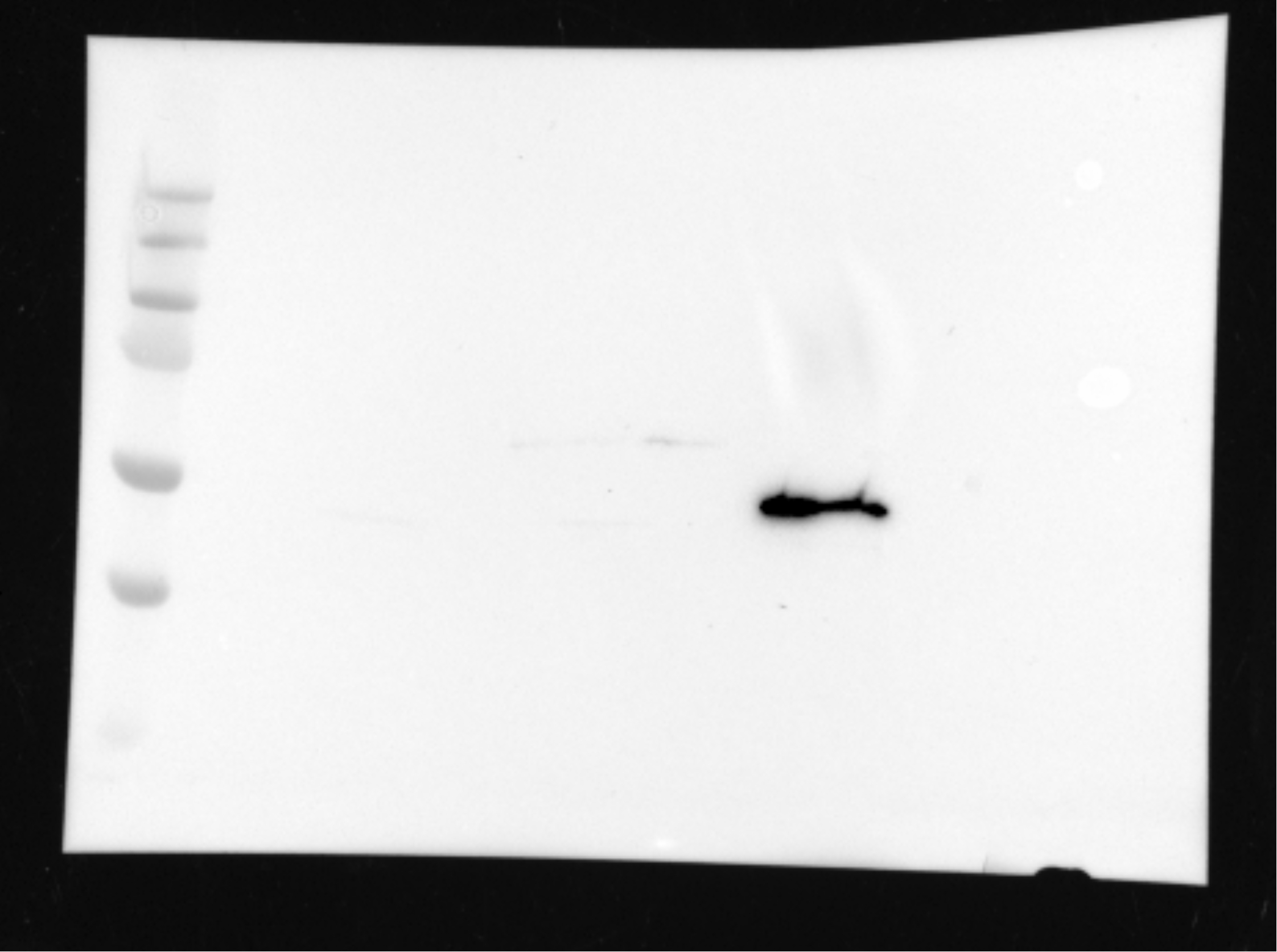

Supplement: Supplementary file 10 — Appendix Figures Source Data [file 44319_2024_203_MOESM10_ESM.zip › Appendix5_RASSF4/Fourthrow/Middle/Pulldown.jpg]

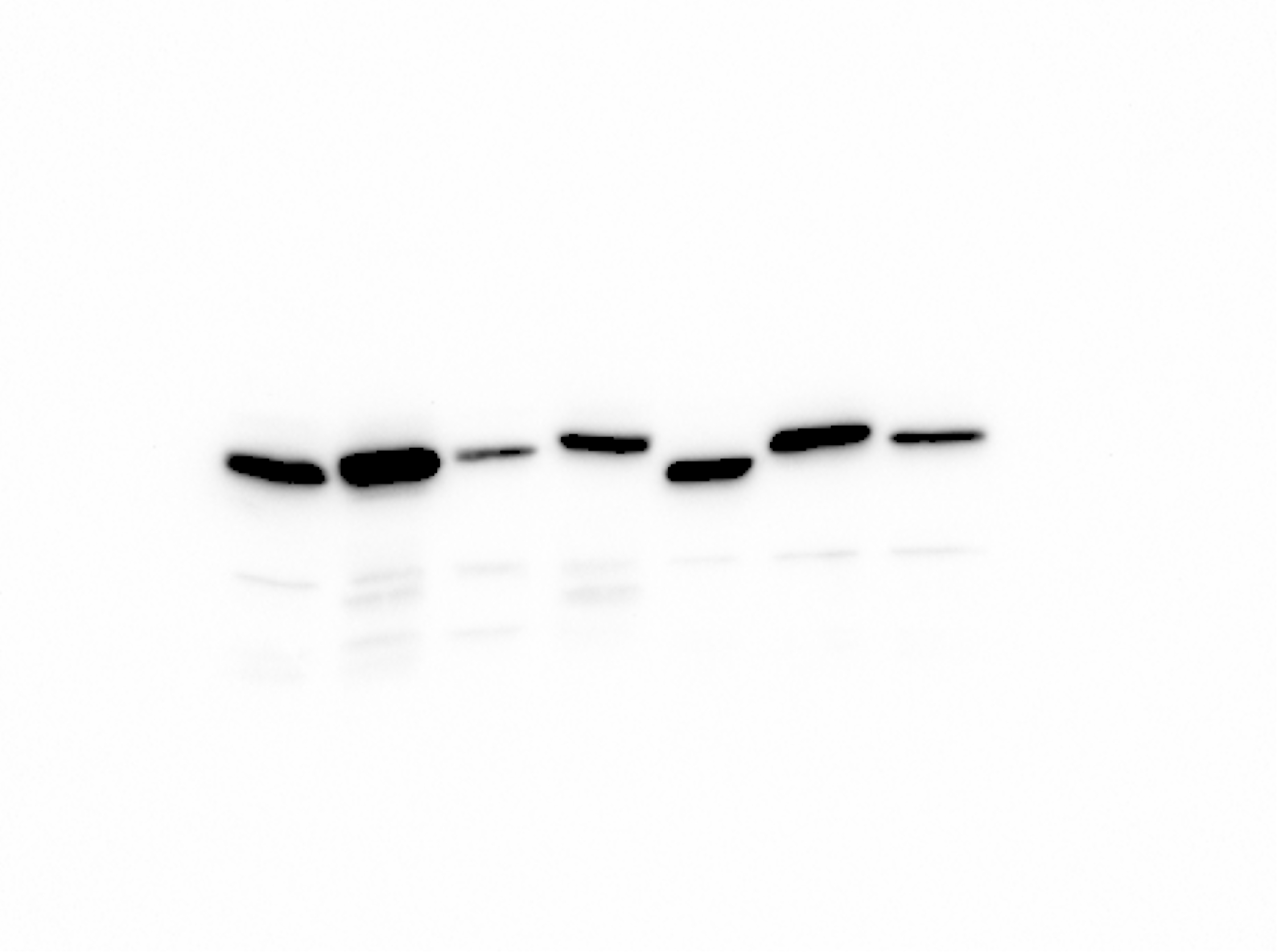

Supplement: Supplementary file 10 — Appendix Figures Source Data [file 44319_2024_203_MOESM10_ESM.zip › Appendix5_RASSF4/Fourthrow/Right/Lysate.jpg]

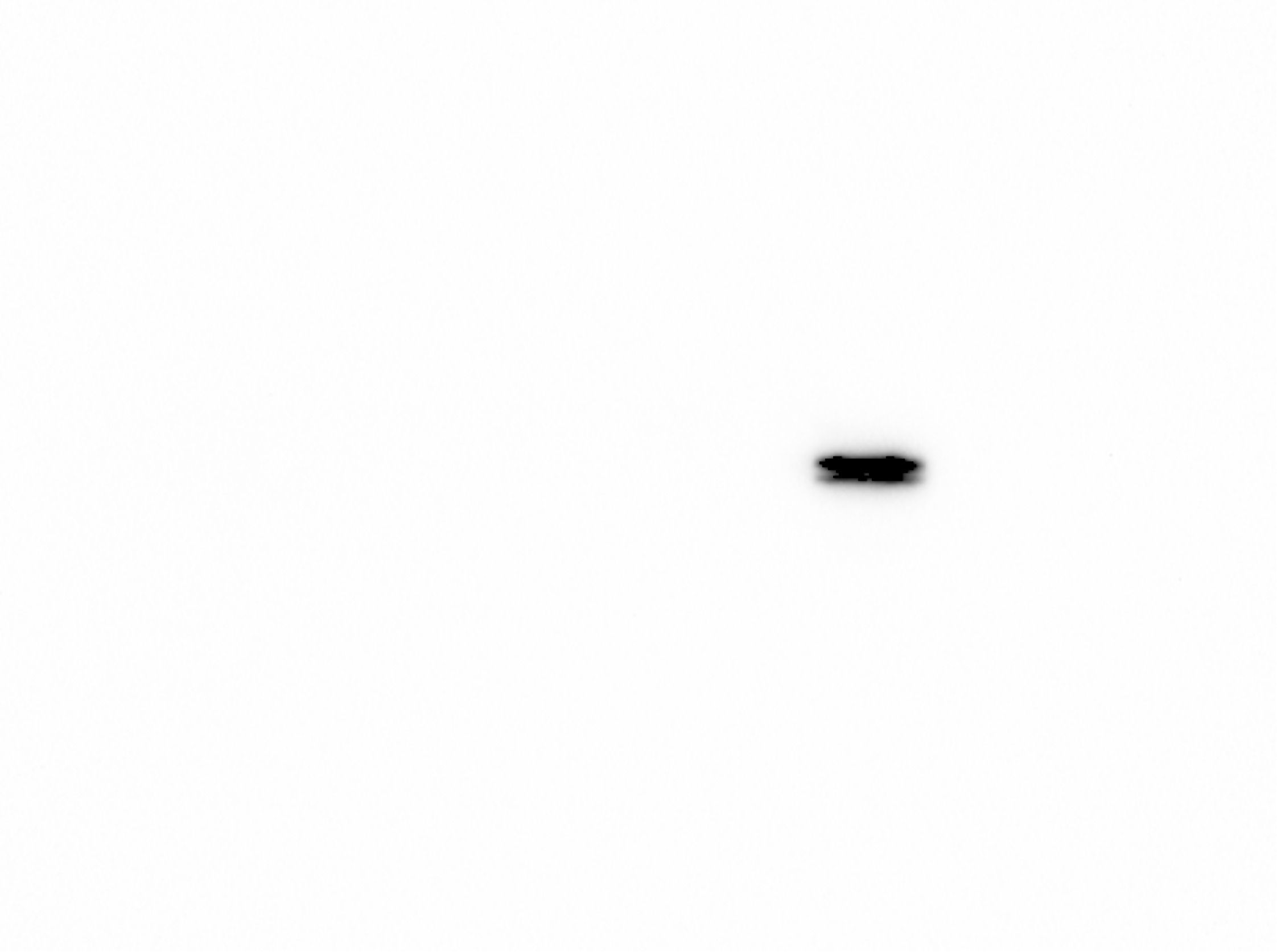

Supplement: Supplementary file 10 — Appendix Figures Source Data [file 44319_2024_203_MOESM10_ESM.zip › Appendix5_RASSF4/Fourthrow/Right/Pulldown.jpg]

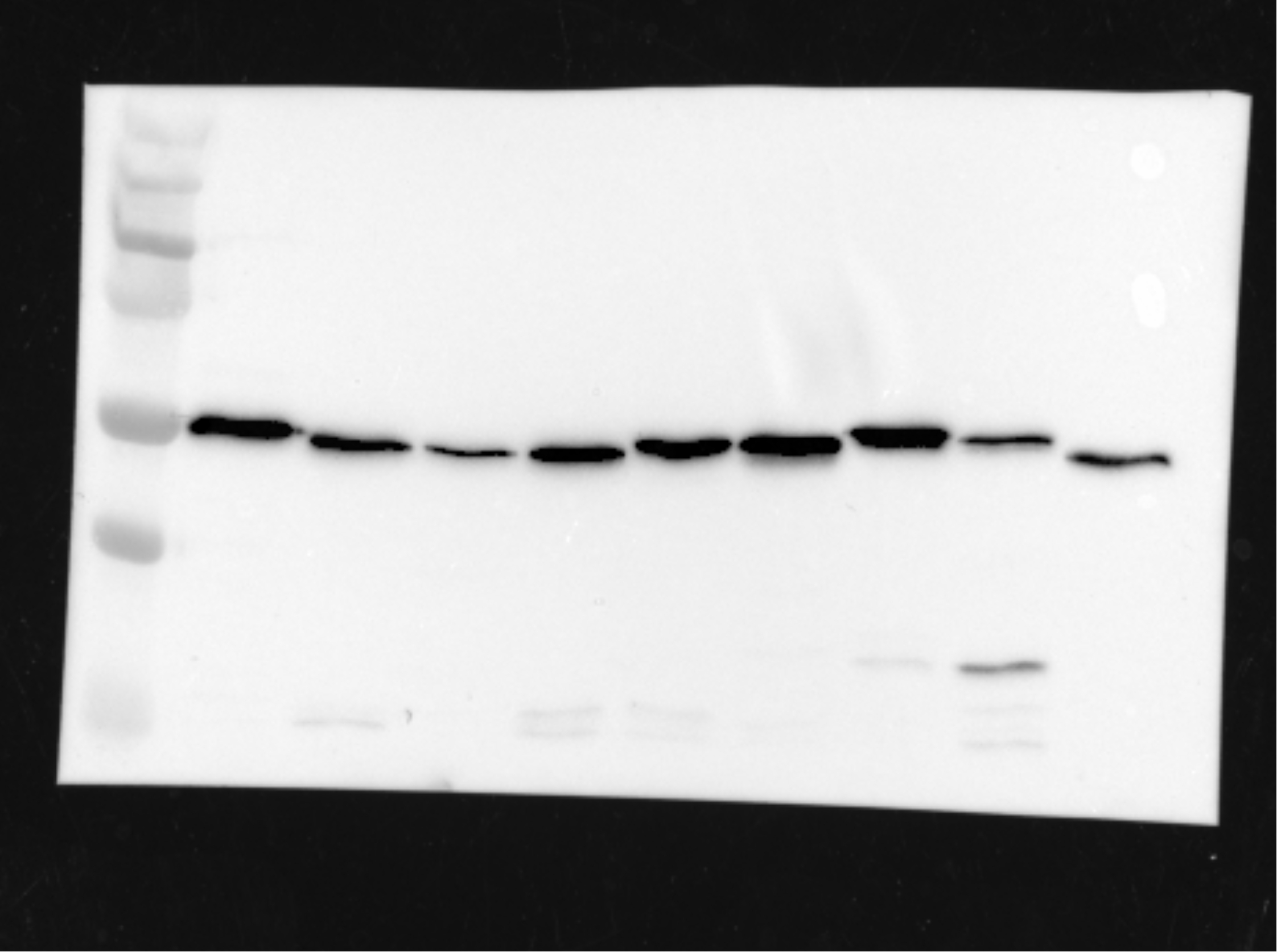

Supplement: Supplementary file 10 — Appendix Figures Source Data [file 44319_2024_203_MOESM10_ESM.zip › Appendix5_RASSF4/Secondrow/Left/Lysate.jpg]

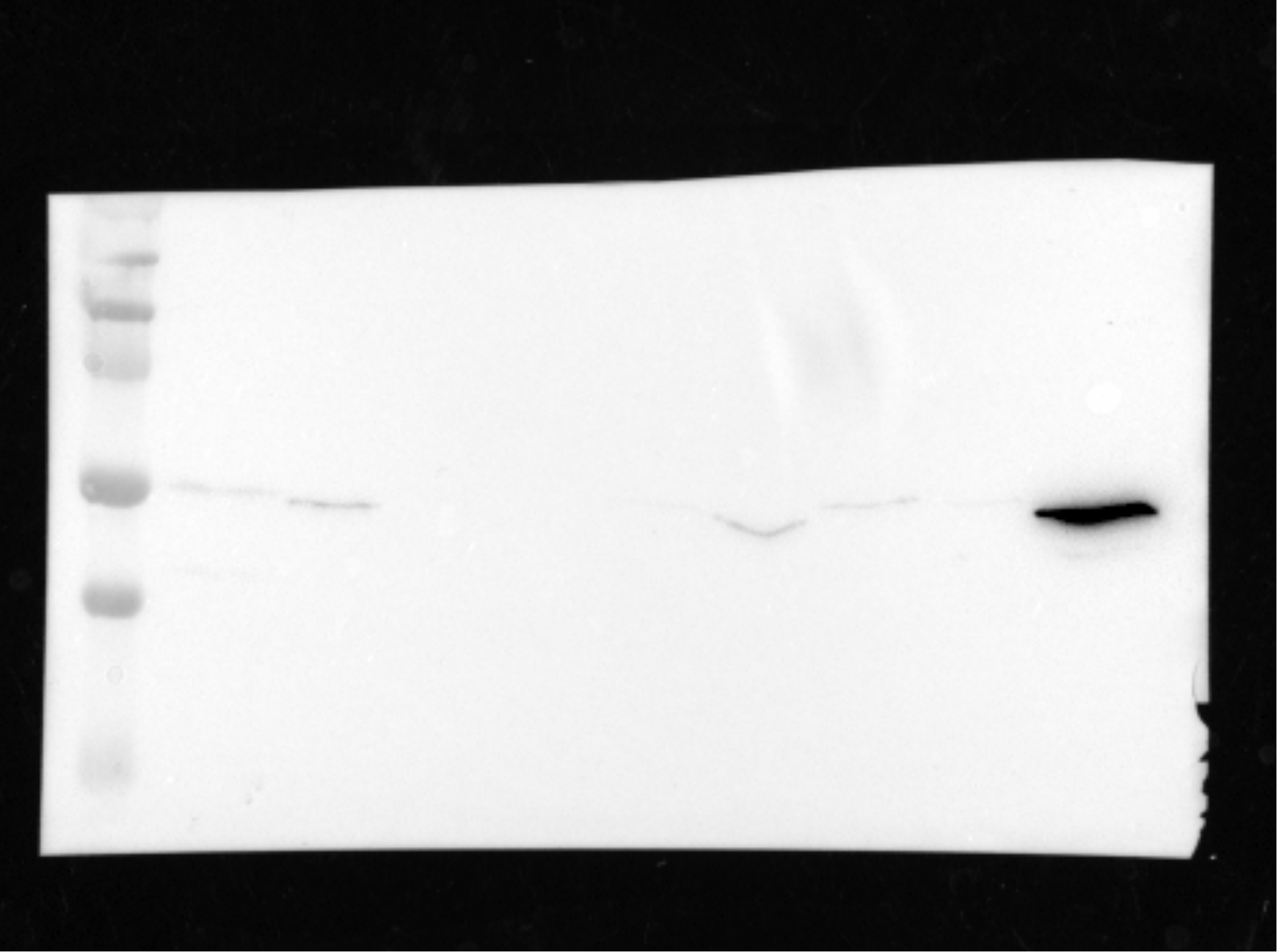

Supplement: Supplementary file 10 — Appendix Figures Source Data [file 44319_2024_203_MOESM10_ESM.zip › Appendix5_RASSF4/Secondrow/Left/Pulldown.jpg]

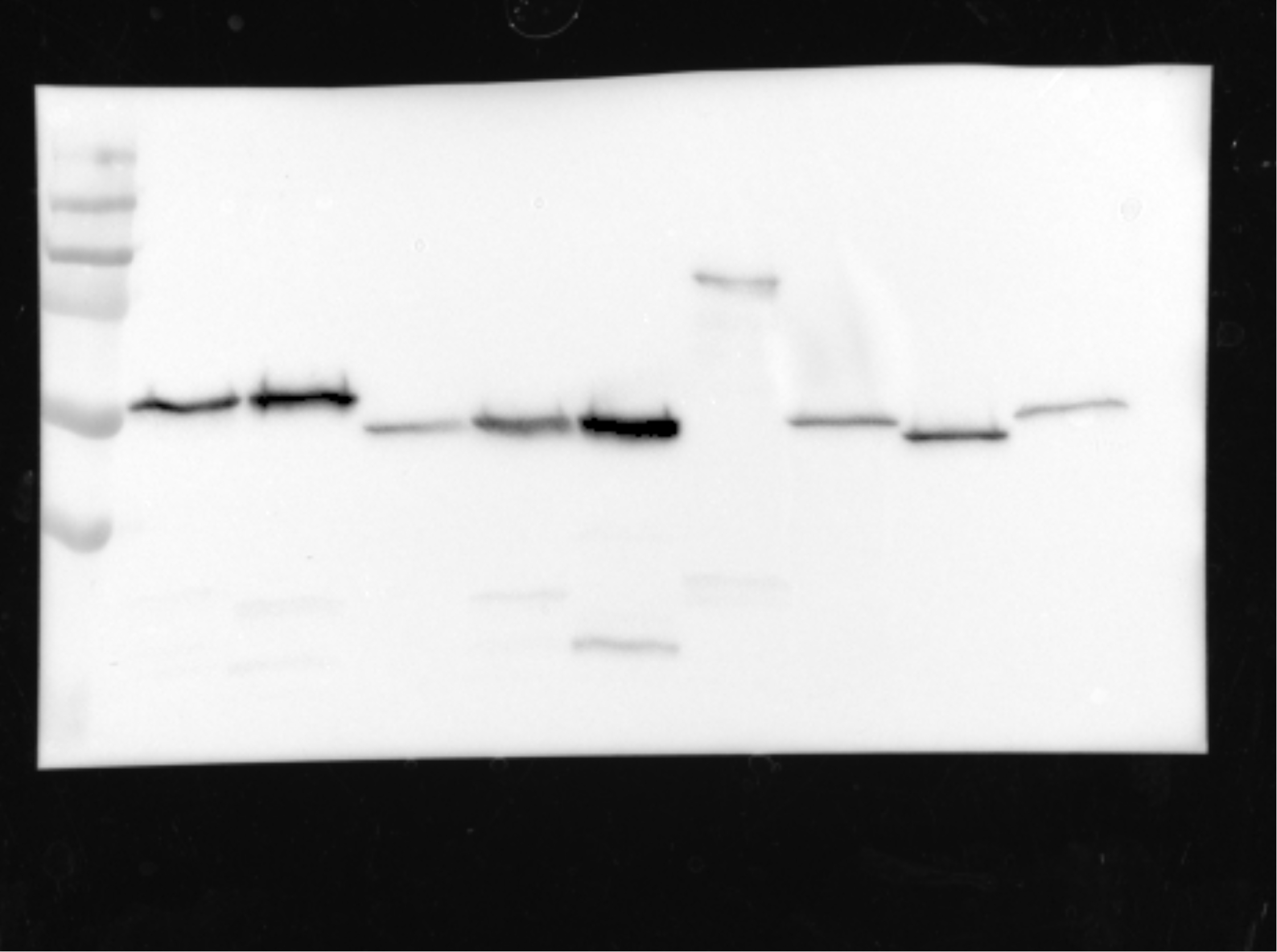

Supplement: Supplementary file 10 — Appendix Figures Source Data [file 44319_2024_203_MOESM10_ESM.zip › Appendix5_RASSF4/Secondrow/Middle/Lysate.jpg]

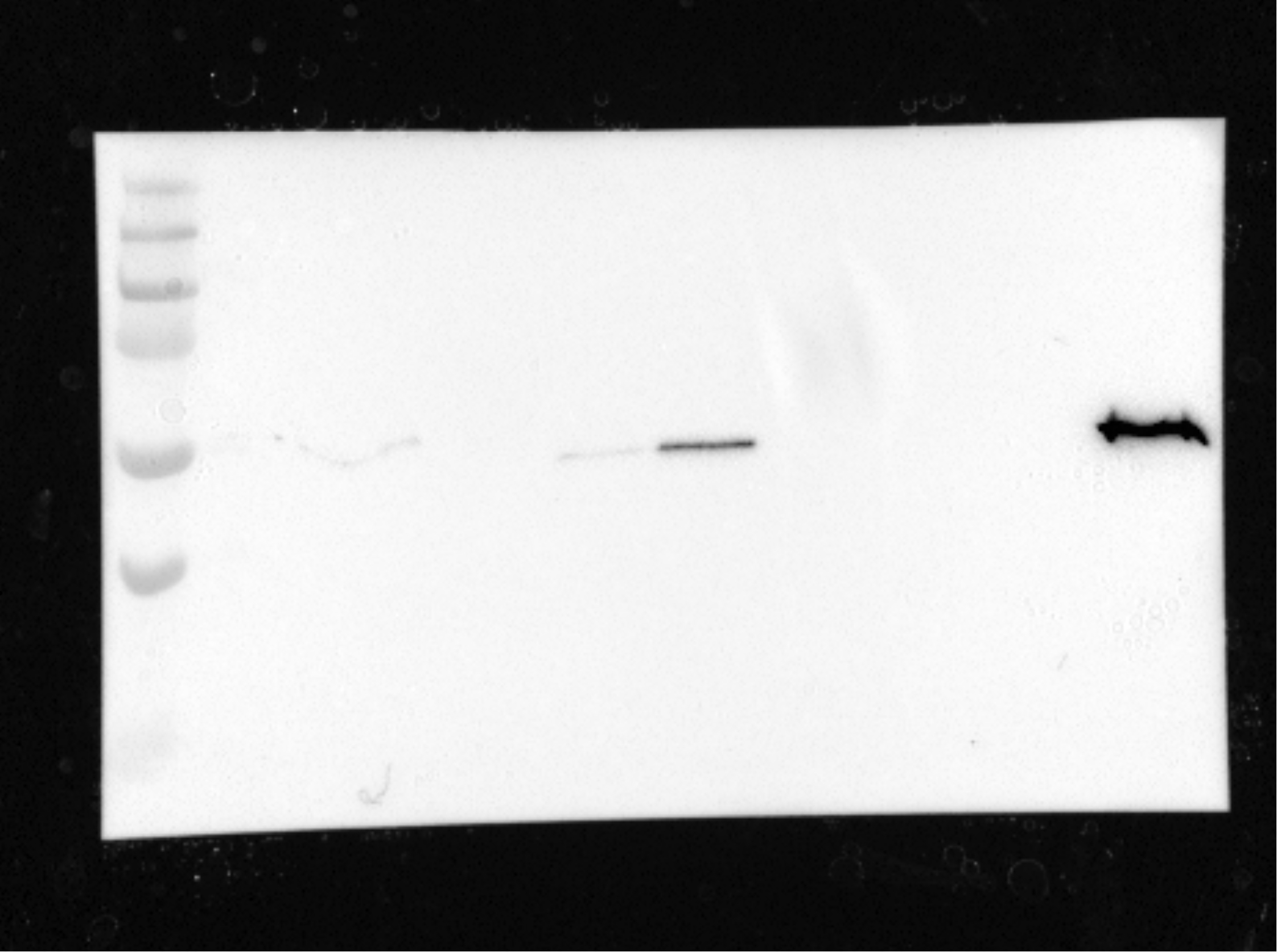

Supplement: Supplementary file 10 — Appendix Figures Source Data [file 44319_2024_203_MOESM10_ESM.zip › Appendix5_RASSF4/Secondrow/Middle/Pulldown.jpg]

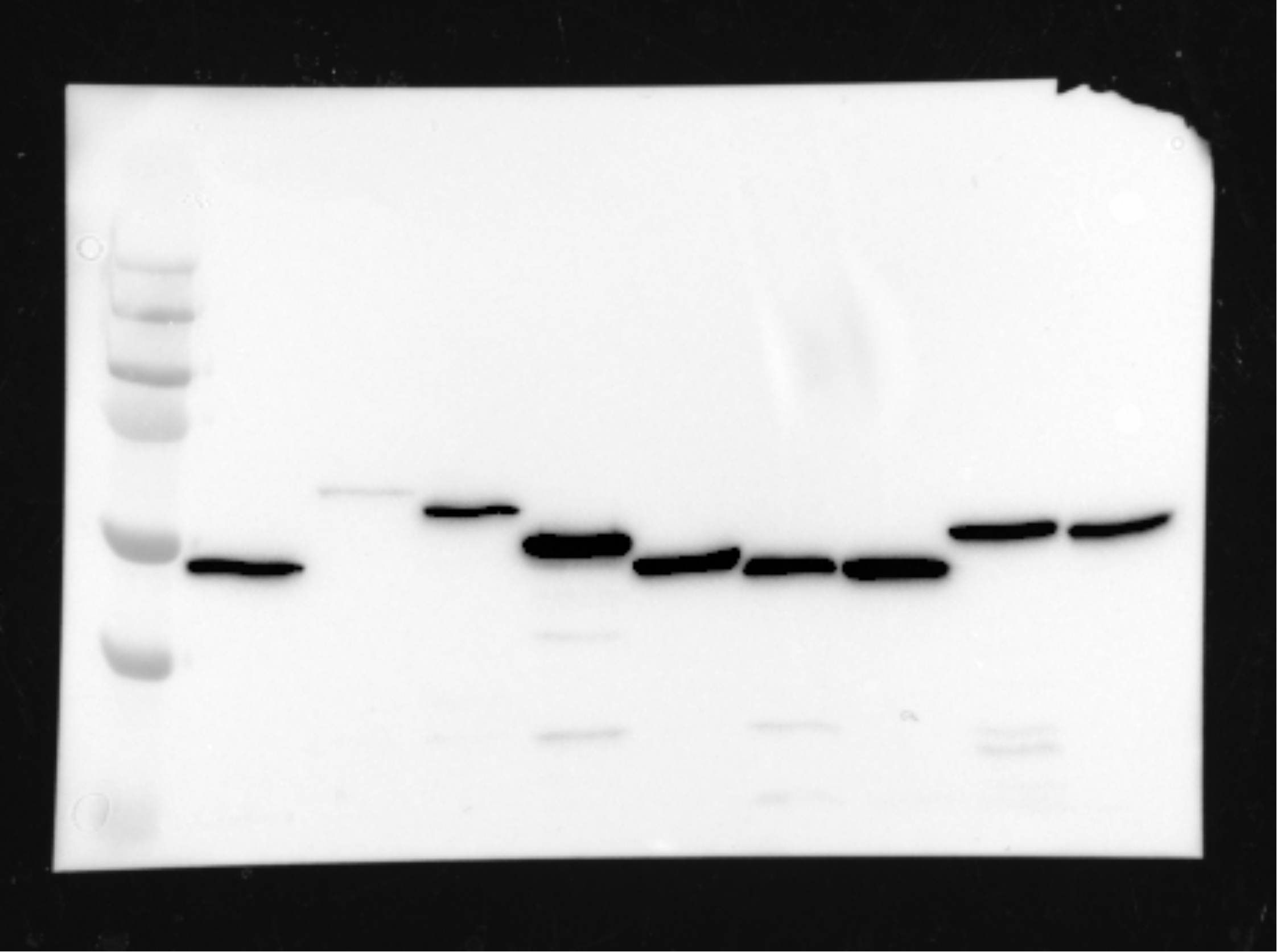

Supplement: Supplementary file 10 — Appendix Figures Source Data [file 44319_2024_203_MOESM10_ESM.zip › Appendix5_RASSF4/Secondrow/Right/Lysate.jpg]

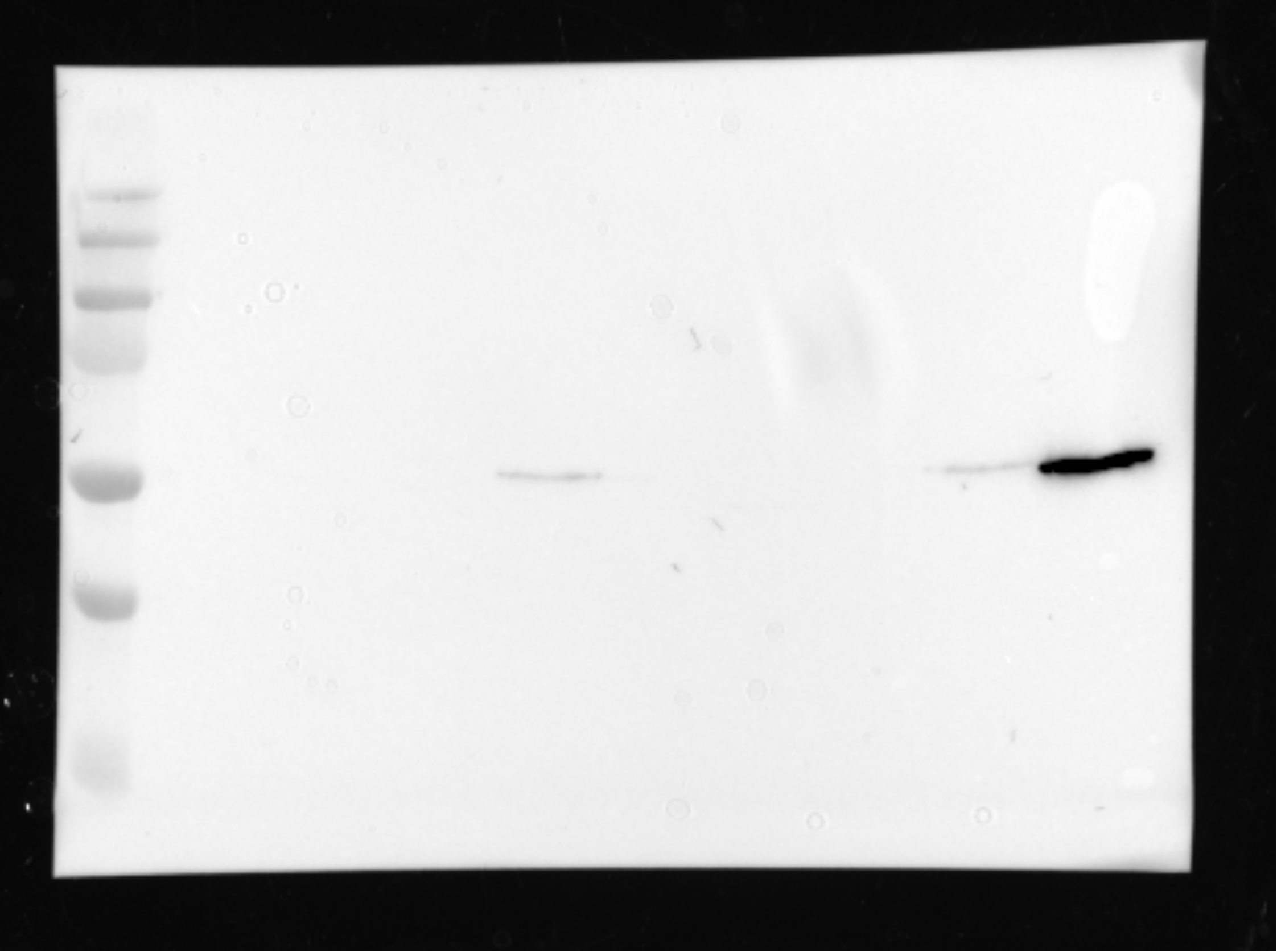

Supplement: Supplementary file 10 — Appendix Figures Source Data [file 44319_2024_203_MOESM10_ESM.zip › Appendix5_RASSF4/Secondrow/Right/Pulldown.jpg]

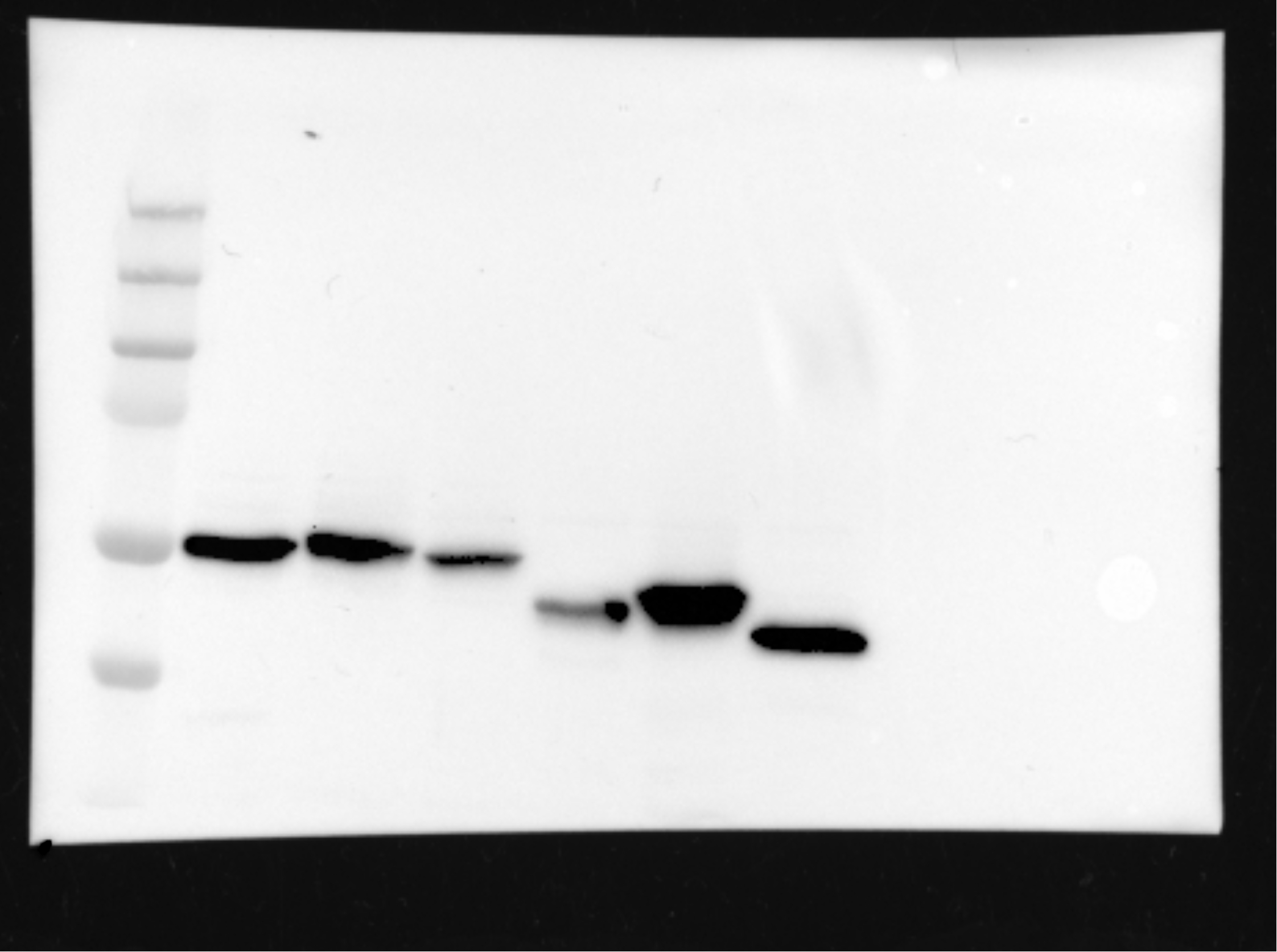

Supplement: Supplementary file 10 — Appendix Figures Source Data [file 44319_2024_203_MOESM10_ESM.zip › Appendix5_RASSF4/Thirdrow/Left/Lysate.jpg]

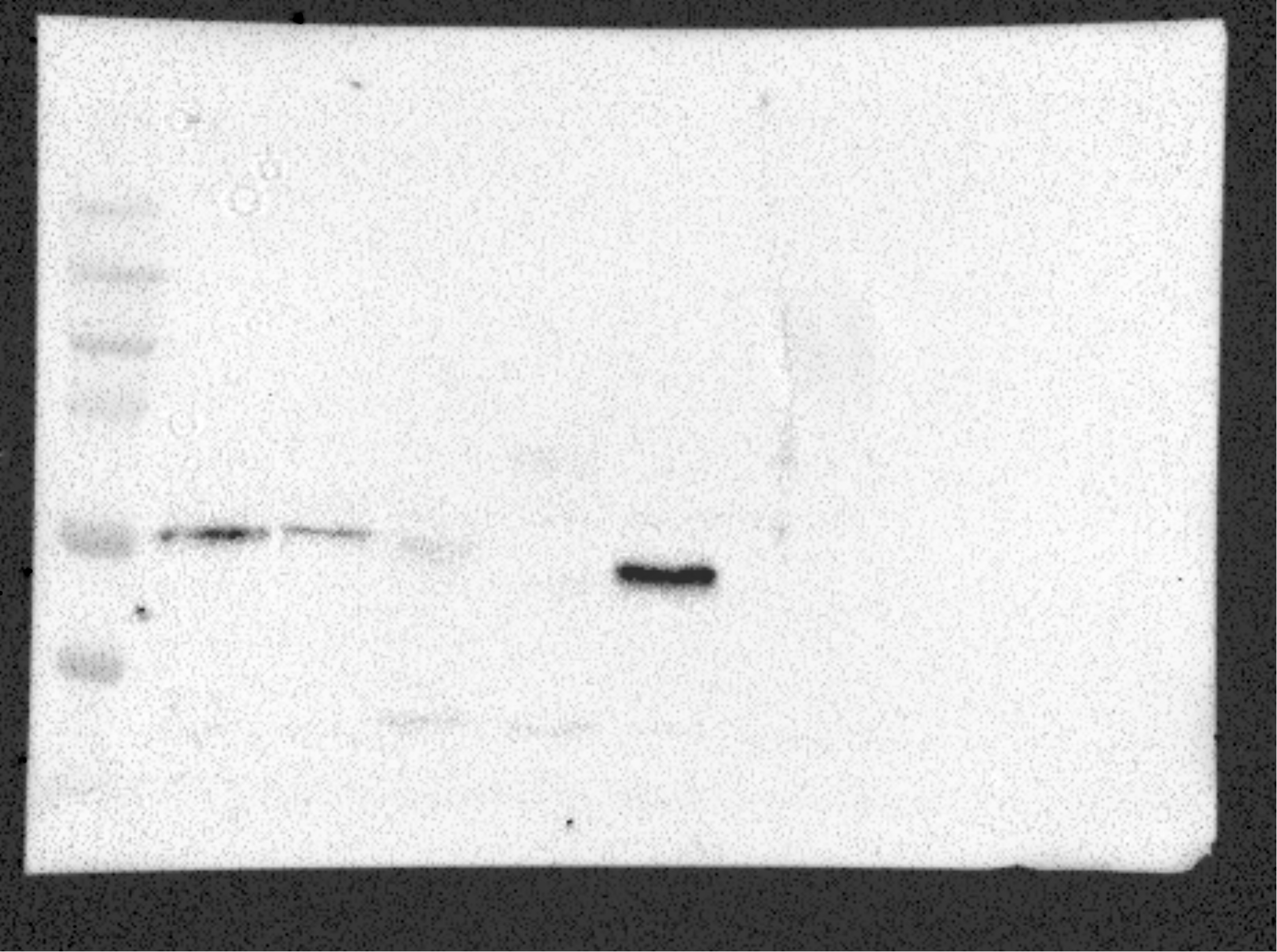

Supplement: Supplementary file 10 — Appendix Figures Source Data [file 44319_2024_203_MOESM10_ESM.zip › Appendix5_RASSF4/Thirdrow/Left/Pulldown.jpg]

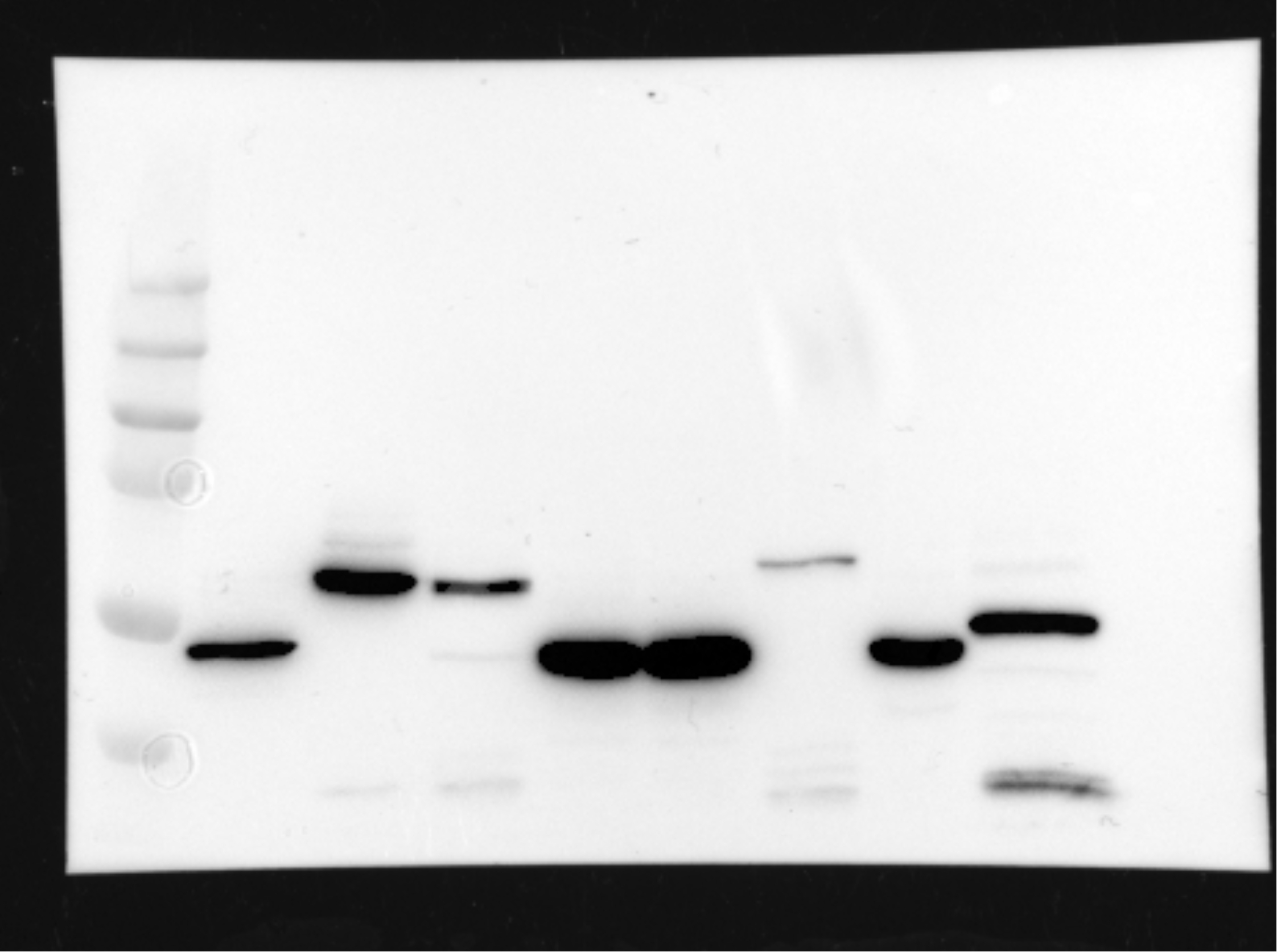

Supplement: Supplementary file 10 — Appendix Figures Source Data [file 44319_2024_203_MOESM10_ESM.zip › Appendix5_RASSF4/Thirdrow/Middle/Lysate.jpg]

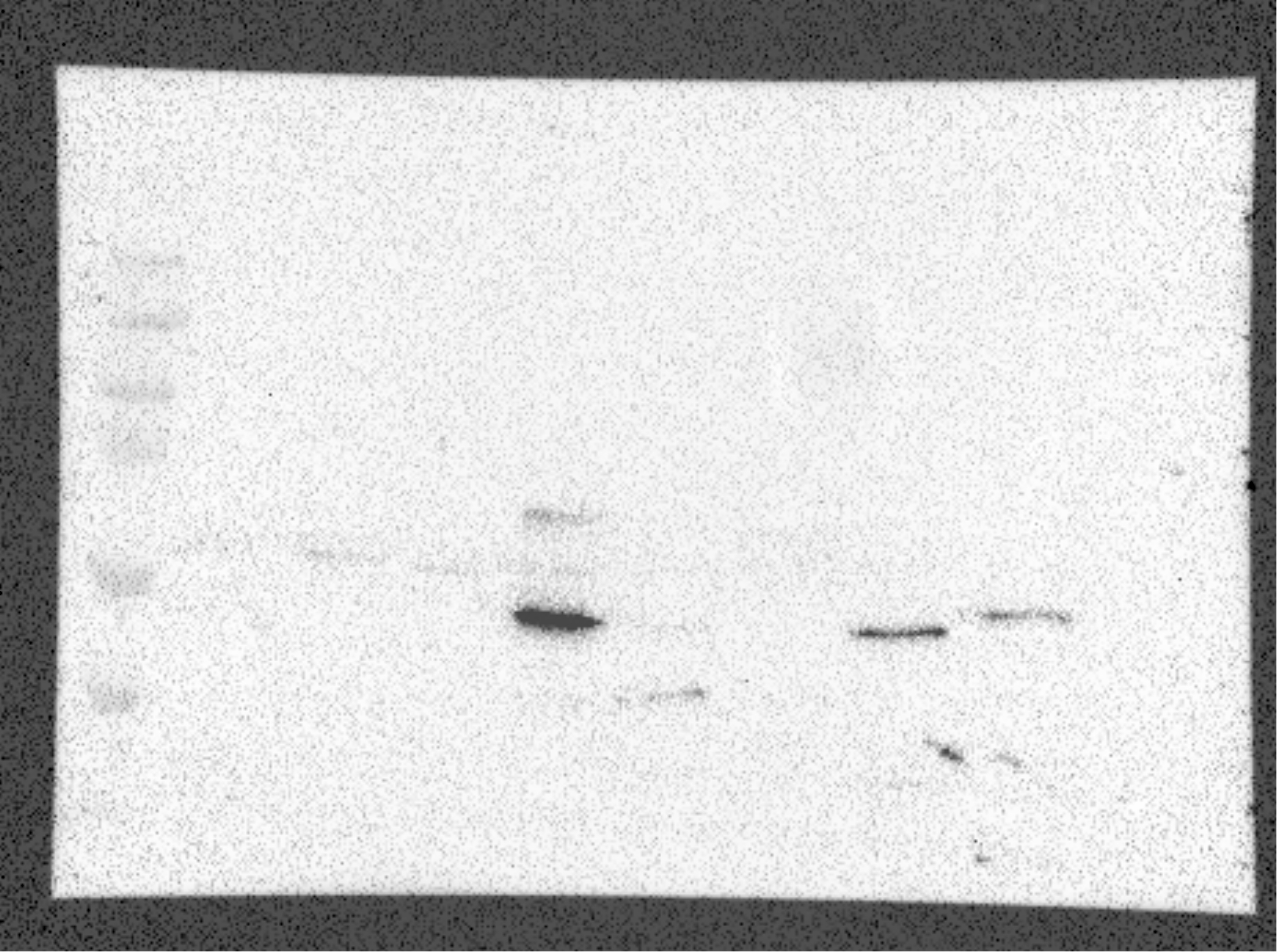

Supplement: Supplementary file 10 — Appendix Figures Source Data [file 44319_2024_203_MOESM10_ESM.zip › Appendix5_RASSF4/Thirdrow/Middle/Pulldown.jpg]

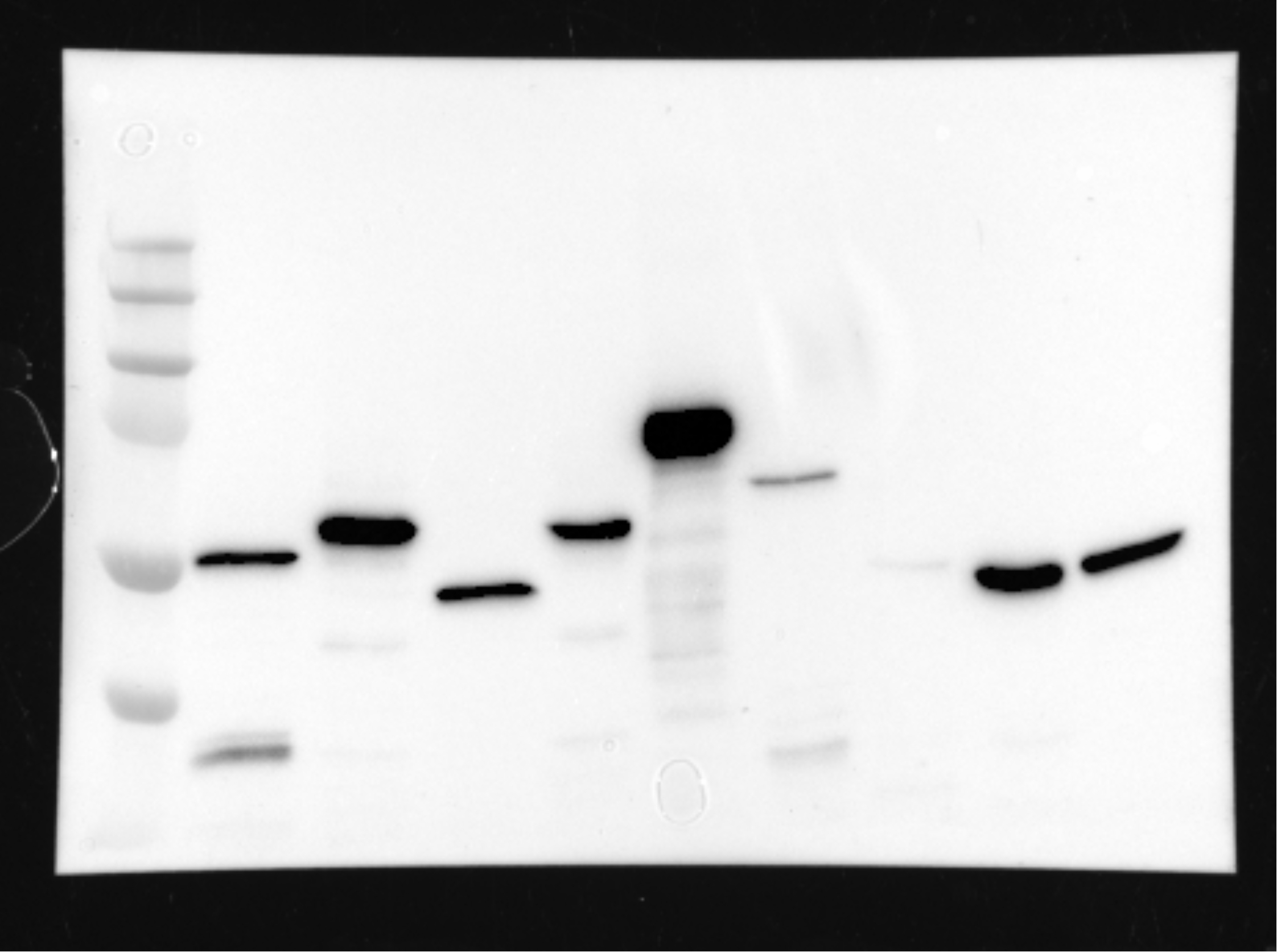

Supplement: Supplementary file 10 — Appendix Figures Source Data [file 44319_2024_203_MOESM10_ESM.zip › Appendix5_RASSF4/Thirdrow/Right/Lysate.jpg]

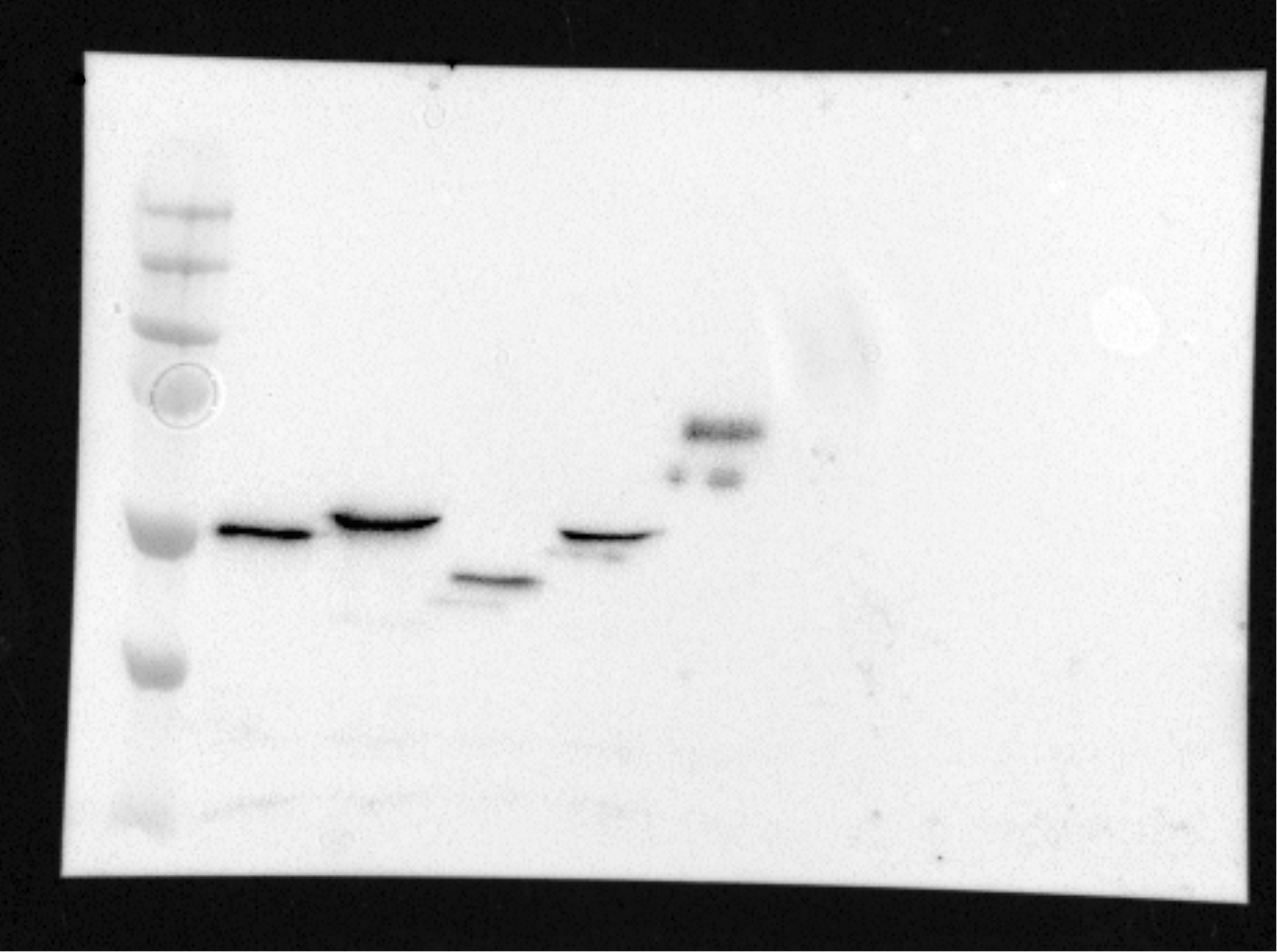

Supplement: Supplementary file 10 — Appendix Figures Source Data [file 44319_2024_203_MOESM10_ESM.zip › Appendix5_RASSF4/Thirdrow/Right/Pulldown.jpg]

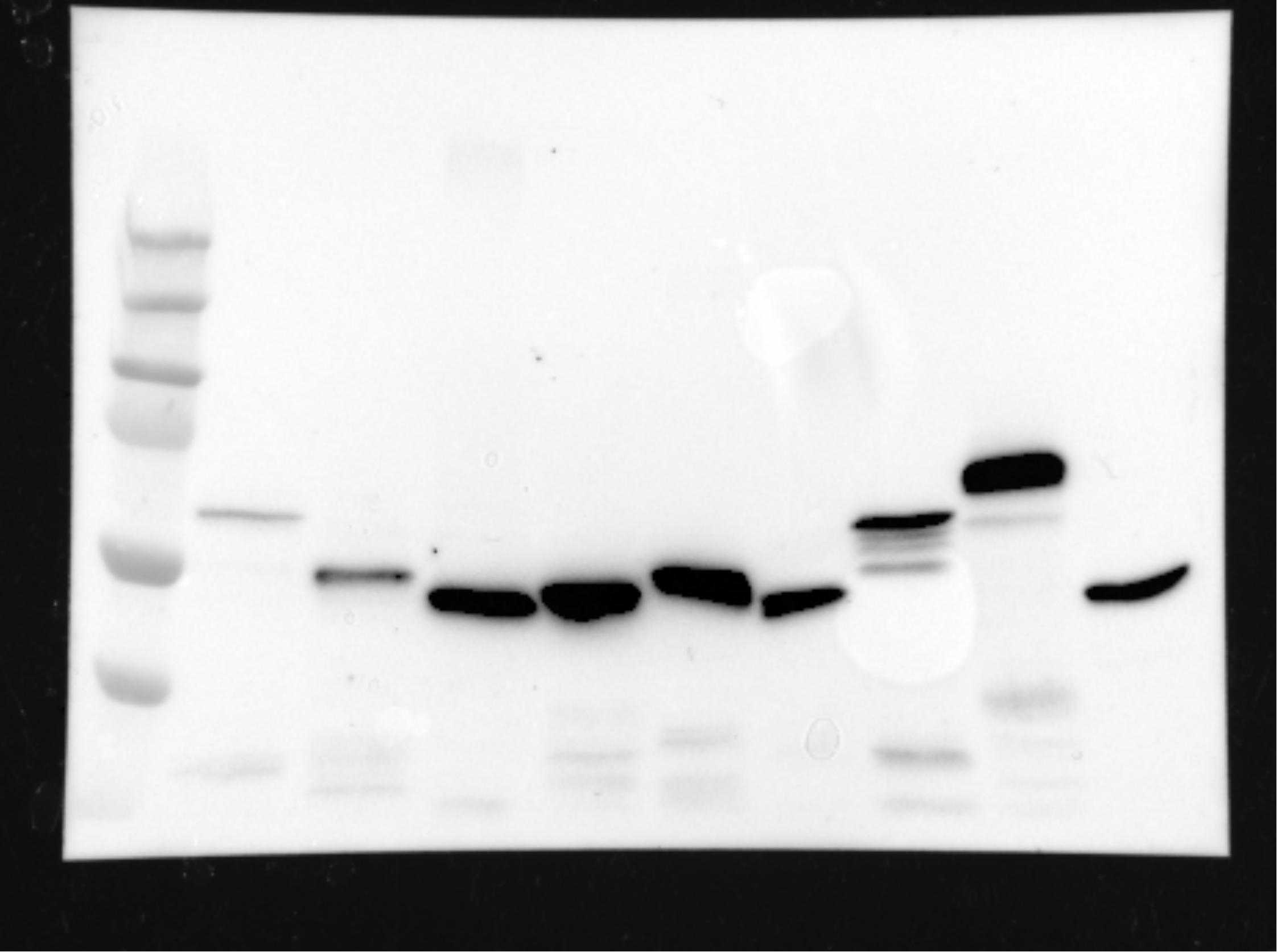

Supplement: Supplementary file 10 — Appendix Figures Source Data [file 44319_2024_203_MOESM10_ESM.zip › Appendix5_RASSF4/Toprow/Left/Lysate.jpg]

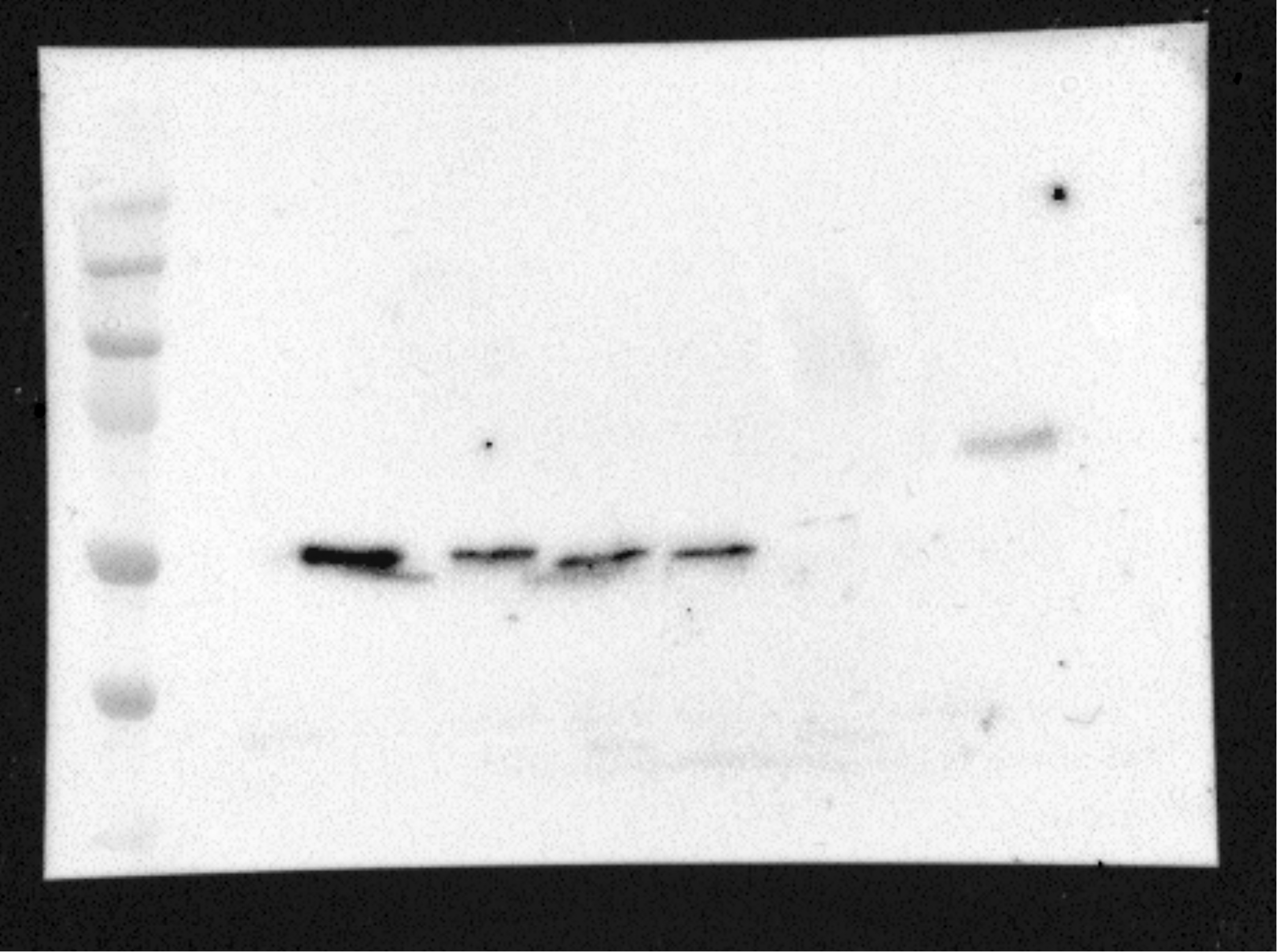

Supplement: Supplementary file 10 — Appendix Figures Source Data [file 44319_2024_203_MOESM10_ESM.zip › Appendix5_RASSF4/Toprow/Left/Pulldown.jpg]

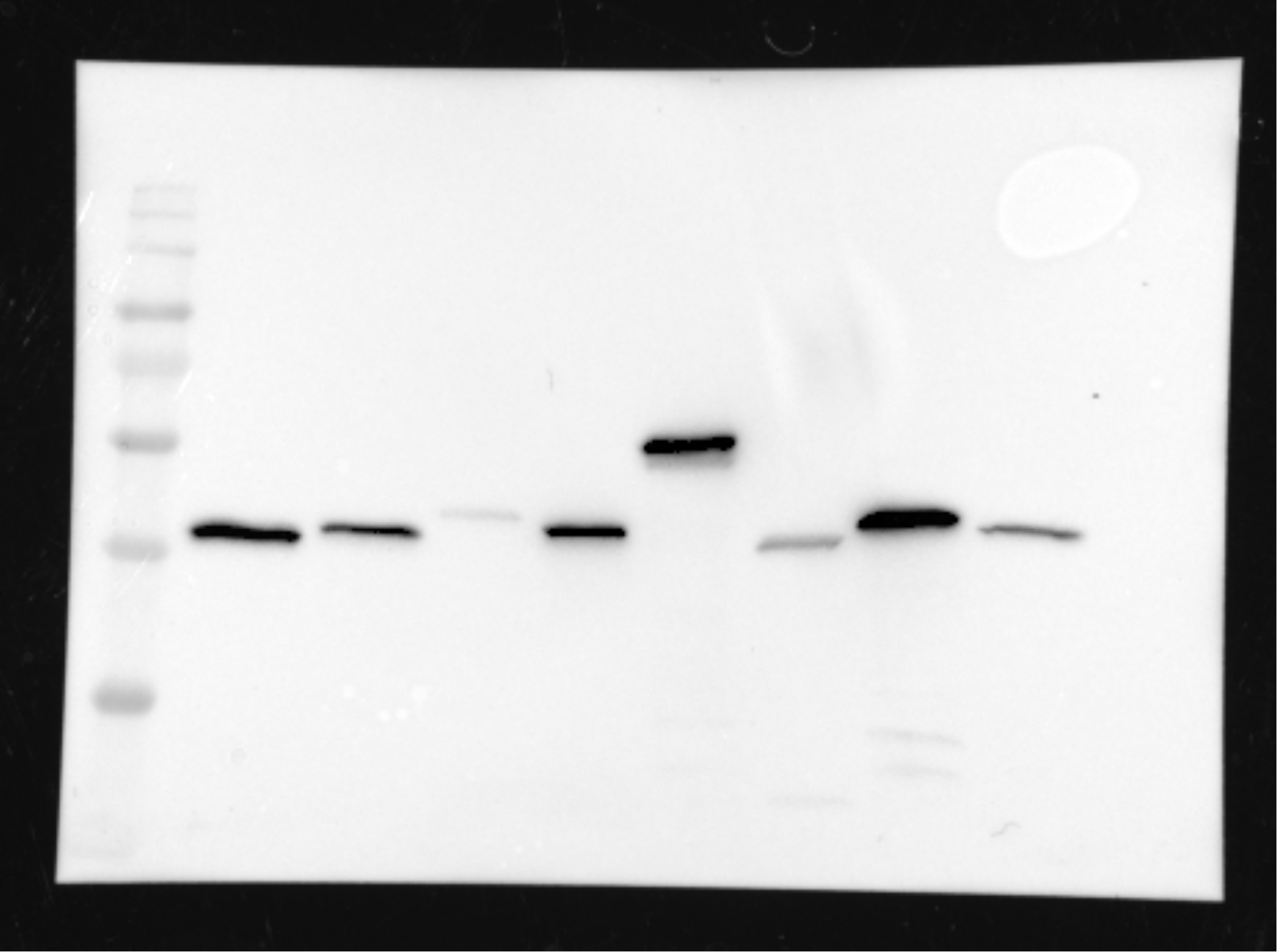

Supplement: Supplementary file 10 — Appendix Figures Source Data [file 44319_2024_203_MOESM10_ESM.zip › Appendix5_RASSF4/Toprow/Middle/Lysate.jpg]

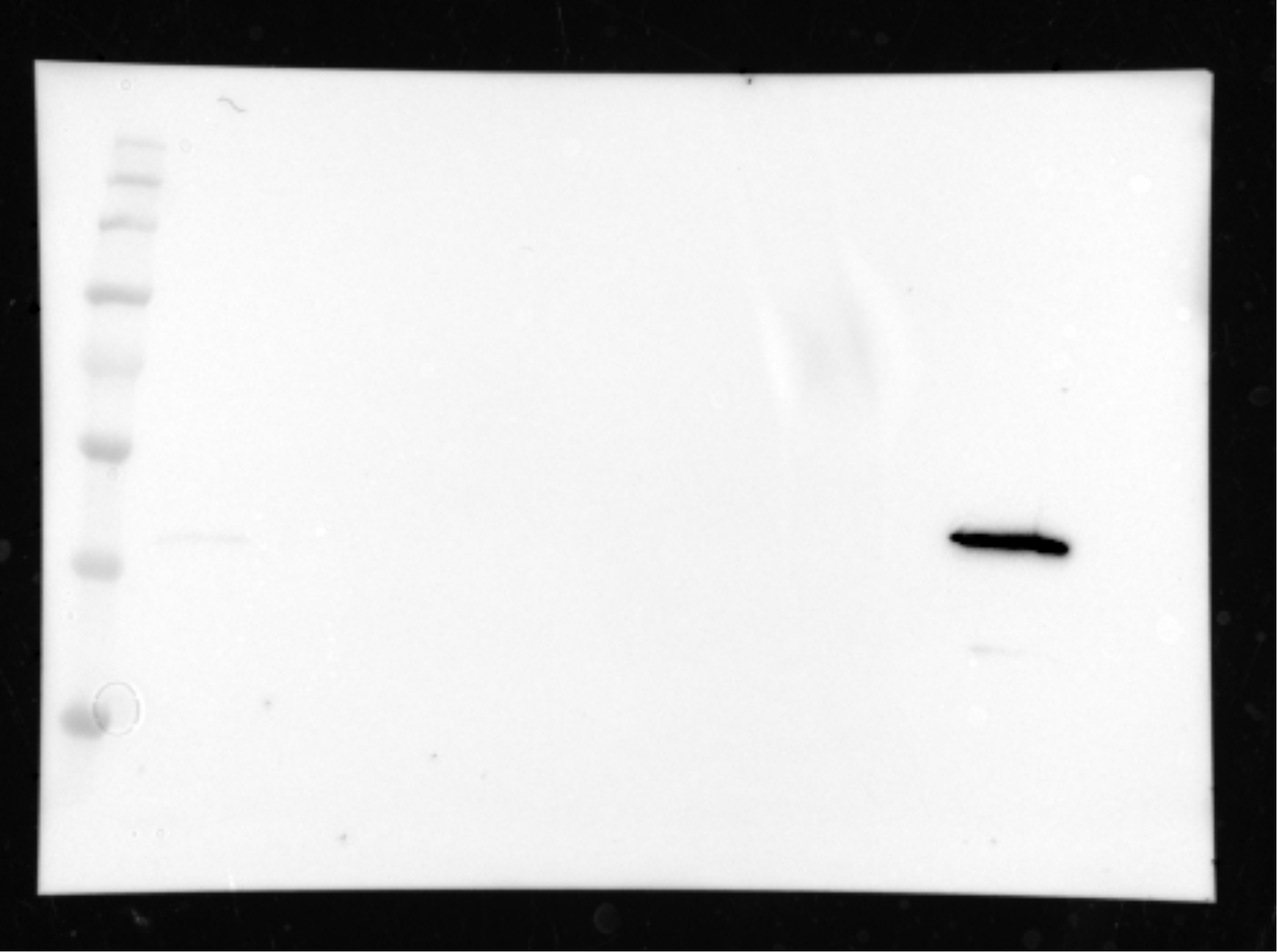

Supplement: Supplementary file 10 — Appendix Figures Source Data [file 44319_2024_203_MOESM10_ESM.zip › Appendix5_RASSF4/Toprow/Middle/Pulldown.jpg]

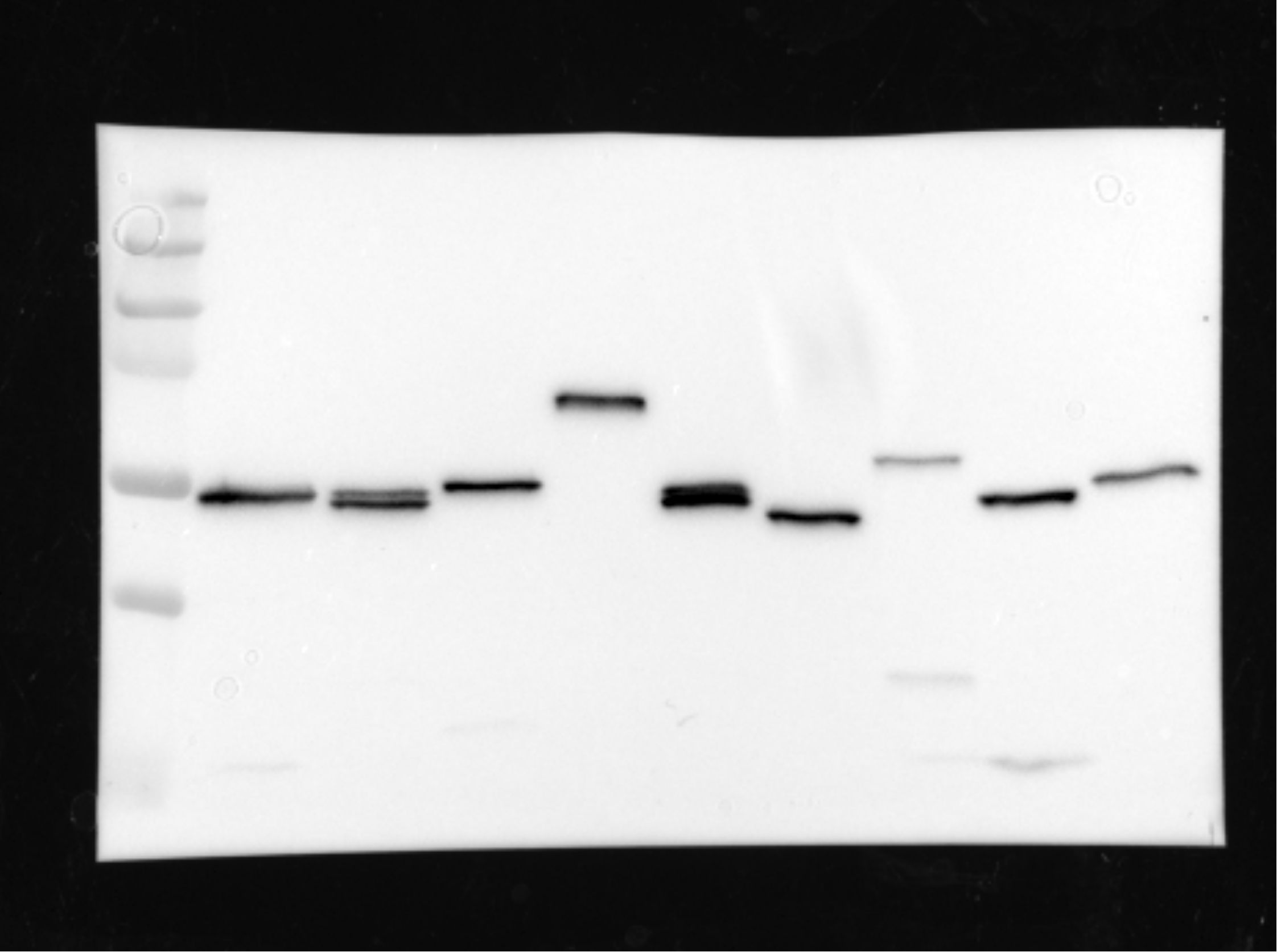

Supplement: Supplementary file 10 — Appendix Figures Source Data [file 44319_2024_203_MOESM10_ESM.zip › Appendix5_RASSF4/Toprow/Right/Lysate.jpg]

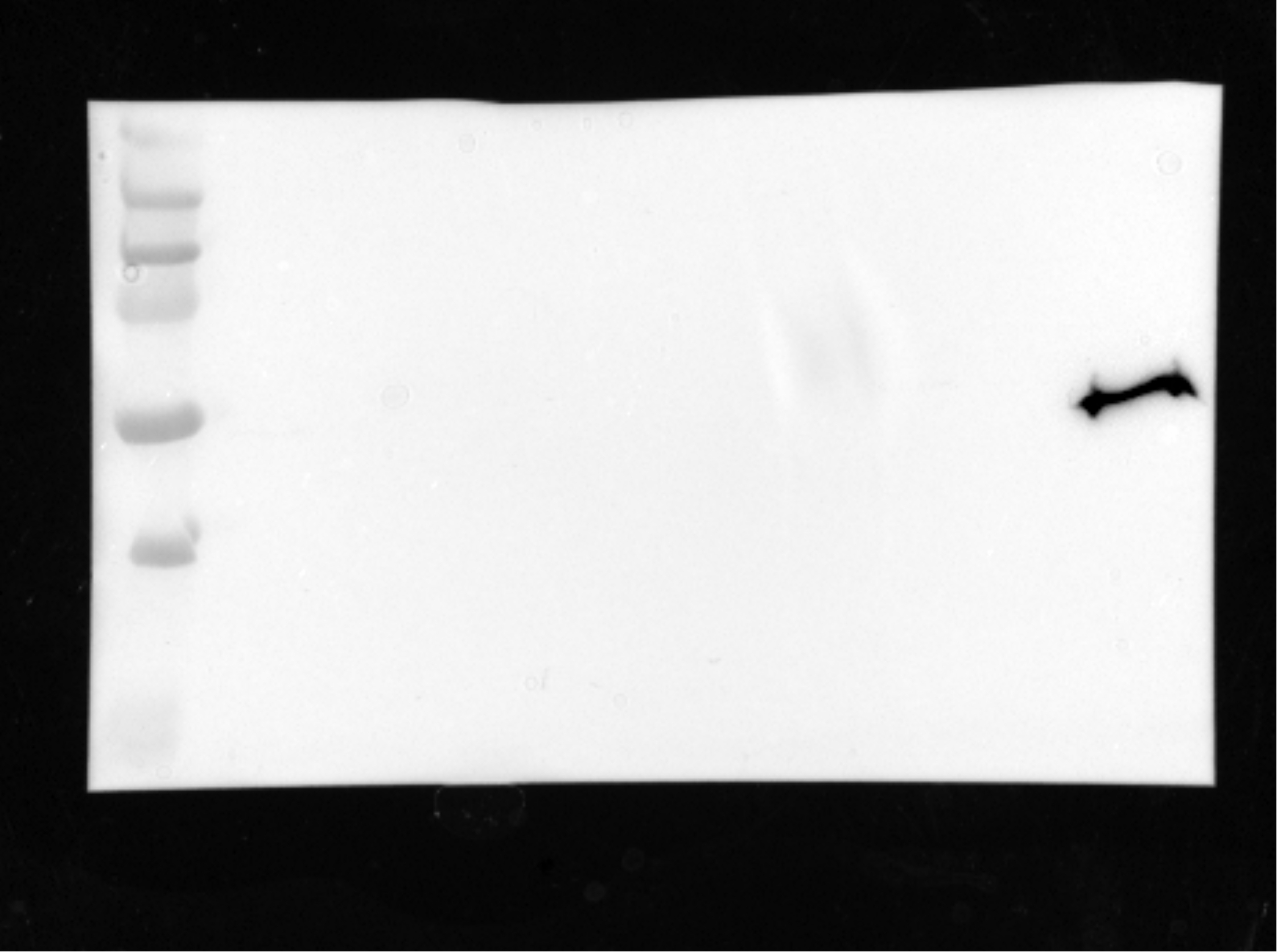

Supplement: Supplementary file 10 — Appendix Figures Source Data [file 44319_2024_203_MOESM10_ESM.zip › Appendix5_RASSF4/Toprow/Right/Pulldown.jpg]
