# Supplementary material for: Complex interplay between RAS GTPases and RASSF effectors regulates subcellular localization of YAP
Source: EMBO Rep. 2024 Jul 15;25(8):22. doi: 10.1038/s44319-024-00203-9 (PMC11316025; doi:10.1038/s44319-024-00203-9)
Supplement: Supplementary file 11 — Expanded View Figures [file 44319_2024_203_MOESM11_ESM.pdf]

Expanded View Figures

Figure EV1. Mapping BRAF and RASSF interactions with the RHO and ARF subfamilies of small GTPases.

(**A**) Heatmap of BRAF RBD and RASSF3/4/5/8 RA binding to activated GTPases of the RHO subfamily. Strong interactions are in red, moderate in yellow and no interaction in black. (**B**) Heatmap of BRAF RBD and RASSF3/4/5/8 RA domains binding to activated GTPases of the ARF subfamily. ARL17, ARL9, and ARL16 were not included in the screen. Strong interactions are in red, moderate in yellow and no interaction in black. (**C**) Recombinantly purified GST-RASSF5 RA complexes with 10 distinct RAS subfamily GTPases following expression of VENUS-tagged, mutationally activated GTPases in HEK 293 T cells. RHEB served as a negative control. (**D**) Co-immunoprecipitation of ten candidate RAS subfamily GTPases following co-expression of the VENUS-tagged, mutationally activated variants with FLAG-RASSF5.

© The Author(s)

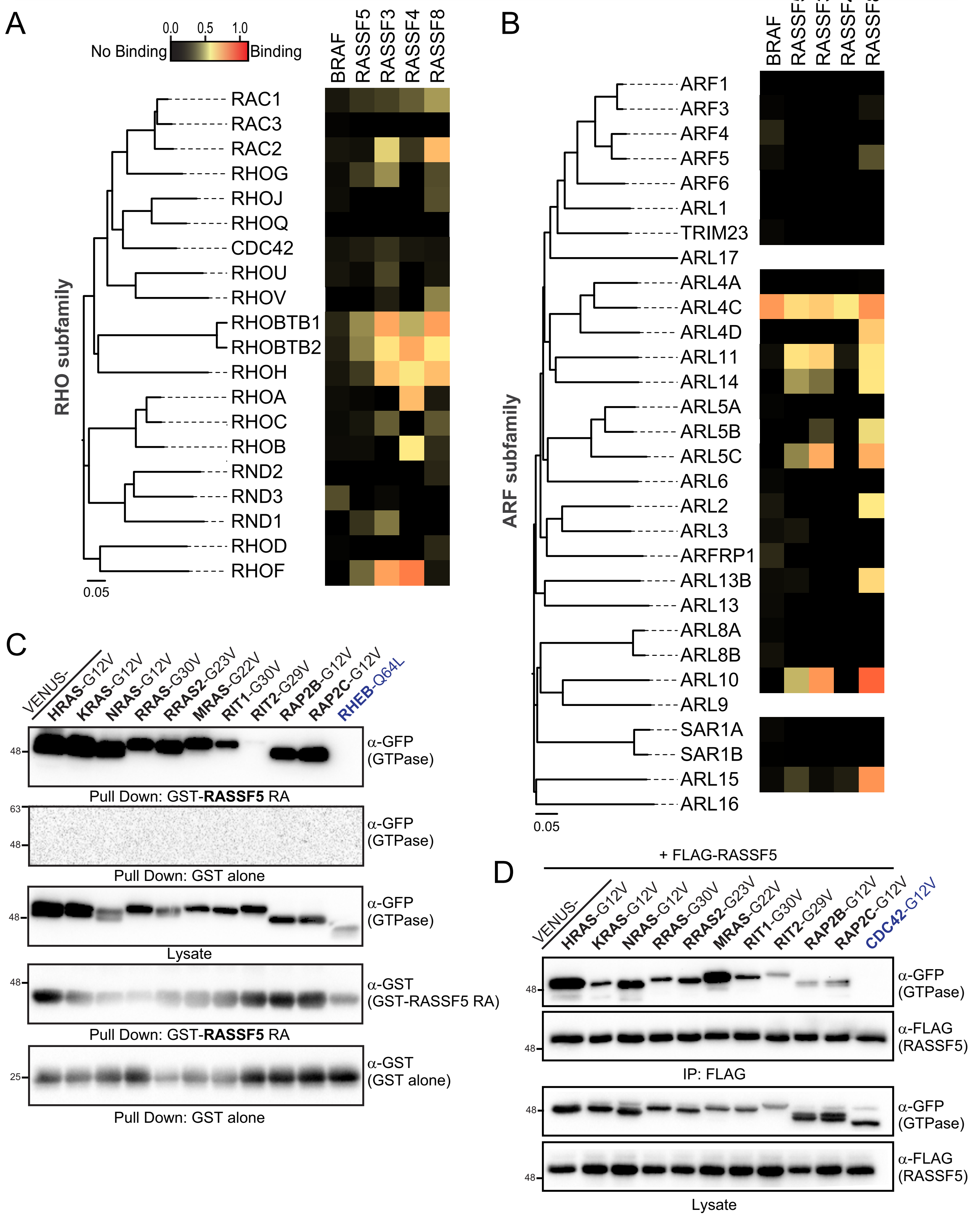
A
No Binding
Binding
0.0 0.5 1.0
BRAF RASSF5 RASSF3 RASSF4 RASSF8
RHO subfamily
RAC1
RAC3
RAC2
RHOG
RHOJ
RHOQ
CDC42
RHOU
RHOV
RHOBTB1
RHOBTB2
RHOH
RHOA
RHOC
RHOB
RND2
RND3
RND1
RHOD
RHOF
0.05
B
ARF subfamily
ARF1
ARF3
ARF4
ARF5
ARF6
ARL1
TRIM23
ARL17
ARL4A
ARL4C
ARL4D
ARL11
ARL14
ARL5A
ARL5B
ARL5C
ARL6
ARL2
ARL3
ARFRP1
ARL13B
ARL13
ARL8A
ARL8B
ARL10
ARL9
SAR1A
SAR1B
ARL15
ARL16
0.05
C
VENUS- HRAS-G12V KRAS-G12V NRAS-G12V RRAS-G30V RRAS2-G23V MRAS-G22V RIT1-G30V RIT2-G29V RAP2B-G12V RAP2C-G12V RHEB-Q64L
α-GFP (GTPase)
Pull Down: GST-RASSF5 RA
α-GFP (GTPase)
Pull Down: GST alone
α-GFP (GTPase)
Lysate
α-GST (GST-RASSF5 RA)
Pull Down: GST-RASSF5 RA
α-GST (GST alone)
Pull Down: GST alone
D
+ FLAG-RASSF5
VENUS- HRAS-G12V KRAS-G12V NRAS-G12V RRAS-G30V RRAS2-G23V MRAS-G22V RIT1-G30V RIT2-G29V RAP2B-G12V RAP2C-G12V CDC42-G12V
α-GFP (GTPase)
α-FLAG (RASSF5)
IP: FLAG
α-GFP (GTPase)
α-FLAG (RASSF5)
Lysate

 © The Author(s)

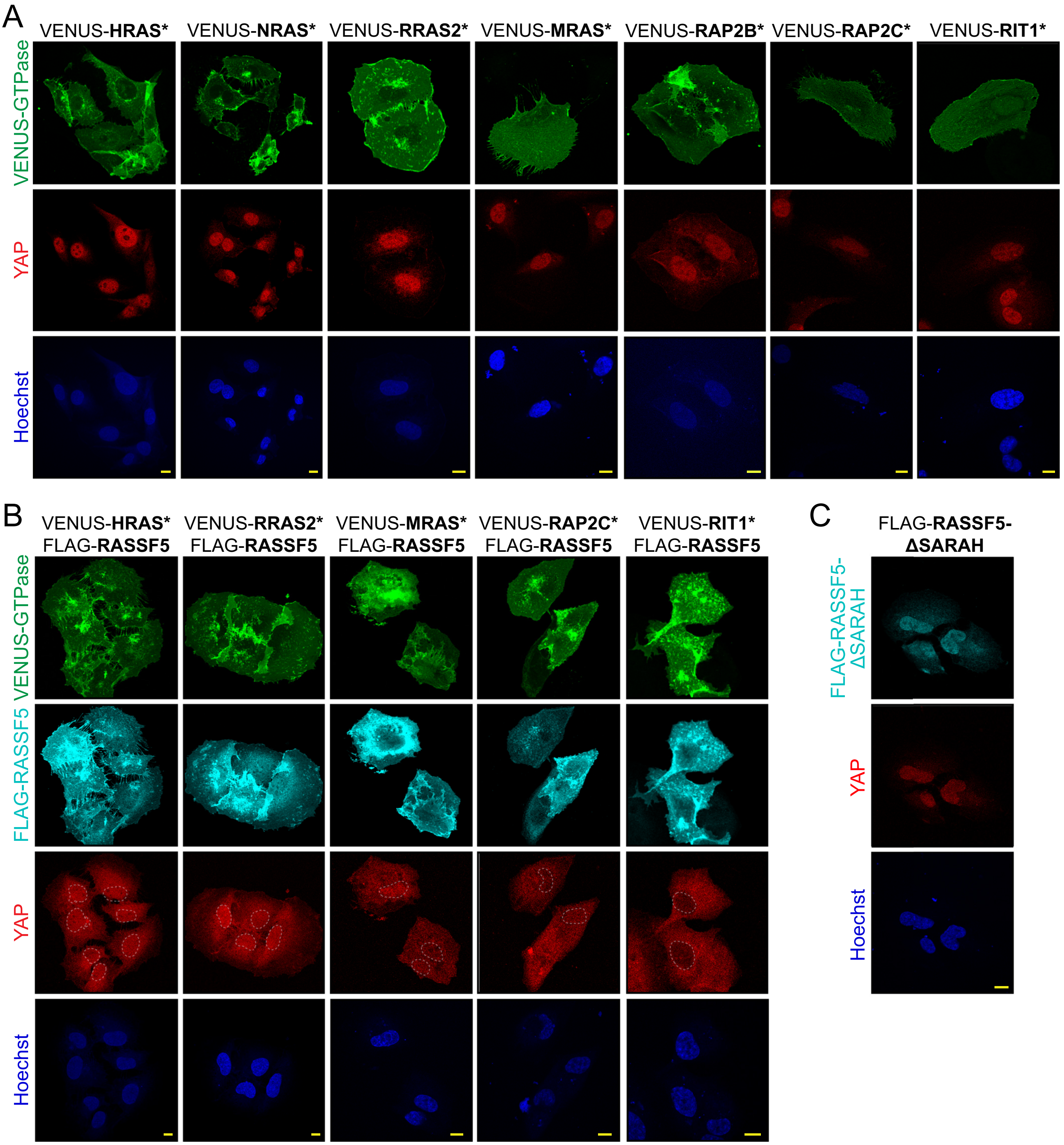

Figure EV2. The RAS subfamily GTPase partners of RASSF5 activate the Hippo pathway in a RASSF5-dependent manner.

(**A**) Immunofluorescence images of endogenous YAP (red) in sparsely confluent U2OS cells transiently expressing mutationally activated (*) VENUS-tagged HRAS-G12V, NRAS-G12V, RRAS2-G23V, MRAS-G22V, RAP2B-G12V, RAP2C-G12V, or RIT1-G30V. Nuclei were visualized with Hoechst staining (blue). Scale bars represent 10 μm. (**B**) Immunostaining of endogenous YAP (red) in sparsely confluent U2OS cells co-transfected with FLAG-RASSF5 (cyan) and mutationally activated (*) GTPases (HRAS-G12V, RRAS2-G23V, MRAS-G22V, RAP2C-G12V or RIT1-G30V). Hoechst staining (blue) was used to visualize nuclei, and scale bars represent 10 μm. (**C**) Immunostaining for YAP (red) in U2OS cells expressing FLAG-RASSF5-ΔSARAH (cyan). Scale bars represent 10 μm.

© The Author(s)

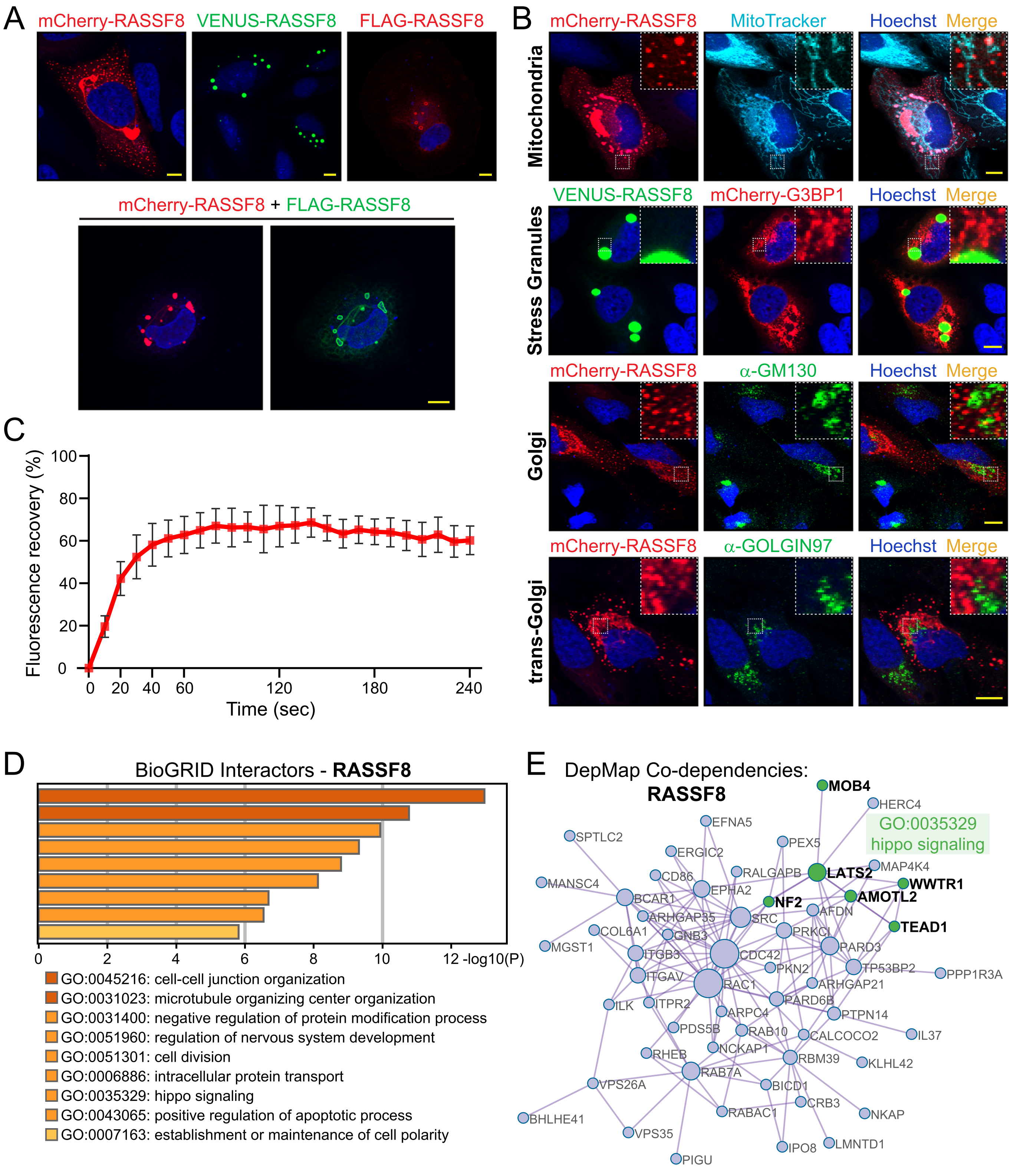
A
mCherry-RASSF8
VENUS-RASSF8
FLAG-RASSF8
mCherry-RASSF8 + FLAG-RASSF8
B
mCherry-RASSF8
MitoTracker
Hoechst Merge
Mitochondria
VENUS-RASSF8
mCherry-G3BP1
Stress Granules
α-GM130
Golgi
α-GOLGIN97
trans-Golgi
C
Fluorescence recovery (%)
Time (sec)
D
BioGRID Interactors - RASSF8
-log10(P)
GO:0045216: cell-cell junction organization
GO:0031023: microtubule organizing center organization
GO:0031400: negative regulation of protein modification process
GO:0051960: regulation of nervous system development
GO:0051301: cell division
GO:0006886: intracellular protein transport
GO:0035329: hippo signaling
GO:0043065: positive regulation of apoptotic process
GO:0007163: establishment or maintenance of cell polarity
E
DepMap Co-dependencies:
RASSF8
GO:0035329
hippo signaling
MOB4
HERC4
EFNA5
SPTLC2
PEX5
ERGIC2
MAP4K4
MANSC4
CD86
RALGAPB
LATS2
WWTR1
EPHA2
BCAR1
NF2
AMOTL2
ARHGAP35
SRC
AFDN
TEAD1
COL6A1
GNB3
PRKCI
MGST1
ITGB3
CDC42
PARD3
PKN2
TP53BP2
PPP1R3A
ITGAV
RAC1
ARHGAP21
ILK
ITPR2
PARD6B
ARPC4
PTPN14
PDS5B
RAB10
CALCOCO2
IL37
RHEB
NCKAP1
RBM39
KLHL42
VPS26A
RAB7A
BICD1
CRB3
BHLHE41
RABAC1
NKAP
VPS35
IPO8
LMNTD1
PIGU

 © The Author(s)

Figure EV3. Characterization of RASSF8 puncta in cells.

(**A**) Representative confocal microscopy images of U2OS cells expressing mCherry-RASSF8, VENUS-RASSF8, or FLAG-RASSF8 (top row). Also shown are cells co-expressing mCherry-RASSF8 with FLAG-RASSF8 followed by immunostaining with anti-FLAG (bottom row). Nuclei were visualized with Hoechst (blue). Scale bars represent 10 µm. (**B**) Fluorescence images of U2OS cells transiently expressing mCherry-RASSF8. These were stained markers for mitochondria (MitoTracker), golgi (anti-GM130), or trans-golgi (GOLGIN97). Alternatively, cells expressing VENUS-RASSF8 were co-transfected with a marker for stress granules (mCherry-G3BP1). Nuclei are in blue. Scale bars represent 10 µm. (**C**) Quantification of FRAP recovery as a fraction of the initial fluorescence intensity of mCherry-RASSF8. Data presented as mean ± SD, $n = 10$ biological replicates. (**D**) Gene Ontology (GO) analysis of all RASSF8 interactors found in the BioGRID database, conducted using Metascape. Enriched GO terms are listed at bottom. Bars are colored by increasing *P* values as determined by Metascape (using a hypergeometric test and Benjamini–Hochberg *p* value correction algorithm). (**E**) Network analysis of RASSF8 genetic interactors as identified by the CRISPR-based cancer dependency map (DepMap). Enriched networks were identified by Metascape, and include multiple proteins related to Hippo signaling.

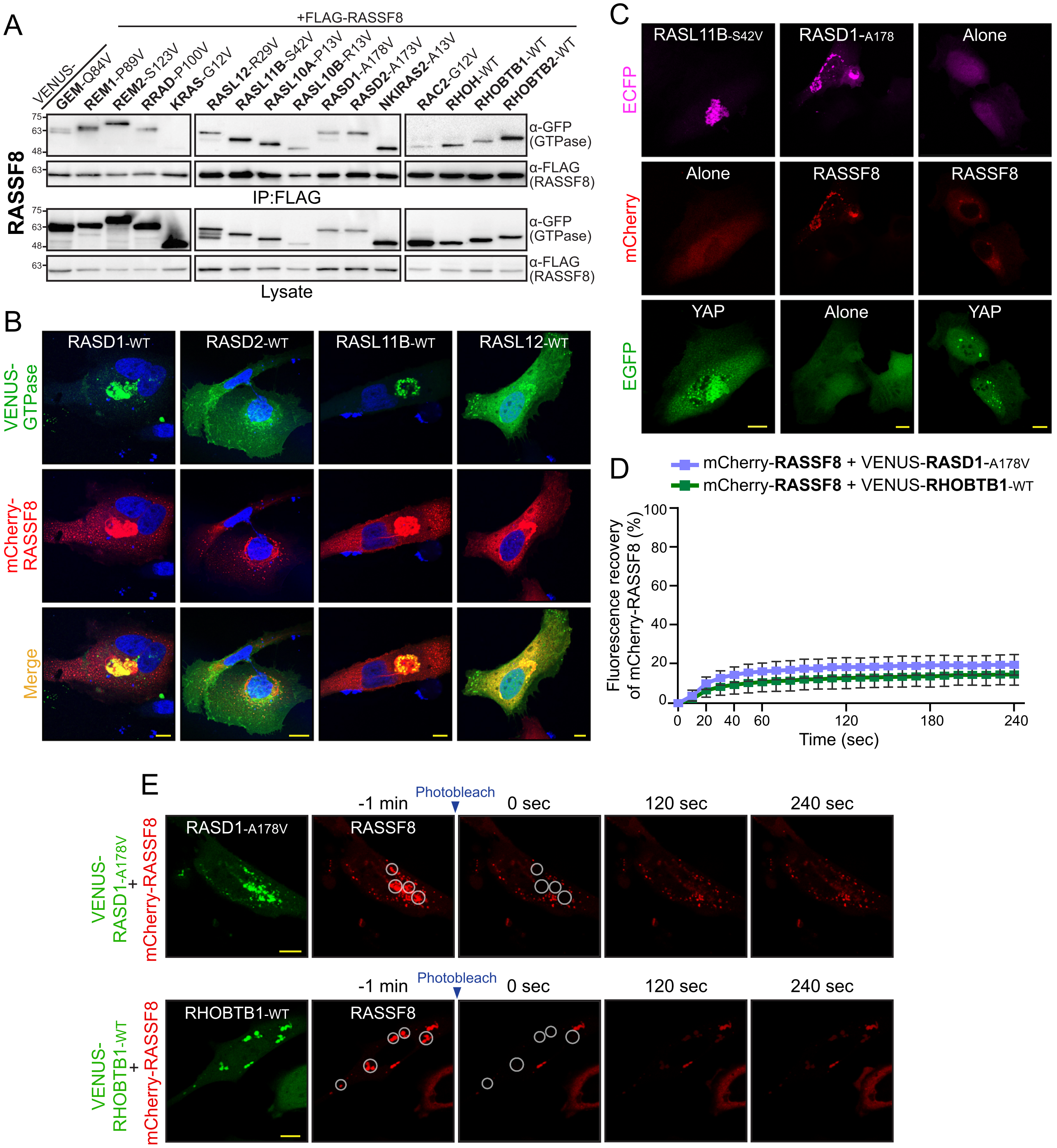
A
+FLAG-RASSF8
VENUS-
GEM-Q84V
REM1-P89V
REM2-S123V
RRAD-P100V
KRAS-G12V
RASL12-R29V
RASL11B-S42V
RASL10A-P13V
RASL10B-R13V
RASD1-A178V
RASD2-A173V
NKIRAS2-A13V
RAC2-G12V
RHOH-WT
RHOBTB1-WT
RHOBTB2-WT
RASSF8
75
63
48
α-GFP (GTPase)
α-FLAG (RASSF8)
IP:FLAG
Lysate
B
RASD1-WT
RASD2-WT
RASL11B-WT
RASL12-WT
VENUS-GTPase
mCherry-RASSF8
Merge
C
RASL11B-S42V
RASD1-A178
Alone
RASSF8
YAP
ECFP
mCherry
EGFP
D
mCherry-RASSF8 + VENUS-RASD1-A178V
mCherry-RASSF8 + VENUS-RHOBTB1-WT
Fluorescence recovery of mCherry-RASSF8 (%)
100
80
60
40
20
0
20
40
60
120
180
240
Time (sec)
E
-1 min
Photobleach
0 sec
120 sec
240 sec
RASD1-A178V
RASSF8
RHOBTB1-WT
VENUS-RASD1-A178V + mCherry-RASSF8
VENUS-RHOBTB1-WT + mCherry-RASSF8

Figure EV4. Validation of RASSF8 interactions with RAS subfamily GTPases.

(**A**) Co-IP of 15 candidate RAS and RHO subfamily GTPases following co-expression of the VENUS-tagged, mutationally activated variants with FLAG-RASSF8 in HEK 293T cells. KRAS-G12V does not interact with RASSF8 and served as a negative control. RHOH and the RHOBTB proteins are pseudoGTPases and were assayed as wild-type. (**B**) Co-distribution of mCherry-RASSF8 and four wild-type candidate VENUS-GTPases following co-expression in HeLa cells. Nuclei are in blue (Hoechst). Scale bars represent 10 µm. (**C**) Controls for confocal images in U2OS cells co-expressing EGFP-YAP, ECFP-GTPases, and mCherry-RASSF8. No bleed through was observed in corresponding channels when EGFP, ECFP, or mCherry tags were expressed alone. Scale bars represent 10 µm. (**D**) Quantification of FRAP recovery as a fraction of the initial fluorescence intensity of mCherry-RASSF8 co-expressed with either RASD1-A178V or RHOBTB1-WT. Data presented as mean ± SD, $n = 10$ biological replicates. (**E**) Representative images from time-lapse live-cell imaging of U2OS cells expressing mCherry-RASSF8 with VENUS-tagged RASD1-A178V or RHOBTB1-WT. Encircled mCherry-RASSF8 puncta were photobleached and exhibited notably reduced fluorescence recovery. Time is indicated above panes and scale bar represents 10 µm.

© The Author(s)

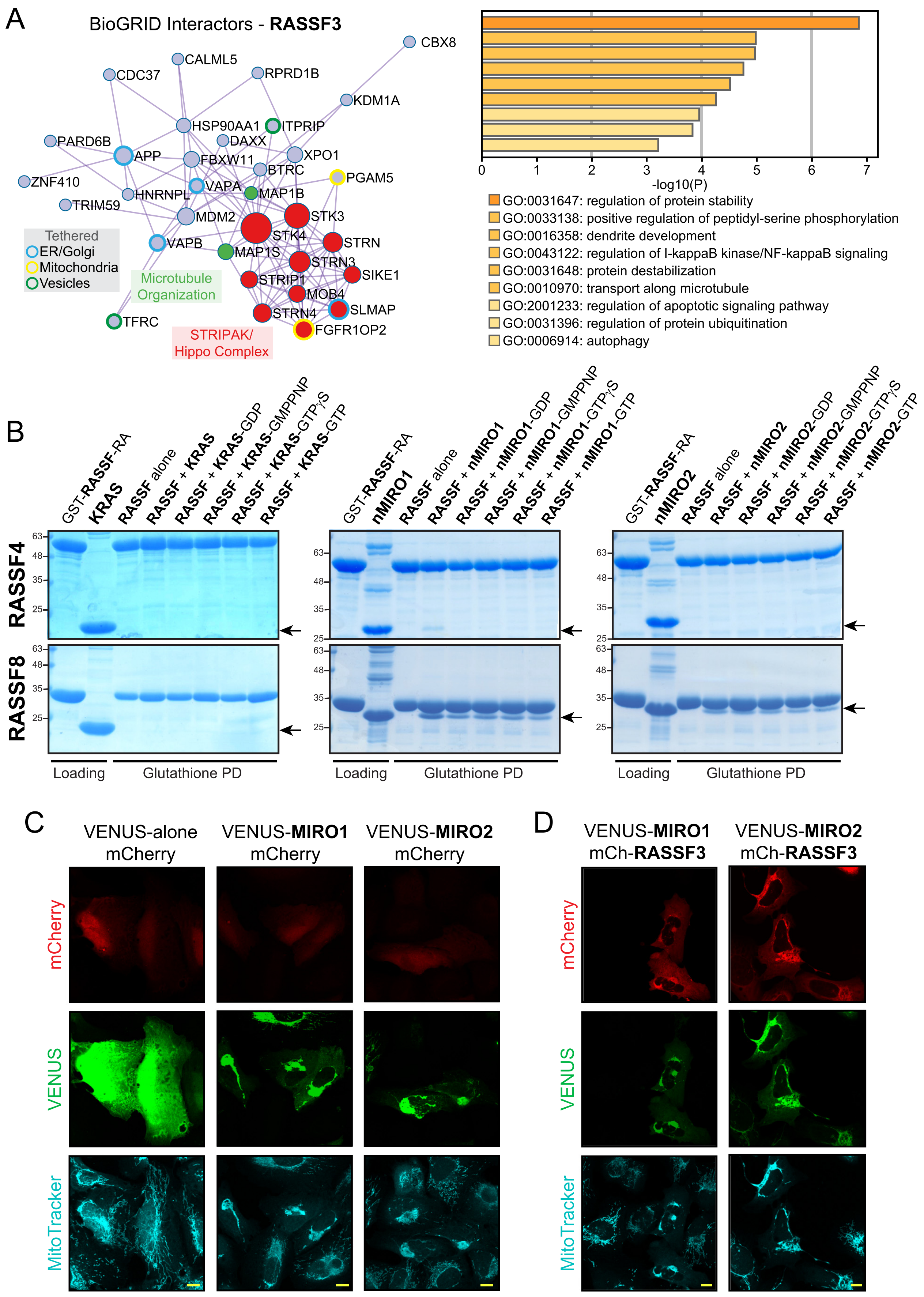
A
BioGRID Interactors - RASSF3
CBX8
CALML5
CDC37
RPRD1B
KDM1A
HSP90AA1
ITPRIP
PARD6B
DAXX
APP
FBXW11
XPO1
BTRC
PGAM5
ZNF410
HNRNPL
VAPA
MAP1B
TRIM59
MDM2
STK3
STK4
STRN
VAPB
MAP1S
STRN3
SIKE1
STRIP1
MOB4
SLMAP
STRN4
TFRC
FGFR1OP2
Tethered
ER/Golgi
Mitochondria
Vesicles
Microtubule Organization
STRIPAK/ Hippo Complex
-log10(P)
GO:0031647: regulation of protein stability
GO:0033138: positive regulation of peptidyl-serine phosphorylation
GO:0016358: dendrite development
GO:0043122: regulation of I-kappaB kinase/NF-kappaB signaling
GO:0031648: protein destabilization
GO:0010970: transport along microtubule
GO:2001233: regulation of apoptotic signaling pathway
GO:0031396: regulation of protein ubiquitination
GO:0006914: autophagy
B
GST-RASSF-RA
KRAS
RASSF alone
RASSF + KRAS
RASSF + KRAS-GDP
RASSF + KRAS-GMPPNP
RASSF + KRAS-GTPγS
RASSF + KRAS-GTP
nMIRO1
RASSF + nMIRO1
RASSF + nMIRO1-GDP
RASSF + nMIRO1-GMPPNP
RASSF + nMIRO1-GTPγS
RASSF + nMIRO1-GTP
nMIRO2
RASSF + nMIRO2
RASSF + nMIRO2-GDP
RASSF + nMIRO2-GMPPNP
RASSF + nMIRO2-GTPγS
RASSF + nMIRO2-GTP
RASSF4
RASSF8
Loading
Glutathione PD
C
VENUS-alone mCherry
VENUS-MIRO1 mCherry
VENUS-MIRO2 mCherry
D
VENUS-MIRO1 mCh-RASSF3
VENUS-MIRO2 mCh-RASSF3
mCherry
VENUS
MitoTracker

 © The Author(s)

Figure EV5. Interaction between RASSF3 and the mitochondrial GTPases MIRO1/2.

(**A**) Enrichment analysis of RASSF3 interactors from the BioGRID database. Metascape identified the STIPAK complex and numerous proteins tethered to organelles in the dataset (left). Enriched GO terms are listed at right. Bars are colored by increasing *P* values as determined by Metascape (using a hypergeometric test and Benjamini–Hochberg *p* value correction algorithm). (**B**) In vitro mixing assays using recombinantly purified, GST-tagged RA domains from RASSF4 (top) and RASSF8 (bottom) to precipitate the small GTPase KRAS (left), nGTPase domain of MIRO1 (nMIRO1, middle) or nGTPase domain of MIRO2 (nMIRO2, right) on glutathione beads. Arrow indicates where the precipitated GTPases should appear if bound. GTPases were pre-loaded with the nucleotides indicated at top. (**C**) Representative confocal microscopy images of collapsed mitochondrial networks in U2OS cells expressing VENUS-MIRO1 or VENUS-MIRO2 with mCherry alone. Scale bars represent 10 µm. (**D**) Confocal images of collapsed mitochondrial networks in U2OS cells co-expressing VENUS-MIRO1 or VENUS-MIRO2 with mCherry-RASSF8. Scale bars represent 10 µm.

© The Author(s)
